# Organocatalytic asymmetric *N*-sulfonyl amide C-N bond activation to access axially chiral biaryl amino acids

**DOI:** 10.1038/s41467-020-14799-8

**Published:** 2020-02-19

**Authors:** Guanjie Wang, Qianqian Shi, Wanyao Hu, Tao Chen, Yingying Guo, Zhouli Hu, Minghua Gong, Jingcheng Guo, Donghui Wei, Zhenqian Fu, Wei Huang

**Affiliations:** 1 Key Laboratory of Flexible Electronics & Institute of Advanced Materials, Jiangsu National Synergetic Innovation Center for Advanced Materials, Nanjing Tech University, 30 South Puzhu Road, Nanjing, 211816 China; 2 College of Chemistry, Zhengzhou University, 100 Science Avenue, Zhengzhou, Henan Province 450001 China; 3 Shaanxi Institute of Flexible Electronics (SIFE), Northwestern Polytechnical University (NPU), 127 West Youyi Road, Xi’an, 710072 China

**Keywords:** Asymmetric catalysis, Organocatalysis, Synthetic chemistry methodology

## Abstract

Amides are among the most fundamental functional groups and essential structural units, widely used in chemistry, biochemistry and material science. Amide synthesis and transformations is a topic of continuous interest in organic chemistry. However, direct catalytic asymmetric activation of amide C-N bonds still remains a long-standing challenge due to high stability of amide linkages. Herein, we describe an organocatalytic asymmetric amide C-N bonds cleavage of *N*-sulfonyl biaryl lactams under mild conditions, developing a general and practical method for atroposelective construction of axially chiral biaryl amino acids. A structurally diverse set of axially chiral biaryl amino acids are obtained in high yields with excellent enantioselectivities. Moreover, a variety of axially chiral unsymmetrical biaryl organocatalysts are efficiently constructed from the resulting axially chiral biaryl amino acids by our present strategy, and show competitive outcomes in asymmetric reactions.

## Abstract (web summary)

Asymmetric activation of amide bonds remains a challenge due to the high stability of amide linkages. Here, the authors show an organocatalytic asymmetric C-N bond cleavage of N-sulfonyl biaryl lactams under mild conditions, to access axially chiral biaryl amino acids.

## Introduction

**Fig. 1:**
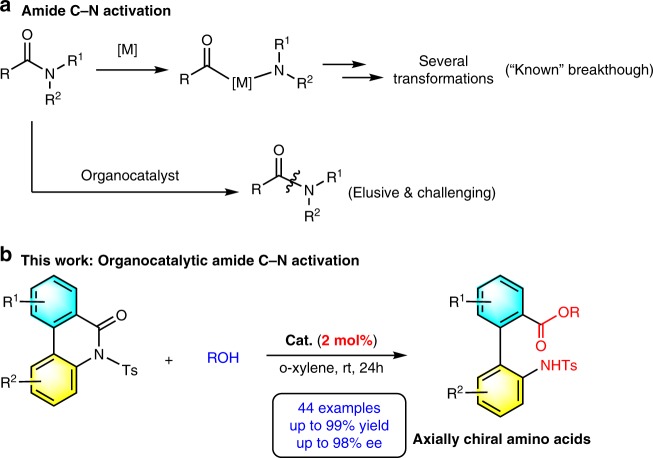
Approaches for amide C–N bond activation. **a** Metal-catalyzed amide C–N activation. **b** Organocatalytic asymmetric *N*-sulfonyl amide bond activation.

As an important functional group, amides are essential structural units of peptides, proteins, and enzymes, and they have a wide variety of applications in chemistry, biochemistry, and material science^1^. Amide synthesis and transformations have been extensively studied in organic chemistry^1^. However, direct activation of unreactive amide C–N bonds remains challenging owing to the high stability of amide linkages^1,2^. A seminal study by Garg, Houk, and coworkers^3^, described nickel-catalyzed conversion of highly stable amides to esters through insertion of nickel into a typically unreactive amide C–N bond (Fig. 1). This work was identified as one of “Top of research of 2015” by ACS C&EN for its significant achievement in amide synthesis and transformations^4,5^. This catalytic strategy has been further explored by Garg^6–13^, Szostak^14–20^, and others^21–29^. Notably, amide bonds commonly need to be activated by electron-withdrawing groups, such as t-butoxycarbonyl (Boc), trifluoromethanesulfonyl (Tf), p-toluenesulfonyl (Ts), and cyclodicarbonyl group^30–32^. Despite these advances, direct organocatalytic activation of amide C–N bond, especially in an enantioselective manner, has yet to be achieved.

Among the most important molecules in nature, chiral amino acids have a central role in life and widespread applications in pharmaceutical, chemical, and food industries^33,34^. Unlike well-studied centrally chiral amino acids^35–37^, there have been fewer reports on the synthesis and application of axially chiral amino acids (amino acids with an axially chiral scaffold, exemplified as **3a** and its X-ray crystal structure, see below), although their derivatives are frequently found in natural products and bioactive compounds^38–42^. The lack of efficient synthetic methods and limited number of examples have considerably impeded applications.

Catalytic asymmetric ring opening of biaryl lactams (such as **1a**, **1b**, and **1c** with the twisted structure of naphthyl phenyl scaffolds) is a straightforward and efficient method for atroposelective construction of axially chiral amino acids^43^. However, organocatalytic ring opening of biaryl lactams for atroposelective construction of axially chiral amino acids remains underexplored. This strategy presents some challenges, including (i) increasing the reactivity of unreactive amide bonds, (ii) choosing a proper organocatalyst to both activate unreactive amide groups and other reactants, and (iii) ensuring excellent enantioselectivity of the desired products. On the basis of Bringmann’s pioneering work^41,42^, and the recent advance in constructing axially chiral backbones by ring opening of conformationally labile bridged biaryls^44–48^, we envisaged that the *N*-electron-withdrawing group configurationally labile biaryl lactams with an inherent torsional strain might act as suitable substrates for activation of amide C–N bonds, promoted by a bifunctional organocatalyst. According to our understanding of organocatalysis^49–54^, we herein present a strategy for direct organocatalytic asymmetric activation of *N*-sulfonyl amide C–N bonds promoted by a bifunctional organocatalyst under mild reaction conditions. We developed a straightforward catalytic asymmetric method for the synthesis of a structurally diverse set of axially chiral biaryl amino acids. Moreover, a variety of axially chiral unsymmetrical biaryl organocatalysts are efficiently constructed from the resulting axially chiral biaryl amino acids by our present strategy, and they show good outcomes in asymmetric reactions.

## Results

### Reaction optimization

We initially attempted the synthesis with biaryl lactams containing a naphthyl phenyl scaffold (*N*-Boc **1a** or *N*-Cbz **1b**) and benzyl alcohol **2a** in the presence of the bifunctional thiourea catalyst^55–60^ **A** in dichloromethane (DCM) at room temperature. Unfortunately, no reaction occurred, perhaps because the amide bond resisted breakage, even when activated by Boc or Cbz (entries 1–2). Next, we used a biaryl lactam **1c** with a stronger electron-withdrawing substituent (Ts) under the same reaction conditions. Gratifyingly, the desired axially chiral biaryl amino ester **3a** was obtained in 92% yield with 75% ee (entry 3). This result confirmed the feasibility of organocatalytic amide C–N activation. Subsequent solvent screening revealed that o-xylene was most effective, providing the desired product **3a** in up to 99% yield with 92% ee (entries 4–9). We investigated other bifunctional organocatalysts **B**, **C**, and **D**. Catalyst **D** was the best catalyst for this reaction, and gave the desired product **3a** in up to 99% yield with up to 97% ee (entry 12). Surprisingly, a 2 mol% catalyst loading was sufficient for this transformation (entry 13). When we lowered the catalyst loading to 1 mol%, the product yield decreased considerably (entry 14). In the absence of catalyst, no product was found (entry 15).

### Substrate scope

With acceptably optimized conditions in hand (Table 1, entry 13), we next evaluated the scope of the reaction for alcohol substrates with the use of biaryl lactam **1c** as a model substrate (Table 2). Benzyl alcohols bearing substituents with a variety of electronic and steric properties on the aromatic ring were well tolerated, generating the corresponding axially chiral biaryl amino esters **3a**–**3f** in excellent yields (92–99%) with excellent enantioselectivities (92–96% ee). Both 2-heteroaryls (such as furyl, thienyl, and pyridinyl), methanol and tryptophol, also worked efficiently, affording the corresponding products **3g**–**j** in excellent yields and enantioselectivities. Notably, methanol, the simplest alcohol, was also a suitable substrate for this transformation, resulting in the formation of the desired product **3k** in 99% yield with 96% ee. Pleasingly, several other groups, such as CF_3_, a three-membered ring, double and triple bonds, trimethylsilyl (TMS), ether, bromide, acetal group, and even NHBoc groups, were well tolerated, and the corresponding axially chiral biaryl amino esters **3n**–**3w** were generated in excellent yields (93–99%) with good-to-excellent enantioselectivities (91–98% ee). Interestingly, an additional activated keto carbonyl group in the alcohol did not influence the chemical yield, but slightly decreased the enantioselectivity (**3x**). (*S*)-1-(2-hydroxynaphthalen-1-yl)naphthalen-2-ol ((*S*)-BINOL)-derived alcohol or l-glycerol–acetonide also were suitable substrates, affording the products **3y** & **3z** in excellent yields and stereoselectivities. Besides alcohols, other nucleophiles, such as phenol, thiophenol, benzyl mercaptan, diethyl malonate, nitromethane, and amine, did not give satisfying outcomes under the current conditions (see SI for details).

**Table 1:** Condition optimization.


Entry^a^	Cat.	Solvent	Yield (%)^b^	ee (%)^c^
1^d^	A	DCM	n.r.	–
2^e^	A	DCM	n.r.	–
3	A	DCM	93	75
4	A	CHCl_3_	96	88
5	A	CCl_4_	96	91
6	A	DCE	86	73
7	A	Toluene	99	91
8	A	Mesitylene	99	90
9	A	o-Xylene	99	92
10	B	o-Xylene	98	91
11	C	o-Xylene	95	−82
12	D	o-Xylene	99	97
13^f^	D	o-Xylene	99	97
14^g^	D	o-Xylene	70	97
15	–	o-Xylene	n.r.	–

^a^Standard condition: **1c** (0.1 mmol), **2a** (1.2 equiv.), catalyst (10 mol%), and solvent (0.2 M), rt, 24 h.

^b^Yield of the isolated product after column chromatography. n.r. = no reaction.

^c^Determined by chiral HPLC, %ee = (R−S)/(R + S) * 100. Absolute configuration of the product was determined via X-ray of **3a**.

^d^**1a** was used.

^e^**1b** was used.

^f^2 mol% catalyst was used.

^g^1 mol% catalyst was used. Boc = t-butoxycarbonyl. Cbz = carbobenzyloxy. Ts = p-toluenesulfonyl.

**Table 2:**
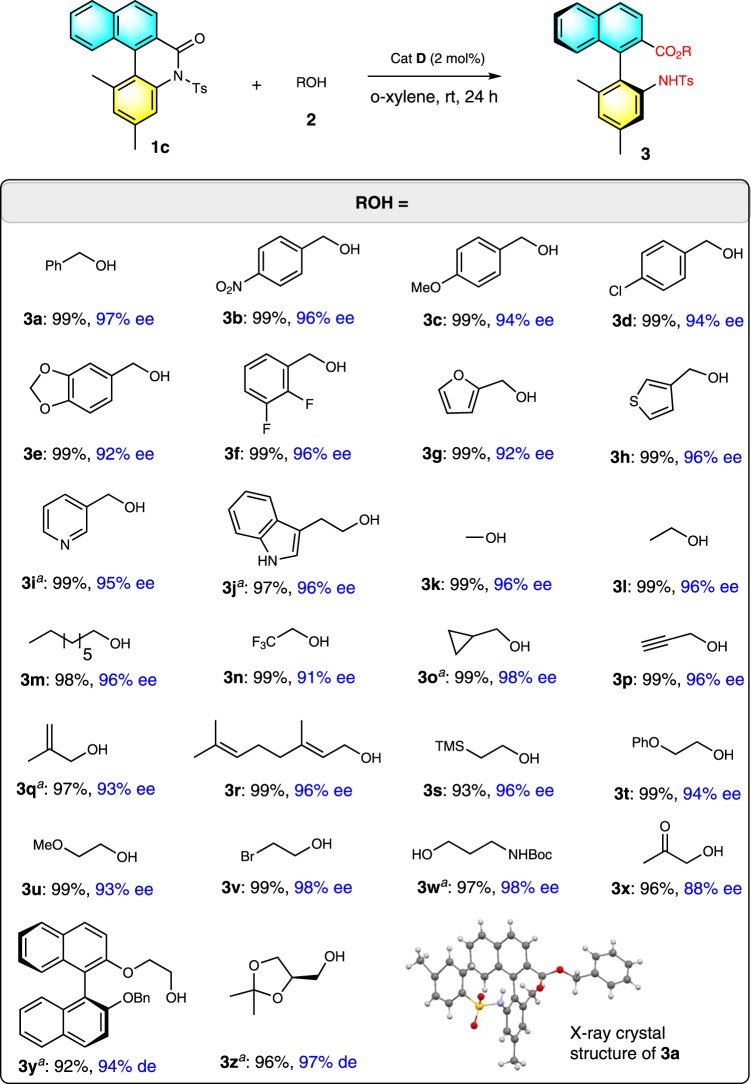
Substrate scope.

Reaction conditions as in Table 1, entry 13; yields (after SiO_2_ chromatography purification) were based on biaryl lactam **1**.

*TMS* trimethylsilyl

^a^5 mol% catalyst was used.

To further demonstrate the generality of this catalytic enantioselective method for the synthesis of axially chiral biaryl amino acids, the scope of biaryl lactam **1** was investigated with the use of benzyl alcohol **2a** as a model substrate (Table 3). *N*-sulfonyl biaryl lactam **1** with a variety of biaryl scaffolds, such as naphthyl phenyl, biphenyl, phenyl naphthyl, and binaphthyl, was evaluated. Reactions with all substrates proceeded smoothly to yield axially chiral biaryl amino acids **3aa**–**3ao** in good-to- excellent yields (92–99%) with good-to-excellent enantioselectivities (79–96% ee). Notably, Ts was easily replaced with other sulfonyl groups, such as phenylsulfonyl (Bs), methylsulfonyl (Ms), cyclohexyl sulfonyl (Cys), and o-nitrobenzenesulfonyl (Ns). The variety of the resulting axially chiral biaryl amino acids will likely have a range of potential applications.

**Table 3:**
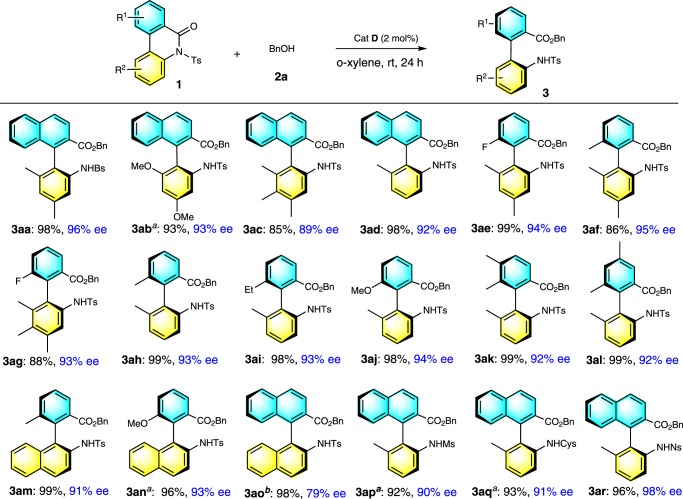
Substrate scope.

Reaction conditions as in Table 1, entry 13; yields (after SiO_2_ chromatography purification) were based on biaryl lactam 1.

*Bs* phenylsulfonyl, *Ms* methylsulfonyl, *Cys* cyclohexyl sulfonyl, *Ns* o-nitrobenzenesulfonyl.

^a^5 mol% catalyst was used.

^b^Reaction conditions as in Table 1, entry 9.

Notably, product **3a** is so stable that it could be stirred at 140 °C in o-xylene for 4 h without any loss in ee. For further investigation on the racemization of compound **3** (**3k** as an example), see Supplementary Table 3. Meanwhile, DFT calculations indicated that the energy barrier via transition state **TS3** for the transformation from **PS** to **PR** is 37.4 kcal/mol depicted in Supplementary Fig. 3, indicating that the racemization between them is very hard to happen even for the heating condition, and this phenomenon is in agreement with the experimental observation.

### Mechanistic studies

**Fig. 2:**
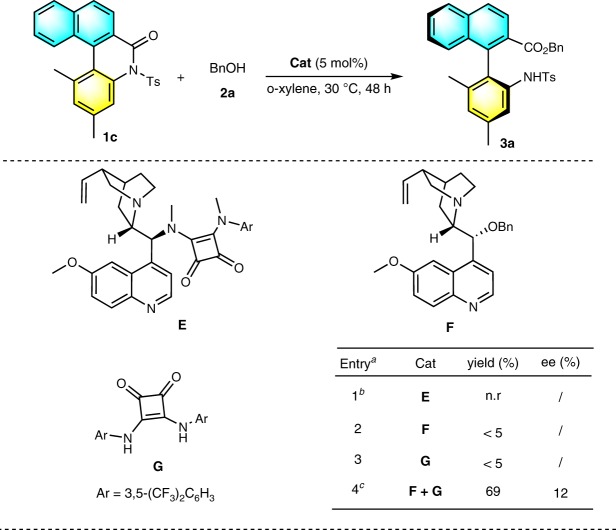
Mechanism study. ^a^Standard condition: **1c** (0.1 mmol), BnOH (1.2 equiv.), catalyst (5 mol%), and o-xylene (0.2 M), rt, 48 h. ^b^n.r = no reaction. ^c^5 mol% **F** and 5 mol% **G** were simultaneously added.

To probe the reaction pathway, control experiments were performed as shown in Fig. 2. No reactions occurred when catalyst **E**, the *N*,*N’*-dimethyl analog of catalyst **D**, was used in this reaction. This result suggested that the H-bond donor motif of the catalyst played a central role in this transformation. When catalysts **G** & **F**, which are two moieties of the optimal catalyst **D**, were investigated separately, a yield of <5% was achieved. When these were used together, product **3a** was generated in 69% yield with 12% ee. These results confirmed that the synergistic effects of the bifunctional moieties of the catalyst controlled this transformation.

**Fig. 3:**
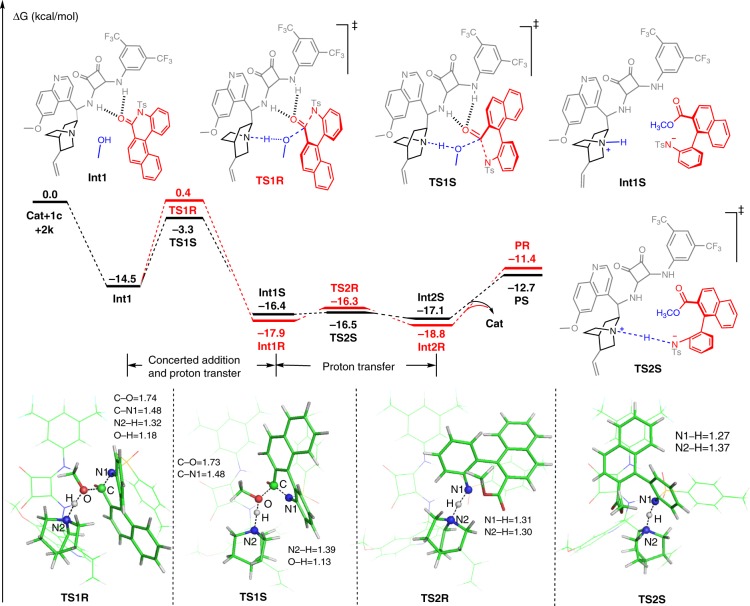
Reaction energy profile. Stereoselective reaction pathways calaulated at the M06-2X-GD3/6-311++G(2d, 2p)/IEF-PCM_o-xylene_//M06-2X/6-31G(d, p)/IEF-PCM_o-xylene_ level.

To understand the mechanism, we performed DFT calculations. The catalytic reaction contains two processes, namely oxygen of **2k** attacks the carbonyl carbon of **1c** in a nucleophilic manner coupled with a six-membered ring opening and proton transfer from **2k** to the catalyst through transition states **TS1R** & **TS1S**, and other proton transfer from the catalyst to N^–^ for formation of **PR** & **PS** via transition states **TS2R** & **TS2S**. According to the energy profiles depicted in Fig. 3, the first step is identified as the stereoselectivity-determining step, and the *S*-configuration product **PS** should be the main product, which is in agreement with the experimental results. The tunneling effect of the proton in **TS1R** (with imaginary frequency as −664.05 cm^−1^) and **TS1S** (with imaginary frequency as −349.36 cm^−1^) was computed, and the tiny difference does not affect the energy differences between the two stereoselective transition states. In addition, the MeO···C and N···H–OMe distances are 1.74 Å and 1.32 Å in **TS1R**, while these are 1.73 Å and 1.39 Å in **TS1S**, indicating that the attacks of the O atom in methanol should be the dominating process in this step. Moreover, the Gibbs free energies of **PR** and **PS** are −11.4 and −12.7 kcal/mol lower than that of reactants **1c** + **2k,** respectively, demonstrating that the entire reaction should be an exothermic process.

**Fig. 4:**
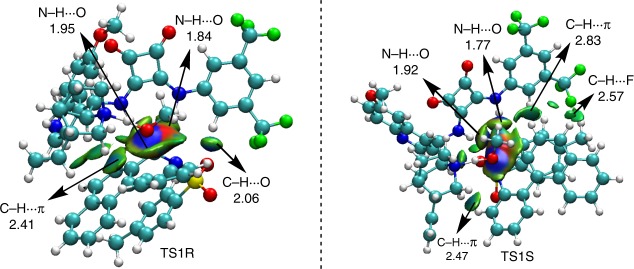
NCI analysis for stereocontrol of transition states TS1R and TS1S. Blue, green, and red coloration represent strong interaction, weak interaction, and steric hindrance, respectively.

We performed further non-covalent interaction (NCI) analyses to explore the origin of stereoselectivity. As illustrated in Fig. 4, there are two N–H⋯O (1.95 and 1.84 Å), one C–H⋯O (2.06 Å), and one C–H⋯π (2.41 Å) interaction in **TS1R,** and two N–H⋯O (1.77 and 1.92 Å), two C–H⋯π (2.47 and 2.83 Å), and one C–H⋯F (2.57 Å) interaction in **TS1S**. The strength and number of hydrogen-bond interactions increased in the *S*-isomer transition state **TS1S**, which resulted in a lower energy barrier via the more stable transition state **TS1S**. Further details of these computations can be found in the SI.

### Synthetic transformations

**Fig. 5:**
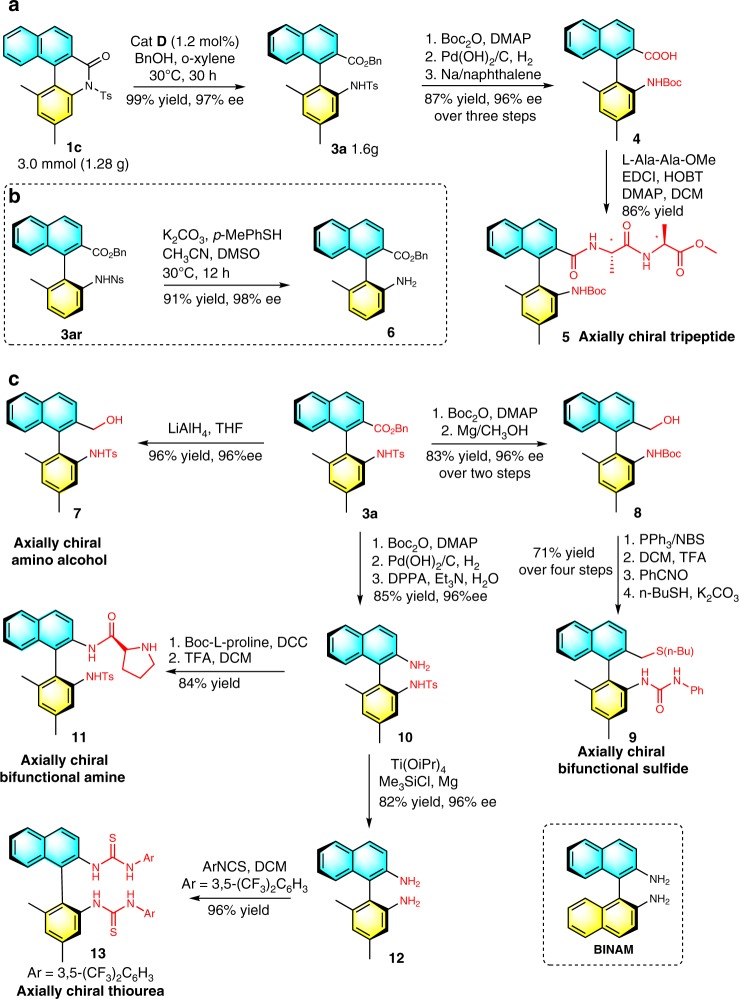
Synthetic transformations. **a** Gram-scale reaction, synthesis of *N*-Boc axially chiral biaryl amino acid, and tripeptide. **b** Synthesis of unprotected axially chiral biaryl amino esters. **c** Synthesis of axially chiral organocatalysts.

To show the practical utility of our present strategy, a gram-scale reaction was performed (Fig. 5a). In the presence of only 1.2 mol% of bifunctional squaramide catalyst **D**, a gram-scale reaction of a biaryl lactam **1c** with a benzyl alcohol proceeded smoothly to afford the axially chiral desired product **3a** in up to 99% yield with 97% ee.

**Fig. 6:**
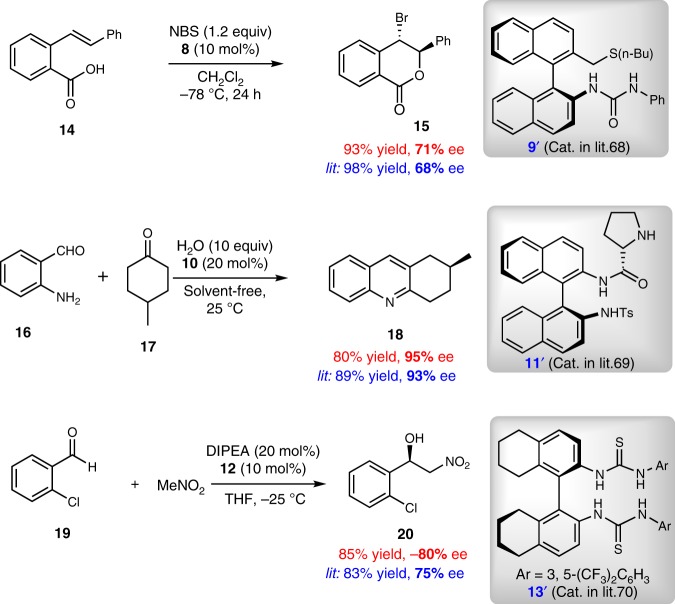
Product application. Initial attempts for unsymmetrical axially chiral bifunctional organocatalysts.

Subsequently, further synthetic transformations of the resulting axially chiral biaryl amino ester **3** were conducted as shown in Fig. 5. *N*-Boc axially chiral biaryl amino acid **4** was obtained in excellent yield without loss of enantiopurity through debenzylation and detosylation of axially chiral amino ester **3a**. Notably, tripeptide **5**, containing both centrally and axially chiral centers, was easily prepared in excellent yield from **4** via an amide formation process (Fig. 5a). A protecting group (i.e., Ns) on the nitrogen atom was easily removed to give the unprotected amine product **6** in 91% yield with 98% ee (Fig. 5b). In addition, the ester moiety was retained under the reaction conditions. As shown in Fig. 5c, reduction of **3a** was readily realized by treatment with LiAlH_4_ in THF at room temperature, leading to the formation of axially chiral biaryl amino alcohol **7** in 96% yield with 96% ee. Notably, the axially chiral BINAM derivative **12** was elegantly prepared from **3a** without any loss of ee. Axially chiral biaryl compounds have been identified as core structural motifs in many common chiral ligands^61–64^ and organocatalysts^65–67^ for asymmetric catalysis. Notably, well-established popular axially chiral organocatalysts often rely on symmetrical axially chiral scaffold. Starting from our axially chiral diaryl product **3a**, a variety of unsymmetrical axially chiral organocatalysts, such as axially chiral bifunctional sulfide **9**, axially chiral bifunctional amine **11**, and axially thiourea **13** were efficiently prepared. Furthermore, these unsymmetrical axially chiral organocatalysts were investigated in asymmetric reactions (Fig. 6). To our delight, these organocatalysts gave comparable or even better outcomes in terms of yield and ee than the previous symmetric organocatalysts under the same reaction conditions^68–70^. These initial results indicate that unsymmetrical axially chiral biaryl compounds synthesized in our present strategy have great potential as organocatalysts and ligands in asymmetric catalysis.

## Discussion

In summary, we have addressed direct organocatalytic asymmetric activation of amide C–N bond under mild conditions, and developed atroposelective synthesis of a structurally diverse set of axially chiral biaryl amino acids in high yields with excellent enantioselectivities. This general and practical strategy features mild reaction conditions, a broad substrate scope, and excellent functional group tolerance. Mechanism studies and DFT calculations demonstrated that the cooperative effects of the bifunctional moieties of the Cinchona-alkaloid-derived thiourea catalyst ensure the transformation with excellent yields and enantioselectivities. A variety of unsymmetrical axially chiral bifunctional organocatalysts have also been efficiently constructed from the resulting axially chiral biaryl amino acids. Further investigations and exploration of this catalytic process are underway in our laboratory.

## Methods

### Synthesis of 3

To a suspension of starting material 1 (0.10 mmol) and catalyst D (1.2 mg, 2 mol%) in o-xylene (0.5 mL, 0.20 M) were added the appropriate alcohols (0.12 mmol, 1.2 equiv.). The mixture was stirred for 24–48 h at 30 °C. Upon completion of the reaction (monitored by TLC), the reaction mixture was directly purified by column chromatography on silica gel to afford the desired product **3**.

## Supplementary information

Supplementary Information

## Data Availability

Experimental procedures, characterization of all new compounds, NMR and HPLC spectra, and computational details are available in the Supplementary Information. The source data underlying Figs. 3, 4 and Supplementary Figs. 3 are provided as a Source Data file. The X-ray crystallographic coordinates for structures of **3a** reported in this article have been deposited at the Cambridge Crystallographic Data Centre (CCDC), under deposition number CCDC 1894533. These data can be obtained free of charge from The Cambridge Crystallographic Data Centre via http://www.ccdc.cam.ac.uk/data_request/cif. All other data are available from the authors upon reasonable request.

## Source data

Source Data
