# Supplementary material for: Organocatalytic asymmetric N-sulfonyl amide C-N bond activation to access axially chiral biaryl amino acids
Source: Nat Commun. 2020 Feb 19;11:946. doi: 10.1038/s41467-020-14799-8 (PMC7031291; doi:10.1038/s41467-020-14799-8)
Supplement: Supplementary file 1 — Supplementary Information [file 41467_2020_14799_MOESM1_ESM.pdf]

Supplementary Information

Organocatalytic Asymmetric N-Sulfonyl Amide C-N Bond Activation to Access Axially Chiral Biaryl Amino Acids

Wang *et al.*

Supplementary Note 1

General Information

Chemicals were purchased as reagent grade and used without further purification. Solvents (THF, toluene) were distilled from appropriate drying agents prior to use. In addition, more solvents were purchased from commercial suppliers and dried over molecular sieves. Thin-layer chromatography (TLC) was performed using silica gel pre-coated glass plates (0.20 mm), which were visualized with a UV lamp (254 or 366 nm). Column chromatography (CC) was carried out using Tsingdao silica gel (60 Å, 200−300 mesh, particle size 0.040−0.063 mm). All reported yields, unless otherwise specified, refer to spectroscopically and chromatographically pure compounds. ^{1}H, ^{13}C, ^{19}F nuclear magnetic resonance (NMR) spectra were recorded on a Bruker spectrometer (400 MHz) in a suitable deuterated solvent. The solvent employed and respective measuring frequency is indicated for each experiment. Chemical shifts are reported with tetramethylsilane (TMS) serving as a universal reference of all nuclides. The resonance multiplicity is described as s (singlet), d (doublet), t (triplet), q (quartet), m (multiplet), and bs (broad singlet). All spectra were recorded at 298 K unless otherwise noted. The residual deuterated solvent signal relative to tetramethylsilane was used as the internal reference in ^{1}H NMR spectra ($CDCl_3$ δ 7.26), and are reported as follows: chemical shift in ppm (multiplicity, coupling constant J in Hz, number of protons). ^{13}C NMR spectra reported in ppm from tetramethylsilane (TMS) with the solvent resonance as the internal standard ($CDCl_3$ δ 77.2). High resolution mass spectrometry (HRMS) was performed on a Waters Q-TOF Permier Spectrometer. Optical rotations were determined with Jasco P-1030 polarimeter. Data are reported as follows: $[\alpha]_{D}^{rt}$, concentration (c; g/100 mL), and solvents. Enantiomeric excesses (ee) were determined on a Shimadzu LC-20AD HPLC system employing a chiral stationary phase column specified in the individual experiment, by comparing the samples with the appropriate racemic mixtures.

Supplementary Note 2. Preparation of Substrates.

Method A:

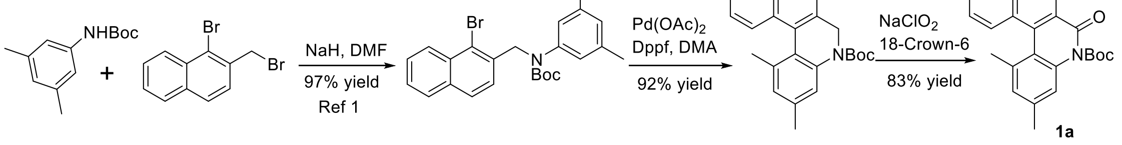

1b was prepared following method A

Method B:

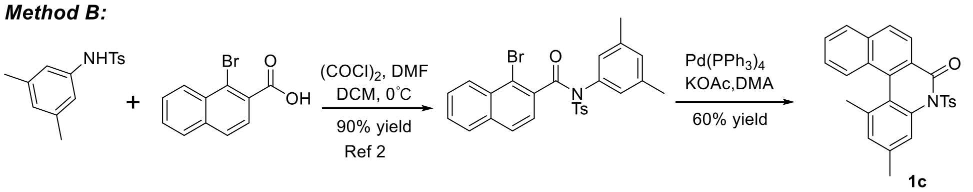

1d, 1e, 1f, 1h, 1i and 1j were prepared following method B

Method C:

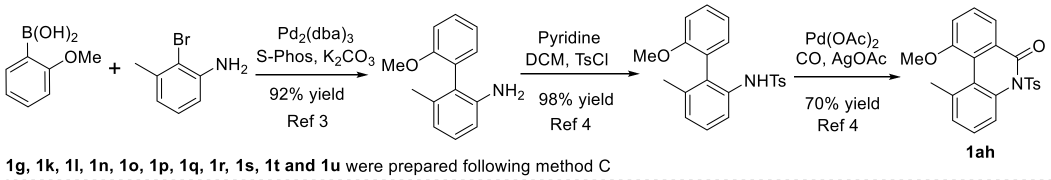

1g, 1k, 1l, 1n, 1o, 1p, 1q, 1r, 1s, 1t and 1u were prepared following method C

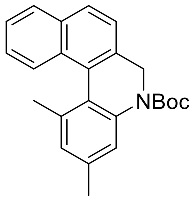

Tert-butyl 1, 3-dimethylbenzo[*k*]phenanthridine-5(6*H*)-carboxylate

An oven-dried flask with a stir bar was charged with $Pd(OAc)_2$ (0.125 mmol, 5 mol%), Dppf (0.125 mmol, 5 mol%), K_2CO_3 (5.0 mmol, 2.0 equiv) and starting materials (2.5 mmol, 1.0 equiv). The flask was evacuated and backfilled with N_2. DMA (anhydrous, 15 mL) was added via syringe, and the mixture stirred for 2 hours at 120 ℃. The reaction was quenched with H_2O and diluted with EtOAc. The aqueous phase was washed with EtOAc twice. The combined organic layer was washed with brine and then dried over Na_2SO_4. The resulting mixture was filtered, concentrated *in vacuo* and purified by column chromatography on silica gel using 5-10% EtOAc in hexanes as eluent to afford the desired product as a white solid (0.82 g, 2.3 mmol, 92% yield).

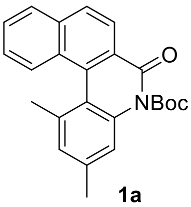

1a

Tert-butyl 1,3-dimethyl-6-oxobenzo[*k*]phenanthridine-5(6*H*)-carboxylate (1a)

To the solution of product obtained above (2.0 mmol, 1.0 equiv) in 1, 1, 2, 2-tetrachloroethane (10 mL) was added $NaClO_2$ (4.0 mmol, 2.0 equiv) and 18-crown-6 (5.0 mmol, 2.5 equiv) at r.t. After vigorous stirring for 12 hours, the reaction was quenched with $Na_2S_2O_3$ (aq) and diluted with DCM. The aqueous layer was extracted with DCM twice and the combined organic layer was dried over Na_2SO_4. The resulting mixture was filtrated, concentrated *in vacuo* and purified by column chromatography on silica gel using 5% EtOAc in hexanes as eluent to afford the desired product as a white solid (0.62 g, 1.66 mmol, 83% yield).

^{1}H NMR (400 MHz, $CDCl_3$) δ 8.40 (d, *J* = 8.4 Hz,1H), 7.91-7.97 (m, 3H), 7.62 (t, *J* = 8.0 Hz, 1H), 7.53 (t, *J* = 8.0 Hz, 1H), 7.08 (s, 1H), 6.93 (s, 1H), 2.48(s, 3H), 2.21(s, 3H), 1.73 (s, 9H); **^{13}C NMR (100 MHz, $CDCl_3$)** δ 160.17, 151.54, 139.06, 137.08, 135.54, 134.22, 129.21, 128.96, 128.20, 128.05, 127.95, 125.61, 124.78, 122.83, 115.99, 111.77, 86.28, 27.67, 24.37, 21.66; **HRMS (ESI)** Calcd for $C_{24}H_{23}NNaO_3^+$ $[M+Na]^+$ 396.1570; Found: 396.1571.

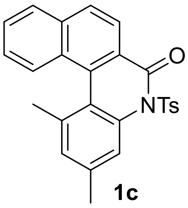

1c

1,3-Dimethyl-5-tosylbenzo[*k*]phenanthridin-6(5*H*)-one (1c)

An oven-dried flask with a stir bar was charged with $Pd(PPh_3)_4$ (0.30 mmol, 10 mol%), KOAc (6.0 mmol, 2.0 equiv) and N-tosylcarboxylic amide (3.0 mmol, 1.0 equiv). The flask was evacuated and backfilled with N_2. DMA (anhydrous, 15 mL) was added via syringe, and the mixture stirred for 12 hours at 100 ℃. After being cooled to room temperature, H_2O and EtOAc were added. The aqueous phase was washed with EtOAc twice. The combined organic layer was washed with brine and then dried over Na_2SO_4. The resulting mixture was filtered, concentrated *in vacuo* and purified by column chromatography on silica gel using EtOAc/hexanes/DCM (1:7:1) to afford the desired product as a white solid (0.76 g, 1.8 mmol, 60% yield).

^{1}H NMR (400 MHz, $CDCl_3$) δ 8.09 (d, *J* = 8.4 Hz, 3H), 7.87-7.94 (m, 3H), 7.79 (s, 1H), 7.64 (t, *J* = 7.2 Hz, 1H), 7.57 (t, *J* = 7.2 Hz, 1H), 7.30 (d, *J* = 8.4 Hz, 2H), 7.10 (s, 1H), 2.48 (s, 3H), 2.40 (s, 3H), 2.08 (s, 3H); **^{13}C NMR (100 MHz, $CDCl_3$)** δ 163.02, 145.03, 138.69, 136.66, 136.57, 135.80, 134.87, 134.18, 129.72, 129.63, 129.08, 128.80, 128.51, 128.46, 128.17, 128.14, 128.09, 126.17, 122.68, 120.09, 118.16,

24.16, 21.83, 21.69; **HRMS (ESI)** Calcd for $C_{26}H_{22}NO_3S^+$ $[M+H]^+$ 428.1315; Found: 428.1307.

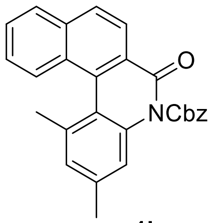

1b

Benzyl 1,3-dimethyl-6-oxobenzo[*k*]phenanthridine-5(6*H*)-carboxylate (1b)

^{1}H NMR (400 MHz, $CDCl_3$) δ 8.39 (d, *J* = 8.8 Hz, 1H), 7.93-7.96 (m, 2H), 7.89 (d, *J* = 8.4 Hz, 1H), 7.62 (t, *J* = 7.6 Hz, 1H), 7.50-7.56 (m, 3H), 7.38-7.45 (m, 3H), 7.04 (s, 1H), 6.69 (s, 1H), 5.59 (s, 2H), 2.34 (s, 3H), 2.18 (s,3H); **^{13}C NMR (100 MHz, $CDCl_3$)** δ 160.37, 153.45, 139.11, 137.04, 135.63, 135.34, 134.37, 134.19, 129.14, 129.09, 129.06, 128.92, 128.80, 128.27, 128.23, 128.19, 127.98, 125.72, 124.77, 122.90, 116.28, 112.33, 71.00, 24.32, 21.44; **HRMS (ESI)** Calcd for $C_{27}H_{21}NNaO_3^+$ $[M+Na]^+$ 430.1414; Found: 430.1417.

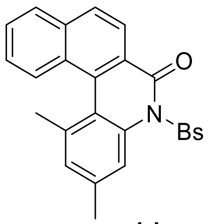

1d

1,3-Dimethyl-5-(phenylsulfonyl)benzo[*k*]phenanthridin-6(5*H*)-one (1d)

^{1}H NMR (400 MHz, $CDCl_3$) δ 8.20-8.22 (m, 2H), 8.08 (d, *J* = 8.8 Hz, 1H), 7.93 (t, *J* = 9.2 Hz, 2H), 7.88 (d, *J* = 8.8 Hz, 1H), 7.77 (s, 1H), 7.50-7.67 (m, 5H), 7.10 (s, 1H), 2.48 (s, 3H), 2.08 (m, 3H); **^{13}C NMR (100 MHz, $CDCl_3$)** δ 163.01, 139.75, 138.74, 136.64, 135.83, 134.13, 133.89, 129.77, 129.08, 129.01, 128.79, 128.55, 128.37, 128.23, 128.16, 128.03, 126.22, 122.65, 120.10, 118.09, 24.16, 21.82; **HRMS (ESI)** Calcd for $C_{25}H_{20}NO_3S^+$ $[M+H]^+$ 414.1158; Found: 414.1163.

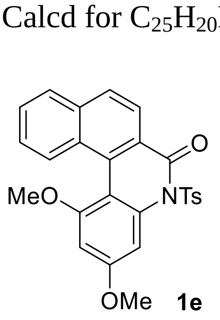

1e

1,3-Dimethoxy-5-tosylbenzo[*k*]phenanthridin-6(5*H*)-one (1e)

^{1}H NMR (400 MHz, $CDCl_3$) δ 8.12 (d, *J* = 8.4 Hz, 2H), 8.06 (d, *J* = 8.4 Hz, 1H), 7.94 (d, *J* = 8.4 Hz, 1H), 7.86 (d, *J* = 8.0 Hz, 1H), 7.81 (d, *J* = 8.4 Hz, 1H), 7.60 (t, *J* = 7.2 Hz, 1H), 7.48 (t, *J* = 8.0 Hz, 1H), 7.30-7.32 (m, 3H), 6.52 (s, 1H), 3.93 (s, 3H),

3.69 (s, 3H), 2.40 (s, 3H); **^{13}C NMR (100 MHz, $CDCl_3$)** δ 163.14, 160.73, 157.33, 145.24, 136.61, 136.06, 136.00, 133.49, 130.07, 129.70, 128.95, 128.51, 128.32, 127.85, 127.61, 126.57, 124.53, 122.50, 106.44, 97.56, 96.32, 55.77, 55.00, 21.71; **HRMS (ESI)** Calcd for $C_{26}H_{22}NO_5S^+$ $[M+H]^+$ 460.1213; Found: 460.1212.

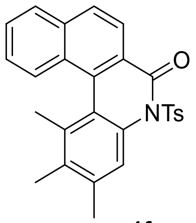

1f

1,2,3-Trimethyl-5-tosylbenzo[*k*]phenanthridin-6(5*H*)-one (1f)

^{1}H NMR (400 MHz, $CDCl_3$) δ 8.08 (t, *J* = 9.2 Hz, 3H), 7.87-7.94 (m, 3H), 7.81 (s, 1H), 7.64 (t, *J* = 8.4 Hz, 1H), 7.56 (t, *J* = 8.4 Hz, 1H), 7.29 (d, *J* = 8.4 Hz, 2H), 2.46 (s, 3H), 2.39 (s, 3H), 2.29 (s, 3H), 1.98 (s, 3H); **^{13}C NMR (100 MHz, $CDCl_3$)** δ 163.19, 144.90, 137.68, 136.74, 135.64, 135.16, 134.93, 133.93, 131.67, 129.59, 129.51, 128.69, 128.34, 128.16, 128.04, 126.26, 122.75, 120.52, 118.61, 77.25, 22.70, 21.67, 21.51, 16.03; **HRMS (ESI)** Calcd for $C_{27}H_{24}NO_3S^+$ $[M+H]^+$ 442.1471; Found: 442.1480.

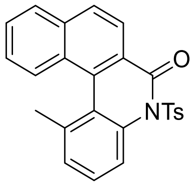

1g

1-Methyl-5-tosylbenzo[*k*]phenanthridin-6(5*H*)-one (1g)

^{1}H NMR (400 MHz, $CDCl_3$) δ8.13 (d, *J* = 8.4 Hz, 3H), 7.90-7.96 (m, 4H), 7.66 (t, *J* = 8.0 Hz, 1H), 7.59 (t, *J* = 8.0 Hz, 1H), 7.44 (t, *J* = 8.4 Hz, 1H), 7.32 (d, *J* = 8.4 Hz, 2H), 7.26 (d, *J* = 7.6 Hz, 1H), 2.41 (s, 3H), 2.11 (s, 3H); **^{13}C NMR (100 MHz, $CDCl_3$)** δ 162.94, 145.10, 136.84, 136.68, 135.76, 134.54, 134.22, 129.69, 129.08, 128.68, 128.63, 128.56, 128.46, 128.41, 128.21, 126.37, 122.67, 122.44, 117.58, 24.29, 21.70; **HRMS (ESI)** Calcd for $C_{25}H_{20}NO_3S^+$ $[M+H]^+$ 414.1158; Found: 414.1151.

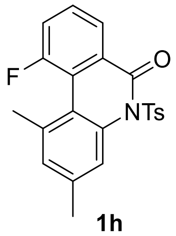

1h

10-Fluoro-1,3-dimethyl-5-tosylphenanthridin-6(5*H*)-one (1h)

^{1}H NMR (400 MHz, $CDCl_3$) δ 8.09 (d, *J* = 8.4 Hz, 2H), 7.92-7.94 (m, 1H), 7.60 (s, 1H), 7.42-7.46 (m, 2H), 7.34 (d, *J* = 8.4 Hz, 2H), 7.06 (s, 1H), 2.43 (s, 3H), 2.41 (s, 3H), 2.39 (d, *J* = 9.6 Hz, 3H); **^{13}C NMR (100 MHz, $CDCl_3$)** δ 162.2 (d_{C-F}, *J* = 14 Hz), 158.9, 156.4, 145.2, 139.0, 137.3, 136.6, 133.9, 131.7 (d_{C-F}, *J* = 16 Hz), 129.7, 129.6, 128.9 (d_{C-F}, *J* = 35 Hz), 128.5, 124.1 (d_{C-F}, *J* = 12 Hz), 122.8 (d_{C-F}, *J* = 60 Hz), 121.3, 121.1, 118.1, 116.5 (d_{C-F}, *J* = 8 Hz), 22.4, 22.2, 21.7; **HRMS (ESI)** Calcd for $C_{22}H_{19}FNO_3S^+$ $[M+H]^+$ 396.1064; Found: 396.1070.

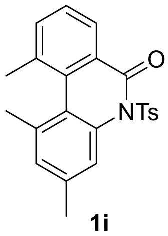

1i

1,3,10-Trimethyl-5-tosylphenanthridin-6(5*H*)-one (1i)
^{1}H NMR (400 MHz, $CDCl_3$) δ 8.10 (d, *J* = 8.4 Hz, 2H), 7.94 (d, *J* = 7.6 Hz, 1H), 7.54-7.56 (m, 2H), 7.32-7.39 (m, 3H), 7.02 (s, 1H), 2.39-2.43 (m, 9H), 2.27 (s, 3H); **^{13}C NMR (100 MHz, $CDCl_3$)** δ 163.6, 144.9, 138.0, 136.9, 136.2, 135.8, 135.7, 134.4, 133.7, 131.1, 129.6, 128.9, 128.3, 127.2, 125.6, 120.9, 118.0, 77.3, 21.7, 21.6, 21.5, 21.2; **HRMS (ESI)** Calcd for $C_{23}H_{22}NO_3S^+$ $[M+H]^+$ 392.1315; Found: 392.1323.

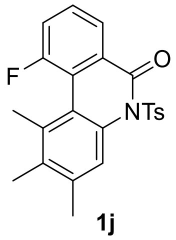

1j

10-Fluoro-1,2,3-trimethyl-5-tosylphenanthridin-6(5*H*)-one (1j)
^{1}H NMR (400 MHz, $CDCl_3$) δ 8.08 (d, *J* = 8.4 Hz, 2H), 7.92-7.94 (m, 1H), 7.63 (s, 1H), 7.41-7.49 (m, 2H), 7.34 (d, *J* = 8.4 Hz, 2H), 2.43 (s, 3H), 2.40 (s, 3H), 2.29 (d, *J* = 10.8 Hz, 2H), 2.26 (s, 3H); **^{13}C NMR (100 MHz, $CDCl_3$)** δ 162.4 (d_{C-F}, *J* = 14 Hz), 159.0, 156.5, 145.1, 138.0, 136.7, 135.7, 133.7, 132.0 (d_{C-F}, *J* = 15 Hz), 131.3, 129.7, 128.7 (d_{C-F}, *J* = 34 Hz), 128.4, 124.0 (d_{C-F}, *J* = 12 Hz), 123.4, 121.1, 120.8, 118.4, 116.8, 21.7, 21.5, 20.4 (d_{C-F}, *J* = 68 Hz), 16.0; **HRMS (ESI)** Calcd for $C_{23}H_{21}FNO_3S^+$ $[M+H]^+$ 410.1221; Found: 410.1227.

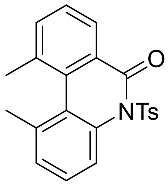

1k

1,10-Dimethyl-5-tosylphenanthridin-6(5*H*)-one (1k)

^{1}H NMR (400 MHz, CDCl$_3$) δ 8.14 (d, *J* = 8.4 Hz, 2H), 8.05 (d, *J* = 7.6 Hz, 1H), 7.72 (d, *J* = 8.4 Hz, 1H), 7.58 (d, *J* = 7.2 Hz, 1H), 7.42 (t, *J* = 7.6 Hz, 1H), 7.31-7.36 (m, 3H), 7.18 (d, *J* = 7.6 Hz, 1H), 2.44 (s, 3H), 2.41 (s, 3H), 2.32 (s, 3H); **^{13}C NMR (100 MHz, CDCl$_3$)** δ 163.5, 145.0, 136.9, 136.5, 136.1, 135.7, 134.1, 133.8, 131.4, 129.7, 128.3, 127.9, 127.9, 127.5, 125.7, 123.3, 117.3, 21.7, 21.6, 21.2; **HRMS (ESI)** Calcd for $C_{22}H_{20}NO_3S^+$ $[M+H]^+$ 378.1158; Found: 378.1152.

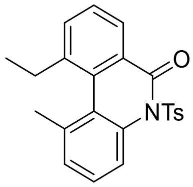

1l

10-Ethyl-1-methyl-5-tosylphenanthridin-6(5*H*)-one (1l)

^{1}H NMR (400 MHz, CDCl$_3$) δ 8.14 (d, *J* = 8.4 Hz, 2H), 8.00 (d, *J* = 7.2 Hz, 1H), 7.74 (d, *J* = 8.4 Hz, 1H), 7.66 (d, *J* = 7.6 Hz, 1H), 7.46 (t, *J* = 7.6 Hz, 1H),7.26-7.35 (m, 3H), 7.17 (d, *J* = 8.0 Hz, 1H), 2.90-2.99 (m, 1H), 2.63-2.72 (m,1H), 2.43 (s, 3H), 2.30 (s, 3H), 1.07 (t, *J* = 7.6 Hz, 3H); **^{13}C NMR (100 MHz, CDCl$_3$)** δ 163.4, 145.0, 142.8, 137.0, 136.5, 133.9, 133.8, 133.1, 131.1, 129.7, 128.3, 127.9, 127.9, 127.8, 125.7, 123.3, 117.4, 26.8, 21.7, 16.4; **HRMS (ESI)** Calcd for $C_{23}H_{22}NO_3S^+$ $[M+H]^+$ 392.1315; Found: 392.1321.

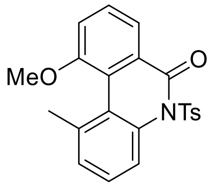

1m

10-Methoxy-1-methyl-5-tosylphenanthridin-6(5*H*)-one (1m)

^{1}H NMR (400 MHz, CDCl$_3$) δ 8.13 (d, *J* = 8.4 Hz, 2H), 7.72-7.77 (m, 2H), 7.46 (t, *J* = 8.0 Hz, 1H), 7.24-7.35 (m, 4H), 7.18 (d, *J* = 7.6 Hz, 1H), 3.92 (s, 3H), 2.42 (s,3H), 2.31 (s, 3H); **^{13}C NMR (100 MHz, CDCl$_3$)** δ 163.1, 155.7, 145.0, 137.8, 136.9, 133.5, 131.9, 129.7, 129.1, 128.4, 128.0, 127.7, 123.7, 121.1, 120.0, 117.0, 115.9, 55.4, 22.9, 21.7; **HRMS (ESI)** Calcd for $C_{22}H_{20}NO_4S^+$ $[M+H]^+$ 394.1108; Found: 394.1101.

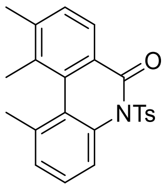

1n

1,9,10-Trimethyl-5-tosylphenanthridin-6(5*H*)-one (1n)

^{1}H NMR (400 MHz, CDCl$_3$) δ 8.14 (d, *J* = 8.4 Hz, 2H), 7.92 (d, *J* = 8.0 Hz, 1H),7.73 (d, *J* = 8.0 Hz, 1H), 7.26-7.35 (m, 4H), 7.18 (d, *J* = 8.0 Hz, 1H), 2.43 (s, 6H), 2.28 (s, 6H); **^{13}C NMR (100 MHz, CDCl$_3$)** δ 163.5, 144.9, 144.0, 137.0, 136.6, 134.7, 134.3,

133.9, 129.7, 129.4, 129.3, 128.3, 127.8, 127.7, 125.6, 123.8, 117.4, 21.7, 21.0, 19.3; **HRMS (ESI)** Calcd for $C_{23}H_{22}NO_3S^+$ $[M+H]^+$ 392.1315; Found: 392.1323.

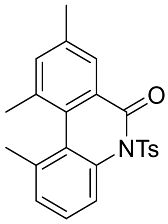

1o

1,8,10-Trimethyl-5-tosylphenanthridin-6(5*H*)-one (1o)

^{1}H NMR (400 MHz, $CDCl_3$) δ 8.15 (d, *J* = 8.4 Hz, 2H), 7.81 (s, 1H), 7.71 (d, *J* = 8.4 Hz, 1H), 7.28-7.39 (m, 4H), 7.17 (d, *J* = 8.0 Hz, 1H), 2.44 (s, 3H), 2.42 (s, 3H), 2.37 (s, 3H), 2.31 (s, 3H); **^{13}C NMR (100 MHz, $CDCl_3$)** δ 163.6, 144.9, 137.7, 137.0, 136.8, 136.3, 136.0, 133.7, 131.7, 131.3, 129.7, 128.3, 127.8, 126.0, 123.4, 117.2, 21.7, 21.6, 21.1, 21.0; **HRMS (ESI)** Calcd for $C_{23}H_{22}NO_3S^+$ $[M+H]^+$ 392.1315; Found: 392.1311.

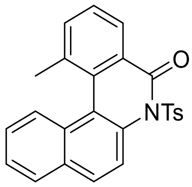

1p

1-Methyl-6-tosylbenzo[a]phenanthridin-5(6*H*)-one (1p)

^{1}H NMR (400 MHz, $CDCl_3$) δ 8.19 (d, *J* = 8.4 Hz, 2H), 8.14 (d, *J* = 9.2 Hz, 1H), 8.10 (d, *J* = 6.4 Hz, 1H), 7.92 (d, *J* = 9.2 Hz, 1H), 7.87-7.89 (m,1H),7.67-7.70 (m, 2H), 7.48-7.53 (m, 3H), 7.37 (d, *J* = 9.2 Hz, 2H), 2.45 (s, 3H), 2.30 (s, 3H); **^{13}C NMR (100 MHz, $CDCl_3$)** δ 163.6, 145.2, 136.9, 136.4, 133.6, 132.1, 131.7, 130.9, 129.8, 129.7, 129.3, 128.3, 128.2, 127.8, 127.2, 126.5, 126.0, 125.6, 118.9, 118.4, 23.0, 21.7; **HRMS (ESI)** Calcd for $C_{25}H_{20}NO_3S^+$ $[M+H]^+$ 414.1158; Found: 414.1153.

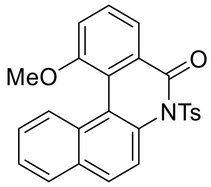

1q

1-Methoxy-6-tosylbenzo[a]phenanthridin-5(6*H*)-one (1q)

^{1}H NMR (400 MHz, $CDCl_3$) δ 8.18 (d, *J* = 8.4 Hz, 2H), 8.12 (d, *J* = 9.2 Hz, 1H), 7.82-7.91 (m, 3H), 7.73 (d, *J* = 8.4 Hz, 1H), 7.55 (t, *J* = 8.0 Hz, 1H), 7.41-7.49 (m,2H), 7.36 (d, *J* = 8.4 Hz, 3H), 3.85 (s, 3H), 2.44 (s, 3H); **^{13}C NMR (100 MHz, $CDCl_3$)** δ 163.3, 155.8, 145.2, 136.9, 132.2, 132.1, 130.9, 129.9, 129.8, 129.2, 129.1, 128.4, 128.3, 127.8, 125.5, 125.2, 123.3, 120.5, 118.5, 116.5, 116.4, 55.3, 21.7; **HRMS (ESI)** Calcd for $C_{25}H_{20}NO_4S^+$ $[M+H]^+$ 430.1108; Found: 430.1113.

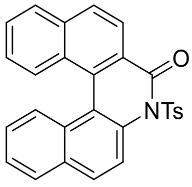

1r

3-Tosyldibenzo[*a,k*]phenanthridin-4(3*H*)-one (1r)

^{1}H NMR (400 MHz, CDCl$_3$) δ 8.30 (d, *J* = 9.2 Hz, 1H), 8.17-8.21 (m, 3H),7.94-8.00 (m, 4H), 7.89 (d, *J* = 7.6 Hz, 1H), 7.63-7.67 (m, 2H), 7.38-7.48 (m, 2H), 7.33 (d, *J* = 8.0 Hz, 2H), 7.23-7.27 (m, 1H), 2.42 (s, 3H); **^{13}C NMR (100 MHz, CDCl$_3$)** δ 163.2, 145.3, 136.6, 136.1, 133.9, 132.7, 131.2, 130.2, 129.9, 129.8, 129.4, 129.2, 129.1, 129.0, 128.8, 128.5, 128.3, 128.2, 127.7, 125.9, 125.8, 122.9, 118.9, 117.6, 21.7; **HRMS (ESI)** Calcd for $C_{28}H_{20}NO_3S^+$ $[M+H]^+$ 450.1158; Found: 450.1164.

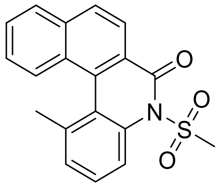

1s

1-Methyl-5-(methylsulfonyl)benzo[*k*]phenanthridin-6(5*H*)-one (1s)

^{1}H NMR (400 MHz, CDCl$_3$) δ 8.22 (d, *J* = 8.4Hz, 1H), 7.95-7.98 (m, 2H), 7.86-7.91 (m, 2H), 7.56-7.68 (m, 2H), 7.46 (t, *J* = 8.0 Hz, 1H),7.27 (d, *J* = 8.0Hz, 1H), 3.71 (s, 3H), 2.12 (s, 3H); **^{13}C NMR (100 MHz, CDCl$_3$)** δ 164.2, 137.2, 136.0, 134.8, 133.9, 129.2, 128.9, 128.8, 128.7, 128.7, 128.6, 128.4, 128.3, 126.6, 122.6, 122.5, 116.9, 44.4, 24.4; **HRMS (ESI)** Calcd for $C_{19}H_{16}NO_3S^+$ $[M+H]^+$ 338.0845; Found: 338.0848.

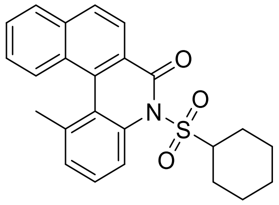

1t

5-(Cyclohexylsulfonyl)-1-methylbenzo[*k*]phenanthridin-6(5*H*)-one (1t)

^{1}H NMR (400 MHz, CDCl$_3$) δ 8.23 (d, *J* = 8.8Hz, 1H), 7.96 (d, *J* = 8.8 Hz, 2H), 7.92 (t, *J* = 8.0 Hz, 1H), 7.56-7.67 (m, 2H), 7.44 (t, *J* = 8.4 Hz, 1H), 7.25 (s, 1H), 4.29-4.37 (m, 1H), 2.31-2.37 (m, 1H), 2.12 (s, 3H), 1.73-2.00 (m, 5H), 1.23-1.46 (m, 3H); **^{13}C NMR (100 MHz, CDCl$_3$)** δ 164.5, 136.8, 135.9, 135.1, 134.8, 129.3, 128.8, 128.8, 128.5, 128.4, 128.3, 126.5, 122.7, 117.8, 66.2, 26.8, 25.7, 25.3, 25.2, 25.2, 24.5; **HRMS (ESI)** Calcd for $C_{24}H_{24}NO_3S^+$ $[M+H]^+$ 406.1471; Found: 406.1473.

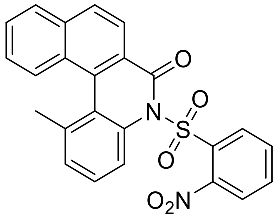

1u

1-Methyl-5-((2-nitrophenyl)sulfonyl)benzo[*k*]phenanthridin-6(5*H*)-one (1u)
^{1}H NMR (400 MHz, CDCl$_3$) δ 8.53 (d, *J* = 8.0Hz, 1H), 8.03 (d, *J* = 8.4 Hz, 1H), 7.98 (d, *J* = 8.4 Hz, 2H), 7.91 (d, *J* = 7.6 Hz, 1H), 7.84 (d, *J* = 8.4 Hz, 1H), 7.78 (t, *J* = 7.6 Hz, 1H), 7.58-7.71 (m, 4H), 7.49 (t, *J* = 8.0 Hz, 1H), 7.34 (d, *J* = 7.6 Hz, 1H), 2.15 (s, 3H); **^{13}C NMR (100 MHz, CDCl$_3$)** δ 161.9, 147.9, 137.0, 135.9, 135.8, 135.1, 134.9, 133.5, 132.4, 131.9, 129.5, 129.3, 129.2, 128.9, 128.6, 128.5, 128.1, 127.2, 126.5, 125.0, 122.6, 122.5, 118.9, 24.6; **HRMS (ESI)** Calcd for $C_{24}H_{17}N_2O_5S^+$ [M+H]$^+$ 445.0853; Found: 445.0861.

Supplementary Note 3. Atroposelective Synthesis of Axially Chiral Biaryl Amino Acids **3**

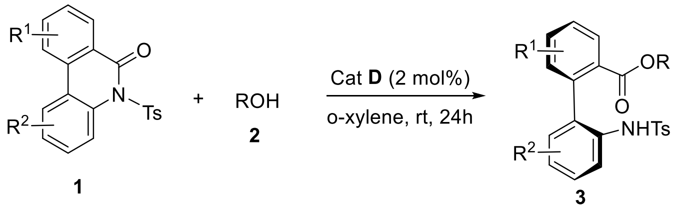

To a suspension of starting materials **1** (0.10 mmol) and catalyst **D** (1.2 mg, 2 mol%) in o-xylene (0.5 mL, 0.20 M) was added the appropriate alcohols (0.12 mmol, 1.2 equiv). The mixture was stirred for 24-48 h at 30 ℃. Upon completion of the reaction (monitored by TLC), the reaction mixture was directly purified by column chromatography on silica gel to afford the desired product **3**.

Supplementary Note 4. Preparation of Catalyst **E**

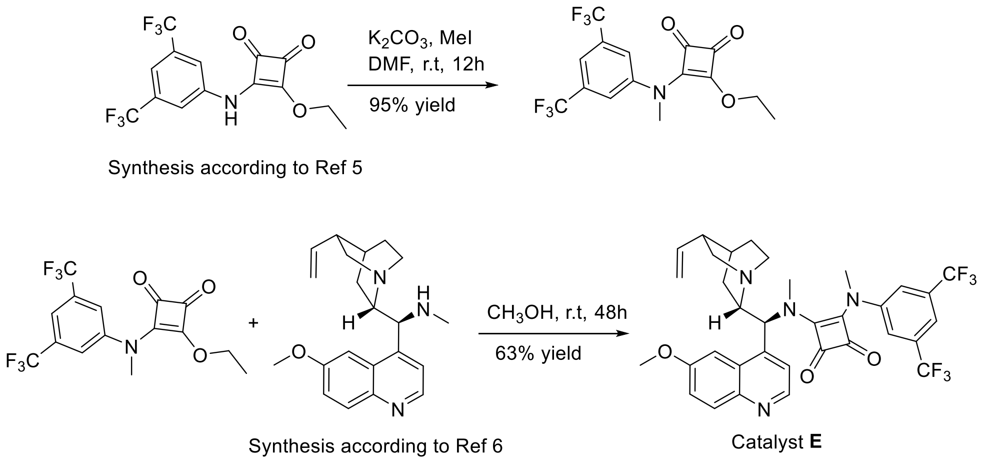

To a suspension of starting materials (3.0 mmol, 1.0 equiv) and K_2CO_3 (3.0 equiv, 9.0 mmol) in DMF (15 mL) was added MeI (2.0 equiv, 6.0 mmol). The mixture was stirred for 12 hours at r.t. Upon completion, the obtained mixture was diluted with 30 mL EtOAc and 50 mL H_2O. The organic layer was separated and aqueous phase was extracted twice with EtOAc. The combined organic layer was washed with brine and then dried over Na_2SO_4. The resulting mixture was filtered, concentrated *in vacuo* and purified by column chromatography on silica gel using 10% EtOAc in hexanes as eluent to afford the desired product as a white solid (1.04 g, 2.85 mmol, 95% yield).

To a solution of product obtained above (0.30 mmol, 1.0 equiv) in CH_3OH (1.5 mL) was added methyl protected amine (0.30 mmol, 1.0 equiv) slowly. The reaction mixture was stirred for 48 hours at r.t. The resulting solution was concentrated *in vacuo* and purified by column chromatography on silica gel to afford the catalyst **E** as a white solid (124 mg, 0.19 mmol, 63% yield). $[\alpha]_D^{23}$ (c 0.32, $CHCl_3$) = + 94.9; **^{1}H NMR** (400 MHz, $CDCl_3$) δ 8.74 (d, *J* = 4.4 Hz, 1H), 8.07 (d, *J* = 9.2 Hz, 1H), 7.75 (d, *J* = 2.4 Hz, 1H), 7.40-7.43 (m, 2H), 7.12 (d, *J* = 4.8 Hz, 1H), 7.09 (s, 2H), 6.63 (d, *J* = 11.2 Hz, 1H), 5.88-5.97 (m, 1H), 5.07-5.12 (m, 2H), 3.94 (s, 3H), 3.69 (s, 3H), 3.43-3.57 (m, 2H), 3.26 (q, *J* = 14.0, 10.0 Hz, 1H), 2.82-2.86 (m, 1H), 2.67-2.74 (m, 1H), 2.38 (s, 3H), 2.32-2.36 (m, 1H), 1.63-1.74 (m, 3H), 1.49-1.58 (m, 1H), 0.73-0.78 (m, 1H); **^{13}C NMR** (100 MHz, $CDCl_3$) δ 188.5, 184.5, 173.5, 164.2, 159.37, 146.6, 145.8, 145.6, 141.9, 137.8, 133.3, 132.9, 132.3, 128.9, 123.9, 123.2, 121.2, 119.8, 118.2, 116.8, 114.7, 100.6, 77.3, 58.3, 56.6, 56.4, 53.9, 41.1, 39.6, 38.8, 33.3, 27.6, 27.4, 27.1. ^{19}F NMR (376 MHz, $CDCl_3$): δ = - 63.4; **HRMS** (ESI) Calcd for $C_{34}H_{33}F_6N_4O_3^+$ $[M+H]^+$ 659.2451; Found: 659.2463.

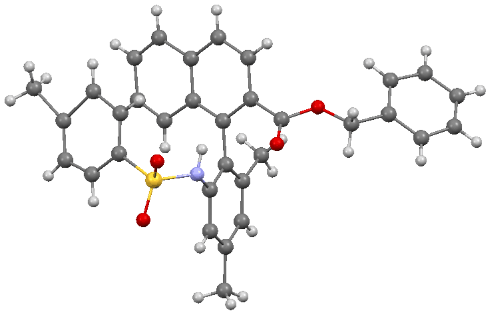

Supplementary Figure 1. X-ray crystal structure of **3a.** Product **3a** was crystallized as a colorless crystal *via* vaporization of a hexane/ethyl acetate solution, and its absolute configuration was determined by X-ray structure analaysis. CCDC1894533 contains the supplementary crystallographic data that can be obtained free of charge from The Cambridge Crystallographic Data Centrevia www.ccdc.cam.ac.uk/data request/cif.

Supplementary Table 1. Crystal data and structure refinement for **3a**

Identification code	111	
Empirical formula	$C_{132}H_{116}N_4O_{16}S_4$	
Formula weight	2142.52	
Temperature	301.76 K	
Crystal system	triclinic	
Space group	P1	
Unit cell dimensions	a = 9.877(3) Å	α= 89.993(7) °
	b = 13.046(4) Å	β= 88.704(7) °
	c = 24.858(7) Å	γ= 89.723(7) °
Volume	3202.3(15) Å	
Z	1	
Density(calculated)	1.111 g/cm^3	
μ	0.135 mm^{-1}	
F(000)	1128.0	
Crystal size	0.2 × 0.2 × 0.1 mm^3	
Radiation	MoKα (λ = 0.71073)	
2Θ range for data collection	1.638 to 57.55 °	
Index ranges	-13 ≤ h ≤ 10, -17 ≤ k ≤ 17, -33 ≤ l ≤ 33	
Reflections collected	29118	
Independent reflections	20966 [Rint = 0.0415, Rsigma = 0.0868]	
Data/restraints/parameters	20966/423/1417	
Goodness-of-fit on F^2	1.014	
Final R indexes [I>=2σ (I)]	R_1 = 0.0716, wR_2 = 0.1869	
Final R indexes [all data]	R_1 = 0.1312, wR_2 = 0.2195	
Largest diff. peak/hole	0.39/-0.29 Å^{-3}	
Flack parameter	0.15(6)	

Supplementary Note 5. Reaction in Gram-scale.

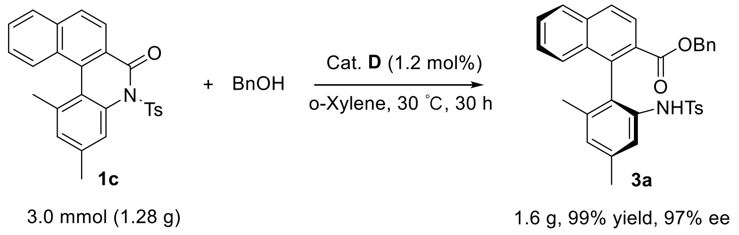

To a suspension of **1c** (1.28 g, 3.0 mmol) and catalyst **D** (22.7 mg, 1.2 mol%) in o-xylene (7.5 mL, 0.40 M) was added benzyl alcohol (0.37 mL, 3.6 mmol, 1.2 equiv).

After being stirred for 30 h at 30 ℃ (completion monitored by TLC), the reaction mixture was directly purified by column chromatography on silica gel to afford the desired product **3a** as a white solid (1.6 g, 2.99 mmol, 99% yield).

Supplementary Table 2. Attempts for non-alcohol nucleophiles

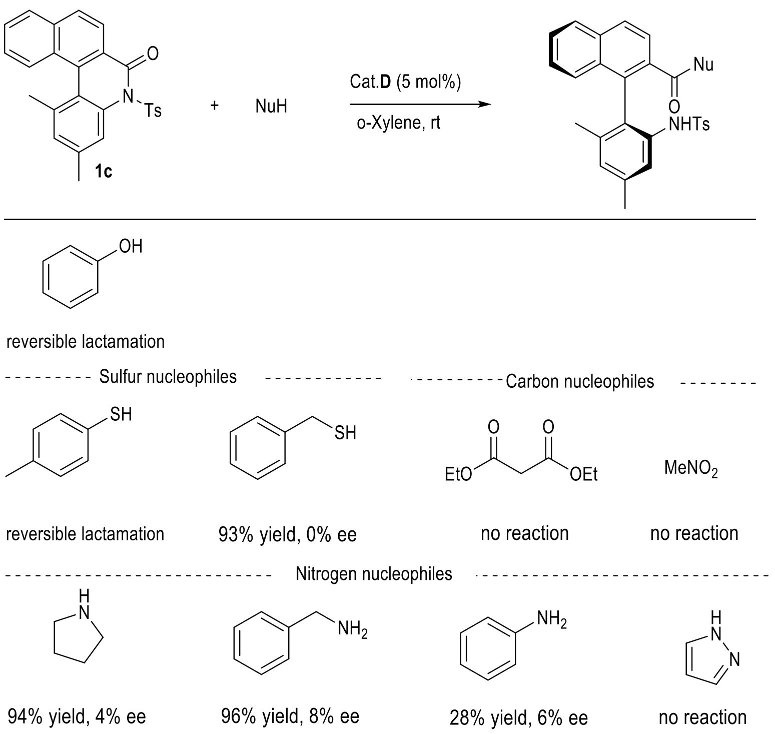

Standard reaction condition: **1c** (0.10 mmol), nucleophiles (1.5 equiv), Catalyst **D** (5 mol%), o-Xylene (0.20 M), r.t, 24 h.

We also examined the effectiveness of non-alcohol nucleophiles on this organocatalytic ring opening reaction. When phenol and p-toluenethiol were used as nucleophiles, we detected the formation of the ring-opening products, but failed to isolate the pure product due to the highly ease of reversible lactonization. Benzyl thioalcohol can promote the ring-opening reaction smoothly under standard reaction conditions with high yield but 0% ee. We have tried CH-acidic compound such as diethyl malonate and $MeNO_2$, but they did not deliver any product. For nitrogen nucleophiles, pyrrolidine and benzylamine promoted the formation of product in high yield but negligible stereoselectivity. The weaker nucleophilic aniline has a lower conversion and no improvement in stereoselectivity. Pyrazole cannot perform this ring opening reaction.

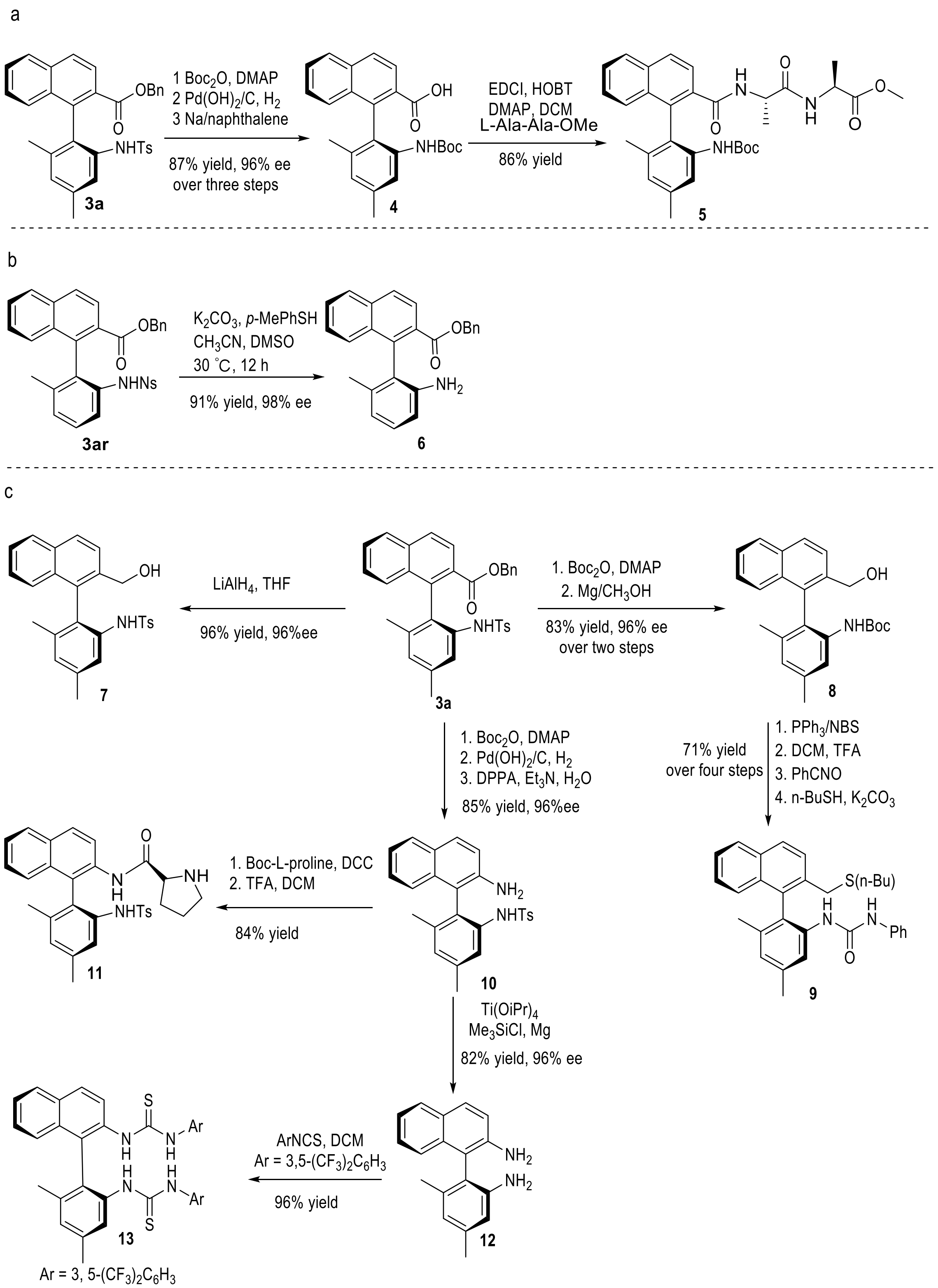

Supplementary Figure 2. Diverse Transformations of Products **3**. a Preparation of N-Boc axially chiral biaryl amino acid **4** & tripeptide **5**. b Synthesis of unprotected axially chiral biaryl amino esters. c Synthesis of axially chiral organocatalysts.

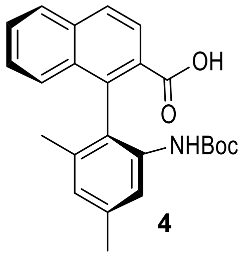

4

(*S*)-1-(2-((Tert-butoxycarbonyl)amino)-4,6-dimethylphenyl)-2-naphthoic acid (4)

To a suspension of **3a** (80 mg, 0.15 mmol) and DMAP (1.8 mg, 0.015 mmol) in THF (2 mL) was added di-tert-butyldicarbonate (23 μl, 0.18 mmol) at r.t. After stirring for 2 hours, the solution was carefully concentrated and purified by column chromatography on silica-gel to give the resulting products (93 mg, 98% yield). The obtained Boc-protected **3a** was dissolved in MeOH (2 mL), and then $Pd(OH)_2/C$ (10 wt% of the starting material) was added in one portion at r.t. After being stirred for 2 hours at 35 ℃ under H_2 atmosphere, the mixture was filtered through a pad of Celite. The solution was concentrated *in vacuo* to give the resulting acid as a white solid (77 mg, 96% yield), which was directly used in the next step without further purification.

Under nitrogen atmosphere, the acid was dissolved in ethylene glycol diethyl ether (0.5 mL) and then Na-naphthalene solution (stirring a mixture of 30 equiv naphthalene and 60 equiv Na in 5 mL ethylene glycol diethyl ether for 1h at r.t) was added dropwise at -78 ℃ until a deep blue color persisted. The reaction mixture was stirred for 16 hours at -78 ℃, quenched with aqueous NH_4Cl solution, and diluted with EtOAc. The organic layer was washed with brine, dried over Na_2SO_4, filtered and concentrated. The residue was purified by column chromatography on silica gel using CH_3OH/DCM = 1:10 as eluent to afford the desired product as a colorless oil. 51.3 mg, 93% yield, 96% ee. $[\alpha]_D^{23}$ (c 0.74, $CHCl_3$) = + 34.0.

HPLC condition: Chiralpak AD-H (Hex/*i*PrOH = 95/5, 1.0 mL/min, t_R (major) = 19.6 min, t_R (minor) = 23.8 min);

^{1}H NMR (400 MHz, $CDCl_3$) δ 7.67-7.80 (m, 3H), 7.47-7.49 (m, 1H), 7.29-7.35 (m, 3H), 6.77 (s, 1H), 6.06 (br s, 1H), 2.26 (s, 3H), 1.62 (s, 3H), 1.16 (s, 9H); **^{13}C NMR** (100 MHz, $CDCl_3$) δ 171.1, 138.3, 137.1, 135.6, 135.2, 131.9, 128.5, 128.2, 128.0, 127.5, 127.3, 126.6, 126.1, 80.6, 28.0, 21.4, 20.0; **HRMS** (ESI) Calcd for $C_{24}H_{26}NO_4^+$ $[M+H]^+$ 392.1856; Found: 392.1851.

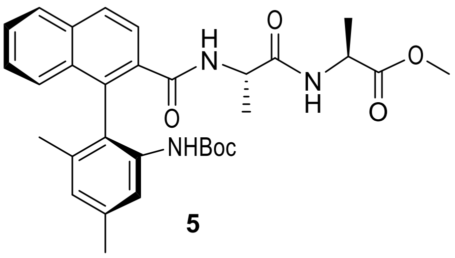

5

Methyl ((*S*)-1-(2-((tert-butoxycarbonyl)amino)-4,6-dimethylphenyl)-2-naphthoyl)-L-alanyl-L-alaninate (5)

To a solution of Z-Ala-Ala-OMe (30.8 mg, 0.10 mmol) in MeOH (1.2 mL) was added Pd/C (3.0 mg, 10 wt% of the starting material) in one portion. After being stirred for 2 hours at r.t under H_2 atmosphere, the mixture was filtered through a pad of Celite. The filter cake was washed with small portions of MeOH, concentrated *in vacuo* to give the resulting product, which was directly used in the next step without further purification. The obtained L-Ala-Ala-OMe was dissolved in DCM (2.0 mL) immediately and added to the oven-dried Schlenk tube containing **4** (20.0 mg, 0.050 mmol), EDCI (11.5 mg, 0.060 mmol), HOBt (6.8 mg, 0.050 mmol) and DMAP (9.0 mg, 0.075 mmol), the mixture was stirred for 8 hours at r.t under N_2 atmosphere. The mixture was quenched with aqueous NH_4Cl solution, and diluted with EtOAc. The organic layer was washed with $NaHCO_3$ (aq) and brine, dried over Na_2SO_4, filtered and concentrated. The residue was purified by column chromatography on silica gel using EtOAc/hexanes = 1:1 as eluent to afford the desired peptide **5**. 23.5 mg, 86% yield. $[\alpha]_D^{23}$ (c 0.18, $CHCl_3$) = + 81.5; **^{1}H NMR** (400 MHz, $CDCl_3$) δ 7.96 (s, 2H), 7.92 (d, *J* = 6.8 Hz, 1H), 7.76 (s, 1H), 7.58 (t, *J* = 6.4 Hz, 1H), 7.43 (t, *J* = 6.8 Hz, 1H), 7.34 (d, *J* = 6.8 Hz, 1H), 6.96 (s, 1H), 6.61 (d, *J* = 6.0 Hz, 1H), 6.41 (d, *J* = 5.6 Hz, 1H), 5.85 (s, 1H), 4.40-4.51 (m, 2H), 3.72 (s, 3H), 2.41 (s, 3H), 1.83 (s, 3H), 1.36 (d, *J* = 5.6 Hz, 3H), 1.26 (s, 9H), 1.05 (d, *J* = 5.6 Hz, 3H); **^{13}C NMR** (100 MHz, $CDCl_3$) δ 173.0, 171.4, 168.2, 153.2, 139.3, 137.1, 136.5, 134.7, 132.7, 132.5, 131.7, 128.9, 128.3, 127.6, 127.4, 127.0, 126.2, 125.8, 125.4, 120.5, 80.5, 52.4, 49.2, 48.1, 28.1, 21.5, 20.0, 18.1, 17.1, 21.6, 19.9; **HRMS** (ESI) Calcd for $C_{31}H_{38}N_3O_6^+$ $[M+H]^+$ 548.2755; Found: 548.2743.

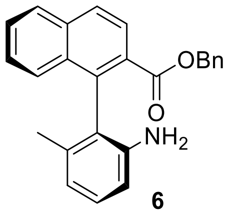

6

Benzyl (*S*)-1-(2-amino-6-methylphenyl)-2-naphthoate (6)

To a mixture of **3ar** (55.2 mg, 0.10 mmol), K_2CO_3 (69.1 mg, 5.0 equiv) and p-toluenethiol (49.7 mg, 4.0 equiv) in acetonitrile (1 mL, 0.10 M) was added one drop DMSO at r.t under N_2 atmosphere. After being stirred for 12 hours at 30 ℃, the resulting solution was concentrated *in vacuo* and purified by column chromatography on silica gel using EtOAc/hexanes = 1:10 as eluents to afford the product as a yellow oil. (36.7 mg, 96% yield, 98% ee), $[\alpha]_D^{23}$ (c 0.28, $CHCl_3$) = -382.4.

HPLC condition: Chiralpak IA (Hex/*i*PrOH = 95/5, 1.0 mL/min, t_R (major) = 18.4 min, t_R (minor) = 13.5 min);

^{1}H NMR (400 MHz, $CDCl_3$) δ 8.04 (d, *J* = 8.8Hz, 1H), 7.93 (t, *J* = 8.0 Hz, 1H), 7.32-7.60 (m, 6H), 7.14-7.22 (m, 3H), 6.74 (d, *J* = 7.2 Hz, 1H), 6.64 (d, *J* = 7.2Hz, 1H), 5.16 (s, 2H), 3.21 (bs, 2H), 1.76 (s, 3H); **^{13}C NMR (100 MHz, $CDCl_3$)** δ 167.9, 144.3, 137.6, 137.4, 135.8, 135.4, 132.0, 129.1, 128.5, 128.5, 128.3, 128.2, 128.1, 127.4, 126.7, 126.2, 124.2, 120.2, 113.1, 67.2, 20.1; **HRMS (ESI)** Calcd for $C_{25}H_{21}NNaO_2^+$ $[M+Na]^+$ 390.1465; Found: 390.1467

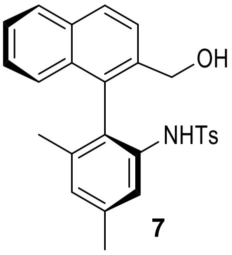

7

(*S*)-N-(2-(2-(hydroxymethyl)naphthalen-1-yl)-3,5-dimethylphenyl)-4-methylbenzenesulfonamide (7)

To a solution of **3a** (0.10 mmol, 1.0 equiv) in THF (1.0 mL) was slowly added $LiAlH_4$ (0.20 mmol, 2.0 equiv) at 0 ℃. After stirring for 2 hours at r.t, the mixture was quenched with aqueous NH_4Cl solution, and diluted with EtOAc. The organic layer was washed with brine, dried over Na_2SO_4, filtered and concentrated. The residue was purified by column chromatography on silica gel using EtOAc/hexanes = 1:3 as eluent to afford the resulting product **6** as a white solid. 96% yield, 96% ee. $[\alpha]_D^{23}$ (c 1.6, $CHCl_3$) = + 26.0.

HPLC condition: Chiralpak AD (Hex/*i*PrOH = 90/10, 1.0 mL/min, t_R (major) = 17.6 min, t_R (minor) = 25.6 min);

^{1}H NMR (400 MHz, $CDCl_3$) δ 7.93 (d, *J* = 8.4 Hz, 1H), 7.86 (d, *J* = 8.0 Hz, 1H), 7.71 (d, *J* = 8.4 Hz, 1H), 7.43-7.46 (m, 2H), 7.35 (d, *J* = 7.2 Hz, 2H), 7.10 (t, *J* = 8.0 Hz, 1H), 7.03 (d, *J* = 6.4 Hz, 2H), 6.91 (s, 1H), 6.79 (d, *J* = 8.4 Hz, 1H), 6.32 (s, 1H), 4.32-4.37 (m, 1H), 4.23-4.27 (m, 1H), 2.39 (s, 3H), 2.36 (s, 3H), 1.89-1.95 (m, 1H), 1.67 (s, 3H); **^{13}C NMR** (100 MHz, $CDCl_3$) δ 143.6, 138.7, 138.0, 137.1, 136.4, 134.9, 133.3, 131.8, 130.9, 129.4, 129.3, 128.3, 127.3, 127.1, 126.8, 126.2, 126.0, 125.2, 124.8, 118.2, 63.1, 21.6, 21.6, 20.0; **HRMS** (ESI) Calcd for $C_{26}H_{25}NNaO_3S^+$ $[M+Na]^+$ 454.1447; Found: 454.1443.

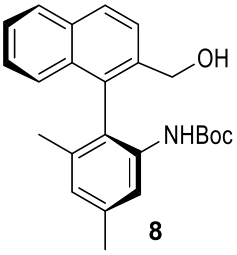

8

Tert-butyl (*S*)-(2-(2-(hydroxymethyl)naphthalen-1-yl)-3,5-dimethylphenyl)carbamate (8)

To a solution of Boc-protected **3a** (95 mg, 0.15 mmol) in CH_3OH was added Mg powder (55 mg, 2.25 mmol, 15.0 equiv) in one portion at r.t. After being sonicated for 1 hour, the mixture was poured into aqueous NH_4Cl solution, extracted with EtOAc. The organic layer was dried over Na_2SO_4, filtered and concentrated. The residue was purified by column chromatography on silica gel using EtOAc/hexanes = 1:3 as eluent to afford the resulting product **8** as a white solid. 48 mg, 85% yield, 95% ee. $[\alpha]_D^{23}$ (c 0.41, $CHCl_3$) = -195.3.

HPLC condition: Chiralpak AD (Hex/*i*PrOH = 95/5, 1.0 mL/min, t_R (major) = 7.9 min, t_R (minor) = 9.9 min);
^{1}H NMR (400 MHz, $CDCl_3$) δ 7.95 (d, *J* = 6.8 Hz, 1H), 7.91 (d, *J* = 6.8 Hz, 1H), 7.81 (s, 1H),7.76 (d, *J* = 6.8 Hz, 1H), 7.49 (t, *J* = 5.2 Hz, 1H),7.37 (t, *J* = 6.4 Hz, 1H),7.29 (d, *J* = 6.8 Hz, 1H),6.93 (s, 1H),5.85 (s, 1H),4.47 (s, 2H), 2.43 (s, 3H), 1.76 (s, 3H), 1.30 (s, 9H); **^{13}C NMR** (100 MHz, $CDCl_3$) δ 153.3, 138.4, 137.2, 137.2, 136.3, 133.3, 132.1, 131.8, 128.7, 128.2, 126.7, 126.2, 126.1, 126.0, 125.2, 124.7, 119.0, 80.3, 63.0, 28.1, 21.5, 19.9, 126.0, 117.8, 113.7, 77.3, 67.0, 55.3, 21.6, 21.6, 19.9; **HRMS** (ESI) Calcd for $C_{24}H_{28}NO_3^+$ $[M+H]^+$ 378.2064; Found: 378.2061.

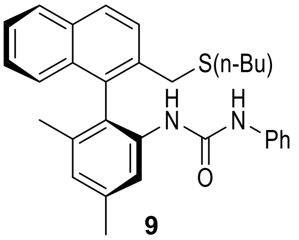

9

(*S*)-1-(2-(2-((Butylthio)methyl)naphthalen-1-yl)-3,5-dimethylphenyl)-3-phenylurea (9)

Preparation of **9** from **8** was operated according to the reported procedures [7], [8]. 71% yield over four steps $[\alpha]_D^{23}$ (c 0.05, $CHCl_3$) = + 535.7; **^{1}H NMR** (400 MHz, $CDCl_3$) δ 8.06 (s, 1H), 7.84 (t, *J* = 6.0 Hz, 2H), 7.52 (d, *J* = 6.8 Hz, 1H), 7.47 (t, *J* = 6.4 Hz, 1H), 7.37 (t, *J* = 6.8 Hz, 1H), 7.24 (d, *J* = 6.4 Hz, 1H), 6.94-6.99 (m, 3H), 6.65-6.71 (m, 2H), 6.20 (d, *J* = 10.8 Hz, 2H), 3.56 (d, *J* = 10.0 Hz, 1H), 3.40 (d, *J* = 10.0 Hz, 1H), 2.53 (s, 3H), 2.26-2.35 (m, 2H), 1.91 (s, 3H), 1.35-1.42 (m, 2H), 1.24-1.31 (m, 2H), 0.84 (t, *J* = 5.6 Hz, 3H); **^{13}C NMR** (100 MHz, $CDCl_3$) δ 152.8, 138.8, 137.1, 137.0, 135.8, 135.6, 133.0, 132.4, 132.1, 129.2, 128.9, 128.2, 127.5, 127.3, 126.3, 125.3, 124.7, 122.5, 122.1, 120.6, 34.4, 32.5, 31.3, 24.5, 21.9, 21.4, 13.6; **HRMS** (ESI) Calcd for $C_{30}H_{33}N_2OS^+$ $[M+H]^+$ 469.2308; Found: 469.2301.

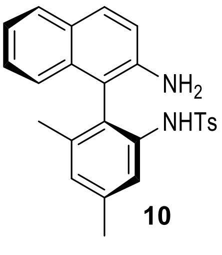

10

(*S*)-N-(2-(2-aminonaphthalen-1-yl)-3, 5-dimethylphenyl)-4-methylbenzenesulfonamide (10)

The acid was prepared following the procedure of **4.** The obtained acid (81.8 mg, 0.15 mmol) was dissolved in toluene, Et_3N (0.75 mmol, 5.0 equiv) and diphenylphosphoryl azide (0.30 mmol, 2.0 equiv) was sequentially added via syringe under N_2 atmosphere. After stirring for 30 minutes at r.t, the reaction was heated to 100 ℃ for another 12 hours. The toluene was removed under reduced pressure and replaced with THF and H_2O (v/v = 1:1, 1.5 mL). The solution was stirred for 12 hours at 100 ℃ and then THF was removed *in vacuo*. To the residue was added K_2CO_3 (aq)

and EtOAc. The two phases were separated and the aqueous layer was extracted with EtOAc twice. The combined organic layer was washed with brine, dried over Na_2SO_4, filtered and concentrated. The residue was purified by column chromatography on silica gel using EtOAc/hexanes = 1:5 as eluent to afford the resulting product **10**. 53.0 mg, 85% yield, 96% ee. $[\alpha]_D^{23}$ (c 0.48, $CHCl_3$) = - 187.5.

HPLC condition: Chiralpak ID (Hex/*i*PrOH = 80/20, 1.0 mL/min, t_R (major) = 14.3 min, t_R (minor) = 10.7 min);

^{1}H NMR (400 MHz, $CDCl_3$) δ 7.72-7.74 (m, 2H), 7.51 (s, 1H), 7.41 (d, *J* = 6.8 Hz, 2H), 7.21 (t, *J* = 6.4 Hz, 1H), 7.04-7.08 (m, 3H), 7.00 (d, *J* = 6.8 Hz, 1H), 6.92 (s, 1H), 6.61 (d, *J* = 6.8 Hz, 1H), 6.33 (s, 1H), 2.39 (s, 3H), 2.36 (s, 3H), 1.79(s, 3H); **^{13}C NMR** (100 MHz, $CDCl_3$) δ 143.5, 142.0, 139.3, 138.9, 136.4, 135.6, 133.0, 130.2, 129.4, 128.3, 128.2, 127.4, 127.3, 127.1, 122.8, 122.7, 122.4, 117.9, 117.8, 111.2, 21.5, 21.5, 19.7; **HRMS** (ESI) Calcd for $C_{25}H_{25}N_2O_2S^+$ $[M+H]^+$ 417.1631; Found: 417.1624.

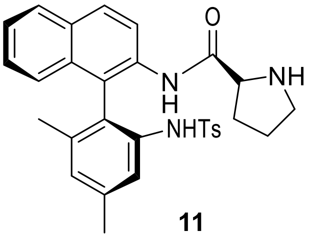

11

(*R*)-N-((*S*)-1-(2, 4-dimethyl-6-((4-methylphenyl) sulfonamido) phenyl) naphthalen-2-yl) pyrrolidine-2-carboxamide (11)

Preparation of **11** from **10** was operated according to the reported procedures [9].84% yield over two steps. $[\alpha]_D^{23}$ (c 0.7, $CHCl_3$) = + 87.5; **^{1}H NMR** (400 MHz, $CDCl_3$) δ 9.54 (s, 1H), 8.73 (d, *J* = 9.2 Hz, 1H), 7.95 (d, *J* = 9.2 Hz, 1H),7.87 (d, *J* = 8.0 Hz, 1H), 7.58 (s, 1H), 7.39-7.42 (m, 3H),7.20 (t, *J* = 7.2 Hz, 1H),7.13 (d, *J* = 8.0 Hz, 2H), 6.93 (s, 1H), 6.90 (d, *J* = 8.4 Hz, 1H), 6.01 (br s, 1H), 3.46-3.49 (m, 1H), 2.66-2.72 (m, 1H), 2.41 (s, 3H), 2.38 (s, 3H), 2.32-2.36 (m, 1H), 1.99-2.06 (m, 1H), 1.86-1.90 (m, 1H), 1.69 (s, 3H), 1.56-1.61 (m, 1H), 1.37-1.44 (m, 1H), 1.06-1.22 (m, 1H); **^{13}C NMR** (100 MHz, $CDCl_3$) δ 173.6, 143.8, 139.6, 138.8, 136.5, 135.5, 135.0, 131.8, 130.8, 130.1, 129.5, 128.6, 127.6, 127.4, 127.3, 125.0, 123.9, 121.9, 119.3, 118.5, 117.8, 60.7, 46.9, 34.1, 30.9, 25.9, 21.7, 19.8; **HRMS** (ESI) Calcd for $C_{30}H_{32}N_3O_3S^+$ $[M+H]^+$ 514.2159; Found: 514.2153.

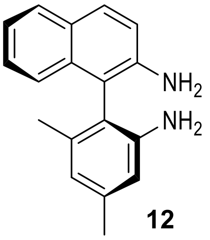

12

(*S*)-1-(2-amino-4,6-dimethylphenyl)naphthalen-2-amine (12)

Deprotection of **10** was operated according to the reported procedures [10]. 82% yield, 96% ee. $[\alpha]_D^{23}$ (c 0.15, $CHCl_3$) = + 170.8.

HPLC condition: Chiralpak AD-H (Hex/*i*PrOH = 80/20, 1.0 mL/min, t_R (major) = 24.1 min, t_R (minor) = 10.4 min).
^{1}H NMR (400 MHz, $CDCl_3$) δ 7.78 (d, *J* = 6.4 Hz, 1H), 7.74 (d, *J* = 6.8 Hz, 1H), 7.24-7.33 (m, 3H),7.10 (d, *J* = 6.8 Hz, 1H), 6.68 (s, 1H), 7.33 (d, *J* = 8.0 Hz, 2H), 7.06-7.09 (m, 3H), 6.98 (d, *J* = 8.0 Hz, 2H), 6.82-6.85 (m, 1H), 6.60 (s, 1H), 3.38 (s, 4H), 2.37 (s, 3H), 1.90 (s, 3H); **^{13}C NMR** (100 MHz, $CDCl_3$) δ 144.7, 141.9, 139.0, 138.5, 133.3, 129.0, 128.4, 128.2, 126.8, 123.5, 122.3, 121.6, 118.2, 118.1, 114.3, 113.9, 21.3, 19.6; **HRMS** (ESI) Calcd for $C_{18}H_{18}N_2Na^+$ $[M+Na]^+$ 285.1362; Found: 285.1357.

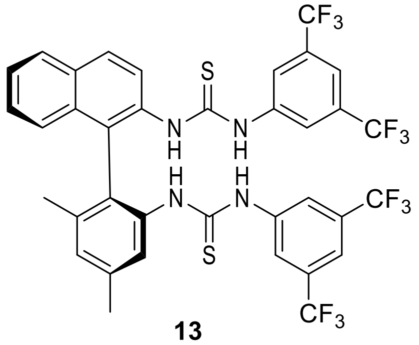

13

(*S*)-1-(3,5-Bis(trifluoromethyl)phenyl)-3-(1-(2-(3-(3,5-bis(trifluoromethyl)phenyl)thioureido)-4,6-dimethylphenyl)naphthalen-2-yl)thiourea (13)

Preparation of **13** from **12** was operated according to the reported procedures [11].
(38.6 mg, 96% yield). $[\alpha]_D^{23}$ (C 0.36, $CHCl_3$) = -111.1.
^{1}H NMR (400 MHz, $CDCl_3$) δ 8.51 (s, 1H), 8.03 (s, 2H), 7.99 (d, *J* = 9.2 Hz, 1H), 7.88 (d, *J* = 8.0 Hz, 1H), 7.73-7.77 (m, 3H), 7.63 (s, 1H), 7.50-7.52 (m, 4H), 7.43 (t, *J* = 8.0 Hz, 1H), 7.20-7.33 (m, 4H), 2.47 (s, 3H), 1.96 (s, 3H); **^{13}C NMR** (100 MHz, $CDCl_3$) δ 180.1, 179.9, 140.8, 140.2, 139.7, 138.8 135.1, 132.9, 132.2, 132.2, 131.9, 131.7, 131.6, 130.5, 129.2, 128.5, 128.3, 127.7, 126.8, 125.4, 125.0, 124.5, 123.2, 121.6, 121.4, 119.5, 21.4, 20.4; **HRMS** (ESI) Calcd for $C_{36}H_{25}F_{12}N_4S_2^+$ $[M+H]^+$ 805.1324; Found: 805.1317.

Supplementary Note 6. Investigation on the racemization for compound **3k**.

We have calculated and located the transition state TS3 for the transformation between products PR and PS. As shown in Supplementary Figure 3, the energy barrier via transition state TS3 for the transformation from PR to PS is 37.4 kcal/mol, indicating the transformation cannot happen at the room temperature.

We next investigated the racemization temperature for compounds **3k**. No racemization was observed after stirring at 140 ℃ for 24 hours (Supplementary Table 3, entry10). Therefore, these results demonstrate that these axial chiral compounds like **3k** have high stability in enantioselectivity.

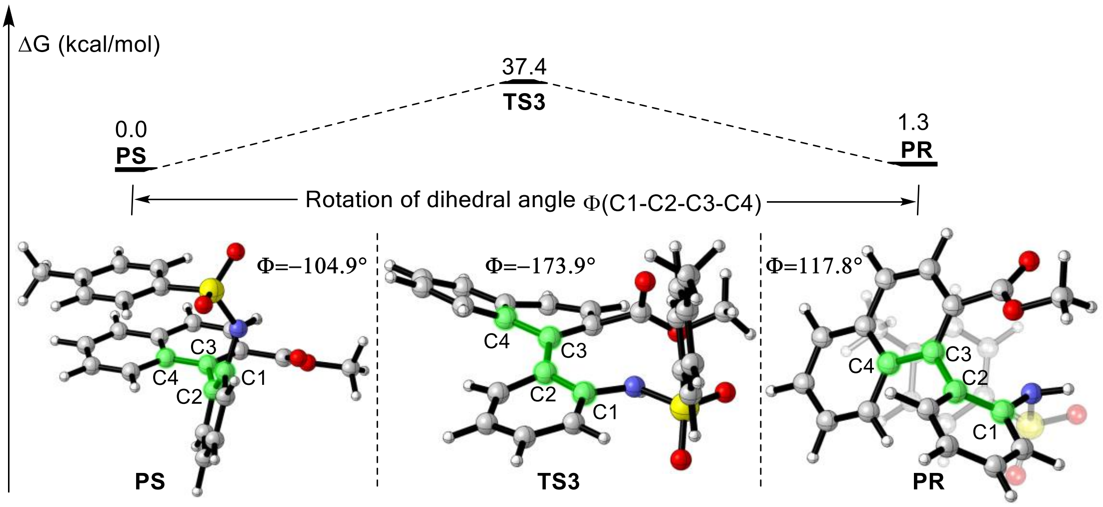

Supplementary Figure 3. Transformation barrier between PR to PS. Transformation between PR to PS via transition state TS3 calculated at the M06-2X-GD3/6-311++G(2d, 2p)/IEF-PCMo-xylene//M06-2X /6-31G(d, p)/IEF-PCMo-xylene level.

Supplementary Table 3. Investigation on the racemization temperature of compound **3k**

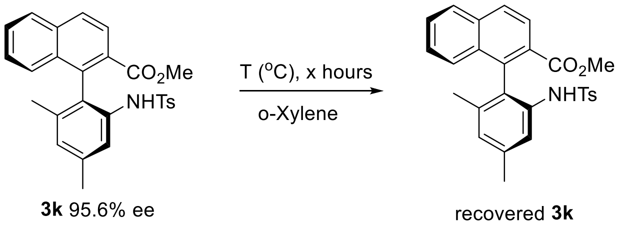

Entry	T (°C)	x (hour)	recovery yield of **3k** (%)[a]	ee of recovered **3k** (%)[b]
1	**40**	24	96	95.6
2	**60**	24	97	95.4
3	**80**	24	96	95.3
4	**100**	24	95	95.3
5	**120**	12	93	95.5
6	**120**	24	90	95.3
7	**140**	4	90	95.5
8	**140**	8	82	95.4
9	**140**	12	71	95.3
10	**140**	24	52	95.3

Reaction conditions: 30.0 mg of enantio-enriched **3k** were stirred in 2.0 mL of o-Xylene. [a] Isolated yield. [b] Determined by chiral HPLC.

Supplementary Note 7. Computational Details.

The DFT calculations were performed using the Gaussian 09 program[12]. All structures were optimized at the M06-2X[13-15]/6-31G(d, p)/IEF-PCM$_{o\text{-}xylene}$ level, and the corresponding vibrational frequencies were calculated at the same level. Then, frequency calculations at the same level of theory were carried out to identify all of

the stationary points as minima (zero imaginary frequency) or transition state (only one frequency), and to provide free energies. We choose to conduct the discussions based on the energy, which is the single-point energy calculated at the M06-2X-GD3/6-311++G(2d, 2p)/IEF-PCM$_{\text{o-xylene}}$[16,17] level plus thermal and entropic corrections to Gibbs free energy at the M06-2X/6-31G(d, p)/IEF-PCM$_{\text{o-xylene}}$ level. The NCI analysis was plotted using Multiwfn[18]. Absolute SPE and GFE of the optimized structures and geometrical coordinates are provided as source data. Source data are provided as a Source Data file.

Supplementary Note 8. Characterization data.

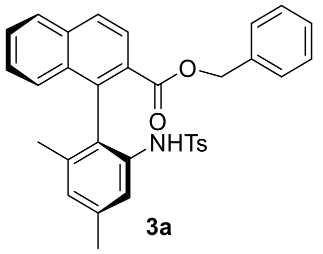

3a

Benzyl (*S*)-1-(2,4-dimethyl-6-((4-methylphenyl)sulfonamido)phenyl)-2-naphthoate (3a): 99% yield, 97% ee. $[\alpha]_D^{23}$ (c 0.41, $CHCl_3$) = + 121.5.
HPLC condition: Chiralpak AS-H (Hex/*i*PrOH = 90/10, 1.0 mL/min, t_R (major) = 37.4 min, t_R (minor) = 48.2 min).
^{1}H NMR (400 MHz, $CDCl_3$) δ 8.05 (d, *J* = 8.4 Hz, 1H), 7.95 (d, *J* = 8.8 Hz, 1H), 7.88 (d, *J* = 8.0 Hz, 1H), 7.52 (t, *J* = 8.0 Hz, 1H), 7.42 (s, 1H), 7.31-7.35 (m, 5H), 7.12-7.15 (m, 2H), 7.10 (t, *J* = 8.0 Hz, 1H), 6.98 (d, *J* = 7.6 Hz, 2H), 6.85 (d, *J* = 8.4 Hz, 1H), 6.74 (s, 1H), 6.06 (s, 1H), 5.13 (d, *J* = 12.0 Hz, 1H), 4.96 (d, *J* = 12.0 Hz, 1H), 2.36 (s, 3H), 2.34 (s, 3H), 1.57 (s, 3H); **^{13}C NMR (100 MHz, $CDCl_3$)** δ 167.0, 143.3, 138.2, 137.2, 136.6, 135.4, 135.2, 135.2, 134.5, 131.9, 129.4, 129.1, 128.9, 128.5, 128.4, 128.2, 128.1, 127.9, 127.4, 127.1, 126.9, 126.1, 126.1, 117.8, 67.2, 21.6, 21.6, 19.9; **HRMS (ESI)** Calcd for $C_{33}H_{29}NNaO_4S^+$ $[M+Na]^+$ 558.1710; Found: 558.1721.

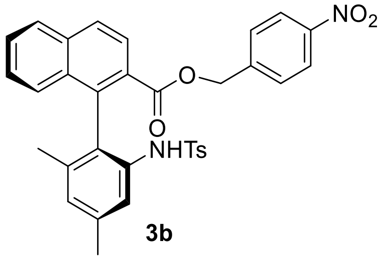

3b

4-Nitrobenzyl (*S*)-1-(2,4-dimethyl-6-((4-methylphenyl)sulfonamido)phenyl)-2-naphthoate (3b): 99% yield, 96% ee, $[\alpha]_D^{23}$ (c 0.53, $CHCl_3$) = + 67.2.
HPLC condition: Chiralpak AZ-H (Hex/*i*PrOH = 80/20, 1.0 mL/min, t_R (major) = 89.0 min, t_R (minor) = 59.9 min).
^{1}H NMR (400 MHz, $CDCl_3$) δ 8.12 (d, *J* = 8.8 Hz, 2H), 8.04 (d, *J* = 8.4 Hz, 1H), 7.97 (d, *J* = 8.8 Hz, 1H), 7.89 (d, *J* = 8.0 Hz, 1H), 7.53 (t, *J* = 7.2 Hz, 1H),7.32-7.35 (m, 3H),7.21 (d, *J* = 8.4 Hz, 2H), 7.10 (t, *J* = 8.0 Hz, 1H),7.00 (d, *J* = 8.0 Hz, 2H), 6.82 (d, *J* = 8.4 Hz, 1H), 6.73 (s, 1H), 6.05 (s, 1H), 5.20 (d, *J* = 12.8 Hz, 1H), 5.06 (d, *J* = 12.8 Hz, 1H), 2.34 (s, 3H), 2.31 (s, 3H), 1.57 (s, 3H); **^{13}C NMR (100 MHz, $CDCl_3$)** δ 166.9, 147.6, 143.5, 142.4, 136.6, 138.4, 137.3, 136.5, 135.3, 135.3, 134.7, 131.8, 129.4, 129.3, 128.8, 128.5, 128.2, 128.1, 127.6, 127.1, 126.7, 126.0, 126.0, 125.8, 123.6, 117.4, 65.7, 21.6, 19.9; **HRMS (ESI)** Calcd for $C_{33}H_{28}N_2NaO_6S^+$ $[M+Na]^+$ 603.1560; Found: 603.1571.

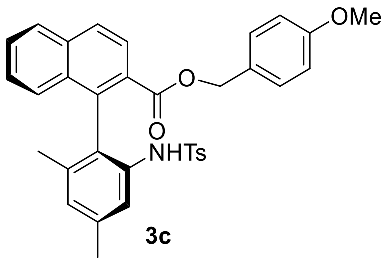

3c

4-Methoxybenzyl (*S*)-1-(2,4-dimethyl-6-((4-methylphenyl)sulfonamido)phenyl)-2-naphthoate (3c): 99% yield, 94% ee, $[\alpha]_D^{23}$ (c 0.43, $CHCl_3$) = - 28.8.
HPLC condition: Chiralpak AS-H (Hex/*i*PrOH = 80/20, 1.0 mL/min, t_R (major) = 21.3 min, t_R (minor) = 30.0 min).
^{1}H NMR (400 MHz, $CDCl_3$) δ 8.02 (d, *J* = 8.8 Hz, 1H), 7.94 (d, *J* = 8.4 Hz, 1H), 7.87 (d, *J* = 8.0 Hz, 1H), 7.51 (t, *J* = 7.6 Hz, 1H), 7.40 (s, 1H), 7.33 (d, *J* = 8.0 Hz, 2H), 7.06-7.09 (m, 3H), 6.98 (d, *J* = 8.0 Hz, 2H), 6.82-6.85 (m, 3H), 6.74 (s, 1H), 6.07 (s, 1H), 5.07 (d, *J* = 12.0 Hz, 1H), 4.88 (d, *J* = 12.0 Hz, 1H), 3.82 (s, 3H), 2.36 (s, 3H), 2.34 (s, 3H), 1.55 (s, 3H); **^{13}C NMR (100 MHz, $CDCl_3$)** δ 167.1, 159.6, 143.3, 138.2, 137.2, 136.7, 135.3, 135.1, 134.5, 131.9, 130.3, 129.4, 129.1, 129.0, 128.1, 127.8, 127.4, 127.3, 127.1, 126.9, 126.1, 126.1, 126.0, 117.8, 113.7, 77.3, 67.0, 55.3, 21.6, 21.6, 19.9; **HRMS (ESI)** Calcd for $C_{34}H_{31}NNaO_5S^+$ $[M+Na]^+$ 588.1815; Found: 588.1816.

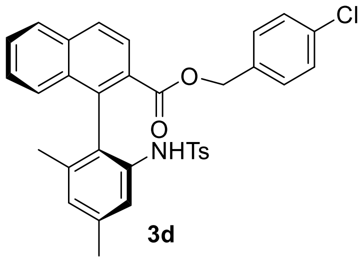

3d

4-Chlorobenzyl (*S*)-1-(2,4-dimethyl-6-((4-methylphenyl)sulfonamido)phenyl)-2-naphthoate (3d): 99% yield, 94% ee. $[\alpha]_D^{23}$ (c 0.45, $CHCl_3$) = - 11.4.

HPLC condition: Chiralpak AS-H (Hex/*i*PrOH = 80/20, 0.8 mL/min, t_R (major) = 26.3 min, t_R (minor) = 31.5 min).
^{1}H NMR (400 MHz, $CDCl_3$) δ 8.02 (d, *J* = 8.8 Hz, 1H), 7.96 (d, *J* = 8.8 Hz, 1H), 7.88 (d, *J* = 8.0 Hz, 1H),7.52 (t, *J* = 8.0 Hz, 1H), 7.37 (s, 1H), 7.33 (d, *J* = 8.4 Hz, 2H), 7.24-7.27 (m, 2H), 7.09 (t, *J* = 7.2 Hz, 1H), 6.98-7.03 (m, 4H), 6.82 (d, *J* = 8.8 Hz, 1H), 6.71 (s, 1H), 6.03 (s, 1H), 5.08 (d, *J* = 12.4 Hz, 1H), 4.90 (d, *J* = 12.4 Hz, 1H), 2.34 (s, 6H), 1.55 (s, 3H); **^{13}C NMR (100 MHz, $CDCl_3$)** δ 167.1, 143.4, 138.3, 137.2, 136.6, 135.2, 135.2, 134.5, 134.1, 133.6, 131.9, 129.9, 129.4, 129.1, 128.8, 128.5, 128.2, 127.9, 127.4, 127.1, 126.8, 126.1, 126.0, 125.9, 117.5, 77.3, 66.4, 21.6, 21.6, 19.9; **HRMS (ESI)** Calcd for $C_{33}H_{28}ClNNaO_4S^+$ $[M+Na]^+$ 592.1320; Found: 592.1332.

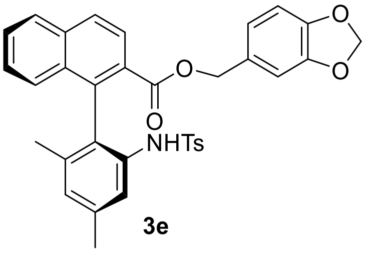

3e

Benzo[*d*][1,3]dioxol-5-ylmethyl (*S*)-1-(2,4-dimethyl-6-((4-methylphenyl)sulfonamido)phenyl)-2-naphthoate (3e): 99% yield, 92% ee. $[\alpha]_D^{23}$ (c 0.51, $CHCl_3$) = - 16.8.
HPLC condition: Chiralpak AS-H (Hex/*i*PrOH = 80/20, 1.0 mL/min, t_R (major) = 30.1 min, t_R (minor) = 46.0 min).
^{1}H NMR (400 MHz, $CDCl_3$) δ 8.02 (d, *J* = 8.4 Hz, 1H), 7.95 (d, *J* = 8.8 Hz, 1H), 7.87 (d, *J* = 8.0 Hz, 1H), 7.52 (t, *J* = 8.0 Hz, 1H), 7.41 (s, 1H), 7.33 (d, *J* = 8.4 Hz, 2H), 7.09 (t, *J* = 8.4 Hz, 1H), 6.99 (d, *J* = 7.6 Hz, 2H), 6.84 (d, *J* = 8.4 Hz, 1H), 6.76 (s, 1H), 6.73 (d, *J* = 8.0 Hz, 1H), 6.64 (d, *J* = 8.0 Hz, 1H), 6.54 (s, 1H), 6.03 (s, 1H), 5.98 (s, 2H), 5.03 (d, *J* = 11.6 Hz, 1H), 4.83 (d, *J* = 11.6 Hz, 1H), 2.37 (s, 3H), 2.35 (s, 3H), 1.56 (s, 3H); **^{13}C NMR (100 MHz, $CDCl_3$)** δ 167.1, 147.6, 147.6, 143.3, 138.3, 137.3, 136.7, 135.2, 135.1, 134.6,131.9, 129.4, 129.0, 129.0, 128.9, 128.1, 127.8, 127.4, 127.1, 126.9, 126.1, 126.0, 122.6, 117.7, 109.3, 108.0, 101.1, 67.2, 21.6, 19.9; **HRMS (ESI)** Calcd for $C_{34}H_{29}NNaO_6S^+$ $[M+Na]^+$ 602.1608; Found: 602.1613.

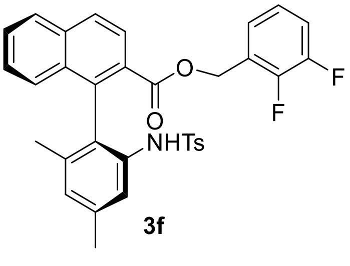

3f

2,3-Difluorobenzyl (*S*)-1-(2,4-dimethyl-6-((4-methylphenyl)sulfonamido)phenyl)-2-naphthoate (3f): 99% yield, 96% ee. $[\alpha]_D^{23}$ (c 0.43, $CHCl_3$) = + 113.8.

HPLC condition: Chiralpak AZ-H (Hex/*i*PrOH = 80/20, 1.0 mL/min, t_R (major) = 29.1 min, t_R (minor) = 20.3 min).

^{1}H NMR (400 MHz, $CDCl_3$) δ 8.06 (d, *J* = 8.8 Hz, 1H), 7.97 (d, *J* = 8.8 Hz, 1H), 7.89 (d, *J* = 8.0 Hz, 1H),7.53 (t, *J* = 8.0 Hz, 1H), 7.31-7.34 (m, 3H), 6.99-7.17 (m, 5H), 6.82-6.88 (m, 2H), 6.72 (s, 1H), 5.99 (s, 1H), 5.22 (d, *J* = 12.4 Hz, 1H), 4.98 (d, *J* = 12.4 Hz, 1H), 2.35 (s, 3H), 2.31 (s, 3H), 1.57 (s, 3H); **^{13}C NMR (100 MHz, $CDCl_3$)** δ 166.8, 143.4, 138.3, 137.2, 136.6, 135.6. 135.3, 134.4, 131.9, 129.4, 129.1, 128.4, 128.2, 128.0, 127.5, 127.1, 126.7, 126.1, 125.9, 125.5, 124.8, 124.7, 124.1, 124.0, 124.0, 117.5, 117.3, 60.3, 21.5, 19.9; **HRMS (ESI)** Calcd for $C_{33}H_{27}F_2NNaO_4S^+$ $[M+Na]^+$ 594.1521; Found: 594.1523.

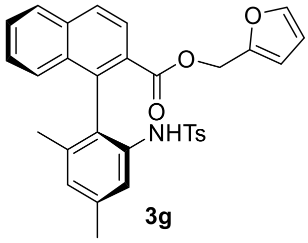

3g

Furan-2-ylmethyl (*S*)-1-(2,4-dimethyl-6-((4-methylphenyl)sulfonamido)phenyl)-2-naphthoate (3g): 99% yield, 92% ee. $[\alpha]_D^{23}$ (0.39, $CHCl_3$) = + 74.3.

HPLC condition: Chiralpak AS-H (Hex/*i*PrOH = 80/20, 0.8 mL/min, t_R (major) = 30.9 min, t_R (minor) = 34.5 min).

^{1}H NMR (400 MHz, $CDCl_3$) δ 8.02 (d, *J* = 8.8 Hz, 1H), 7.93 (d, *J* = 8.4 Hz, 1H), 7.87 (d, *J* = 8.0 Hz, 1H), 7.52 (t, *J* = 8.0 Hz, 1H), 7.42 (s, 1H), 7.39 (s, 1H), 7.33 (d, *J* = 8.4 Hz, 2H), 7.09 (t, *J* = 8.0 Hz, 1H), 7.00 (d, *J* = 8.0 Hz, 2H), 6.87 (d, *J* = 8.4 Hz, 1H), 6.78 (s, 1H), 6.26-6.34 (m, 2H), 6.01 (s, 1H), 5.08 (d, *J* = 13.2 Hz, 1H), 4.90 (d, *J* = 13.2 Hz, 1H), 2.36 (s, 3H), 2.35 (s, 3H), 1.58 (s, 3H); **^{13}C NMR (100 MHz, $CDCl_3$)** δ 166.6, 148.9, 143.3, 143.2, 138.2, 137.2, 136.7, 135.8, 135.2, 134.4, 131.9, 129.4, 129.0, 128.5, 128.1, 127.9, 127.4, 127.1, 126.9, 126.2, 126.1, 126.1, 118.0, 110.7, 110.5, 58.7, 21.6, 21.6, 19.9; **HRMS (ESI)** Calcd for $C_{31}H_{27}NNaO_5S^+$ $[M+Na]^+$ 548.1502; Found: 548.1500.

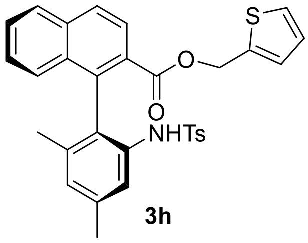

3h

Thiophen-2-ylmethyl (*S*)-1-(2,4-dimethyl-6-((4-methylphenyl)sulfonamido)phenyl)-2-naphthoate (3h): 99% yield, 96% ee. $[\alpha]_D^{23}$ (c 0.41, $CHCl_3$) = + 48.1

HPLC condition: Chiralpak AZ-H (Hex/*i*PrOH = 80/20, 1.0 mL/min, t_R (major) = 34.5 min, t_R (minor) = 23.5 min).

^{1}H NMR (400 MHz, $CDCl_3$) δ 8.02 (d, *J* = 8.4 Hz, 1H), 7.95 (d, *J* = 8.8 Hz, 1H), 7.88 (d, *J* = 8.4 Hz, 1H), 7.52 (t, *J* = 8.0 Hz, 1H), 7.43 (s, 1H), 7.34 (d, *J* = 8.4 Hz,

2H), 7.24-7.26 (m, 1H), 7.07-7.11 (m, 2H), 6.99 (d, *J* = 8.4 Hz, 2H), 6.90 (d, *J* = 5.2 Hz, 1H), 6.85 (d, *J* = 8.4 Hz, 1H), 6.76 (s, 1H), 6.06 (s, 1H),5.13 (d, *J* = 12.4 Hz, 1H), 4.95 (d, *J* = 12.4 Hz, 1H), 2.37 (s, 3H), 2.34 (s, 3H), 1.56 (s, 3H); **^{13}C NMR (100 MHz, $CDCl_3$)** δ 167.0,143.3, 138.3, 137.3, 136.6, 136.0, 135.3, 135.1, 134.5, 131.9, 129.4, 129.1, 128.9, 128.2, 127.9, 127.7, 127.4, 127.1, 126.9, 126.1, 126.1, 126.0, 125.9, 124.7, 117.8, 62.0, 21.7, 21.6, 19.9; **HRMS (ESI)** Calcd for $C_{31}H_{27}NNaO4S_2^+$ $[M+Na]^+$ 564.1274; Found: 564.1288.

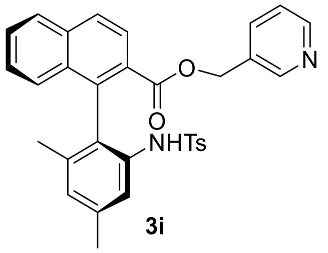

3i

Pyridin-3-ylmethyl (*S*)-1-(2,4-dimethyl-6-((4-methylphenyl)sulfonamido)phenyl)-2-naphthoate (3i): 99% yield, 95% ee. $[\alpha]_D^{23}$ (c 0.38, $CHCl_3$) = + 109.1.

HPLC condition: Chiralpak AD (Hex/*i*PrOH = 80/20, 1.0 mL/min, t_R (major) = 31.1 min, t_R (minor) = 35.0 min).

^{1}H NMR (400 MHz, $CDCl_3$) δ 8.55 (d, *J* = 5.6 Hz, 1H), 8.34 (s, 1H), 8.02 (d, *J* = 8.4 Hz, 1H), 7.95 (d, *J* = 8.8 Hz, 1H), 7.87 (d, *J* = 8.0 Hz, 1H), 7.52 (t, *J* = 8.0 Hz, 1H), 7.40 (d, *J* = 8.0 Hz, 1H), 7.32-7.34 (m, 3H), 7.20-7.26 (m, 1H), 7.09 (t, *J* = 8.4 Hz, 1H), 7.00 (d, *J* = 8.0 Hz, 2H), 6.83 (d, *J* = 8.4 Hz, 1H), 6.68 (s, 1H), 6.02 (s, 1H),5.11 (d, *J* = 12.4 Hz, 1H), 4.90 (d, *J* = 12.4 Hz, 1H), 2.34 (s, 3H), 2.32 (s, 3H), 1.53 (s, 3H); **^{13}C NMR (100 MHz, $CDCl_3$)** δ 167.0, 149.8, 149.6, 143.4, 138.4, 137.2, 136.6, 136.4, 135.4, 135.3, 134.5, 131.8, 130.7, 129.4, 129.2, 128.6, 128.2, 128.0, 127.5, 127.1, 126.7, 126.1, 126.1, 125.8, 123.4, 117.4, 77.3, 64.5, 21.6, 21.6, 19.9; **HRMS (ESI)** Calcd for $C_{32}H_{29}N_2O_4S^+$ $[M+H]^+$ 537.1843; Found: 537.1851.

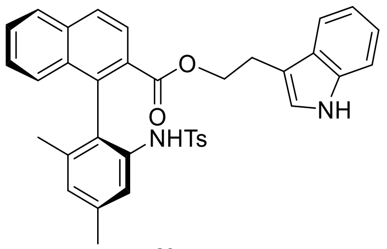

3j

(1*H*-indol-3-yl)methyl (*S*)-1-(2,4-dimethyl-6-((4-methylphenyl)sulfonamido)phenyl)-2-naphthoate (3j): 97% yield, 96% ee. $[\alpha]_D^{23}$ (c 0.46, $CHCl_3$) = + 88.4.

HPLC condition: Chiralpak OD-H (Hex/*i*PrOH = 80/20, 1.0 mL/min, t_R (major) = 17.5 min, t_R (minor) = 21.0 min).

^{1}H NMR (400 MHz, $CDCl_3$) δ 8.24 (s, 1H), 8.01 (d, *J* = 8.4 Hz, 1H), 7.95 (d, *J* = 8.8 Hz, 1H), 7.89 (d, *J* = 8.0 Hz, 1H), 7.50-7.56 (m, 3H),7.36 (d, *J* = 8.4 Hz, 3H), 7.22 (t, *J* = 6.8 Hz, 1H), 7.10-7.17 (m, 2H), 6.90-6.98 (m, 4H), 6.86 (s, 1H), 6.18 (s, 1H),

4.35-4.41 (m, 1H), 4.21-4.27 (m, 1H), 2.73-2.94 (m, 2H), 2.38 (s, 3H), 2.27 (s, 3H), 1.65 (s, 3H); **^{13}C NMR (100 MHz, CDCl$_3$)** δ 167.4, 143.5, 138.4, 137.5, 136.5, 136.3, 135.2, 134.7, 131.9, 129.4, 129.3, 129.1, 128.2, 127.8, 127.4, 127.1, 126.9, 126.2, 126.1, 126.1, 122.2, 122.0, 119.3, 118.6, 117.6, 111.4, 111.3, 65.1, 24.2, 21.6, 21.5, 20.0; **HRMS (ESI)** Calcd for $C_{36}H_{32}N_2NaO_4S^+$ $[M+Na]^+$ 611.1975; Found: 611.1969.

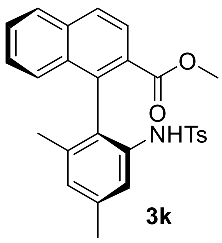

3k

Methyl (*S*)-1-(2,4-dimethyl-6-((4-methylphenyl)sulfonamido)phenyl)-2-naphthoate (3k): 99% yield, 96% ee. $[\alpha]_D^{23}$ (c 0.35, CHCl$_3$) = + 56.5.
HPLC condition: Chiralpak AS-H (Hex/*i*PrOH = 80/20, 1.0 mL/min, t_R (major) = 18.8 min, t_R (minor) = 28.9 min).
^{1}H NMR (400 MHz, CDCl$_3$) δ 8.00 (d, *J* = 8.4 Hz, 1H), 7.96 (d, *J* = 8.8 Hz, 1H), 7.88 (d, *J* = 8.4 Hz, 1H), 7.49-7.54 (m, 2H), 7.33 (d, *J* = 8.4 Hz, 2H), 7.11 (t, *J* = 8.0 Hz, 1H), 6.99 (d, *J* = 8.0 Hz, 2H), 6.88 (d, *J* = 8.8 Hz, 2H), 6.08 (s, 1H), 3.62 (s, 3H), 2.39 (s, 3H), 2.35 (s, 3H), 1.62 (s, 3H); **^{13}C NMR (100 MHz, CDCl$_3$)** δ 167.3, 143.3, 138.3, 137.2, 136.6, 135.7, 135.1, 134.6, 131.9, 129.3, 129.0, 128.7, 128.1, 127.9, 127.4, 127.1, 126.9, 126.1, 125.9, 117.9, 52.3, 21.6, 21.6, 20.0; **HRMS (ESI)** Calcd for $C_{27}H_{25}NNaO_4S^+$ $[M+Na]^+$ 482.1397; Found: 482.1391.

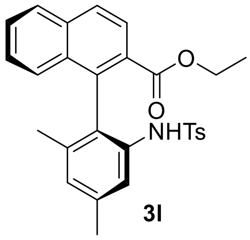

3l

Ethyl (*S*)-1-(2,4-dimethyl-6-((4-methylphenyl)sulfonamido)phenyl)-2-naphthoate (3l): 99% yield, 96% ee. $[\alpha]_D^{23}$ (c 0.35, CHCl$_3$) = + 6.87.
HPLC condition: Chiralpak AZ-H (Hex/*i*PrOH = 90/10, 1.0 mL/min, t_R (major) = 45.1 min, t_R (minor) = 31.5 min).
^{1}H NMR (400 MHz, CDCl$_3$) δ 8.01 (d, *J* = 8.8 Hz, 1H), 7.95 (d, *J* = 8.4 Hz, 1H), 7.88 (d, *J* = 8.4 Hz, 1H), 7.48-7.54 (m, 2H), 7.33 (d, *J* = 8.4 Hz, 2H), 7.09 (t, *J* = 8.0 Hz, 1H), 6.99 (d, *J* = 8.0 Hz, 2H), 6.87 (d, *J* = 8.8 Hz, 2H), 6.07 (s, 1H), 4.06-4.14 (m, 1H), 3.94-4.02 (m, 1H), 2.39 (s, 3H), 2.35 (s, 3H), 1.63 (s, 3H), 0.99 (t, *J* = 7.2 Hz, 3H); **^{13}C NMR (100 MHz, CDCl$_3$)** δ 167.2, 143.3, 138.3, 137.3, 136.6, 135.1, 134.7, 131.9, 129.3, 129.3, 129.0,128.1, 127.7, 127.3, 127.1, 126.8, 126.2, 126.1, 126.0, 117.6, 61.2, 21.6, 21.6, 19.9, 13.6; **HRMS (ESI)** Calcd for $C_{28}H_{27}NNaO_4S^+$ $[M+Na]^+$ 496.1553; Found: 496.1557.

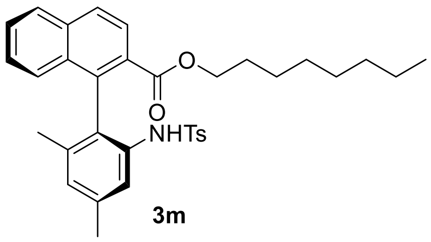

3m

Octyl (*S*)-1-(2,4-dimethyl-6-((4-methylphenyl)sulfonamido)phenyl)-2-naphthoate (3m): 98% yield, 96% ee. $[\alpha]_D^{23}$ (c 0.42, $CHCl_3$) = + 52.5.

HPLC condition: Chiralpak AZ-H (Hex/*i*PrOH = 90/10, 1.0 mL/min, t_R (major) = 32.0 min, t_R (minor) = 21.7 min).

^{1}H NMR (400 MHz, CDCl$_3$) δ 8.02 (d, *J* = 8.4 Hz, 1H), 7.95 (d, *J* = 8.8 Hz, 1H), 7.88 (d, *J* = 8.0 Hz, 1H),7.49-7.53 (m, 2H), 7.33 (d, *J* = 8.0 Hz, 2H), 7.08 (t, *J* = 8.0 Hz, 1H), 6.99 (d, *J* = 8.0 Hz, 2H), 6.84 (d, *J* = 8.4 Hz, 2H), 6.08 (s, 1H), 4.02-4.08 (m, 1H), 3.89-3.95 (m, 1H), 2.39 (s, 3H), 2.35 (s, 3H), 1.63 (s, 3H), 1.16-1.35 (m, 12H), 0.99 (t, *J* = 7.2 Hz, 3H); **^{13}C NMR (100 MHz, CDCl$_3$)** δ 167.4, 143.3, 138.3, 137.3, 136.6, 135.1, 135.0, 134.7, 131.9, 129.3, 129.0, 128.1, 127.7, 127.3, 127.1, 126.8, 126.2, 126.1, 126.0, 117.7, 65.6, 31.9, 29.3, 29.2, 28.3, 25.9, 22.7, 21.6, 21.6, 20.0, 14.2; **HRMS (ESI)** Calcd for $C_{34}H_{39}NNaO_4S^+$ $[M+Na]^+$ 580.2492; Found: 580.2490.

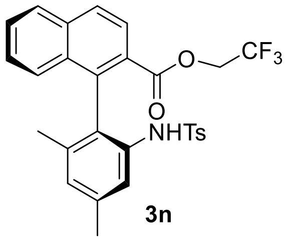

3n

2,2,2-Frifluoroethyl (*S*)-1-(2,4-dimethyl-6-((4-methylphenyl)sulfonamido)phenyl)-2-naphthoate (3n): 99% yield, 91% ee. $[\alpha]_D^{23}$ (c 0.41, $CHCl_3$) = + 111.0.

HPLC condition: Chiralpak AS-H (Hex/*i*PrOH = 80/20, 1.0 mL/min, t_R (major) = 13.1 min, t_R (minor) = 39.4 min).

^{1}H NMR (400 MHz, CDCl$_3$) δ 8.07 (d, *J* = 8.8 Hz, 1H), 7.99 (d, *J* = 8.8 Hz, 1H), 7.91 (d, *J* = 8.0 Hz, 1H),7.57 (t, *J* = 8.0 Hz, 1H), 7.47(s, 1H), 7.37 (d, *J* = 8.4 Hz, 2H), 7.17 (t, *J* = 8.4 Hz, 1H), 7.03 (d, *J* = 8.0 Hz, 2H), 6.95 (d, *J* = 8.4 Hz, 1H), 6.89 (s, 1H), 5.91 (s, 1H), 4.41-4.50 (m, 1H), 4.27-4.37 (m, 1H), 2.39 (s, 3H), 2.37 (s, 3H),1.63 (s, 3H); **^{13}C NMR (100 MHz, CDCl$_3$)** δ 164.9, 143.4, 138.6, 137.1, 137.1, 136.6, 135.6, 134.4, 131.9, 129.4, 129.3, 128.5, 128.2, 127.7, 127.2, 127.1, 126.5, 126.3, 126.0, 125.6, 117.8, 60.9 ($q_{C\text{-}F}$, *J* = 146 Hz, 292 Hz), 21.6, 21.5, 19.9; **HRMS (ESI)** Calcd for $C_{28}H_{24}F_3NNaO_4S^+$ $[M+Na]^+$ 550.1270; Found: 550.1275.

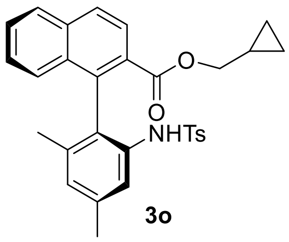

3o

Cyclopropylmethyl (*S*)-1-(2,4-dimethyl-6-((4-methylphenyl)sulfonamido)phenyl)-2-naphthoate (3o): 99% yield, 98% ee. $[\alpha]_D^{23}$ (c 0.38, $CHCl_3$) = + 91.0.

HPLC condition: Chiralpak AS-H (Hex/*i*PrOH = 90/10, 1.0 mL/min, t_R (major) = 23.3 min, t_R (minor) = 32.0 min).

^{1}H NMR (400 MHz, $CDCl_3$) δ 8.02 (d, *J* = 8.8 Hz, 1H), 7.96 (d, *J* = 8.4 Hz, 1H), 7.88 (d, *J* = 8.0 Hz, 1H),7.49-7.53 (m, 2H), 7.32 (d, *J* = 8.0 Hz, 2H), 7.08 (t, *J* = 8.4 Hz, 1H), 6.98 (d, *J* = 8.4 Hz, 2H), 6.85 (s, 1H), 6.84 (d, *J* = 8.0 Hz, 1H), 6.12 (s, 1H), 3.87-3.91 (m, 1H), 3.73-3.78 (m, 1H), 2.38 (s, 3H), 2.34 (s, 3H), 1.64 (s, 3H), 0.82-0.92 (m, 1H), 0.41-0.46 (m, 2H), 0.06-0.14 (m, 2H); **^{13}C NMR (100 MHz, $CDCl_3$)** δ 167.5, 143.3, 138.2, 137.4, 136.6, 135.1, 135.0, 134.7, 131.9, 129.5, 129.3, 129.0, 128.1, 127.7, 127.3, 127.1, 126.9, 126.2, 126.1, 126.0, 117.8, 70.3, 21.6, 21.6, 20.0, 9.4, 3.3, 3.1; **HRMS (ESI)** Calcd for $C_{30}H_{29}NNaO_4S^+$ $[M+Na]^+$ 522.1710; Found: 522.1714.

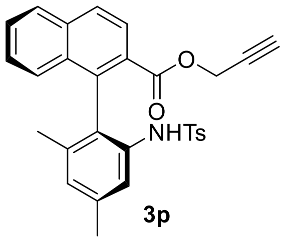

3p

Prop-2-yn-1-yl (*S*)-1-(2,4-dimethyl-6-((4-methylphenyl)sulfonamido)phenyl)-2-naphthoate (3p): 99% yield, 96% ee. $[\alpha]_D^{23}$ (c 0.40, $CHCl_3$) = + 66.9.

HPLC condition: Chiralpak AS-H (Hex/*i*PrOH = 80/20, 1.0 mL/min, t_R (major) = 28.5 min, t_R (minor) = 48.5 min).

^{1}H NMR (400 MHz, $CDCl_3$) δ 8.06 (d, *J* = 8.8 Hz, 1H), 7.96 (d, *J* = 8.4 Hz, 1H), 7.89 (d, *J* = 8.0 Hz, 1H),7.55 (t, *J* = 8.0 Hz, 1H), 7.47 (s, 1H), 7.36 (d, *J* = 8.0 Hz, 2H), 7.14 (t, *J* = 8.4 Hz, 1H), 7.02 (d, *J* = 8.0 Hz, 2H), 6.93 (d, *J* = 8.8 Hz, 1H), 6.87 (s, 1H), 5.98 (s, 1H), 4.53-4.66 (m, 2H), 2.44 (t, *J* = 2.4 Hz, 1H), 2.39 (s, 3H), 2.36 (s, 3H), 1.63 (s, 3H); **^{13}C NMR (100 MHz, $CDCl_3$)** δ 165.8, 143.3, 138.4, 137.2, 136.6, 136.3, 135.3, 134.5, 131.9, 129.4, 129.1, 128.2, 128.2, 127.7, 127.5, 127.1, 127.0, 126.2, 125.9, 117.9, 77.2, 75.1, 52.6, 21.6, 21.6, 20.0; **HRMS (ESI)** Calcd for $C_{29}H_{25}NNaO_4S^+$ $[M+Na]^+$ 506.1397; Found: 506.1399.

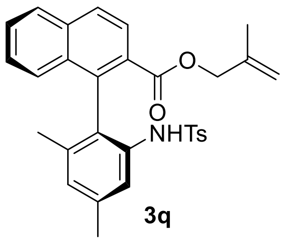

3q

2-Methylallyl (*S*)-1-(2,4-dimethyl-6-((4-methylphenyl)sulfonamido)phenyl)-2-naphthoate (3q): 97% yield, 93% ee. $[\alpha]_D^{23}$ (c 0.39, $CHCl_3$) = + 71.5.

HPLC condition: Chiralpak AS-H (Hex/*i*PrOH = 90/10, 1.0 mL/min, t_R (major) = 27.7 min, t_R (minor) = 31.2 min).

^{1}H NMR (400 MHz, $CDCl_3$) δ 8.05 (d, *J* = 8.4 Hz, 1H), 7.96 (d, *J* = 8.4 Hz, 1H), 7.88 (d, *J* = 8.0 Hz, 1H),7.52 (t, *J* = 7.2 Hz, 1H), 7.49 (s, 1H), 7.33 (d, *J* = 8.4 Hz, 2H), 7.09 (t, *J* = 8.4 Hz, 1H), 6.98 (d, *J* = 8.0 Hz, 2H), 6.86(d, *J* = 8.4 Hz, 2H), 6.12 (s, 1H), 4.89 (s, 2H), 4.48 (q, *J* = 12.8 Hz, 26.8Hz, 2H), 2.39 (s, 3H), 2.35 (s, 3H),1.68 (s, 3H), 1.64 (s, 3H); **^{13}C NMR (100 MHz, $CDCl_3$)** δ 166.7, 143.3, 139.5, 138.4, 137.2, 136.6, 135.6, 135.1, 134.6, 131.9, 129.3, 129.0, 128.8, 128.1, 127.8, 127.4, 127.1, 127.0, 126.1, 125.9, 118.0, 113.6, 68.8, 21.6, 21.6, 20.0, 19.5; **HRMS (ESI)** Calcd for $C_{30}H_{29}NNaO_4S^+$ $[M+Na]^+$ 522.1710; Found:522.1704.

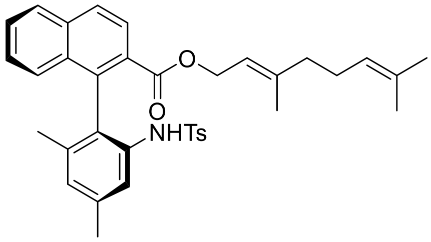

3r

(*E*)-3,7-dimethylocta-2,6-dien-1-yl (*S*)-1-(2,4-dimethyl-6-((4-methylphenyl)sulfonamido)phenyl)-2-naphthoate (3r): 99% yield, 96% ee. $[\alpha]_D^{23}$ (c 0.43, $CHCl_3$) = + 100.7.

HPLC condition: Chiralpak AS-H (Hex/*i*PrOH = 90/10, 1.0 mL/min, t_R (major) = 11.0 min, t_R (minor) = 15.5 min).

^{1}H NMR (400 MHz, $CDCl_3$) δ 8.01 (d, *J* = 8.8 Hz, 1H), 7.95 (d, *J* = 8.8 Hz, 1H), 7.88 (d, *J* = 8.0 Hz, 1H), 7.48-7.53 (m, 2H), 7.33 (d, *J* = 8.4 Hz, 2H), 7.08 (t, *J* = 8.4 Hz, 1H), 6.98 (d, *J* = 8.4 Hz, 2H), 6.85-6.86(m, 2H), 6.09 (s, 1H), 5.01-5.12 (m, 2H), 4.58-4.63 (m, 1H), 4.40-4.45 (m, 1H), 2.38 (s, 3H), 2.34 (s, 3H), 2.05-2.10 (m, 2H), 1.97-2.01 (m, 2H), 1.70 (s, 3H), 1.61-1.63 (m, 9H); **^{13}C NMR (100 MHz, $CDCl_3$)** δ 167.3, 143.2, 142.3, 138.2, 137.3, 136.7, 135.1, 135.0, 134.7, 131.9, 131.8, 129.3, 129.0, 128.1, 127.7, 127.3, 127.1, 126.9, 126.3, 126.1, 126.0, 123.8, 117.9, 117.7, 62.2, 39.5, 26.3, 25.8, 21.6, 20.0, 17.8, 16.4; **HRMS (ESI)** Calcd for $C_{36}H_{39}NNaO_4S^+$ $[M+Na]^+$ 604.2492; Found: 604.2489.

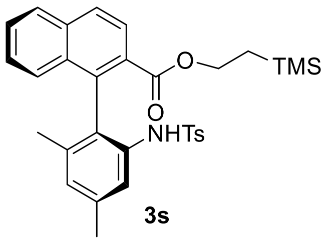

3s

2-(Trimethylsilyl)ethyl (*S*)-1-(2,4-dimethyl-6-((4-methylphenyl)sulfonamido)phenyl)-2-naphthoate (3s): 93% yield, 96% ee. $[\alpha]_D^{23}$ (c 0.39, $CHCl_3$) = + 83.5.

HPLC condition: Chiralpak AS-H (Hex/*i*PrOH = 90/10, 1.0 mL/min, t_R (major) = 9.3 min, t_R (minor) = 14.1 min).

^{1}H NMR (400 MHz, $CDCl_3$) δ 7.99 (d, *J* = 8.8 Hz, 1H), 7.95 (d, *J* = 8.8 Hz, 1H), 7.87 (d, *J* = 8.0 Hz, 1H),7.48-7.53 (m, 2H), 7.33 (d, *J* = 8.4 Hz, 2H), 7.08 (t, *J* = 7.2 Hz, 1H), 6.97 (d, *J* = 8.4 Hz, 2H), 6.85-6.87 (m, 2H), 6.12 (s, 1H), 4.11-4.19 (m, 1H), 3.99-4.06 (m, 1H), 2.38 (s, 3H), 2.34 (s, 3H), 1.63 (s, 3H), 0.73-0.78 (m, 2H), 0.01 (s, 9H); **^{13}C NMR (100 MHz, $CDCl_3$)** δ 167.4, 143.2, 138.2, 137.4, 136.7, 135.1, 135.0, 134.7, 131.9, 129.5, 129.3, 129.0, 128.1, 127.7, 127.3, 127.1, 126.8, 126.3, 126.1, 125.8, 117.9, 63.6, 21.6, 21.6, 20.0, 16.9, 1.53; **HRMS (ESI)** Calcd for $C_{31}H_{35}NNaO_4SSi^+$ $[M+Na]^+$ 568.1948; Found: 568.1955.

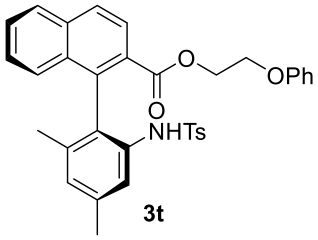

3t

2-Phenoxyethyl (*S*)-1-(2,4-dimethyl-6-((4-methylphenyl)sulfonamido)phenyl)-2-naphthoate (3t): 99% yield, 94% ee. $[\alpha]_D^{23}$ (c 0.45, $CHCl_3$) = + 63.3.

HPLC condition: Chiralpak AS-H (Hex/*i*PrOH = 80/20, 1.0 mL/min, t_R (major) = 25.3 min, t_R (minor) = 32.1 min).

^{1}H NMR (400 MHz, $CDCl_3$) δ 8.08 (d, *J* = 8.8 Hz, 1H), 7.97 (d, *J* = 8.8 Hz, 1H), 7.89 (d, *J* = 8.0 Hz, 1H), 7.53 (t, *J* = 7.6 Hz, 1H), 7.29-7.36 (m, 4H), 7.11 (t, *J* = 8.0 Hz, 1H), 6.97-7.01 (m, 3H), 6.89 (d, *J* = 8.4 Hz, 1H), 6.81 (d, *J* = 8.4 Hz, 2H), 6.65 (s, 1H), 6.03 (s, 1H), 4.41-4.47 (m, 1H), 4.17-4.22 (m, 1H), 3.73-3.79 (m, 1H), 3.60-3.65 (m, 1H), 2.34 (s, 3H), 2.26 (s, 3H),1.62 (s, 3H); **^{13}C NMR (100 MHz, $CDCl_3$)** δ 167.0, 158.4, 143.4, 138.2, 137.5, 136.6, 135.6, 135.3, 134.5, 131.9, 129.5, 129.4, 129.1, 128.5, 128.2, 128.0, 127.4, 127.1, 126.9, 126.3, 126.3, 126.2, 121.1, 117.6, 114.4, 65.0, 63.2, 21.6, 21.5, 20.0; **HRMS (ESI)** Calcd for $C_{34}H_{31}NNaO_5S^+$ $[M+H]^+$ 588.1815; Found: 588.1823.

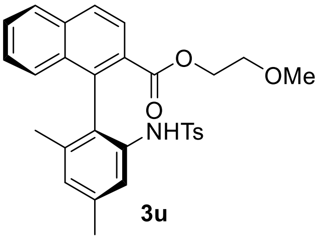

3u

2-Methoxyethyl (*S*)-1-(2,4-dimethyl-6-((4-methylphenyl)sulfonamido)phenyl)-2-naphthoate (3u): 99% yield, 93% ee. $[\alpha]_D^{23}$ (c 0.39, $CHCl_3$) = + 51.7.

HPLC condition: Chiralpak AS-H (Hex/*i*PrOH = 90/10, 1.0 mL/min, t_R (major) = 31.5 min, t_R (minor) = 47.7 min).

^{1}H NMR (400 MHz, $CDCl_3$) δ 8.03 (d, *J* = 8.4 Hz, 1H), 7.94 (d, *J* = 8.8 Hz, 1H), 7.87 (d, *J* = 8.4 Hz, 1H),7.48-7.53 (m, 2H), 7.30 (d, *J* = 8.0 Hz, 2H), 7.08 (d, *J* = 8.4 Hz, 1H), 6.96 (d, *J* = 8.0 Hz, 2H), 6.84-6.88 (m, 2H), 6.12 (s, 1H), 3.30-3.41 (m, 2H), 3.33 (s, 3H), 2.40 (s, 3H), 2.34 (s, 3H), 1.63(s, 3H); **^{13}C NMR (100 MHz, $CDCl_3$)** δ 167.0, 143.2, 138.2, 137.4, 136.7, 135.5, 135.1, 134.6, 131.9, 129.3, 129.0, 128.8, 128.1, 127.8, 127.3, 127.0, 126.6, 126.1, 126.0, 118.3, 70.0, 64.0, 58.9, 21.6, 21.6, 20.0; **HRMS (ESI)** Calcd for $C_{29}H_{29}NNaO_5S^+$ $[M+Na]^+$ 526.1659; Found: 526.1667.

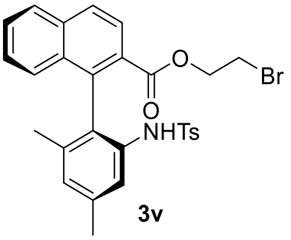

3v

2-Bromoethyl (*S*)-1-(2,4-dimethyl-6-((4-methylphenyl)sulfonamido)phenyl)-2-naphthoate (3v): 99% yield, 98% ee. $[\alpha]_D^{23}$ (c 0.42, $CHCl_3$) = + 94.4.

HPLC condition: Chiralpak AS-H (Hex/*i*PrOH = 80/20, 1.0 mL/min, t_R (major) = 20.9 min, t_R (minor) = 36.5 min).

^{1}H NMR (400 MHz, $CDCl_3$) δ 8.06 (d, *J* = 8.4 Hz, 1H), 7.97 (d, *J* = 8.8 Hz, 1H), 7.90 (d, *J* = 8.0 Hz, 1H),7.54 (t, *J* = 8.0 Hz, 1H), 7.47 (s, 1H), 7.37 (d, *J* = 8.0 Hz, 2H), 7.14 (t, *J* = 7.6 Hz, 1H), 7.04 (d, *J* = 7.6 Hz, 2H), 6.92 (d, *J* = 7.6 Hz, 1H), 6.87 (s, 1H), 6.00 (s, 1H), 4.29-4.36 (m, 1H), 4.15-4.21 (m, 1H),3.09-3.18 (m, 2H), 2.40 (s, 3H), 2.37 (s, 3H), 1.64 (s, 3H); **^{13}C NMR (100 MHz, $CDCl_3$)** δ 166.4, 143.5, 138.5, 137.4, 136.6, 135.7, 135.3, 134.6, 131.9, 129.4, 129.2, 128.2, 128.2, 128.1, 127.5, 127.2, 126.9, 126.2, 126.1, 126.0, 117.4, 64.3, 27.8, 21.6, 20.0; **HRMS (ESI)** Calcd for $C_{28}H_{26}BrNNaO_4S^+$ $[M+Na]^+$ 574.0658; Found: 574.0661.

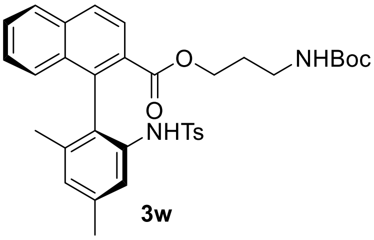

3w

3-((Tert-butoxycarbonyl)amino)propyl (*S*)-1-(2,4-dimethyl-6-((4-methylphenyl)sulfonamido)phenyl)-2-naphthoate (3w): 97% yield, 98% ee. $[\alpha]_D^{23}$ (c 0.46, $CHCl_3$) = + 97.2.

HPLC condition: Chiralpak AS-H (Hex/*i*PrOH = 90/10, 1.0 mL/min, t_R (major) = 29.9 min, t_R (minor) = 42.4 min).

^{1}H NMR (400 MHz, $CDCl_3$) δ 8.00 (d, *J* = 8.8 Hz, 1H),7.95 (d, *J* = 8.8 Hz, 1H), 7.88 (d, *J* = 8.0 Hz, 1H),7.52 (t, *J* = 7.2 Hz, 1H), 7.44 (s, 1H), 7.34 (d, *J* = 8.0 Hz, 2H), 7.09 (t, *J* = 7.2 Hz, 1H), 7.00 (d, *J* = 8.0 Hz, 2H), 6.82-6.86 (m, 2H), 6.08 (s, 1H), 4.64 (s, 1H), 4.08-4.14 (m, 1H), 3.98-4.04 (m, 1H), 2.89-3.00 (m, 2H), 2.38 (s, 3H), 2.35 (s, 3H), 1.63 (s, 3H), 1.54-1.60 (m, 2H), 1.44 (s, 9H); **^{13}C NMR (100 MHz, $CDCl_3$)** δ 167.0, 149.8, 149.6, 143.4, 138.4, 137.2, 137.2, 136.6, 136.4, 135.4, 135.3, 134.5, 131.8, 130.7, 129.4, 129.2, 128.6, 128.2, 128.0, 127.5, 127.1, 126.7, 126.1, 126.1, 125.8, 123.4, 117.4, 64.5, 21.6, 21.6, 19.9; **HRMS (ESI)** Calcd for $C_{34}H_{38}N_2NaO_6S^+$ $[M+Na]^+$ 625.2348; Found: 625.2349.

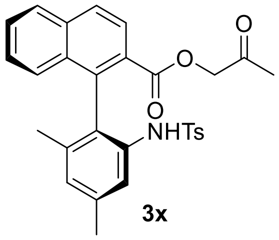

3x

2-Oxopropyl (*S*)-1-(2,4-dimethyl-6-((4-methylphenyl)sulfonamido)phenyl)-2-naphthoate (3x): 96% yield, 88% ee. $[\alpha]_D^{23}$ (c 0.39, $CHCl_3$) = + 103.8.

HPLC condition: Chiralpak AD (Hex/*i*PrOH = 80/20, 1.0 mL/min, t_R (major) = 46.4 min, t_R (minor) = 18.7 min).

^{1}H NMR (400 MHz, $CDCl_3$) δ 8.13 (d, *J* = 8.8 Hz, 1H),7.99 (d, *J* = 8.4 Hz, 1H), 7.91 (d, *J* = 8.4 Hz, 1H),7.56 (t, *J* = 8.0 Hz, 1H), 7.46 (s, 1H), 7.33 (d, *J* = 8.0 Hz, 2H), 7.14 (t, *J* = 7.2 Hz, 1H), 6.98 (d, *J* = 8.0 Hz, 2H), 6.90-6.92 (m, 2H), 6.21 (s, 1H), 4.66 (s, 2H), 2.40 (s, 3H), 2.36 (s, 3H), 2.10(s, 3H), 1.68 (s, 3H); **^{13}C NMR (100 MHz, $CDCl_3$)** δ 201.4, 166.0, 143.2, 138.4, 137.4, 136.7, 136.4, 135.3, 134.5, 131.9, 129.3, 129.0, 128.1, 128.0, 127.6, 127.4, 127.2, 127.0, 126.5, 126.3, 126.0, 118.7, 68.9, 26.0, 21.6, 21.6, 20.0, 1.06; **HRMS (ESI)** Calcd for $C_{29}H_{27}NNaO_5S^+$ $[M+H]^+$ 524.1502; Found: 524.1506.

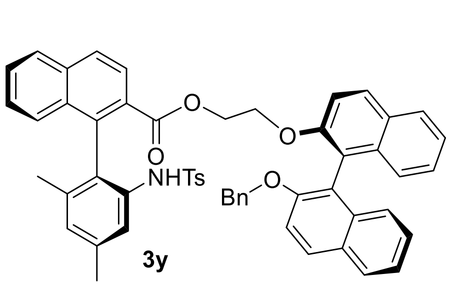

3y

2-(((S)-2'-(benzyloxy)-[1,1'-binaphthalen]-2-yl)oxy)ethyl (S)-1-(2,4-dimethyl-6-((4-methylphenyl)sulfonamido)phenyl)-2-naphthoate (3y): 92% yield, 94% ee. $[\alpha]_D^{23}$ (c 0.56, $CHCl_3$) = - 142.2.

HPLC condition: Chiralpak OD-H (Hex/*i*PrOH = 80/20, 1.0 mL/min, t_R (major) = 11.6 min, t_R (minor) = 17.1 min).

^{1}H NMR (400 MHz, $CDCl_3$) δ 8.01 (d, *J* = 9.2 Hz, 1H), 7.83-7.94 (m, 4H), 7.77 (d, *J* = 8.8 Hz, 1H), 7.52-7.56 (m, 2H), 7.43-7.46 (m, 2H), 7.31-7.40 (m, 5H), 7.19-7.25 (m, 4H), 7.10-7.16 (m, 4H), 6.97-6.99 (m, 2H), 6.93 (d, *J* = 8.0 Hz, 2H), 6.88 (d, *J* = 8.4 Hz, 1H), 6.75 (s, 1H), 5.98 (s, 1H), 5.04-5.12 (m, 2H), 4.00-4.06 (m, 1H), 3.82-3.95 (m, 3H), 2.34 (s, 3H), 2.25 (s, 3H), 1.44 (s, 3H); **^{13}C NMR (100 MHz, $CDCl_3$)** δ 166.5, 154.2, 154.0, 143.5, 138.3, 137.6, 137.5, 136.6, 135.6, 135.2, 134.6, 134.2, 134.2, 131.9, 129.8, 129.6, 129.5, 129.5, 129.5, 129.0, 128.3, 128.2, 128.1, 128.0, 128.0, 127.5, 127.4, 127.1, 127.0, 126.9, 126.5, 126.4, 126.2, 126.1, 125.8, 125.5, 124.0, 123.8, 121.0, 120.4, 117.8, 116.0, 115.9, 71.2, 67.1, 63.6, 21.7, 21.6, 19.9; **HRMS (ESI)** Calcd for $C_{55}H_{45}NNaO_6S^+$ $[M+Na]^+$ 870.2860; Found: 870.2864.

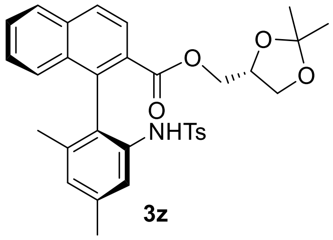

3z

((R)-2,2-dimethyl-1,3-dioxolan-4-yl)methyl (S)-1-(2,4-dimethyl-6-((4-methylphenyl)sulfonamido)phenyl)-2-naphthoate (3z): 96% yield, 97% ee. $[\alpha]_D^{23}$ (c 0.32, $CHCl_3$) = + 233.2.

HPLC condition: Chiralpak AD-H (Hex/*i*PrOH = 80/20, 1.0 mL/min, t_R (major) = 19.6 min, t_R (minor) = 14.4 min).

^{1}H NMR (400 MHz, $CDCl_3$) δ 8.05 (d, *J* = 8.4 Hz, 1H), 7.96 (d, *J* = 8.8 Hz, 1H), 7.88 (d, *J* = 8.0 Hz, 1H), 7.53 (t, *J* = 8.0 Hz, 1H), 7.48 (s, 1H), 7.33 (d, *J* = 8.4 Hz, 2H), 7.10 (t, *J* = 7.2 Hz, 1H), 7.00 (d, *J* = 8.0 Hz, 2H), 6.85-6.87 (m, 2H), 6.03 (s, 1H), 4.09-4.13 (m, 1H), 3.87-3.96 (m, 3H), 3.50-3.53 (m, 1H), 2.39 (s, 3H), 2.35 (s, 3H), 1.63 (s, 3H), 1.40 (s, 3H) , 1.33 (s, 3H); **^{13}C NMR (100 MHz, $CDCl_3$)** δ 166.8, 143.5, 138.5, 137.5, 136.6, 135.6, 135.3, 134.7, 131.9, 129.5, 129.2, 128.5, 128.3, 128.1, 127.5, 127.2, 127.0, 126.3, 126.2, 117.8, 109.7, 73.2, 66.7, 65.6, 27.0, 25.3, 21.7, 20.1; **HRMS (ESI)** Calcd for $C_{32}H_{34}NO_6S^+$ $[M+H]^+$ 560.2101; Found: 560.2120.

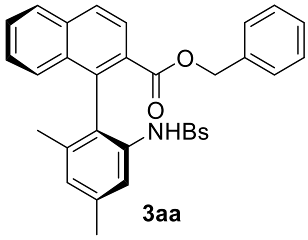

3aa

Benzyl (*S*)-1-(2,4-dimethyl-6-(phenylsulfonamido)phenyl)-2-naphthoate (3y): 98% yield, 96% ee. $[\alpha]_D^{23}$ (c 0.38, $CHCl_3$) = + 62.4.

HPLC condition: Chiralpak AZ-H (Hex/*i*PrOH = 80/20, 1.0 mL/min, t_R (major) = 27.3 min, t_R (minor) = 16.2 min).

^{1}H NMR (400 MHz, CDCl$_3$) δ 8.05 (d, *J* = 8.8 Hz, 1H),7.96 (d, *J* = 8.4 Hz, 1H), 7.88 (d, *J* = 8.4 Hz, 1H),7.41-7.54 (m, 5H), 7.31-7.33 (m, 3H), 7.21-7.25 (m, 3H), 7.11-7.15 (m, 3H), 6.87 (d, *J* = 8.4 Hz, 1H), 6.74 (s, 1H), 6.05 (s, 1H), 5.12 (d, *J* = 12.4 Hz, 1H), 4.94 (d, *J* = 12.4 Hz, 1H), 2.35 (s, 3H), 1.57 (s, 3H); **^{13}C NMR (100 MHz, CDCl$_3$)** δ 167.0, 139.6, 138.3, 137.3, 135.3, 135.2, 134.4, 132.7, 131.9, 129.1, 128.9, 128.8, 128.5, 128.4, 128.2, 128.2, 128.1, 127.6, 127.1, 127.0, 126.1, 126.1, 126.0, 117.6, 77.3, 67.2, 21.6, 19.9; **HRMS (ESI)** Calcd for $C_{32}H_{27}NNaO_4S^+$ $[M+Na]^+$ 544.1553; Found: 544.1561.

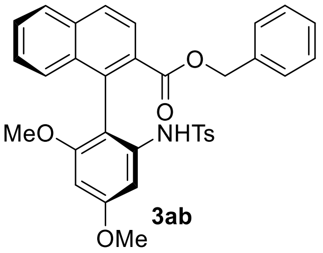

3ab

Benzyl (*R*)-1-(2,4-dimethoxy-6-((4-methylphenyl)sulfonamido)phenyl)-2-naphthoate (3z): 93% yield, 93% ee. $[\alpha]_D^{23}$ (c 0.41, CHCl$_3$) = + 59.6.

HPLC condition: Chiralpak AS-H (Hex/*i*PrOH = 80/20, 1.0 mL/min, t_R (major) = 40.1 min, t_R (minor) = 65.3 min).

^{1}H NMR (400 MHz, CDCl$_3$) δ 8.06 (d, *J* = 8.4 Hz, 1H),7.94 (d, *J* = 8.4 Hz, 1H), 7.87 (d, *J* = 8.4 Hz, 1H), 7.52 (t, *J* = 8.4 Hz, 1H), 7.38 (d, *J* = 8.4 Hz, 2H), 7.31 (t, *J* = 3.6 Hz, 3H), 7.13-7.16 (m, 3H), 6.97-7.04 (m, 4H), 6.10-6.13 (m, 2H), 5.14 (d, *J* = 12.4 Hz, 1H), 4.92 (d, *J* = 12.4 Hz, 1H), 3.85 (s, 3H), 3.32 (s, 3H), 2.35 (s, 3H); **^{13}C NMR (100 MHz, CDCl$_3$)** δ 167.1, 160.8, 158.1, 143.6, 136.4, 136.1, 135.4, 135.2, 132.6, 132.6, 129.6, 129.5, 129.0, 128.5, 128.4, 128.2, 128.1, 127.7, 127.3, 127.2, 126.4, 126.3, 110.5, 96.3, 94.9, 67.0, 55.6, 55.4, 21.6; **HRMS (ESI)** Calcd for $C_{33}H_{30}NO_6S^+$ $[M+H]^+$ 568.1788; Found: 568.1794.

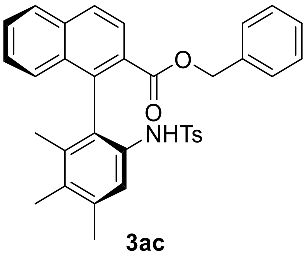

3ac

Benzyl (*S*)-1-(2,3,4-trimethyl-6-((4-methylphenyl)sulfonamido)phenyl)-2-naphthoate (3aa): 85% yield, 89% ee. $[\alpha]_D^{23}$ (c 0.43, CHCl$_3$) = + 112.3.

HPLC condition: Chiralpak AZ-H (Hex/*i*PrOH = 80/20, 1.0 mL/min, t_R (major) = 44.1 min, t_R (minor) = 25.3 min).

^{1}H NMR (400 MHz, CDCl$_3$) δ 8.04 (d, *J* = 8.8 Hz, 1H),7.94 (d, *J* = 8.4 Hz, 1H), 7.87 (d, *J* = 8.4 Hz, 1H), 7.51 (t, *J* = 8.0 Hz, 1H), 7.43 (s, 1H), 7.29-7.31 (m, 5H), 7.05-7.09 (m, 3H), 6.95 (d, *J* = 8.0 Hz, 2H), 6.83 (d, *J* = 8.4 Hz, 1H), 5.96 (s, 1H), 5.11 (d, *J* = 12.0 Hz, 1H), 4.49 (d, *J* = 12.0 Hz, 1H), 2.32 (s, 3H), 2.01 (s, 3H), 1.49 (s, 3H); **^{13}C NMR (100 MHz, CDCl$_3$)** δ 167.2, 143.1, 136.7, 136.7, 136.5, 135.7, 135.2, 135.1,

132.1, 131.8, 131.6, 129.3, 128.8, 128.8, 128.5, 128.3, 128.2, 128.0, 127.8, 127.3, 127.0, 126.5, 126.0, 119.2, 67.2, 21.6, 21.2, 17.4, 15.5; **HRMS (ESI)** Calcd for $C_{34}H_{31}NNaO_4S^+$ $[M+Na]^+$ 572.1866; Found: 572.1859.

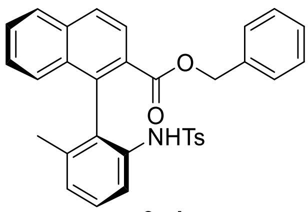

3ad

Benzyl (*S*)-1-(2-methyl-6-((4-methylphenyl)sulfonamido)phenyl)-2-naphthoate (3ab): 98% yield, 92% ee. $[\alpha]_D^{23}$ (c 0.41, $CHCl_3$) = + 99.6.

HPLC condition: Chiralpak AZ-H (Hex/*i*PrOH = 80/20, 1.0 mL/min, t_R (major) = 33.7 min, t_R (minor) = 23.2 min).

^{1}H NMR (400 MHz, $CDCl_3$) δ 8.07 (d, *J* = 8.4 Hz, 1H), 7.98 (d, *J* = 8.8 Hz, 1H), 7.90 (d, *J* = 8.0 Hz, 1H), 7.60 (d, *J* = 8.4 Hz, 1H), 7.54 (t, *J* = 7.2 Hz, 1H), 7.31-7.37 (m, 5H), 7.24 (t, *J* = 8.0 Hz, 1H), 7.09-7.15 (m, 3H), 7.00 (d, *J* = 8.0 Hz, 2H), 6.93 (d, *J* = 8.0 Hz, 1H), 6.84 (d, *J* = 8.4 Hz, 1H), 6.07 (s, 1H), 5.11 (d, *J* = 12.0 Hz, 1H), 4.94 (d, *J* = 12.0 Hz, 1H), 2.34 (s, 3H), 1.61 (s, 3H); **^{13}C NMR (100 MHz, $CDCl_3$)** δ 166.9, 143.4, 137.6, 136.6, 135.2, 134.8, 129.4, 129.2, 128.9, 128.7, 128.5, 128.4, 128.3, 128.2, 128.0, 127.5, 127.2, 126.1, 126.0, 125.9, 116.9, 67.2, 21.6, 20.0; **HRMS (ESI)** Calcd for $C_{32}H_{27}NNaO_4S^+$ $[M+Na]^+$ 544.1553; Found: 544.1554.

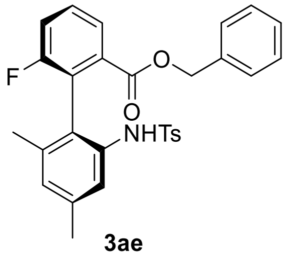

3ae

Benzyl (*R*)-6-fluoro-2',4'-dimethyl-6'-((4-methylphenyl)sulfonamido)-[1,1'-biphenyl]-2-carboxylate (3ac): 99% yield, 94% ee. $[\alpha]_D^{23}$ (c 0.39, $CHCl_3$) = + 64.6.

HPLC condition: Chiralpak AZ-H (Hex/*i*PrOH = 90/10, 1.0 mL/min, t_R (major) = 42.4 min, t_R (minor) = 37.7 min).

^{1}H NMR (400 MHz, $CDCl_3$) δ 7.82 (d, *J* = 8.0 Hz, 1H),7.53 (d, *J* = 8.4 Hz, 2H), 7.42-7.48 (m, 1H), 7.27-7.31 (m, 3H), 7.22 (s, 1H), 7.08-7.18 (m, 5H), 6.74 (s, 1H), 6.16 (s, 1H), 5.09 (d, *J* = 12.0 Hz, 1H), 4.93 (d, *J* = 12.0 Hz, 1H), 2.38 (s, 3H), 2.29 (s, 3H), 1.75 (s, 3H); **^{13}C NMR (100 MHz, $CDCl_3$)** δ 165.7 (d_{C-F}, *J* = 13 Hz), 161.0, 158.5, 143.5, 138.6, 137.0, 136.8, 134.9, 134.2, 133.0 (d_{C-F}, *J* = 9 Hz), 130.2, 130.1, 129.5, 128.4 (d_{C-F}, *J* = 8 Hz), 128.3, 127.5, 127.2, 126.6 (d_{C-F}, *J* = 14 Hz), 124.7, 124.5, 123.0, 119.9, 119.7, 67.4, 21.6, 21.5, 20.1; **HRMS (ESI)** Calcd for $C_{29}H_{26}FNNaO_4S^+$ $[M+Na]^+$ 526.1459; Found: 526.1467.

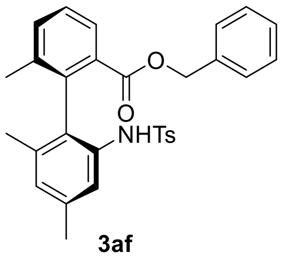

3af

Benzyl (*S*)-2',4',6-trimethyl-6'-((4-methylphenyl)sulfonamido)-[1,1'-biphenyl]-2-carboxylate (3ad): 86% yield, 95% ee. $[\alpha]_D^{23}$ (c 0.39, $CHCl_3$) = + 110.5.

HPLC condition: Chiralpak AD (Hex/*i*PrOH = 80/20, 1.0 mL/min, t_R (major) = 7.5 min, t_R (minor) = 9.1 min).

^{1}H NMR (400 MHz, $CDCl_3$) δ 7.86 (d, *J* = 7.2 Hz, 1H), 7.65 (d, *J* = 8.0 Hz, 2H), 7.37-7.44 (m, 2H), 7.20-7.29 (m, 6H), 7.06-7.08 (m, 2H), 6.66 (s, 1H), 6.04 (s, 1H), 5.02 (d, *J* = 12.0 Hz, 1H), 4.85 (d, *J* = 12.0 Hz, 1H), 2.37 (s, 3H), 2.28 (s, 3H), 1.67 (s, 3H), 1.63 (s, 3H); **^{13}C NMR (100 MHz, $CDCl_3$)** δ 167.0, 143.7, 138.8, 137.7, 137.2, 136.1, 135.6, 135.3, 134.5, 133.8, 131.6, 129.6, 128.6, 128.5, 128.4, 128.3, 128.1, 127.4, 127.0, 126.7, 116.7, 66.9, 21.6, 21.6, 19.9, 19.5; **HRMS (ESI)** Calcd for $C_{30}H_{29}NNaO_4S^+$ $[M+Na]^+$ 522.1710; Found: 522.1717.

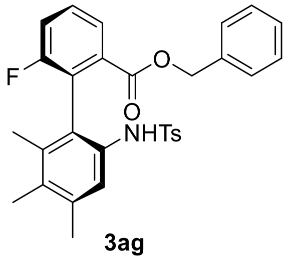

3ag

Benzyl (*R*)-6-fluoro-2',3',4'-trimethyl-6'-((4-methylphenyl)sulfonamido)-[1,1'-biphenyl]-2-carboxylate (3ae): 88% yield, 93% ee. $[\alpha]_D^{23}$ (c 0.40, $CHCl_3$) = + 69.1.

HPLC condition: Chiralpak AZ-H (Hex/*i*PrOH = 80/20, 1.0 mL/min, t_R (major) = 26.5 min, t_R (minor) = 21.2 min).

^{1}H NMR (400 MHz, $CDCl_3$) δ 7.81 (d, *J* = 8.0 Hz, 1H), 7.51 (d, *J* = 8.4 Hz, 2H), 7.41-7.46 (m, 1H), 7.25-7.31 (m, 3H), 7.22 (s, 1H), 7.11-7.16 (m, 3H), 7.03-7.07 (m, 2H), 6.06 (s, 1H), 5.09 (d, *J* = 12.0 Hz, 1H), 4.88 (d, *J* = 12.0 Hz, 1H), 2.38 (s, 3H), 2.25 (s, 3H), 2.01 (s, 3H), 1.68 (s, 3H); **^{13}C NMR (100 MHz, $CDCl_3$)**δ 165.9 ($d_{C\text{-}F}$, *J* = 13 Hz),161.0, 158.5, 143.3, 137.1, 137.1, 135.4, 134.5, 133.0, 132.4, 131.4, 129.9, 129.8, 129.4, 128.5, 128.4, 128.3, 127.2, 126.5, 126.5, 125.8, 125.6, 124.3, 121.4, 119.8, 119.6, 77.3, 67.3, 21.6, 21.1, 17.4, 15.7; **HRMS (ESI)** Calcd for $C_{30}H_{28}FNNaO_4S^+$ $[M+Na]^+$ 540.1615; Found: 540.1623.

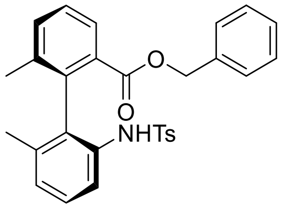

3ah

Benzyl (*S*)-2',6-dimethyl-6'-((4-methylphenyl)sulfonamido)-[1,1'-biphenyl]-2-carboxylate (3af): 99% yield, 93% ee. $[\alpha]_D^{23}$ (c 0.37, $CHCl_3$) = - 11.1.
HPLC condition: Chiralpak AD (Hex/*i*PrOH = 90/10, 1.0 mL/min, t_R (major) = 14.5min, t_R (minor) = 18.4 min).
^{1}H NMR (400 MHz, $CDCl_3$) δ 7.88 (d, *J* = 6.4 Hz, 1H), 7.67 (d, *J* = 8.4 Hz, 2H), 7.39-7.47 (m, 3H), 7.26-7.29 (m, 3H), 7.21 (d, *J* = 8.0 Hz, 1H), 7.07-7.14 (m, 3H), 6.86 (d, *J* = 7.6 Hz, 1H), 5.01 (d, *J* = 12.0 Hz, 1H), 4.84 (d, *J* = 12.0 Hz, 1H), 2.37 (s, 3H), 1.71 (s, 3H), 1.65 (s, 3H), 1.68 (s, 3H); **^{13}C NMR (100 MHz, $CDCl_3$)** δ 166.8, 143.7, 138.6, 137.2, 136.5, 135.4, 135.3, 134.6, 134.1, 131.4, 129.8, 129.6, 128.7, 128.7, 128.4, 128.3, 128.2, 128.0, 127.4, 125.7, 115.9, 66.9, 21.6, 20.0, 19.4; **HRMS (ESI)** Calcd for $C_{29}H_{27}NNaO_4S^+$ $[M+Na]^+$ 508.1553; Found: 508.1559.

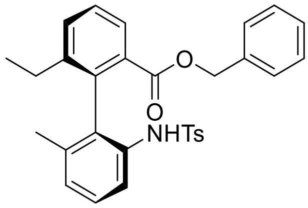

3ai

Benzyl (*S*)-6-ethyl-2'-methyl-6'-((4-methylphenyl)sulfonamido)-[1,1'-biphenyl]-2-carboxylate (3ag): 98% yield, 93% ee. $[\alpha]_D^{23}$ (c 0.38, $CHCl_3$) = + 78.0.
HPLC condition: Chiralpak AD (Hex/*i*PrOH = 90/10, 1.0 mL/min, t_R (major) = 12.6min, t_R (minor) = 19.3 min).
^{1}H NMR (400 MHz, $CDCl_3$) δ 7.88 (d, *J* = 6.0 Hz, 1H), 7.70 (d, *J* = 8.4 Hz, 2H), 7.46-7.55 (m, 2H), 7.37 (d, *J* = 8.0 Hz, 1H), 7.26-7.29 (m, 3H), 7.22 (d, *J* = 8.0 Hz, 2H), 7.06-7.13 (m, 3H), 6.83(d, *J* = 7.2 Hz, 1H), 6.08 (s, 1H), 4.98(d, *J* = 12.4 Hz, 1H), 4.89 (d, *J* = 12.4 Hz, 1H), 2.37 (s, 3H), 1.94-2.07 (m, 2H), 1.72 (s, 3H), 0.98 (t, *J* = 7.6 Hz, 3H), 1.68 (s, 3H); **^{13}C NMR (100 MHz, $CDCl_3$)** δ 166.9, 144.0, 143.8, 137.1, 136.9, 135.4, 135.3, 134.7, 134.5, 132.5, 131.6, 129.7, 129.0, 128.8, 128.6, 128.4, 128.2, 128.2, 128.1, 127.4, 125.4, 114.9, 66.8, 25.4, 21.6, 20.2, 14.0; **HRMS (ESI)** Calcd for $C_{30}H_{29}NNaO_4S^+$ $[M+Na]^+$ 522.1710; Found: 522.1717.

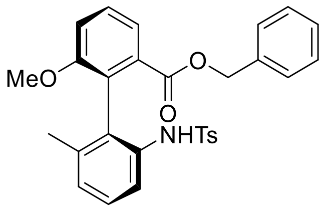

3aj

Benzyl (*R*)-6-methoxy-2'-methyl-6'-((4-methylphenyl)sulfonamido)-[1,1'-biphenyl]-2-carboxylate (3ah): 98% yield, 94% ee. $[\alpha]_D^{23}$ (c 0.38, $CHCl_3$) = + 19.5.
HPLC condition: Chiralpak AZ-H (Hex/*i*PrOH = 80/20, 1.0 mL/min, t_R (major) = 31.4 min, t_R (minor) = 25.3 min).
^{1}H NMR (400 MHz, $CDCl_3$) δ 7.60-7.63 (m, 3H),7.48 (t, *J* = 8.0 Hz, 1H), 7.41 (d, *J* = 8.4 Hz, 1H), 7.28-7.30 (m, 3H), 7.17 (d, *J* = 8.0 Hz, 2H), 7.06-7.13 (m, 4H), 6.85 (d, *J* = 7.6 Hz, 1H), 6.19 (s, 1H), 4.98 (d, *J* = 12.0 Hz, 1H), 4.82 (d, *J* = 12.0 Hz, 1H), 3.56 (s, 3H), 2.36 (s, 3H), 1.74 (s, 3H); **^{13}C NMR (100 MHz, $CDCl_3$)** δ 166.4, 157.0, 143.3, 137.4, 137.2, 135.2, 134.6, 132.7, 130.0, 129.4, 128.4, 128.3, 128.2, 128.0, 127.5, 127.4, 125.7, 124.8, 122.9, 116.6, 114.8, 67.0, 56.0, 21.6, 20.1; **HRMS (ESI)** Calcd for $C_{29}H_{27}NNaO_5S^+$ $[M+Na]^+$ 524.1502; Found: 524.1511.

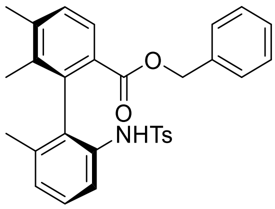

3ak

Benzyl (*S*)-2',5,6-trimethyl-6'-((4-methylphenyl)sulfonamido)-[1,1'-biphenyl]-2-carboxylate (3ai): 99% yield, 92% ee. $[\alpha]_D^{23}$ (c 0.39, $CHCl_3$) = +78.0.
HPLC condition: Chiralpak AD (Hex/*i*PrOH = 90/10, 1.0 mL/min, t_R (major) = 18.2 min, t_R (minor) = 24.1 min).
^{1}H NMR (400 MHz, $CDCl_3$) δ 7.82 (d, *J* = 8.0 Hz, 1H), 7.64(d, *J* = 8.4 Hz, 2H), 7.42 (d, *J* = 8.0 Hz, 1H), 7.28-7.30 (m, 4H), 7.20(d, *J* = 8.0 Hz, 2H), 7.07-7.14 (m, 3H), 6.85 (d, *J* = 7.6 Hz, 1H), 6.08 (s, 1H), 4.98 (d, *J* = 12.4 Hz, 1H), 4.80 (d, *J* = 12.4 Hz, 1H), 2.37 (s, 3H), 2.33 (s, 3H), 1.70 (s, 3H), 1.53 (s, 3H); **^{13}C NMR (100 MHz, $CDCl_3$)** δ 166.8, 143.6, 142.7, 137.2, 137.0, 136.7, 135.5, 135.4, 134.2, 130.5, 128.8, 128.6, 128.4, 128.2, 128.1, 127.9, 127.4, 125.6, 115.9, 66.7, 21.6, 21.1, 20.1, 15.5; **HRMS (ESI)** Calcd for $C_{30}H_{29}NNaO_4S^+$ $[M+Na]^+$ 522.1710; Found: 522.1715.

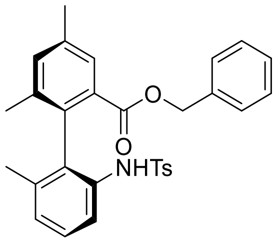

3al

Benzyl (*S*)-2',4,6-trimethyl-6'-((4-methylphenyl)sulfonamido)-[1,1'-biphenyl]-2-carboxylate (3aj): 99% yield, 92% ee. $[\alpha]_D^{23}$ (c 0.39, $CHCl_3$) = - 26.0.
HPLC condition: Chiralpak AD-H (Hex/*i*PrOH = 90/10, 1.0 mL/min, t_R (major) = 17.2 min, t_R (minor) = 15.7 min).
^{1}H NMR (400 MHz, $CDCl_3$) δ 7.66-7.69 (m, 3H), 7.38 (d, *J* = 8.4 Hz, 1H), 7.26-7.29 (m, 4H), 7.20 (d, *J* = 8.0 Hz, 2H), 7.04-7.12 (m, 3H), 6.83 (d, *J* = 7.6 Hz, 1H), 6.10 (s, 1H), 4.98 (d, *J* = 12.4 Hz, 1H), 4.80 (d, *J* = 12.4 Hz, 1H), 2.42 (s, 3H), 2.37 (s, 3H), 1.71 (s, 3H), 1.62 (s, 3H); **^{13}C NMR (100 MHz, $CDCl_3$)** δ 167.0, 143.7, 138.6, 138.3, 137.1, 136.8, 135.5, 135.4, 134.2, 132.3, 131.2, 129.7, 129.6, 129.3, 128.4, 128.3, 128.1, 127.9, 127.4, 125.5, 115.5, 66.8, 21.6, 21.1, 20.0, 19.4; **HRMS (ESI)** Calcd for $C_{30}H_{29}NNaO_4S^+$ $[M+Na]^+$ 522.1711; Found: 522.1713.

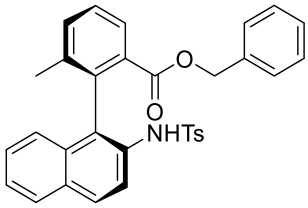

3am

Benzyl (*S*)-3-methyl-2-(2-((4-methylphenyl)sulfonamido)naphthalen-1-yl)benzoate (3ak): 99% yield, 91% ee. $[\alpha]_D^{23}$ (c 0.41, $CHCl_3$) = + 118.5.
HPLC condition: Chiralpak AD (Hex/*i*PrOH = 90/10, 1.0 mL/min, t_R (major) = 20.0 min, t_R (minor) = 28.3 min).
^{1}H NMR (400 MHz, $CDCl_3$) δ 7.98-8.01 (m, 1H), 7.82 (d, *J* = 8.8 Hz, 1H), 7.75 (t, *J* = 8.8 Hz, 2H), 7.69 (d, *J* = 8.4 Hz, 2H), 7.49-7.53 (m, 2H), 7.34 (t, *J* = 8.0 Hz, 1H), 7.20-7.24 (m, 4H), 7.12-7.16 (m, 2H), 6.91 (d, *J* = 8.4 Hz, 1H), 6.79 (d, *J* = 7.6 Hz, 2H), 6.42 (s, 1H), 4.85 (d, *J* = 12.4 Hz, 1H), 4.65 (d, *J* = 12.4 Hz, 1H), 2.37 (s, 3H), 1.51 (s, 3H); **^{13}C NMR (100 MHz, $CDCl_3$)** δ 166.7, 143.9, 139.6, 137.4, 135.0, 134.7, 134.3, 132.3, 132.2, 131.7, 130.6, 129.7, 129.0, 128.9, 128.9, 128.3, 128.2, 128.0, 127.9, 127.3, 126.8, 125.7, 124.9, 124.7, 118.5, 66.8, 21.6, 19.5; **HRMS (ESI)** Calcd for $C_{32}H_{27}NNaO_4S^+$ $[M+Na]^+$ 544.1553; Found: 544.1561.

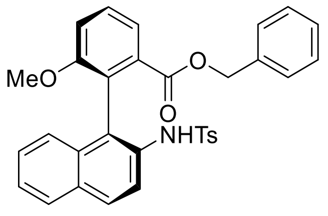

3an

Benzyl (*R*)-3-methoxy-2-(2-((4-methylphenyl)sulfonamido)naphthalen-1-yl)benzoate (3al): 96% yield, 93% ee. $[\alpha]_D^{23}$ (c 0.42, $CHCl_3$) = + 102.9.
HPLC condition: Chiralpak AD (Hex/*i*PrOH = 90/10, 1.0 mL/min, t_R (major) = 37.9 min, t_R (minor) = 53.6 min).
^{1}H NMR (400 MHz, $CDCl_3$) δ 7.82 (d, *J* = 8.8 Hz, 1H), 7.69-7.73 (m, 3H), 7.54-7.60 (m, 3H), 7.31 (t, *J* = 8.4 Hz, 1H), 7.11-7.23 (m, 7H), 6.98 (d, *J* = 8.4 Hz, 1H), 6.74 (d, *J* = 6.8 Hz, 2H), 6.55 (s, 1H), 4.72 (d, *J* = 12.4 Hz, 1H), 4.60 (d, *J* = 12.4 Hz, 1H), 3.47 (s, 3H), 2.34 (s, 3H); **^{13}C NMR (100 MHz, $CDCl_3$)** δ 166.2, 157.7, 143.3, 137.4, 134.9, 133.6, 132.9, 132.1, 130.7, 130.3, 129.5, 128.8, 128.3, 128.1,128.0, 128.0, 127.3, 126.5, 124.8, 124.7, 123.8, 123.7, 123.2, 119.5, 115.0, 77.3, 66.8, 56.1, 21.5; **HRMS (ESI)** Calcd for $C_{32}H_{27}NNaO_5S^+$ $[M+Na]^+$ 560.1502; Found: 560.1511.

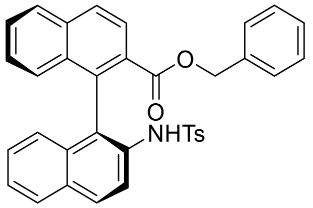

3ao

Benzyl (*S*)-2'-((4-methylphenyl)sulfonamido)-[1,1'-binaphthalene]-2-carboxylate (3ao): 98% yield, 79% ee. $[\alpha]_D^{23}$ (c 0.40, $CHCl_3$) = +91.2.
HPLC condition: Chiralpak AZ-H (Hex/*i*PrOH = 85/15, 1.0 mL/min, t_R (major) = 65.1 min, t_R (minor) = 54.1 min).
^{1}H NMR (400 MHz, $CDCl_3$) δ 8.18 (d, *J* = 8.4 Hz, 1H), 8.07 (d, *J* = 8.8 Hz, 1H), 8.00 (d, *J* = 8.8 Hz, 1H), 7.93 (d, *J* = 8.0 Hz, 1H), 7.84 (d, *J* = 8.8 Hz, 1H), 7.80 (d, *J* = 8.0 Hz, 1H), 7.52 (t, *J* = 8.0 Hz, 1H), 7.37(d, *J* = 8.4 Hz, 2H), 7.33 (d, *J* = 7.2 Hz, 1H), 7.09-7.26 (m, 4H), 6.95-6.98 (m, 3H), 6.84 (d, *J* = 7.2 Hz, 2H), 6.73 (d, *J* = 8.8 Hz, 1H), 6.67 (d, *J* = 8.4 Hz, 1H), 6.39 (s, 1H), 4.94 (d, *J* = 12.0 Hz, 1H), 4.77 (d, *J* = 12.0 Hz, 1H), 2.33 (s, 3H); **^{13}C NMR (100 MHz, $CDCl_3$)** δ 166.7, 143.5, 136.7, 135.2, 134.9, 134.1, 133.1, 132.5, 132.4, 130.6, 129.7, 129.6, 129.5, 129.3, 128.4, 128.1, 128.1, 128.1, 128.0, 128.0, 127.4, 127.0, 126.8, 126.7, 126.3, 125.5, 125.0, 124.7, 119.2, 67.1, 21.6; **HRMS (ESI)** Calcd for $C_{35}H_{27}NNaO_4S^+$ $[M+Na]^+$ 580.1553; Found: 580.1557.

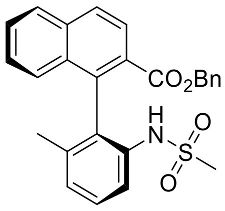

3ap

Benzyl (*S*)-1-(2-methyl-6-(methylsulfonamido)phenyl)-2-naphthoate (3ap): 92% yield, 90% ee. $[\alpha]_D^{23}$ (c 0.28, $CHCl_3$) = -316.2.

HPLC condition: Chiralpak AD-H (Hex/*i*PrOH = 80/20, 1.0 mL/min, t_R (major) = 11.9 min, t_R (minor) = 9.4 min).

^{1}H NMR (400 MHz, CDCl$_3$) δ 8.04 (q, *J* = 24.4 Hz, 8.4Hz, 2H), 7.95 (d, *J* = 8.0 Hz, 1H), 7.59-7.63 (m, 2H), 7.43 (t, *J* = 8.4 Hz, 1H), 7.28-7.38 (m, 5H), 7.17-7.20 (m, 2H), 7.09 (d, *J* = 7.6 Hz, 1H), 5.71(s, 1H), 5.13 (q, *J* = 16.8 Hz, 12.0Hz, 2H), 2.58 (s, 3H), 1.75 (s, 3H); **^{13}C NMR (100 MHz, CDCl$_3$)** δ 167.1, 138.0, 135.5, 135.4, 135.3, 135.1, 131.7, 129.5, 129.0, 128.9, 128.8, 128.7, 128.7, 128.6, 128.0, 126.2, 126.2, 116.5, 67.5, 39.4, 20.2; **HRMS (ESI)** Calcd for $C_{26}H_{23}NNaO_4S^+$ $[M+Na]^+$ 468.1240; Found: 468.1245.

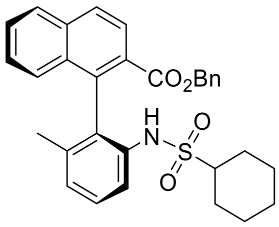

3aq

Benzyl (*S*)-1-(2-(cyclohexanesulfonamido)-6-methylphenyl)-2-naphthoate (3aq): 93% yield, 91% ee. $[\alpha]_D^{23}$ (c 0.32, $CHCl_3$) = -263.1.

HPLC condition: Chiralpak AD-H (Hex/*i*PrOH = 80/20, 1.0 mL/min, t_R (major) = 30.6 min, t_R (minor) = 17.5 min).

^{1}H NMR (400 MHz, CDCl$_3$) δ 8.03 (q, *J* = 32.4 Hz, 8.8Hz, 2H), 7.95 (d, *J* = 8.0 Hz, 1H), 7.58-7.65 (m, 2H), 7.44 (t, *J* = 8.4 Hz, 1H), 7.30-7.36 (m, 5H), 7.19-7.21 (m, 2H), 7.05 (d, *J* = 7.6 Hz, 1H), 5.68 (s, 1H), 5.15 (q, *J* = 24.4 Hz, 12.4Hz, 2H), 2.61-2.69 (m, 1H), 1.72 (s, 3H), 1.64-1.68 (m, 1H), 1.47-1.50 (m, 2H), 1.33-1.36 (m, 1H), 1.13-1.27 (m, 2H), 0.89-1.01 (m, 2H), 0.61-0.73 (m, 1H); **^{13}C NMR (100 MHz, CDCl$_3$)** δ 167.1, 137.7, 135.9, 135.7, 135.5, 135.3, 131.8, 129.3, 129.2, 128.9, 128.8, 128.7, 128.6, 128.6, 128.5, 127.9, 126.3, 126.2, 126.1, 118.1, 67.4, 60.9, 26.3, 26.2, 25.0, 24.9, 24.8, 20.2; **HRMS (ESI)** Calcd for $C_{31}H_{31}NNaO_4S^+$ $[M+Na]^+$ 536.1866; Found: 536.1867.

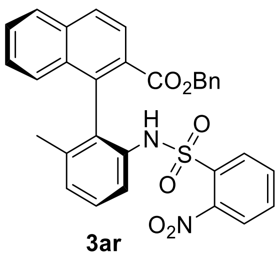

3ar

Benzyl (*S*)-1-(2-methyl-6-((2-nitrophenyl)sulfonamido)phenyl)-2-naphthoate (3ar): 96% yield, 98% ee. $[\alpha]_D^{23}$ (c 0.40, $CHCl_3$) = -250.0.
HPLC condition: Chiralpak AD (Hex/*i*PrOH = 80/20, 1.0 mL/min, t_R (major) = 46.9 min, t_R (minor) = 17.1 min).
^{1}H NMR (400 MHz, $CDCl_3$) δ 7.99 (d, *J* = 8.8Hz, 1H), 7.85 (d, *J* = 8.8 Hz, 1H), 7.28-7.42 (m, 8H), 7.18-7.20 (m, 2H), 7.09 (d, *J* = 7.6Hz, 1H), 6.95 (t, *J* = 8.0Hz, 1H), 6.75 (d, *J* = 7.6Hz, 1H), 7.30-7.36 (m, 5H), 7.19-7.21 (m, 2H), 7.05 (d, *J* = 7.6 Hz, 1H), 5.13 (d, *J* = 12.4Hz, 1H), 5.01 (d, *J* = 12.4Hz, 1H), 1.62 (s, 3H); **^{13}C NMR (100 MHz, $CDCl_3$)** δ 166.9, 146.5, 137.6, 135.7, 135.3, 135.1, 134.7, 134.1, 133.1, 132.7, 132.1, 131.7, 130.5, 129.1, 128.7, 128.6, 128.5, 128.4, 128.2, 127.7, 127.6, 127.2, 126.1, 125.8, 125.4, 121.9, 67.3, 20.1; **HRMS (ESI)** Calcd for $C_{31}H_{24}N_2NaO_6S^+$ $[M+Na]^+$ 575.1247; Found: 575.1253.

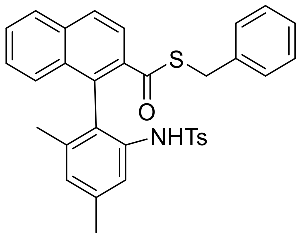

S-benzyl 1-(2,4-dimethyl-6-((4-methylphenyl)sulfonamido)phenyl)naphthalene-2-carbothioate
^{1}H NMR (400 MHz, $CDCl_3$) δ 7.94 (d, *J* = 8.8 Hz, 1H), 7.86 (d, *J* = 8.0 Hz, 1H), 7.81 (d, *J* = 8.4 Hz, 1H), 7.50 (t, *J* = 8.0 Hz, 1H), 7.45 (s, 1H), 7.33 (d, *J* = 8.0 Hz, 2H), 7.22-7.27 (m, 3H), 7.08 – 7.15 (m, 3H), 6.92 (d, *J* = 8.0 Hz, 2H), 6.84 – 6.86 (m, 2H), 6.34 (s, 1H), 4.12 (s, 2H), 2.39 (s, 3H), 2.30 (s, 3H), 1.63 (s, 3H); **^{13}C NMR (100 MHz, $CDCl_3$)** δ 193.6, 143.3, 138.9, 138.0, 137.3, 136.9, 136.2, 135.4, 134.8, 132.3, 132.0, 129.4, 129.3, 128.8, 128.7, 128.2, 127.7, 127.7, 127.4, 127.3, 127.1, 126.4, 125.4, 124.0, 118.9, 34.1, 21.7, 21.7, 20.2; **HRMS (ESI)** Calcd for $C_{33}H_{30}NO_3S_2^+$ $[M+H]^+$ 552.1662; Found: 552.1661.

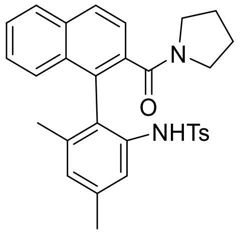

N-(3,5-dimethyl-2-(2-(pyrrolidine-1-carbonyl)naphthalen-1-yl)phenyl)-4 methylbenzenesulfonamide

^{1}H NMR (400 MHz, CDCl$_3$) δ 8.87 (bs, 1H), 7.84 (d, J = 8.4 Hz, 1H), 7.75 (d, J = 8.0 Hz, 1H), 7.44 (s, 1H), 7.37-7.39 (m, 2H), 6.99-7.05 (m, 3H), 6.90 (s, 1H), 6.66 – 6.63 (d, J = 8.4 Hz, 1H), 6.56 (d, J = 7.9 Hz, 2H), 3.49 – 3.55 (m, 1H), 3.29 – 3.43 (m, 2H), 2.98 – 3.03 (m, 1H), 2.36 (s, 3H), 2.14 (s, 3H), 1.79-1.90 (m, 3H), 1.65-1.71 (m, 1H), 1.66 (s, 3H); **^{13}C NMR (100 MHz, CDCl$_3$)** δ 170.0, 141.9, 138.7, 137.9, 137.7, 135.4, 134.4, 133.2, 132.4, 131.7, 128.8, 128.4, 127.9, 127.8, 127.0, 126.2, 126.1, 123.0, 48.7, 45.6, 26.1, 24.3, 21.5, 21.4, 20.4; **HRMS (ESI)** Calcd for $C_{30}H_{31}N_2O_3S^+$ $[M+H]^+$ 499.2050; Found: 499.2064.

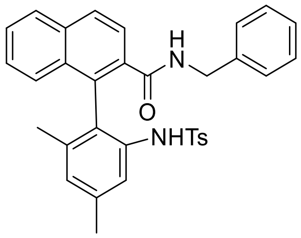

N-benzyl-1-(2,4-dimethyl-6-((4-methylphenyl)sulfonamido)phenyl)-2-naphthamide

^{1}H NMR (400 MHz, CDCl$_3$) δ 7.92 (d, *J* = 8.4 Hz, 1H), 7.86 (d, *J* = 8.2 Hz, 1H), 7.82 (d, *J* = 8.5 Hz, 1H), 7.49 (t, *J* = 8.0 Hz, 1H), 7.28 – 7.31 (m, 3H), 7.13 – 7.23 (m, 4H), 6.95 (d, *J* = 8.0 Hz, 2H), 6.84-6.88 (m, 3H), 6.79 (s, 1H), 6.70 (s, 1H), 6.13 (t, *J* = 5.2 Hz, 1H), 4.38 (dd, *J* = 14.8, 6.0 Hz, 1H), 4.22 (dd, *J* = 14.8, 6.0 Hz, 1H), 2.32 (s, 3H), 2.30 (s, 3H), 1.64 (s, 3H); **^{13}C NMR (100 MHz, CDCl$_3$)** δ 168.8, 143.5, 139.2, 138.1, 137.6, 136.8, 135.0, 134.3, 134.0, 131.6, 131.3, 129.4, 129.3, 128.5, 128.4, 128.0, 127.7, 127.5, 127.3, 127.2, 127.1, 126.4, 125.7, 125.3, 120.1, 44.0, 21.6, 21.6, 20.1; **HRMS (ESI)** Calcd for $C_{33}H_{31}N_2O_3S^+$ $[M+H]^+$ 535.2050; Found: 535.2054.

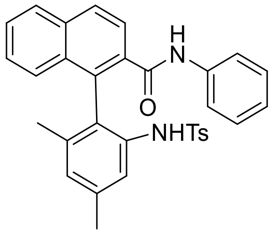

1-(2,4-dimethyl-6-((4-methylphenyl)sulfonamido)phenyl)-N-phenyl-2-naphthamide

^{1}H NMR (400 MHz, CDCl$_3$) δ 8.16 (d, *J* = 8.8 Hz, 1H), 8.04 (d, *J* = 8.8 Hz, 1H), 7.95 (d, *J* = 8.0 Hz, 1H), 7.59 (t, *J* = 8.0 Hz, 1H), 7.47 (s, 1H), 7.39 (d, *J* = 8.4 Hz, 3H), 7.30-7.34 (m, 1H), 7.19 – 7.23 (m, 2H), 7.03 – 7.10 (m, 4H), 6.96 (s, 1H), 6.93 (d, *J* = 8.0 Hz, 2H), 6.27 (s, 1H), 2.40 (s, 3H), 2.20 (s, 3H), 1.77 (s, 3H); **^{13}C NMR (100 MHz, CDCl$_3$)** δ 165.5, 144.1, 140.0, 138.7, 137.7, 136.0, 135.3, 135.0, 133.2, 131.4, 131.2, 129.7, 128.8, 128.7, 128.0, 127.9, 127.1, 126.4, 125.7, 124.9, 124.4, 120.0, 118.7, 21.7, 21.6, 20.2; **HRMS (ESI)** Calcd for $C_{32}H_{29}N_2O_3S^+$ $[M+H]^+$ 521.1893; Found: 521.1906.

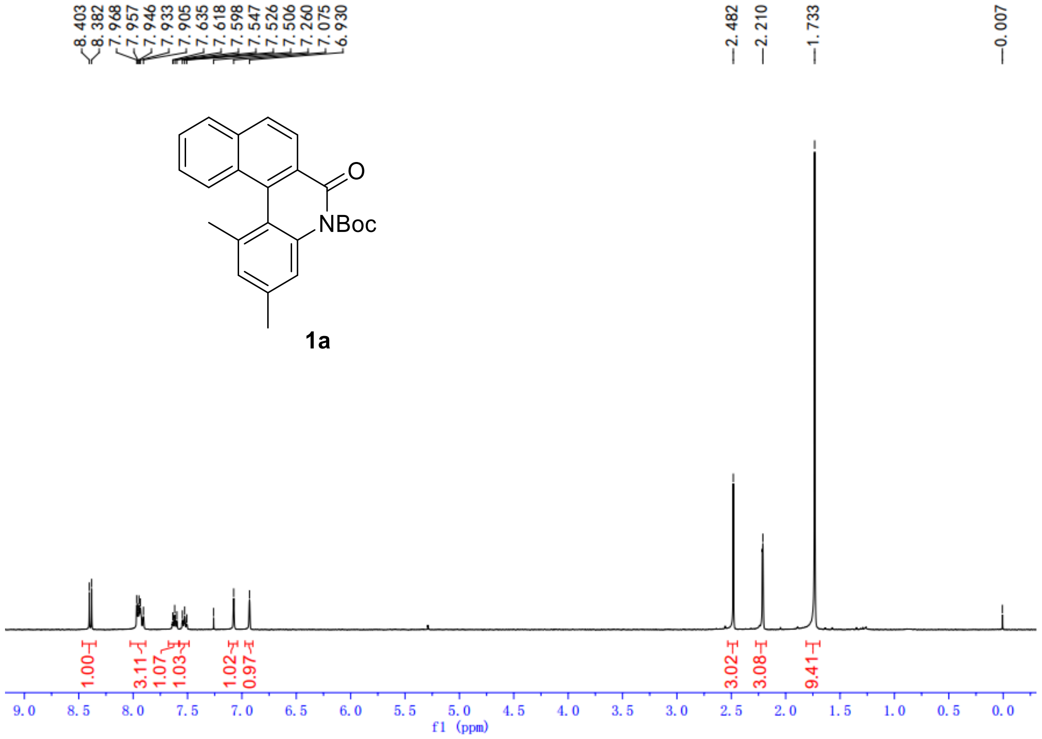

Supplementary Figure 4. ^{1}H NMR Spectrum of **1a**

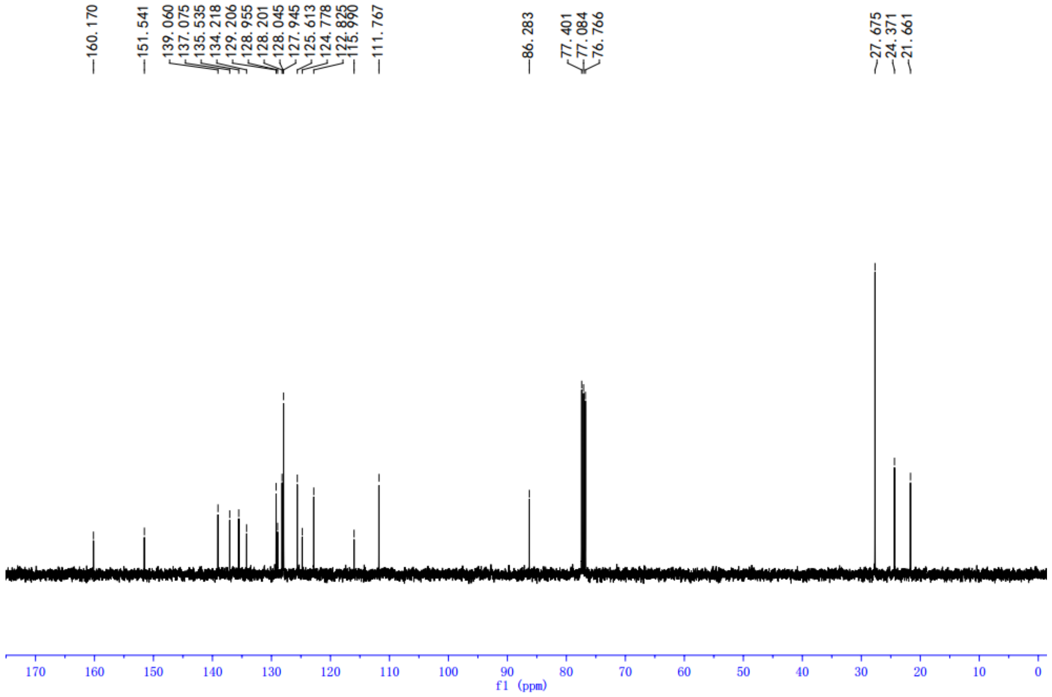

Supplementary Figure 5. ^{13}C NMR Spectrum of **1a**

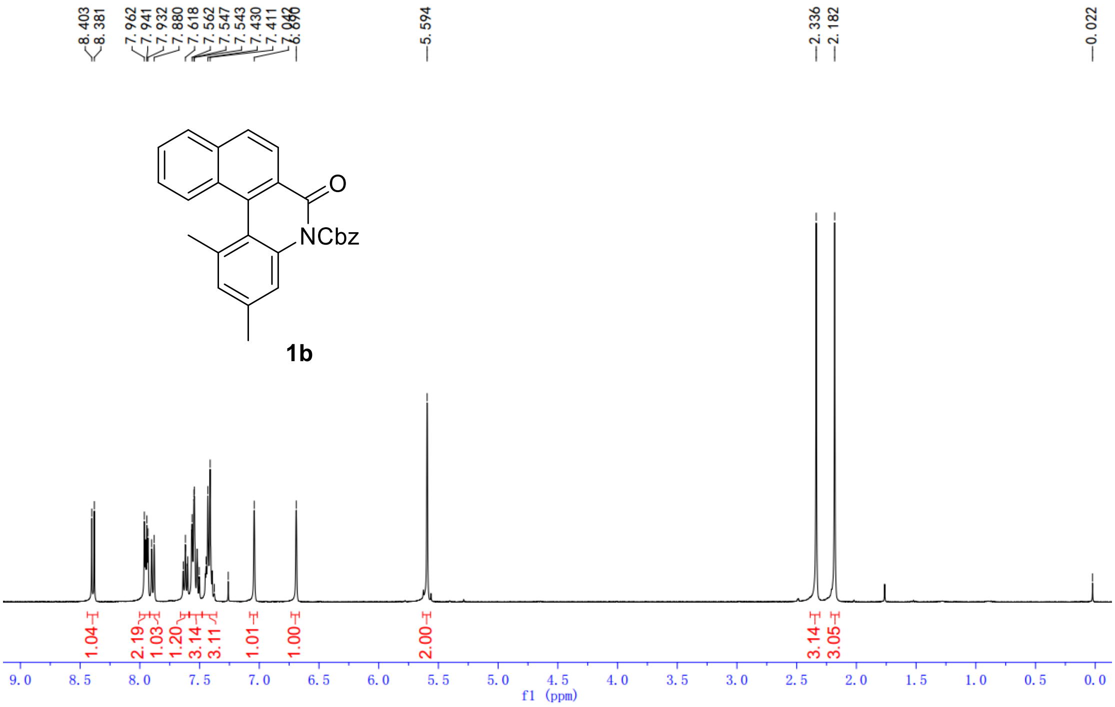

Supplementary Figure 6. ^{1}H NMR Spectrum of **1b**

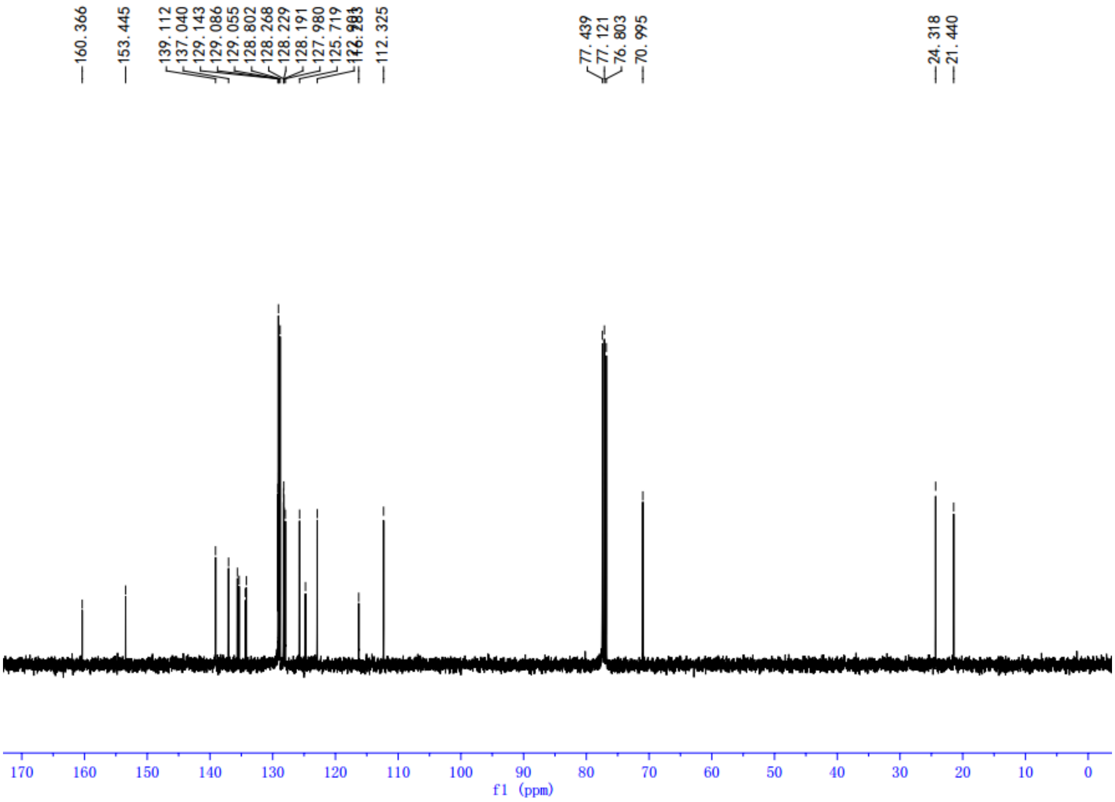

Supplementary Figure 7. ^{13}C NMR Spectrum of **1b**

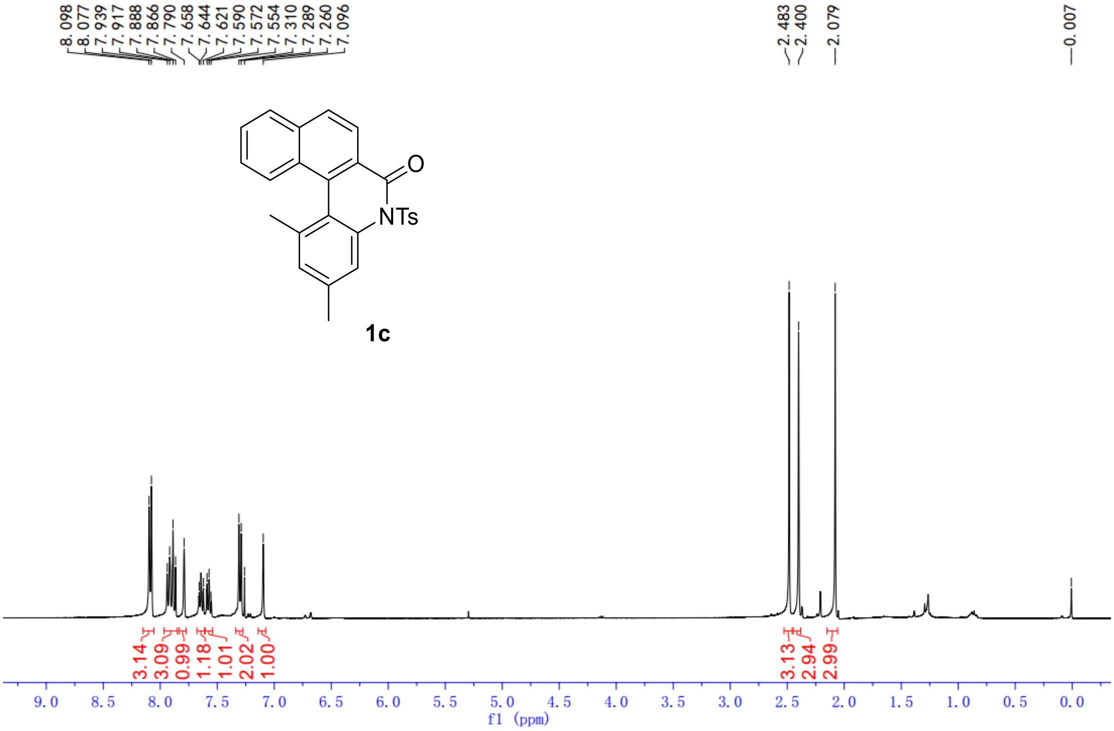

Supplementary Figure 8. ^{1}H NMR Spectrum of **1c**

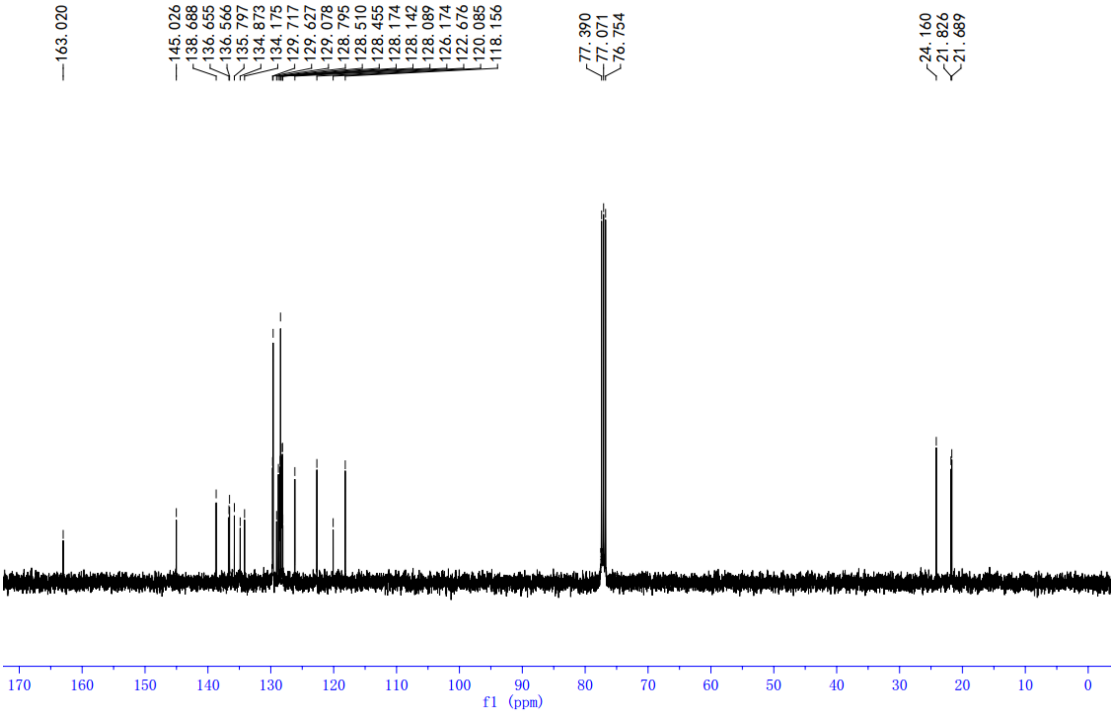

Supplementary Figure 9. ^{13}C NMR Spectrum of **1c**

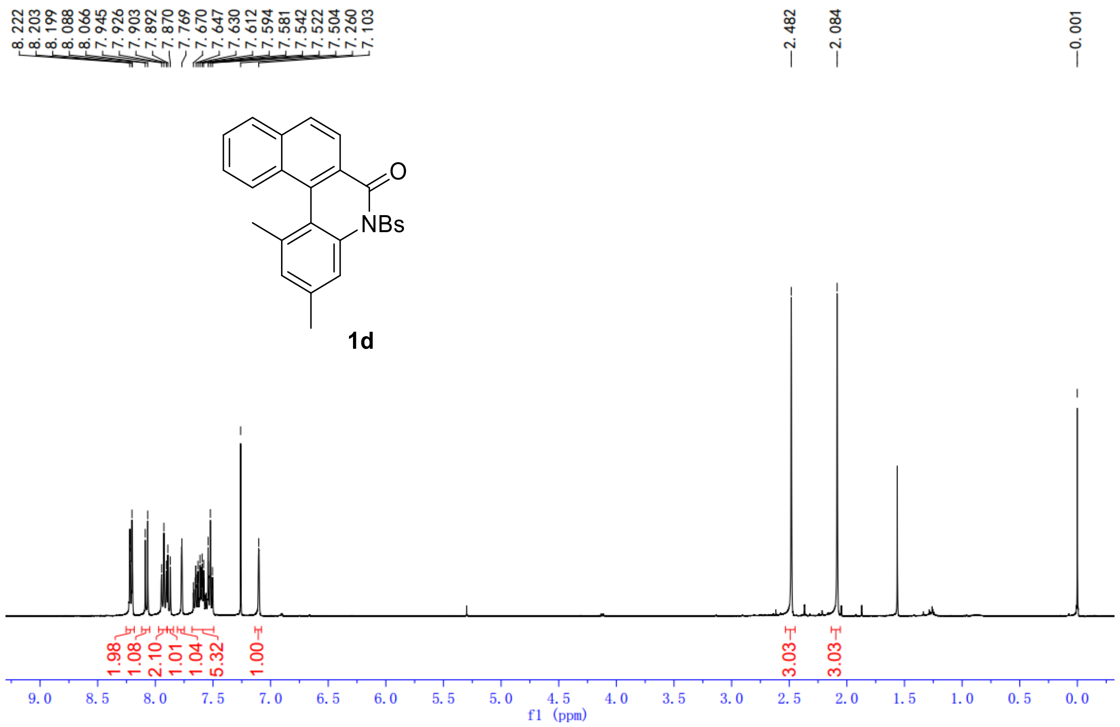

Supplementary Figure 10. ^{1}H NMR Spectrum of **1d**

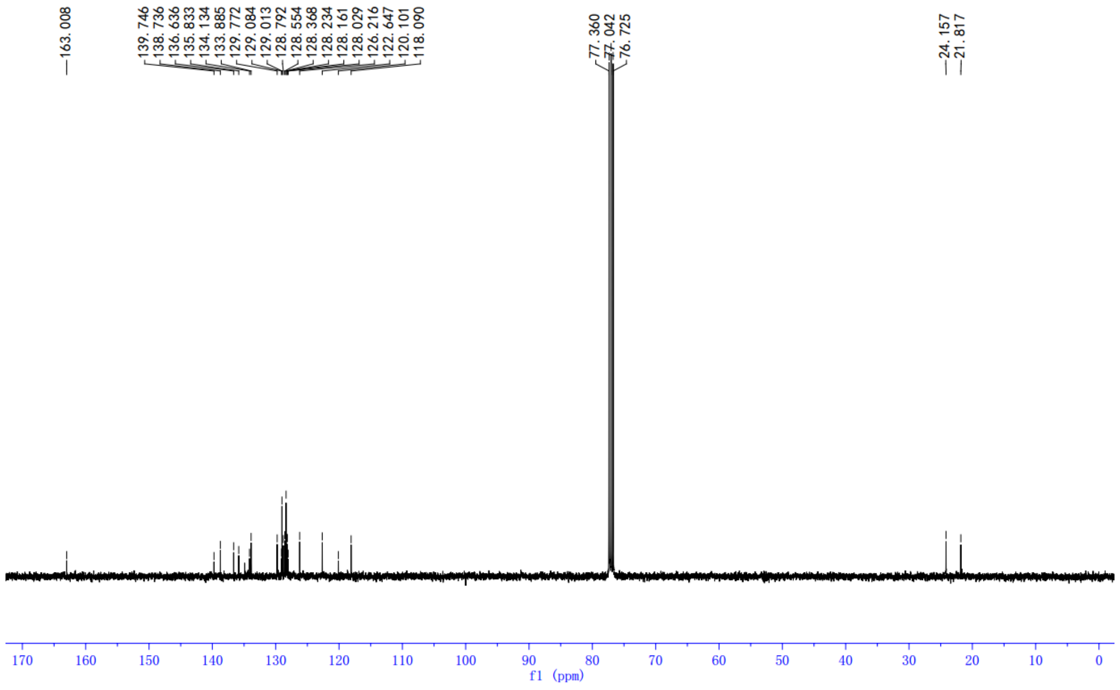

Supplementary Figure 11. ^{13}C NMR Spectrum of **1d**

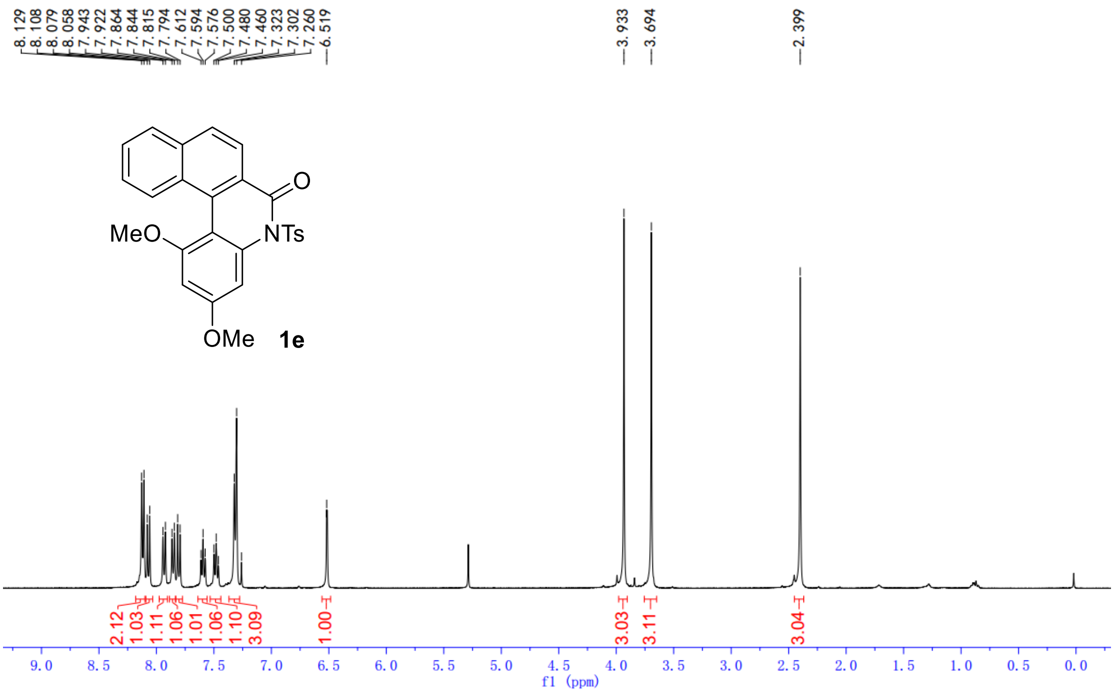

Supplementary Figure 12. ^{1}H NMR Spectrum of **1e**

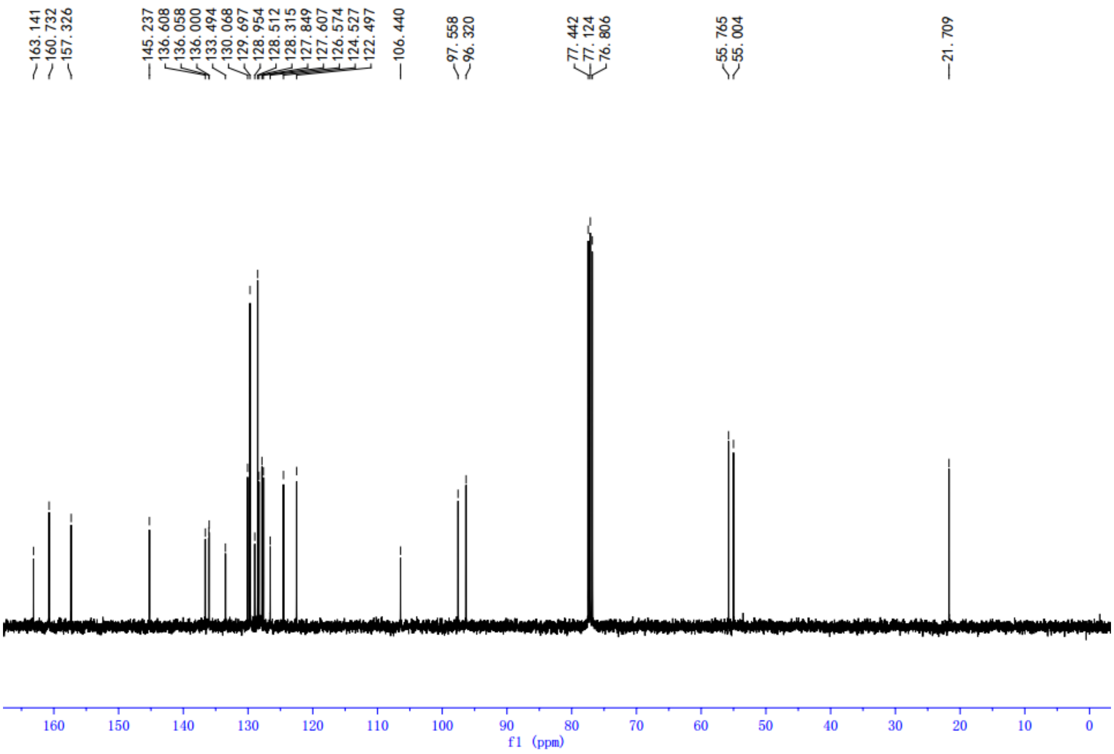

Supplementary Figure 13. ^{13}C NMR Spectrum of **1e**

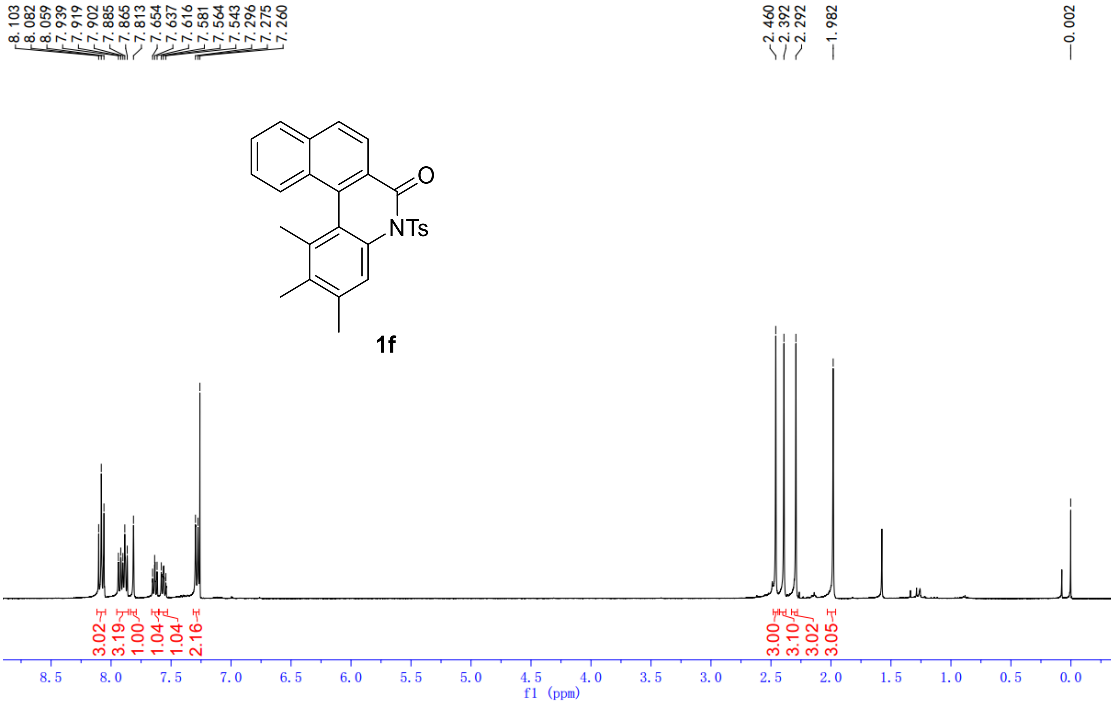

Supplementary Figure 14. ^{1}H NMR Spectrum of **1f**

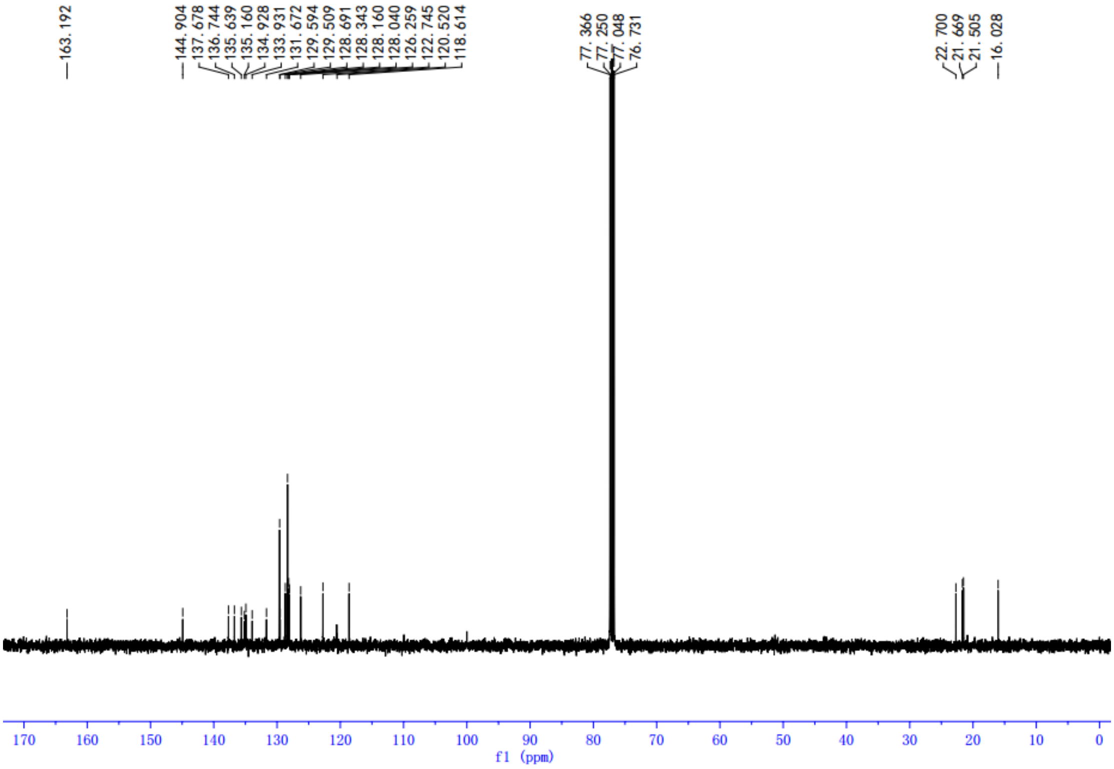

Supplementary Figure 15. ^{13}C NMR Spectrum of **1f**

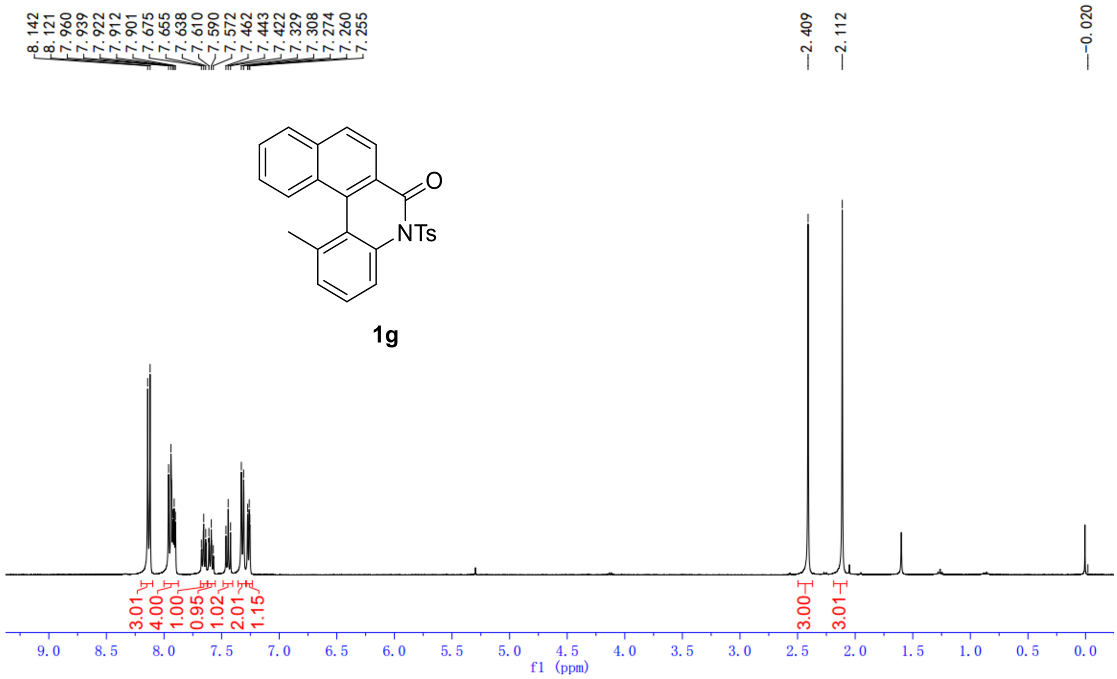

Supplementary Figure 16. ^{1}H NMR Spectrum of **1g**

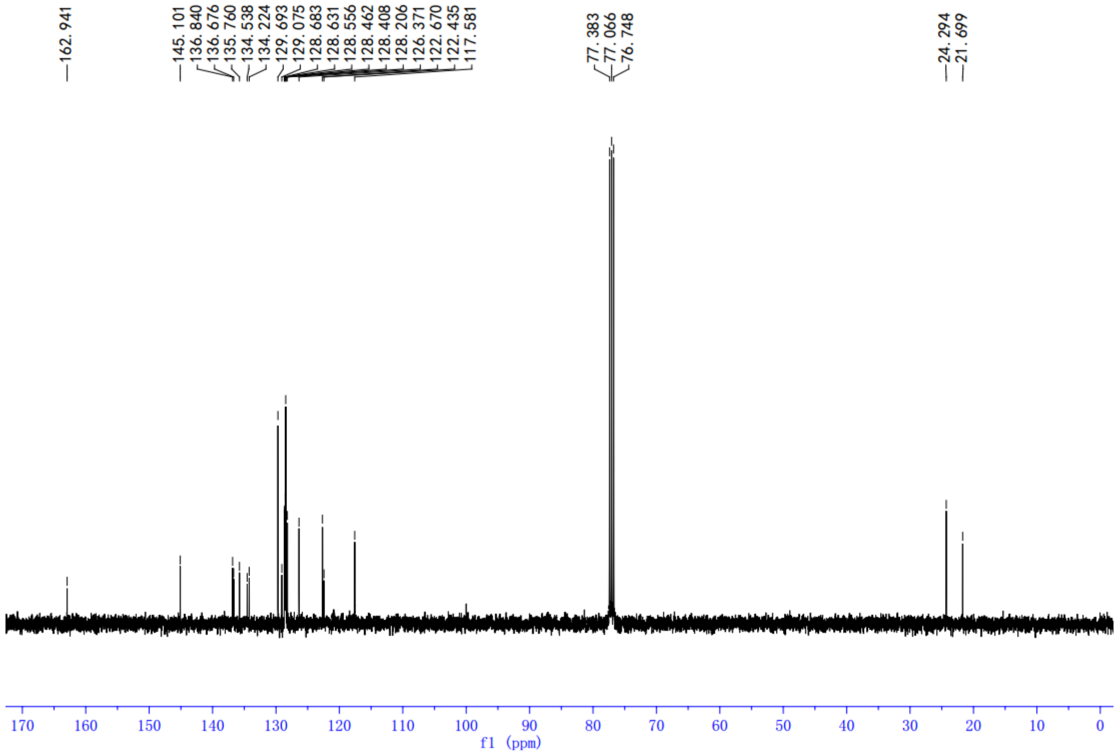

Supplementary Figure 17. ^{13}C NMR Spectrum of **1g**

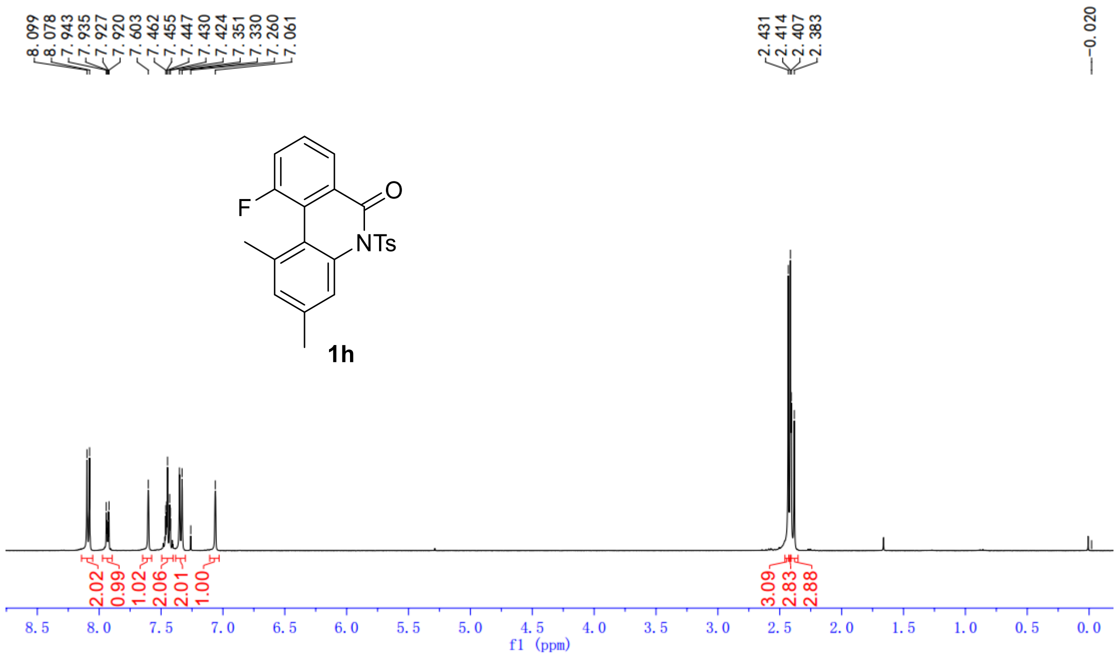

Supplementary Figure 18. ^{1}H NMR Spectrum of **1h**

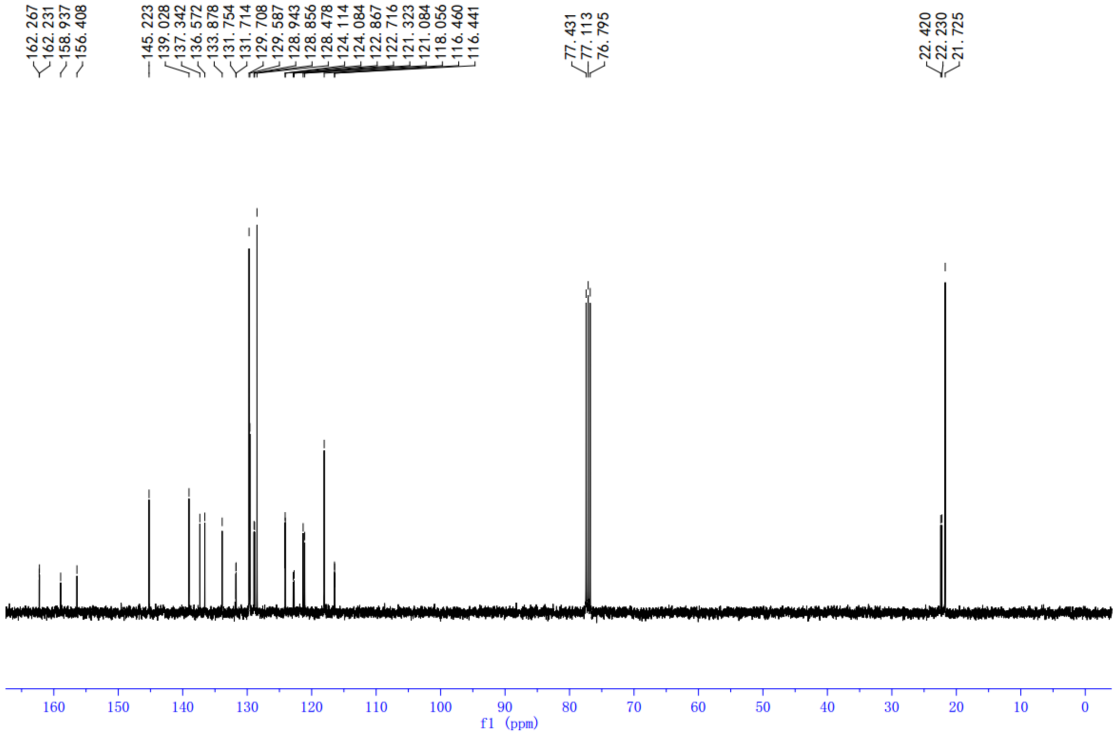

Supplementary Figure 19. ^{13}C NMR Spectrum of **1h**

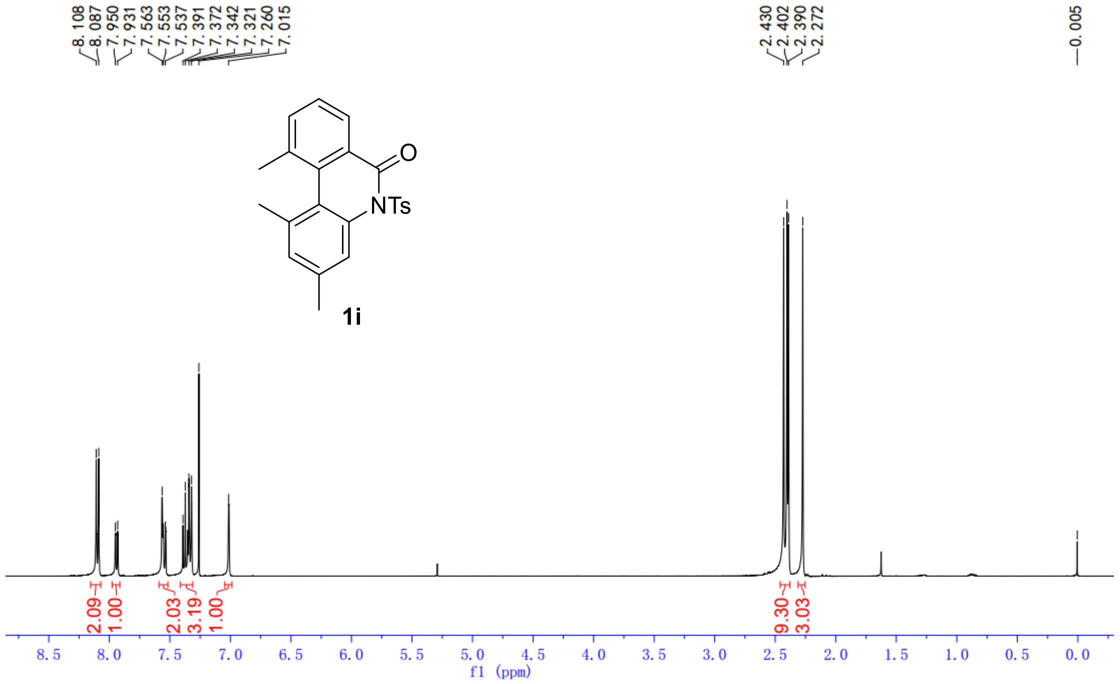

Supplementary Figure 20. ^{1}H NMR Spectrum of **1i**

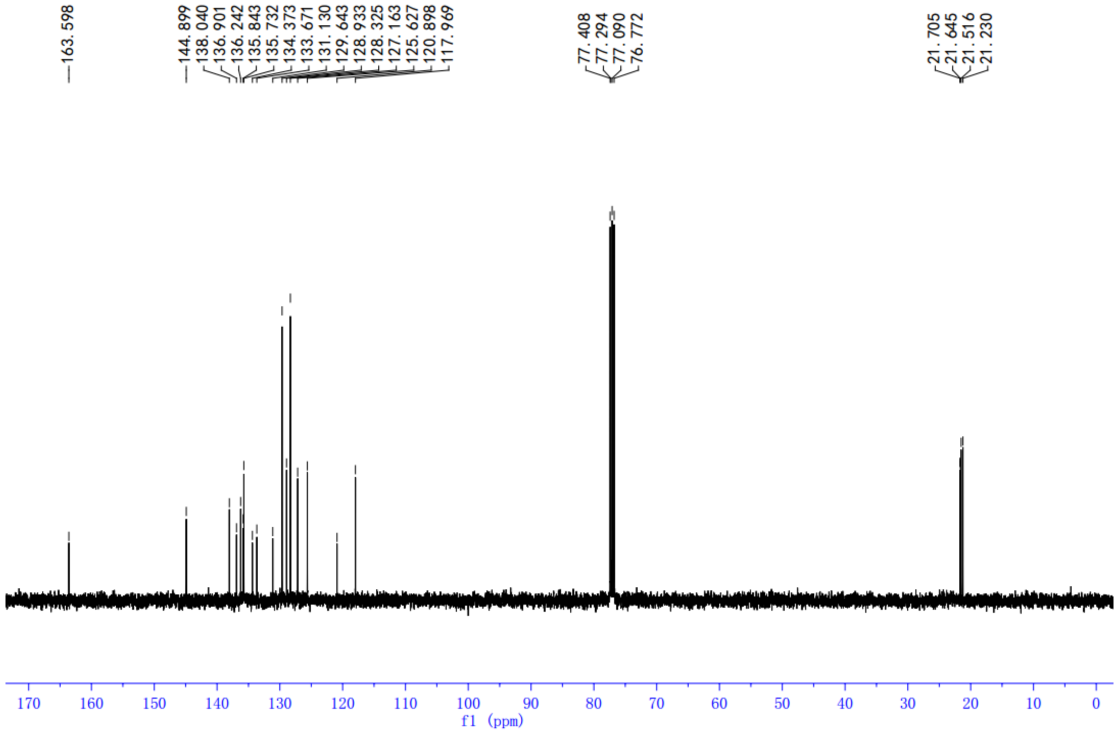

Supplementary Figure 21. ^{13}C NMR Spectrum of **1i**

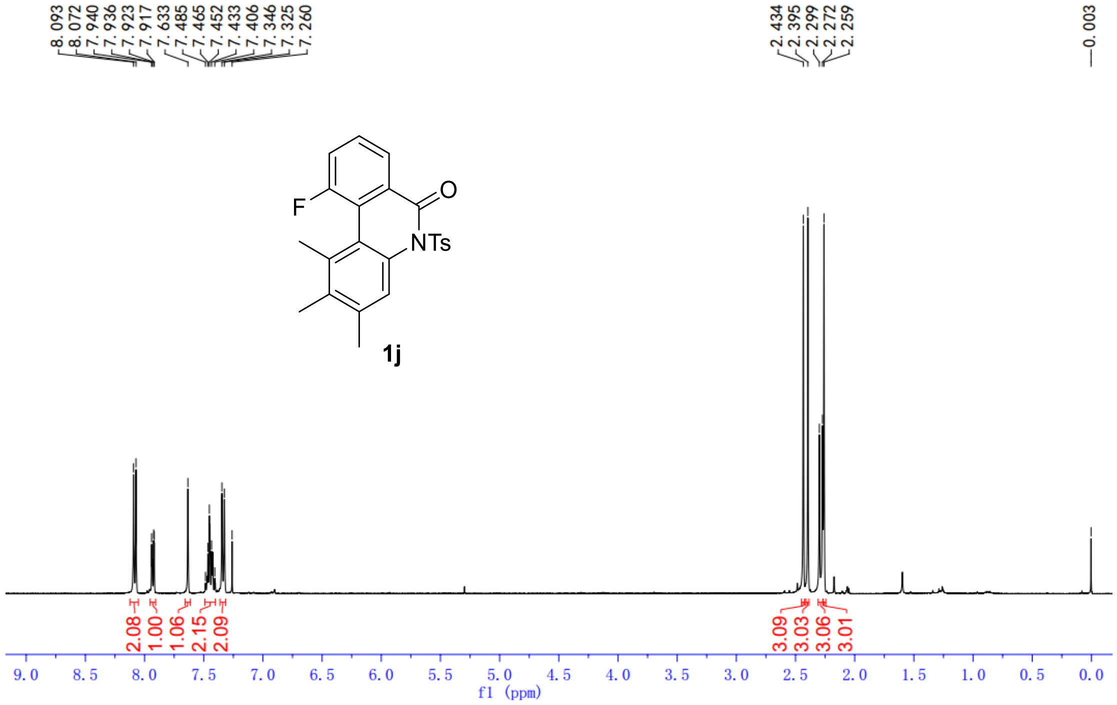

Supplementary Figure 22. ^{1}H NMR Spectrum of **1j**

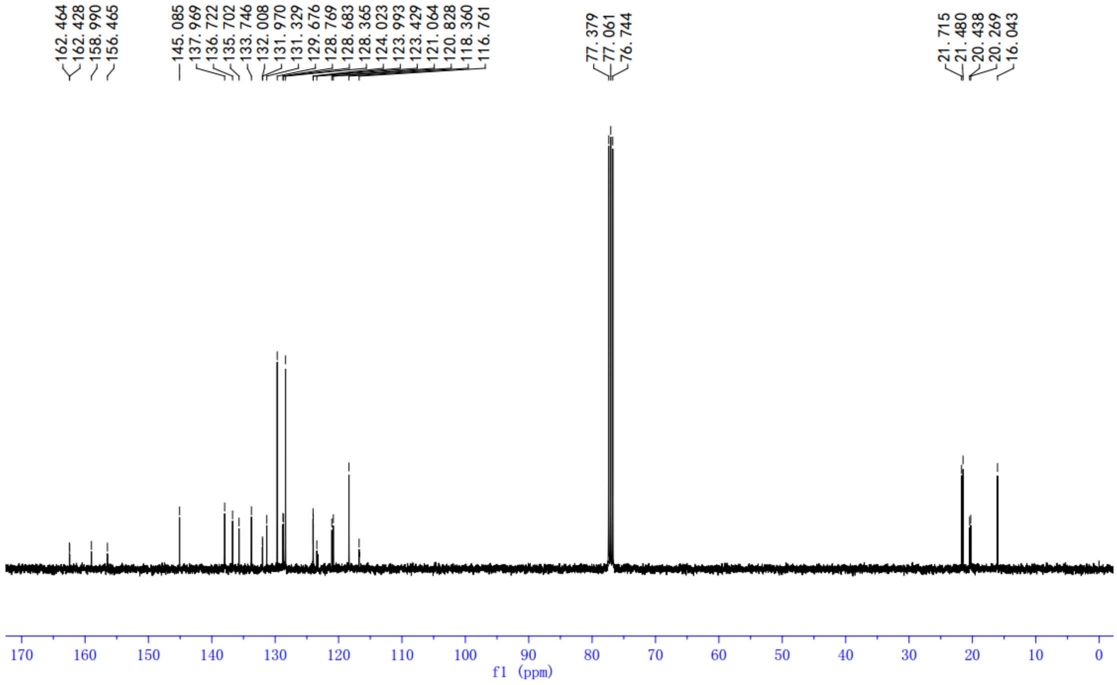

Supplementary Figure 23. ^{13}C NMR Spectrum of **1j**

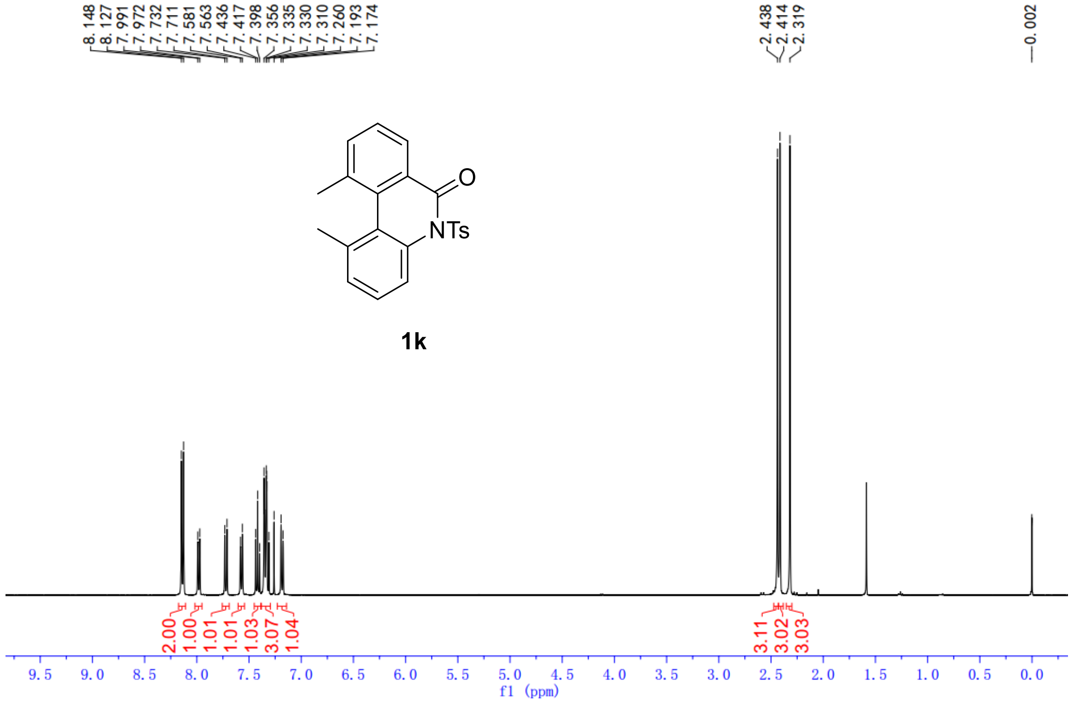

Supplementary Figure 24. ^{1}H NMR Spectrum of **1k**

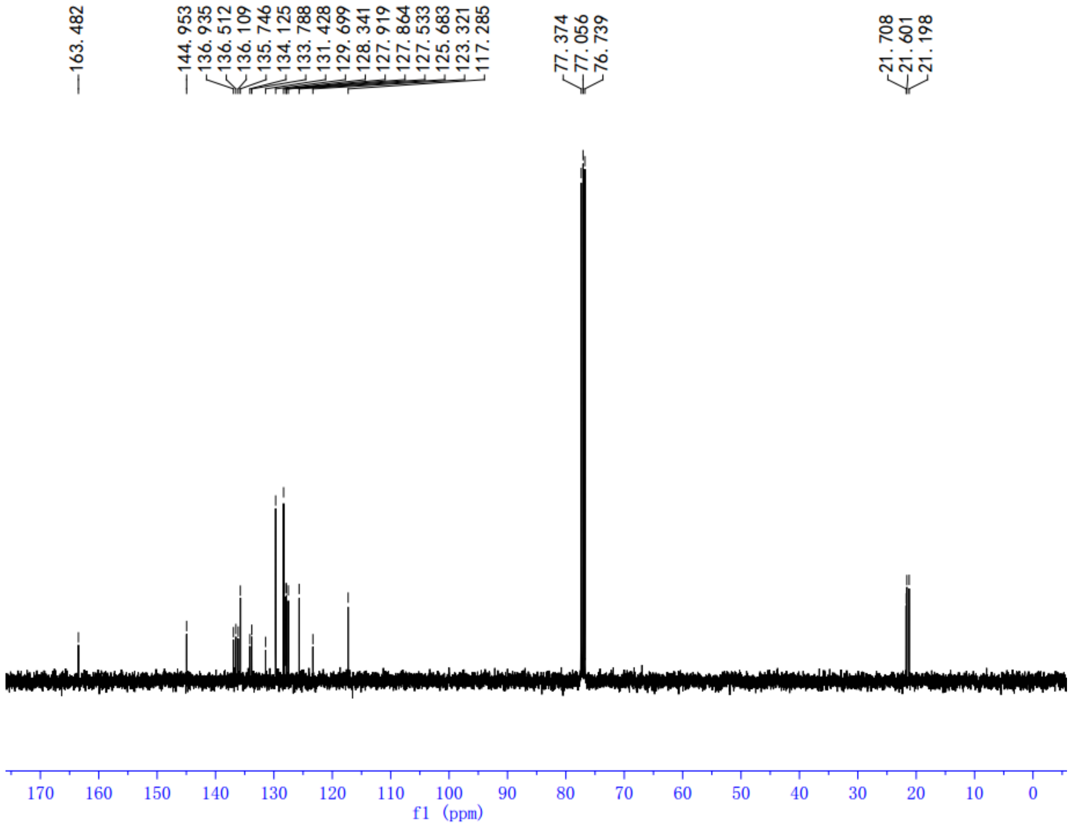

Supplementary Figure 25. ^{13}C NMR Spectrum of **1k**

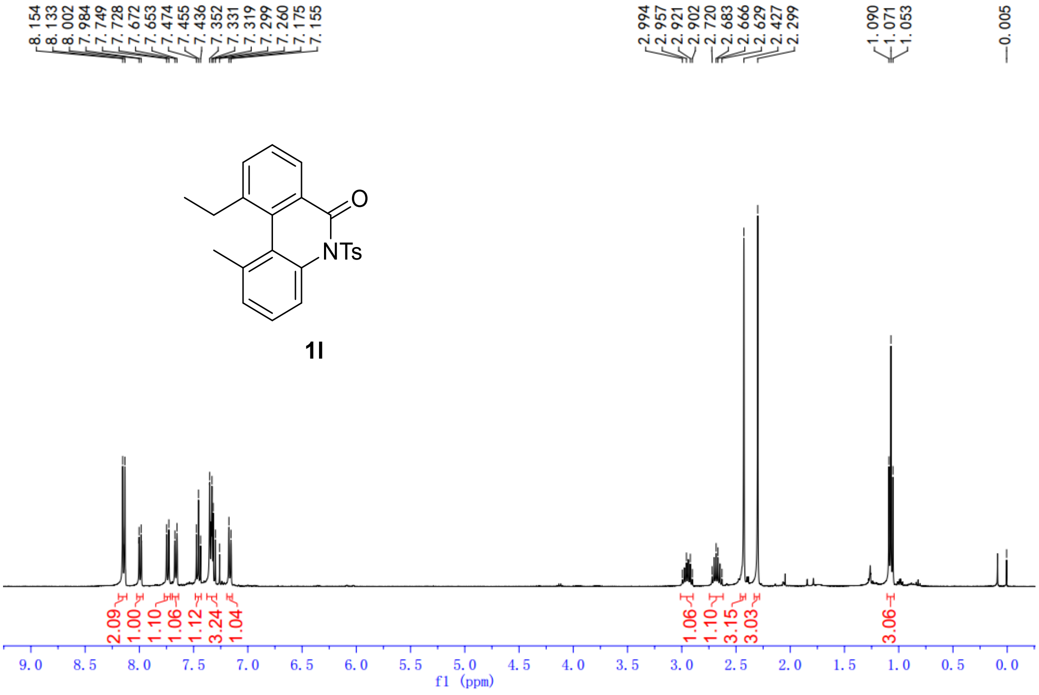

Supplementary Figure 26. ^{1}H NMR Spectrum of **1l**

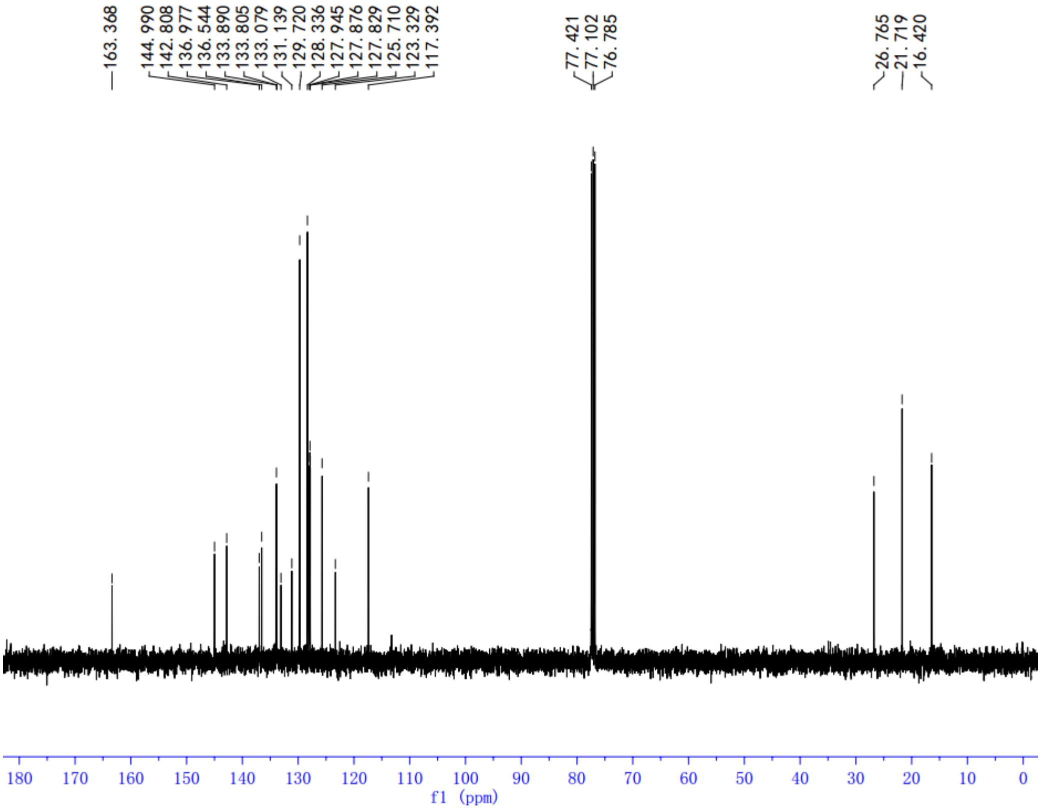

Supplementary Figure 27. ^{13}C NMR Spectrum of **1l**

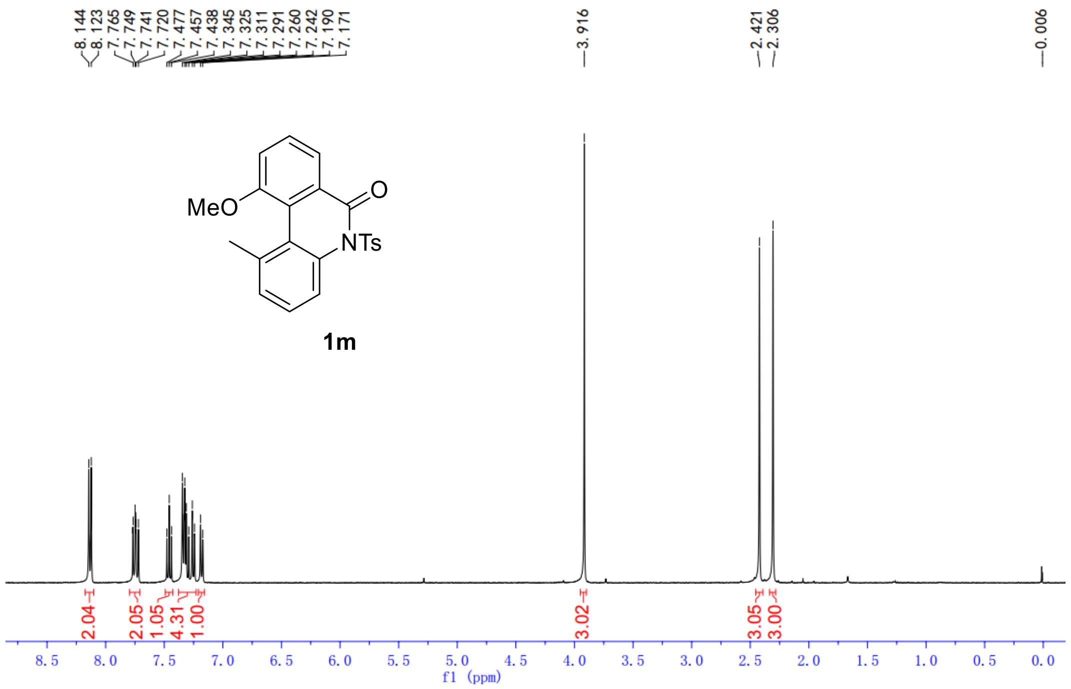

Supplementary Figure 28. ^{1}H NMR Spectrum of **1m**

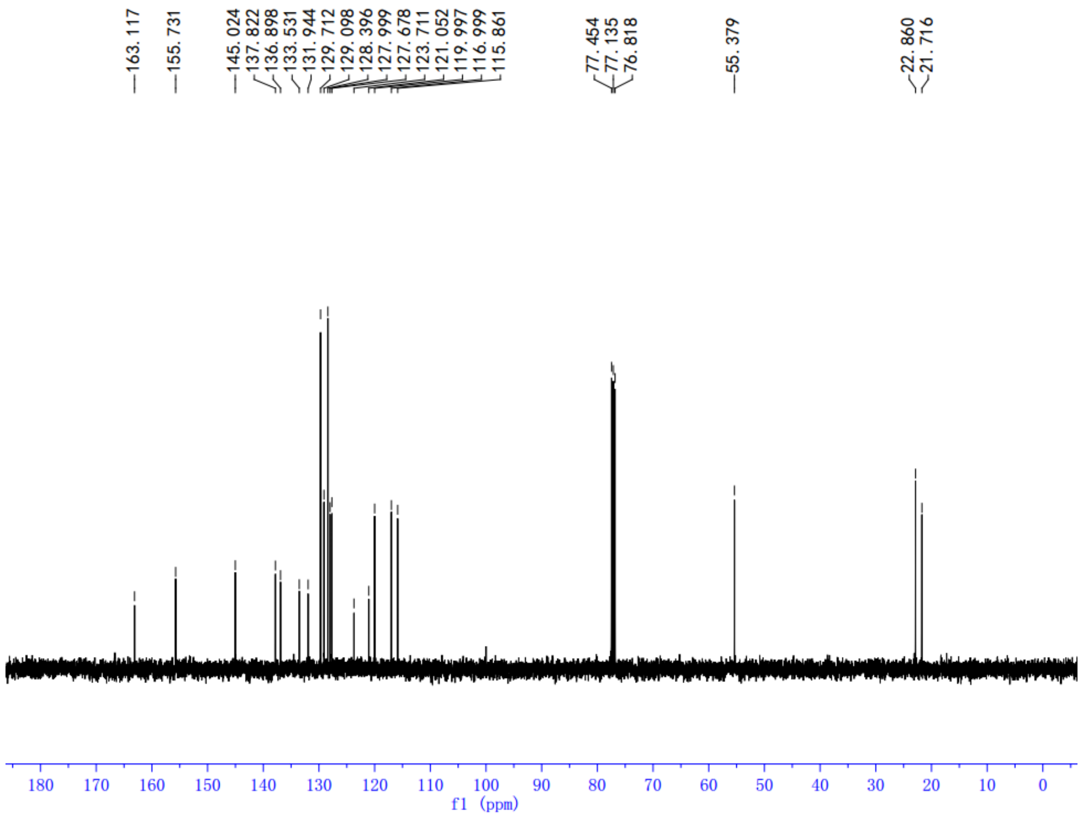

Supplementary Figure 29. ^{13}C NMR Spectrum of **1m**

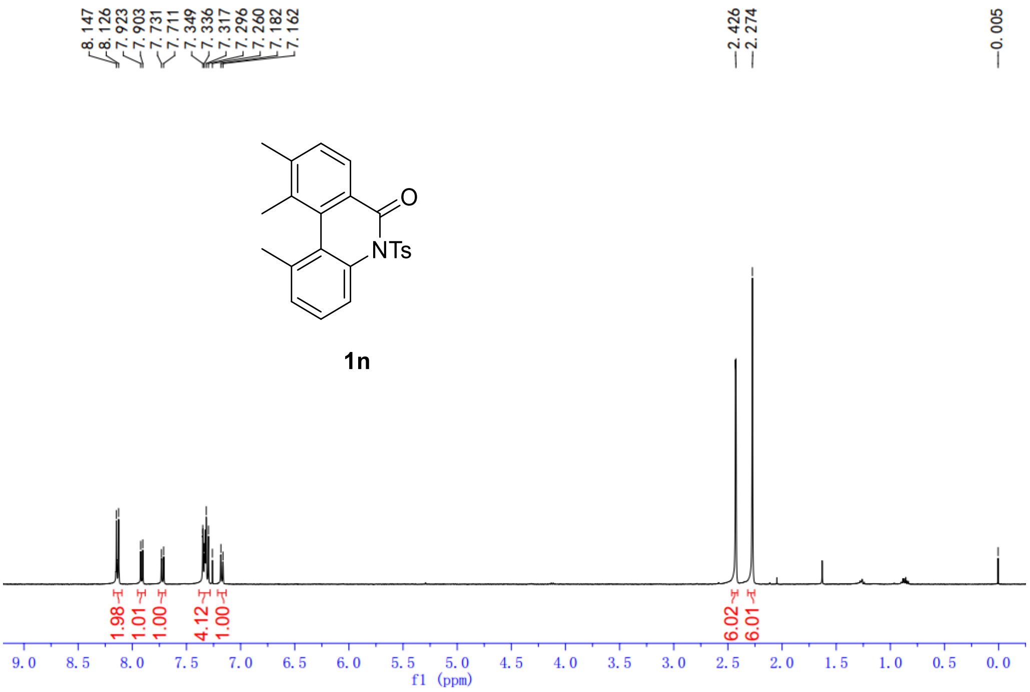

Supplementary Figure 30. ^{1}H NMR Spectrum of **1n**

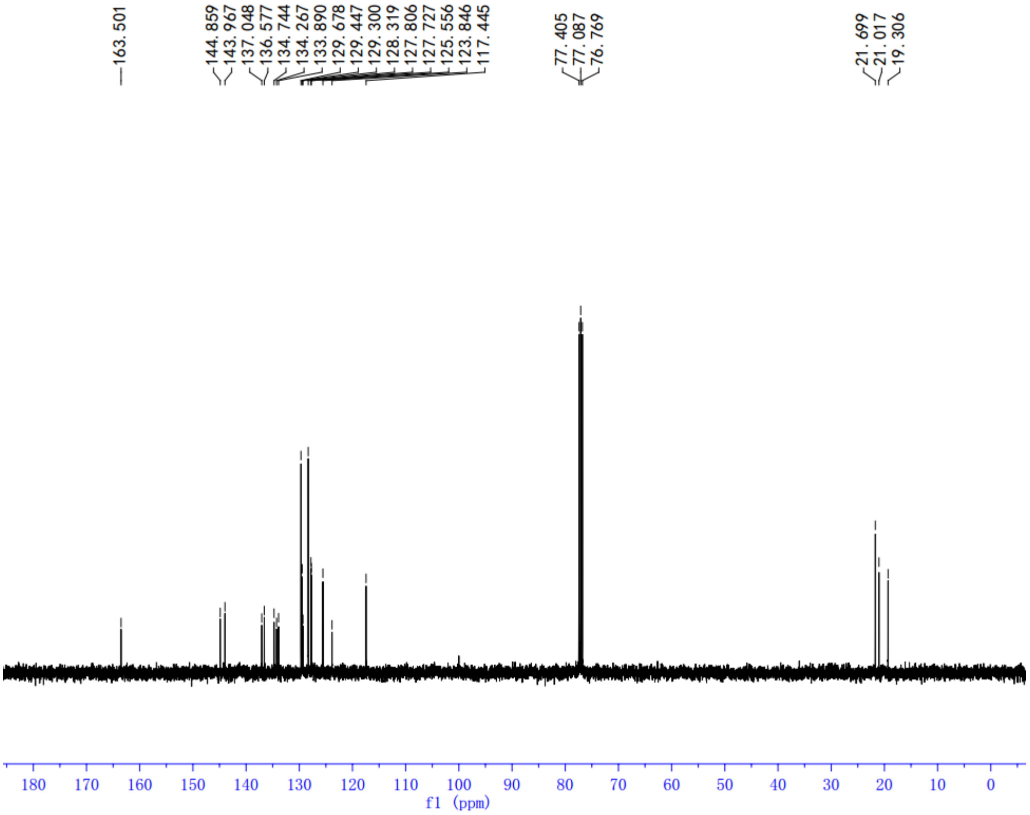

Supplementary Figure 31. ^{13}C NMR Spectrum of **1n**

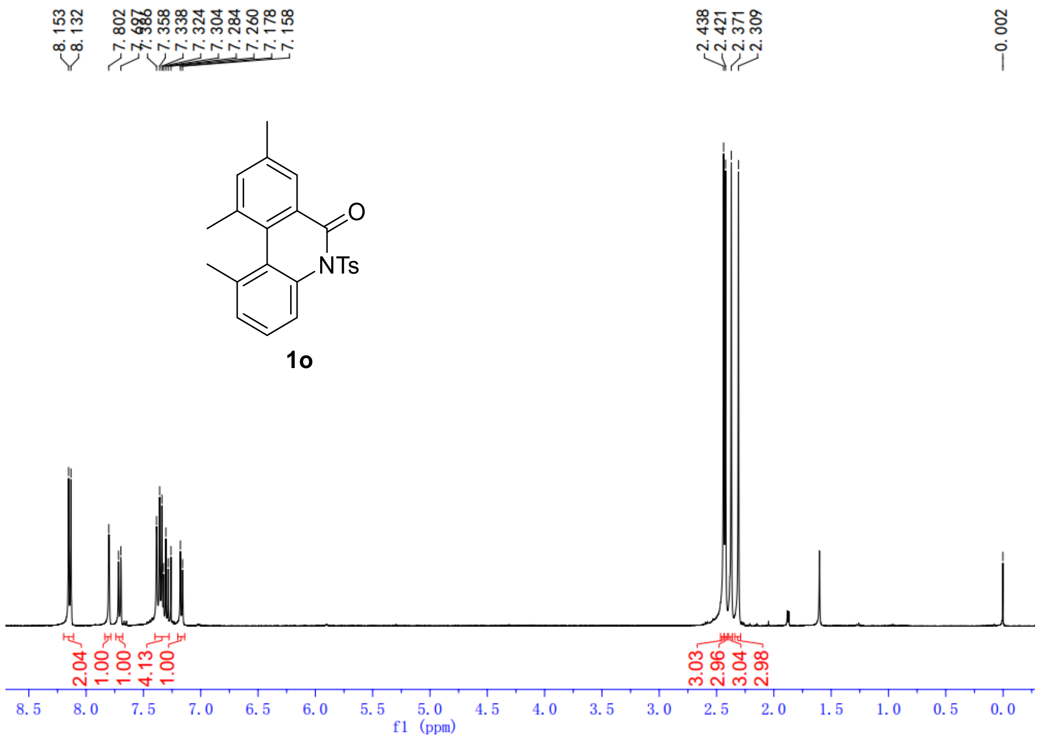

Supplementary Figure 32. ^{1}H NMR Spectrum of **1o**

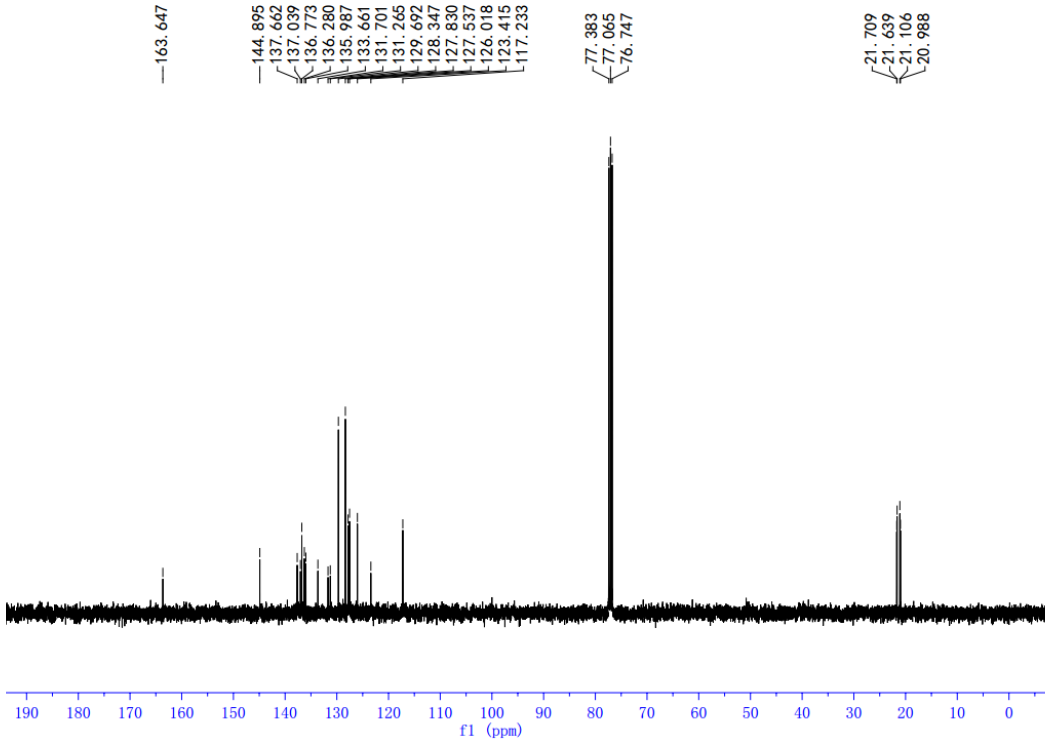

Supplementary Figure 33. ^{13}C NMR Spectrum of **1o**

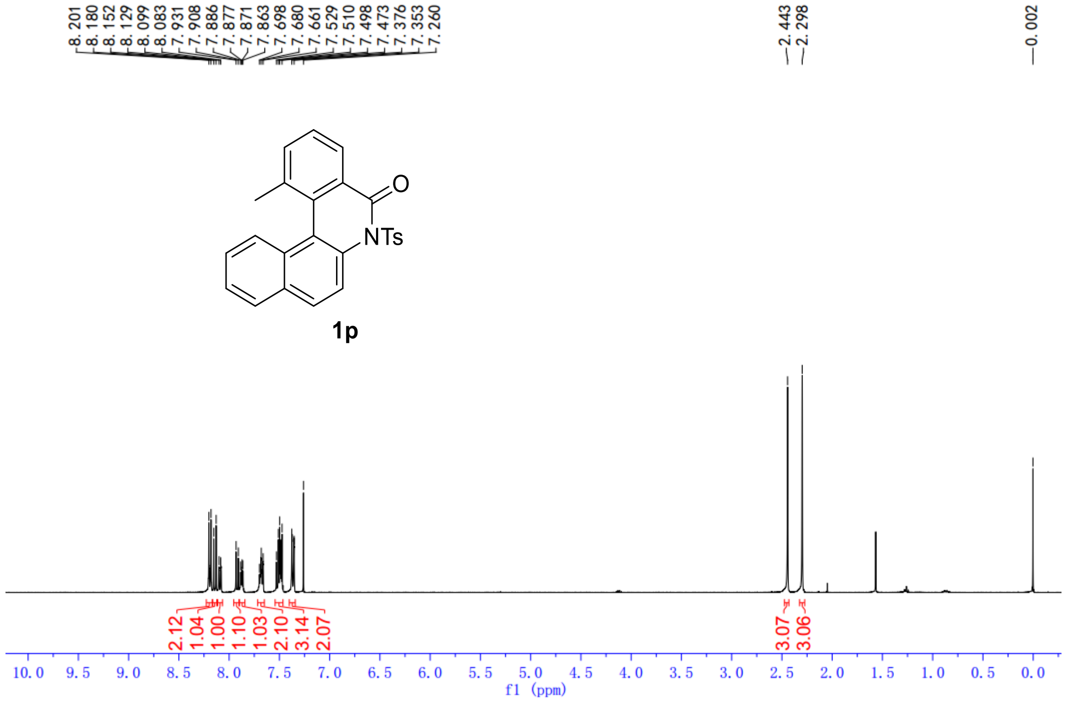

Supplementary Figure 34. ^{1}H NMR Spectrum of **1p**

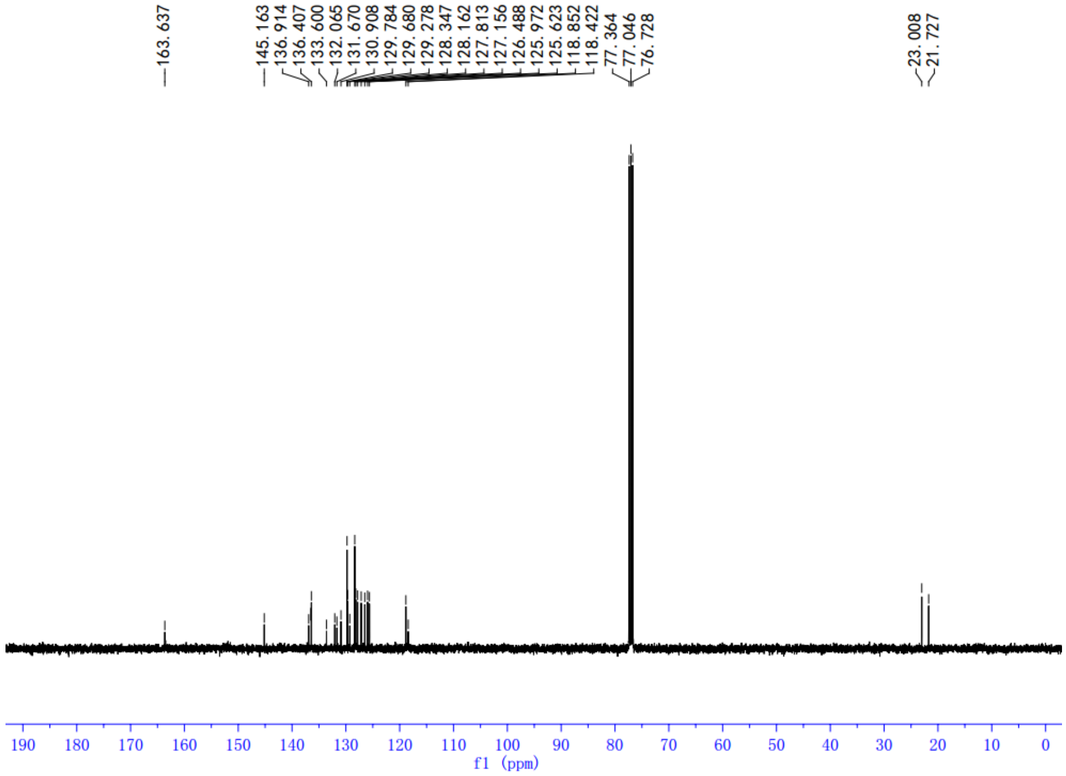

Supplementary Figure 35. ^{13}C NMR Spectrum of **1p**

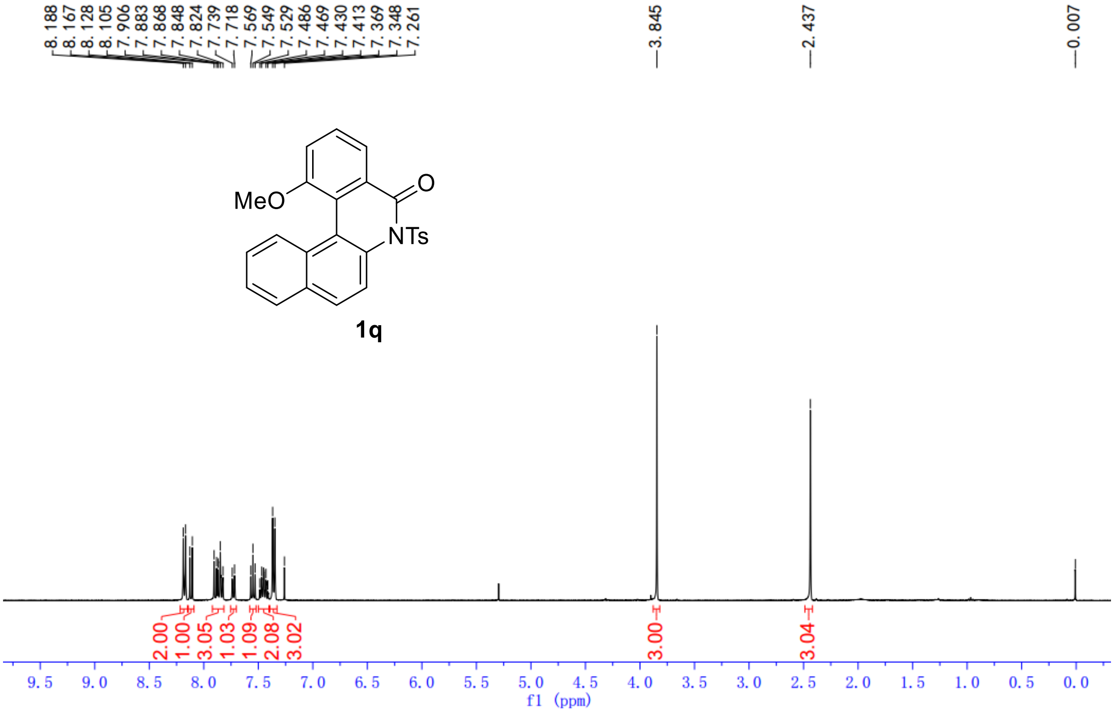

Supplementary Figure 36. ^{1}H NMR Spectrum of **1q**

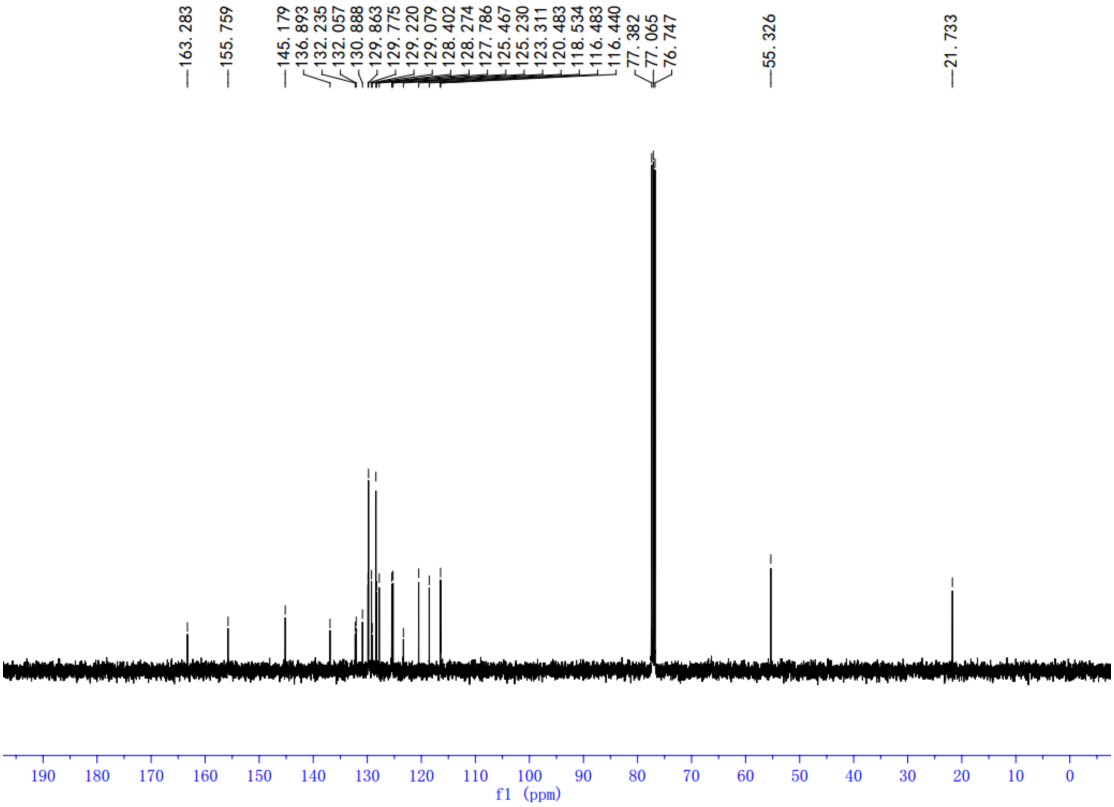

Supplementary Figure 37. ^{13}C NMR Spectrum of **1q**

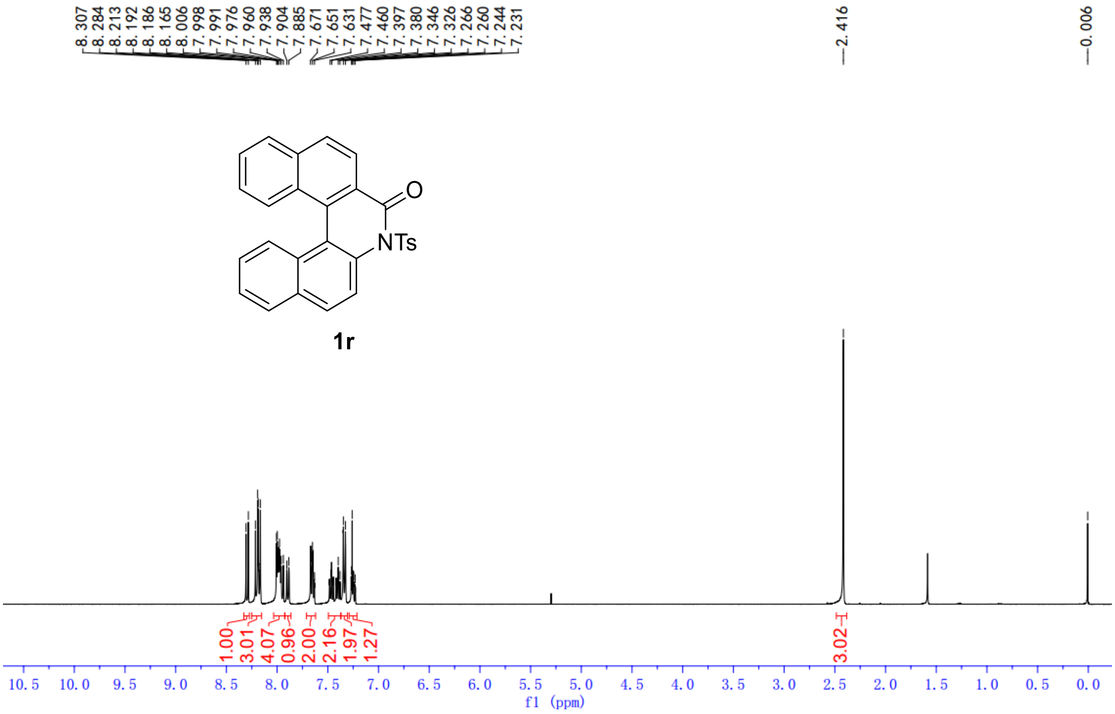

Supplementary Figure 38. ^{1}H NMR Spectrum of **1r**

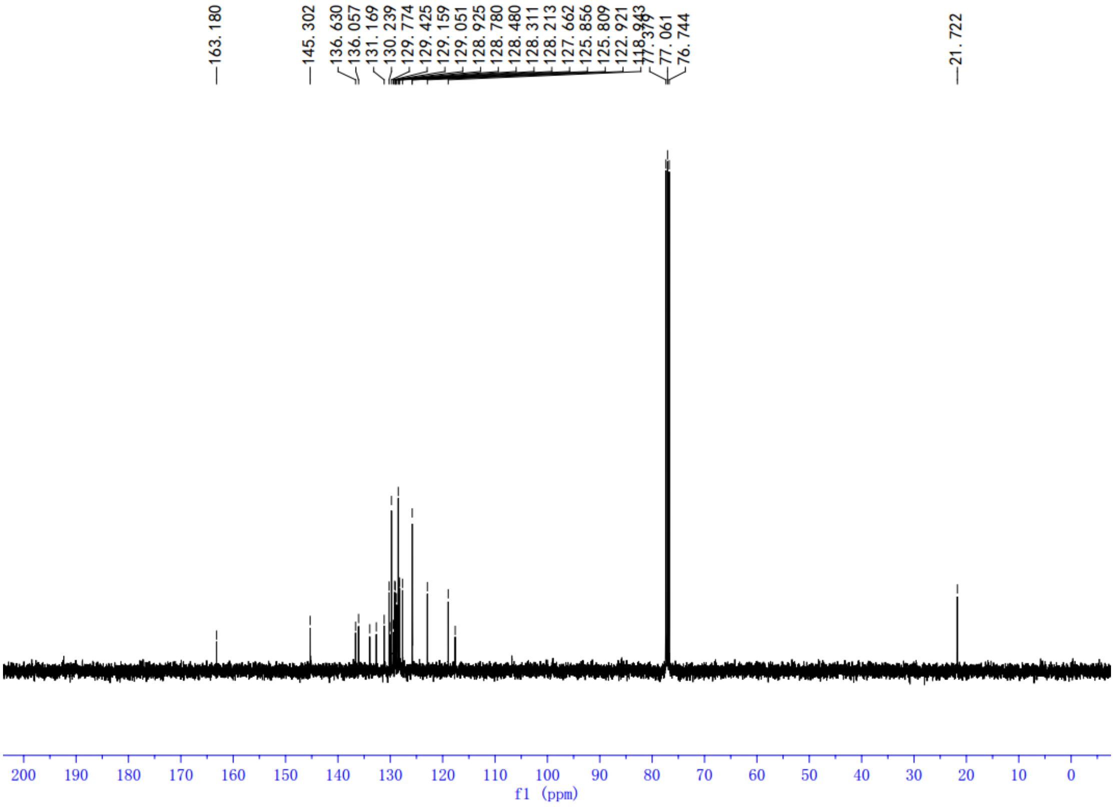

Supplementary Figure 39. ^{13}C NMR Spectrum of **1r**

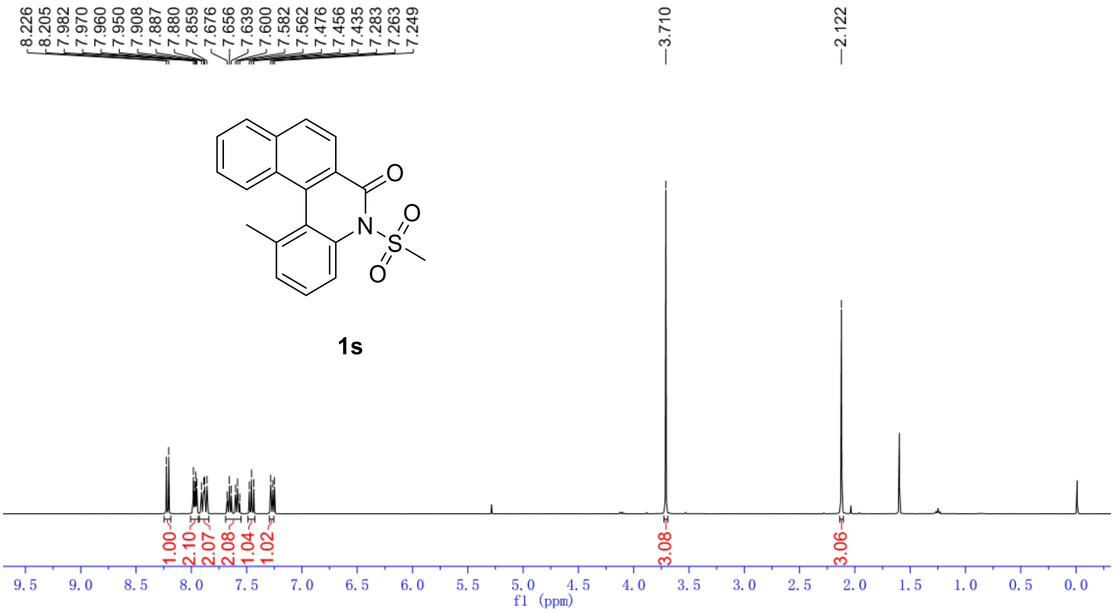

Supplementary Figure 40. ^{1}H NMR Spectrum of **1s**

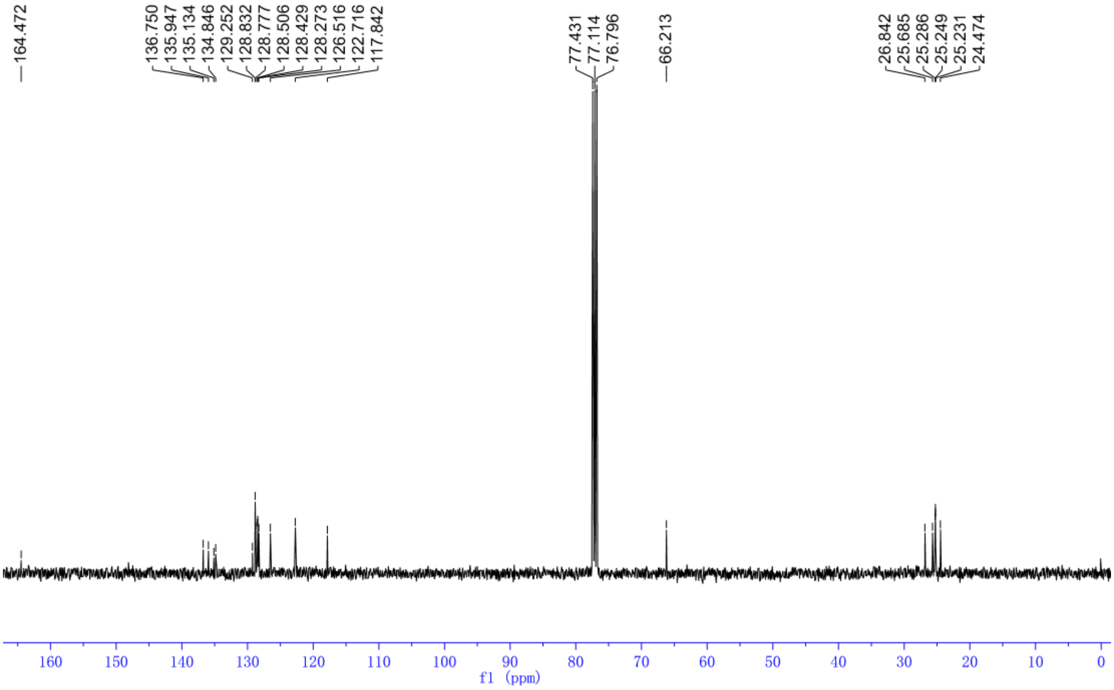

Supplementary Figure 41. ^{13}C NMR Spectrum of **1s**

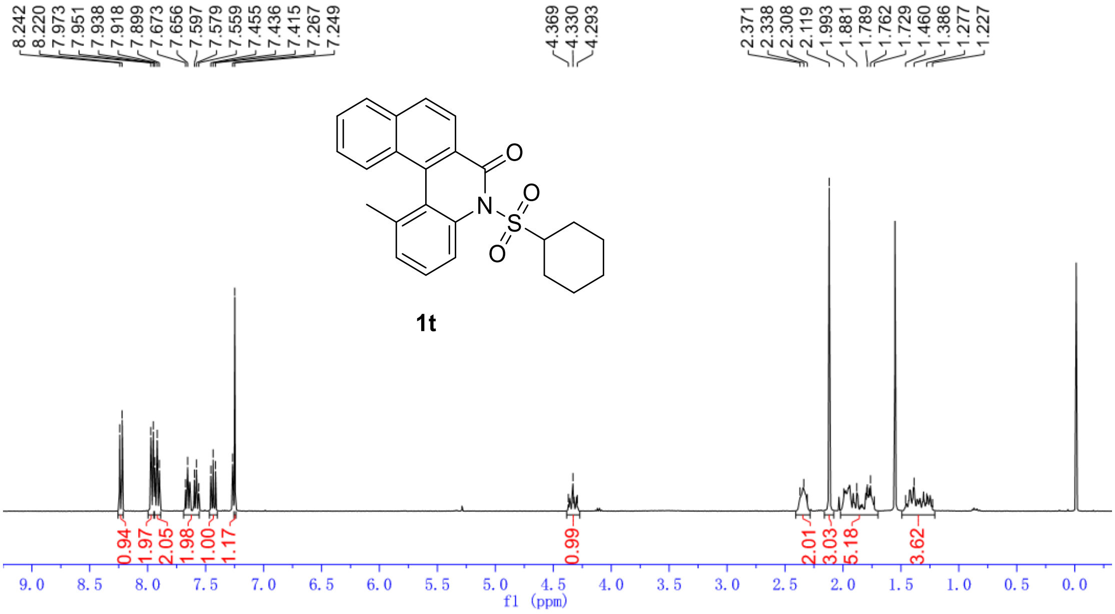

Supplementary Figure 42. ^{1}H NMR Spectrum of **1t**

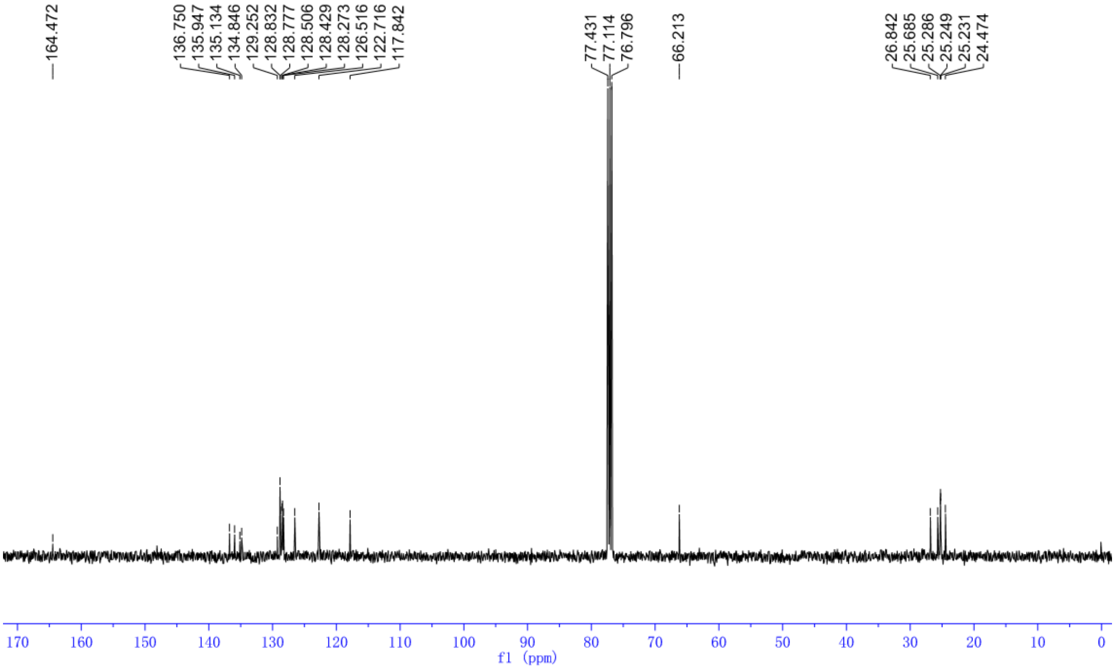

Supplementary Figure 43. ^{13}C NMR Spectrum of **1t**

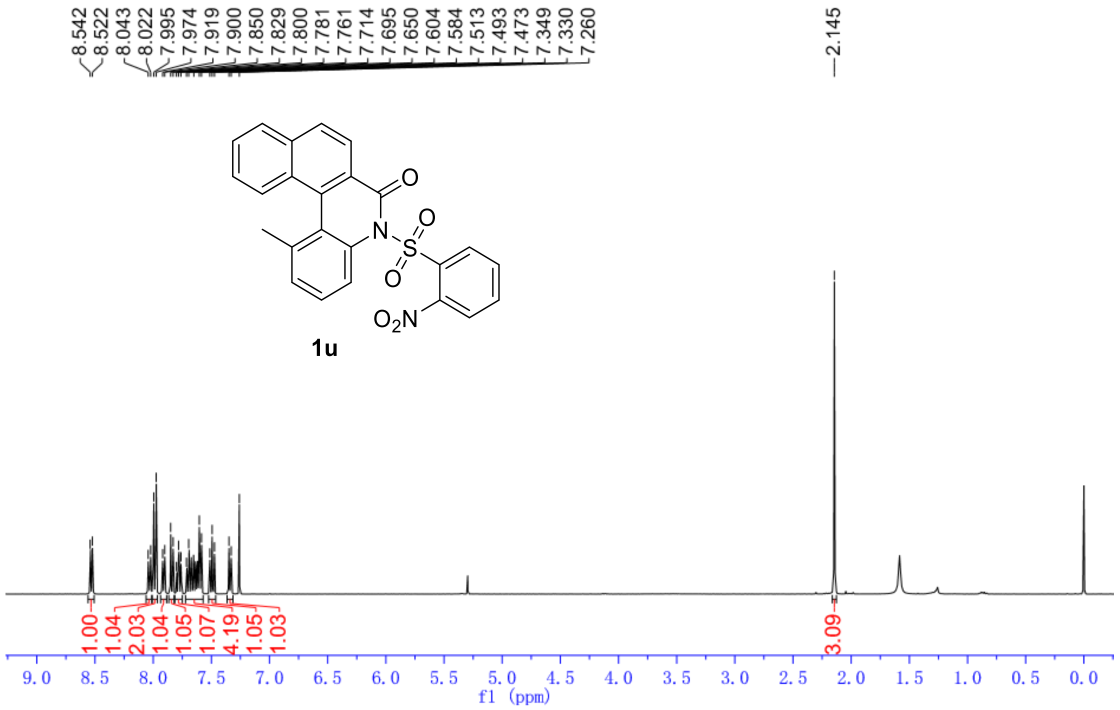

Supplementary Figure 44. ^{1}H NMR Spectrum of **1u**

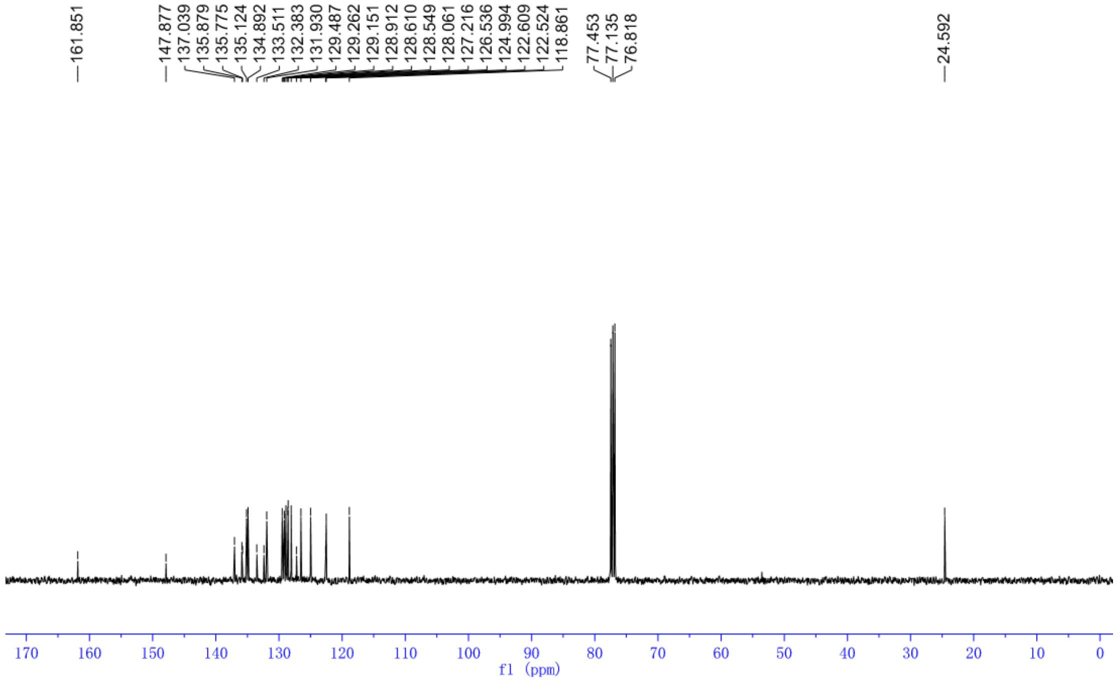

Supplementary Figure 45. ^{13}C NMR Spectrum of **1u**

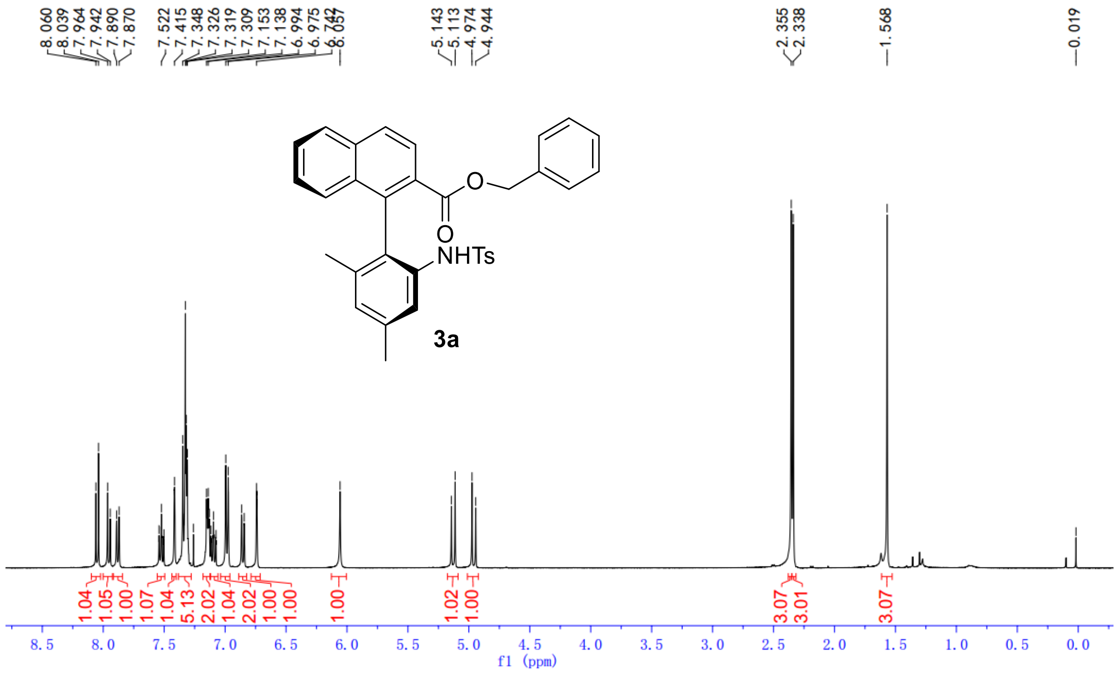

Supplementary Figure 46. ^{1}H NMR Spectrum of **3a**

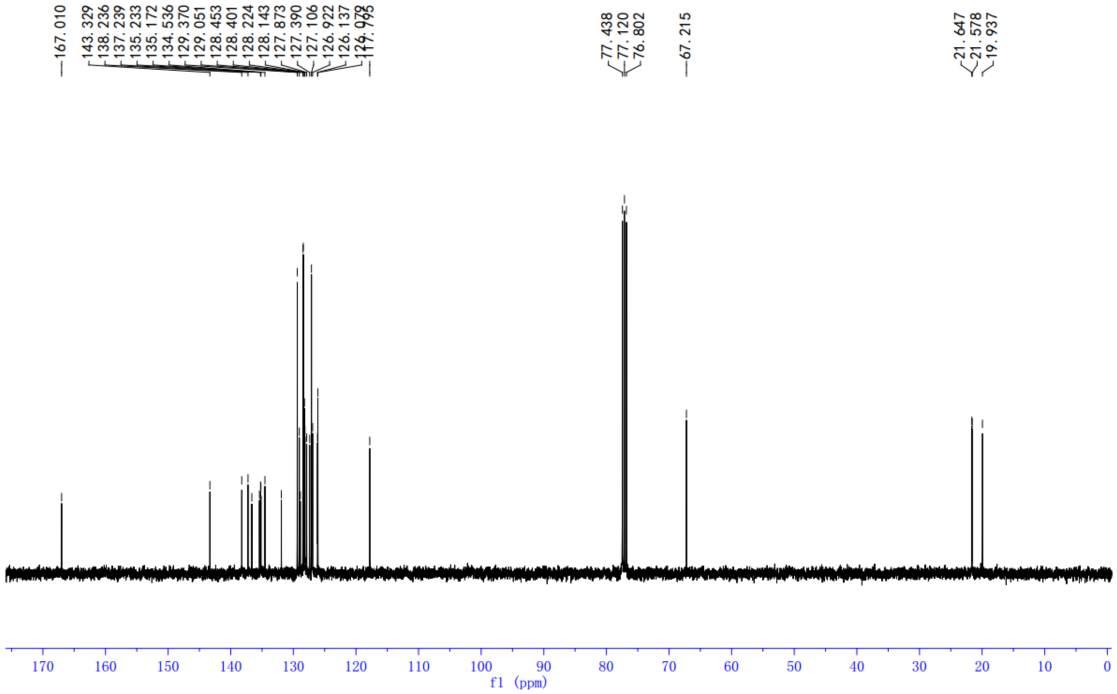

Supplementary Figure 47. ^{13}C NMR Spectrum of **3a**

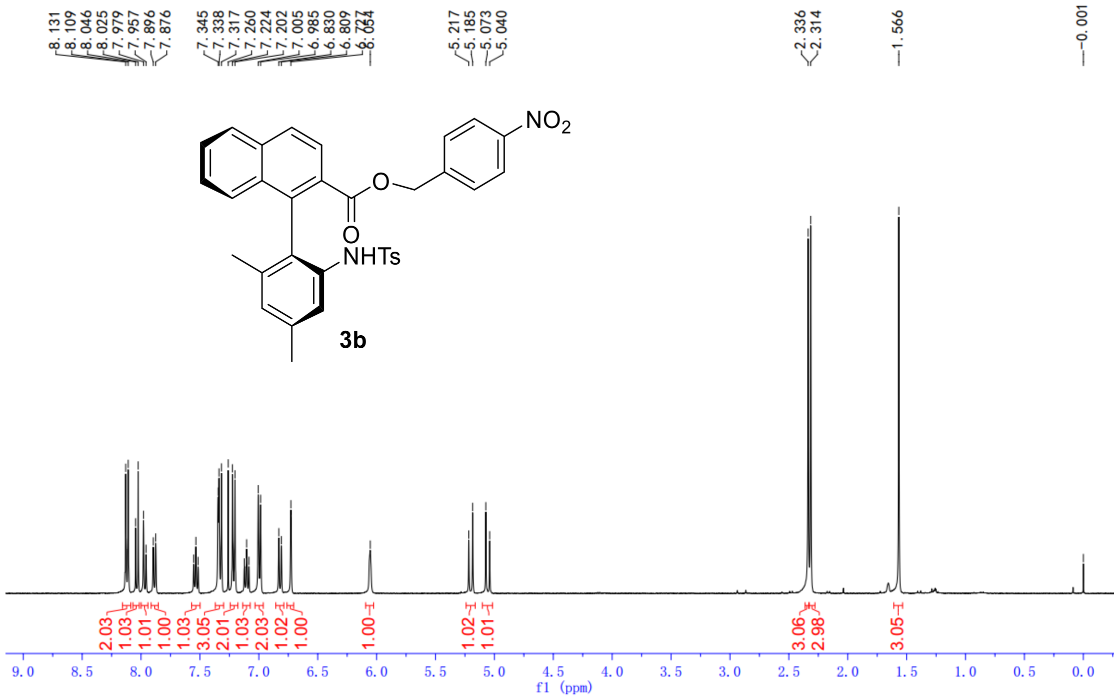

Supplementary Figure 48. ^{1}H NMR Spectrum of **3b**

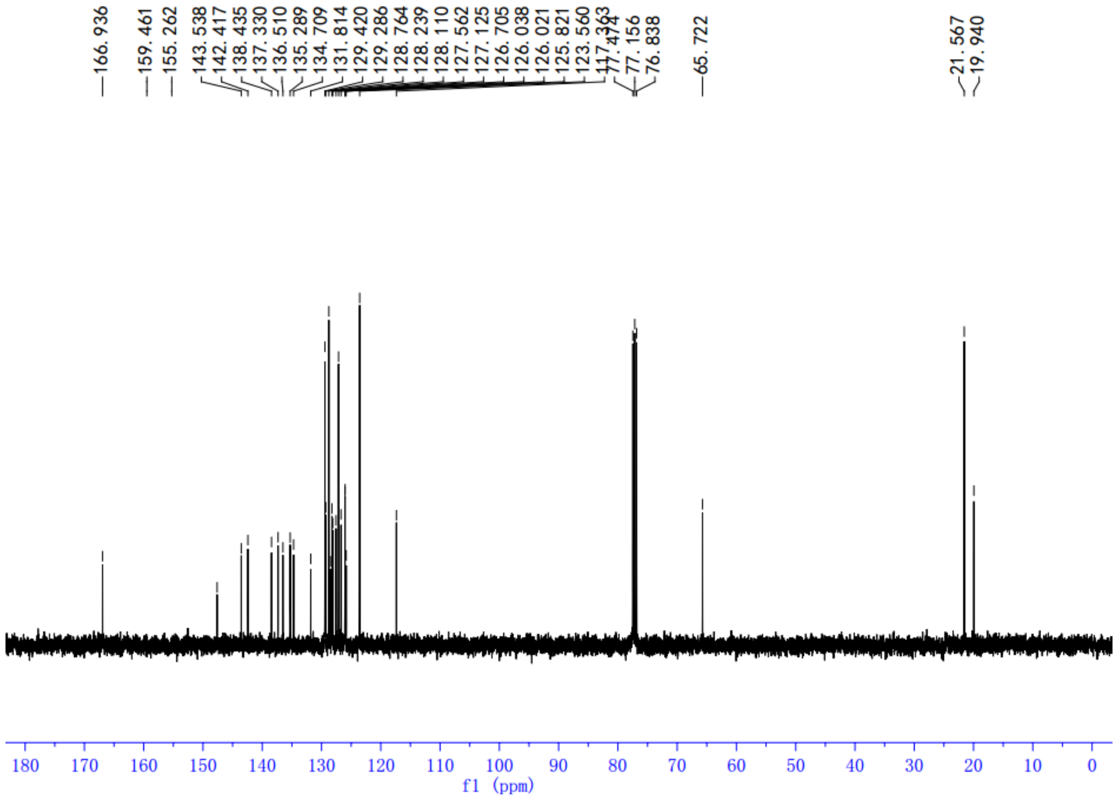

Supplementary Figure 49. ^{13}C NMR Spectrum of **3b**

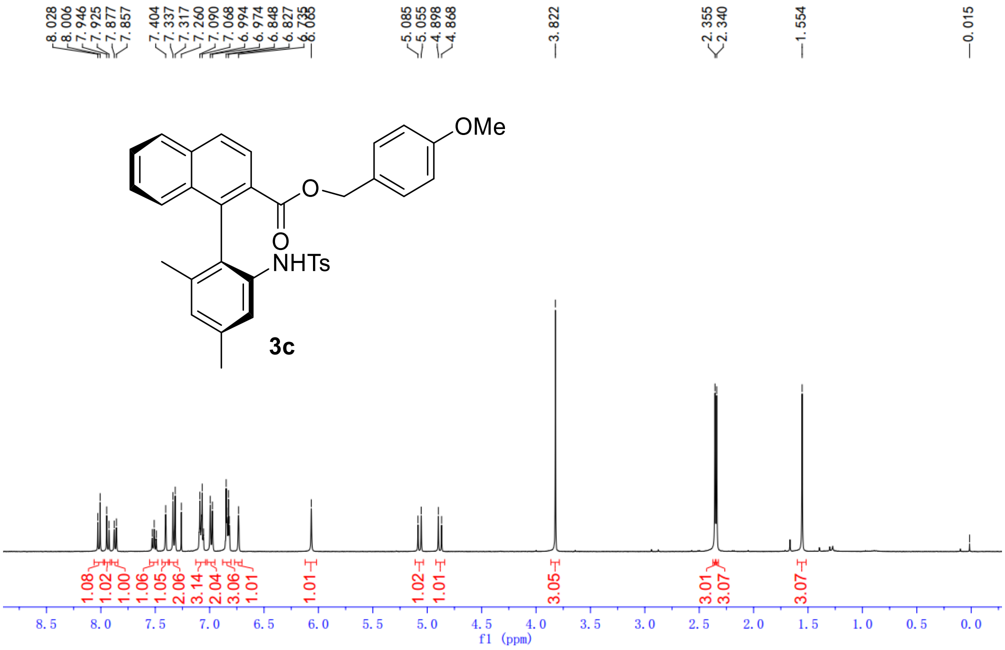

Supplementary Figure 50. ^{1}H NMR Spectrum of **3c**

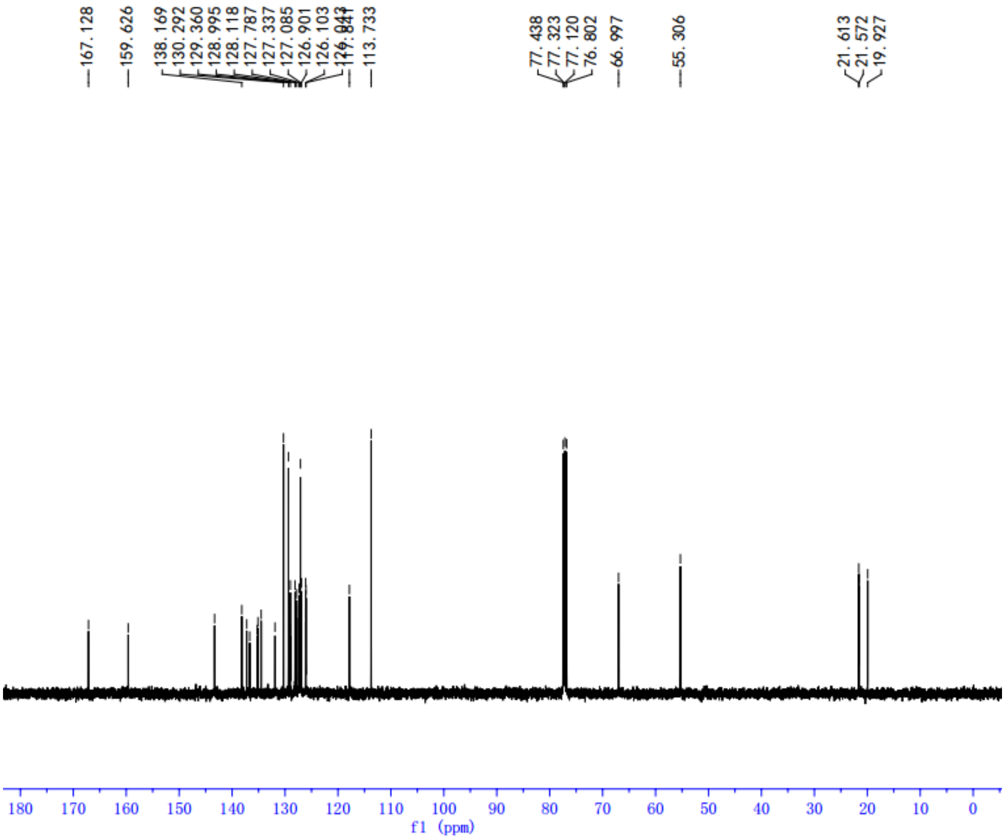

Supplementary Figure 51. ^{13}C NMR Spectrum of **3c**

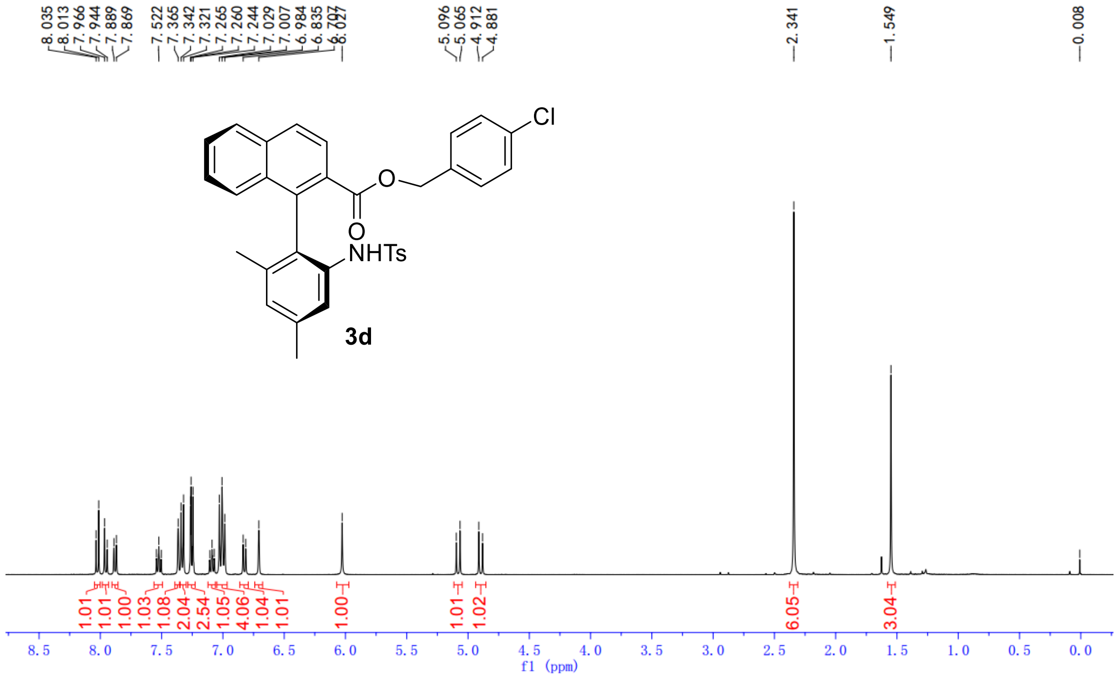

Supplementary Figure 52. ^{1}H NMR Spectrum of **3d**

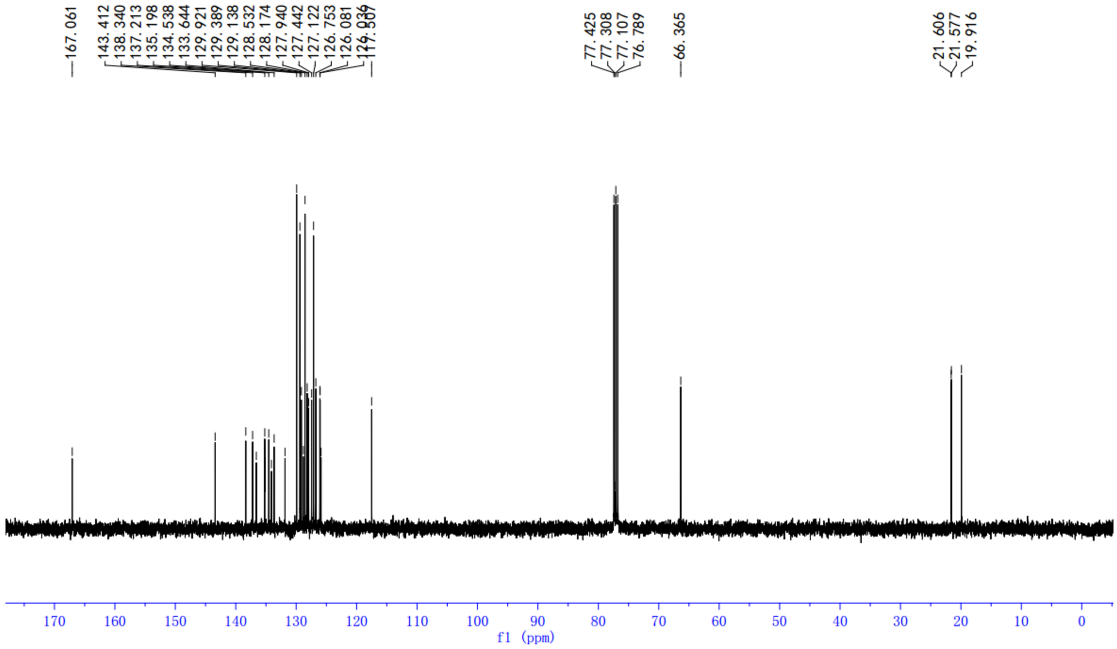

Supplementary Figure 53. ^{13}C NMR Spectrum of **3d**

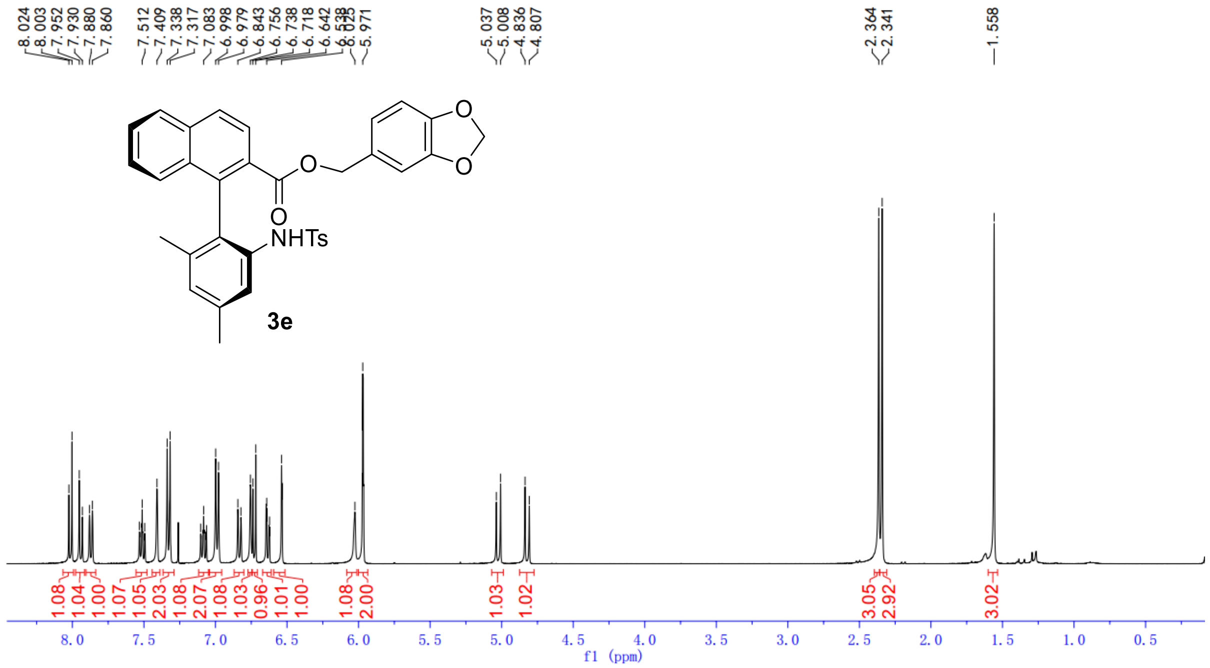

Supplementary Figure 54. ^{1}H NMR Spectrum of **3e**

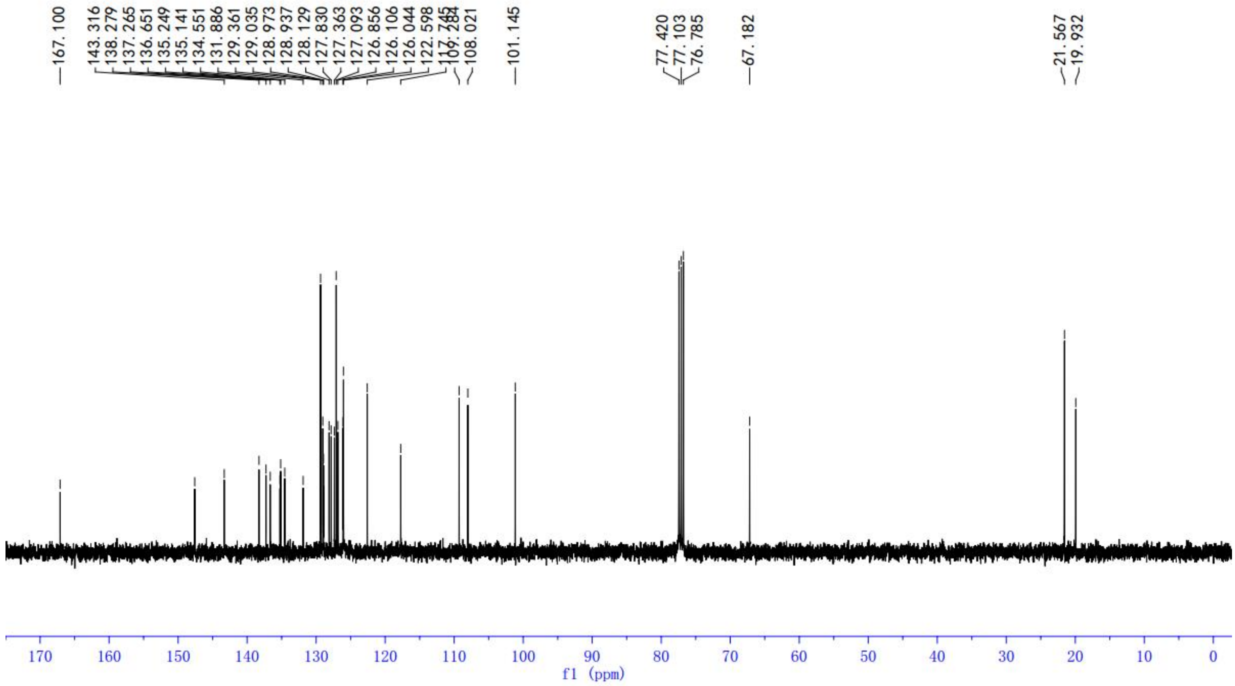

Supplementary Figure 55. ^{13}C NMR Spectrum of **3e**

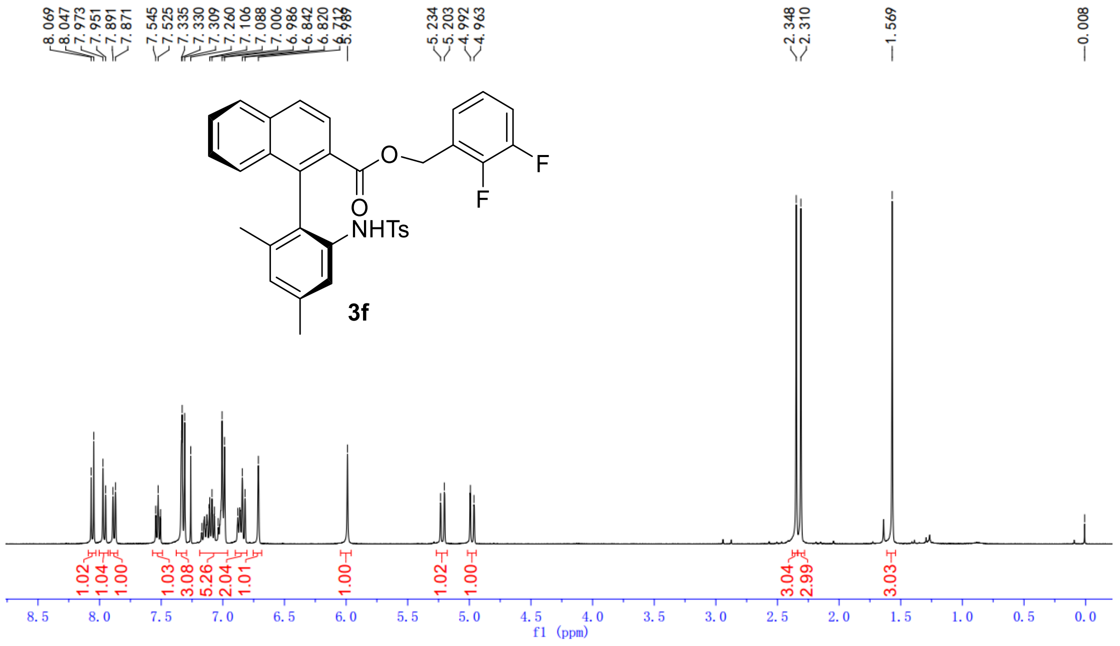

Supplementary Figure 56. ^{1}H NMR Spectrum of **3f**

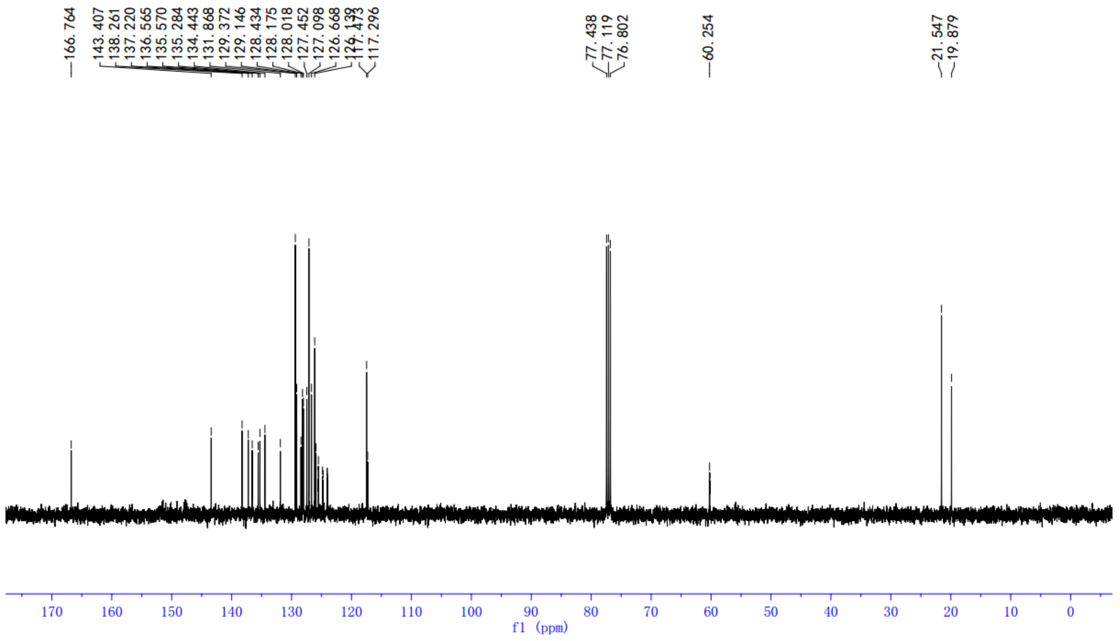

Supplementary Figure 57. ^{13}C NMR Spectrum of **3f**

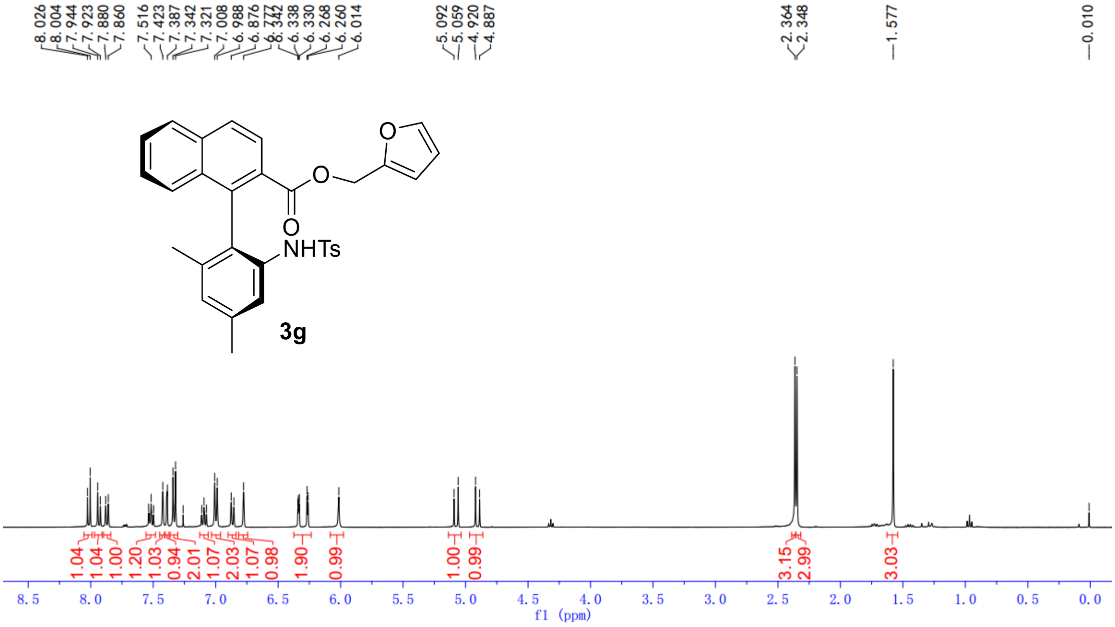

Supplementary Figure 58. ^{1}H NMR Spectrum of **3g**

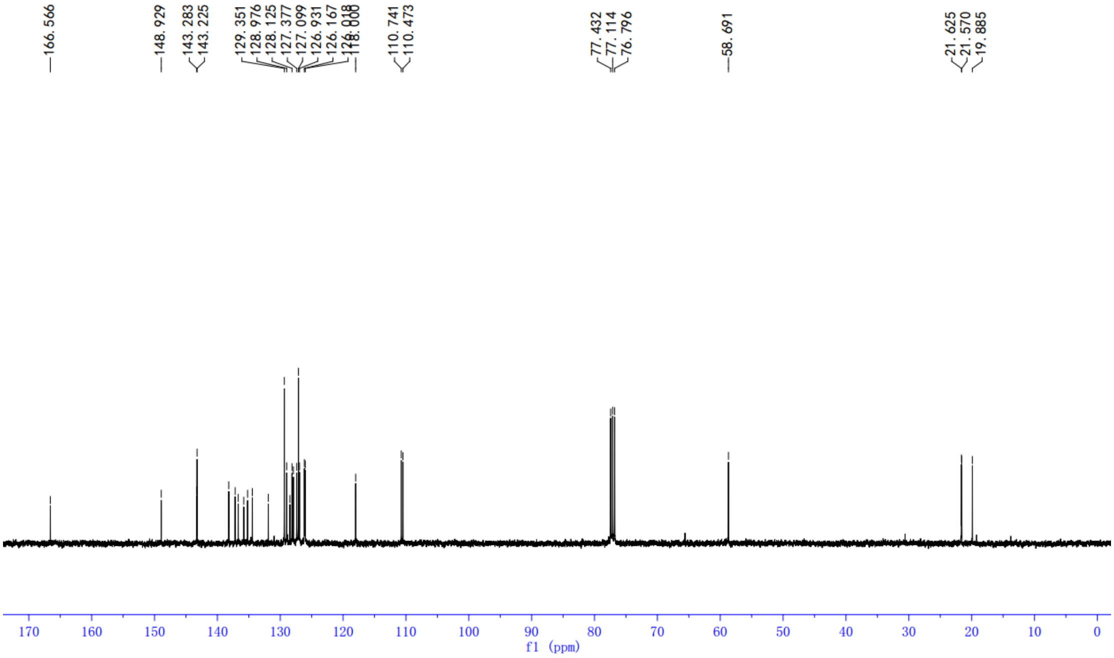

Supplementary Figure 59. ^{13}C NMR Spectrum of **3g**

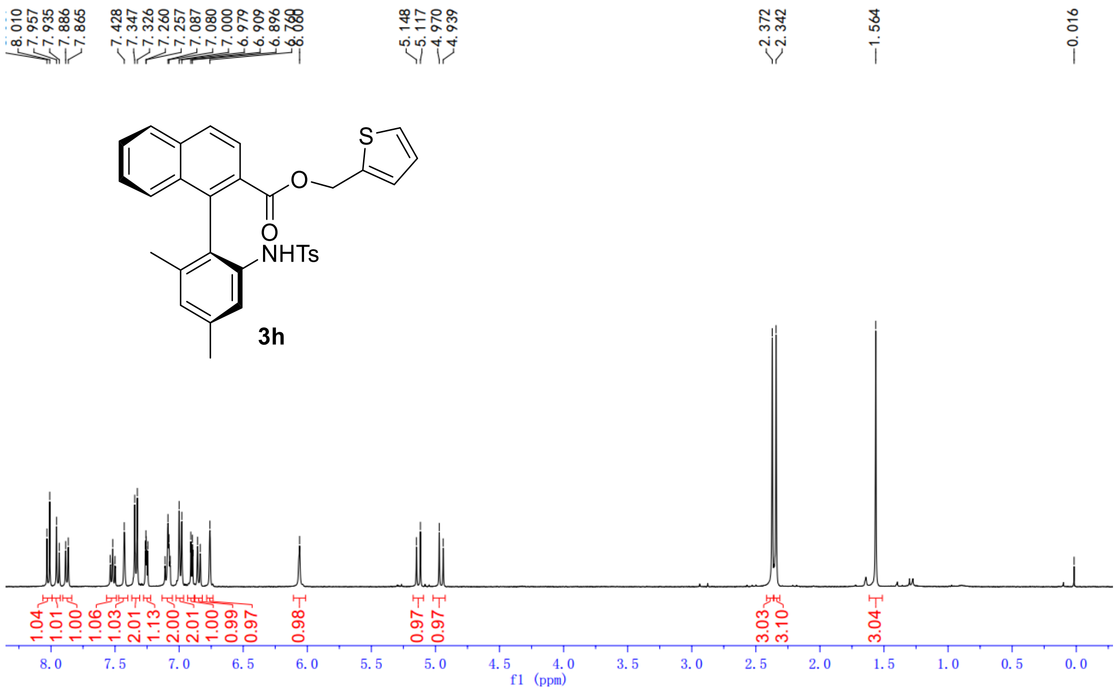

Supplementary Figure 60. ^{1}H NMR Spectrum of **3h**

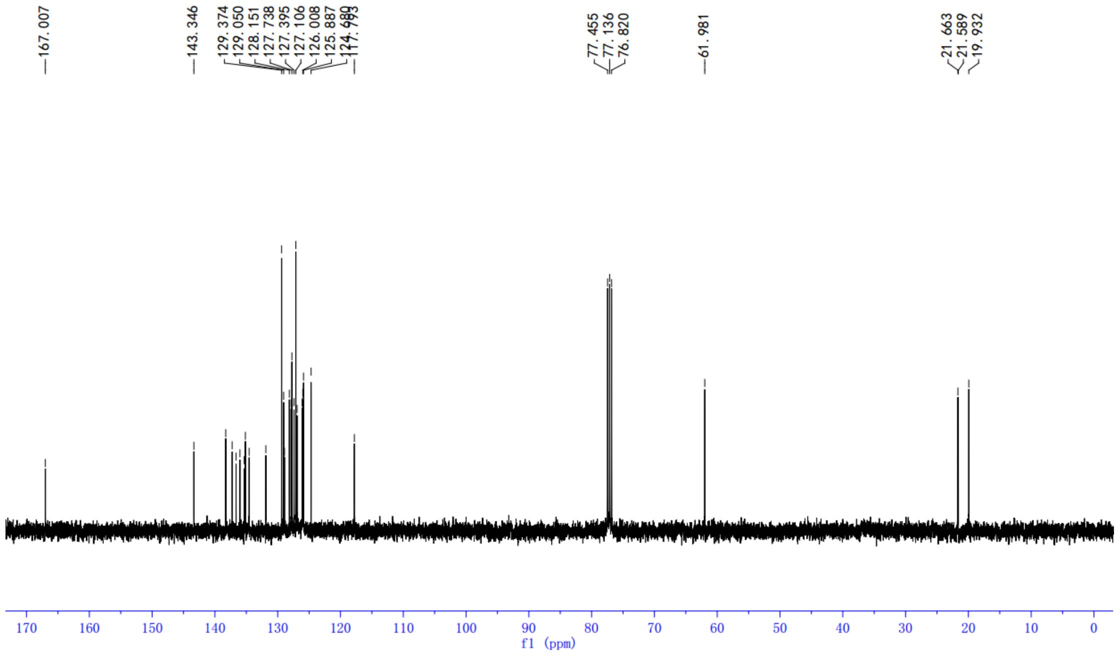

Supplementary Figure 61. ^{13}C NMR Spectrum of **3h**

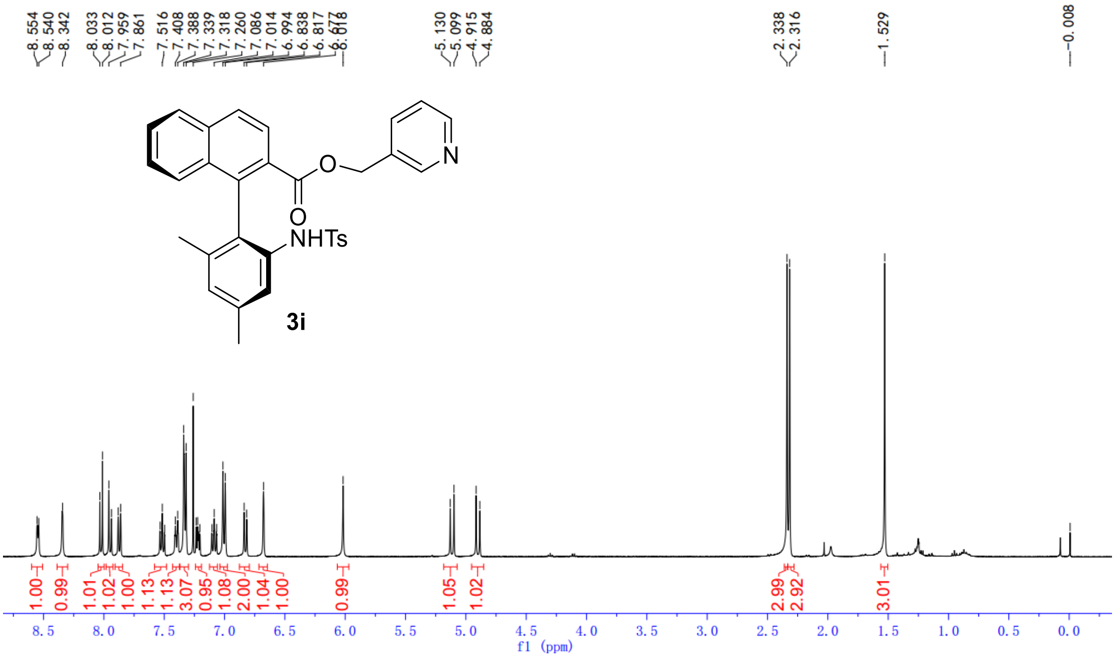

Supplementary Figure 62. ^{1}H NMR Spectrum of **3i**

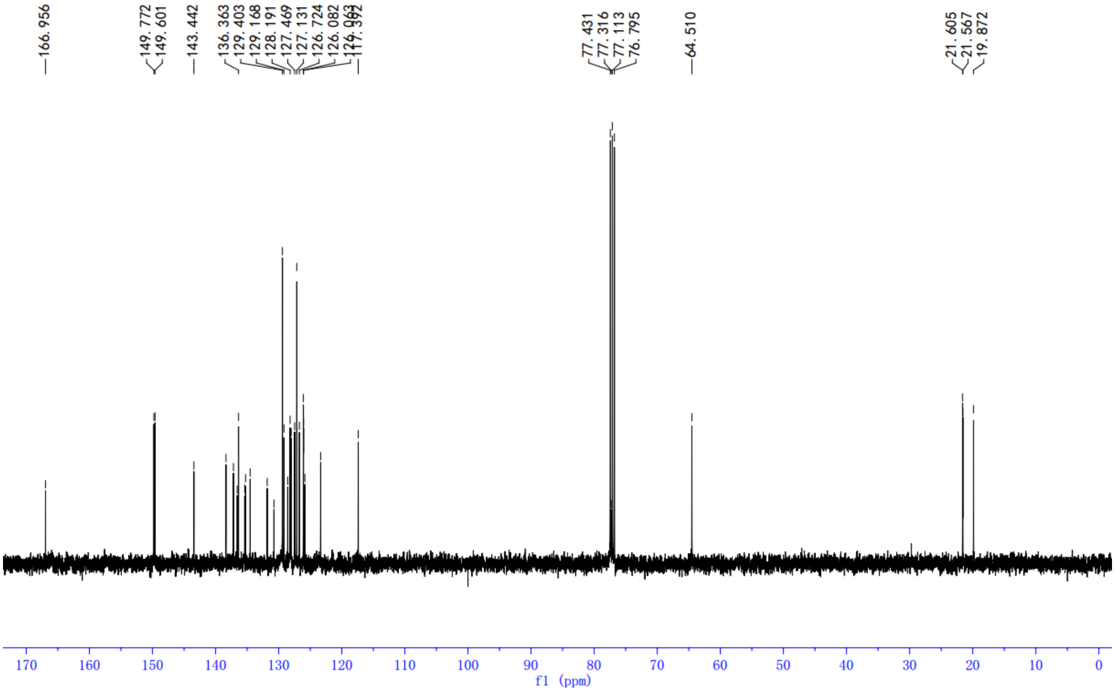

Supplementary Figure 63. ^{13}C NMR Spectrum of **3i**

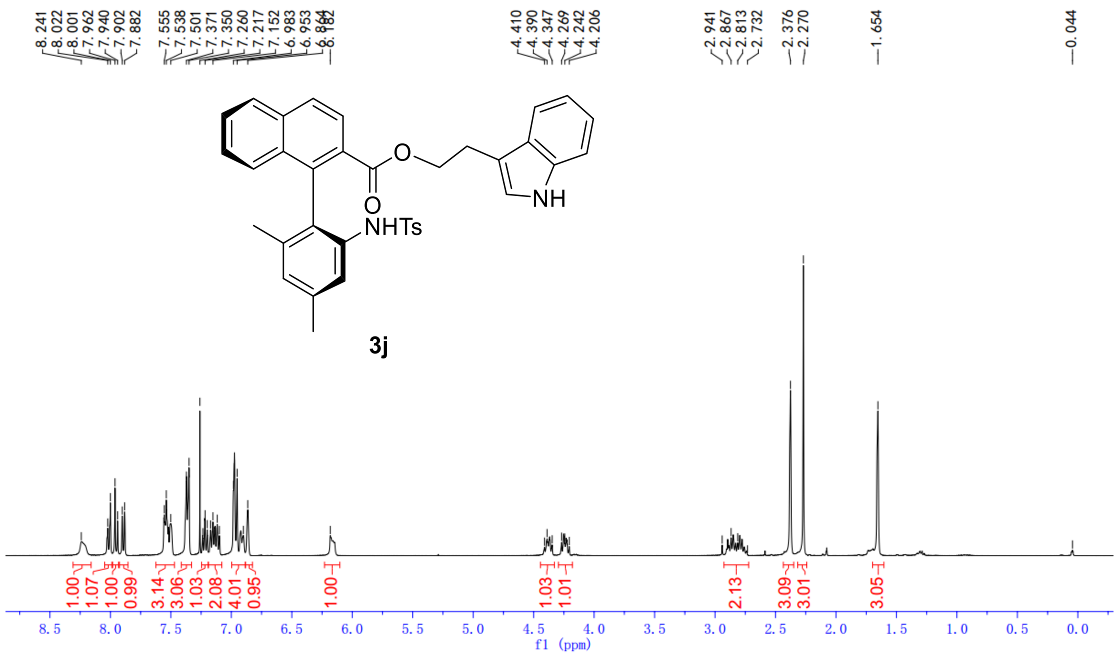

Supplementary Figure 64. ^{1}H NMR Spectrum of **3j**

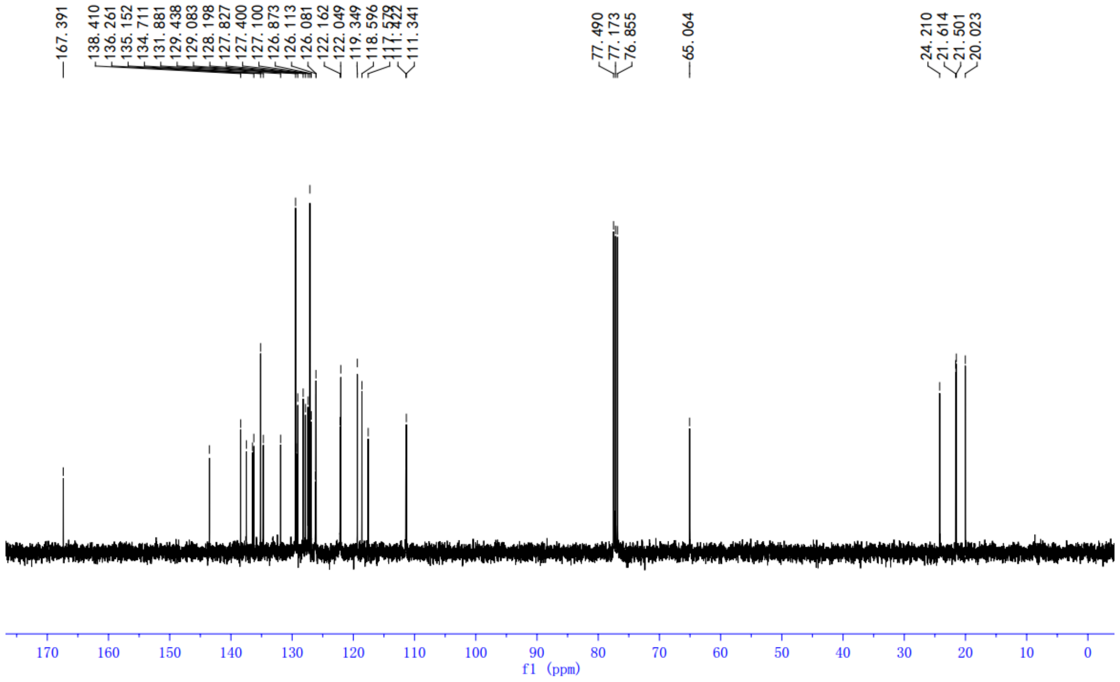

Supplementary Figure 65. ^{13}C NMR Spectrum of **3j**

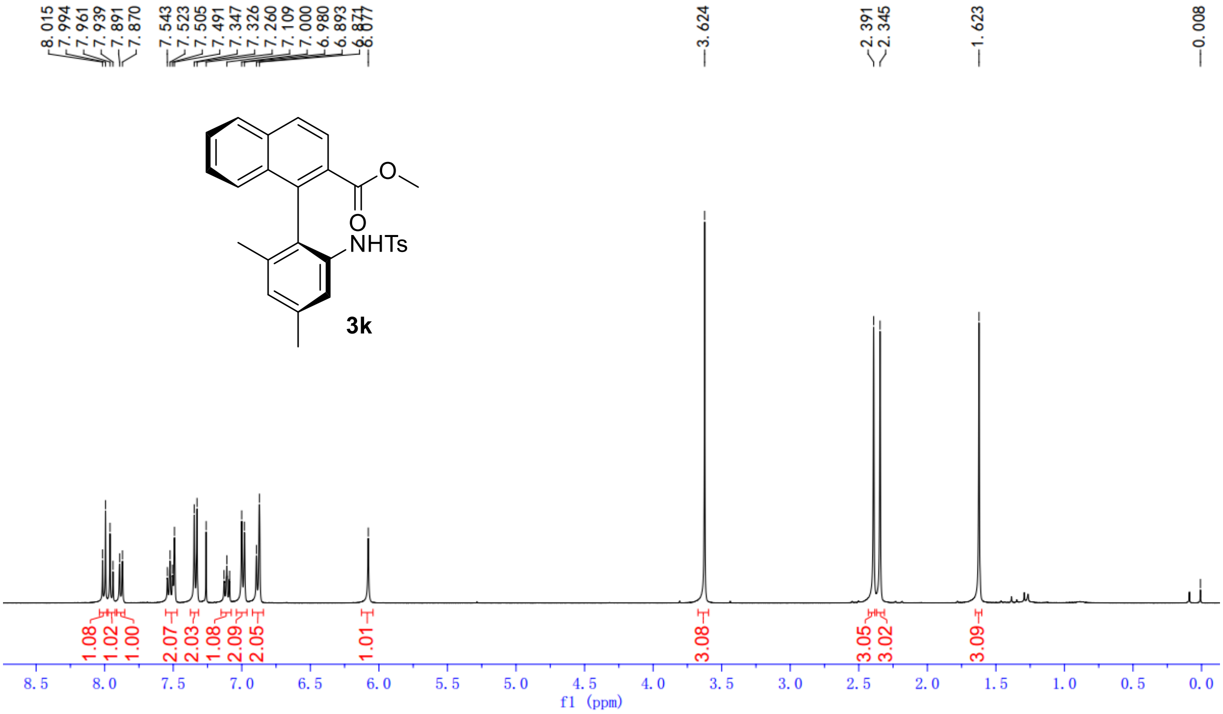

Supplementary Figure 66. ^{1}H NMR Spectrum of **3k**

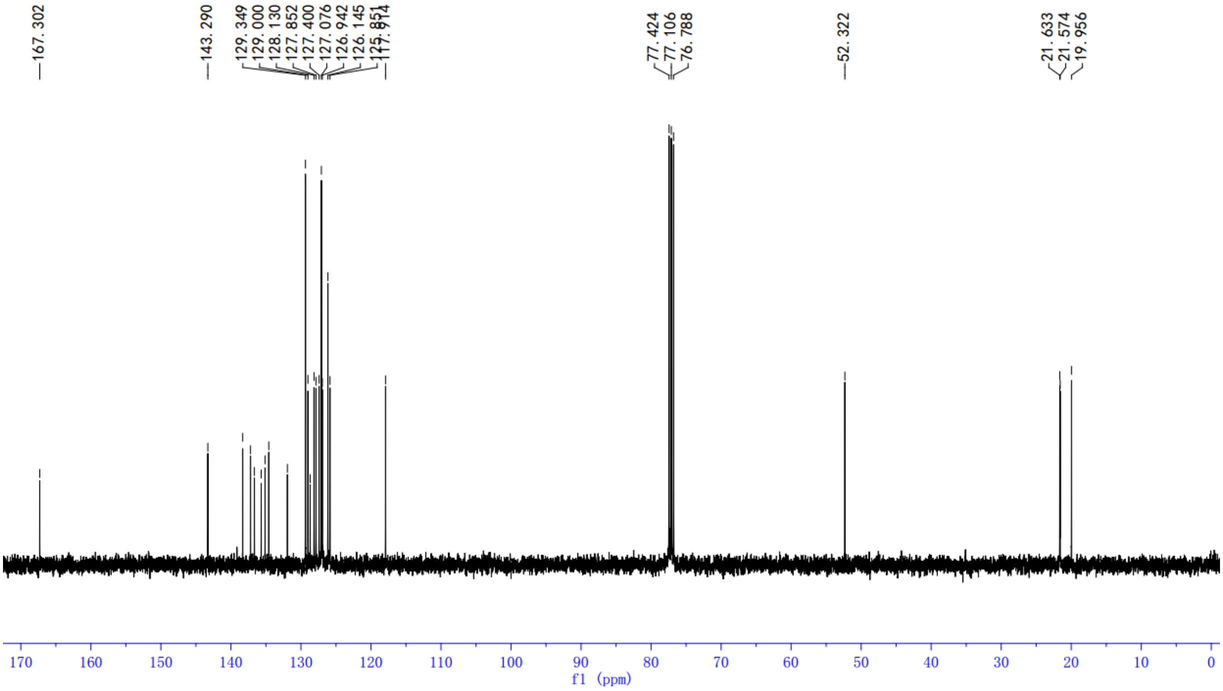

Supplementary Figure 67. ^{13}C NMR Spectrum of **3k**

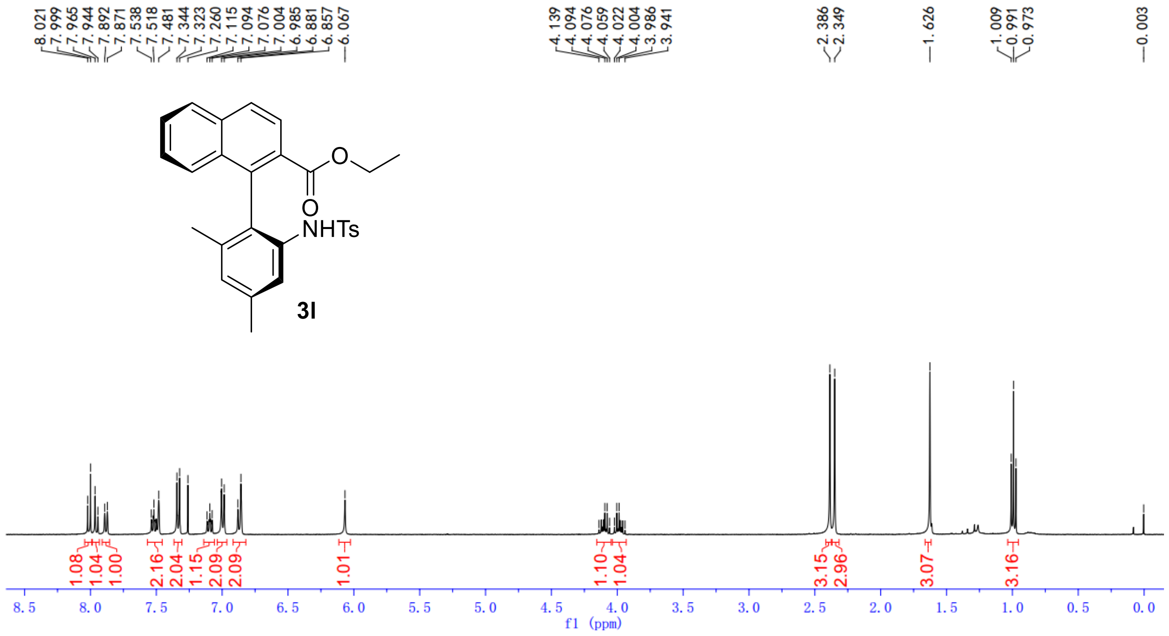

Supplementary Figure 68. ^{1}H NMR Spectrum of **3l**

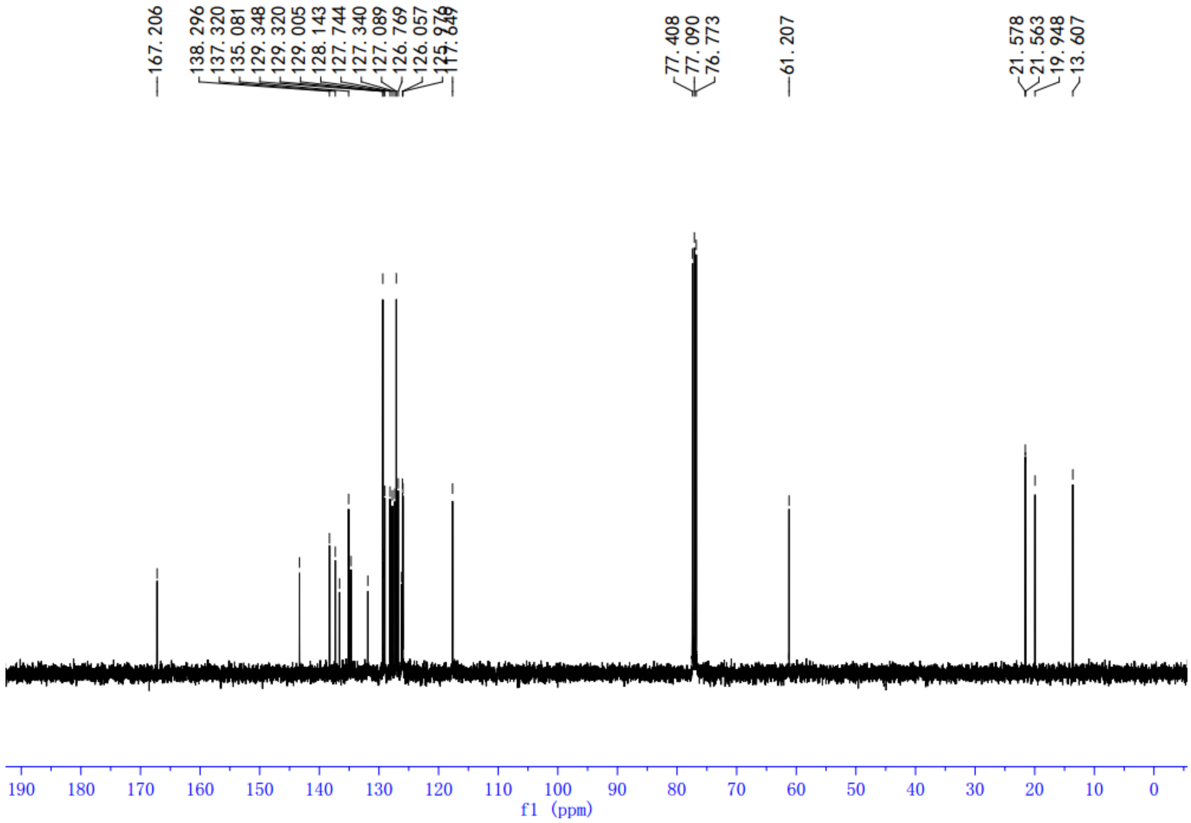

Supplementary Figure 69. ^{13}C NMR Spectrum of **3l**

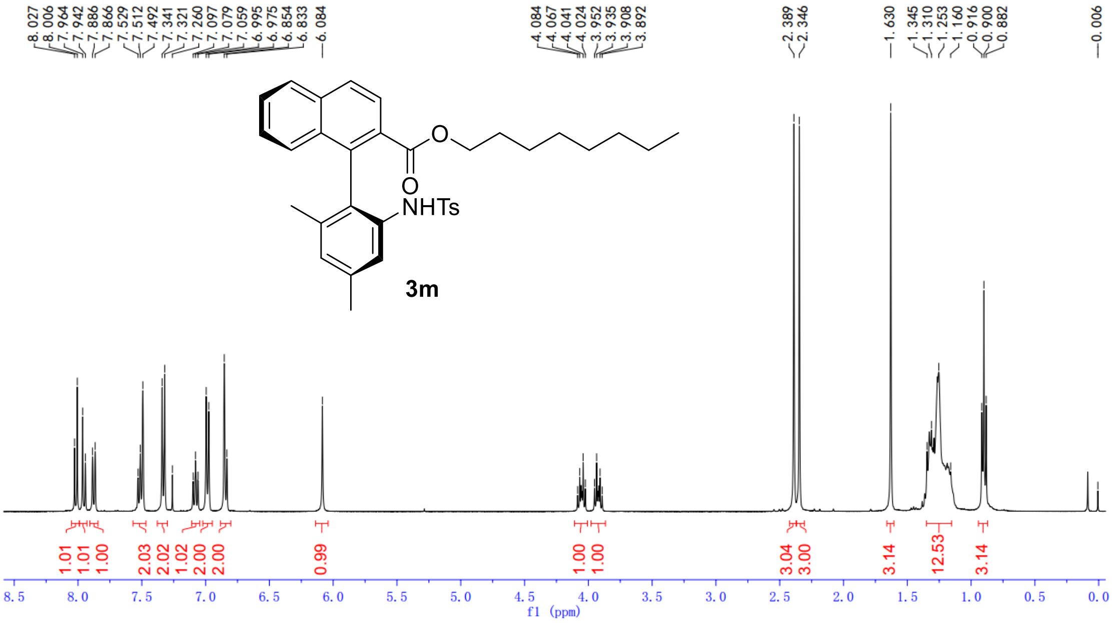

Supplementary Figure 70. ^{1}H NMR Spectrum of **3m**

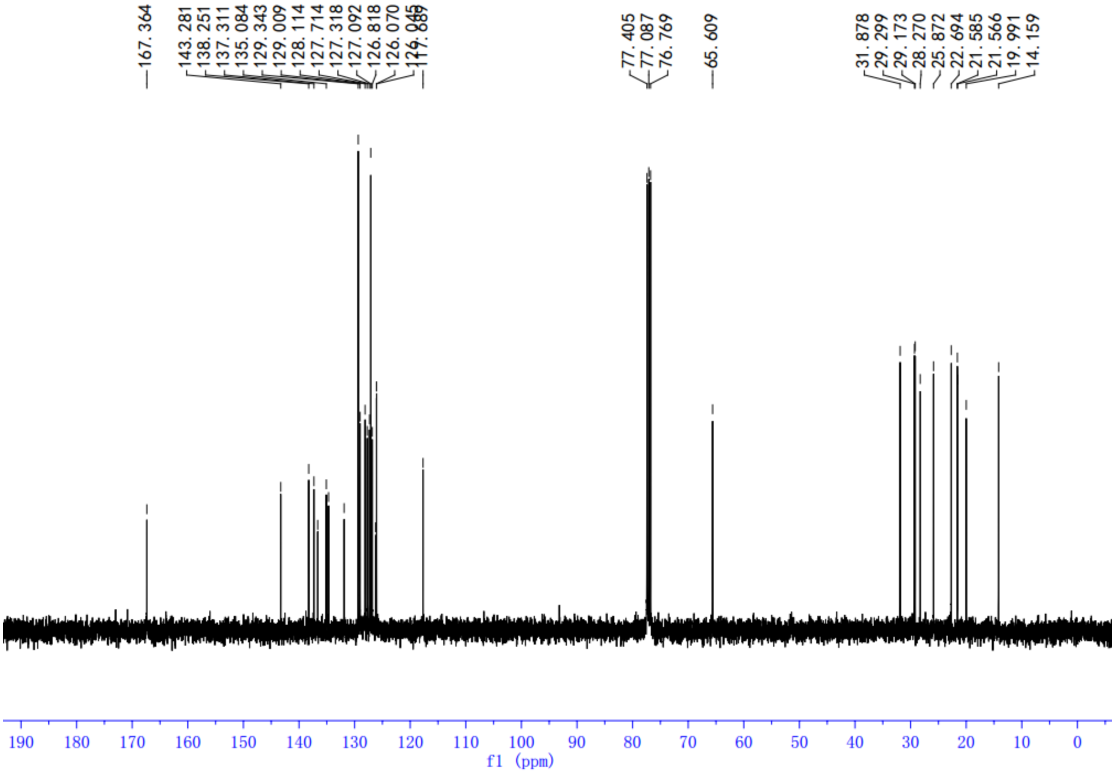

Supplementary Figure 71. ^{13}C NMR Spectrum of **3m**

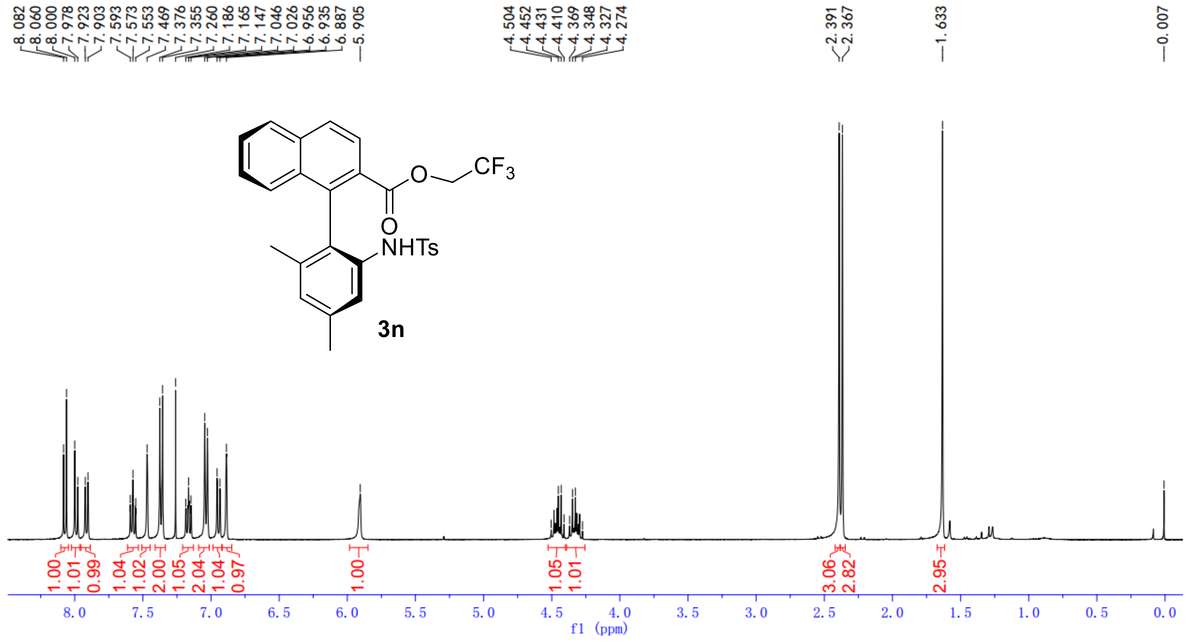

Supplementary Figure 72. ^{1}H NMR Spectrum of **3n**

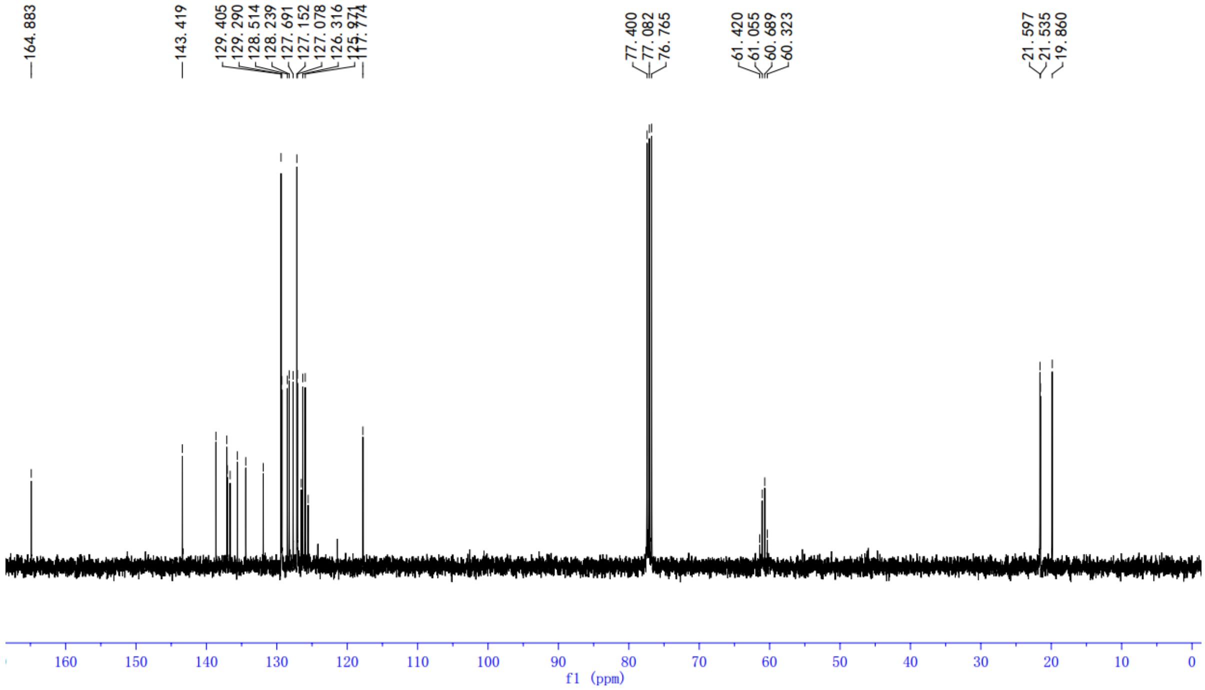

Supplementary Figure 73. ^{13}C NMR Spectrum of **3n**

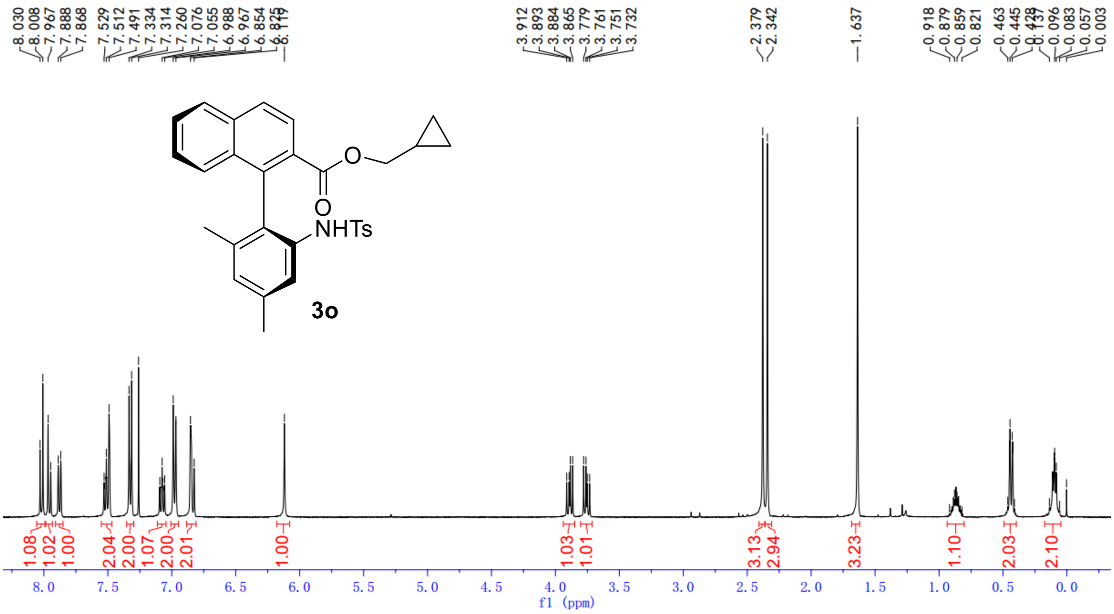

Supplementary Figure 74. ^{1}H NMR Spectrum of **3o**

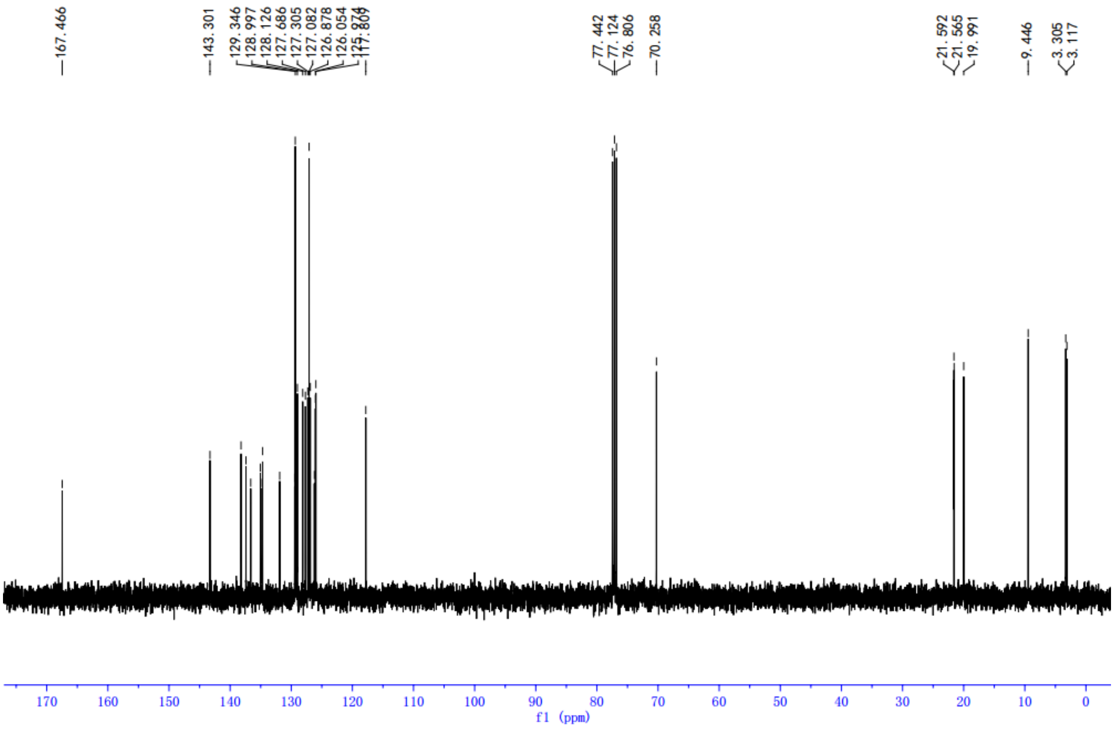

Supplementary Figure 75. ^{13}C NMR Spectrum of **3o**

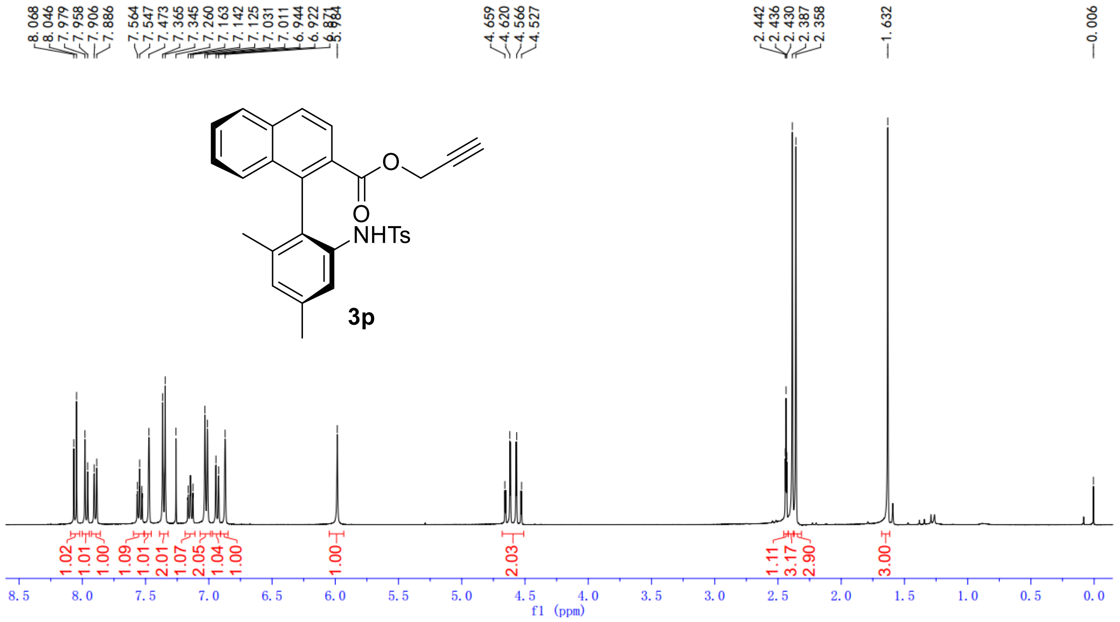

Supplementary Figure 76. ^{1}H NMR Spectrum of **3p**

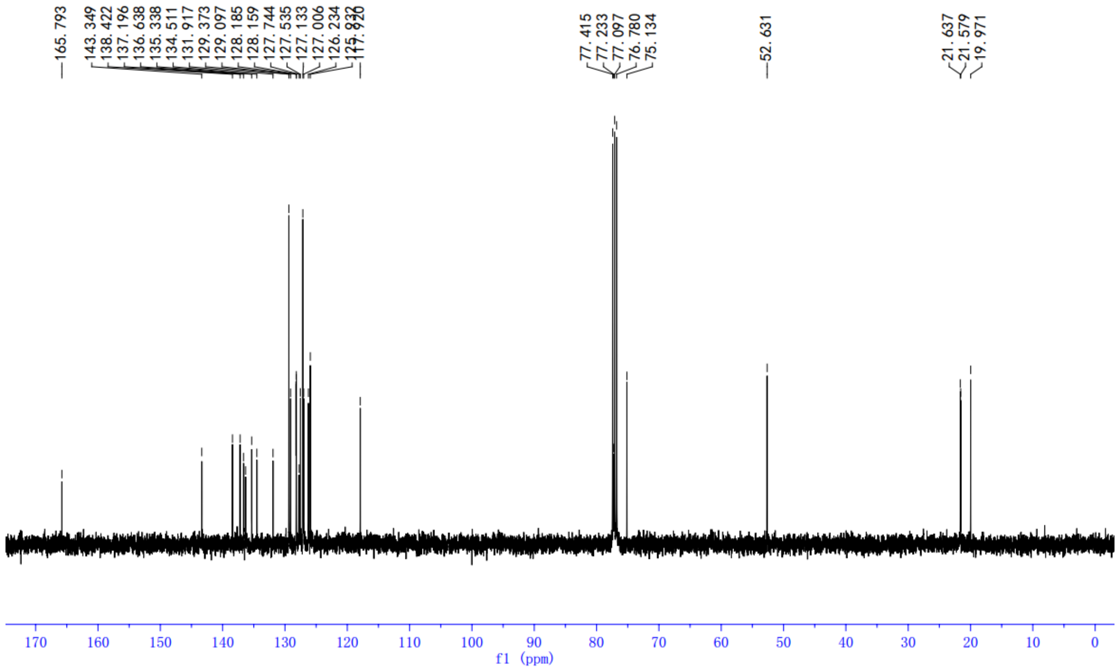

Supplementary Figure 77. ^{13}C NMR Spectrum of **3p**

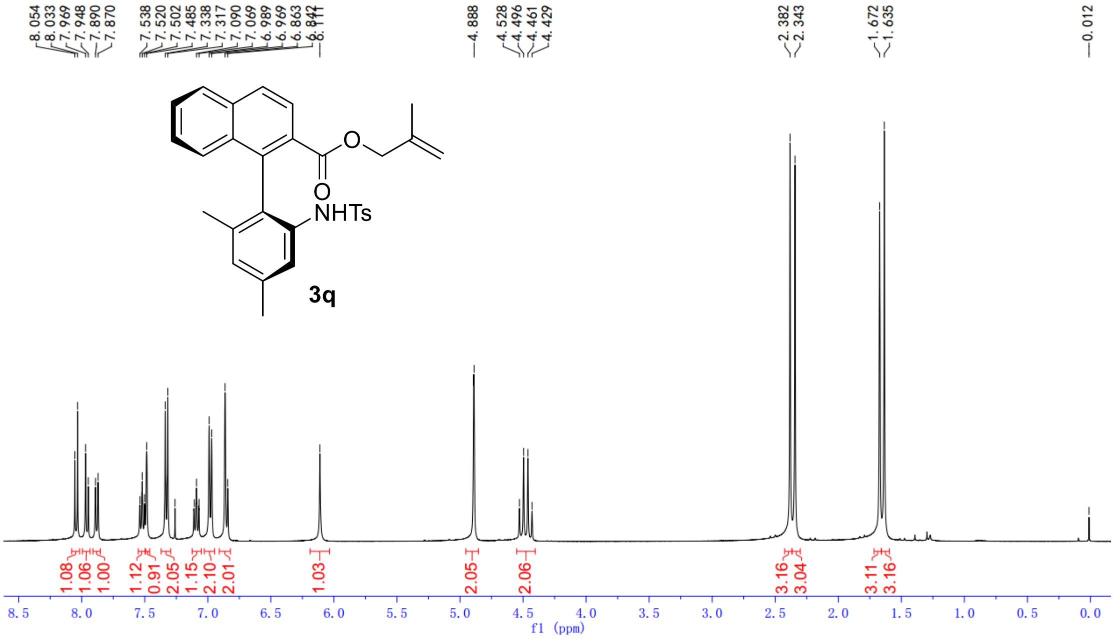

Supplementary Figure 78. ^{1}H NMR Spectrum of **3q**

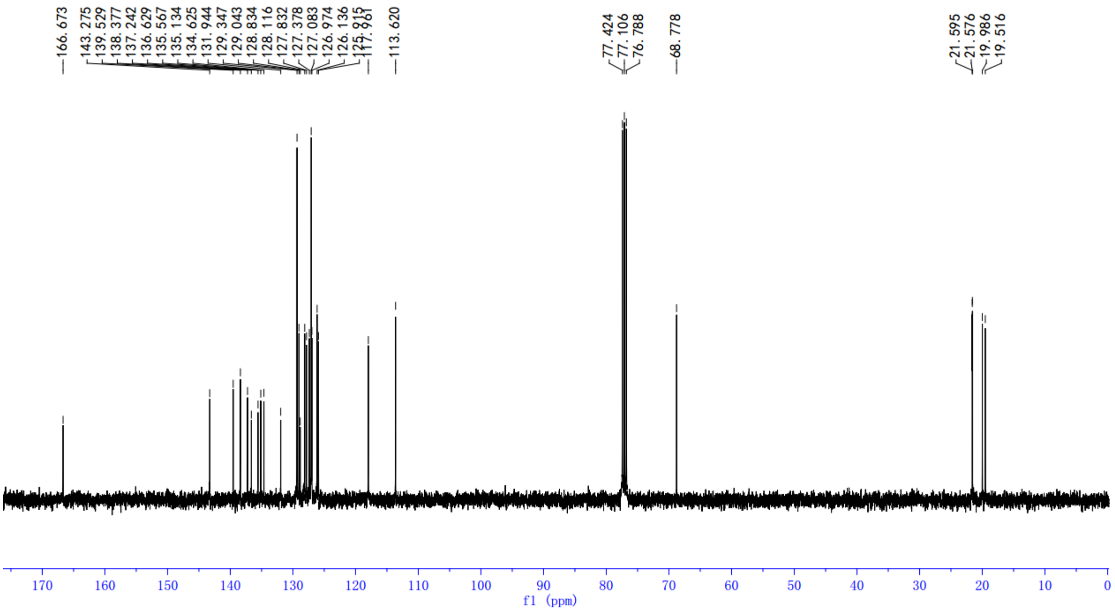

Supplementary Figure 79. ^{13}C NMR Spectrum of **3q**

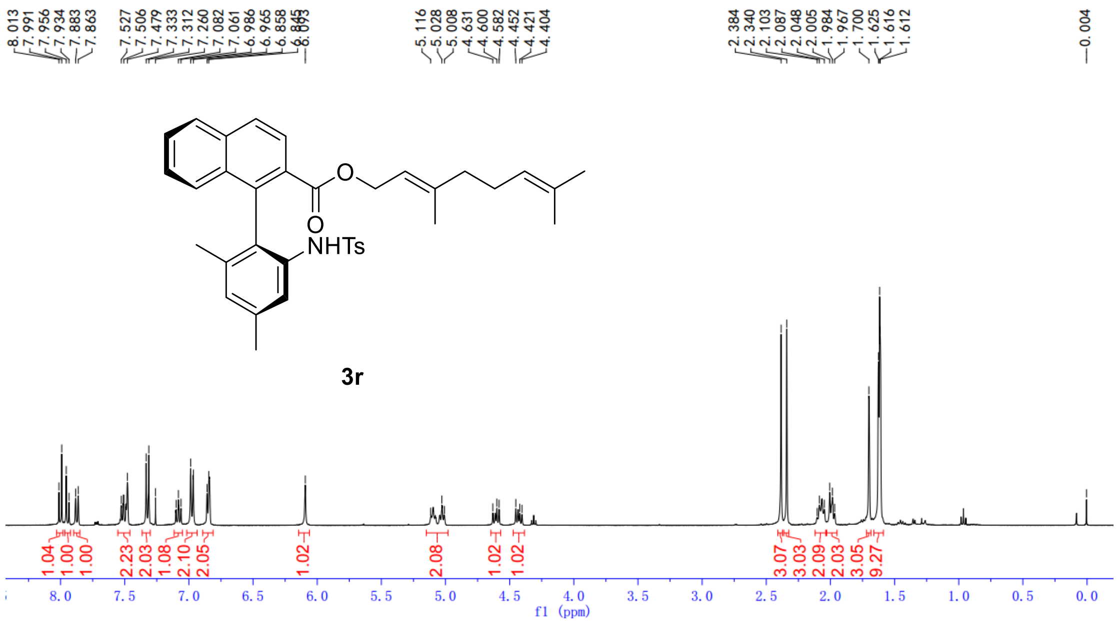

Supplementary Figure 80. ^{1}H NMR Spectrum of **3r**

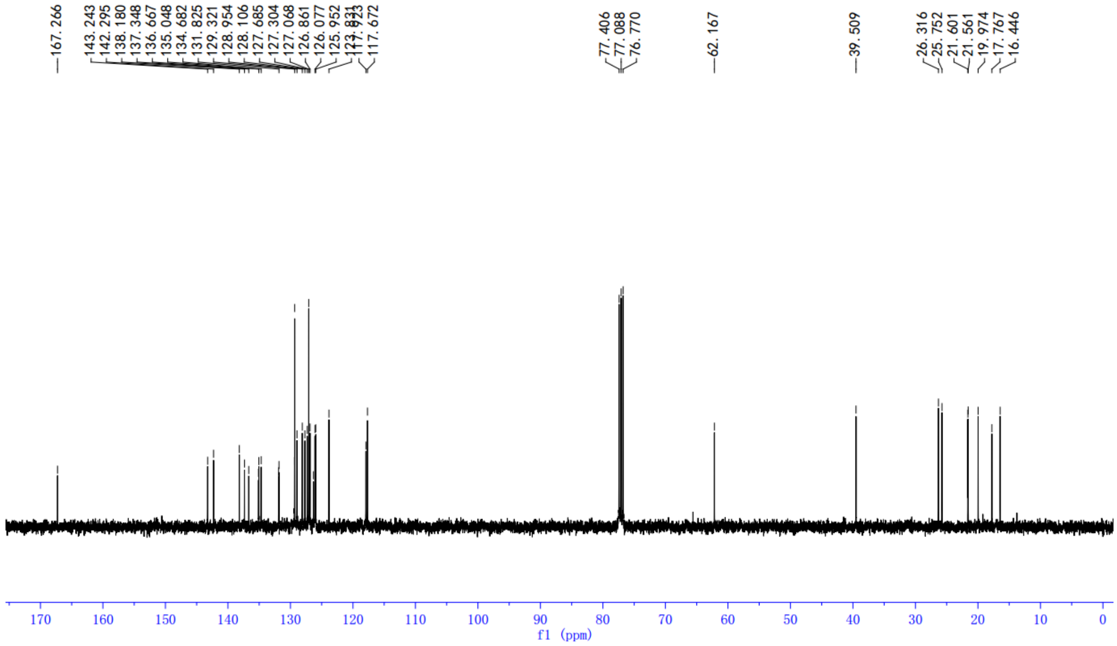

Supplementary Figure 81. ^{13}C NMR Spectrum of **3r**

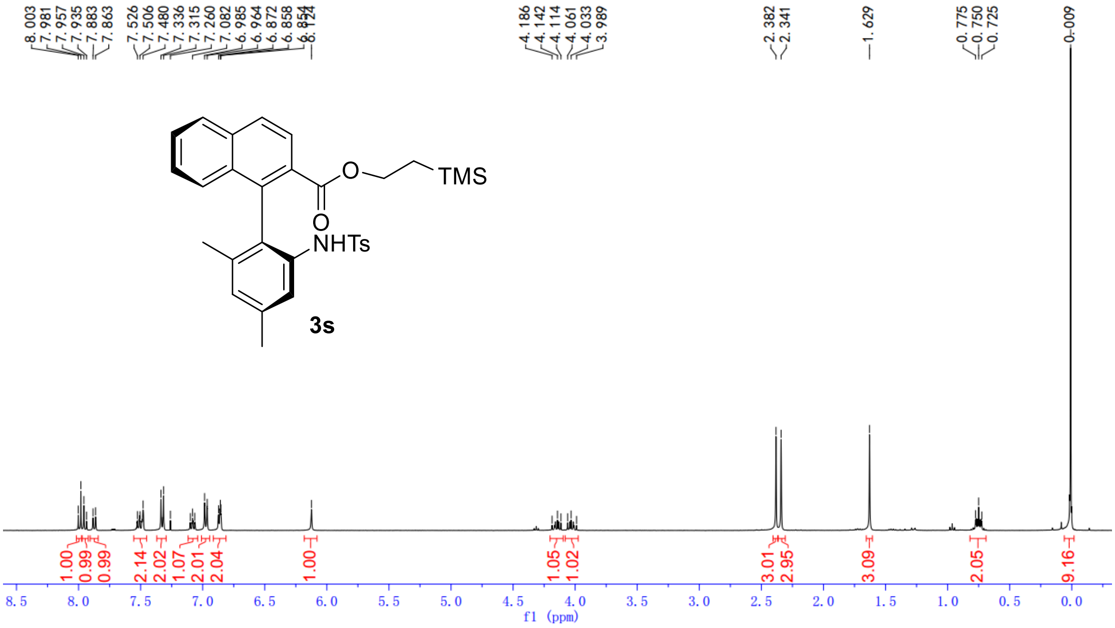

Supplementary Figure 82. ^{1}H NMR Spectrum of **3s**

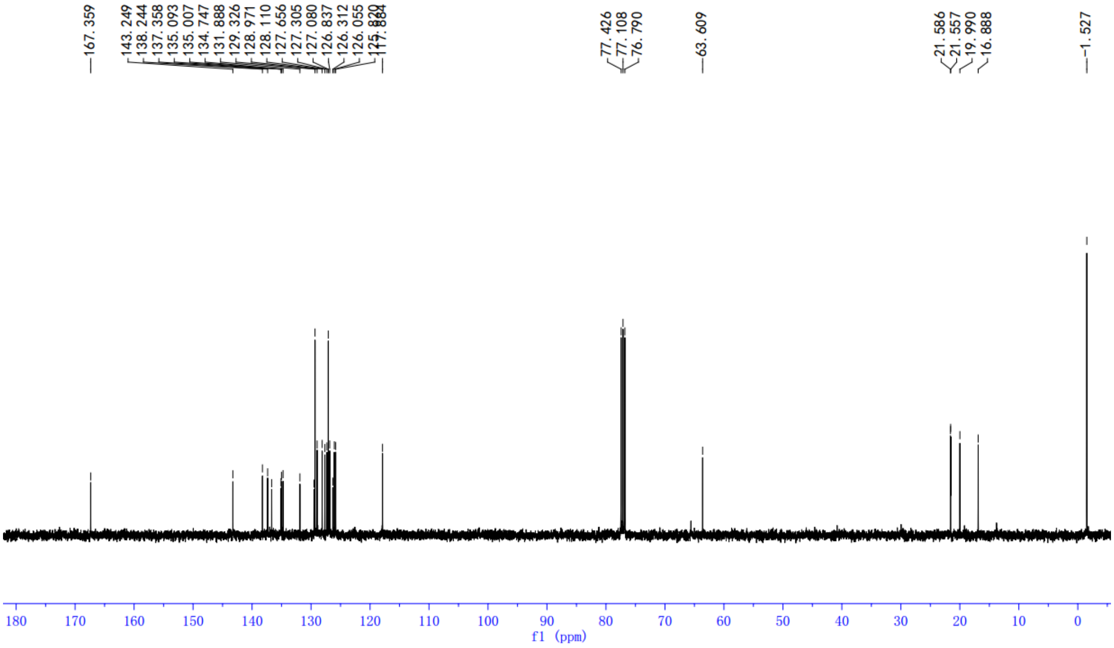

Supplementary Figure 83. ^{13}C NMR Spectrum of **3s**

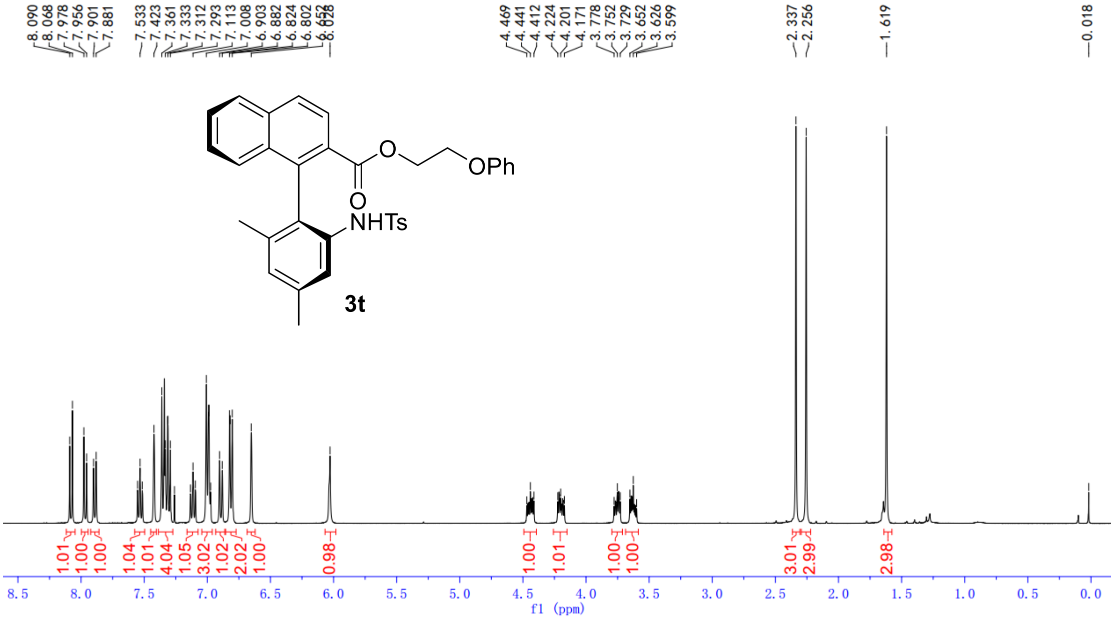

Supplementary Figure 84. ^{1}H NMR Spectrum of **3t**

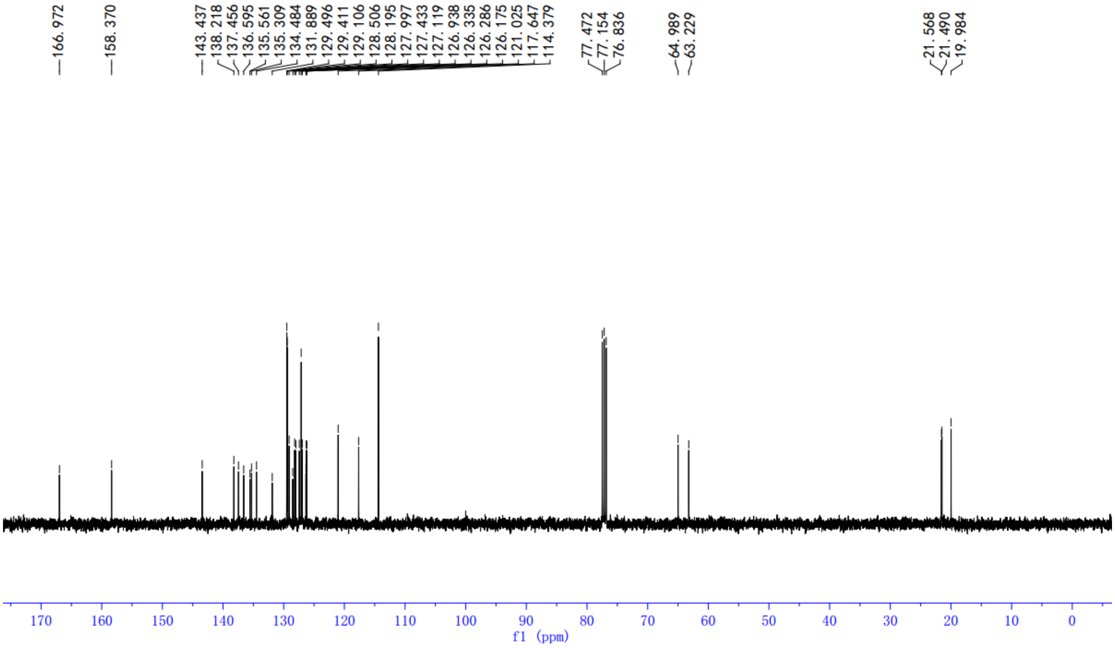

Supplementary Figure 85. ^{13}C NMR Spectrum of **3t**

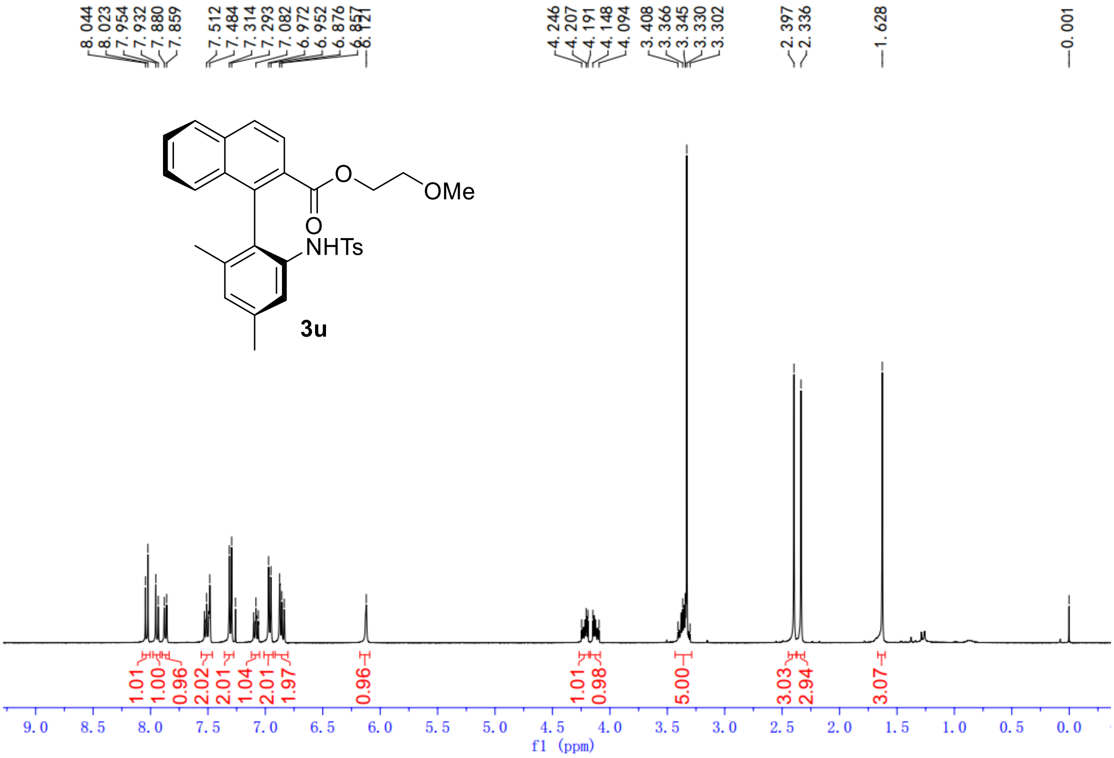

Supplementary Figure 86. ^{1}H NMR Spectrum of **3u**

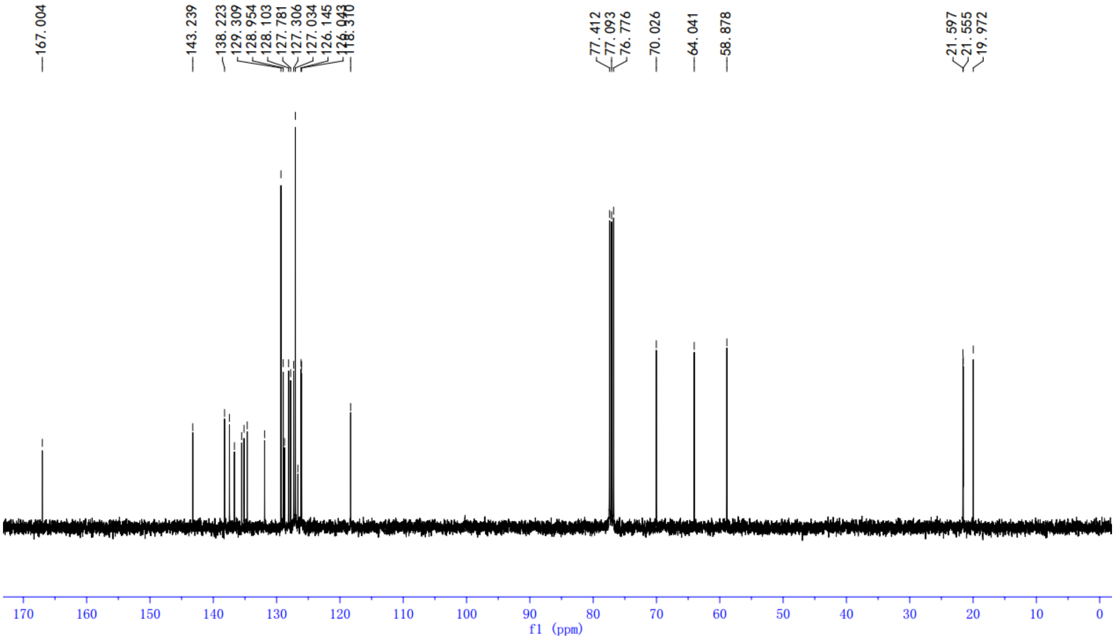

Supplementary Figure 87. ^{13}C NMR Spectrum of **3u**

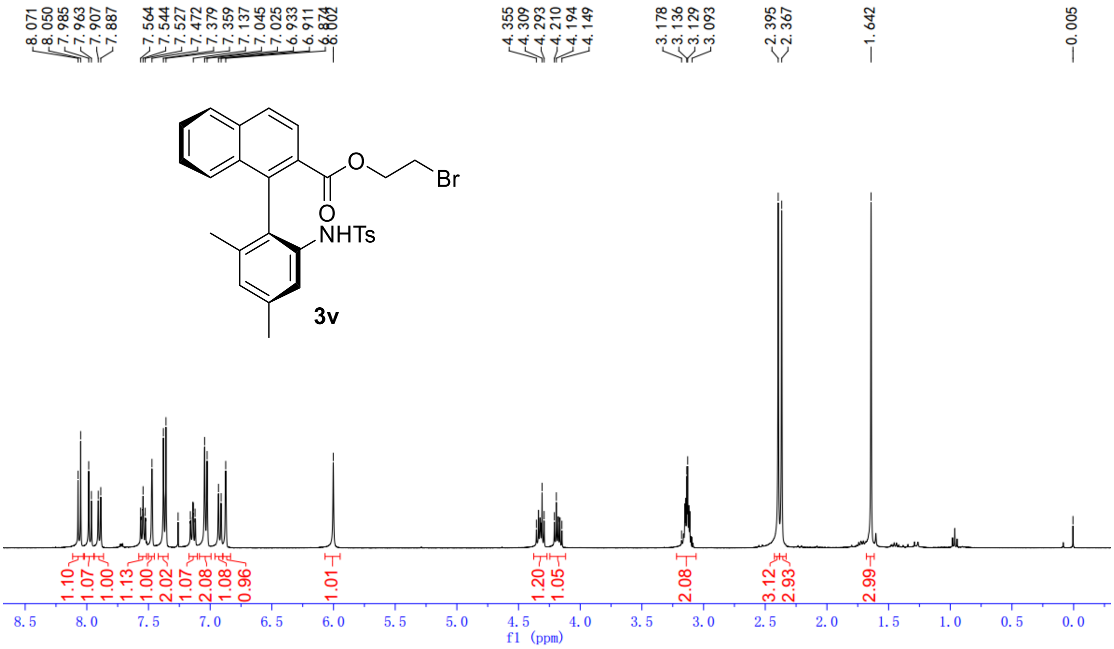

Supplementary Figure 88. ^{1}H NMR Spectrum of **3v**

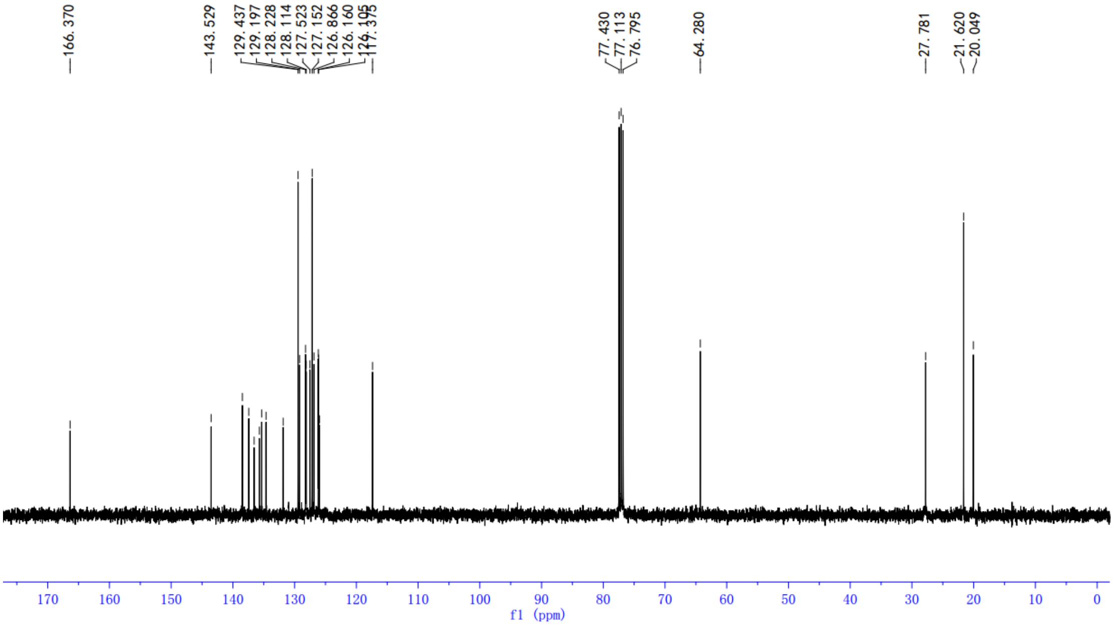

Supplementary Figure 89. ^{13}C NMR Spectrum of **3v**

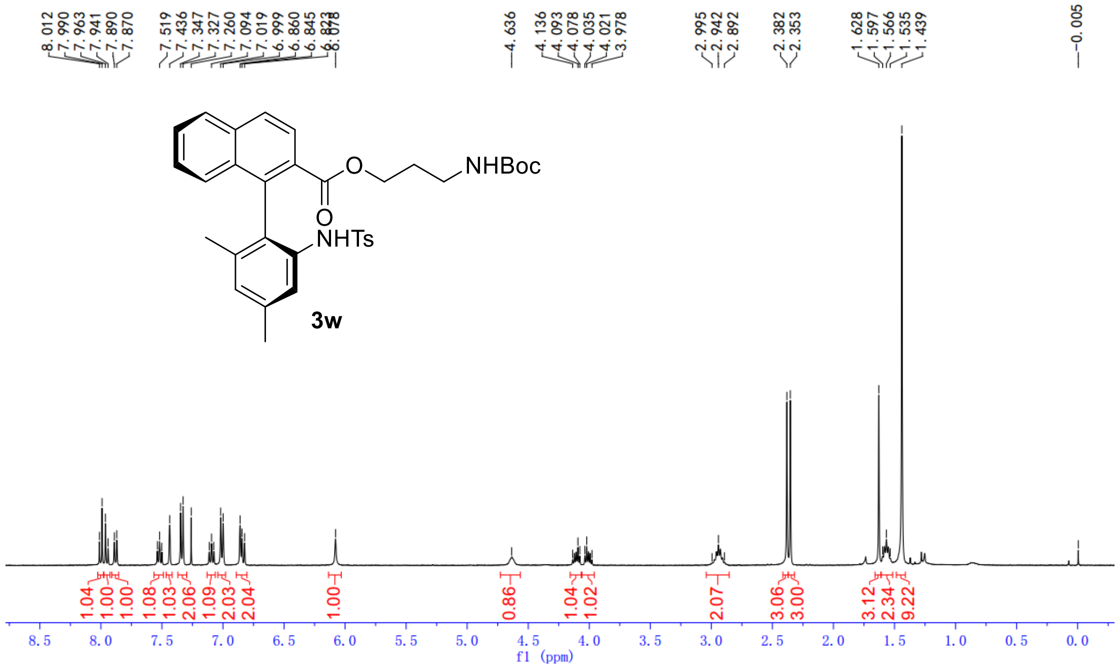

Supplementary Figure 90. ^{1}H NMR Spectrum of **3w**

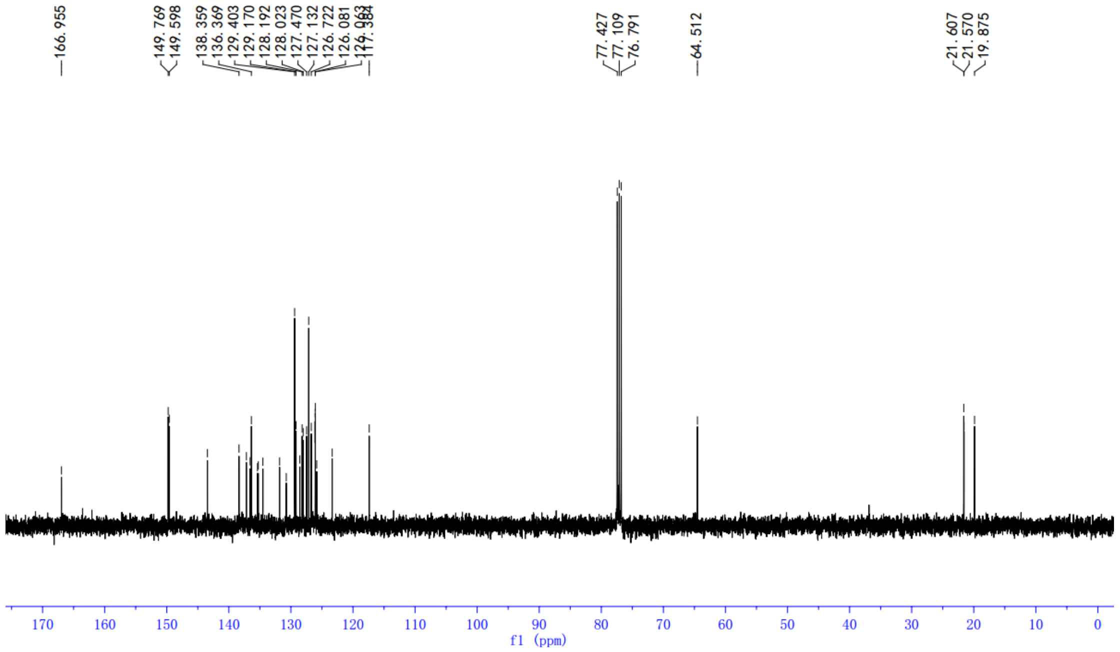

Supplementary Figure 91. ^{13}C NMR Spectrum of **3w**

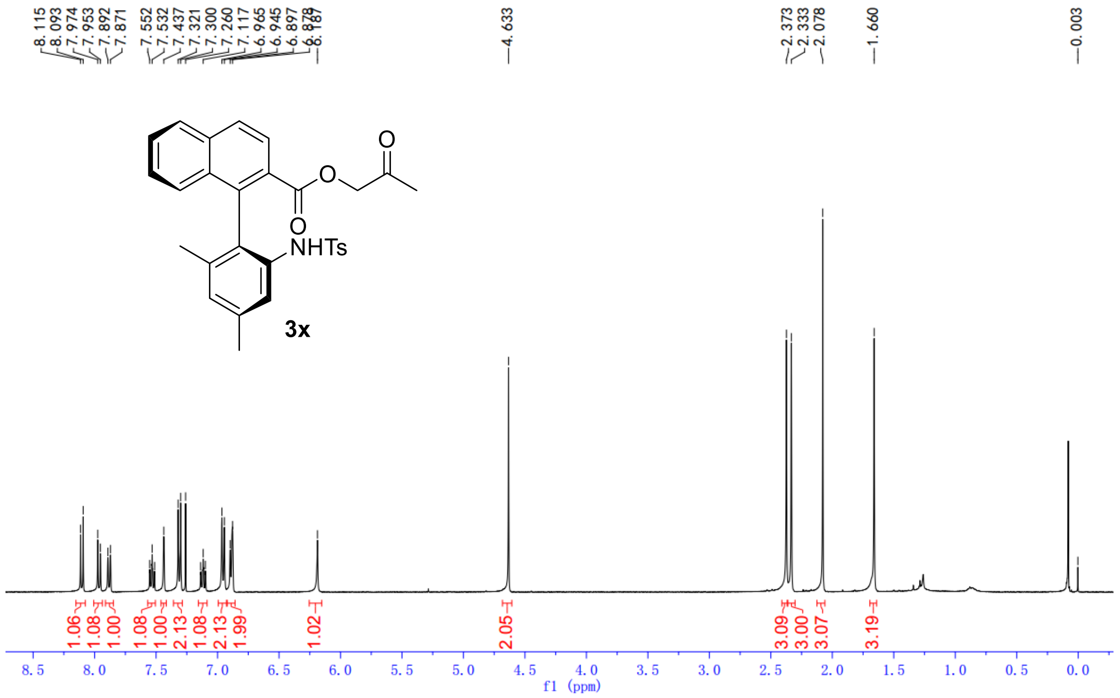

Supplementary Figure 92. ^{1}H NMR Spectrum of **3x**

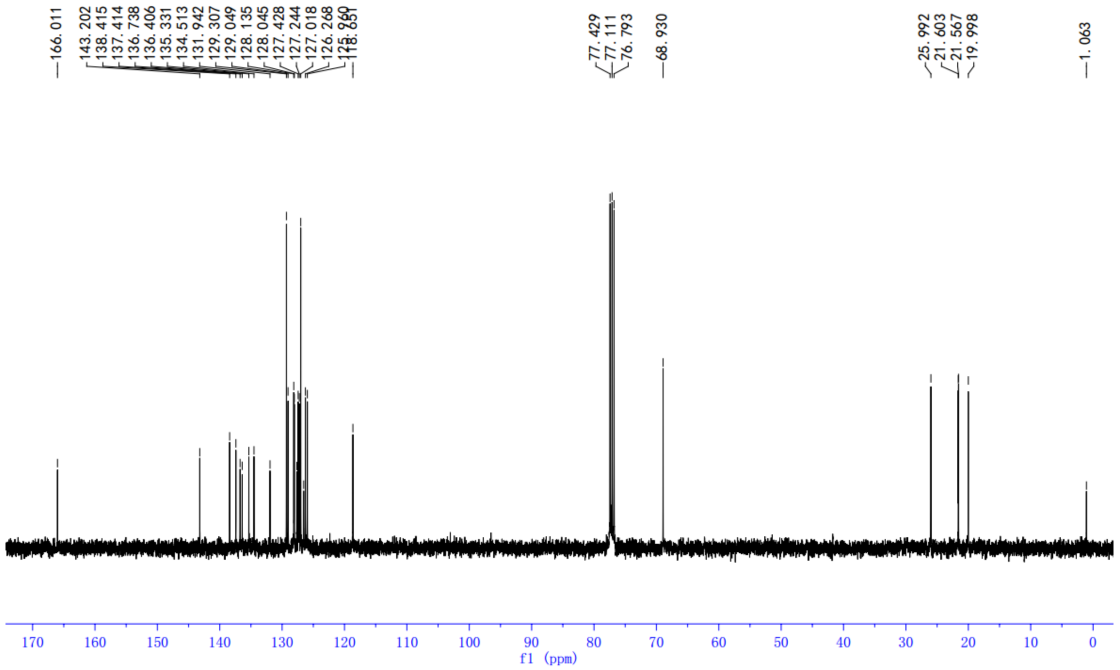

Supplementary Figure 93. ^{13}C NMR Spectrum of **3x**

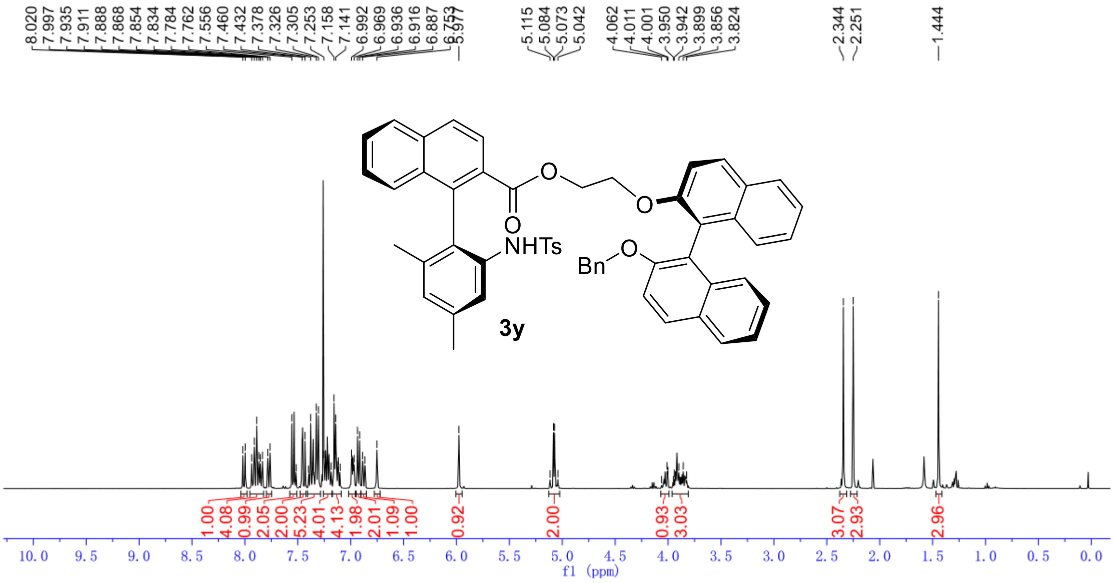

Supplementary Figure 94. ^{1}H NMR Spectrum of **3y**

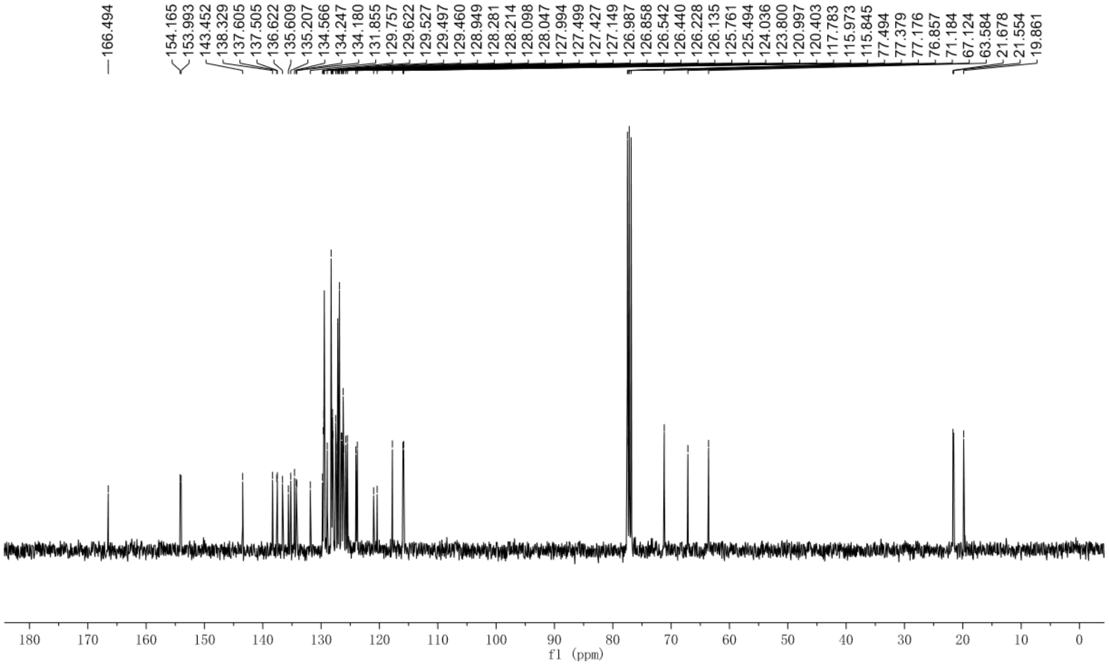

Supplementary Figure 95. ^{13}C NMR Spectrum of **3y**

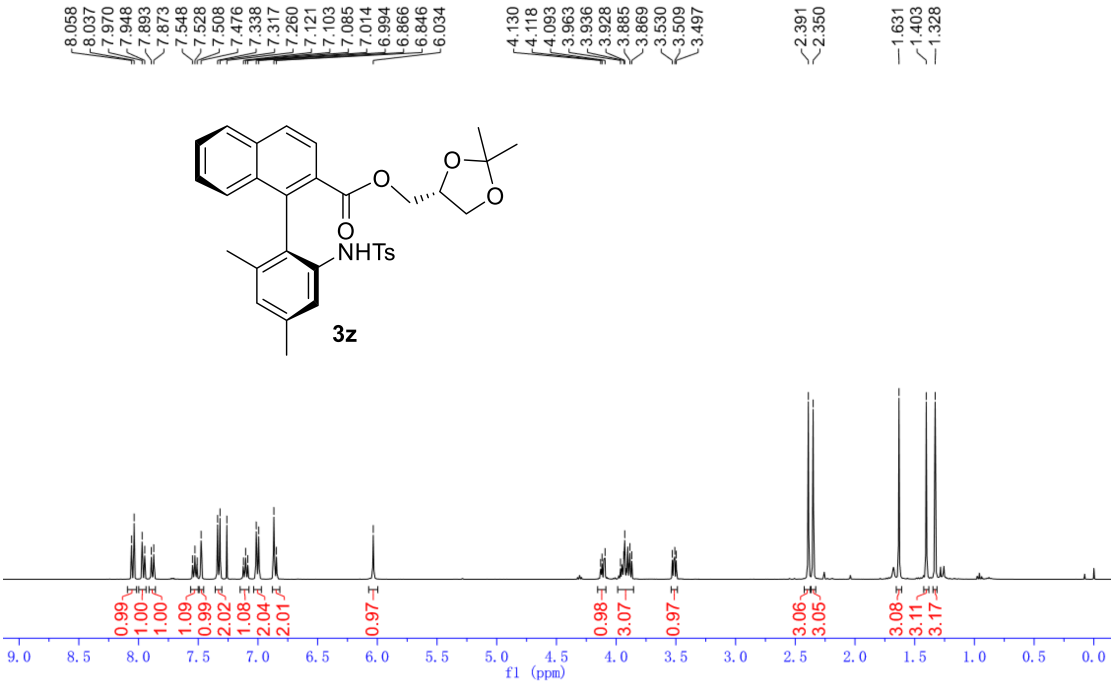

Supplementary Figure 96. ^{1}H NMR Spectrum of **3z**

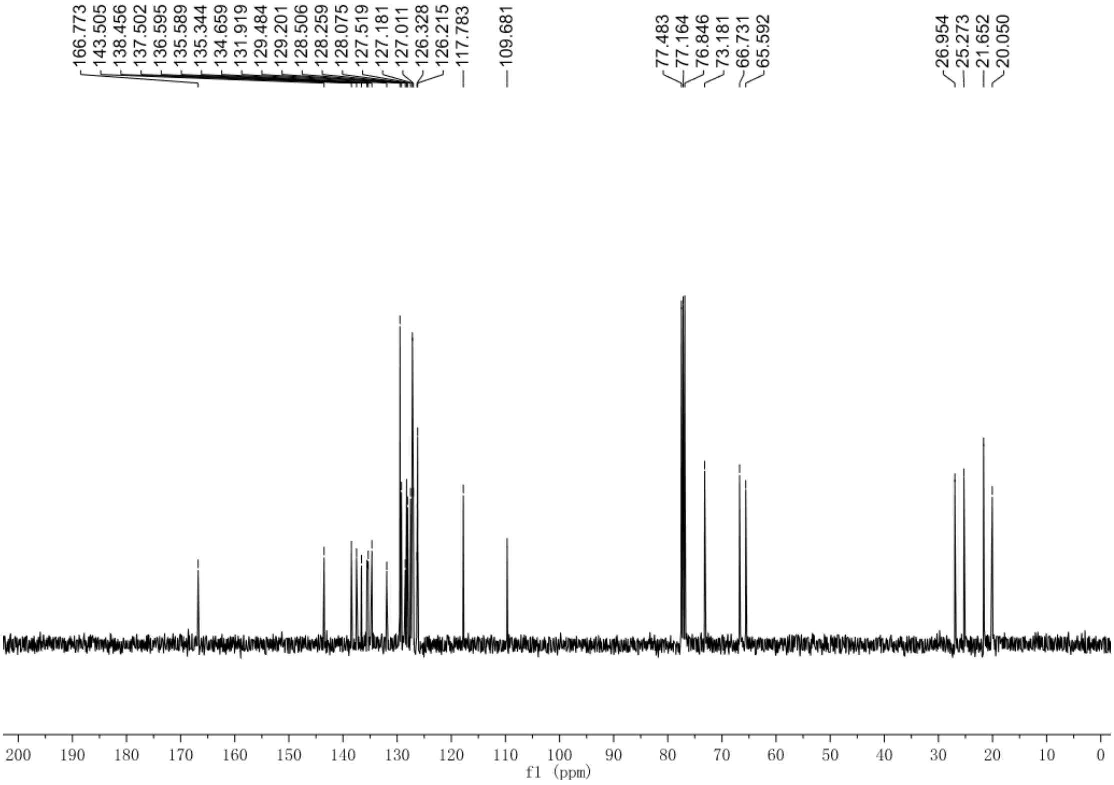

Supplementary Figure 97. ^{13}C NMR Spectrum of **3z**

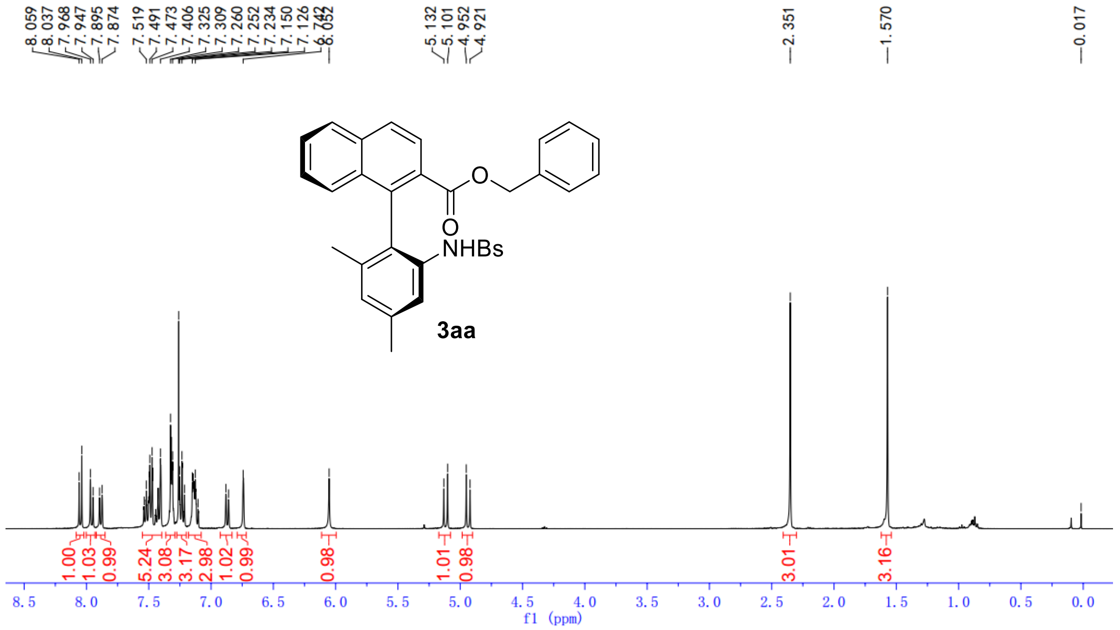

Supplementary Figure 98. ^{1}H NMR Spectrum of **3aa**

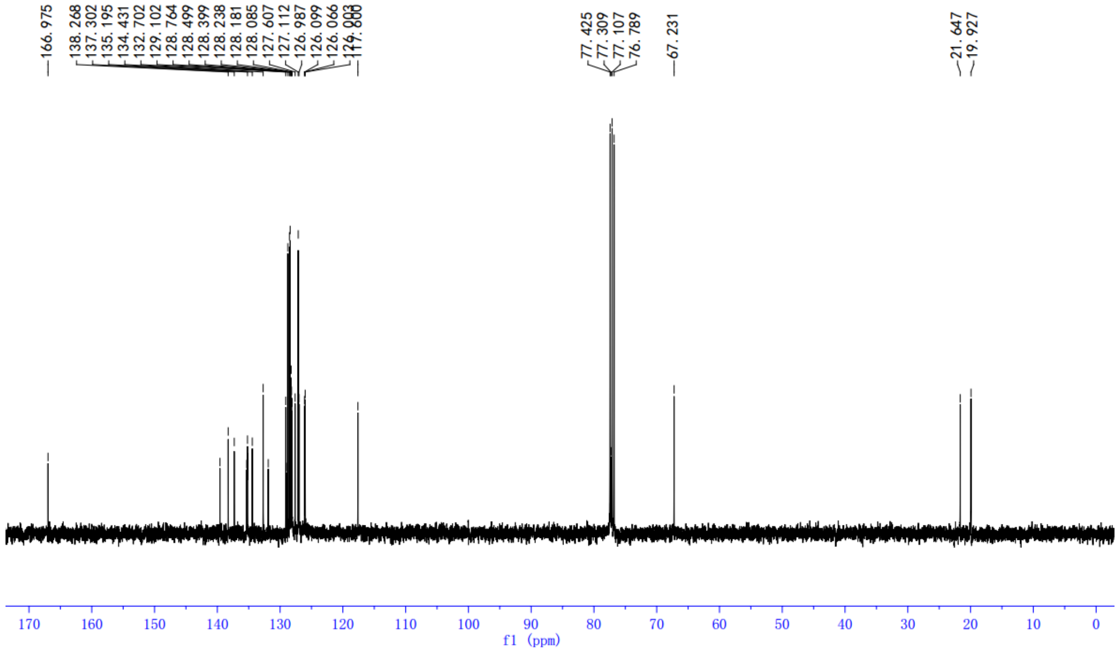

Supplementary Figure 99. ^{13}C NMR Spectrum of **3aa**

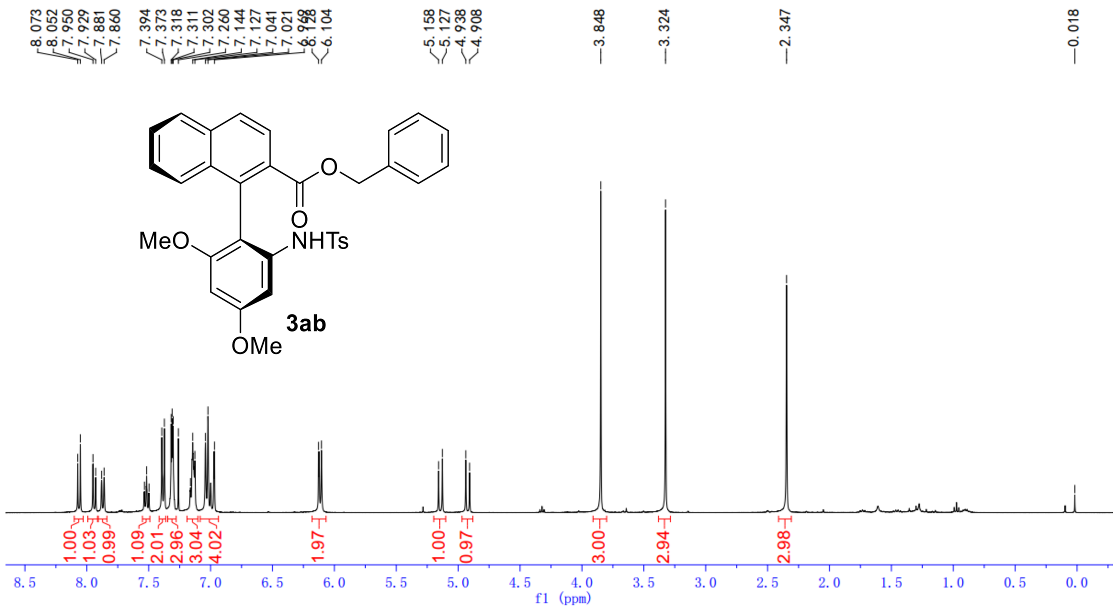

Supplementary Figure 100. ^{1}H NMR Spectrum of **3ab**

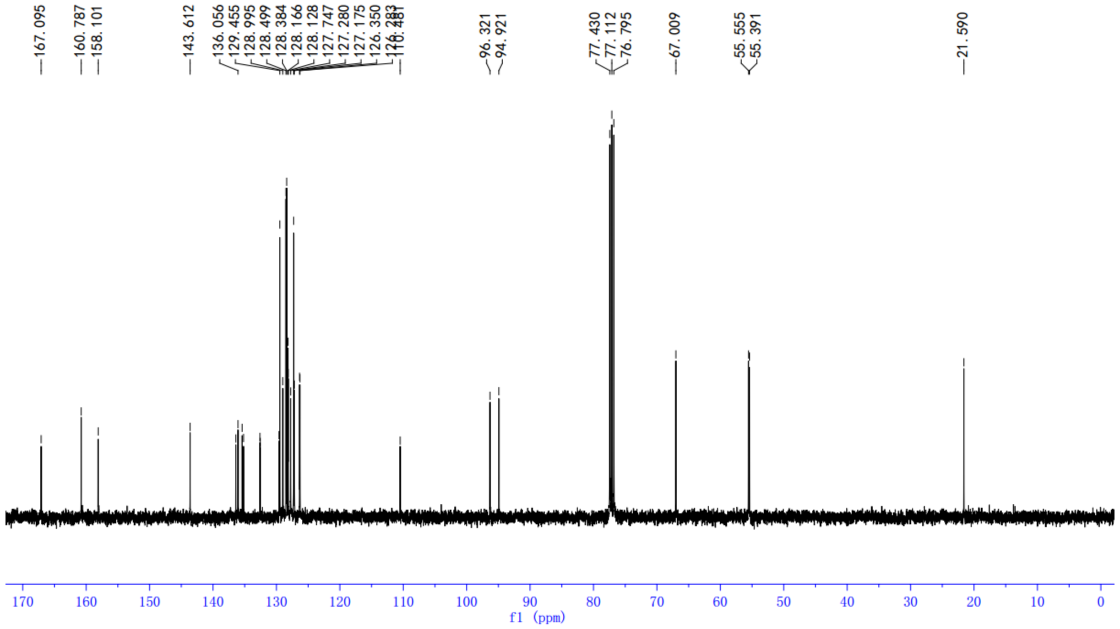

Supplementary Figure 101. ^{13}C NMR Spectrum of **3ab**

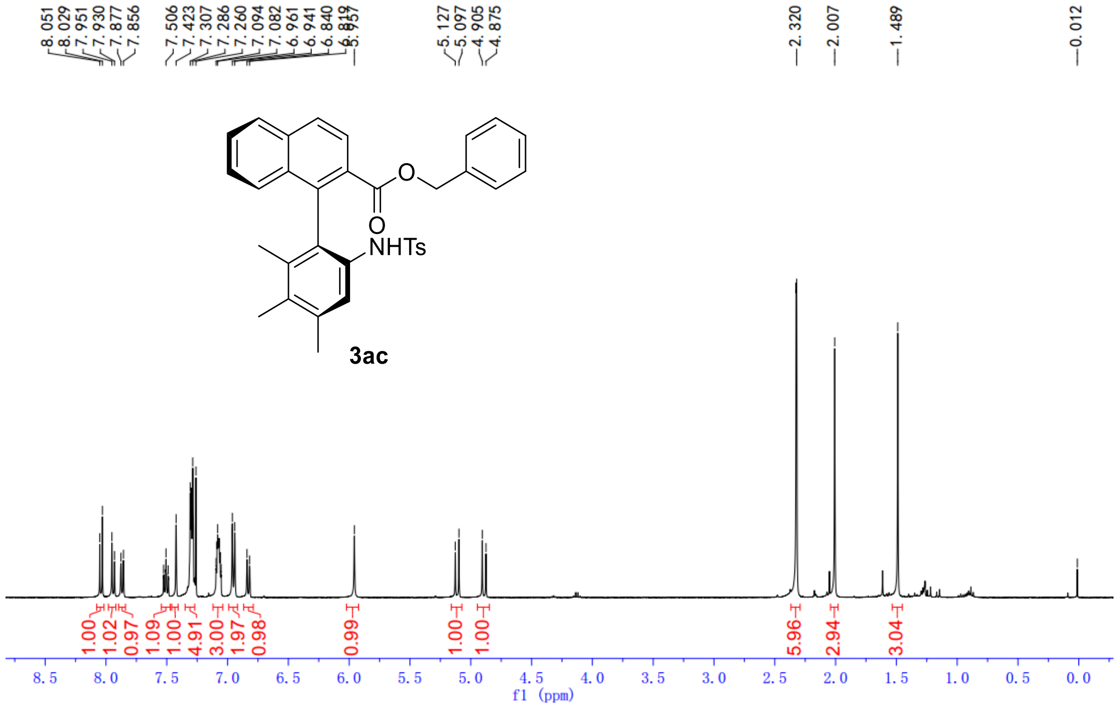

Supplementary Figure 102. ^{1}H NMR Spectrum of **3ac**

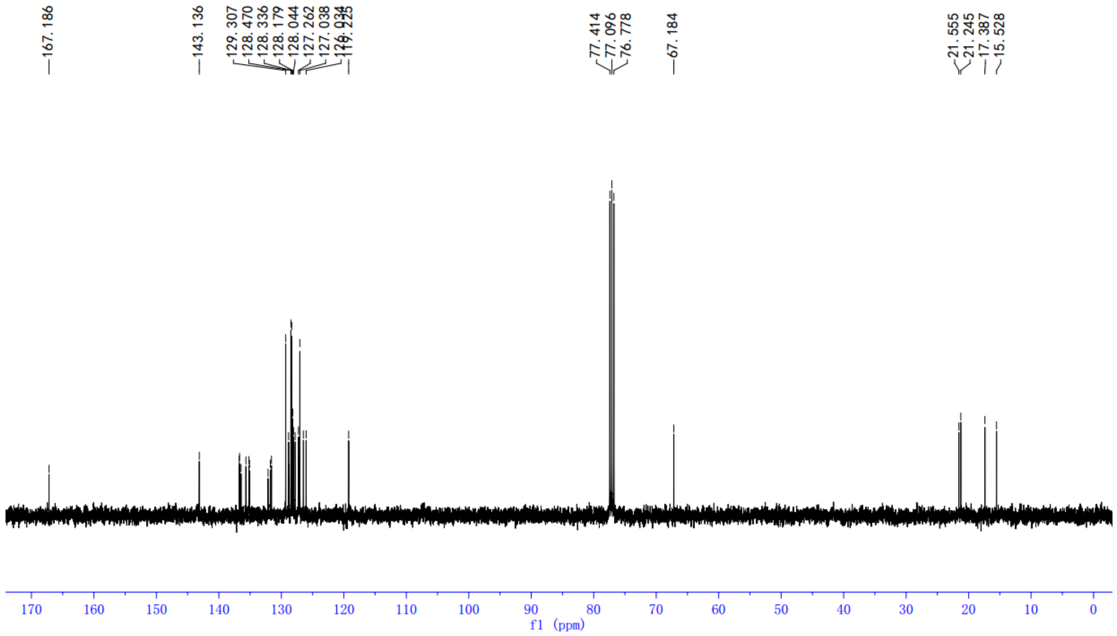

Supplementary Figure 103. ^{13}C NMR Spectrum of **3ac**

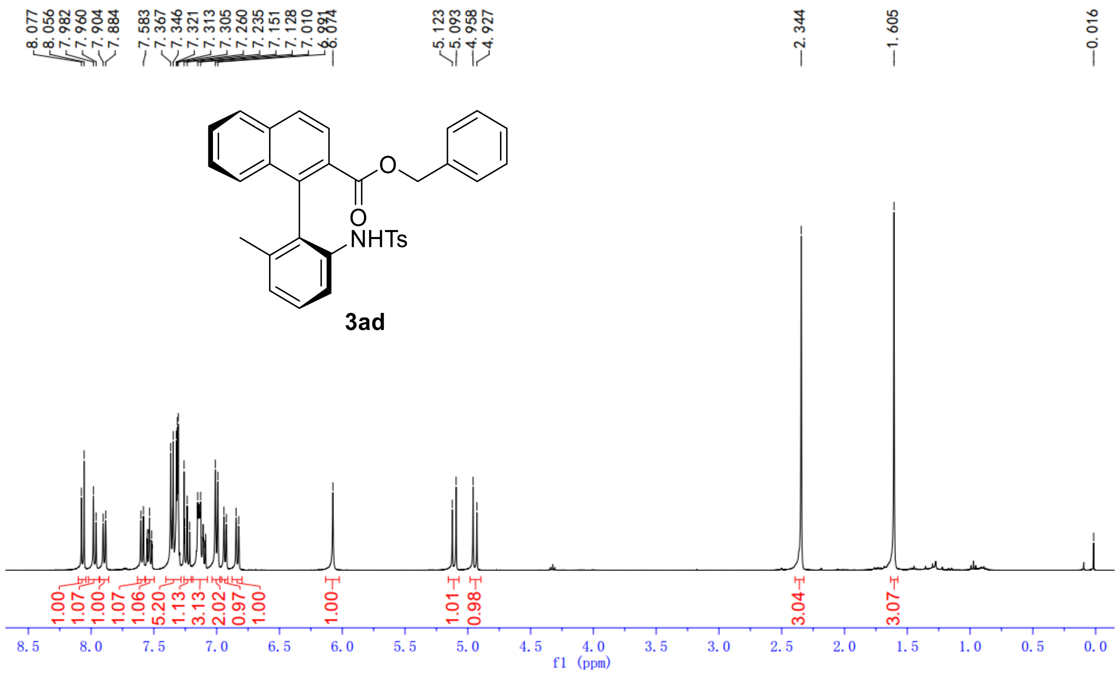

Supplementary Figure 104. ^{1}H NMR Spectrum of **3ad**

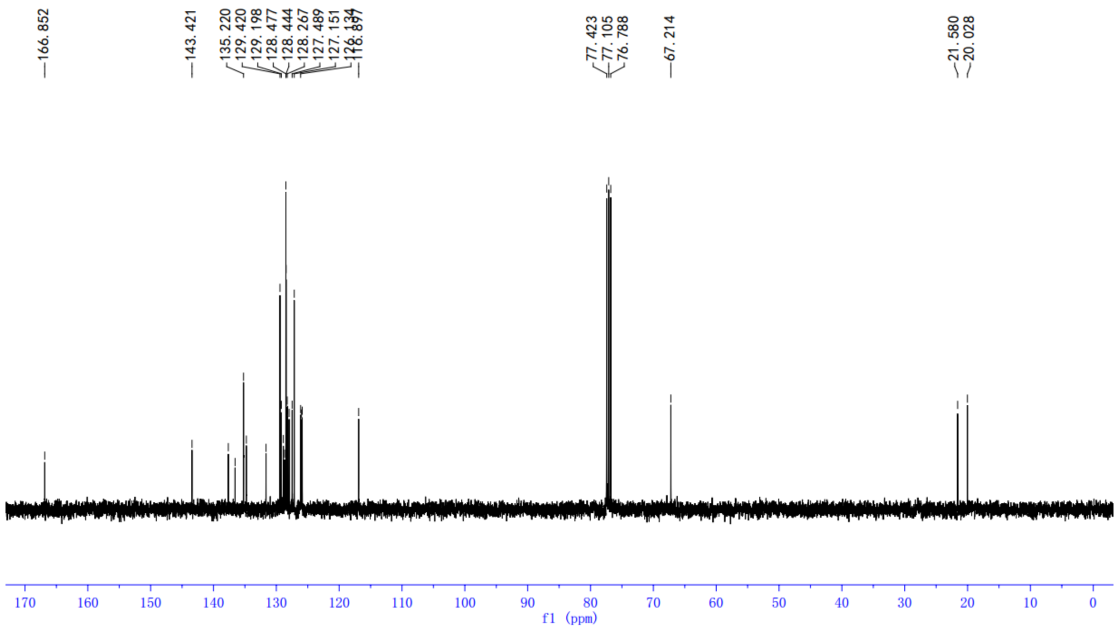

Supplementary Figure 105. ^{13}C NMR Spectrum of **3ad**

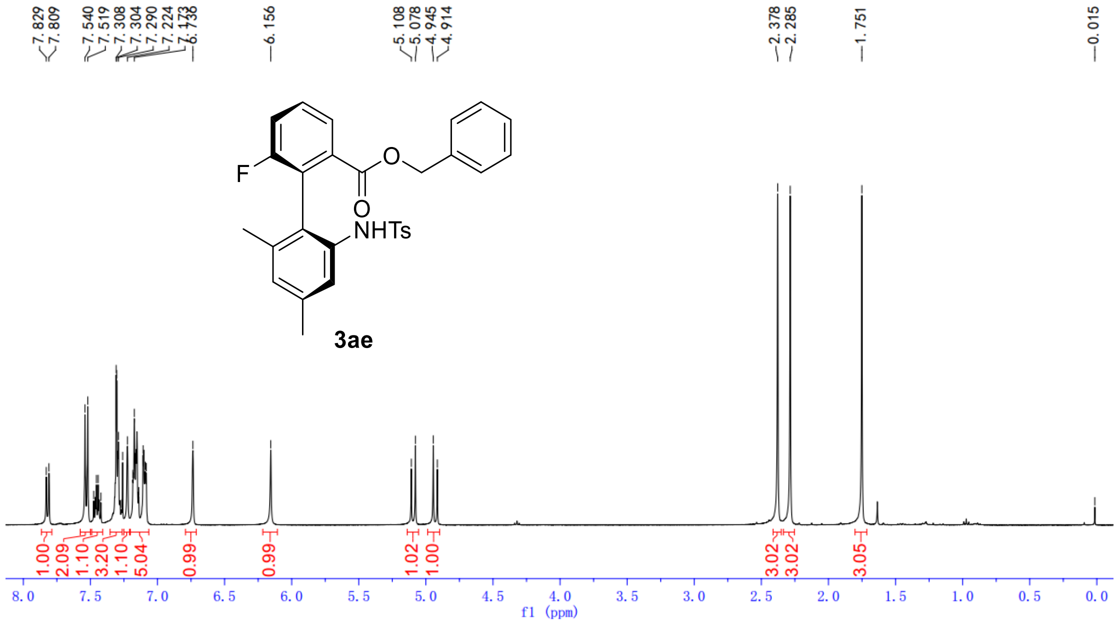

Supplementary Figure 106. ^{1}H NMR Spectrum of **3ae**

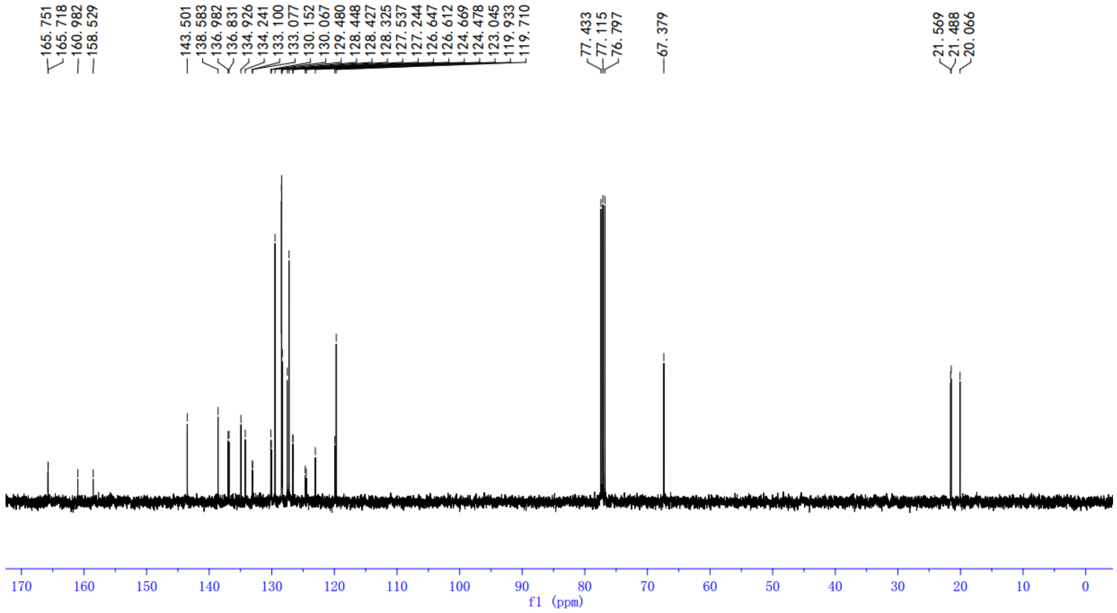

Supplementary Figure 107. ^{13}C NMR Spectrum of **3ae**

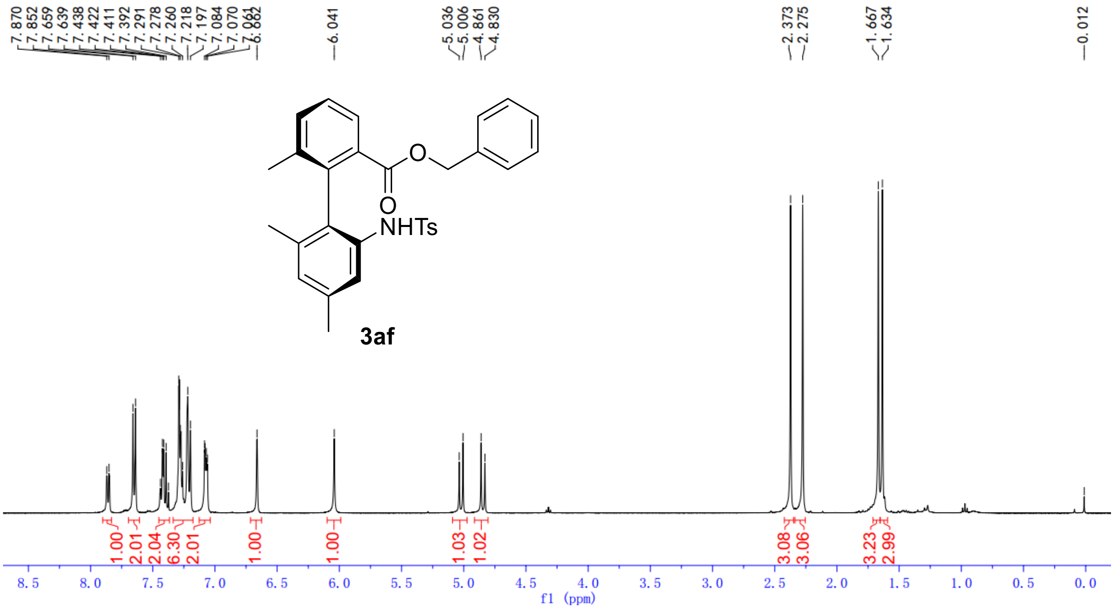

Supplementary Figure 108. ^{1}H NMR Spectrum of **3af**

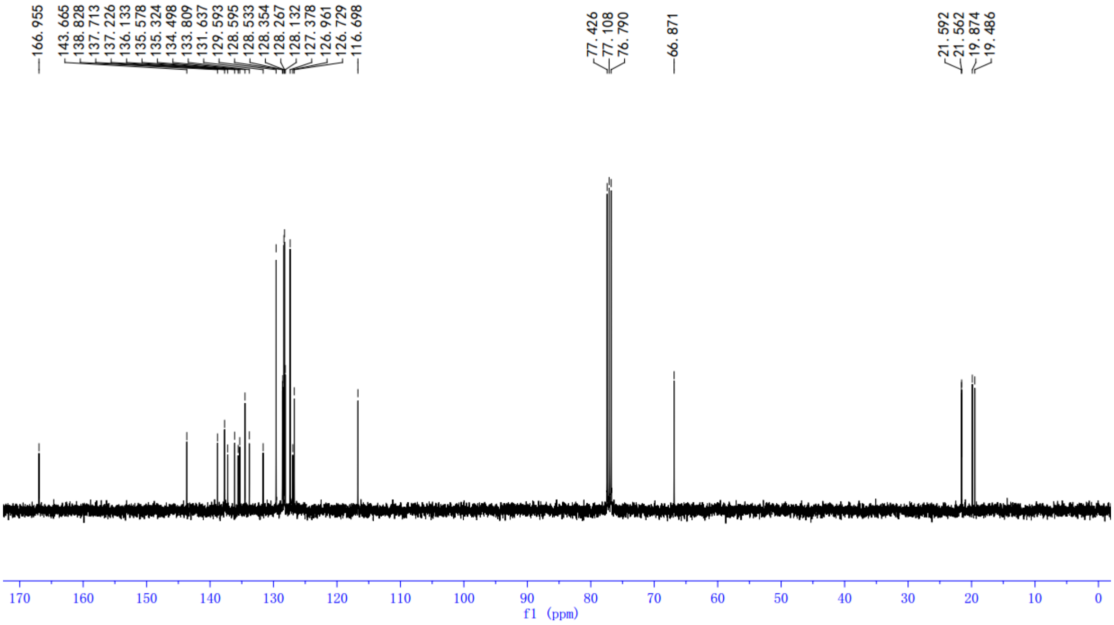

Supplementary Figure 109. ^{13}C NMR Spectrum of **3af**

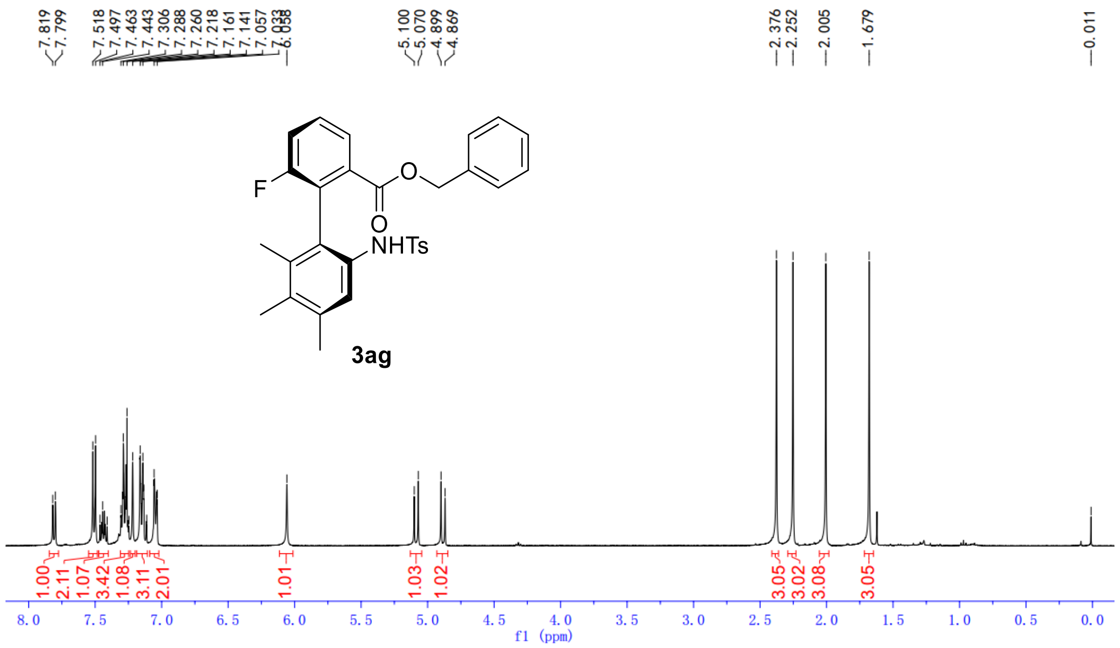

Supplementary Figure 110. ^{1}H NMR Spectrum of **3ag**

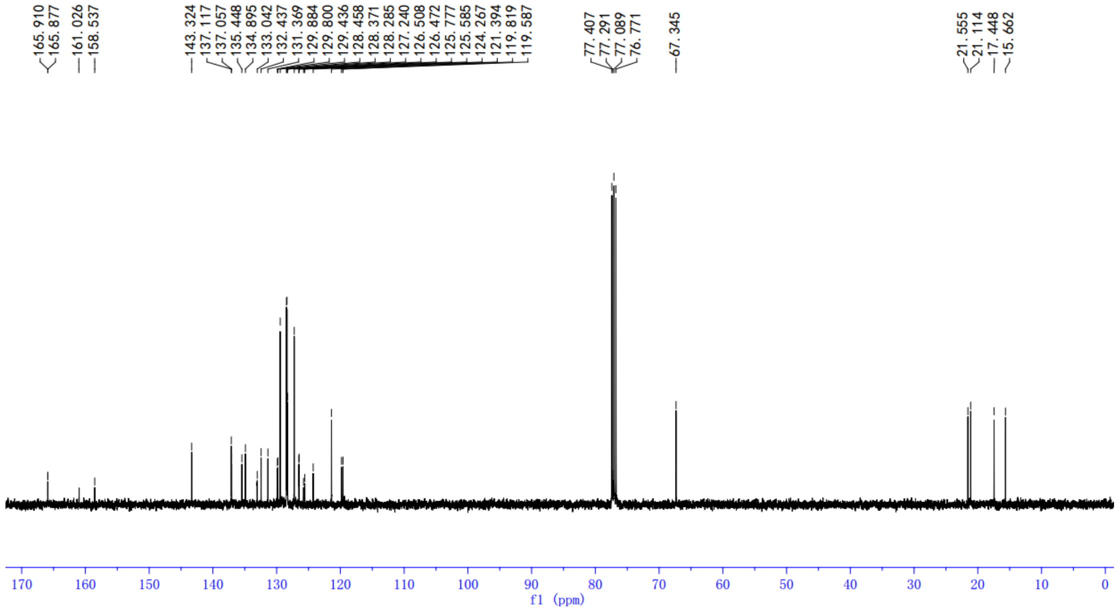

Supplementary Figure 111. ^{13}C NMR Spectrum of **3ag**

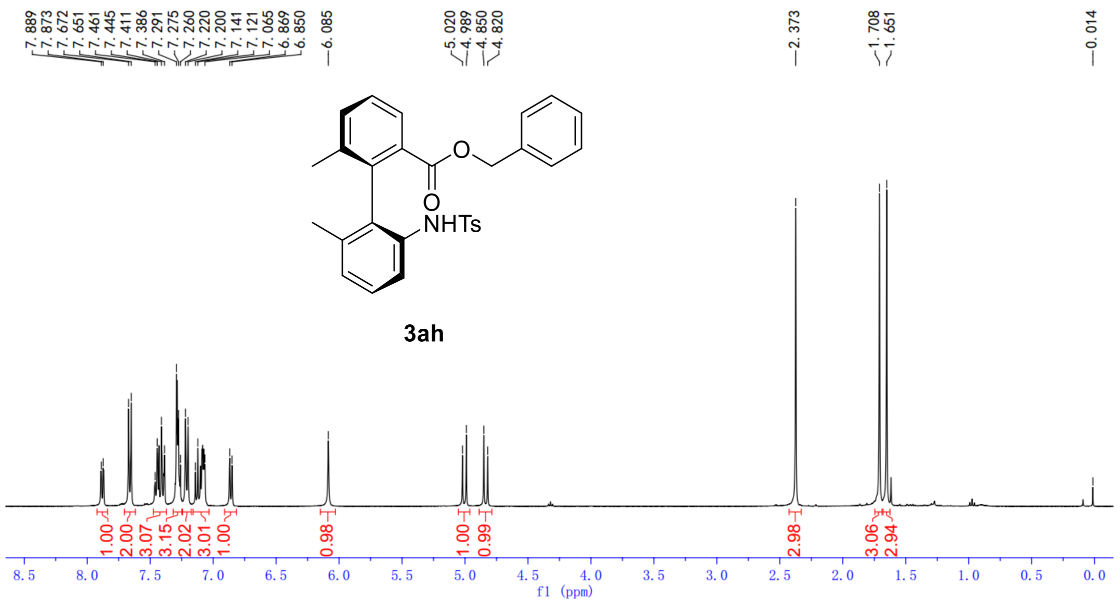

Supplementary Figure 112. ^{1}H NMR Spectrum of **3ah**

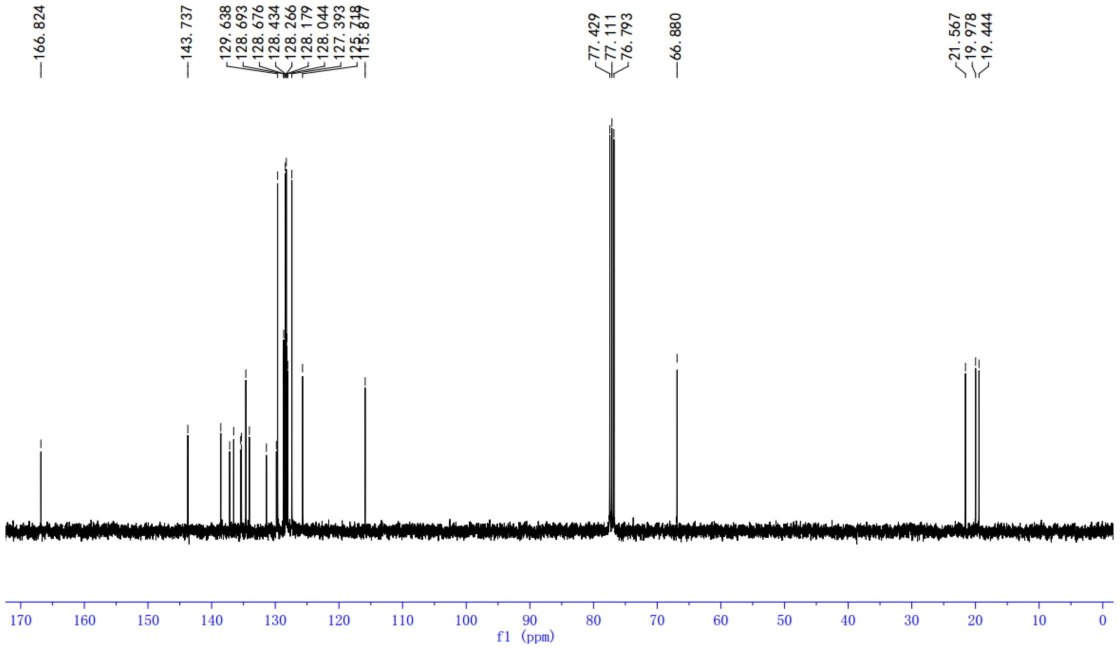

Supplementary Figure 113. ^{13}C NMR Spectrum of **3ah**

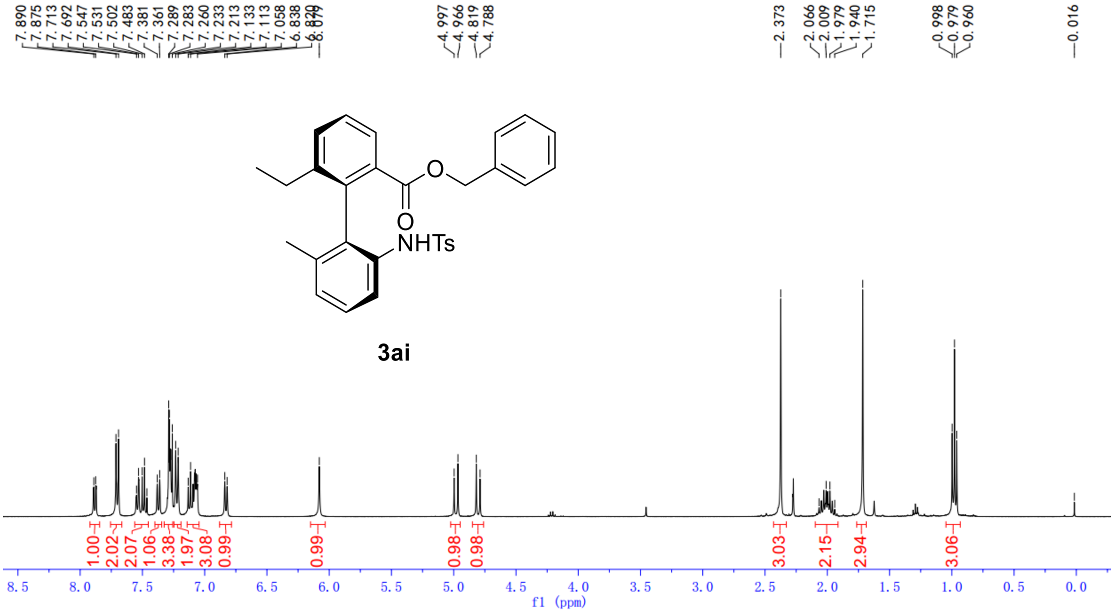

Supplementary Figure 114. ^{1}H NMR Spectrum of **3ai**

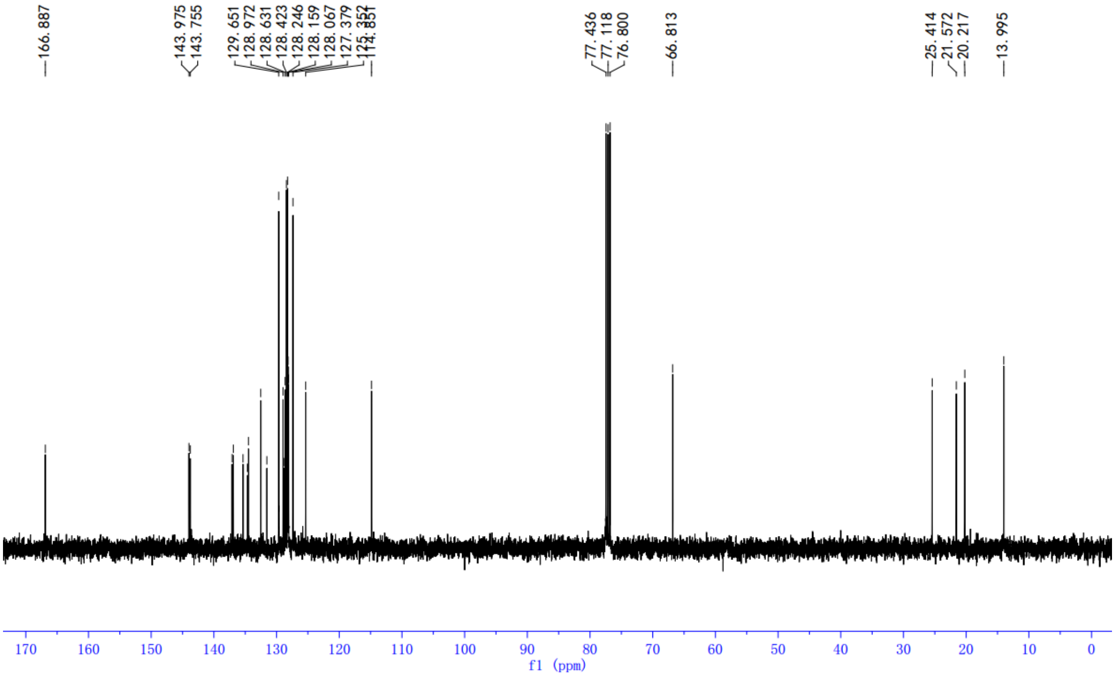

Supplementary Figure 115. ^{13}C NMR Spectrum of **3ai**

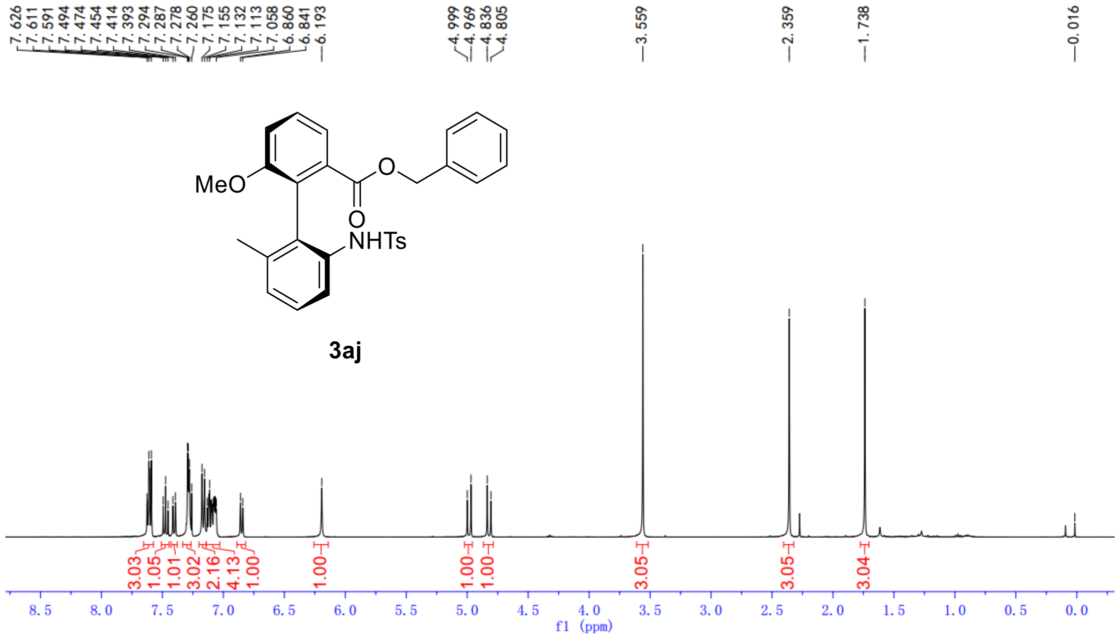

Supplementary Figure 116. ^{1}H NMR Spectrum of **3aj**

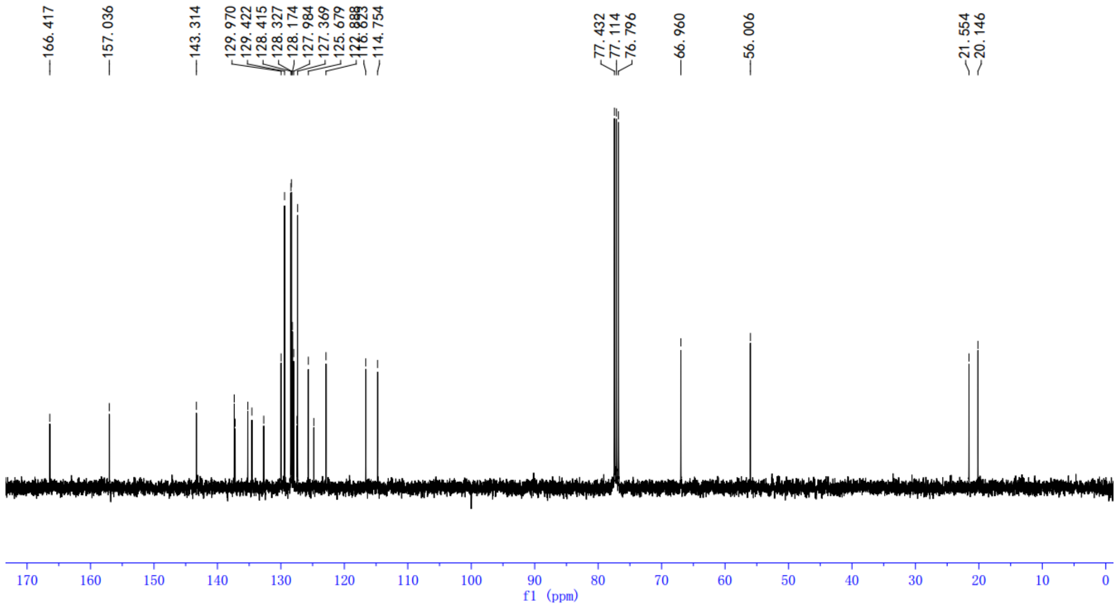

Supplementary Figure 117. ^{13}C NMR Spectrum of **3aj**

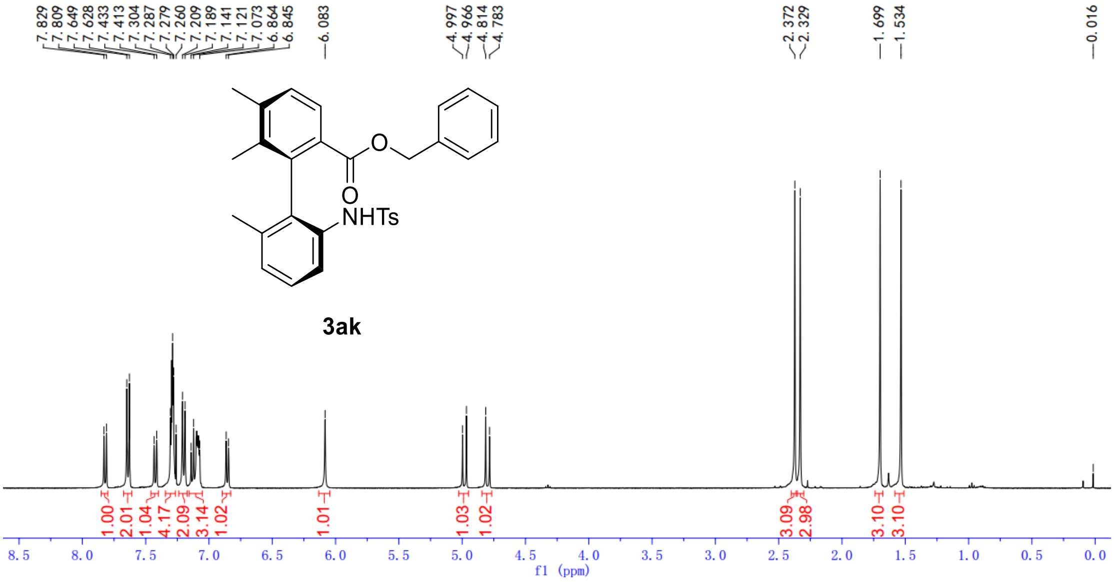

Supplementary Figure 118. ^{1}H NMR Spectrum of **3ak**

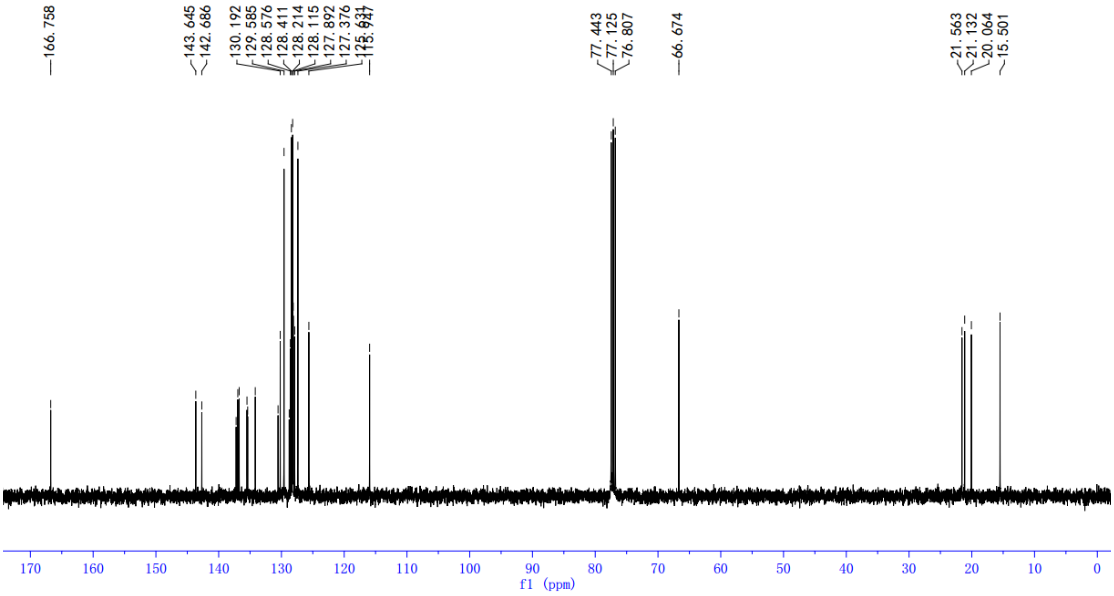

Supplementary Figure 119. ^{13}C NMR Spectrum of **3ak**

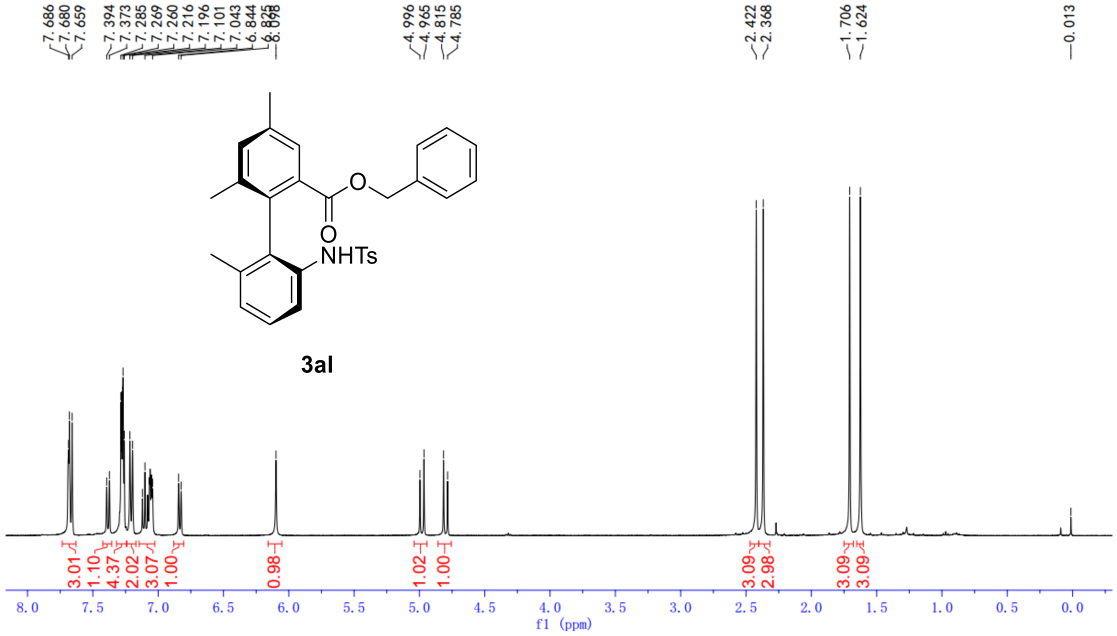

Supplementary Figure 120. ^{1}H NMR Spectrum of **3al**

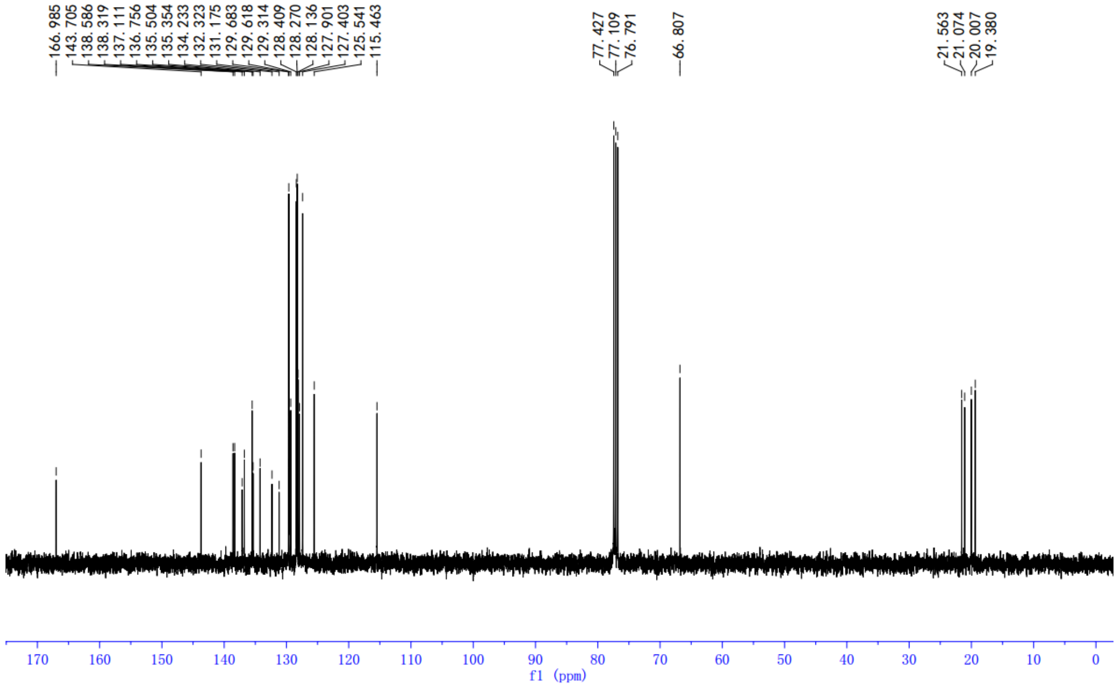

Supplementary Figure 121. ^{13}C NMR Spectrum of **3al**

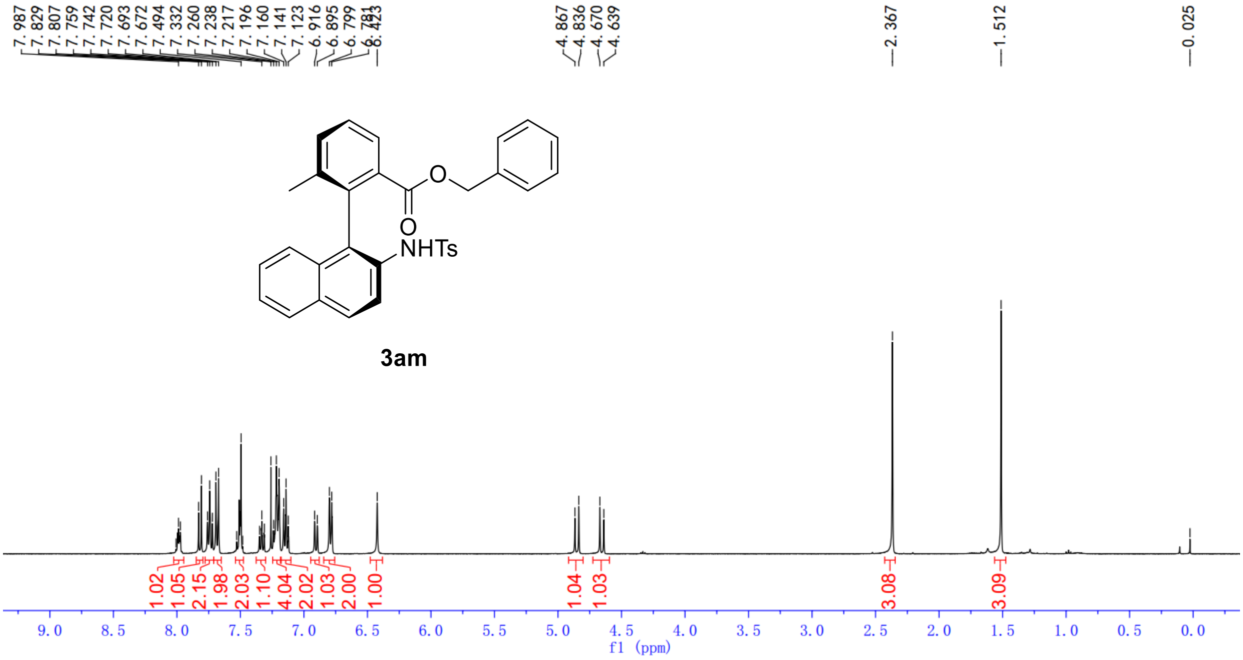

Supplementary Figure 122. ^{1}H NMR Spectrum of **3am**

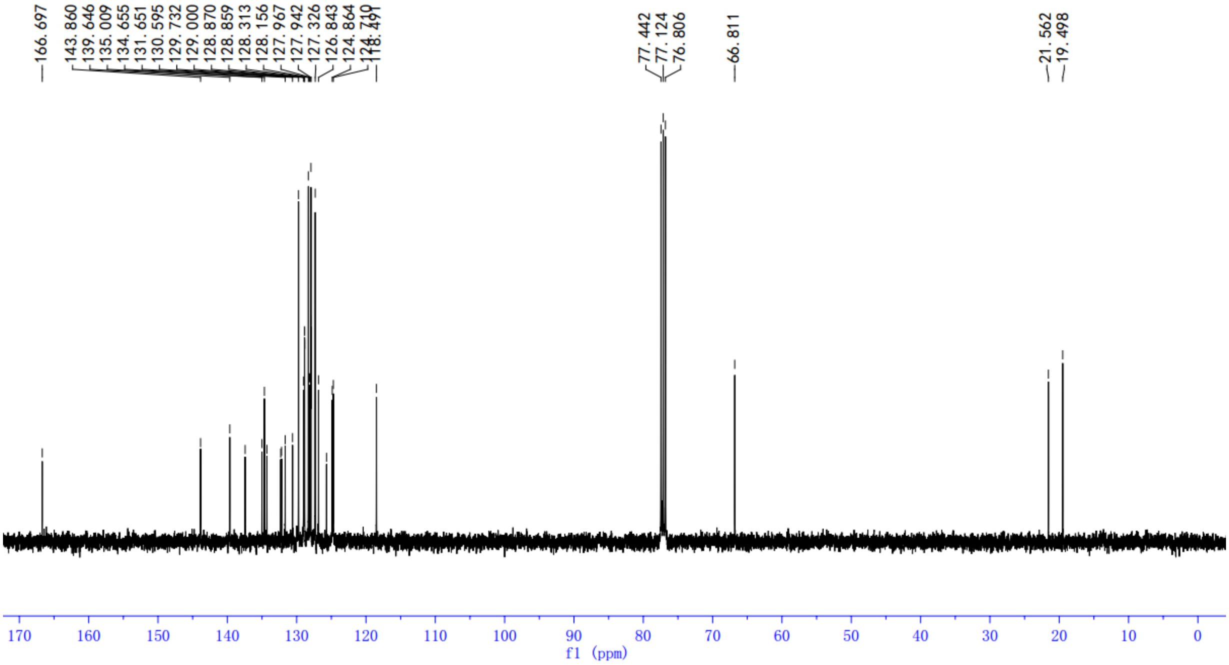

Supplementary Figure 123. ^{13}C NMR Spectrum of **3am**

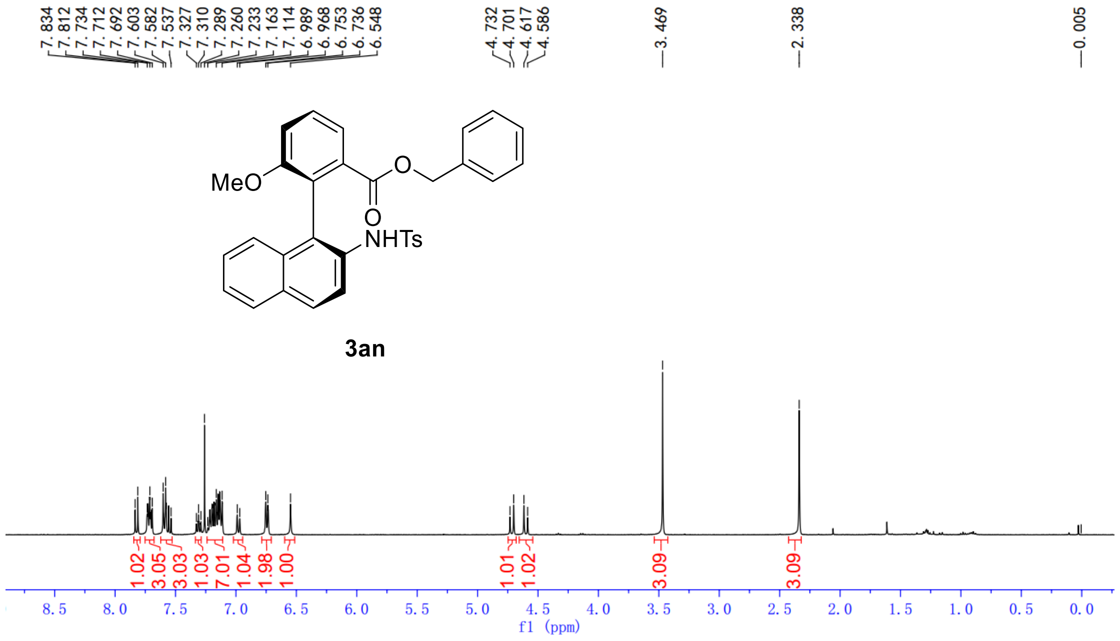

Supplementary Figure 124. ^{1}H NMR Spectrum of **3an**

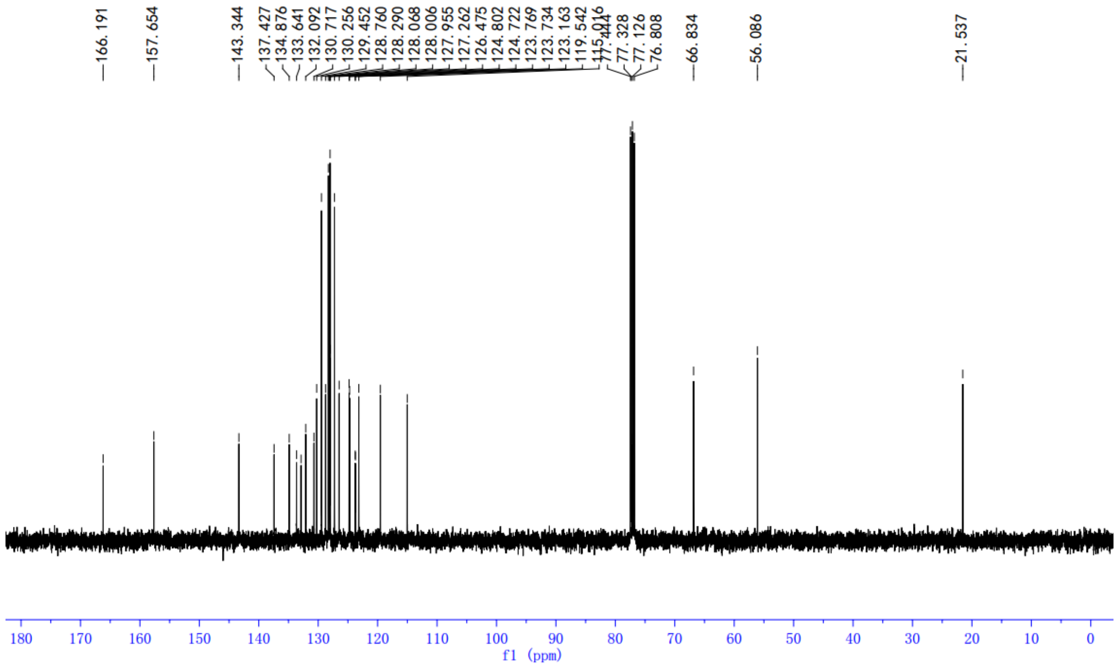

Supplementary Figure 125. ^{13}C NMR Spectrum of **3an**

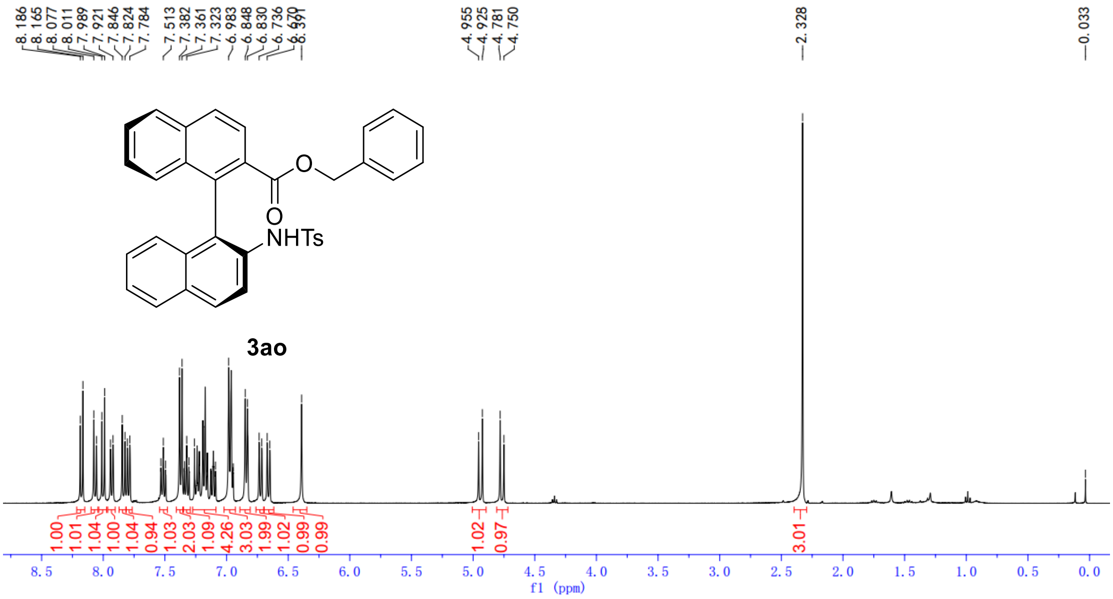

Supplementary Figure 126. ^{1}H NMR Spectrum of **3ao**

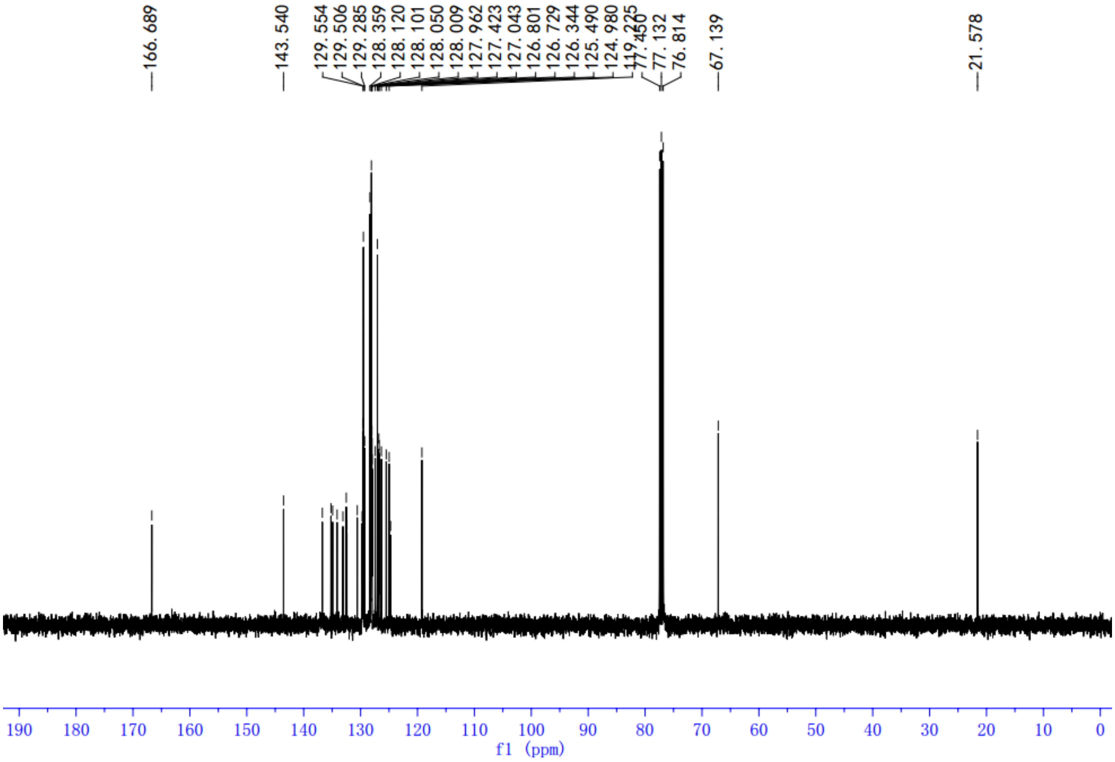

Supplementary Figure 127. ^{13}C NMR Spectrum of **3ao**

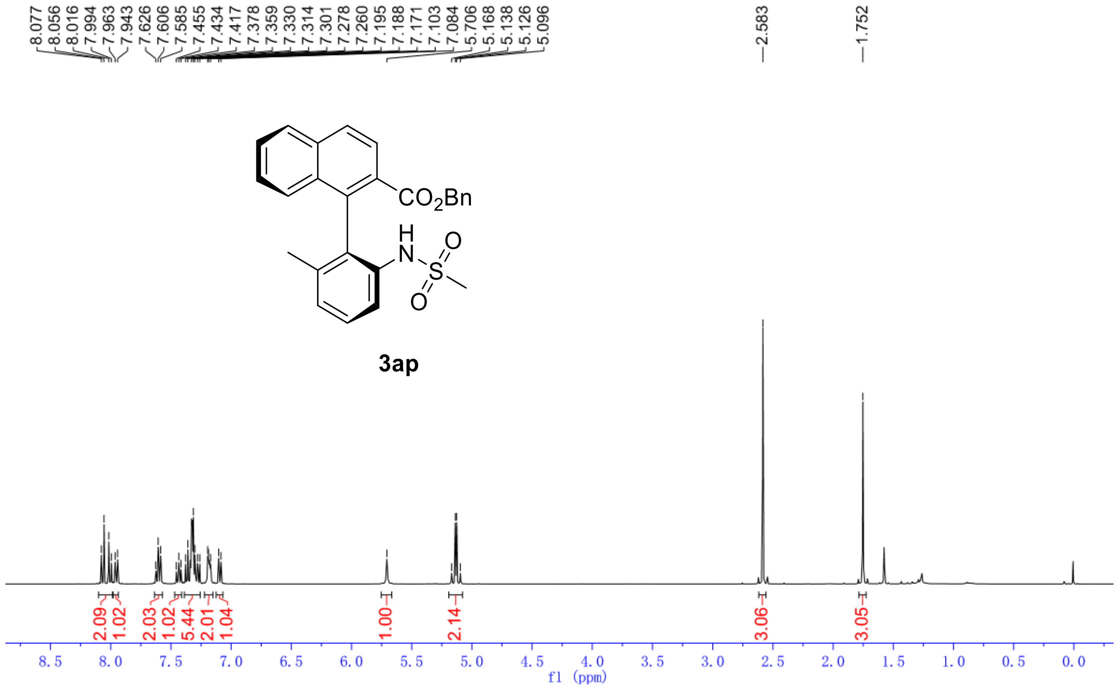

Supplementary Figure 128. ^{1}H NMR Spectrum of **3ap**

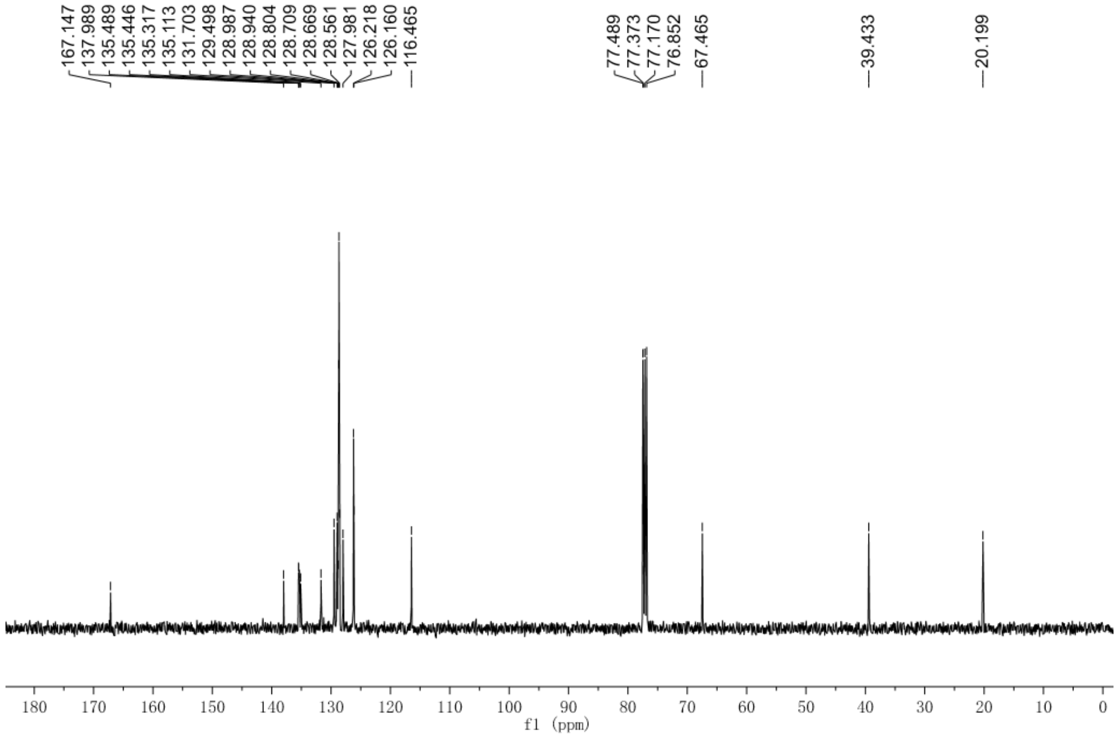

Supplementary Figure 129. ^{13}C NMR Spectrum of **3ap**

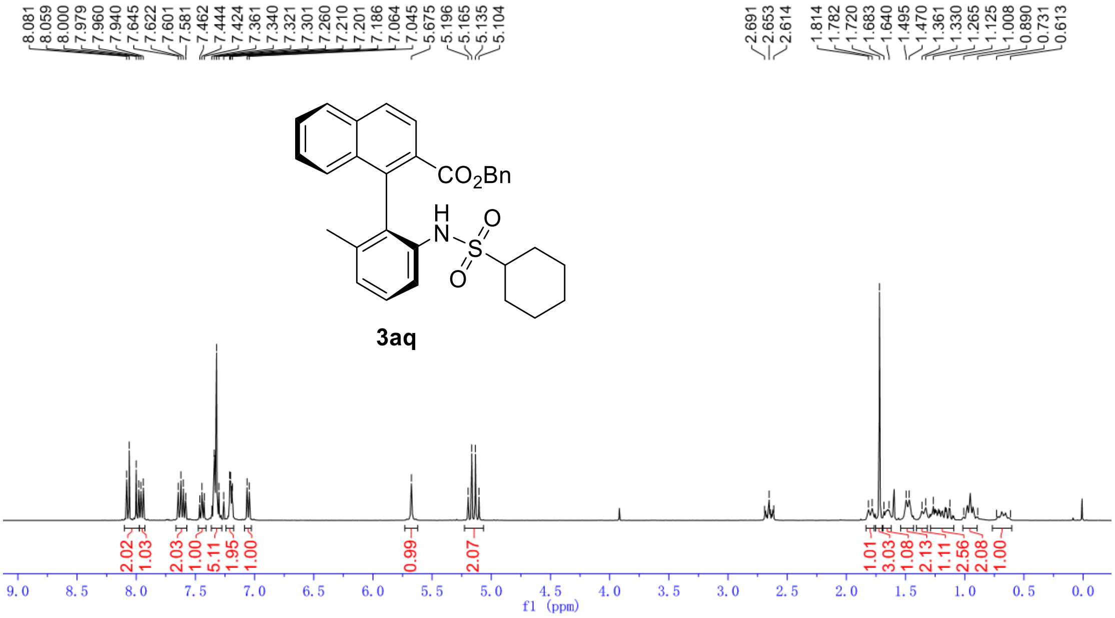

Supplementary Figure 130. ^{1}H NMR Spectrum of **3aq**

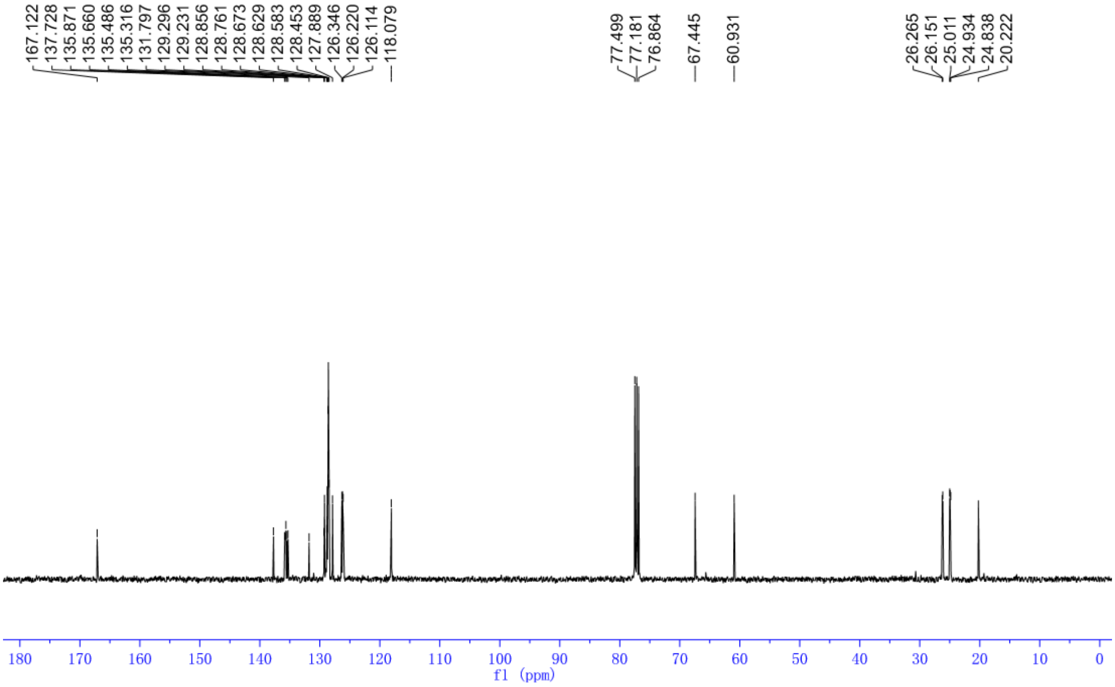

Supplementary Figure 131. ^{13}C NMR Spectrum of **3aq**

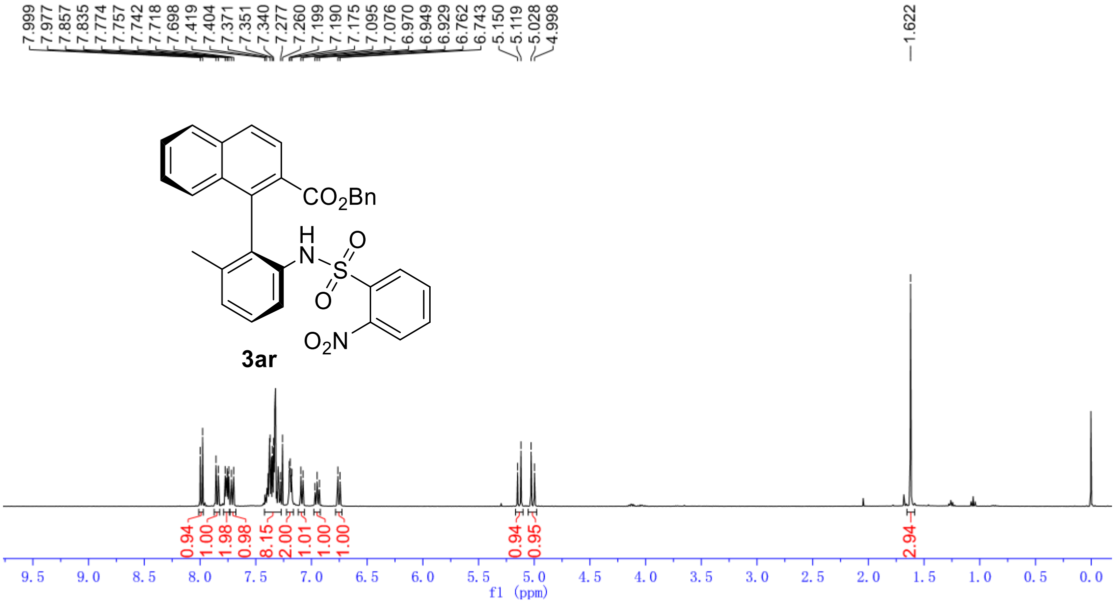

Supplementary Figure 132. ^{1}H NMR Spectrum of **3ar**

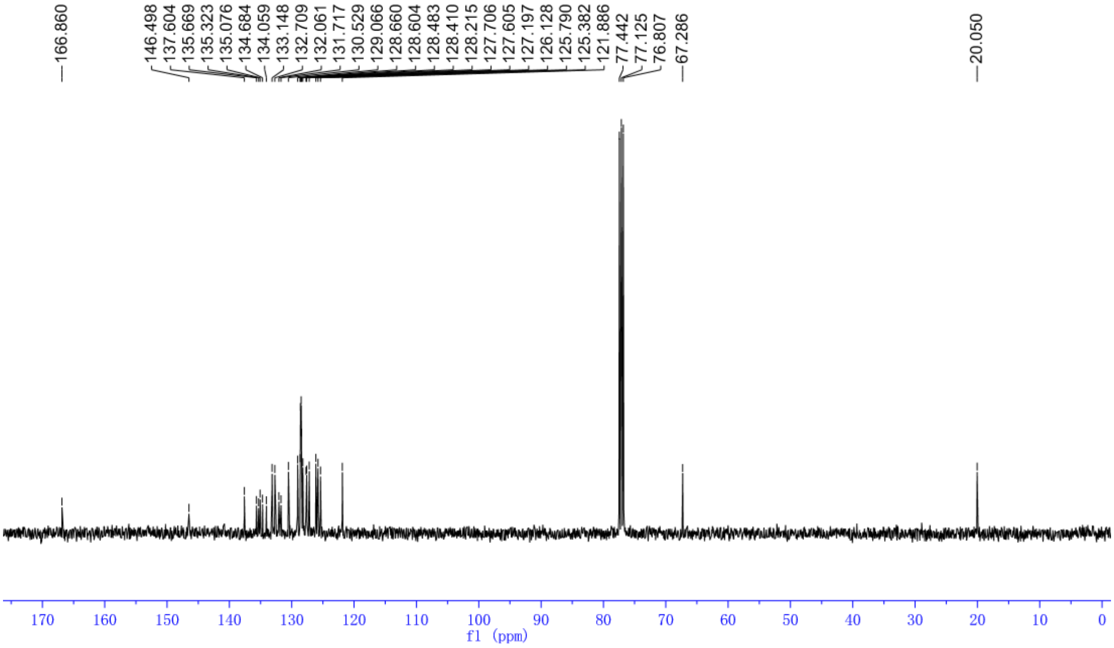

Supplementary Figure 133. ^{13}C NMR Spectrum of **3ar**

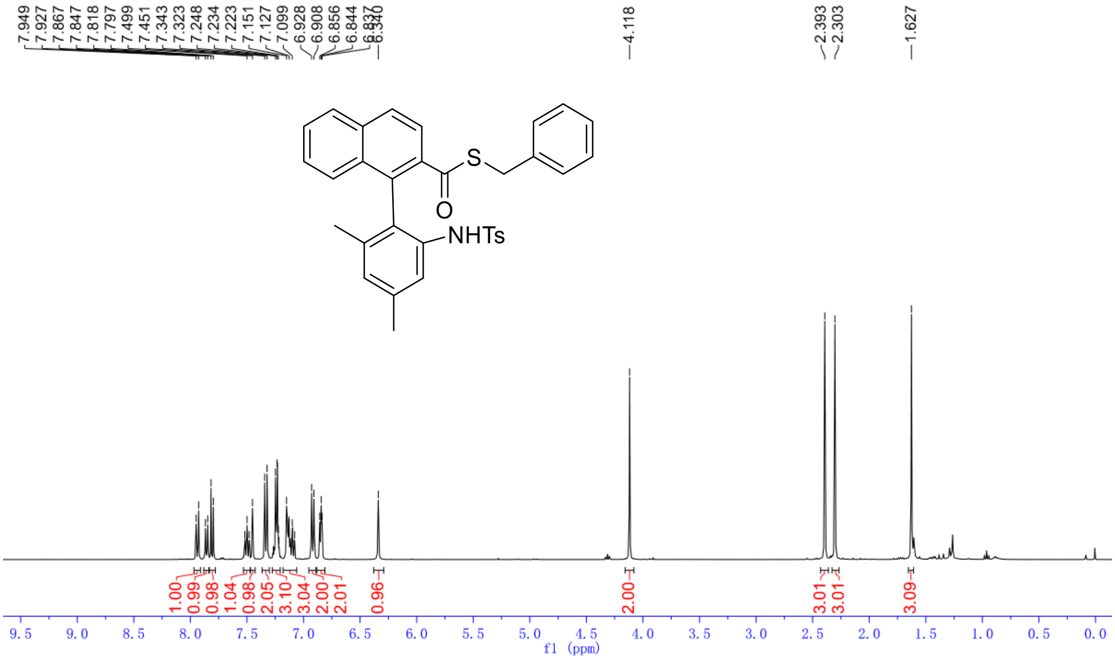

Supplementary Figure 134. ^{1}H NMR Spectrum

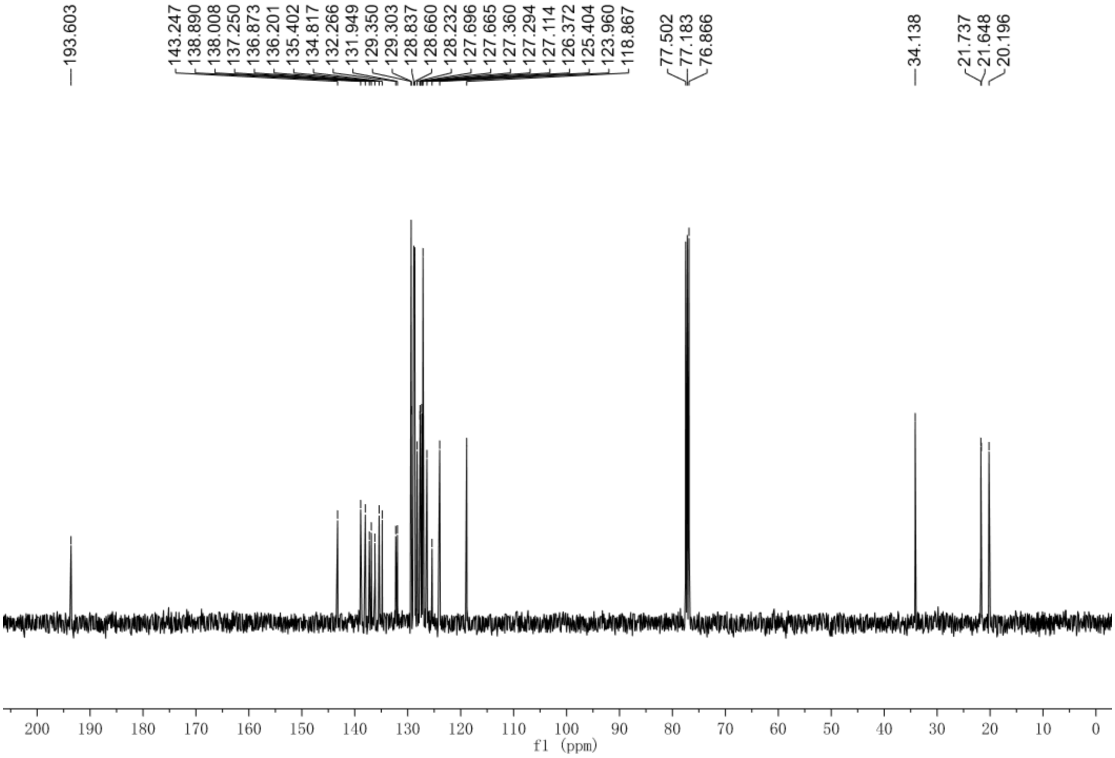

Supplementary Figure 135. ^{13}C NMR Spectrum

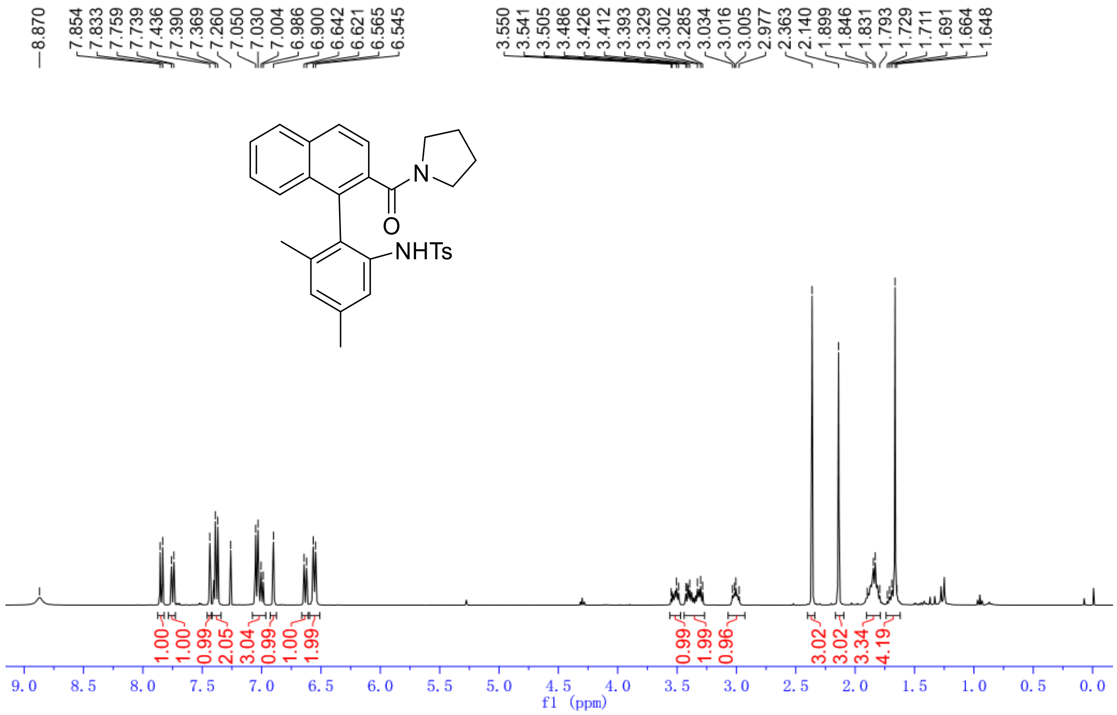

Supplementary Figure 136. ^{1}H NMR Spectrum

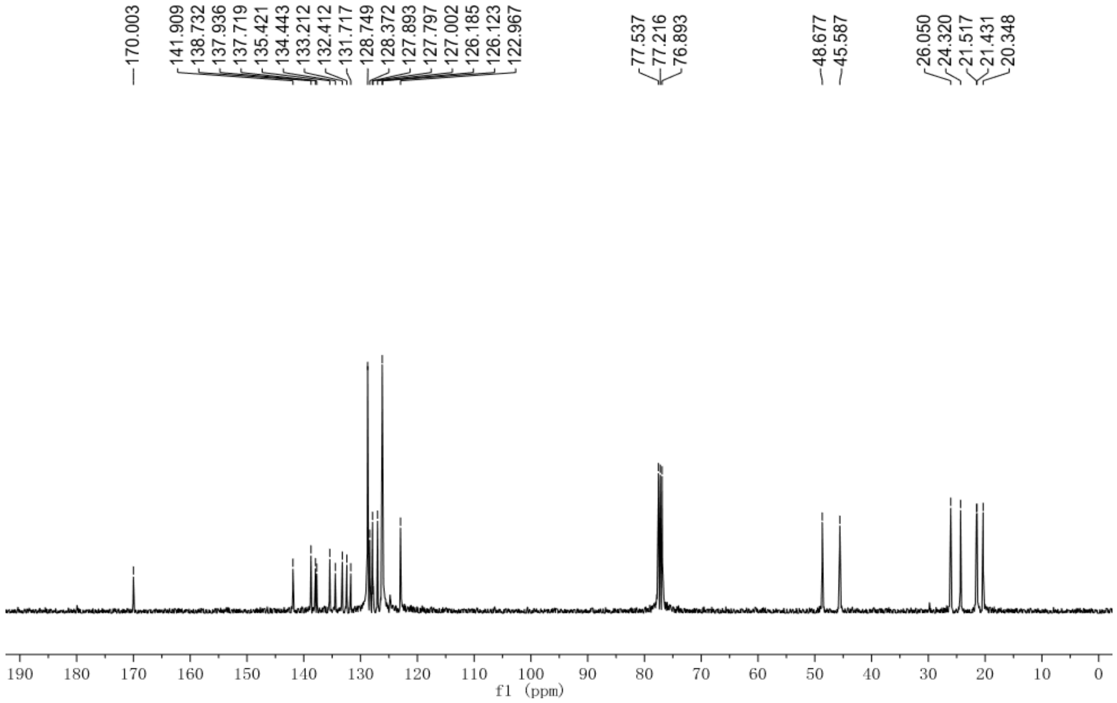

Supplementary Figure 137. ^{13}C NMR Spectrum

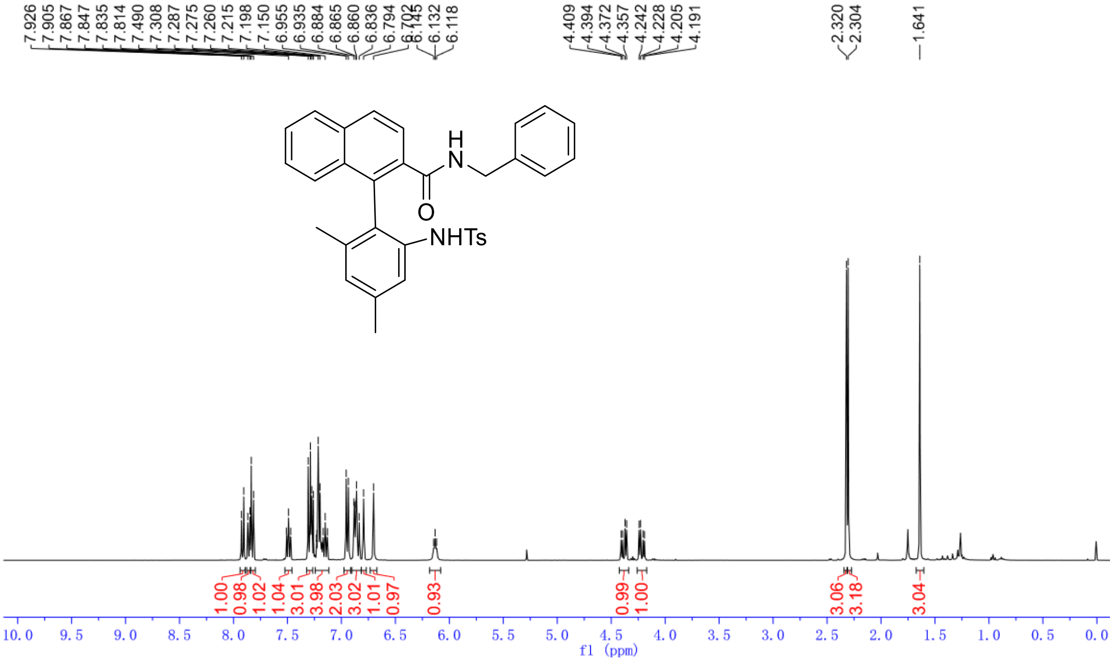

Supplementary Figure 138. ^{1}H NMR Spectrum

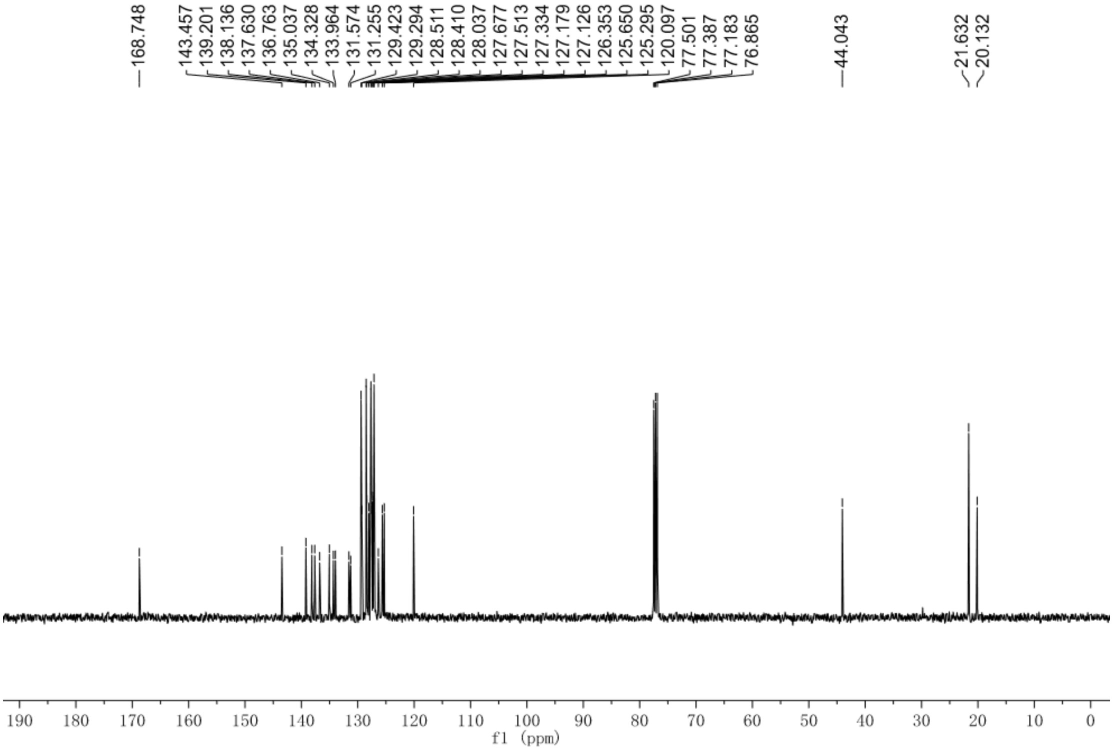

Supplementary Figure 139. ^{13}C NMR Spectrum

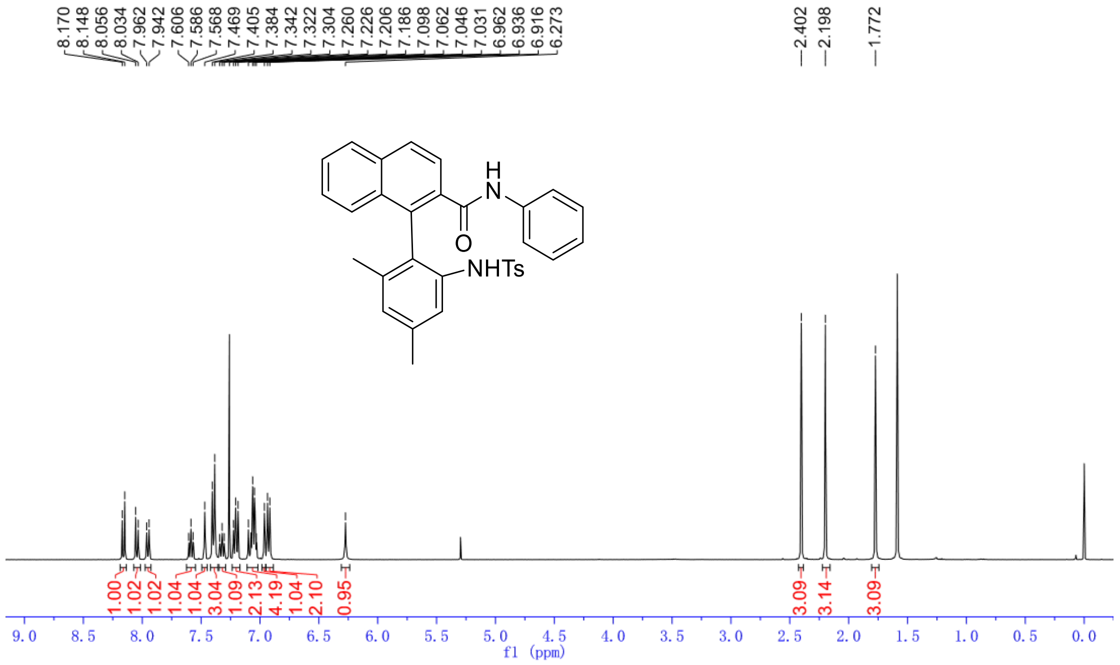

Supplementary Figure 140. ^{1}H NMR Spectrum

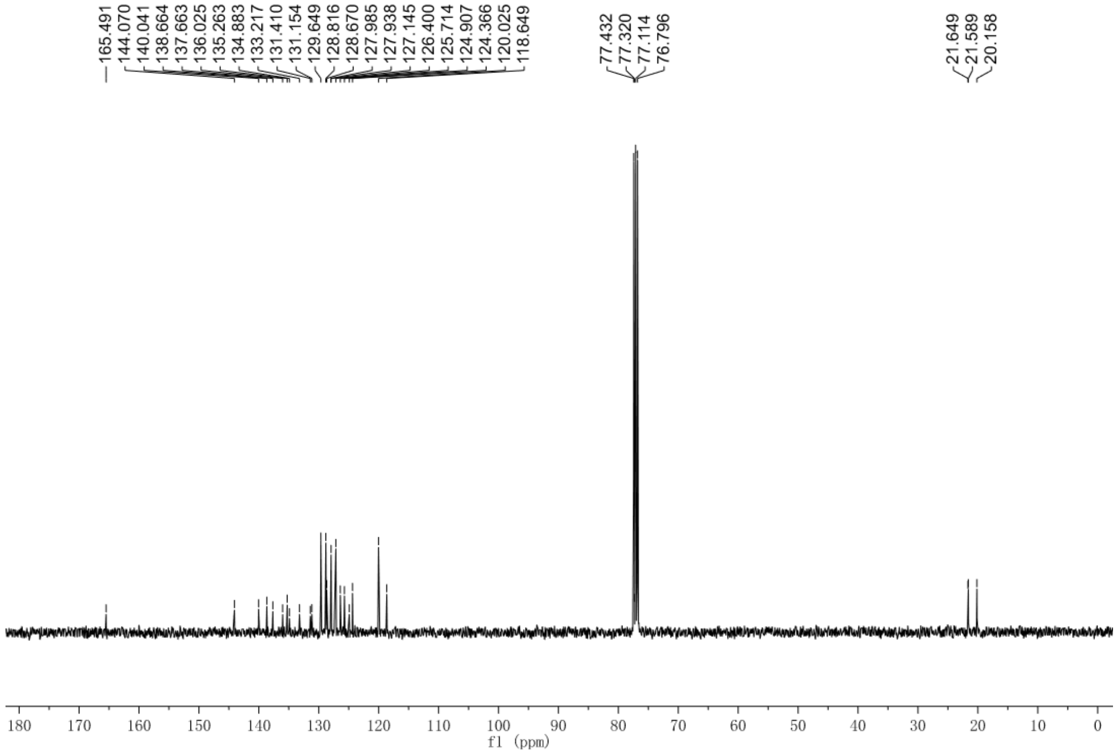

Supplementary Figure 141. ^{13}C NMR Spectrum

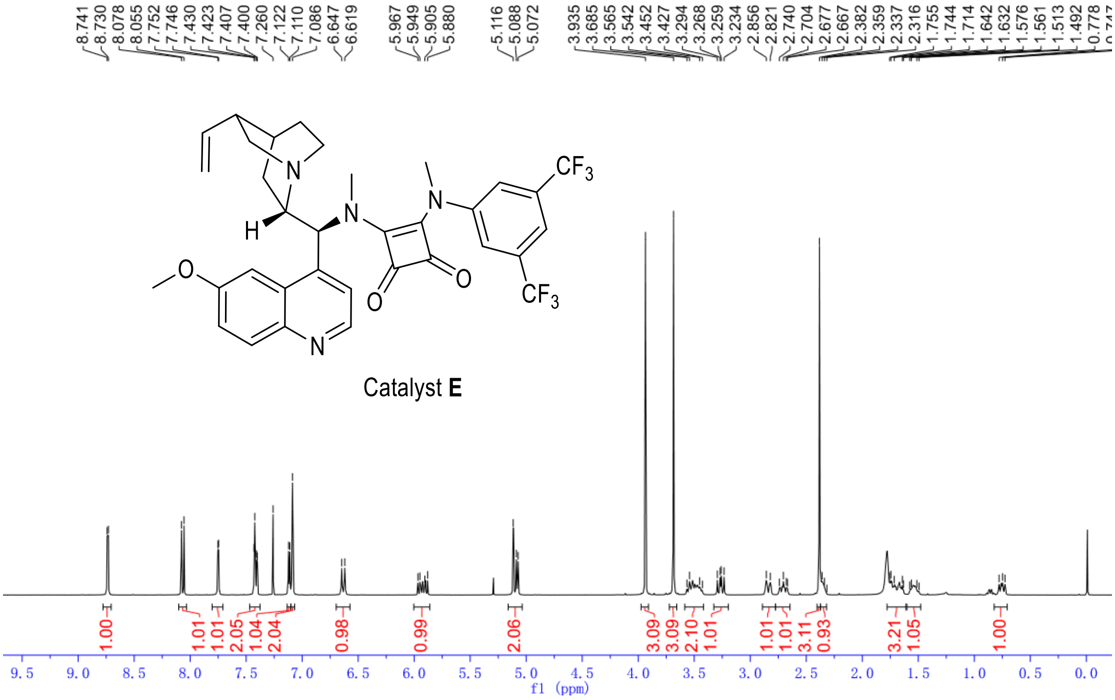

Supplementary Figure 142. ^{1}H NMR Spectrum of Catalyst **E**

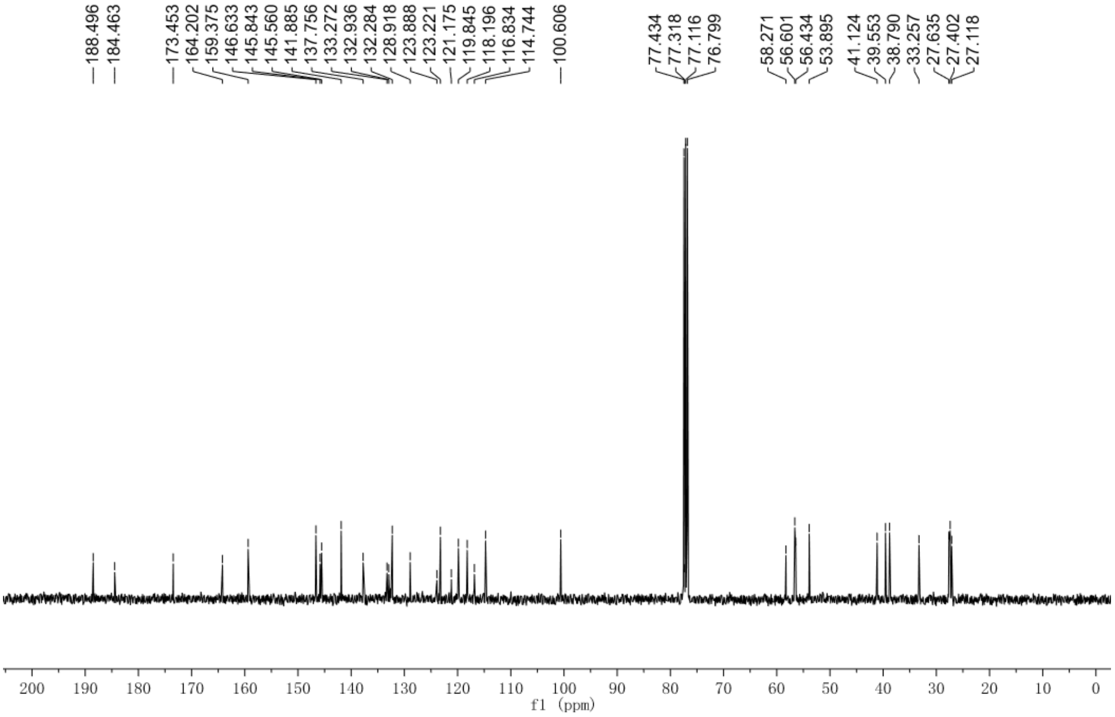

Supplementary Figure 143. ^{13}C NMR Spectrum of Catalyst **E**

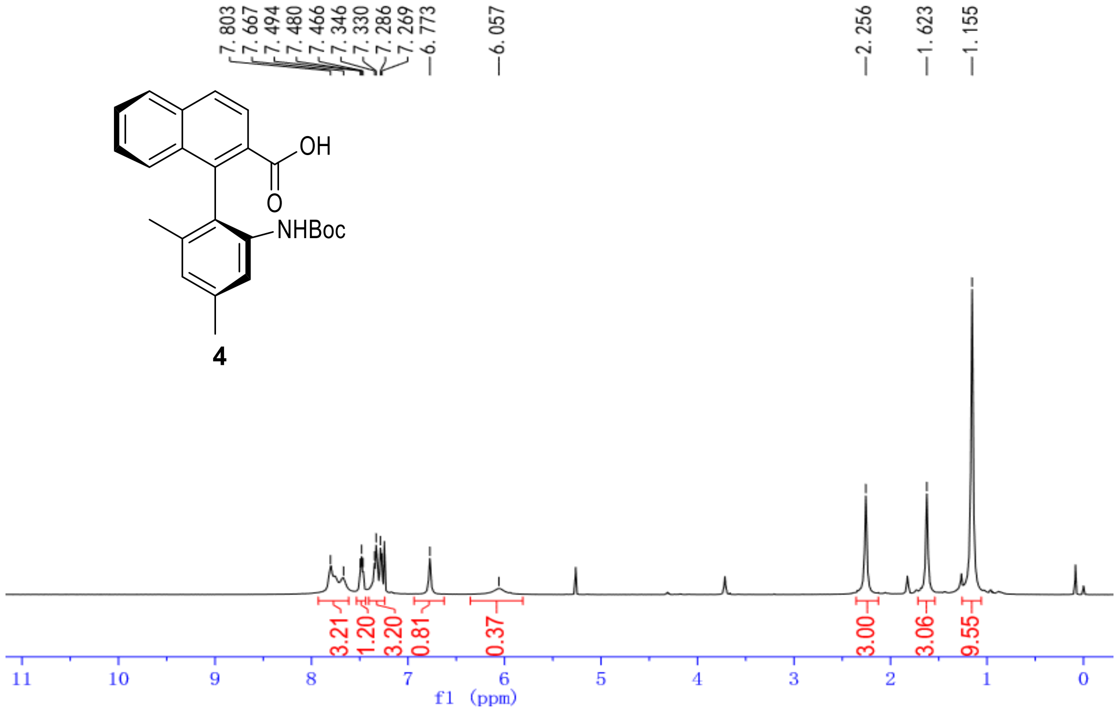

Supplementary Figure 144. ^{1}H NMR Spectrum of **4**

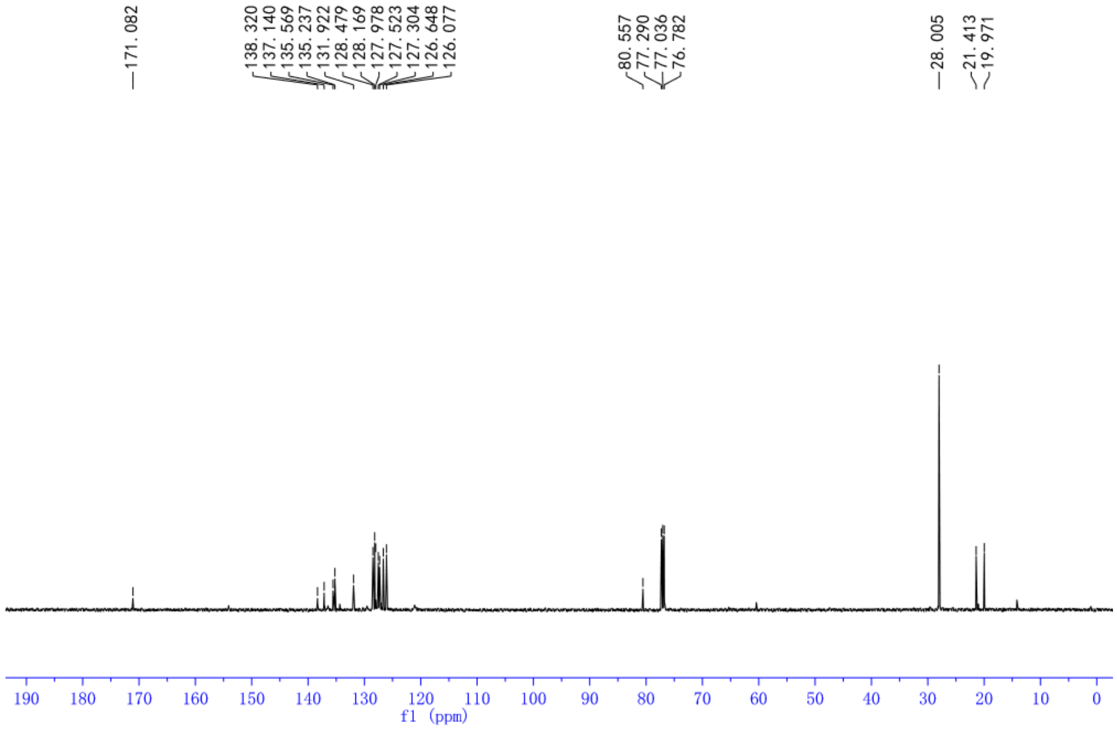

Supplementary Figure 145. ^{13}C NMR Spectrum of **4**

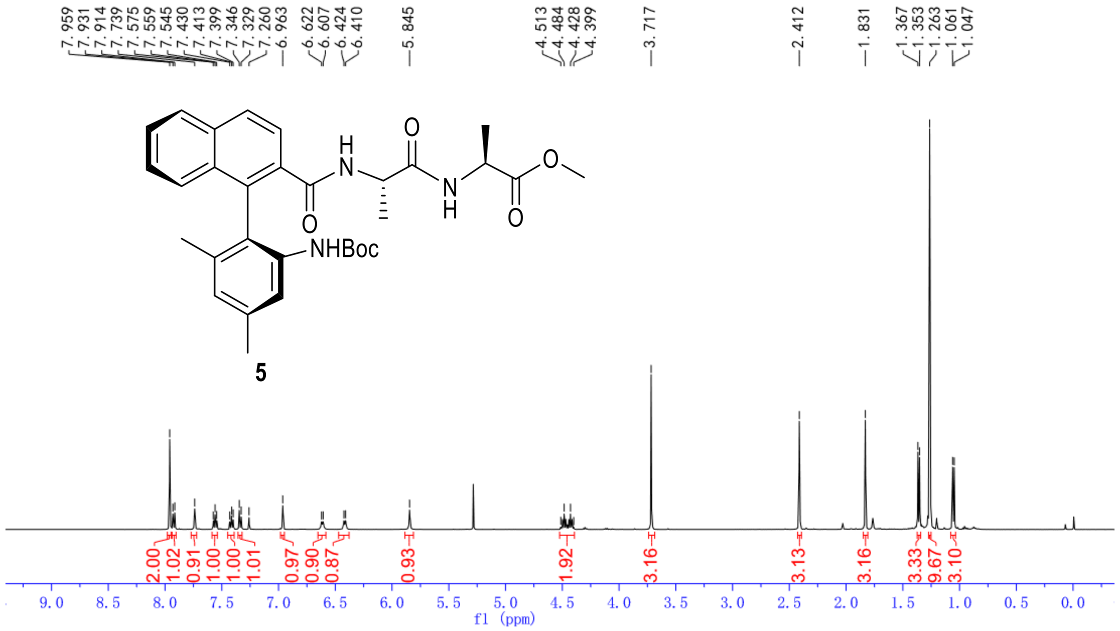

Supplementary Figure 146. ^{1}H NMR Spectrum of **5**

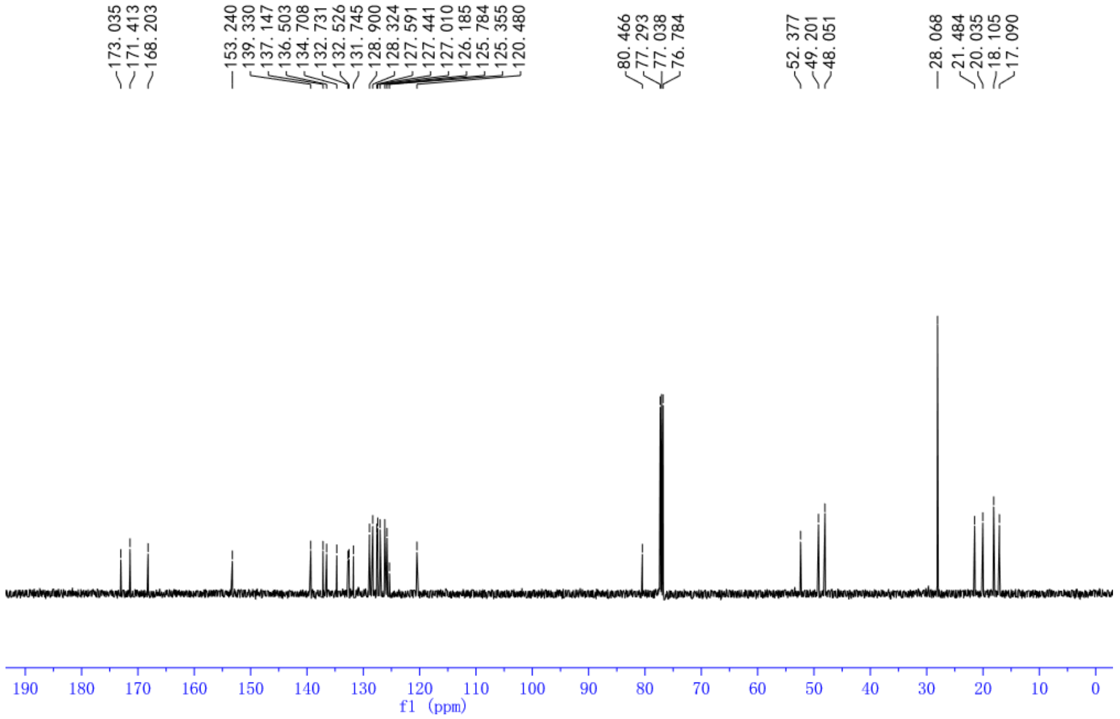

Supplementary Figure 147. ^{13}C NMR Spectrum of **5**

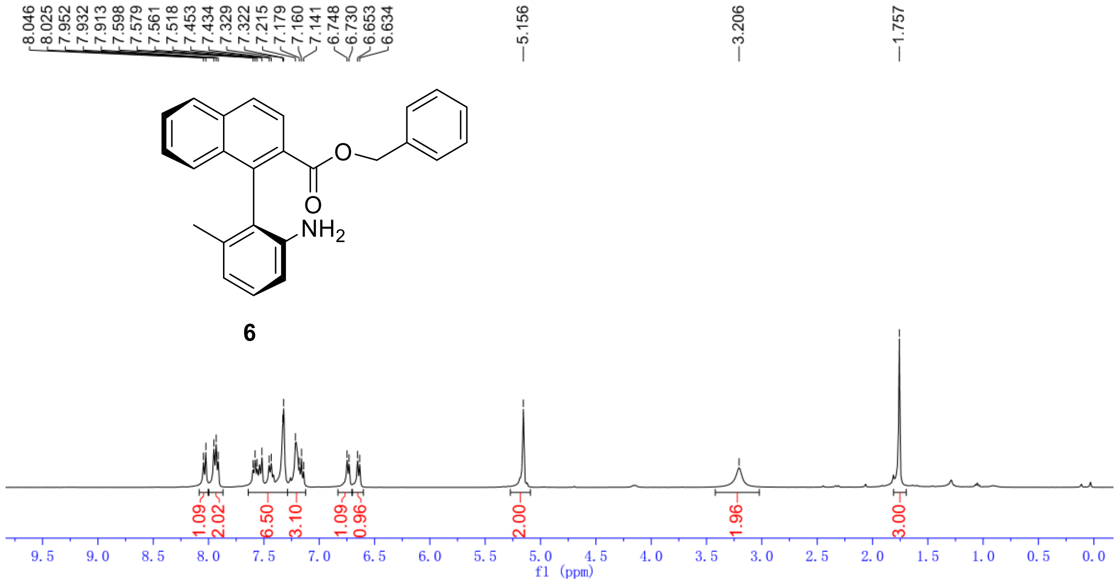

Supplementary Figure 148. ^{1}H NMR Spectrum of **6**

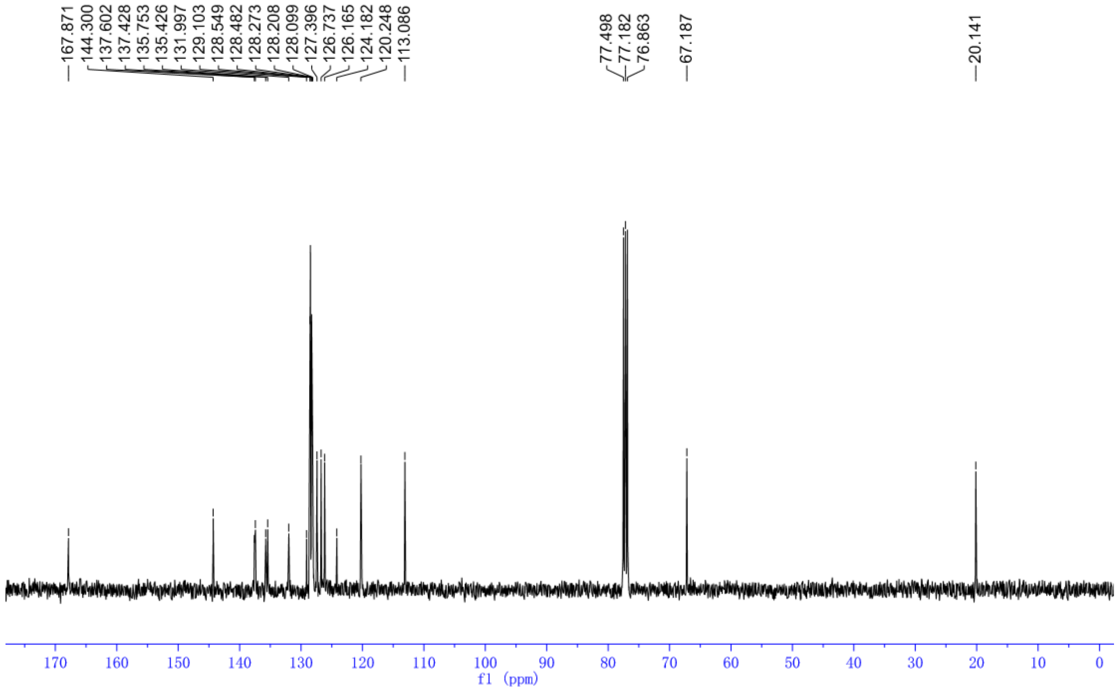

Supplementary Figure 149. ^{13}C NMR Spectrum of **6**

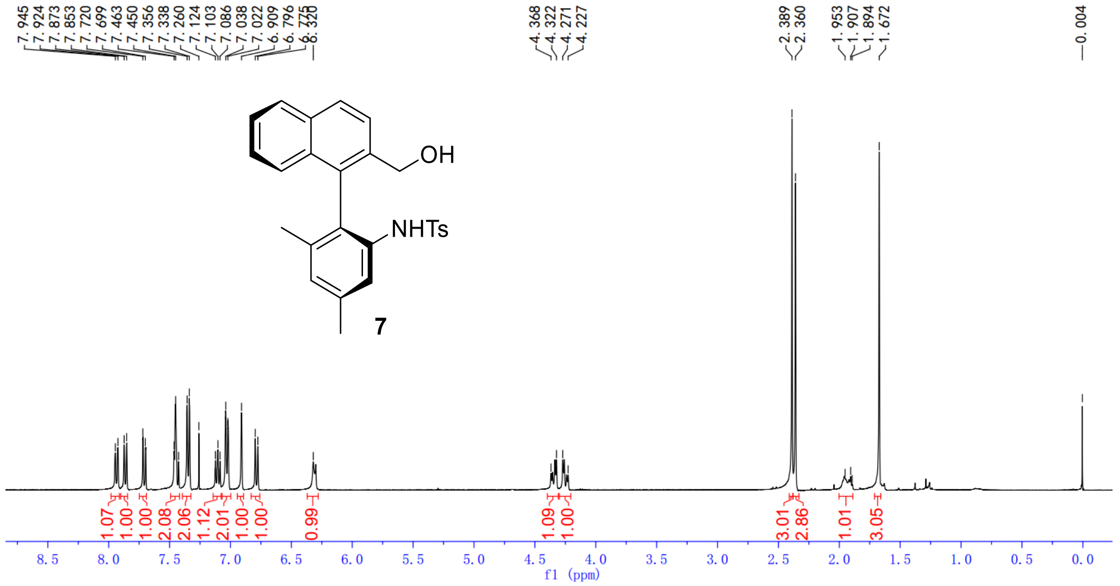

Supplementary Figure 150. ^{1}H NMR Spectrum of **7**

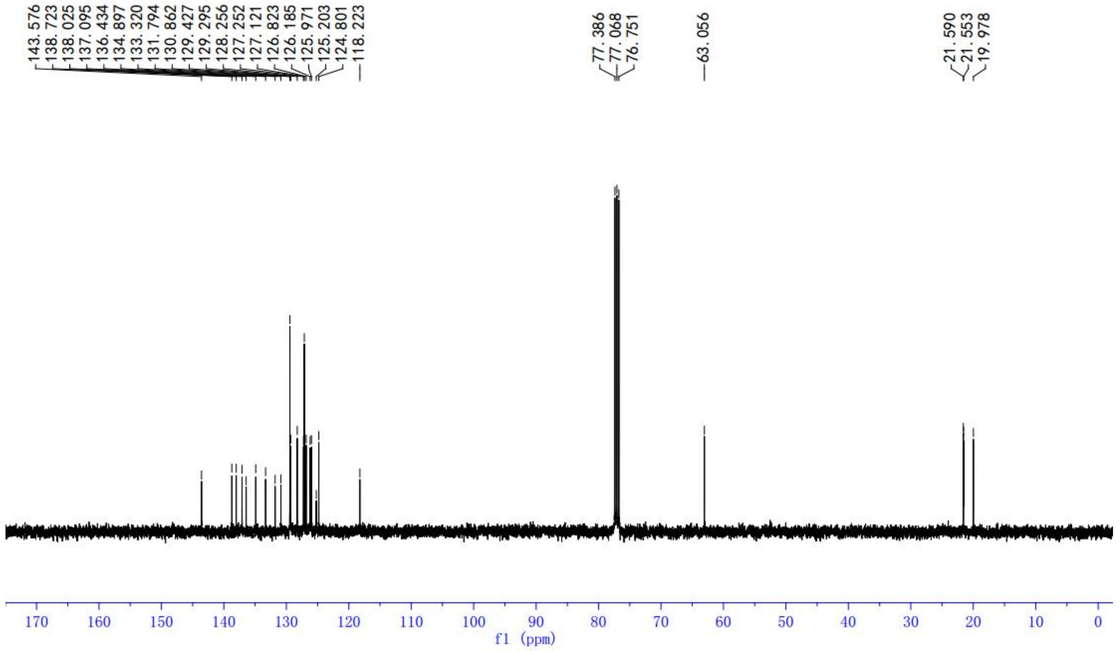

Supplementary Figure 151. ^{13}C NMR Spectrum of **7**

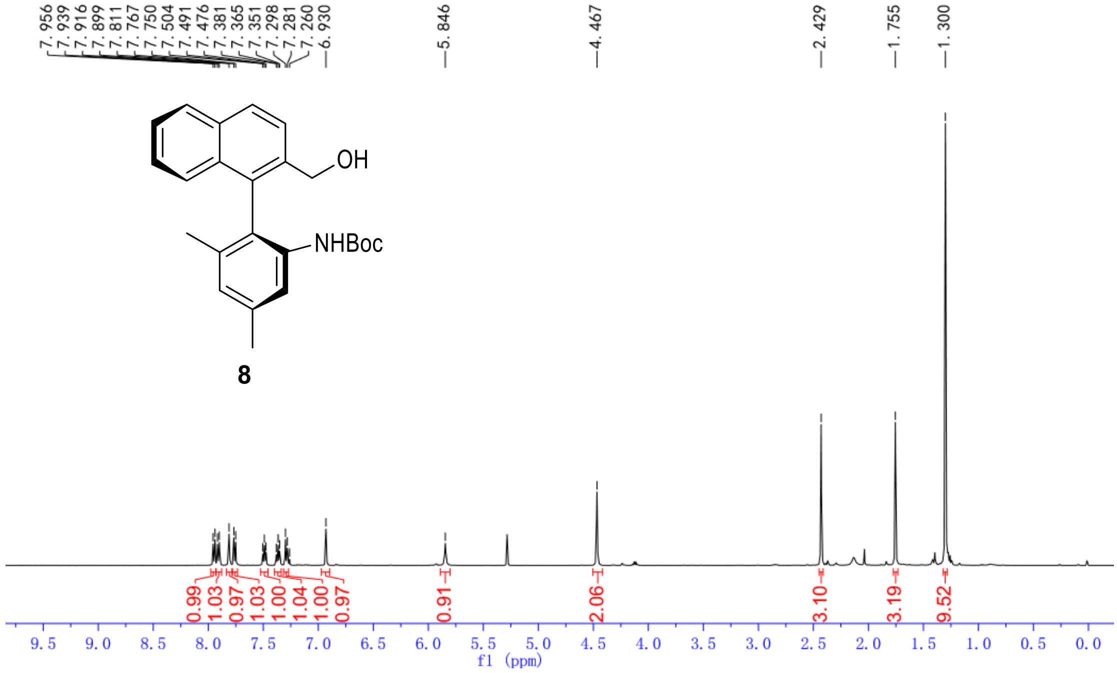

Supplementary Figure 152. ^{1}H NMR Spectrum of **8**

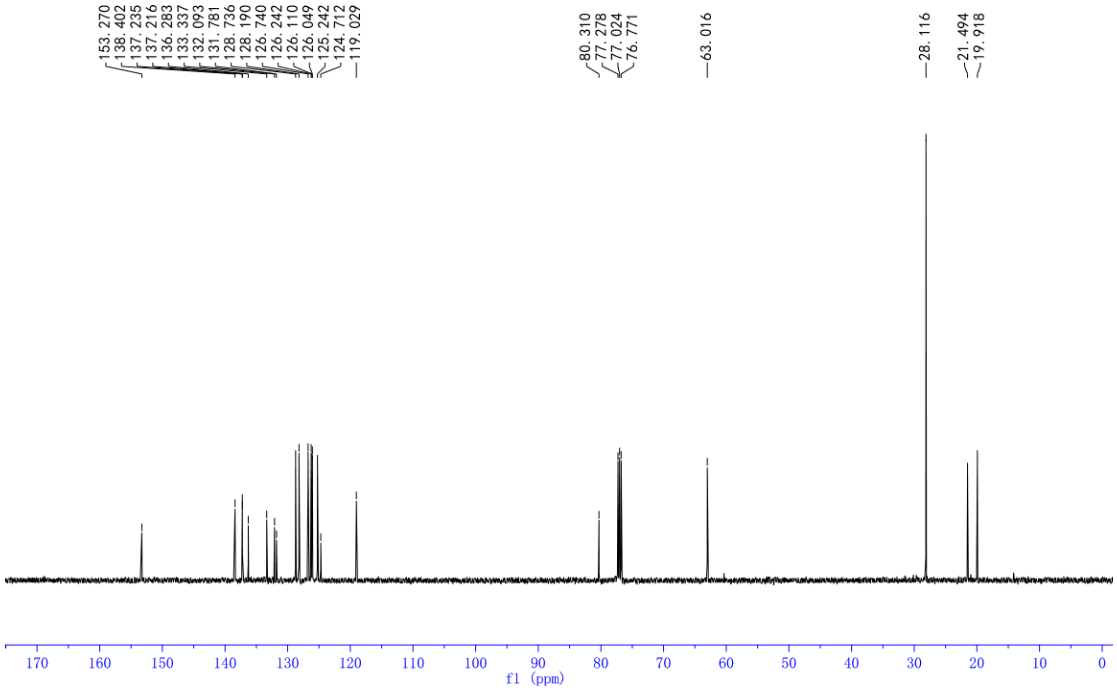

Supplementary Figure 153. ^{13}C NMR Spectrum of **8**

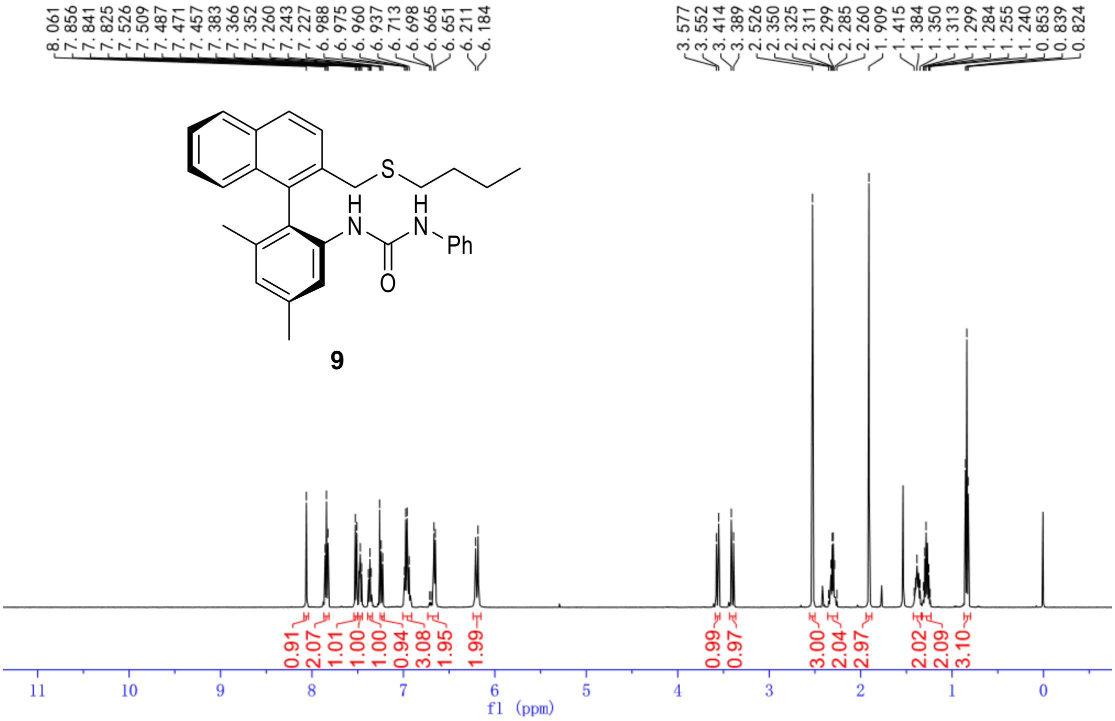

Supplementary Figure 154. ^{1}H NMR Spectrum of **9**

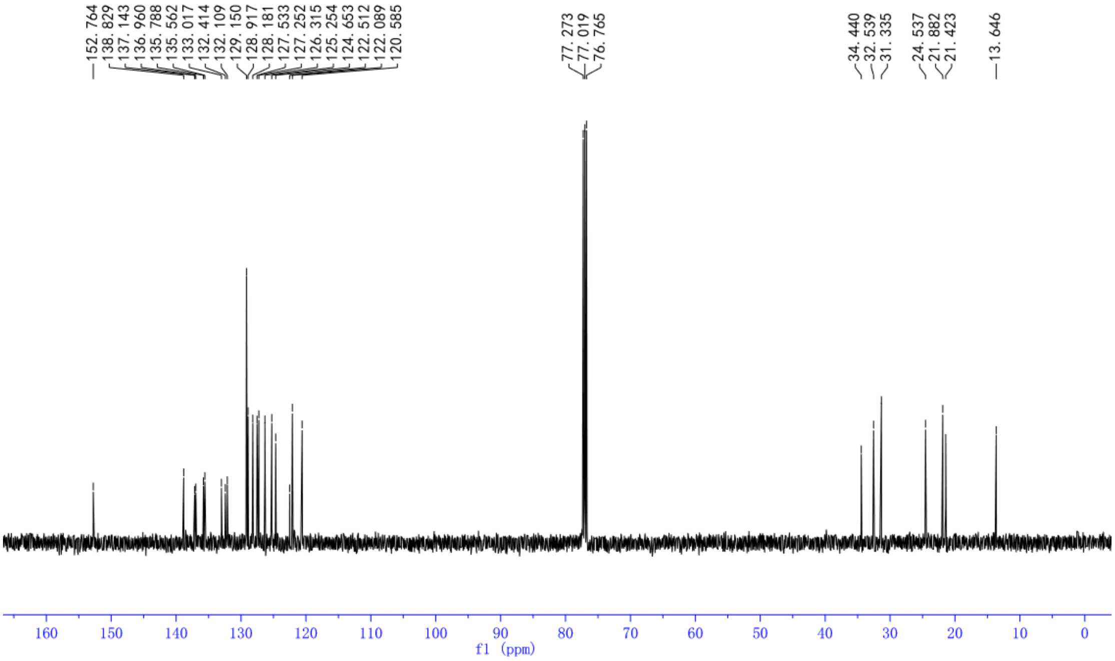

Supplementary Figure 155. ^{13}C NMR Spectrum of **9**

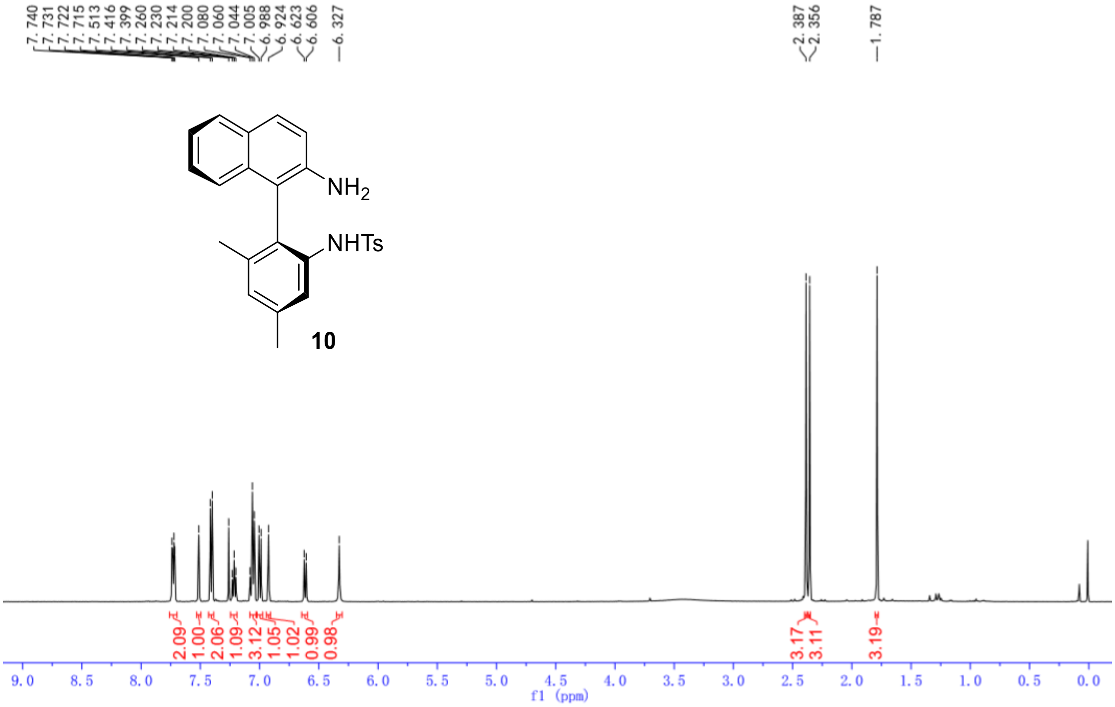

Supplementary Figure 156. ^{1}H NMR Spectrum of **10**

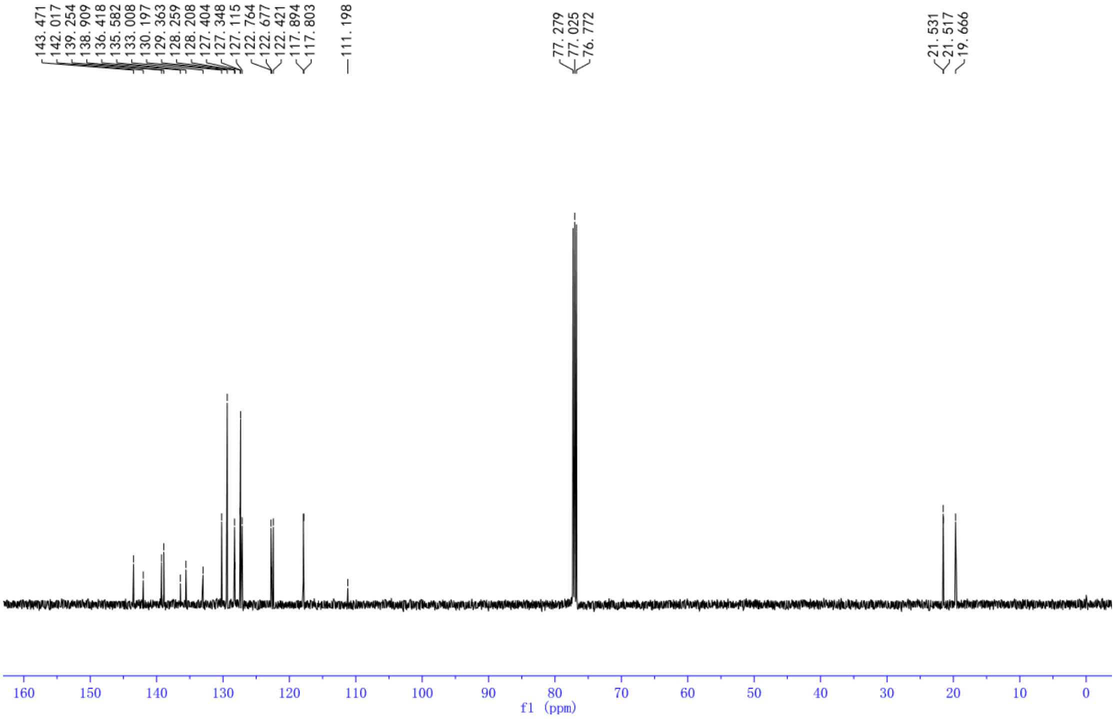

Supplementary Figure 157. ^{13}C NMR Spectrum of **10**

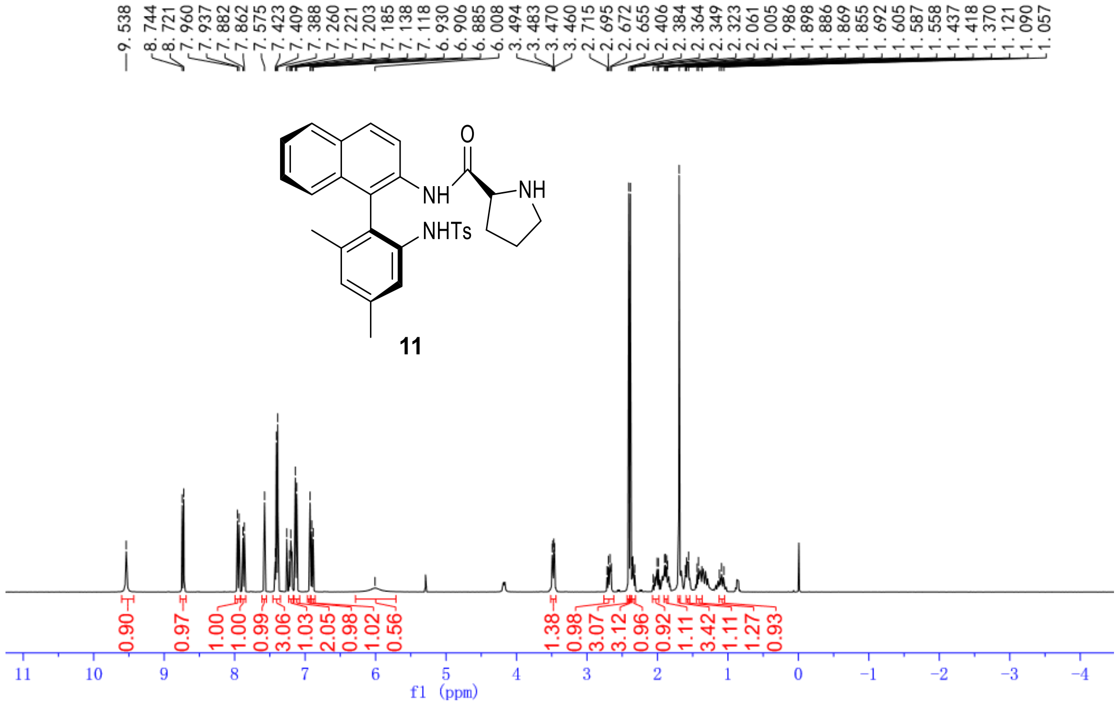

Supplementary Figure 158. ^{1}H NMR Spectrum of **11**

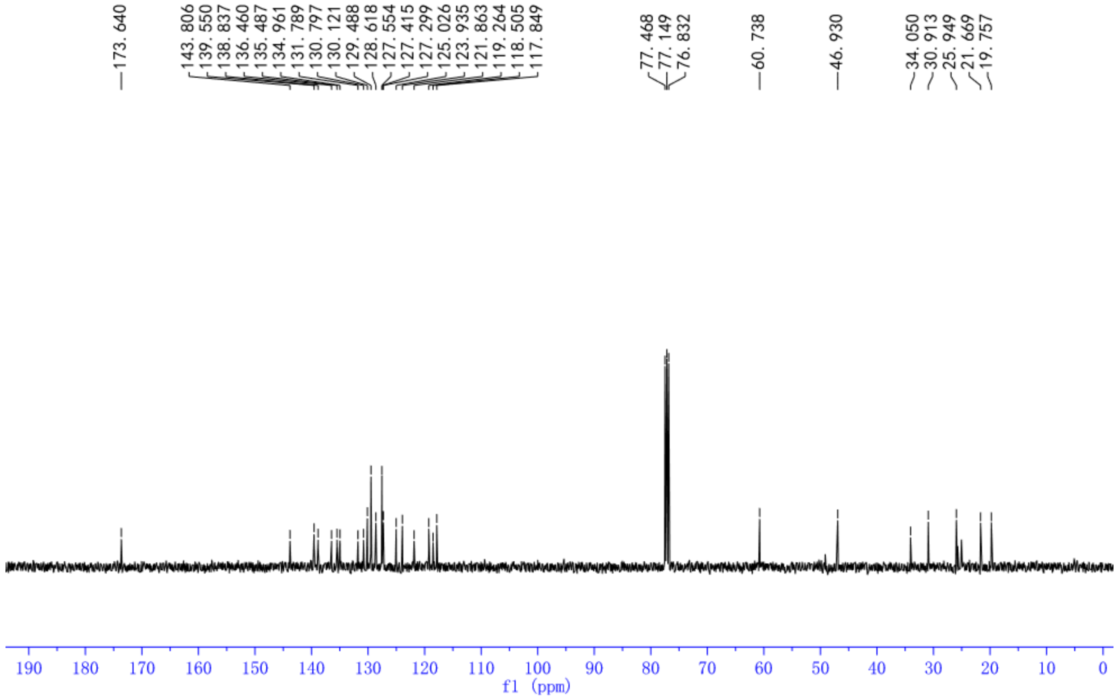

Supplementary Figure 159. ^{13}C NMR Spectrum of **11**

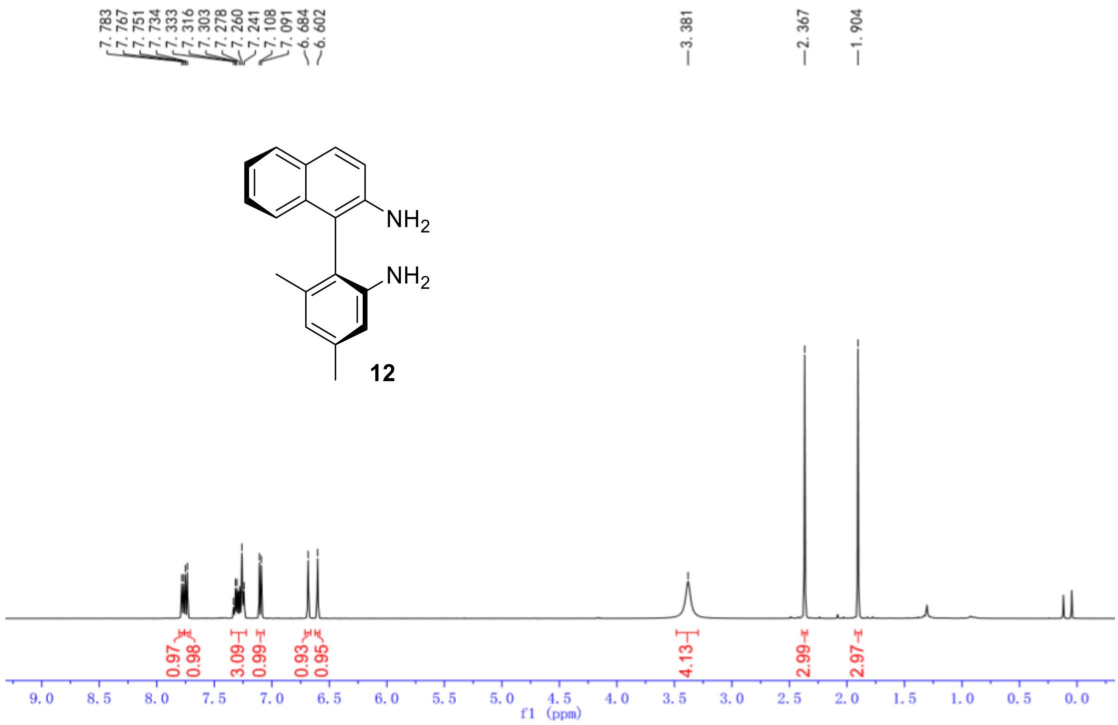

Supplementary Figure 160. ^{1}H NMR Spectrum of **12**

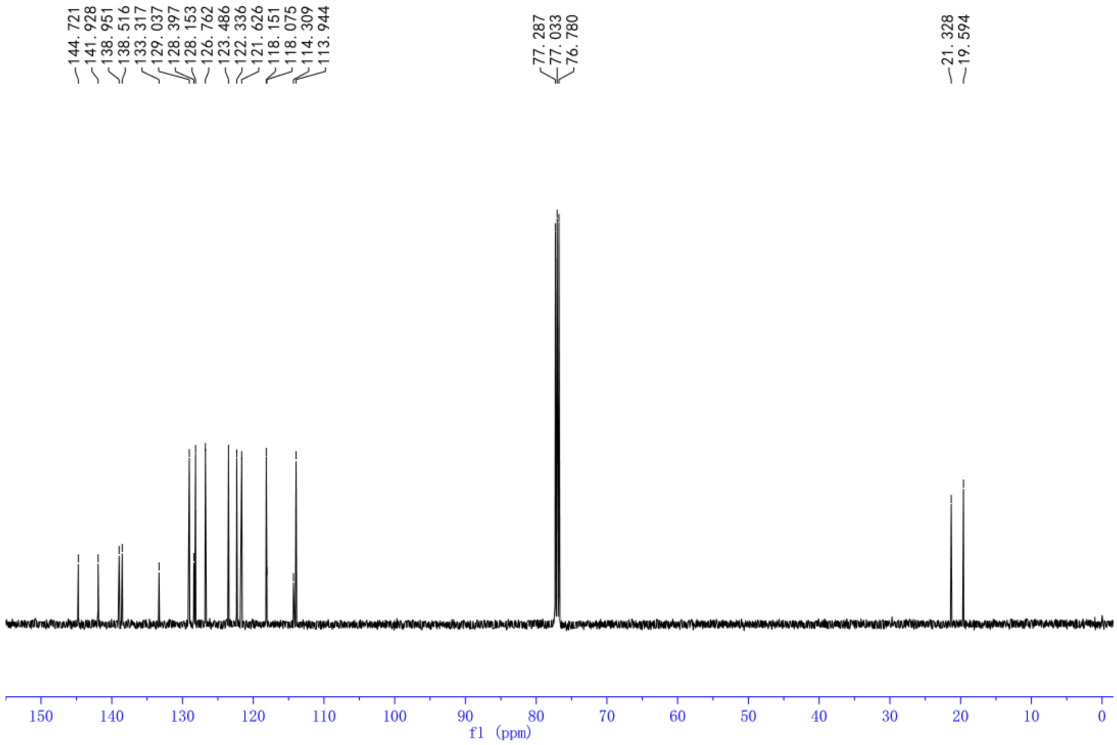

Supplementary Figure 161. ^{13}C NMR Spectrum of **12**

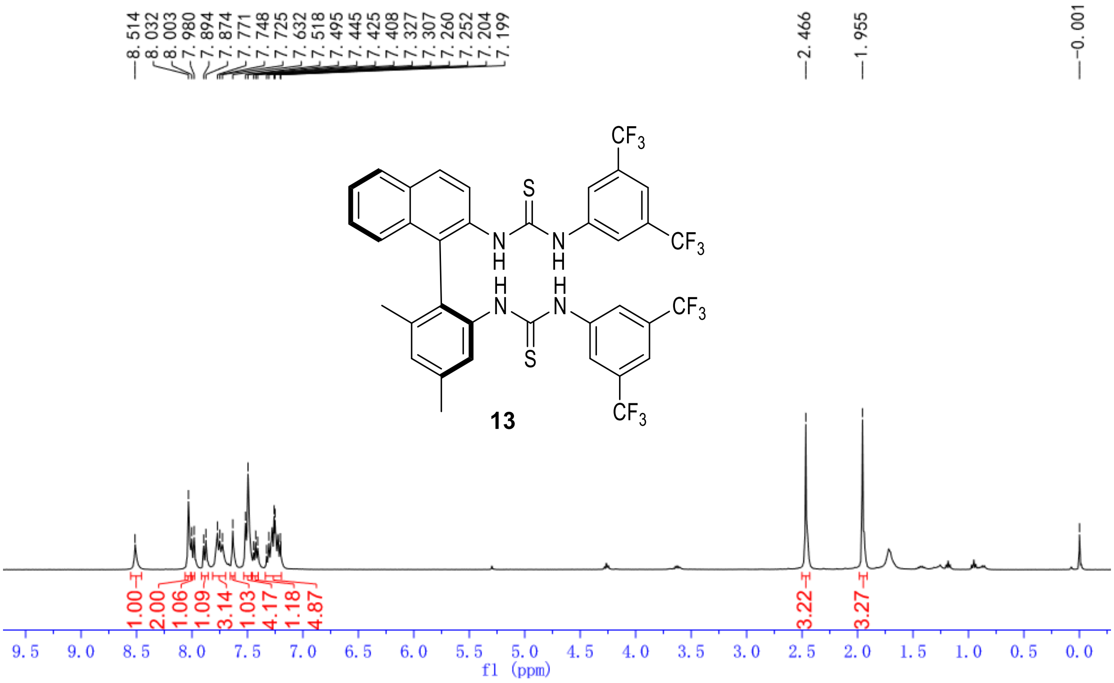

Supplementary Figure 162. ^{1}H NMR Spectrum of **13**

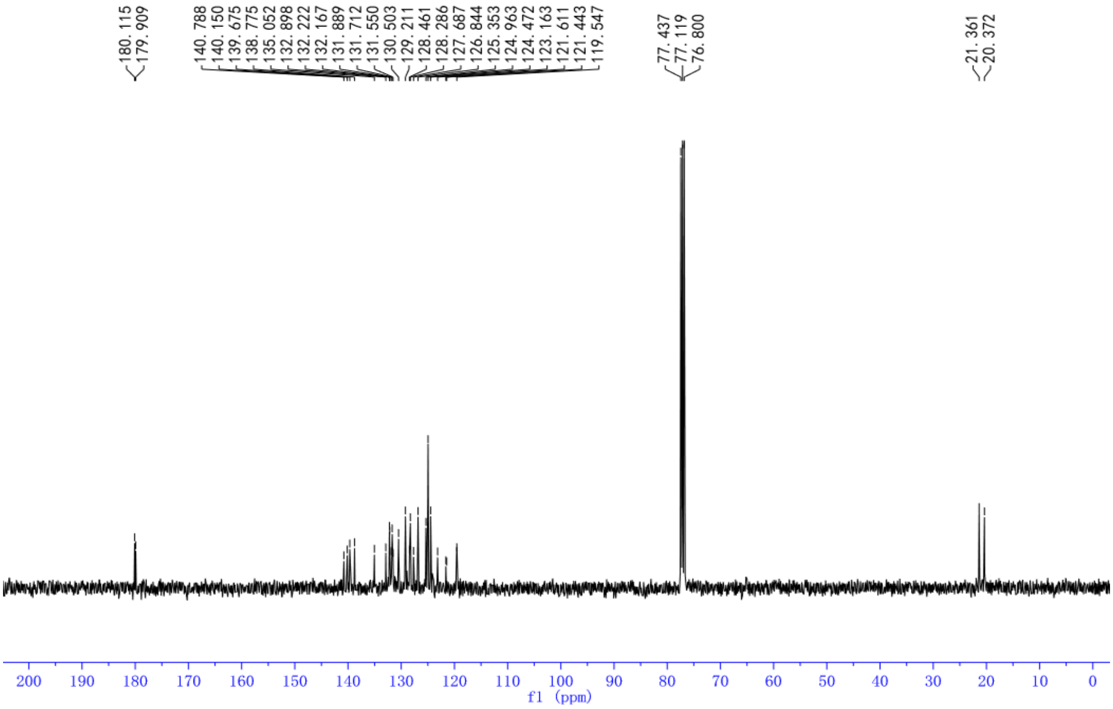

Supplementary Figure 163. ^{13}C NMR Spectrum of **13**

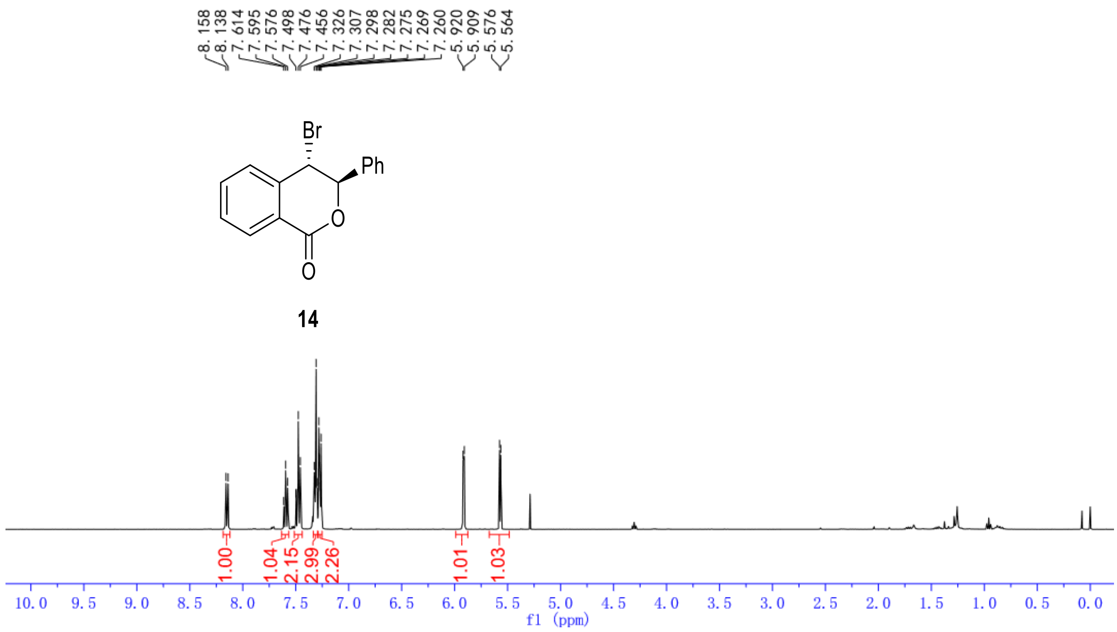

Supplementary Figure 164. ^{1}H NMR Spectrum of **14**

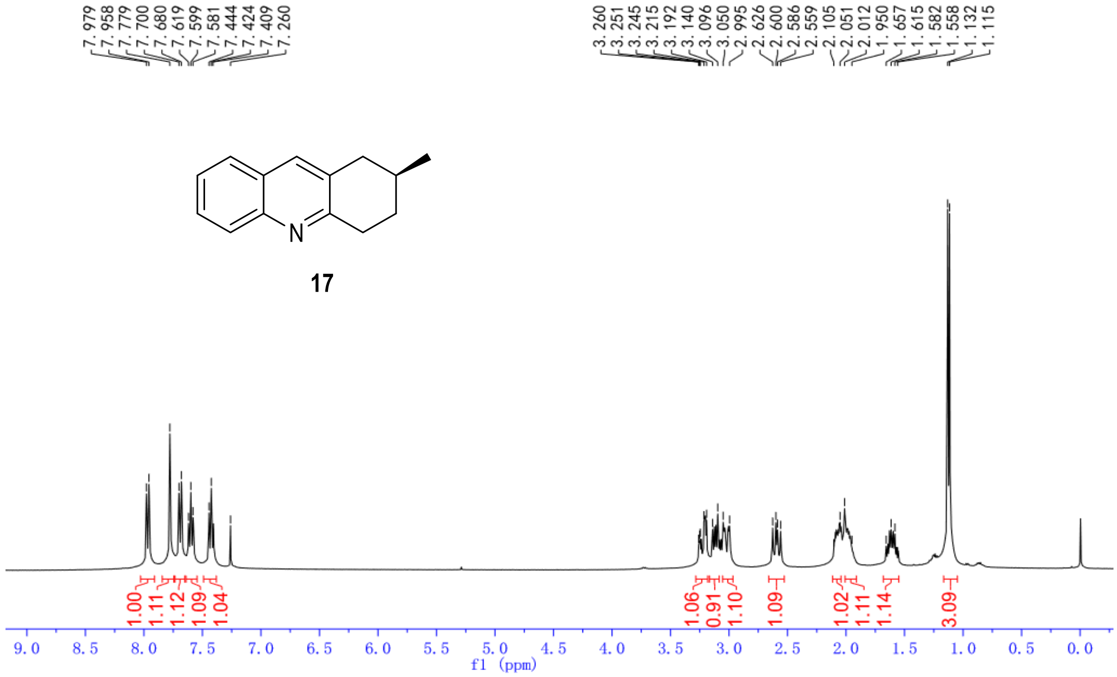

Supplementary Figure 165. ^{1}H NMR Spectrum of **17**

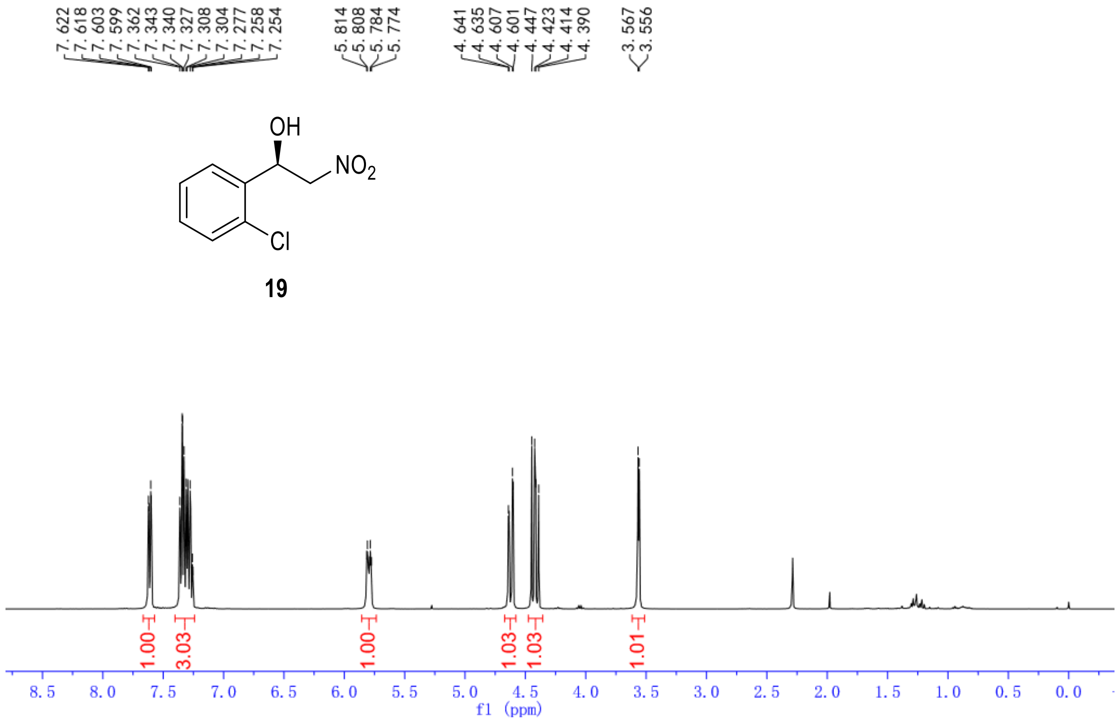

Supplementary Figure 166. ^{1}H NMR Spectrum of **19**

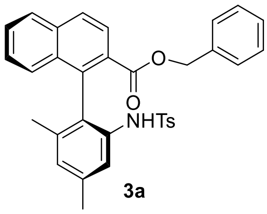

3a

<Chromatogram>

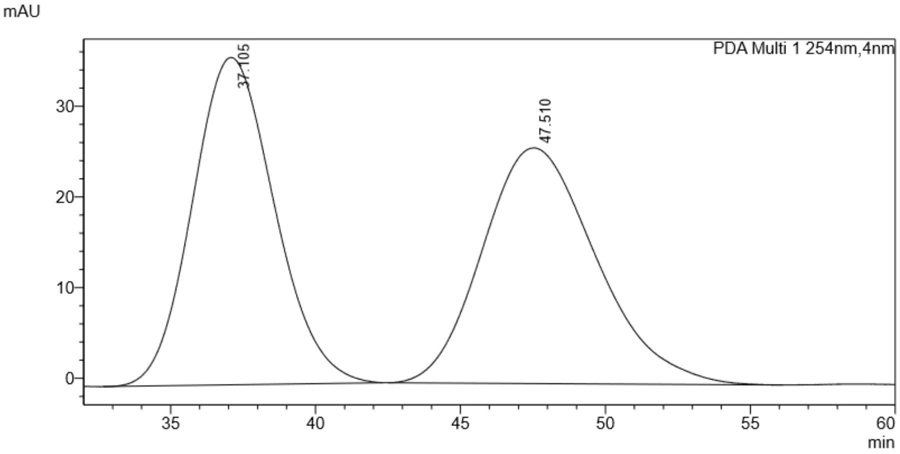

<Peak Table>

PDA Ch1 254nm

Peak#	Ret. Time	Area	Area%	Height
1	37.105	7095507	50.001	36092
2	47.510	7095100	49.999	25989
Total		14190607	100.000	62081

Supplementary Figure 167. HPLC Spectra of racemic **3a**

<Chromatogram>

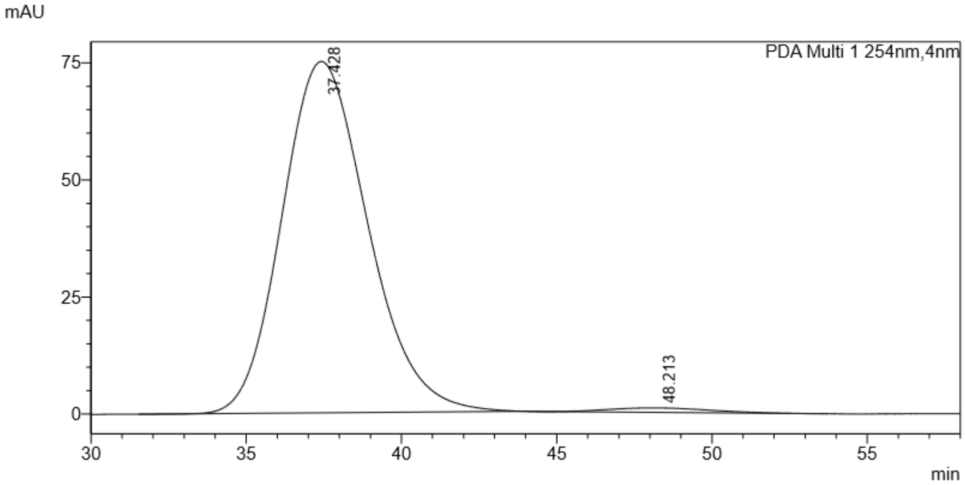

<Peak Table>

PDA Ch1 254nm

Peak#	Ret. Time	Area	Area%	Height
1	37.428	14456381	98.538	75039
2	48.213	214448	1.462	962
Total		14670828	100.000	76001

Supplementary Figure 168. HPLC Spectra of **3a**

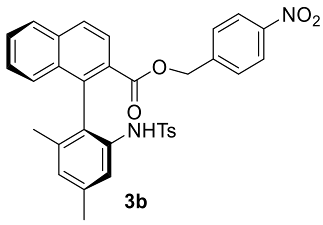

3b

<Chromatogram>

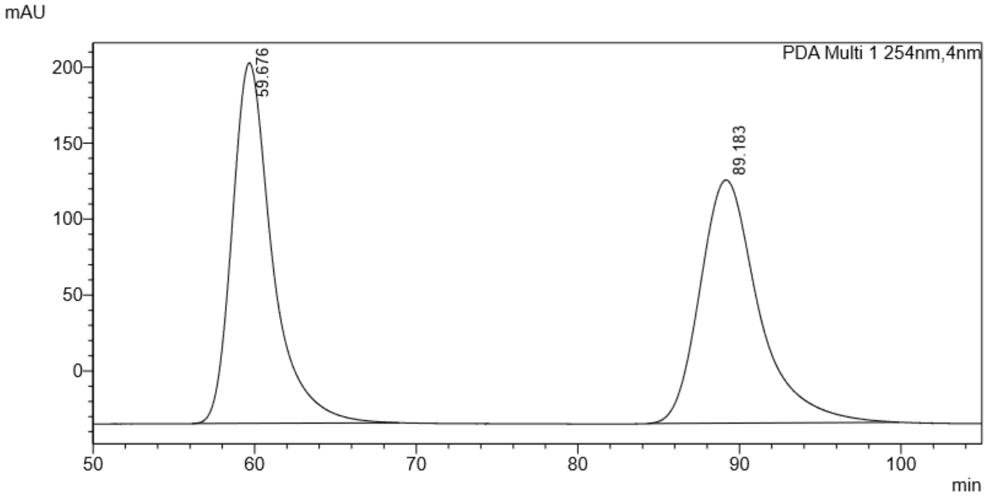

<Peak Table>

PDA Ch1 254nm

Peak#	Ret. Time	Area	Area%	Height
1	59.676	40898856	50.303	237335
2	89.183	40405967	49.697	160126
Total		81304823	100.000	397461

Supplementary Figure 169. HPLC Spectra of racemic **3b**

<Chromatogram>

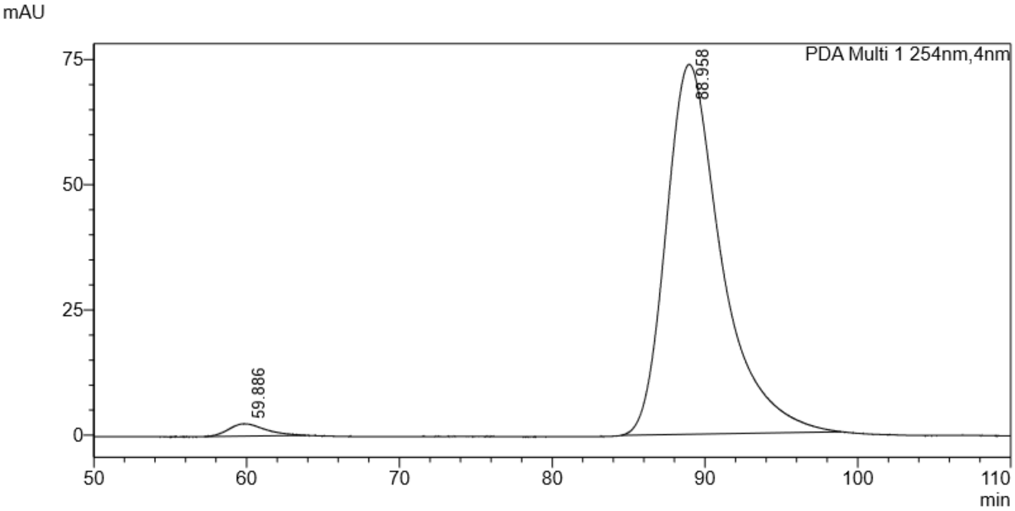

<Peak Table>

PDA Ch1 254nm

Peak#	Ret. Time	Area	Area%	Height
1	59.886	408580	2.144	2459
2	88.958	18651997	97.856	73842
Total		19060577	100.000	76300

Supplementary Figure 170. HPLC Spectra of **3b**

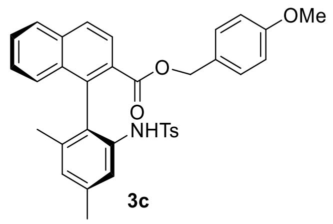

3c

<Chromatogram>

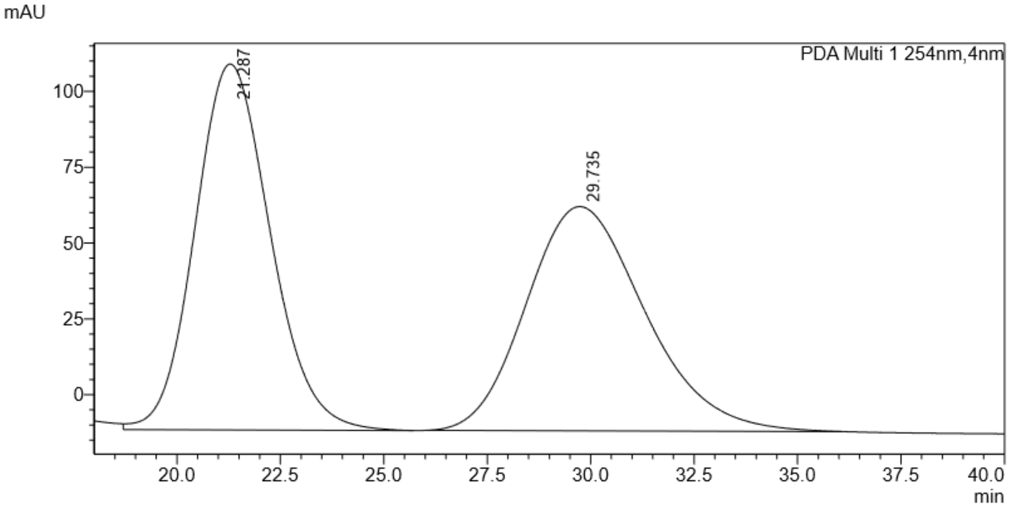

<Peak Table>

PDA Ch1 254nm

Peak#	Ret. Time	Area	Area%	Height
1	21.287	15318159	50.912	120680
2	29.735	14769177	49.088	73939
Total		30087336	100.000	194619

Supplementary Figure 171. HPLC Spectra of racemic **3c**

<Chromatogram>

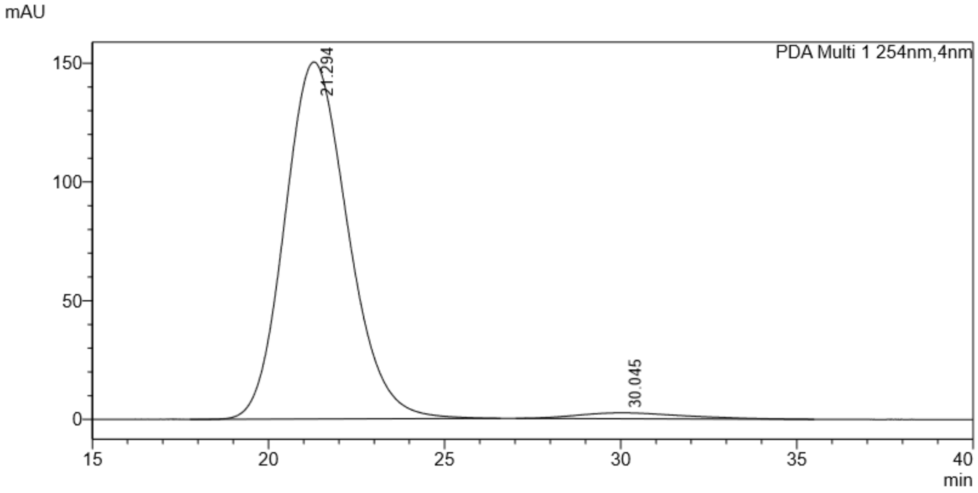

<Peak Table>

PDA Ch1 254nm

Peak#	Ret. Time	Area	Area%	Height
1	21.294	18753084	97.301	150272
2	30.045	520257	2.699	2474
Total		19273340	100.000	152746

Supplementary Figure 172. HPLC Spectra of **3c**

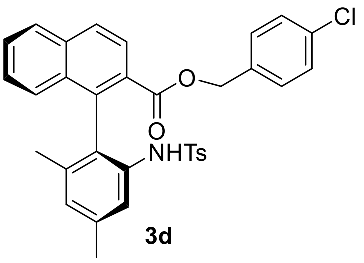

3d

<Chromatogram>

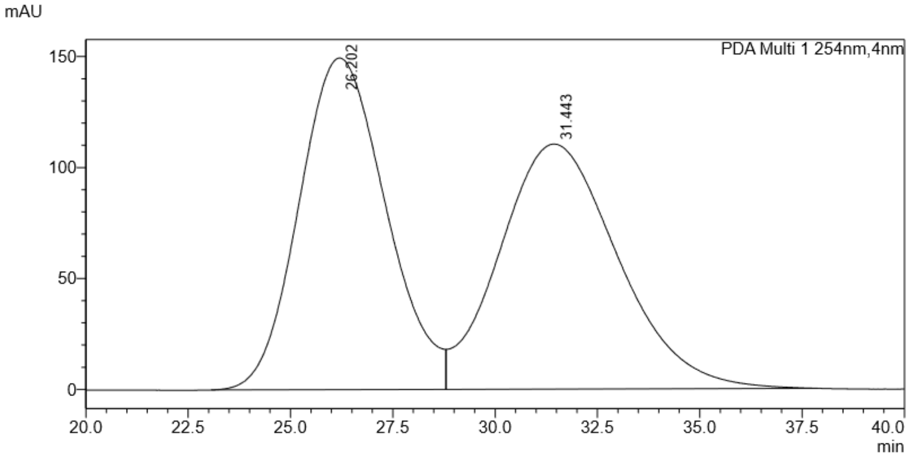

<Peak Table>

PDA Ch1 254nm

Peak#	Ret. Time	Area	Area%	Height
1	26.202	22041344	49.811	149382
2	31.443	22208965	50.189	110392
Total		44250309	100.000	259774

Supplementary Figure 173. HPLC Spectra of racemic **3d**

<Chromatogram>

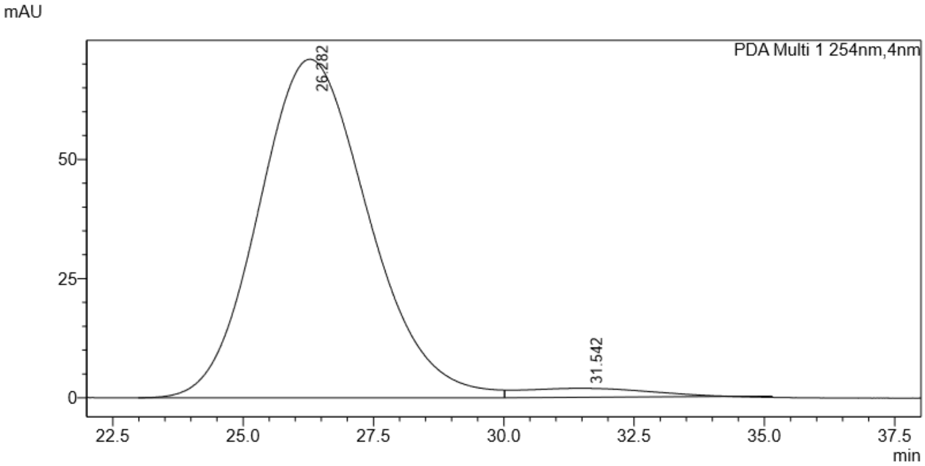

<Peak Table>

PDA Ch1 254nm

Peak#	Ret. Time	Area	Area%	Height
1	26.282	10417166	97.057	70993
2	31.542	315896	2.943	1901
Total		10733061	100.000	72894

Supplementary Figure 174. HPLC Spectra of **3d**

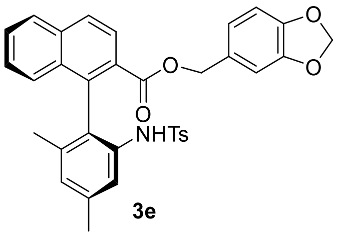

3e

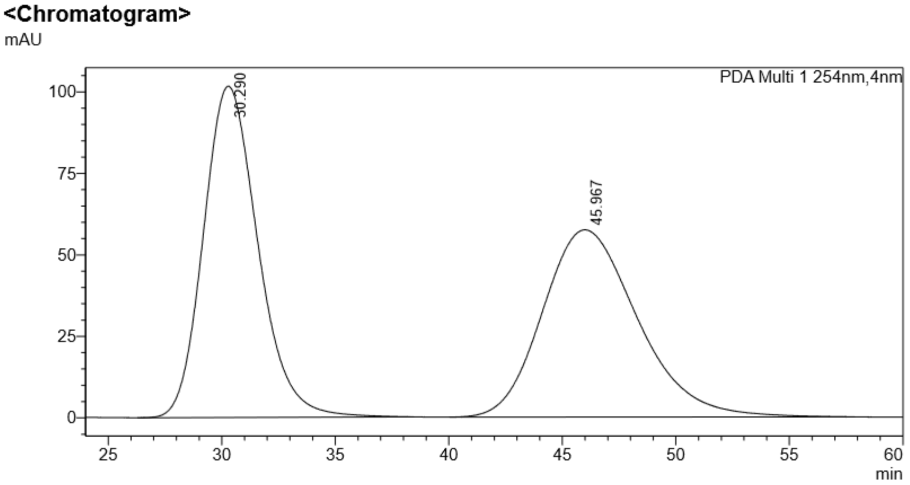

<Peak Table>

PDA Ch1 254nm

Peak#	Ret. Time	Area	Area%	Height
1	30.290	16961457	50.318	101638
2	45.967	16747050	49.682	57453
Total		33708506	100.000	159090

Supplementary Figure 175. HPLC Spectra of racemic **3e**

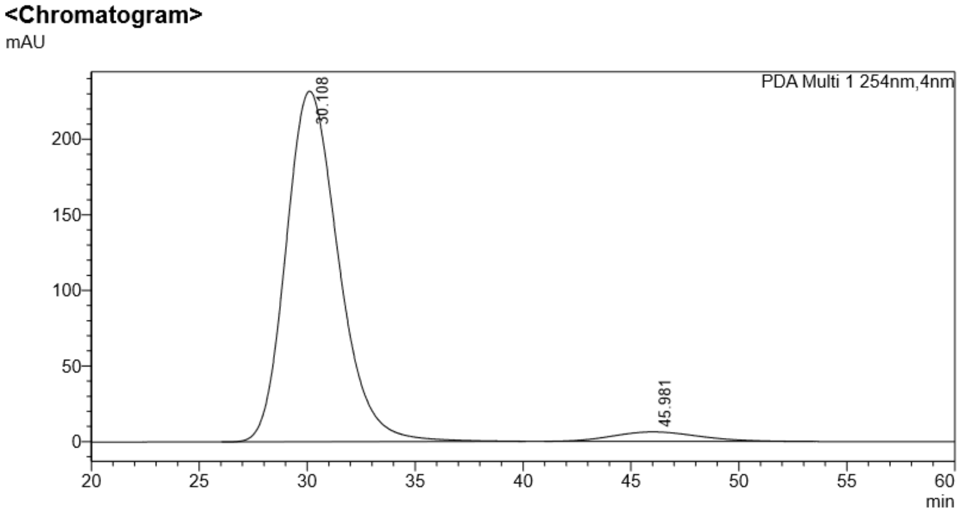

<Peak Table>

PDA Ch1 254nm

Peak#	Ret. Time	Area	Area%	Height
1	30.108	39142249	95.707	231801
2	45.981	1755815	4.293	6305
Total		40898064	100.000	238106

Supplementary Figure 176. HPLC Spectra of **3e**

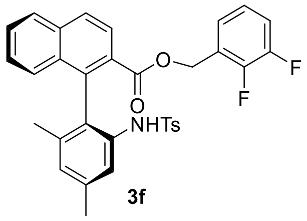

3f

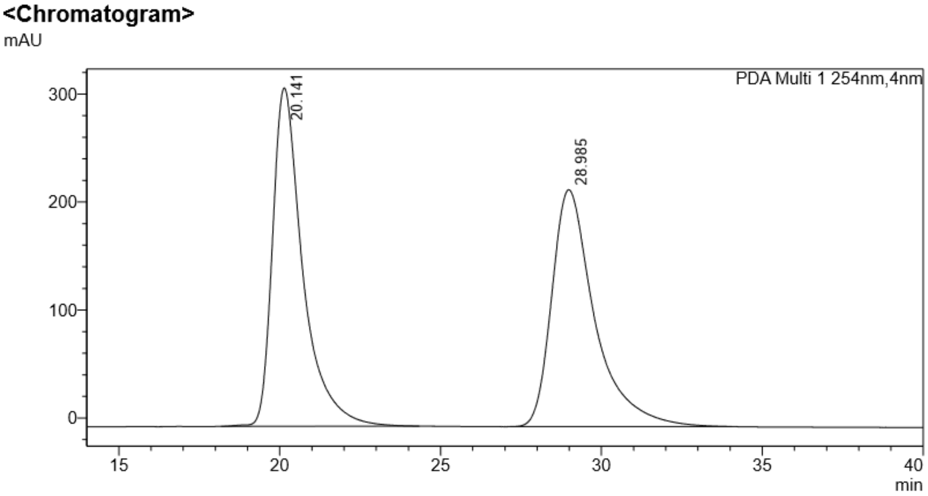

<Peak Table>

PDA Ch1 254nm

Peak#	Ret. Time	Area	Area%	Height
1	20.141	20513303	50.200	313302
2	28.985	20349871	49.800	219390
Total		40863174	100.000	532691

Supplementary Figure 177. HPLC Spectra of racemic **3f**

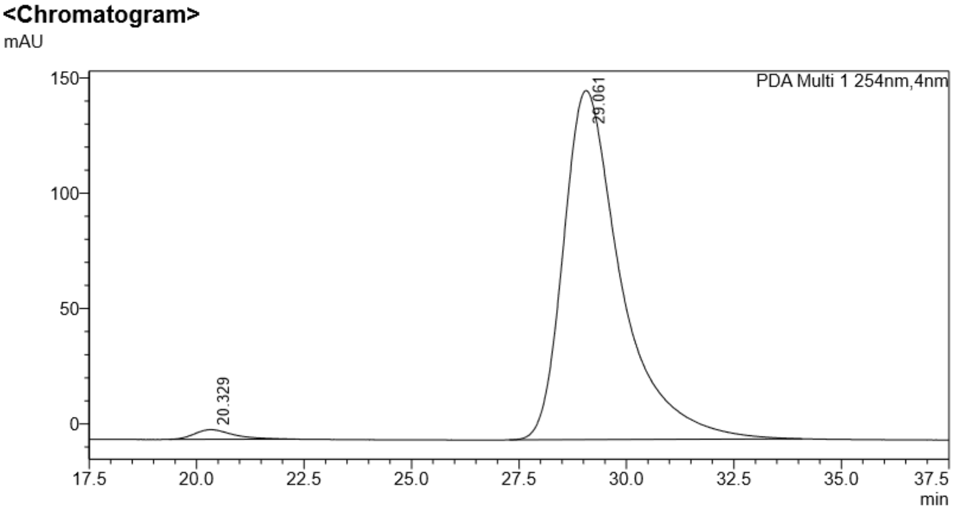

<Peak Table>

PDA Ch1 254nm

Peak#	Ret. Time	Area	Area%	Height
1	20.329	264194	1.836	4179
2	29.061	14122650	98.164	151307
Total		14386844	100.000	155485

Supplementary Figure 178. HPLC Spectra of **3f**

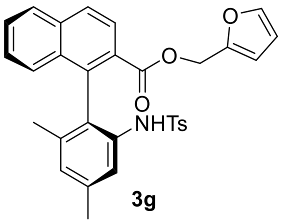

3g

<Chromatogram>

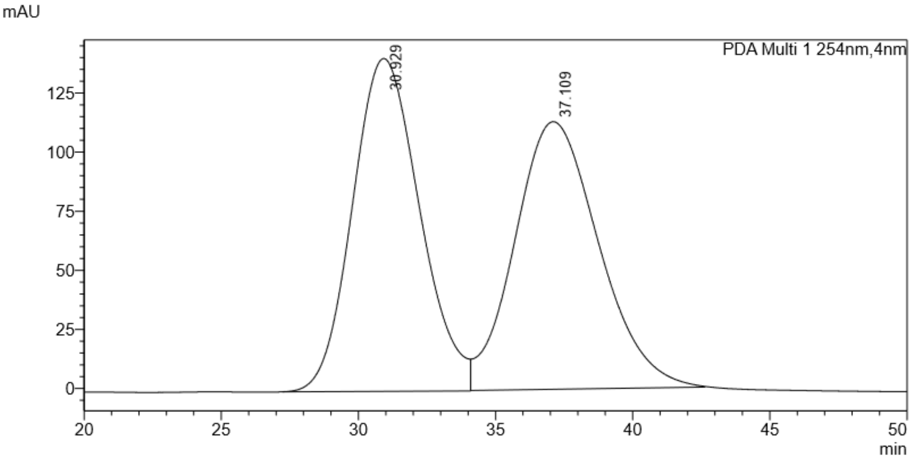

<Peak Table>

PDA Ch1 254nm

Peak#	Ret. Time	Area	Area%	Height
1	30.929	24006017	49.897	140772
2	37.109	24105541	50.103	113173
Total		48111558	100.000	253945

Supplementary Figure 179. HPLC Spectra of racemic **3g**

<Chromatogram>

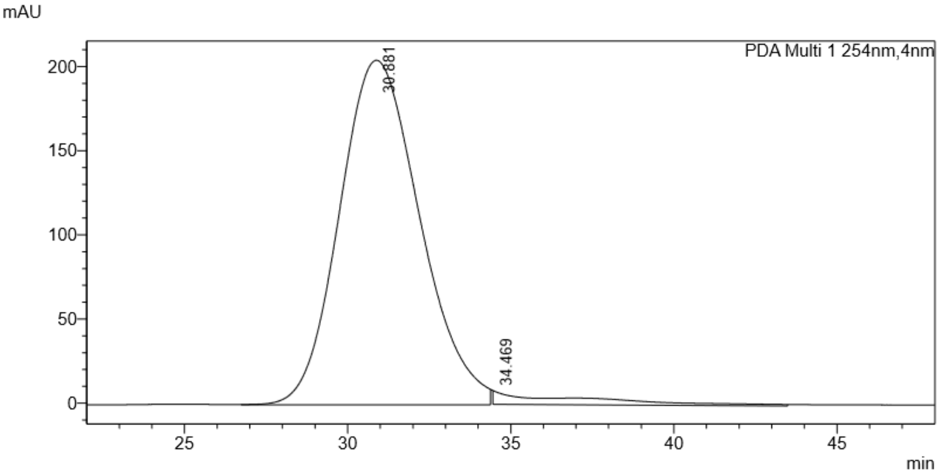

<Peak Table>

PDA Ch1 254nm

Peak#	Ret. Time	Area	Area%	Height
1	30.881	35042113	96.051	204702
2	34.469	1440741	3.949	8149
Total		36482854	100.000	212851

Supplementary Figure 180. HPLC Spectra of **3g**

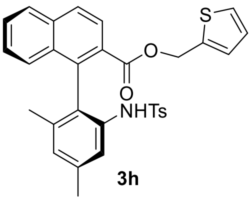

3h

<Chromatogram>

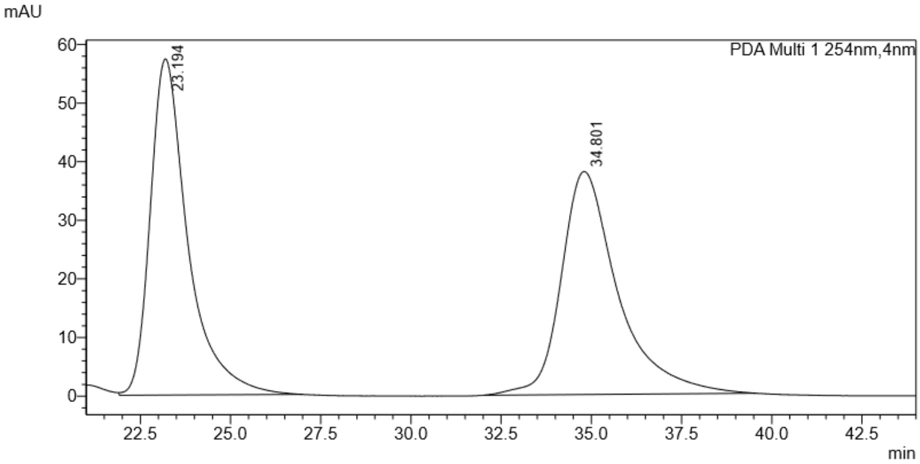

<Peak Table>

PDA Ch1 254nm

Peak#	Ret. Time	Area	Area%	Height
1	23.194	4160348	49.975	57354
2	34.801	4164566	50.025	38044
Total		8324914	100.000	95398

Supplementary Figure 181. HPLC Spectra of racemic **3h**

<Chromatogram>

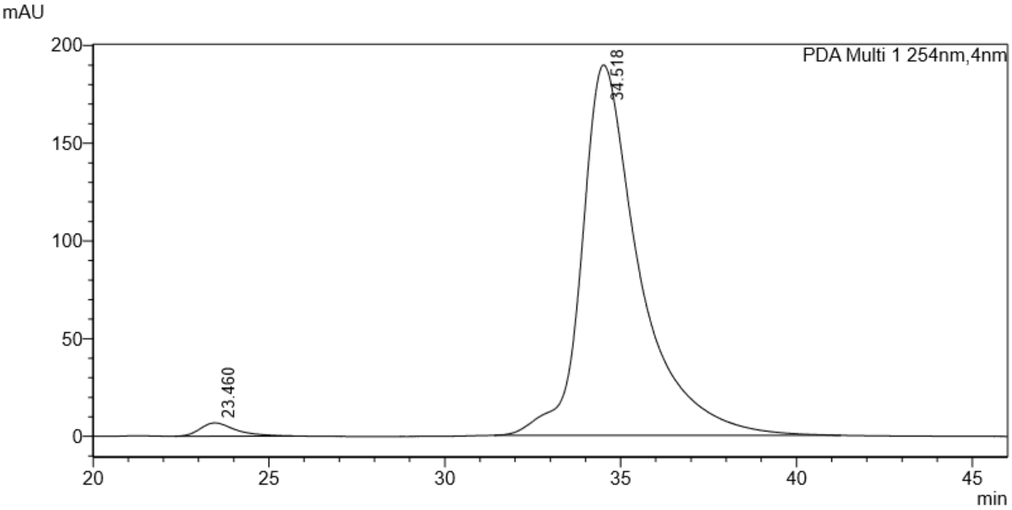

<Peak Table>

PDA Ch1 254nm

Peak#	Ret. Time	Area	Area%	Height
1	23.460	472248	2.138	6761
2	34.518	21618867	97.862	189496
Total		22091114	100.000	196257

Supplementary Figure 182. HPLC Spectra of **3h**

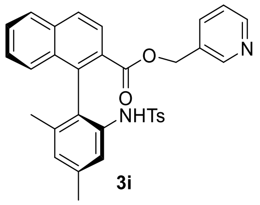

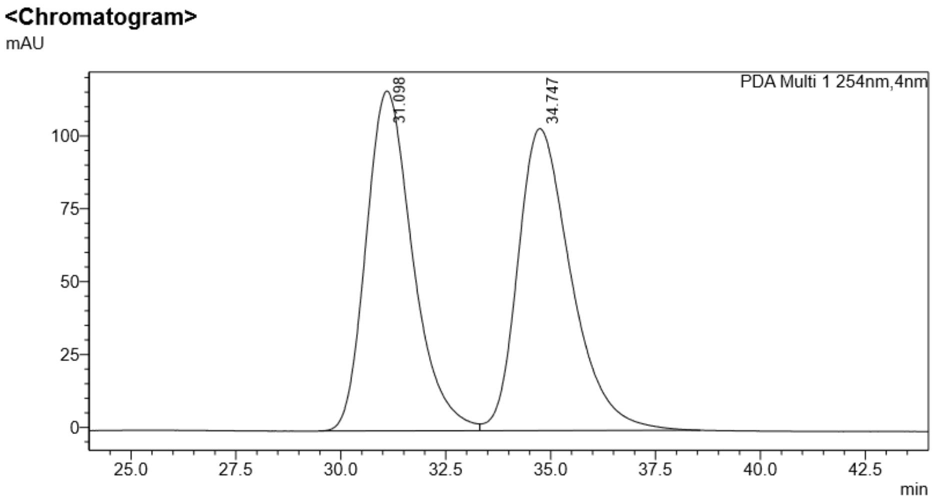

<Peak Table>

PDA Ch1 254nm

Peak#	Ret. Time	Area	Area%	Height
1	31.098	9008987	49.621	116586
2	34.747	9146590	50.379	103591
Total		18155578	100.000	220176

Supplementary Figure 183. HPLC Spectra of racemic **3i**

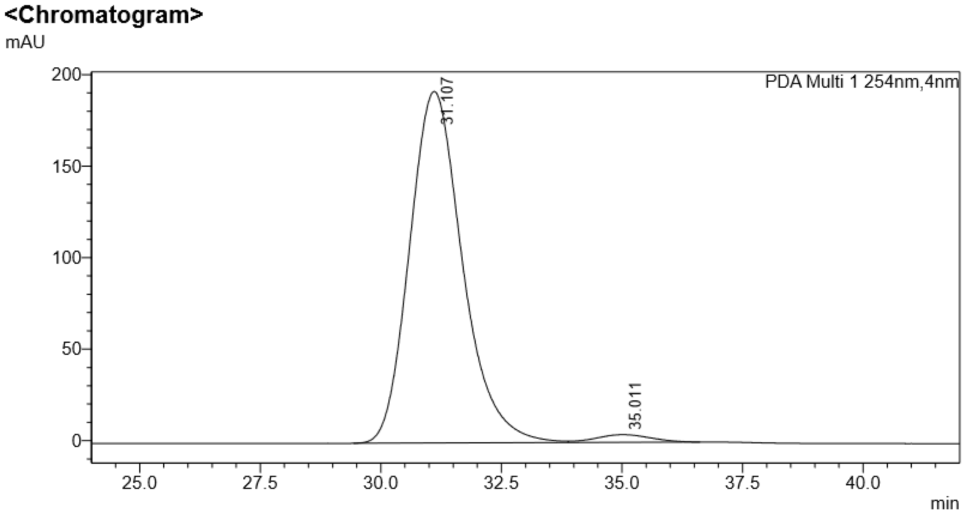

<Peak Table>

PDA Ch1 254nm

Peak#	Ret. Time	Area	Area%	Height
1	31.107	14691833	97.785	192074
2	35.011	332738	2.215	4222
Total		15024572	100.000	196297

Supplementary Figure 184. HPLC Spectra of **3i**

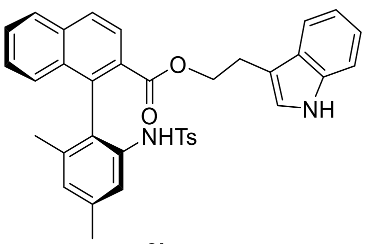

3j

<Chromatogram>

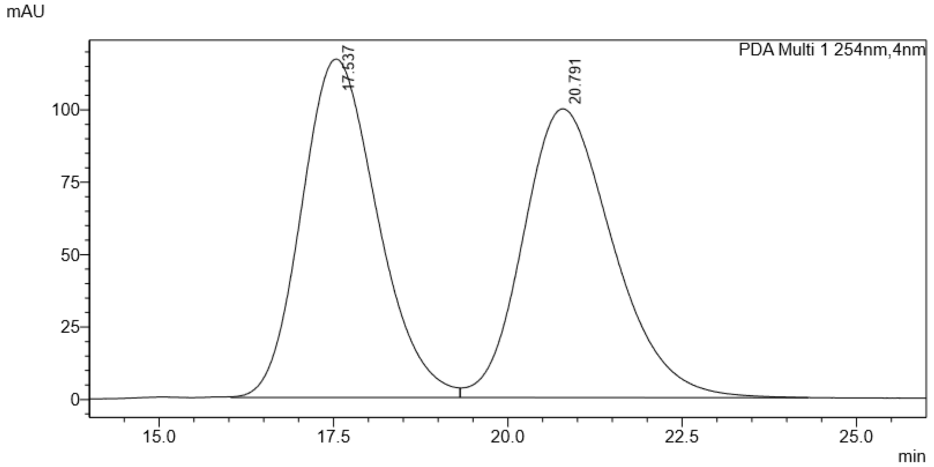

<Peak Table>

PDA Ch1 254nm

Peak#	Ret. Time	Area	Area%	Height
1	17.537	8835600	49.680	116820
2	20.791	8949388	50.320	99688
Total		17784988	100.000	216509

Supplementary Figure 185. HPLC Spectra of racemic **3j**

<Chromatogram>

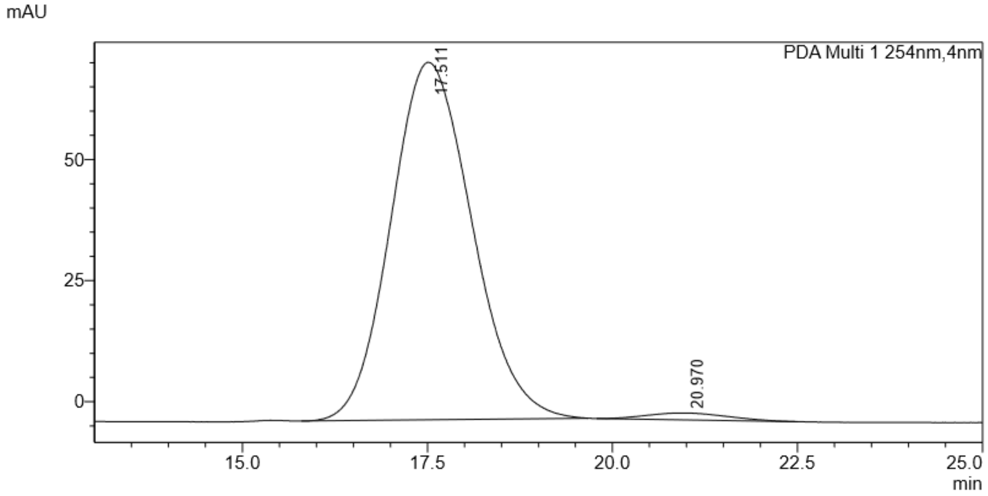

<Peak Table>

PDA Ch1 254nm

Peak#	Ret. Time	Area	Area%	Height
1	17.511	5631649	98.118	73817
2	20.970	108017	1.882	1423
Total		5739667	100.000	75241

Supplementary Figure 186. HPLC Spectra of **3j**

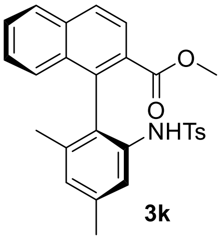

3k

<Chromatogram>

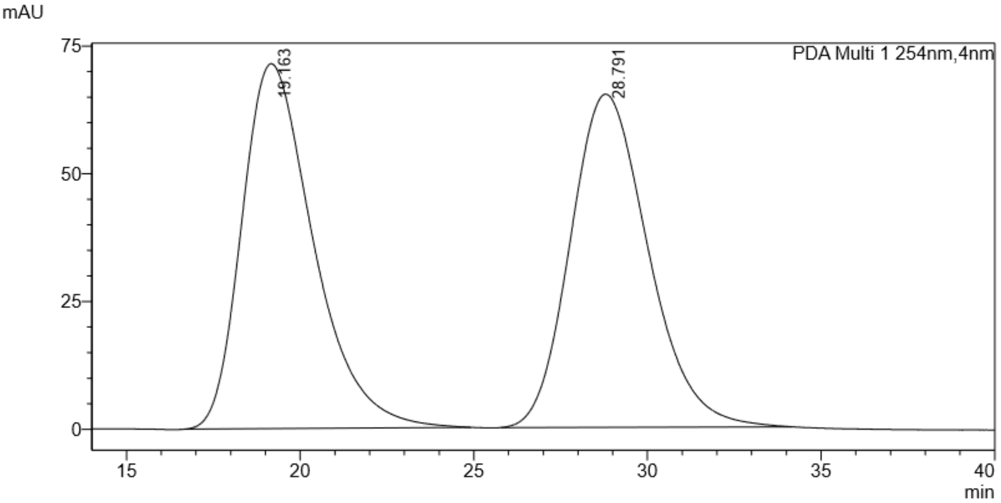

<Peak Table>

PDA Ch1 254nm

Peak#	Ret. Time	Area	Area%	Height
1	19.163	10245797	50.142	71441
2	28.791	10187739	49.858	65199
Total		20433536	100.000	136639

Supplementary Figure 187. HPLC Spectra of racemic **3k**

<Chromatogram>

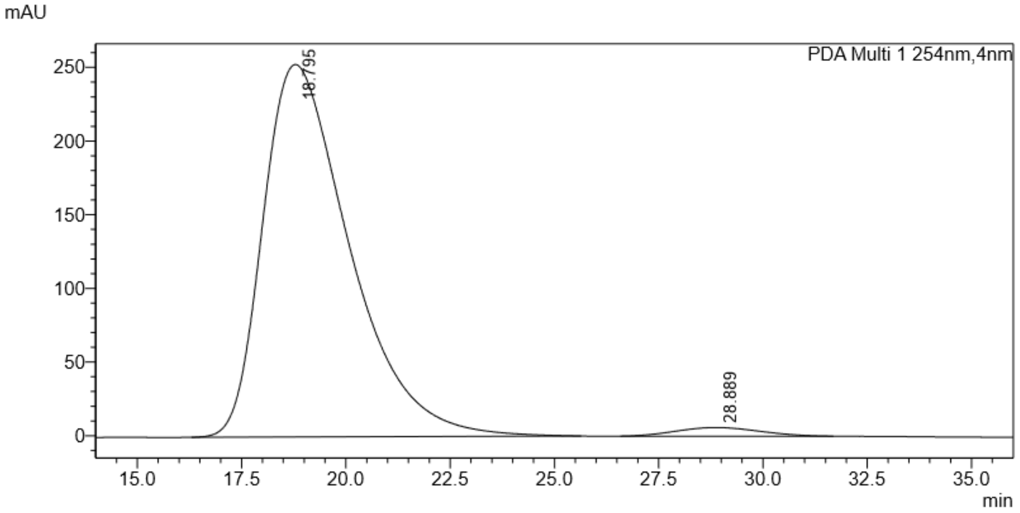

<Peak Table>

PDA Ch1 254nm

Peak#	Ret. Time	Area	Area%	Height
1	18.795	36605683	97.808	252767
2	28.889	820429	2.192	5882
Total		37426113	100.000	258648

Supplementary Figure 188. HPLC Spectra of **3k**

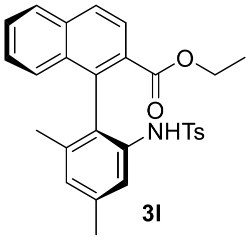

3l

<Chromatogram>

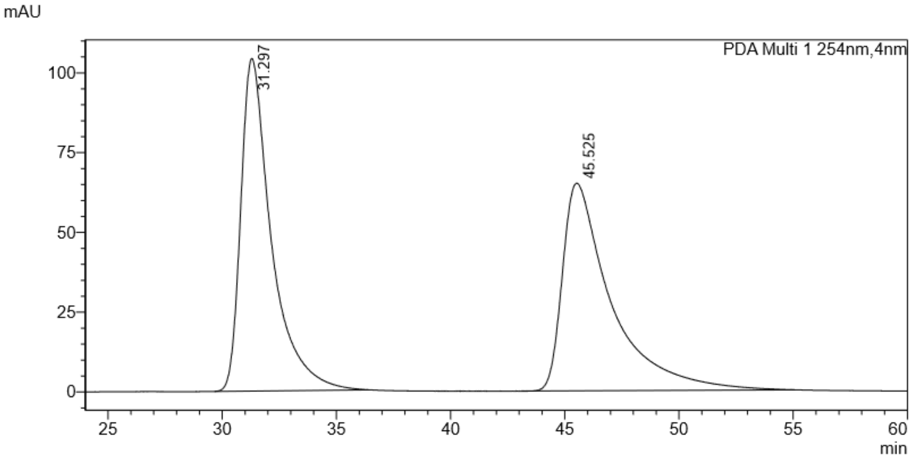

<Peak Table>

PDA Ch1 254nm

Peak#	Ret. Time	Area	Area%	Height
1	31.297	9690881	50.078	104194
2	45.525	9660884	49.922	65070
Total		19351765	100.000	169264

Supplementary Figure 189. HPLC Spectra of racemic **3l**

<Chromatogram>

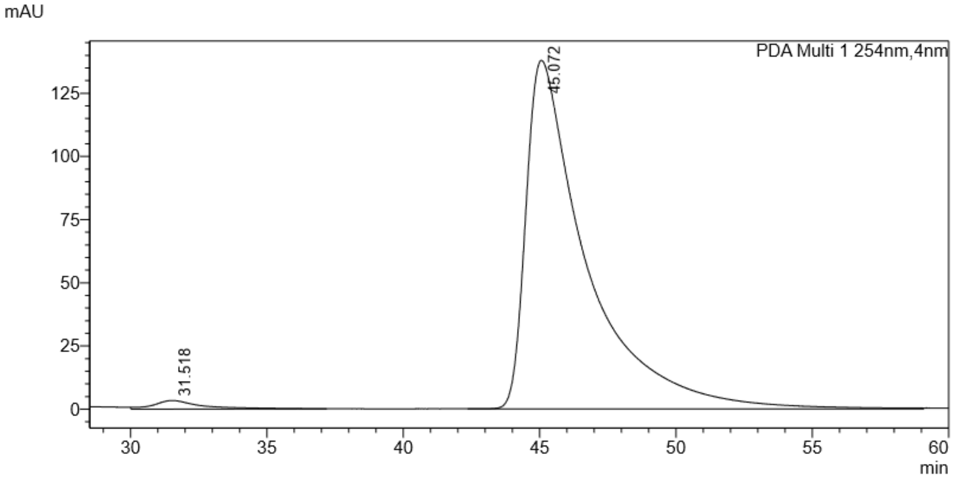

<Peak Table>

PDA Ch1 254nm

Peak#	Ret. Time	Area	Area%	Height
1	31.518	430652	1.972	3385
2	45.072	21407441	98.028	137920
Total		21838093	100.000	141306

Supplementary Figure 190. HPLC Spectra of **3l**

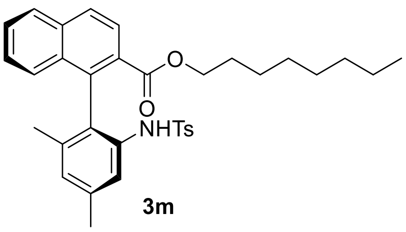

3m

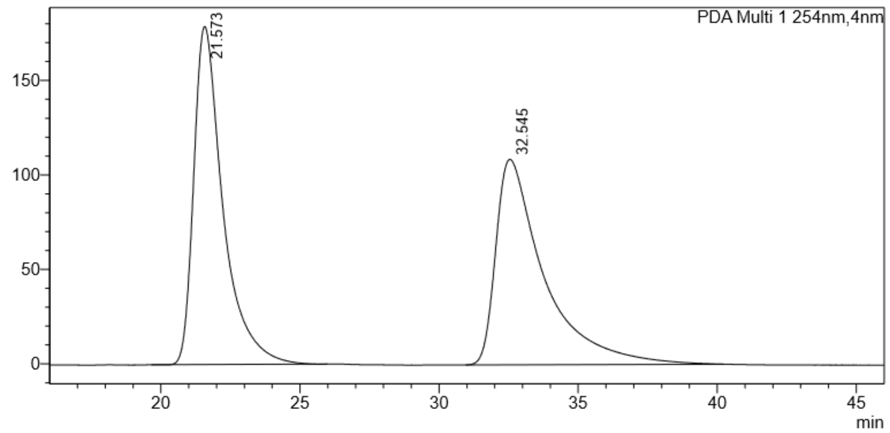

<Peak Table>

PDA Ch1 254nm

Peak#	Ret. Time	Area	Area%	Height
1	21.573	13069137	49.933	178862
2	32.545	13104311	50.067	108715
Total		26173448	100.000	287577

Supplementary Figure 191. HPLC Spectra of racemic **3m**

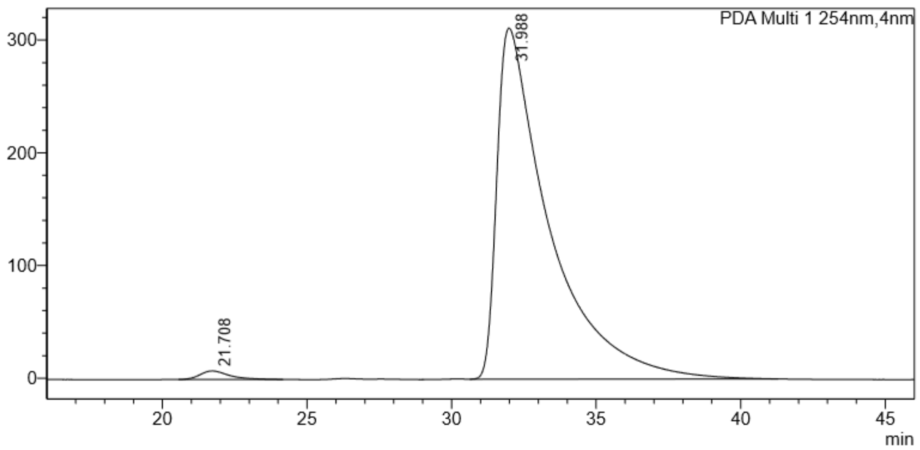

<Peak Table>

PDA Ch1 254nm

Peak#	Ret. Time	Area	Area%	Height
1	21.708	531648	1.342	7547
2	31.988	39073925	98.658	311399
Total		39605573	100.000	318946

Supplementary Figure 192. HPLC Spectra of **3m**

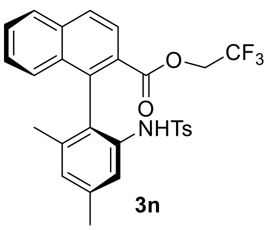

3n

<Chromatogram>

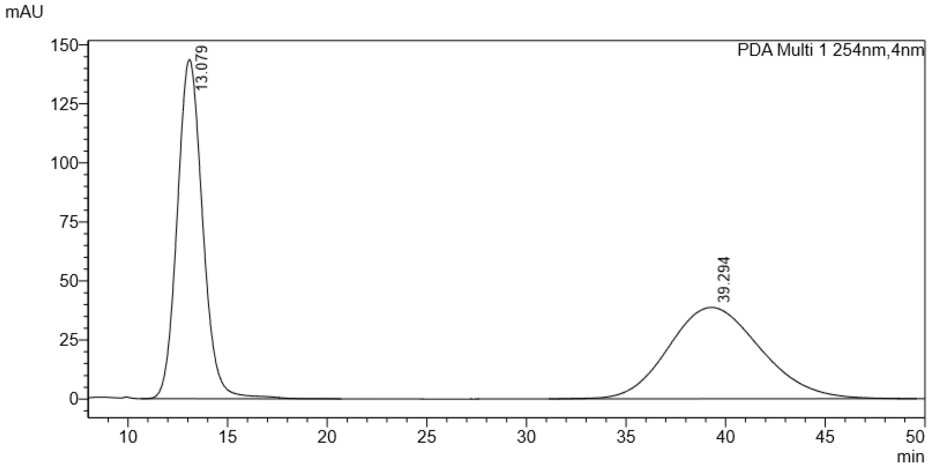

<Peak Table>

PDA Ch1 254nm

Peak#	Ret. Time	Area	Area%	Height
1	13.079	12728919	50.476	143631
2	39.294	12488816	49.524	38692
Total		25217735	100.000	182323

Supplementary Figure 193. HPLC Spectra of racemic **3n**

<Chromatogram>

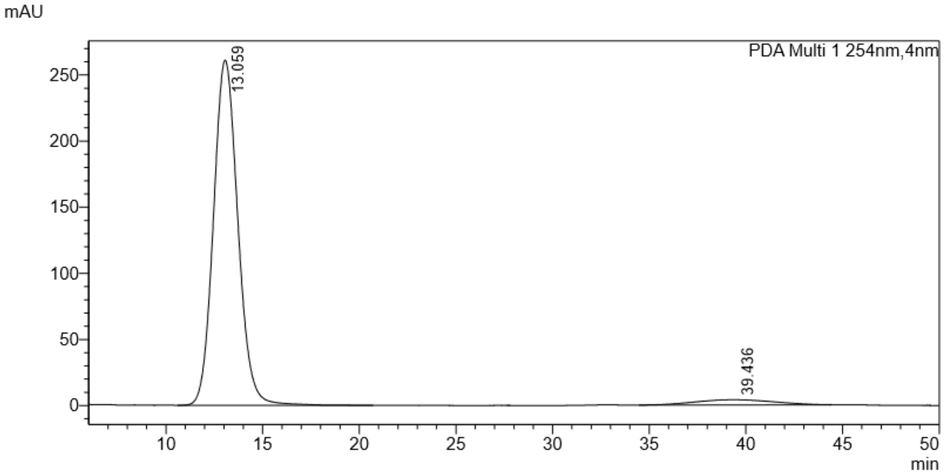

<Peak Table>

PDA Ch1 254nm

Peak#	Ret. Time	Area	Area%	Height
1	13.059	22888971	95.399	260950
2	39.436	1103925	4.601	3938
Total		23992896	100.000	264888

Supplementary Figure 194. HPLC Spectra of **3n**

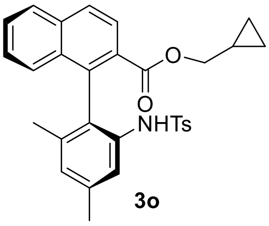

3o

<Chromatogram>

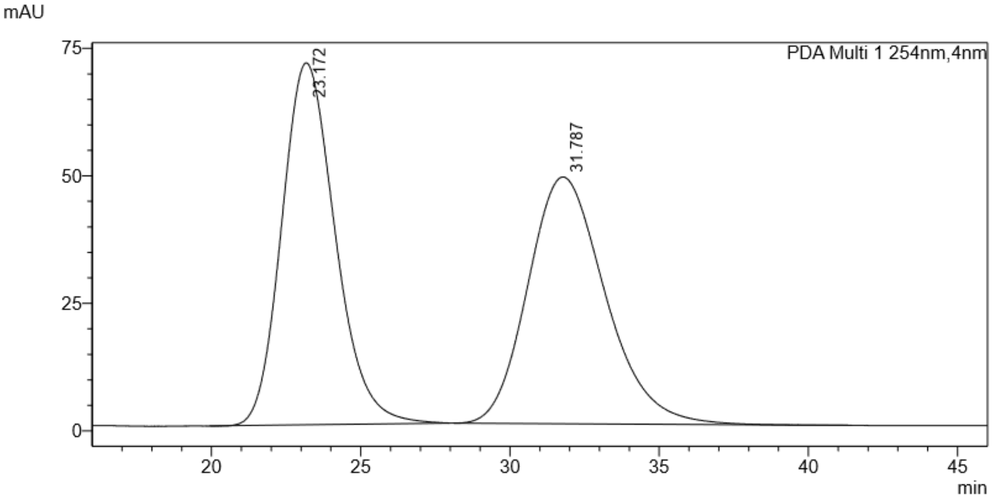

<Peak Table>

PDA Ch1 254nm

Peak#	Ret. Time	Area	Area%	Height
1	23.172	8846096	50.290	70969
2	31.787	8744220	49.710	48386
Total		17590316	100.000	119355

Supplementary Figure 195. HPLC Spectra of racemic **3o**

<Chromatogram>

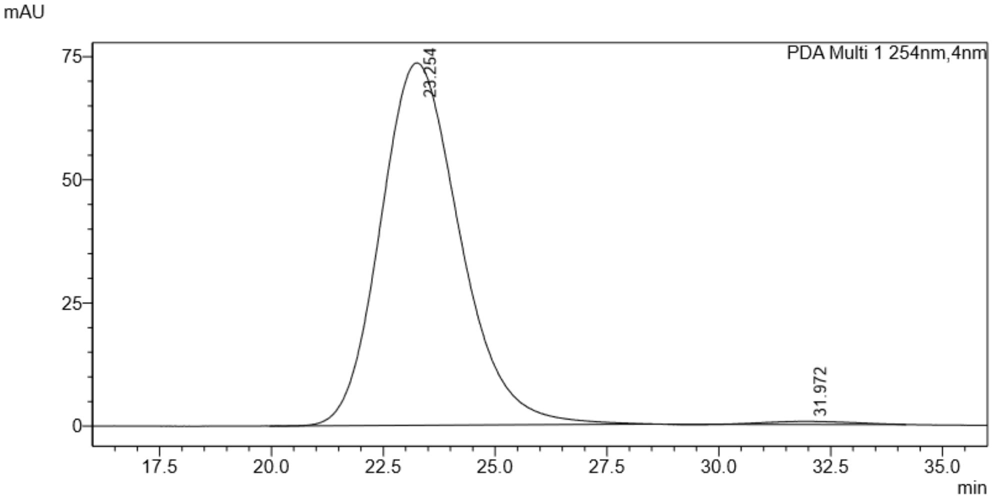

<Peak Table>

PDA Ch1 254nm

Peak#	Ret. Time	Area	Area%	Height
1	23.254	9092503	99.082	73522
2	31.972	84227	0.918	626
Total		9176729	100.000	74147

Supplementary Figure 196. HPLC Spectra of **3o**

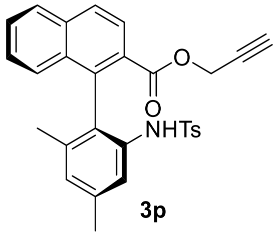

3p

<Chromatogram>

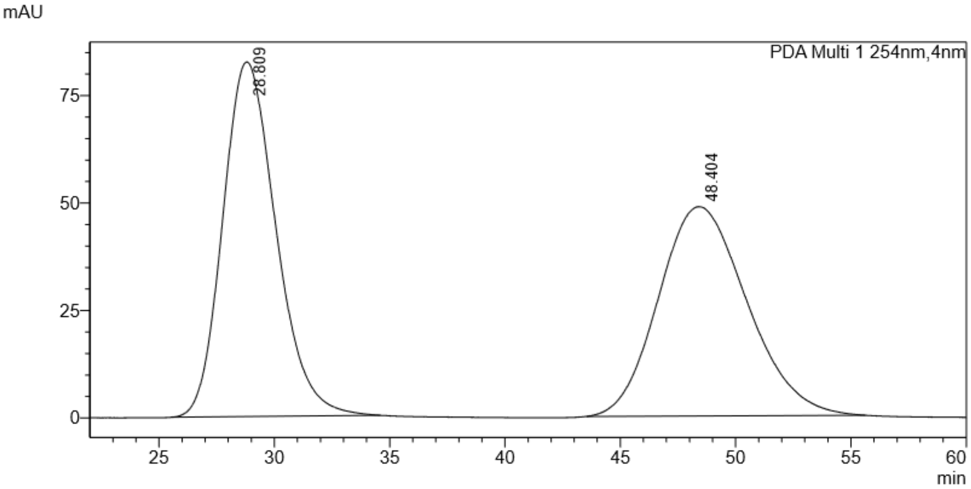

<Peak Table>

PDA Ch1 254nm

Peak#	Ret. Time	Area	Area%	Height
1	28.809	13246986	50.440	82510
2	48.404	13015733	49.560	48681
Total		26262719	100.000	131191

Supplementary Figure 197. HPLC Spectra of racemic **3p**

<Chromatogram>

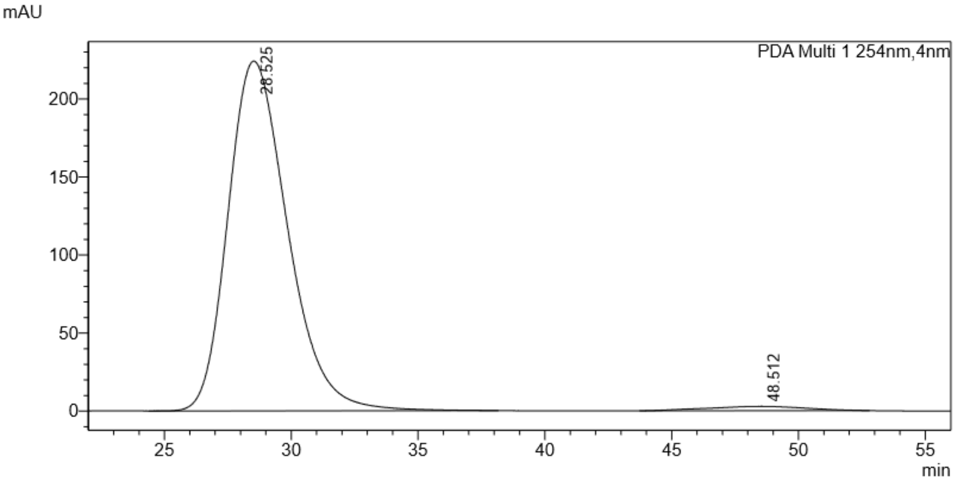

<Peak Table>

PDA Ch1 254nm

Peak#	Ret. Time	Area	Area%	Height
1	28.525	36655142	98.005	224043
2	48.512	746329	1.995	2746
Total		37401471	100.000	226789

Supplementary Figure 198. HPLC Spectra of **3p**

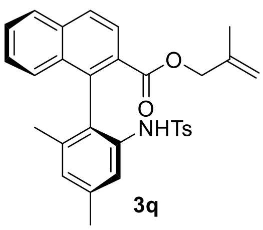

3q

<Chromatogram>

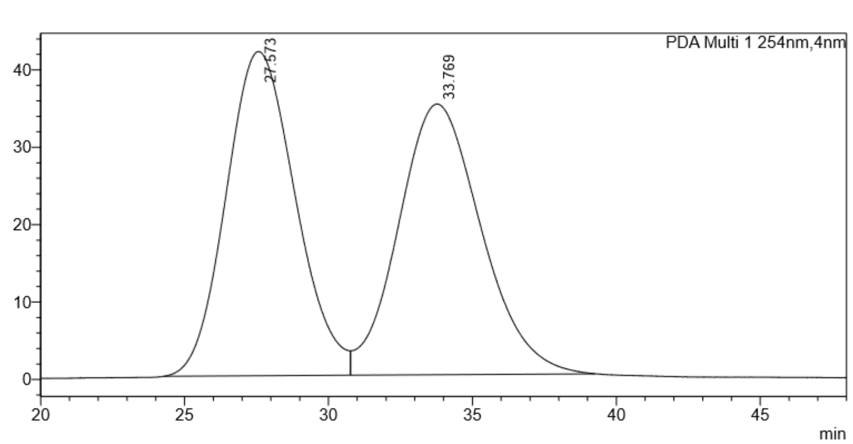

<Peak Table>

PDA Ch1 254nm

Peak#	Ret. Time	Area	Area%	Height
1	27.573	7019381	50.116	41910
2	33.769	6986949	49.884	34990
Total		14006329	100.000	76899

Supplementary Figure 199. HPLC Spectra of racemic **3q**

<Chromatogram>

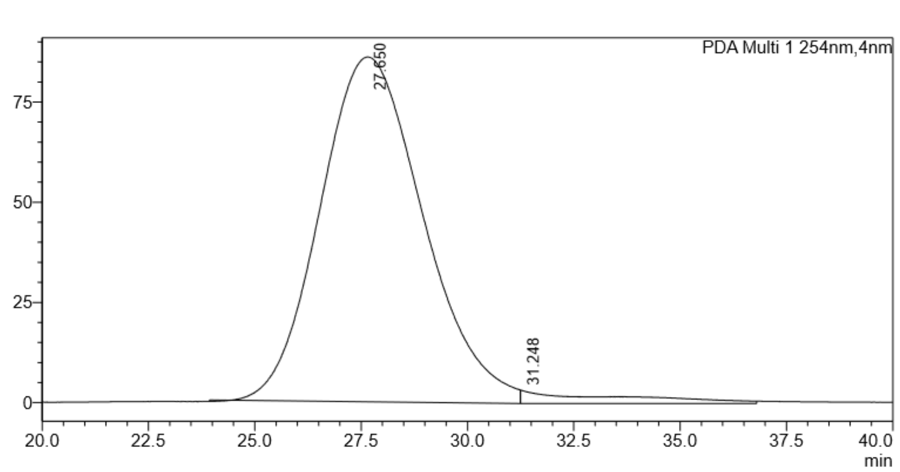

<Peak Table>

PDA Ch1 254nm

Peak#	Ret. Time	Area	Area%	Height
1	27.650	14456738	96.654	86001
2	31.248	500399	3.346	3368
Total		14957138	100.000	89369

Supplementary Figure 200. HPLC Spectra of **3q**

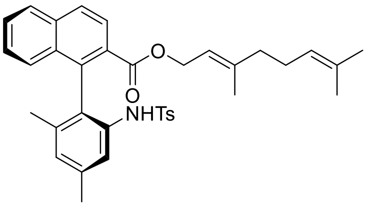

3r

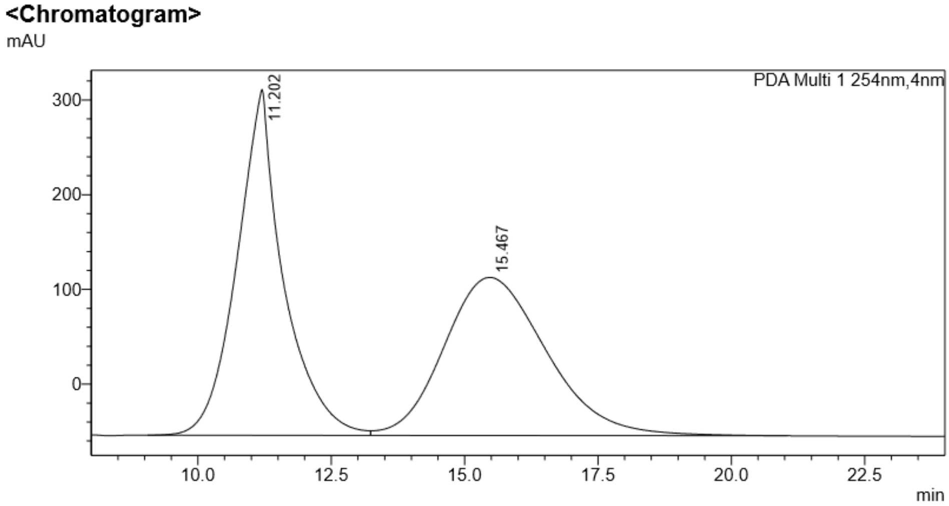

<Peak Table>

PDA Ch1 254nm

Peak#	Ret. Time	Area	Area%	Height
1	11.202	22143622	49.756	364872
2	15.467	22360930	50.244	166882
Total		44504552	100.000	531754

Supplementary Figure 201. HPLC Spectra of racemic **3r**

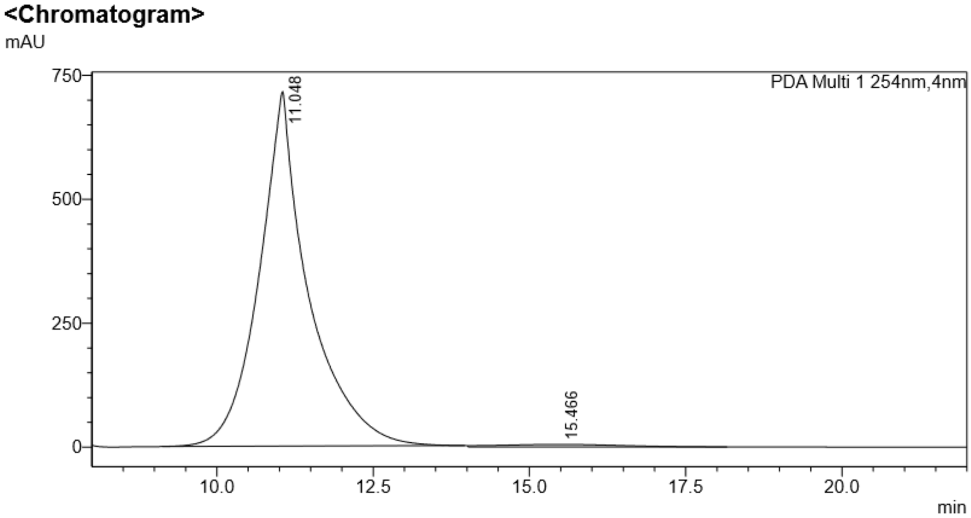

<Peak Table>

PDA Ch1 254nm

Peak#	Ret. Time	Area	Area%	Height
1	11.048	39110480	98.220	714618
2	15.466	708859	1.780	5187
Total		39819339	100.000	719805

Supplementary Figure 202. HPLC Spectra of **3r**

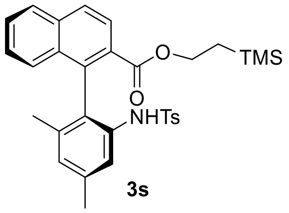

3s

<Chromatogram>

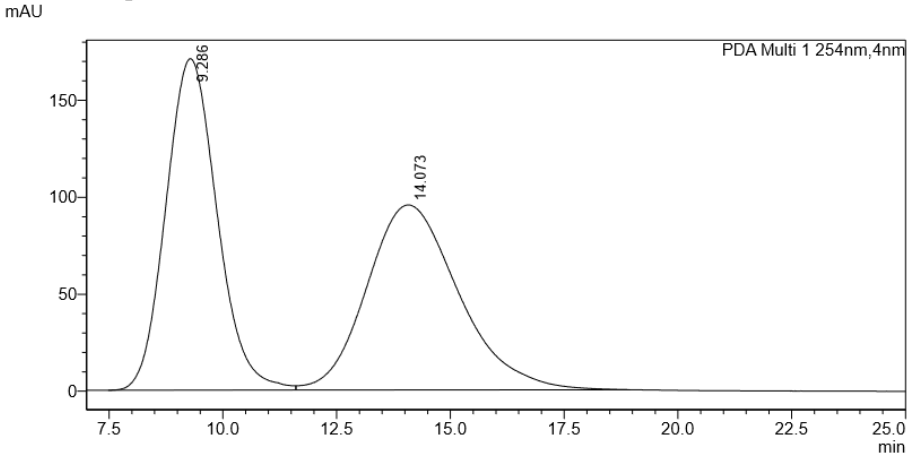

<Peak Table>

PDA Ch1 254nm

Peak#	Ret. Time	Area	Area%	Height
1	9.286	13389438	49.974	170854
2	14.073	13403205	50.026	95379
Total		26792642	100.000	266233

Supplementary Figure 203. HPLC Spectra of racemic **3s**

<Chromatogram>

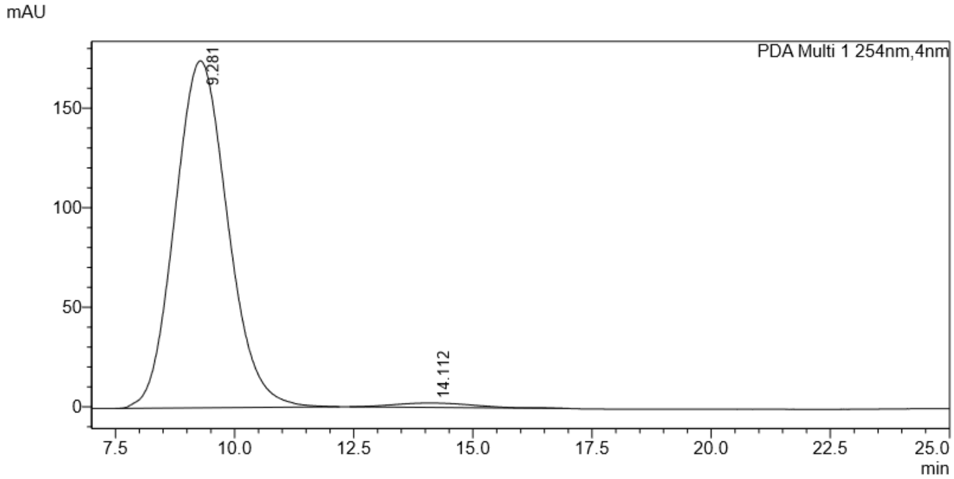

<Peak Table>

PDA Ch1 254nm

Peak#	Ret. Time	Area	Area%	Height
1	9.281	13470150	98.092	174240
2	14.112	262061	1.908	2223
Total		13732212	100.000	176463

Supplementary Figure 204. HPLC Spectra of **3s**

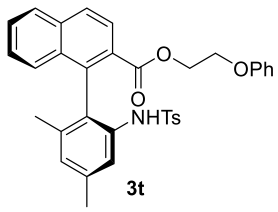

3t

<Chromatogram>

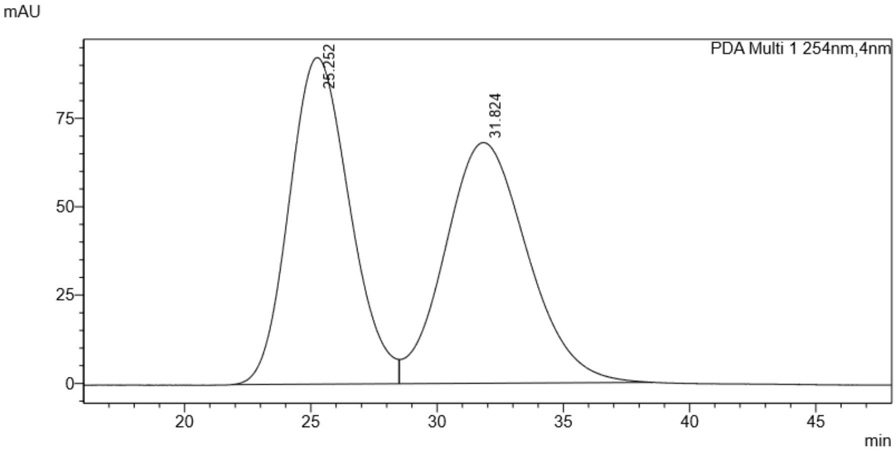

<Peak Table>

PDA Ch1 254nm

Peak#	Ret. Time	Area	Area%	Height
1	25.252	15385578	49.845	92431
2	31.824	15481545	50.155	68118
Total		30867122	100.000	160549

Supplementary Figure 205. HPLC Spectra of racemic **3t**

<Chromatogram>

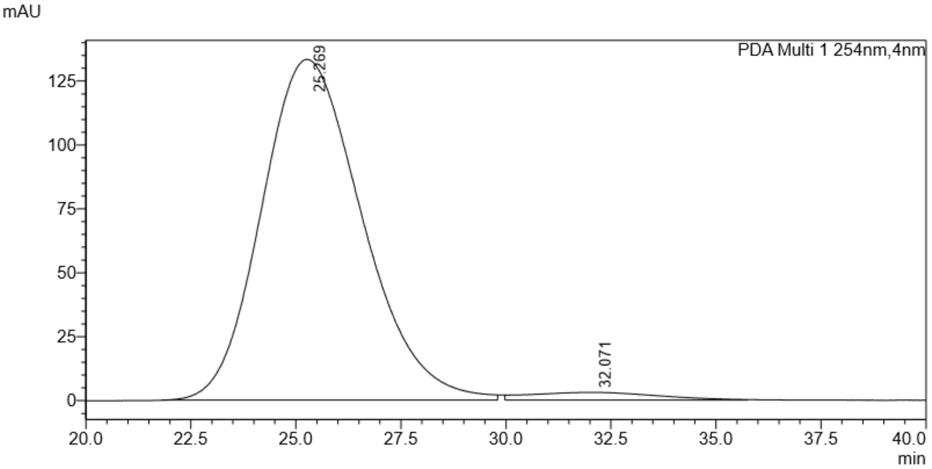

<Peak Table>

PDA Ch1 254nm

Peak#	Ret. Time	Area	Area%	Height
1	25.269	22205061	97.192	133243
2	32.071	641559	2.808	3007
Total		22846620	100.000	136250

Supplementary Figure 206. HPLC Spectra of **3t**

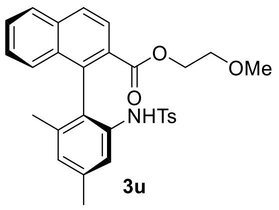

3u

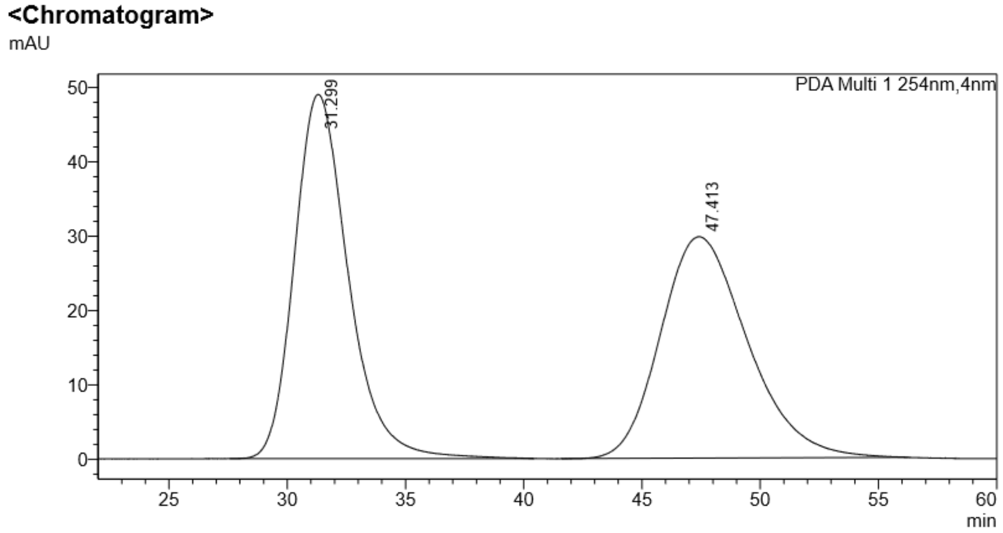

<Peak Table>

PDA Ch1 254nm

Peak#	Ret. Time	Area	Area%	Height
1	31.299	7772244	50.448	48967
2	47.413	7634107	49.552	29785
Total		15406351	100.000	78752

Supplementary Figure 207. HPLC Spectra of racemic **3u**

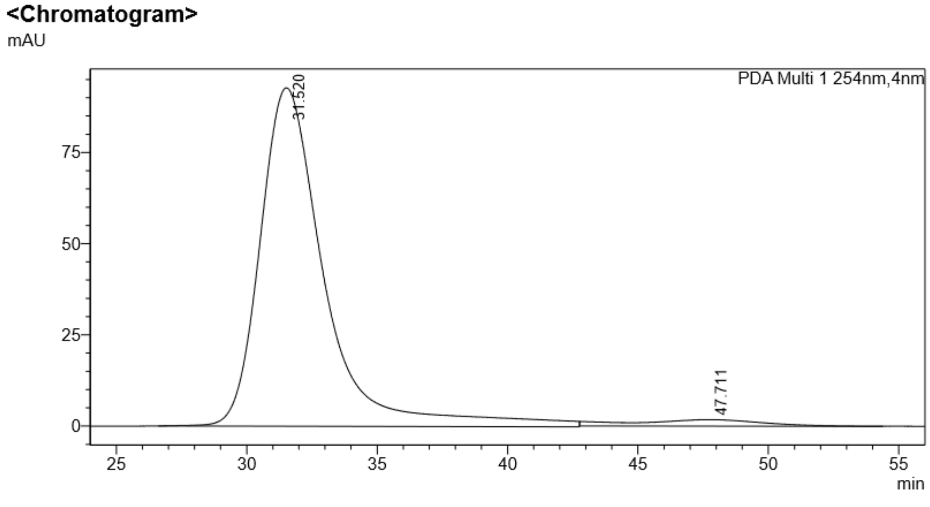

<Peak Table>

PDA Ch1 254nm

Peak#	Ret. Time	Area	Area%	Height
1	31.520	15936563	96.179	92776
2	47.711	633113	3.821	1794
Total		16569676	100.000	94570

Supplementary Figure 208. HPLC Spectra of **3u**

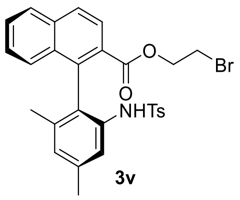

<Chromatogram>

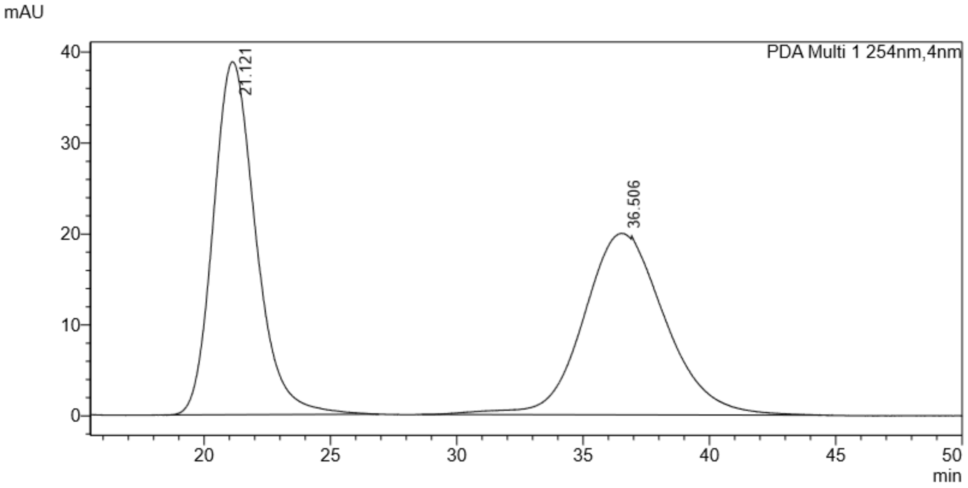

<Peak Table>

PDA Ch1 254nm

Peak#	Ret. Time	Area	Area%	Height
1	21.121	4544904	50.243	38818
2	36.506	4500852	49.757	19950
Total		9045756	100.000	58768

Supplementary Figure 209. HPLC Spectra of racemic **3v**

<Chromatogram>

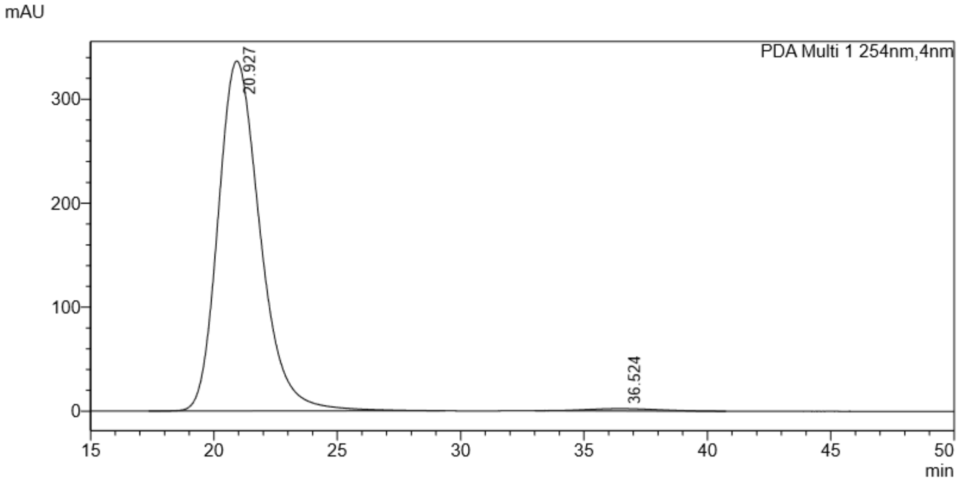

<Peak Table>

PDA Ch1 254nm

Peak#	Ret. Time	Area	Area%	Height
1	20.927	39803652	98.959	336596
2	36.524	418591	1.041	2155
Total		40222243	100.000	338751

Supplementary Figure 210. HPLC Spectra of **3v**

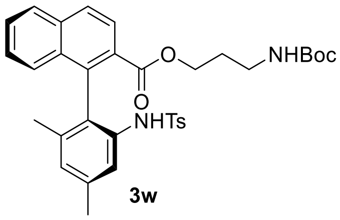

<Chromatogram>

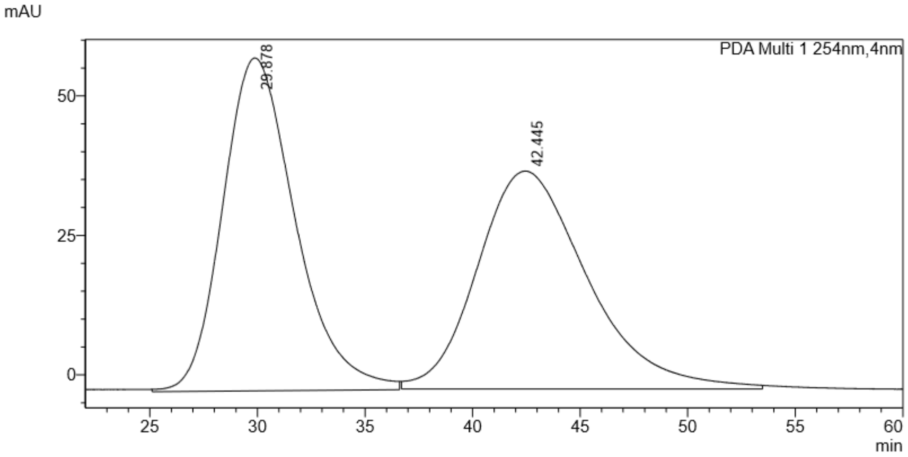

<Peak Table>

PDA Ch1 254nm

Peak#	Ret. Time	Area	Area%	Height
1	29.878	14002361	50.143	59645
2	42.445	13922507	49.857	39051
Total		27924867	100.000	98695

Supplementary Figure 211. HPLC Spectra of racemic **3w**

<Chromatogram>

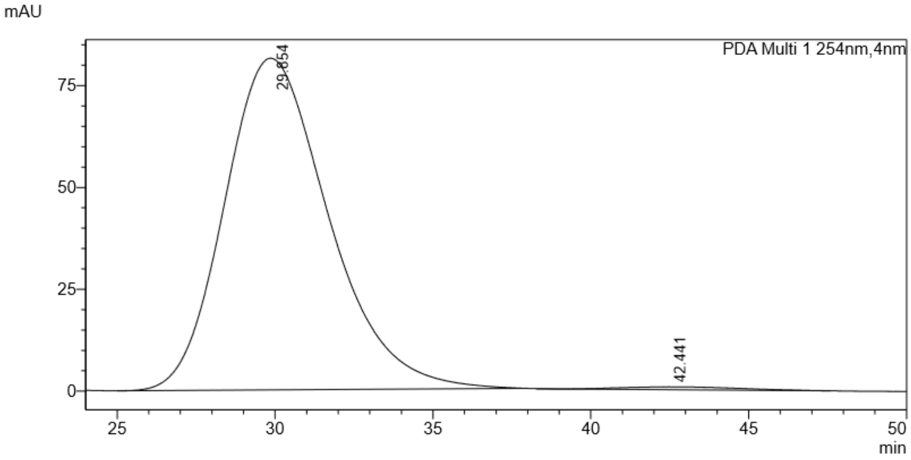

<Peak Table>

PDA Ch1 254nm

Peak#	Ret. Time	Area	Area%	Height
1	29.854	18697526	99.053	81438
2	42.441	178735	0.947	691
Total		18876261	100.000	82129

Supplementary Figure 212. HPLC Spectra of **3w**

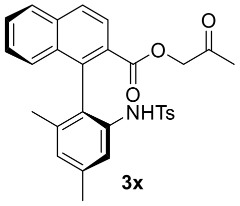

3x

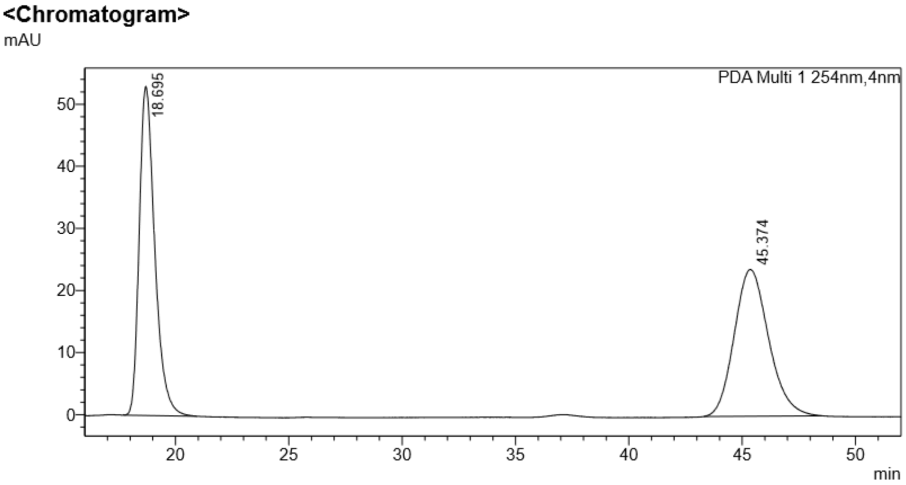

<Peak Table>

PDA Ch1 254nm

Peak#	Ret. Time	Area	Area%	Height
1	18.695	2523991	50.288	52966
2	45.374	2495051	49.712	23627
Total		5019043	100.000	76594

Supplementary Figure 213. HPLC Spectra of racemic **3x**

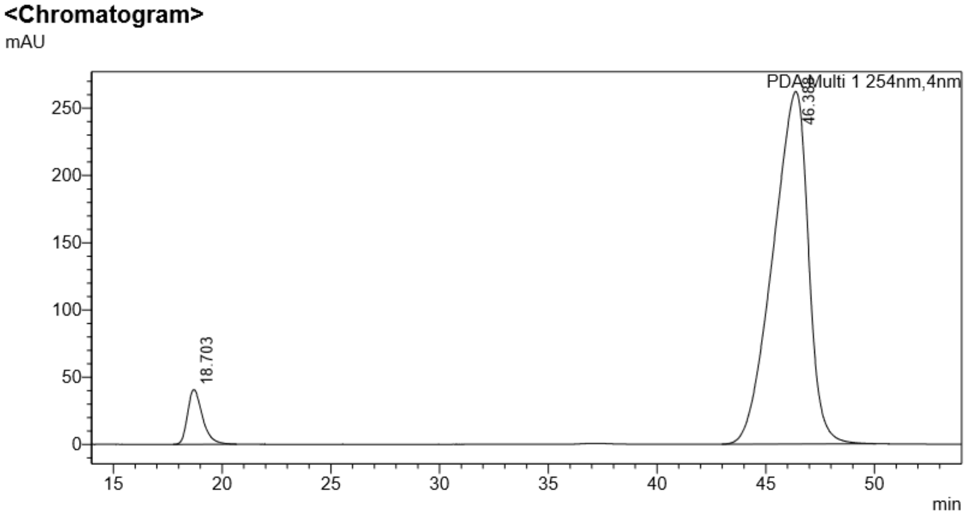

<Peak Table>

PDA Ch1 254nm

Peak#	Ret. Time	Area	Area%	Height
1	18.703	1933681	6.117	40629
2	46.388	29680071	93.883	262125
Total		31613752	100.000	302754

Supplementary Figure 214. HPLC Spectra of **3x**

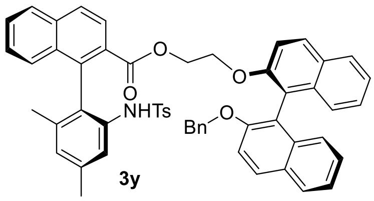

<Chromatogram>

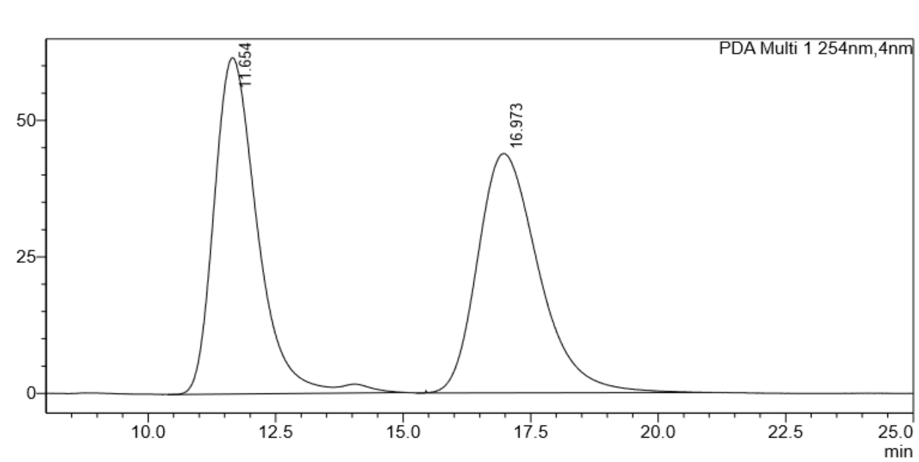

<Peak Table>

PDA Ch1 254nm

Peak#	Ret. Time	Area	Area%	Height
1	11.654	3653538	50.033	61578
2	16.973	3648740	49.967	43814
Total		7302278	100.000	105393

Supplementary Figure 215. HPLC Spectra of racemic **3y**

<Chromatogram>

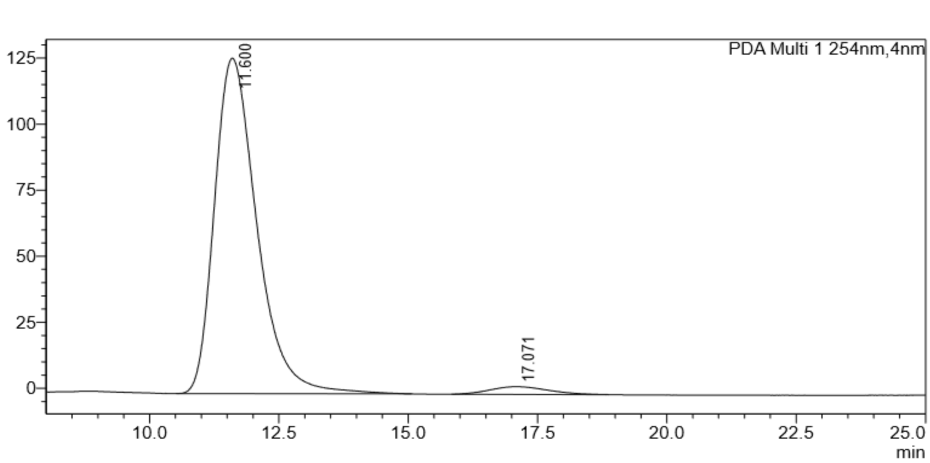

<Peak Table>

PDA Ch1 254nm

Peak#	Ret. Time	Area	Area%	Height
1	11.600	7257043	96.950	126966
2	17.071	228281	3.050	2926
Total		7485324	100.000	129892

Supplementary Figure 216. HPLC Spectra of **3y**

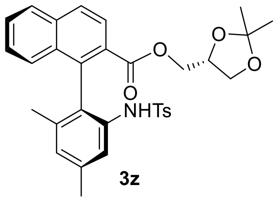

3z

<Chromatogram>

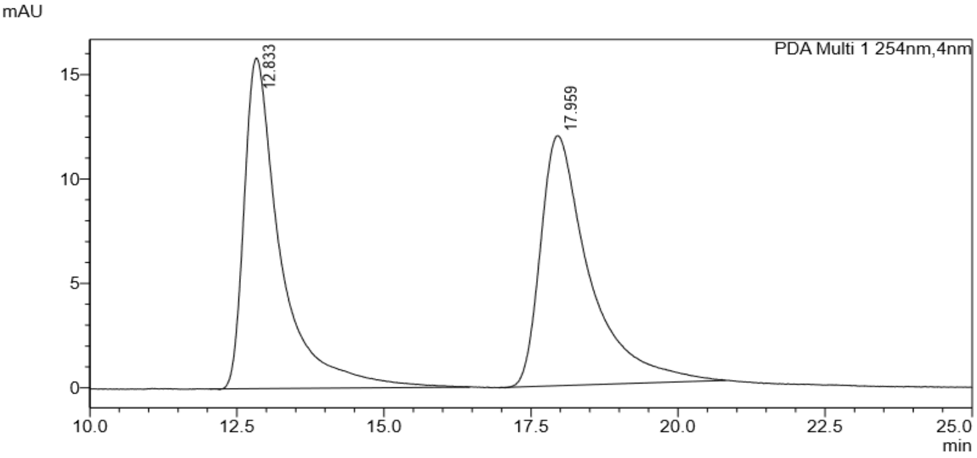

<Peak Table>

PDA Ch1 254nm

Peak#	Ret. Time	Area	Area%	Height
1	12.833	680947	49.524	15838
2	17.959	694034	50.476	11979
Total		1374980	100.000	27817

Supplementary Figure 217. HPLC Spectra of racemic **3z**

<Chromatogram>

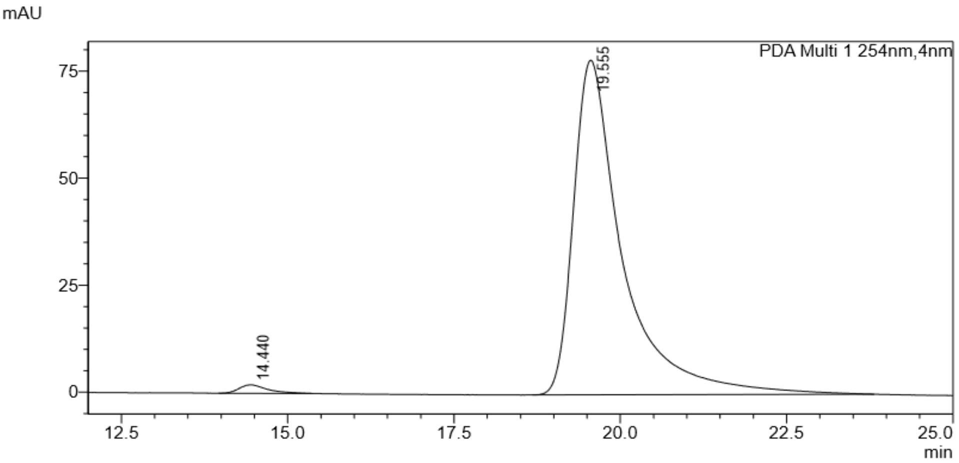

<Peak Table>

PDA Ch1 254nm

Peak#	Ret. Time	Area	Area%	Height
1	14.440	58994	1.442	1973
2	19.555	4031216	98.558	78155
Total		4090210	100.000	80128

Supplementary Figure 218. HPLC Spectra of **3z**

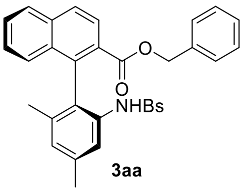

3aa

<Chromatogram>

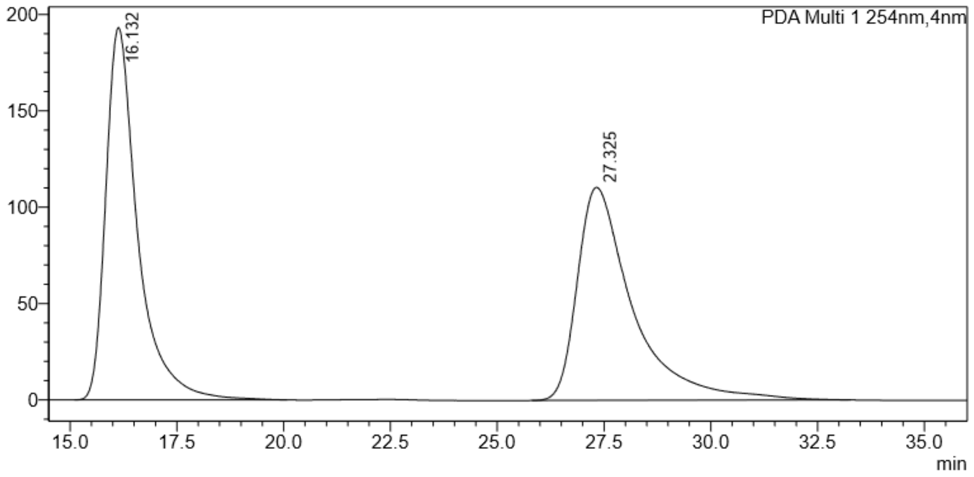

<Peak Table>

PDA Ch1 254nm

Peak#	Ret. Time	Area	Area%	Height
1	16.132	10036025	50.086	193104
2	27.325	10001671	49.914	110486
Total		20037695	100.000	303590

Supplementary Figure 219. HPLC Spectra of racemic **3aa**

<Chromatogram>

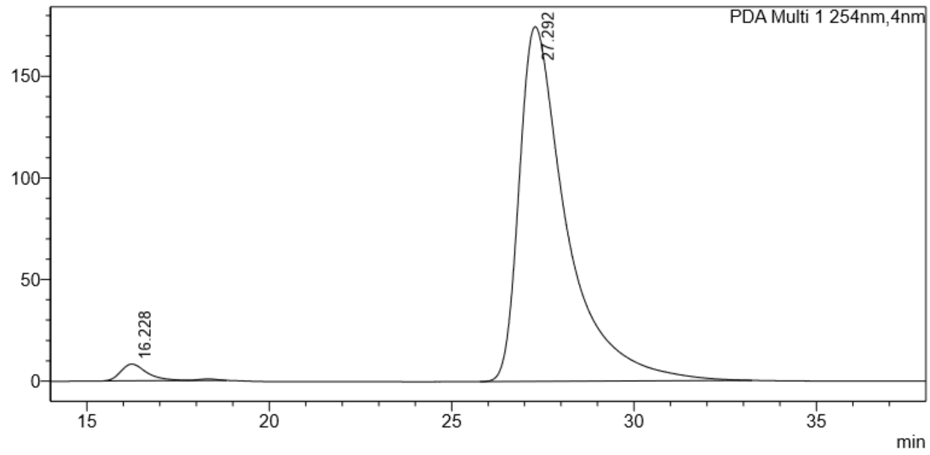

<Peak Table>

PDA Ch1 254nm

Peak#	Ret. Time	Area	Area%	Height
1	16.228	435079	2.681	8214
2	27.292	15794328	97.319	174628
Total		16229407	100.000	182842

Supplementary Figure 220. HPLC Spectra of **3aa**

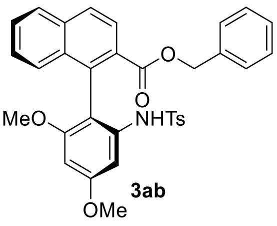

3ab

<Chromatogram>

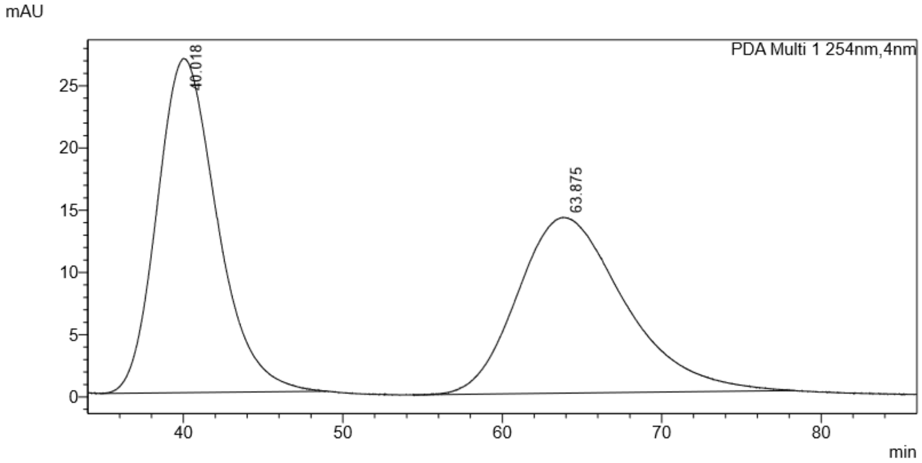

<Peak Table>

PDA Ch1 254nm

Peak#	Ret. Time	Area	Area%	Height
1	40.018	7019917	51.453	26840
2	63.875	6623559	48.547	14115
Total		13643476	100.000	40955

Supplementary Figure 221. HPLC Spectra of racemic **3ab**

<Chromatogram>

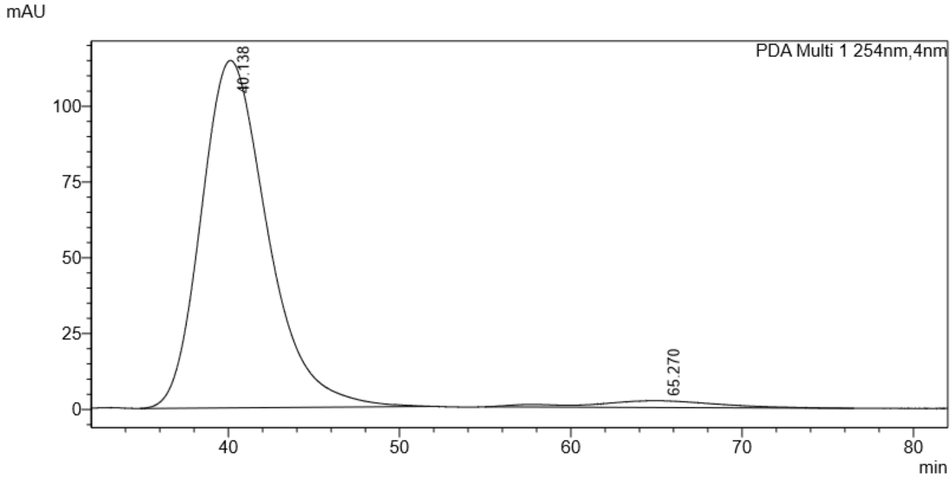

<Peak Table>

PDA Ch1 254nm

Peak#	Ret. Time	Area	Area%	Height
1	40.138	30905776	96.342	114585
2	65.270	1173361	3.658	2281
Total		32079137	100.000	116867

Supplementary Figure 222. HPLC Spectra of **3ab**

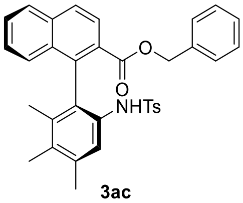

3ac

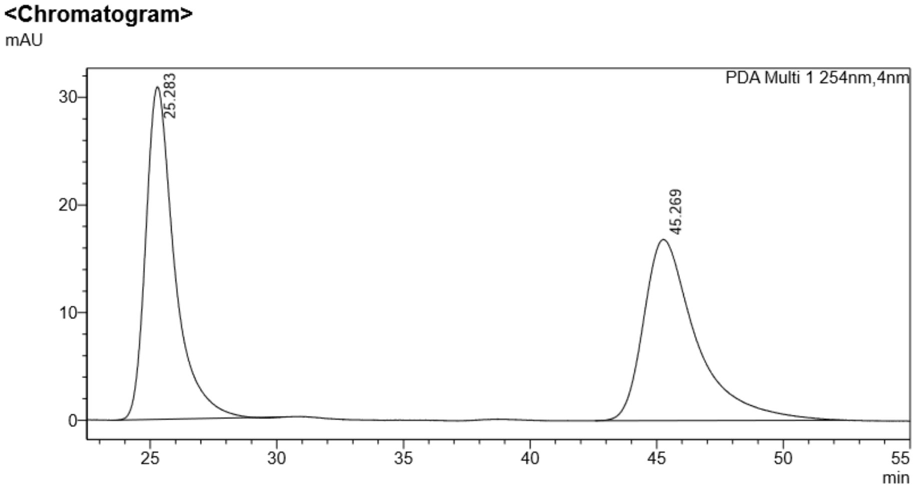

<Peak Table>

PDA Ch1 254nm

Peak#	Ret. Time	Area	Area%	Height
1	25.283	2439647	50.016	30892
2	45.269	2438123	49.984	16828
Total		4877770	100.000	47720

Supplementary Figure 223. HPLC Spectra of racemic **3ac**

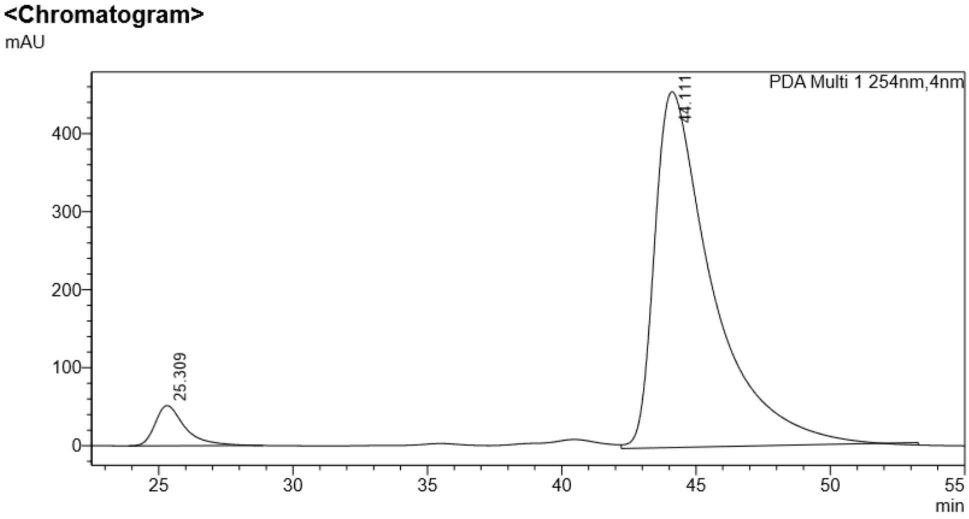

<Peak Table>

PDA Ch1 254nm

Peak#	Ret. Time	Area	Area%	Height
1	25.309	3981058	5.533	51409
2	44.111	67965504	94.467	455687
Total		71946562	100.000	507096

Supplementary Figure 224. HPLC Spectra of **3ac**

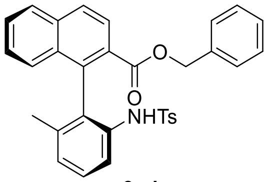

3ad

<Chromatogram>

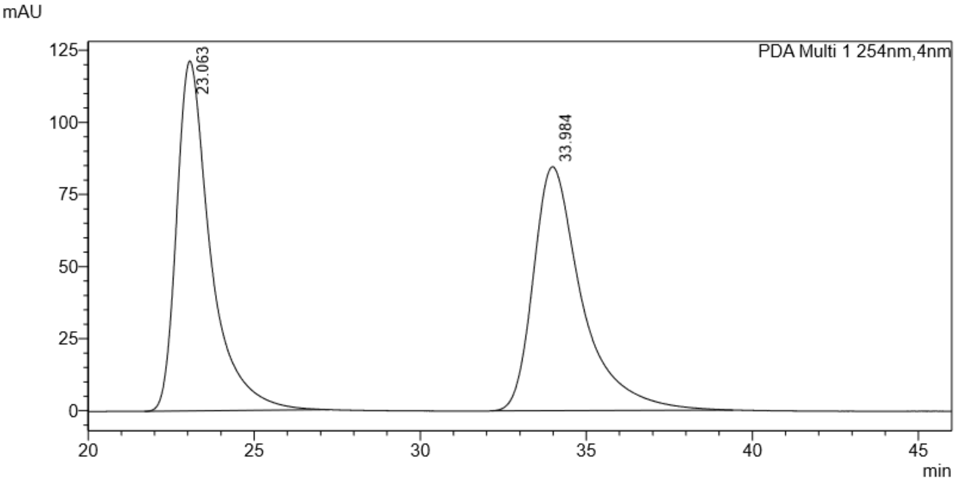

<Peak Table>

PDA Ch1 254nm

Peak#	Ret. Time	Area	Area%	Height
1	23.063	8610483	49.963	121385
2	33.984	8623384	50.037	84610
Total		17233867	100.000	205994

Supplementary Figure 225. HPLC Spectra of racemic **3ad**

<Chromatogram>

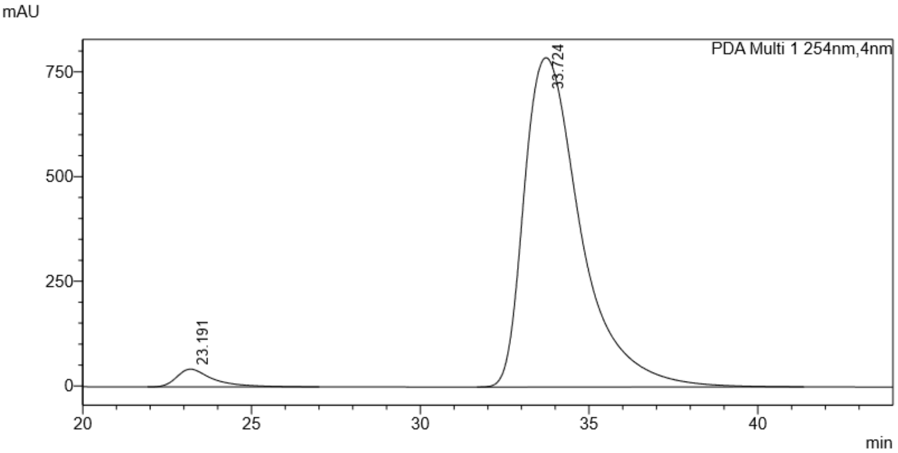

<Peak Table>

PDA Ch1 254nm

Peak#	Ret. Time	Area	Area%	Height
1	23.191	3174465	3.341	42400
2	33.724	91847585	96.659	786030
Total		95022051	100.000	828430

Supplementary Figure 226. HPLC Spectra of **3ad**

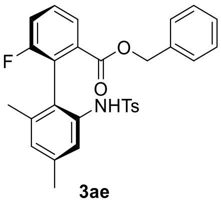

3ae

<Chromatogram>

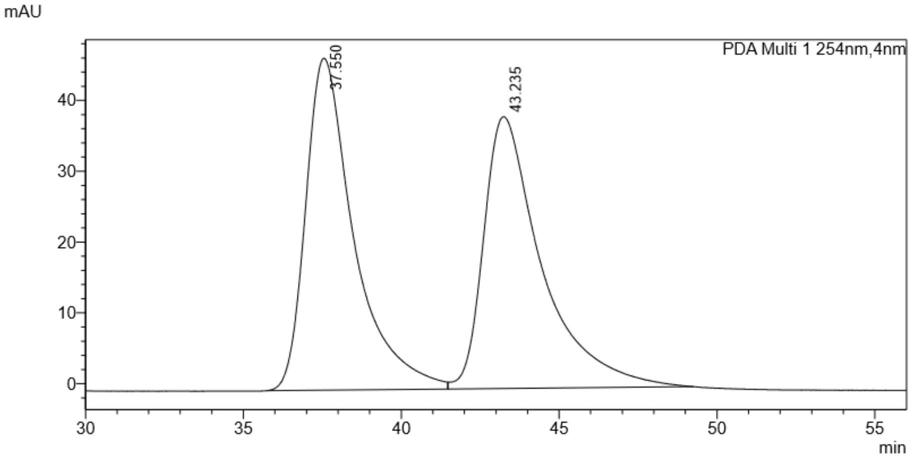

<Peak Table>

PDA Ch1 254nm

Peak#	Ret. Time	Area	Area%	Height
1	37.550	4972395	50.324	46857
2	43.235	4908436	49.676	38372
Total		9880831	100.000	85228

Supplementary Figure 227. HPLC Spectra of racemic **3ae**

<Chromatogram>

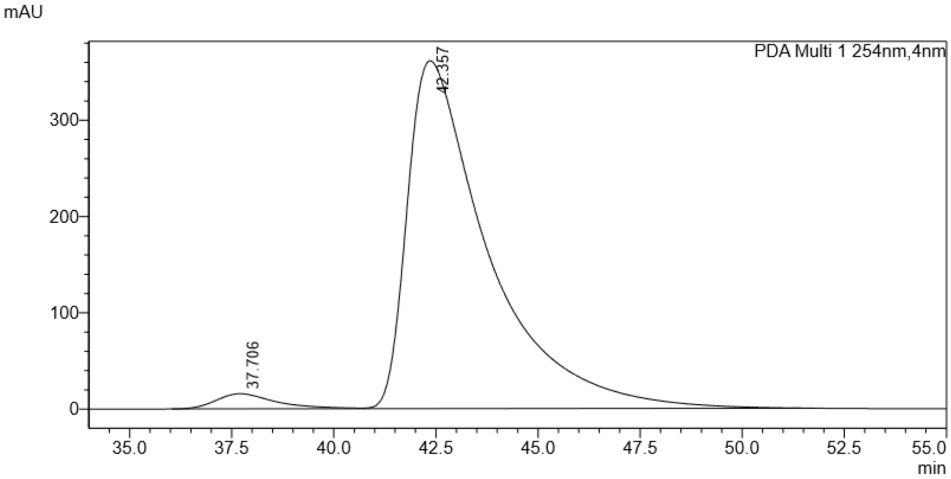

<Peak Table>

PDA Ch1 254nm

Peak#	Ret. Time	Area	Area%	Height
1	37.706	1612297	3.126	15724
2	42.357	49965390	96.874	361224
Total		51577687	100.000	376949

Supplementary Figure 228. HPLC Spectra of **3ae**

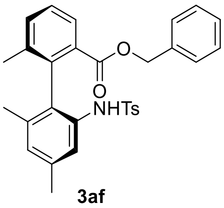

3af

<Chromatogram>

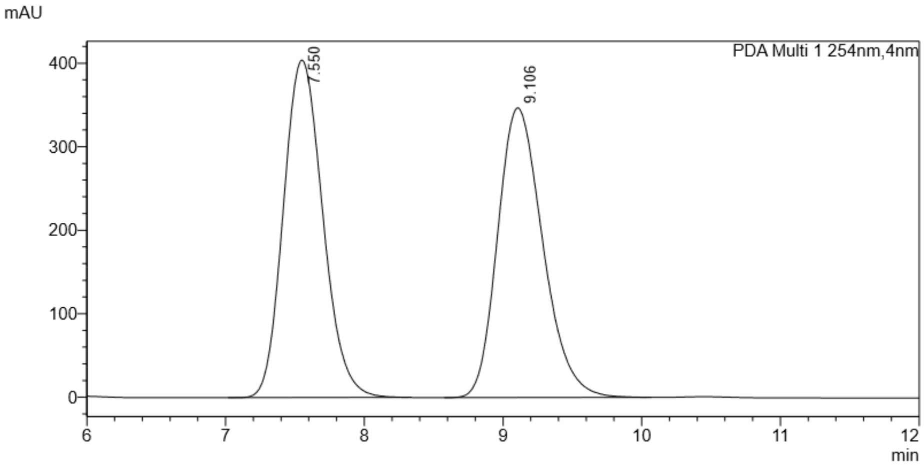

<Peak Table>

PDA Ch1 254nm

Peak#	Ret. Time	Area	Area%	Height
1	7.550	7987620	50.386	404121
2	9.106	7865364	49.614	346803
Total		15852984	100.000	750924

Supplementary Figure 229. HPLC Spectra of racemic **3af**

<Chromatogram>

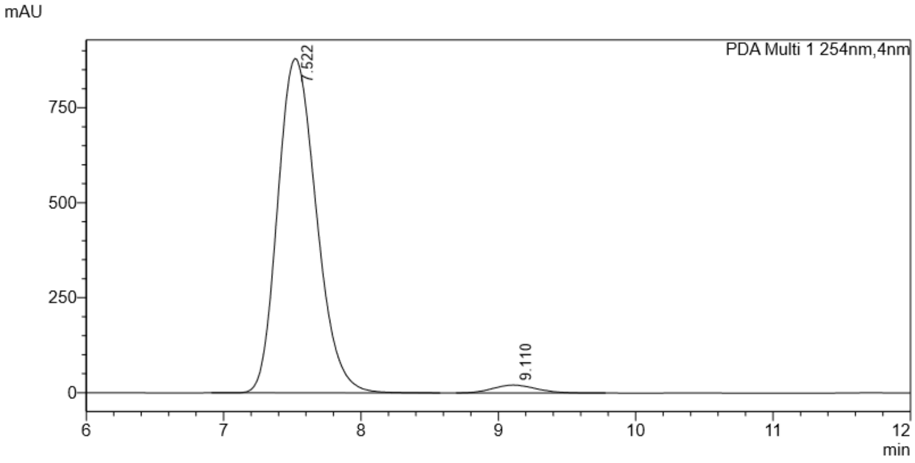

<Peak Table>

PDA Ch1 254nm

Peak#	Ret. Time	Area	Area%	Height
1	7.522	17214629	97.433	879861
2	9.110	453544	2.567	20677
Total		17668174	100.000	900539

Supplementary Figure 230. HPLC Spectra of **3af**

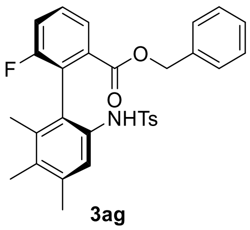

3ag

<Chromatogram>

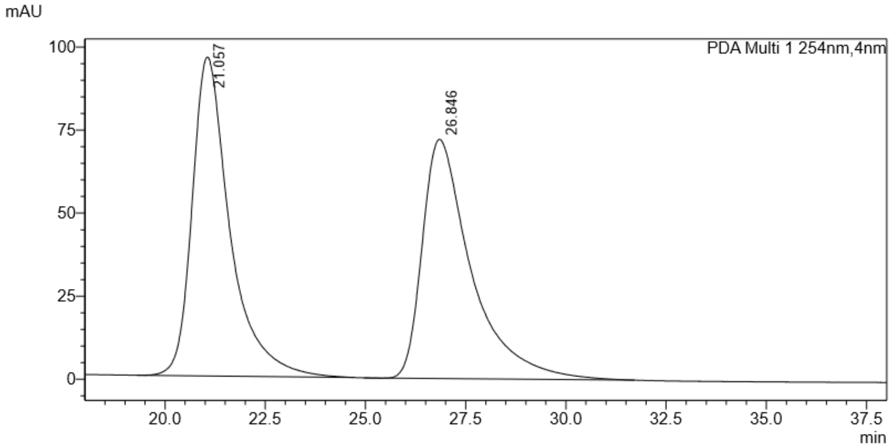

<Peak Table>

PDA Ch1 254nm

Peak#	Ret. Time	Area	Area%	Height
1	21.057	6306001	50.750	96023
2	26.846	6119684	49.250	72004
Total		12425686	100.000	168027

Supplementary Figure 231. HPLC Spectra of racemic **3ag**

<Chromatogram>

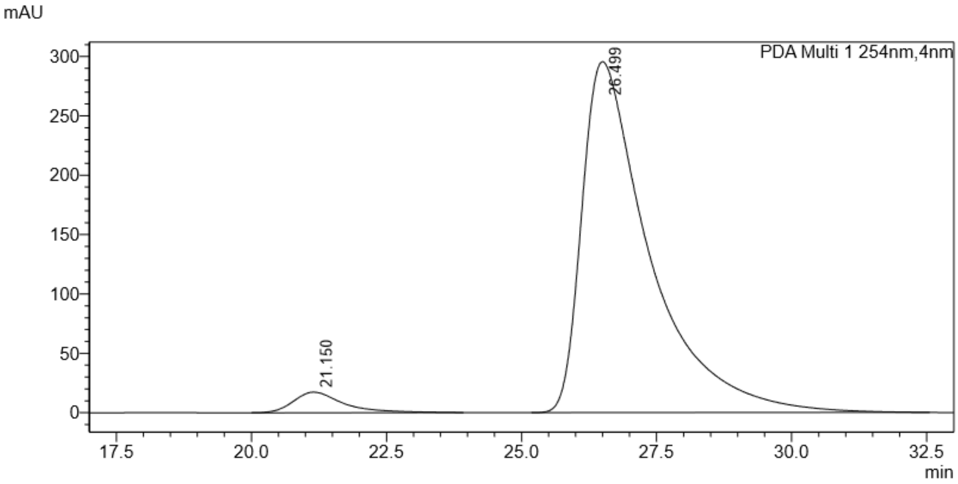

<Peak Table>

PDA Ch1 254nm

Peak#	Ret. Time	Area	Area%	Height
1	21.150	1112511	4.122	17308
2	26.499	25873858	95.878	295532
Total		26986368	100.000	312841

Supplementary Figure 232. HPLC Spectra of **3ag**

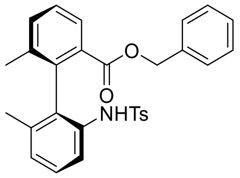

3ah

<Chromatogram>

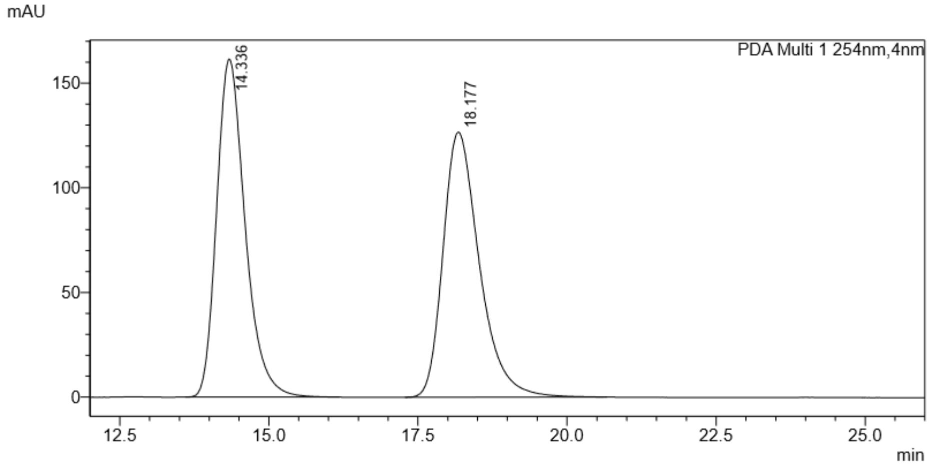

<Peak Table>

PDA Ch1 254nm

Peak#	Ret. Time	Area	Area%	Height
1	14.336	5366616	49.999	161507
2	18.177	5366759	50.001	126636
Total		10733375	100.000	288144

Supplementary Figure 233. HPLC Spectra of racemic **3ah**

<Chromatogram>

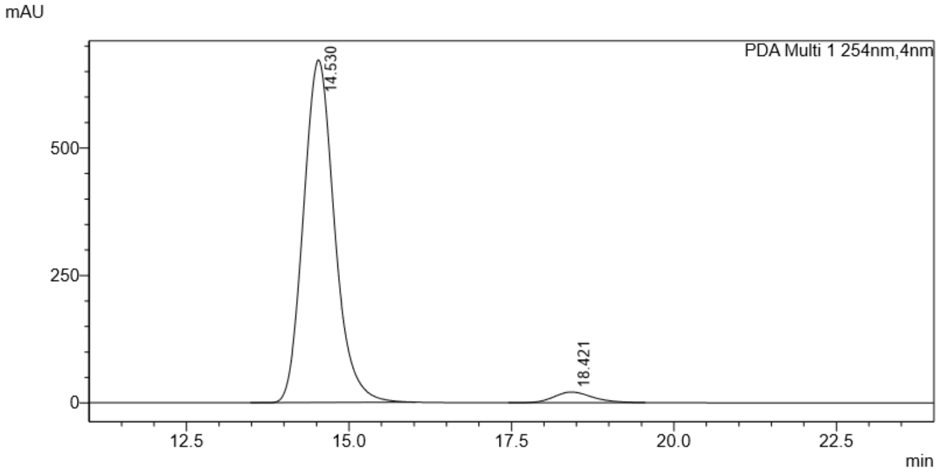

<Peak Table>

PDA Ch1 254nm

Peak#	Ret. Time	Area	Area%	Height
1	14.530	22354880	96.337	671916
2	18.421	849969	3.663	20719
Total		23204849	100.000	692635

Supplementary Figure 234. HPLC Spectra of **3ah**

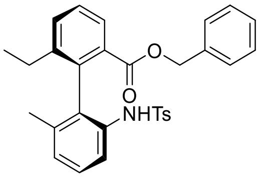

3ai

<Chromatogram>

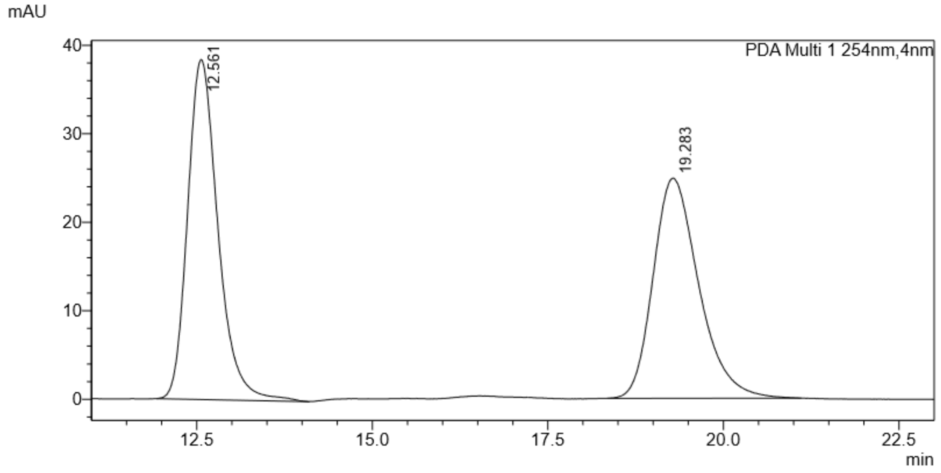

<Peak Table>

PDA Ch1 254nm

Peak#	Ret. Time	Area	Area%	Height
1	12.561	1156334	50.678	38408
2	19.283	1125410	49.322	24863
Total		2281744	100.000	63271

Supplementary Figure 235. HPLC Spectra of racemic **3ai**

<Chromatogram>

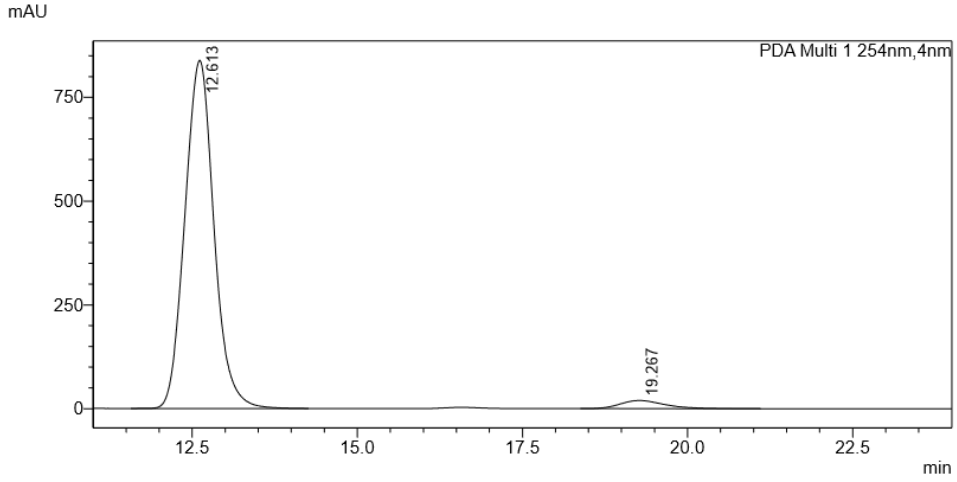

<Peak Table>

PDA Ch1 254nm

Peak#	Ret. Time	Area	Area%	Height
1	12.613	25721851	96.541	838498
2	19.267	921507	3.459	19564
Total		26643358	100.000	858061

Supplementary Figure 236. HPLC Spectra of **3ai**

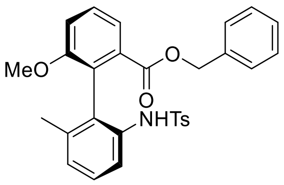

3aj

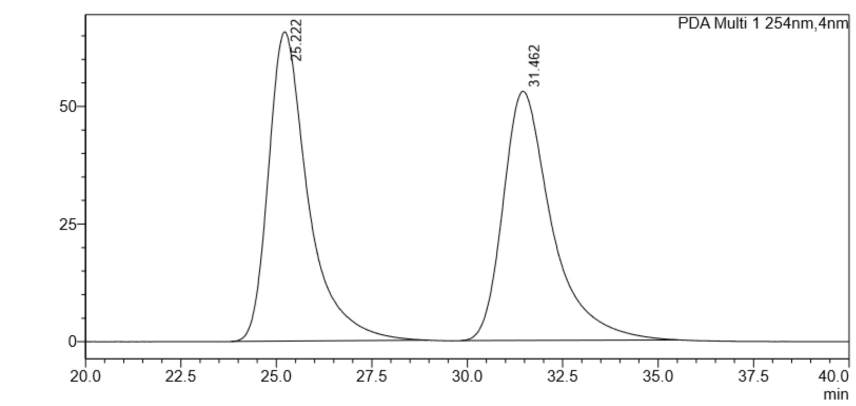

<Peak Table>

PDA Ch1 254nm

Peak#	Ret. Time	Area	Area%	Height
1	25.222	4765831	50.362	65734
2	31.462	4697311	49.638	52958
Total		9463142	100.000	118692

Supplementary Figure 237. HPLC Spectra of racemic **3aj**

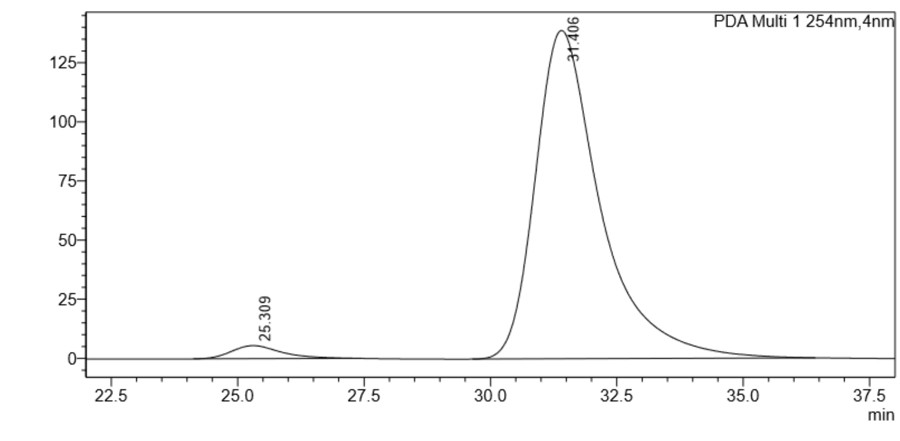

<Peak Table>

PDA Ch1 254nm

Peak#	Ret. Time	Area	Area%	Height
1	25.309	385716	2.998	5546
2	31.406	12481284	97.002	138847
Total		12867001	100.000	144393

Supplementary Figure 238. HPLC Spectra of **3aj**

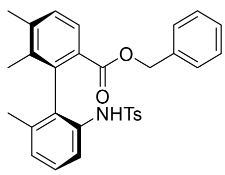

3ak

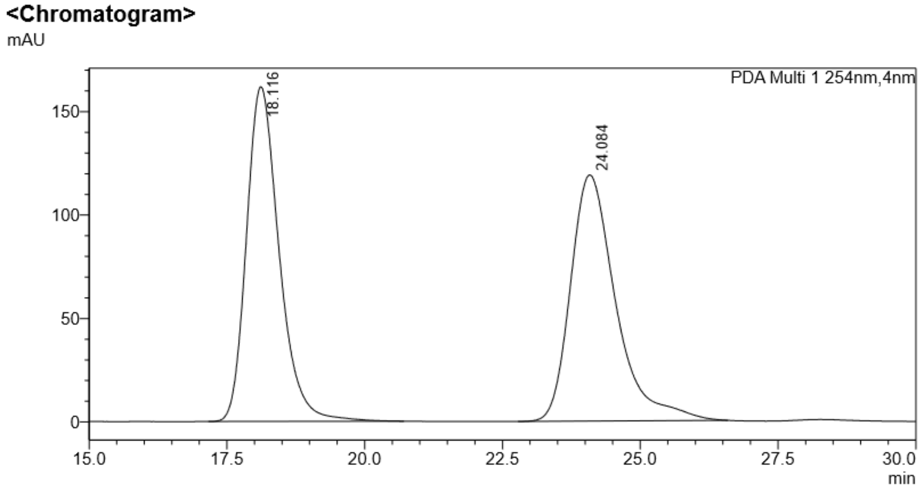

<Peak Table>

PDA Ch1 254nm

Peak#	Ret. Time	Area	Area%	Height
1	18.116	6675727	49.567	161740
2	24.084	6792474	50.433	119005
Total		13468202	100.000	280745

Supplementary Figure 239. HPLC Spectra of racemic **3ak**

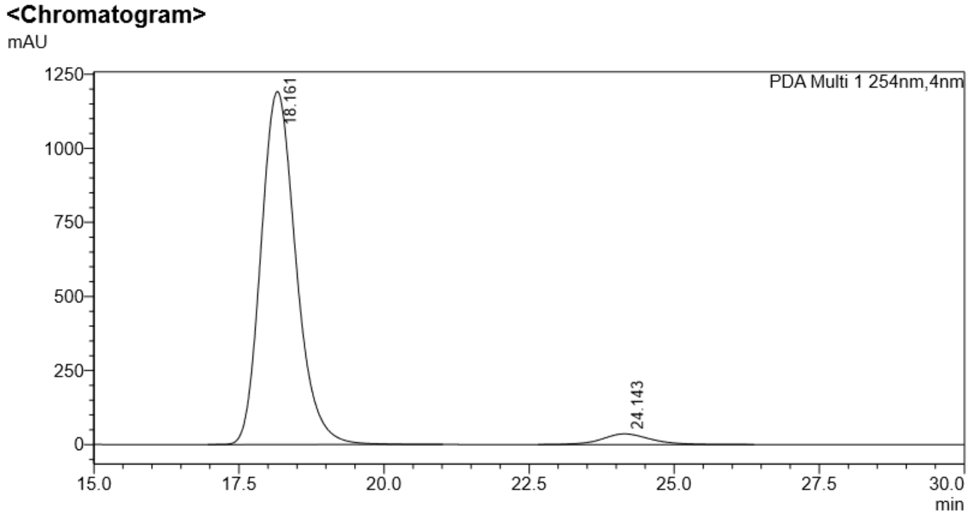

<Peak Table>

PDA Ch1 254nm

Peak#	Ret. Time	Area	Area%	Height
1	18.161	49894363	95.973	1191366
2	24.143	2093676	4.027	36046
Total		51988039	100.000	1227411

Supplementary Figure 240. HPLC Spectra of **3ak**

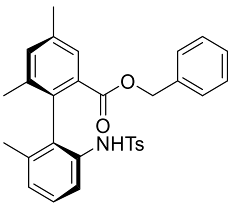

3al

<Chromatogram>

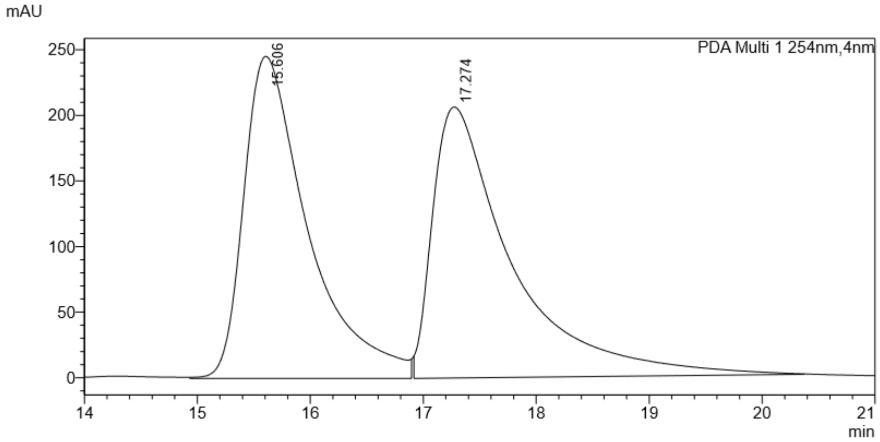

<Peak Table>

PDA Ch1 254nm

Peak#	Ret. Time	Area	Area%	Height
1	15.606	9709871	49.053	245587
2	17.274	10084887	50.947	206576
Total		19794758	100.000	452163

Supplementary Figure 241. HPLC Spectra of racemic **3al**

<Chromatogram>

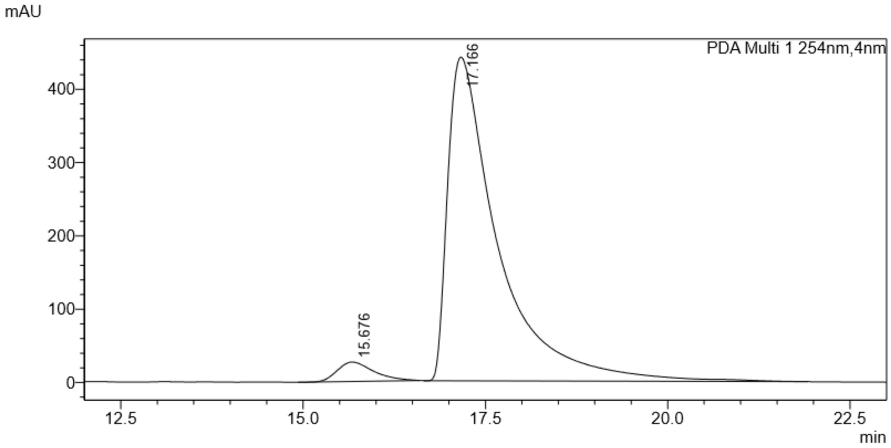

<Peak Table>

PDA Ch1 254nm

Peak#	Ret. Time	Area	Area%	Height
1	15.676	914784	4.123	26358
2	17.166	21271340	95.877	441493
Total		22186124	100.000	467851

Supplementary Figure 242. HPLC Spectra of **3al**

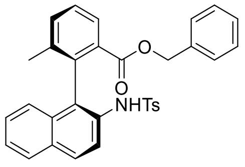

3am

<Chromatogram>

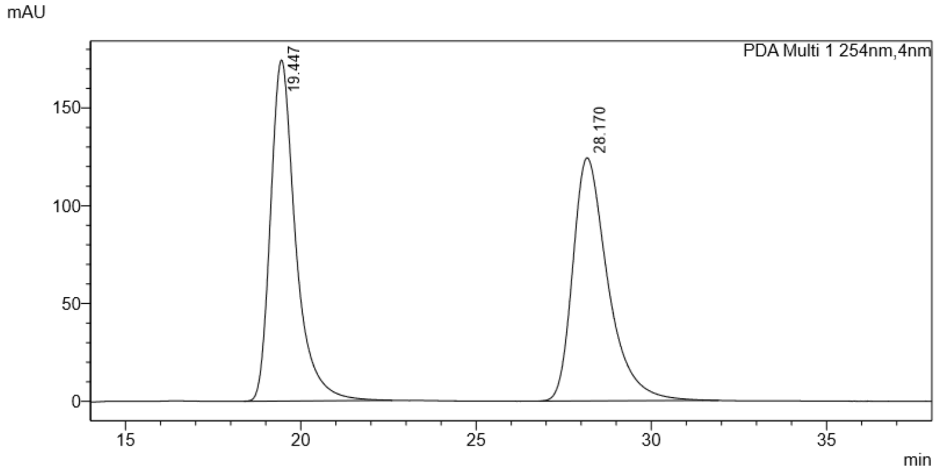

<Peak Table>

PDA Ch1 254nm

Peak#	Ret. Time	Area	Area%	Height
1	19.447	8683767	50.069	174296
2	28.170	8659751	49.931	124233
Total		17343518	100.000	298529

Supplementary Figure 243. HPLC Spectra of racemic **3am**

<Chromatogram>

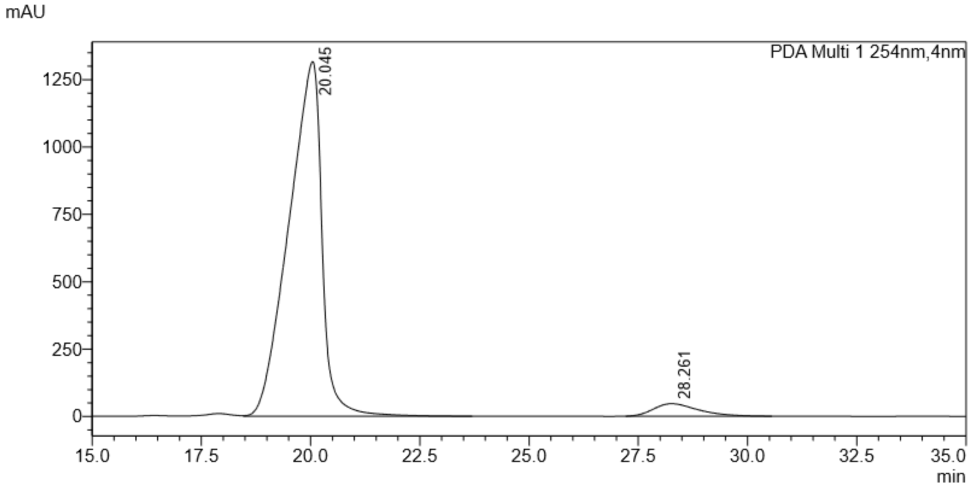

<Peak Table>

PDA Ch1 254nm

Peak#	Ret. Time	Area	Area%	Height
1	20.045	69305959	95.461	1315542
2	28.261	3295146	4.539	46387
Total		72601104	100.000	1361929

Supplementary Figure 244. HPLC Spectra of **3am**

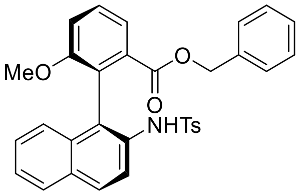

3an

<Chromatogram>

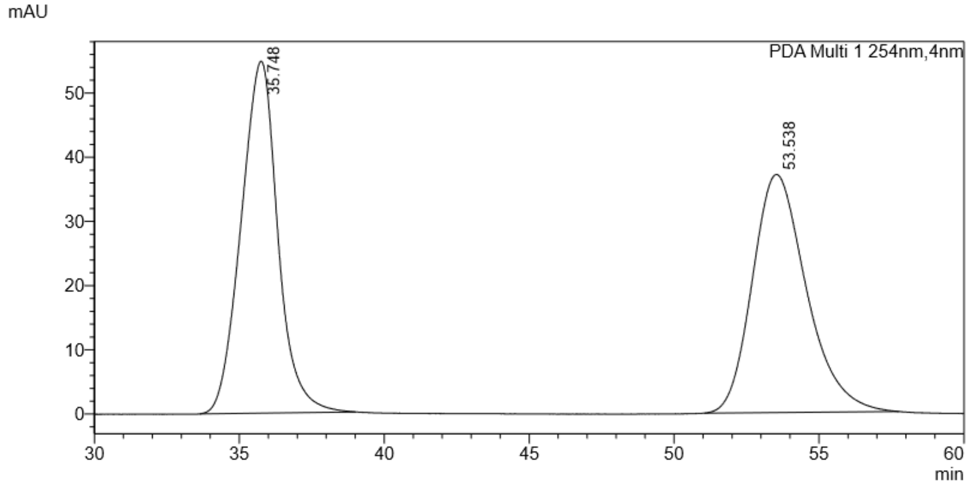

<Peak Table>

PDA Ch1 254nm

Peak#	Ret. Time	Area	Area%	Height
1	35.748	4839942	50.108	54827
2	53.538	4819044	49.892	37091
Total		9658986	100.000	91917

Supplementary Figure 245. HPLC Spectra of racemic **3an**

<Chromatogram>

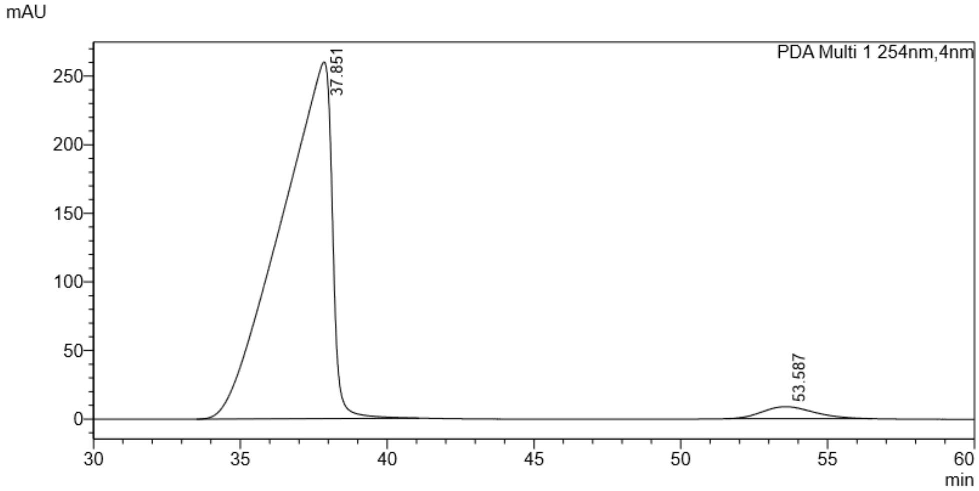

<Peak Table>

PDA Ch1 254nm

Peak#	Ret. Time	Area	Area%	Height
1	37.851	31658595	96.694	259943
2	53.587	1082468	3.306	8811
Total		32741063	100.000	268754

Supplementary Figure 246. HPLC Spectra of **3an**

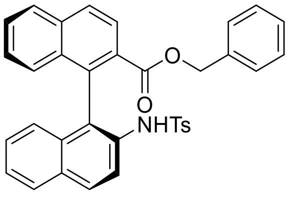

3ao

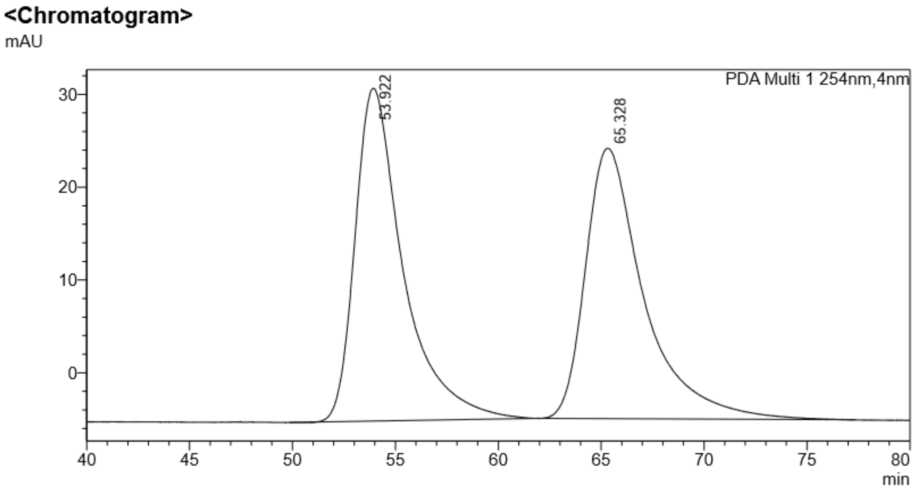

<Peak Table>

PDA Ch1 254nm

Peak#	Ret. Time	Area	Area%	Height
1	53.922	5822410	50.289	35854
2	65.328	5755589	49.711	29142
Total		11577999	100.000	64996

Supplementary Figure 247. HPLC Spectra of racemic **3ao**

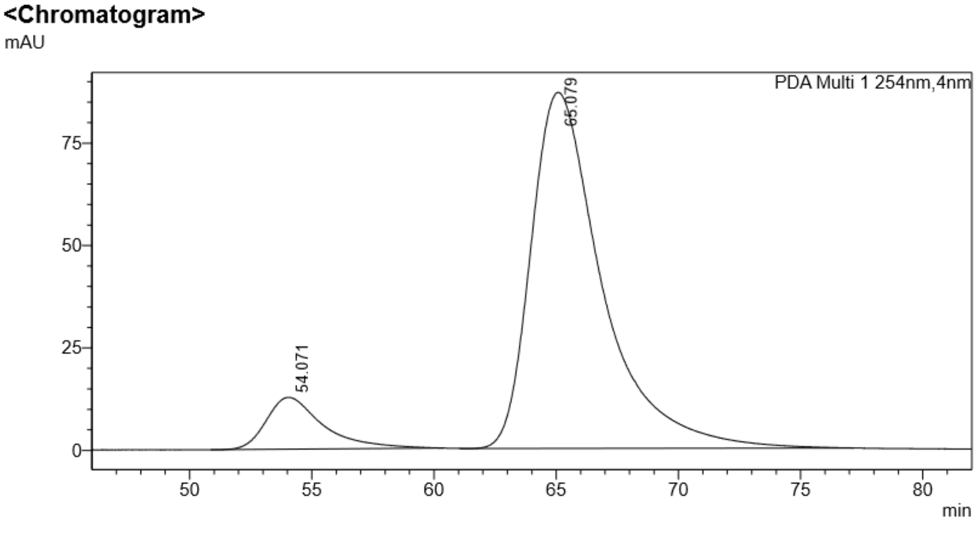

<Peak Table>

PDA Ch1 254nm

Peak#	Ret. Time	Area	Area%	Height
1	54.071	2046172	10.413	12650
2	65.079	17604126	89.587	86858
Total		19650298	100.000	99508

Supplementary Figure 248. HPLC Spectra of **3ao**

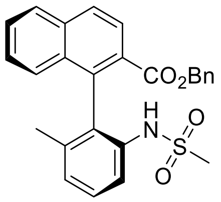

3ap

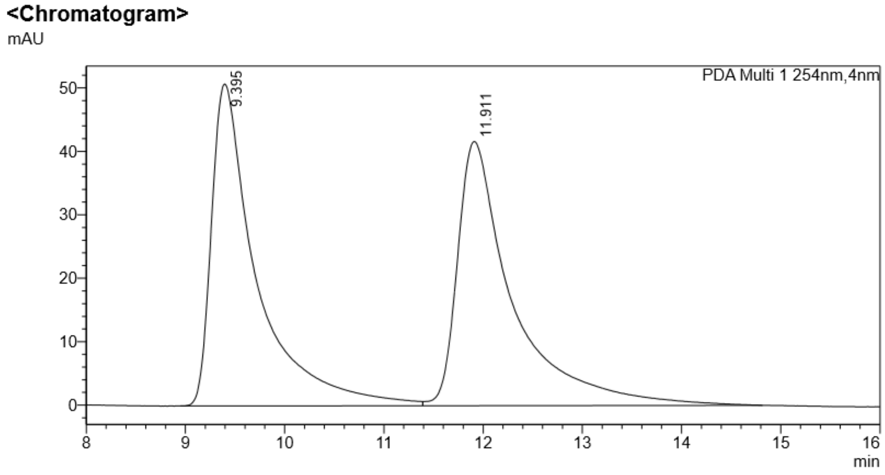

<Peak Table>

PDA Ch1 254nm

Peak#	Ret. Time	Area	Area%	Height
1	9.395	1606487	49.961	50737
2	11.911	1609014	50.039	41624
Total		3215501	100.000	92361

Supplementary Figure 249. HPLC Spectra of racemic **3ap**

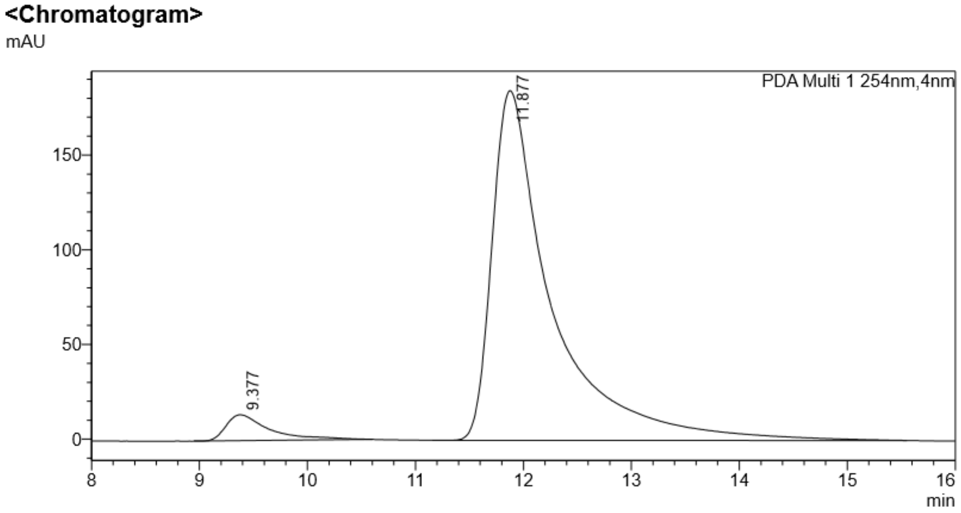

<Peak Table>

PDA Ch1 254nm

Peak#	Ret. Time	Area	Area%	Height
1	9.377	387536	5.153	13718
2	11.877	7132440	94.847	184606
Total		7519976	100.000	198324

Supplementary Figure 250. HPLC Spectra of **3ap**

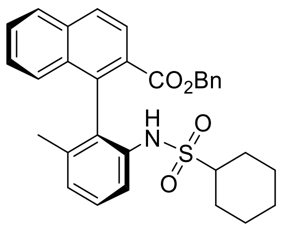

3aq

<Chromatogram>

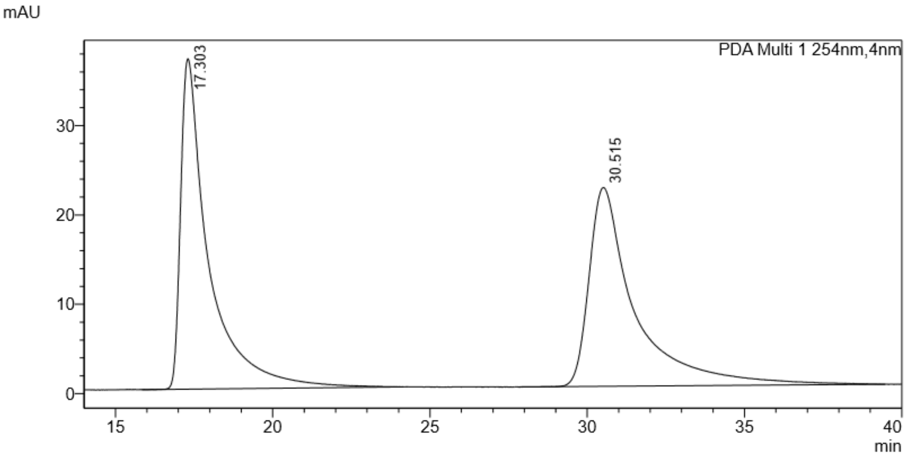

<Peak Table>

PDA Ch1 254nm

Peak#	Ret. Time	Area	Area%	Height
1	17.303	2291824	50.226	36959
2	30.515	2271227	49.774	22248
Total		4563052	100.000	59207

Supplementary Figure 251. HPLC Spectra of racemic **3aq**

<Chromatogram>

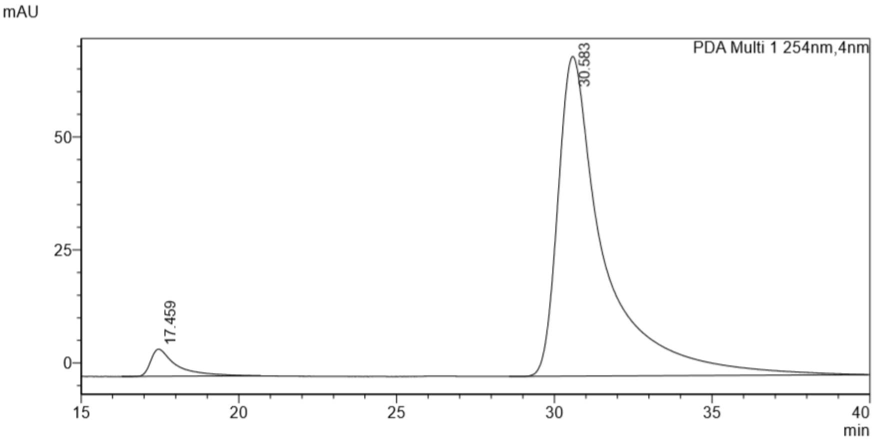

<Peak Table>

PDA Ch1 254nm

Peak#	Ret. Time	Area	Area%	Height
1	17.459	342861	4.526	6003
2	30.583	7233065	95.474	70699
Total		7575926	100.000	76702

Supplementary Figure 252. HPLC Spectra of **3aq**

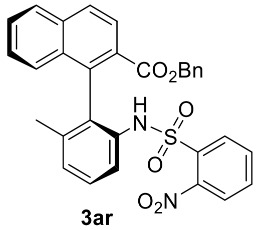

3ar

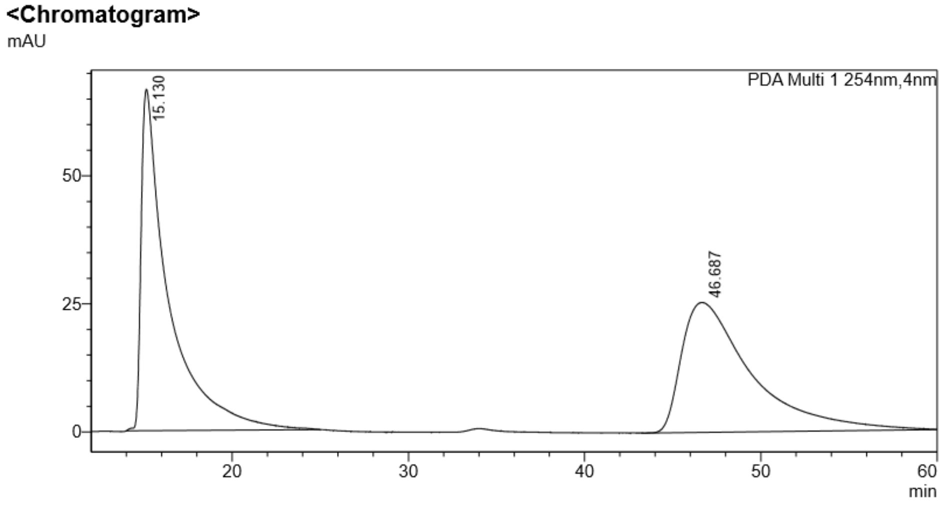

<Peak Table>

PDA Ch1 254nm

Peak#	Ret. Time	Area	Area%	Height
1	15.130	7169147	50.551	66649
2	46.687	7012793	49.449	25348
Total		14181940	100.000	91997

Supplementary Figure 253. HPLC Spectra of racemic **3ar**

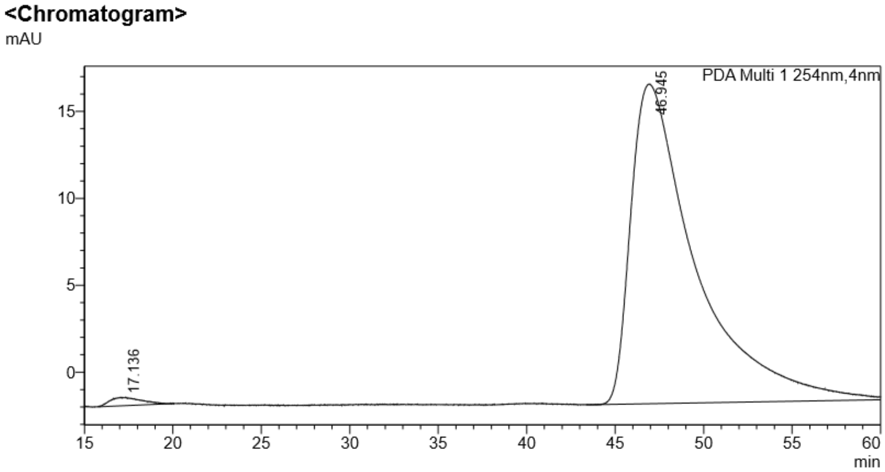

<Peak Table>

PDA Ch1 254nm

Peak#	Ret. Time	Area	Area%	Height
1	17.136	58028	1.222	472
2	46.945	4690034	98.778	18378
Total		4748062	100.000	18850

Supplementary Figure 254. HPLC Spectra of **3ar**

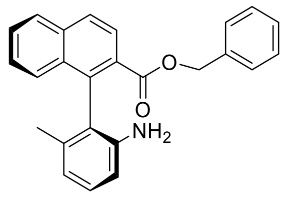

6

<Chromatogram>

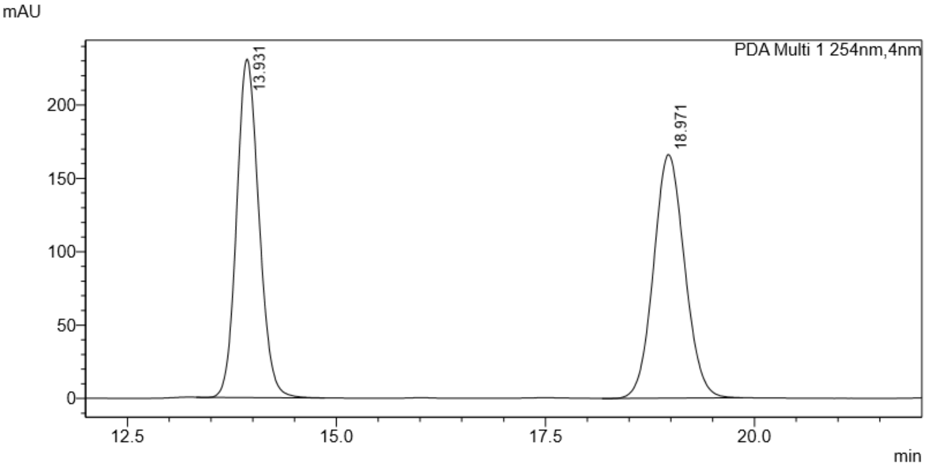

<Peak Table>

PDA Ch1 254nm

Peak#	Ret. Time	Area	Area%	Height
1	13.931	4282667	49.915	230584
2	18.971	4297259	50.085	165913
Total		8579926	100.000	396497

Supplementary Figure 255. HPLC Spectra of racemic **6**

<Chromatogram>

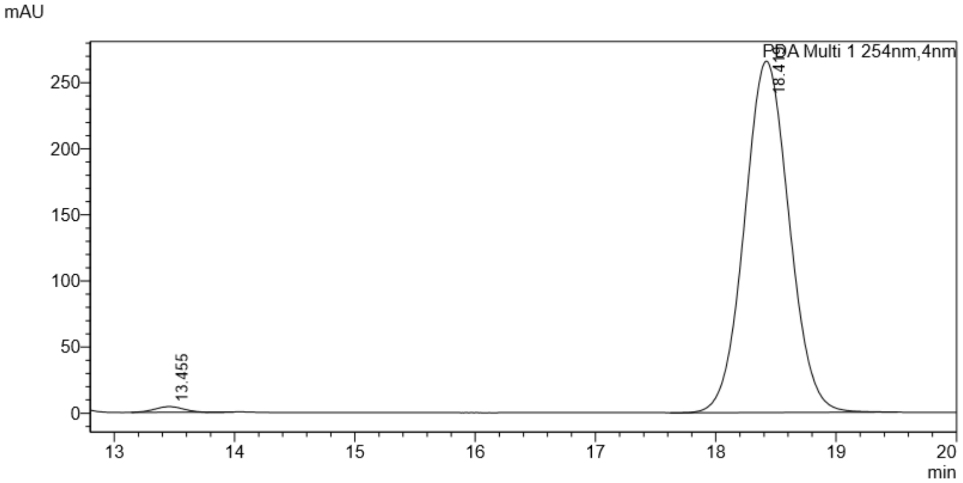

<Peak Table>

PDA Ch1 254nm

Peak#	Ret. Time	Area	Area%	Height
1	13.455	66781	0.963	4201
2	18.419	6865495	99.037	265819
Total		6932276	100.000	270020

Supplementary Figure 256. HPLC Spectra of **6**

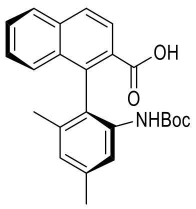

4

<Chromatogram>

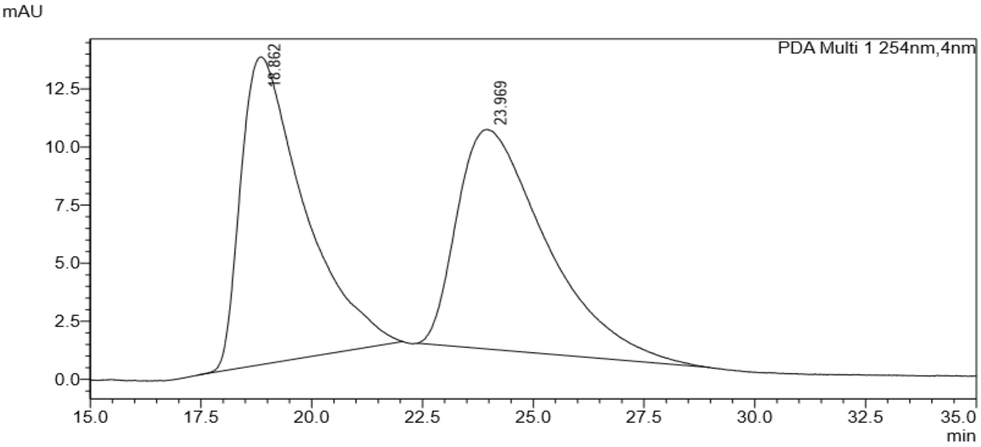

<Peak Table>

PDA Ch1 254nm

Peak#	Ret. Time	Area	Area%	Height
1	18.862	1321243	49.845	13244
2	23.969	1329458	50.155	9449
Total		2650701	100.000	22694

Supplementary Figure 257. HPLC Spectra of racemic **4**

<Chromatogram>

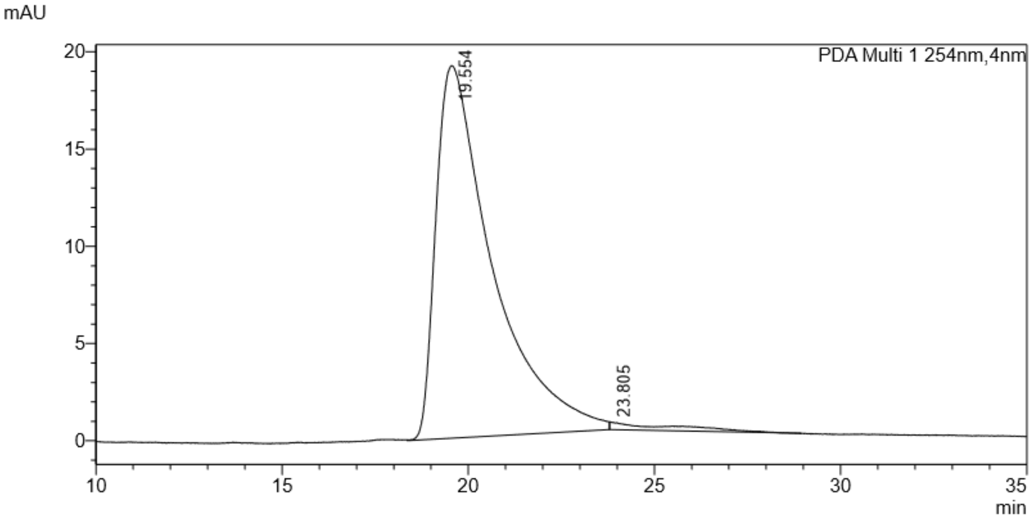

<Peak Table>

PDA Ch1 254nm

Peak#	Ret. Time	Area	Area%	Height
1	19.554	2018102	97.747	19157
2	23.805	46510	2.253	401
Total		2064611	100.000	19558

Supplementary Figure 258. HPLC Spectra of **4**

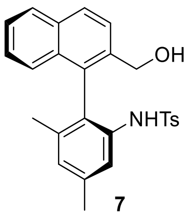

7

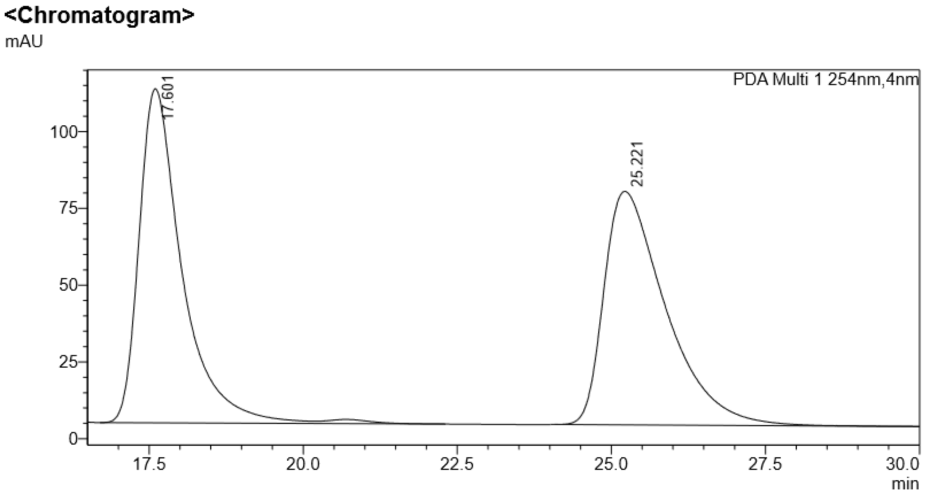

<Peak Table>

PDA Ch1 254nm

Peak#	Ret. Time	Area	Area%	Height
1	17.601	5316323	49.998	108802
2	25.221	5316761	50.002	76040
Total		10633084	100.000	184843

Supplementary Figure 259. HPLC Spectra of racemic **7**

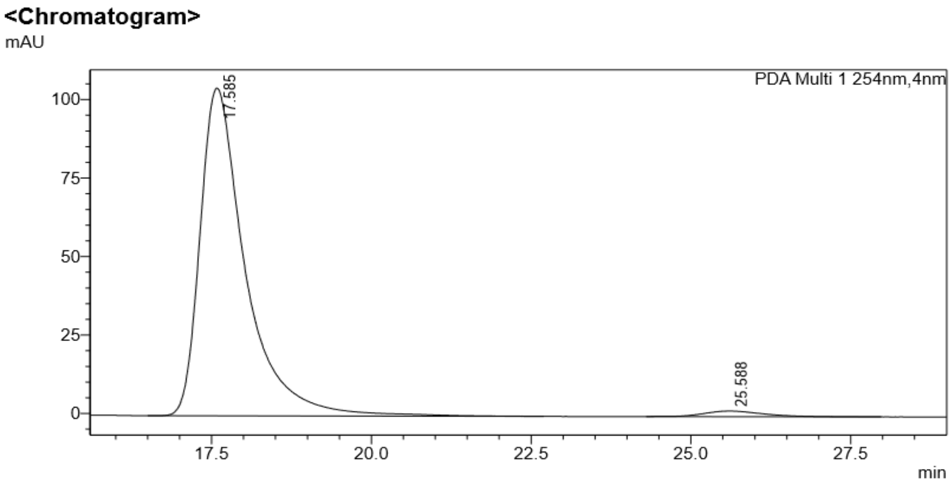

<Peak Table>

PDA Ch1 254nm

Peak#	Ret. Time	Area	Area%	Height
1	17.585	5146010	97.846	104382
2	25.588	113267	2.154	1772
Total		5259277	100.000	106153

Supplementary Figure 260. HPLC Spectra of **7**

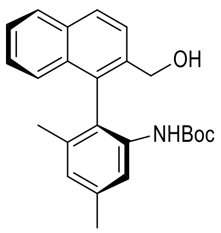

8

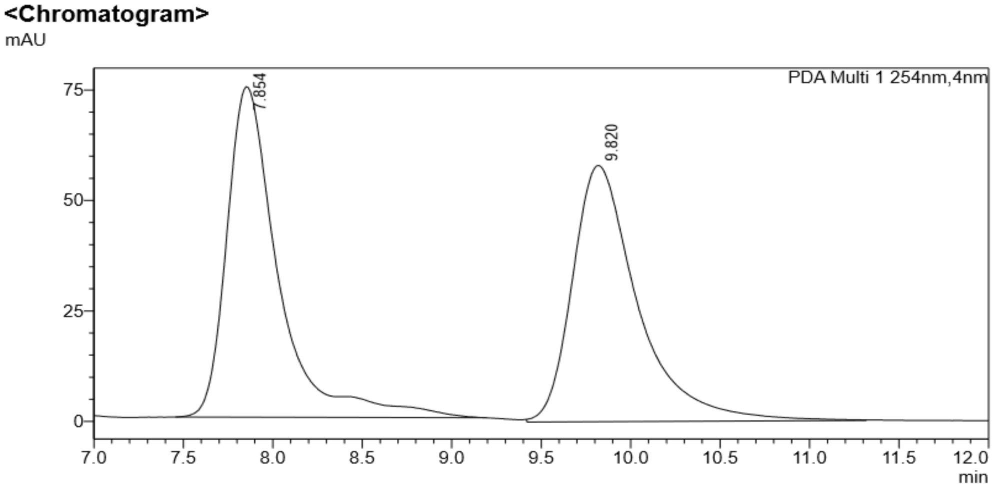

<Peak Table>

PDA Ch1 254nm

Peak#	Ret. Time	Area	Area%	Height
1	7.854	1506312	50.535	74786
2	9.820	1474392	49.465	57973
Total		2980703	100.000	132758

Supplementary Figure 261. HPLC Spectra of racemic **8**

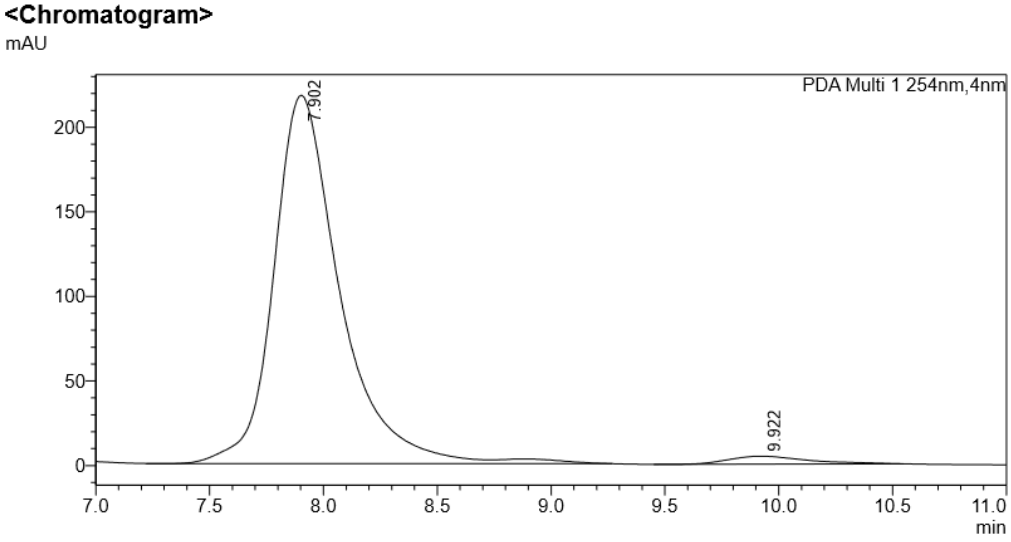

<Peak Table>

PDA Ch1 254nm

Peak#	Ret. Time	Area	Area%	Height
1	7.902	4425334	97.489	217835
2	9.922	113966	2.511	4688
Total		4539299	100.000	222523

Supplementary Figure 262. HPLC Spectra of **8**

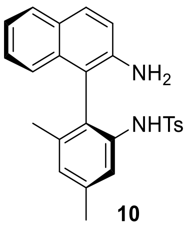

10

<Chromatogram>

mAU

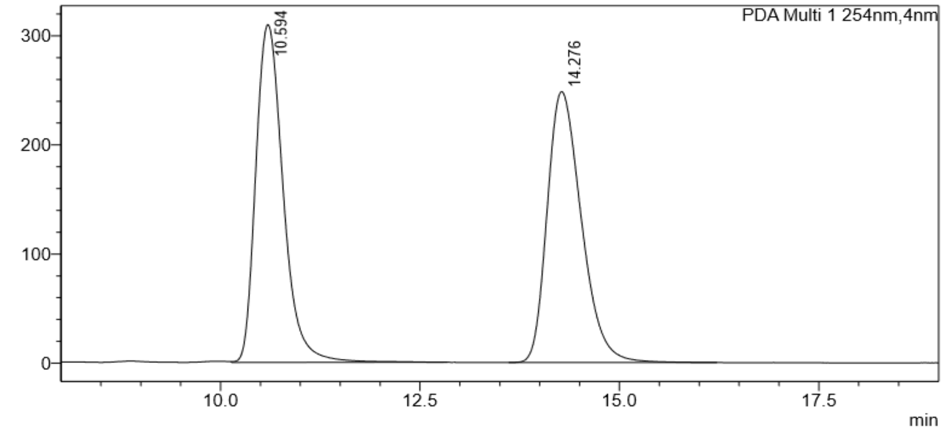

<Peak Table>

PDA Ch1 254nm

Peak#	Ret. Time	Area	Area%	Height
1	10.594	7395336	49.982	309545
2	14.276	7400750	50.018	248245
Total		14796086	100.000	557790

Supplementary Figure 263. HPLC Spectra of racemic **10**

<Chromatogram>

mAU

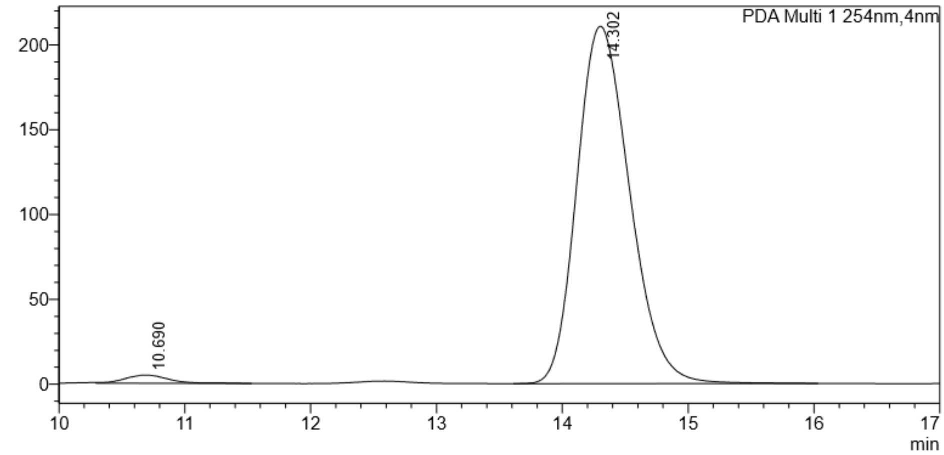

<Peak Table>

PDA Ch1 254nm

Peak#	Ret. Time	Area	Area%	Height
1	10.690	116644	1.864	4793
2	14.302	6140716	98.136	210634
Total		6257360	100.000	215427

Supplementary Figure 264. HPLC Spectra of **10**

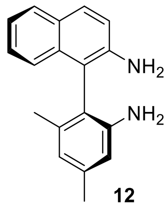

12

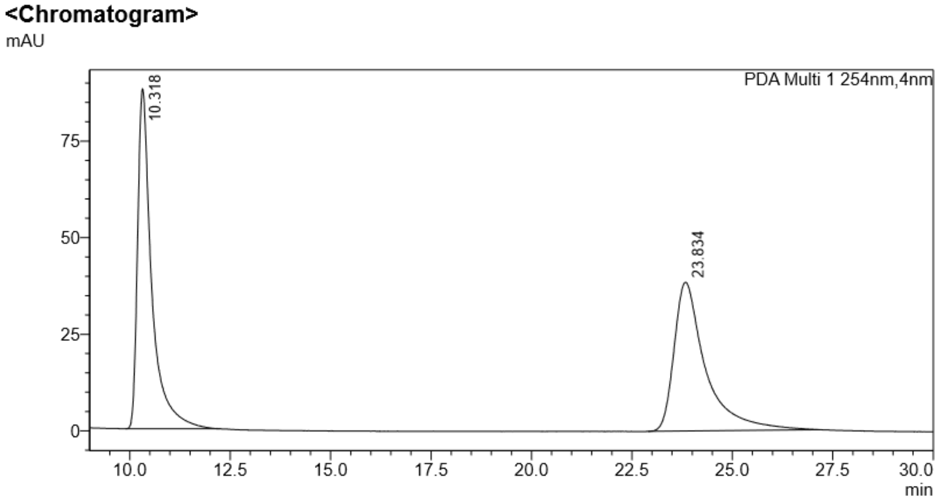

<Peak Table>

PDA Ch1 254nm

Peak#	Ret. Time	Area	Area%	Height
1	10.318	2154053	50.547	87962
2	23.834	2107439	49.453	38498
Total		4261492	100.000	126460

Supplementary Figure 265. HPLC Spectra of racemic **12**

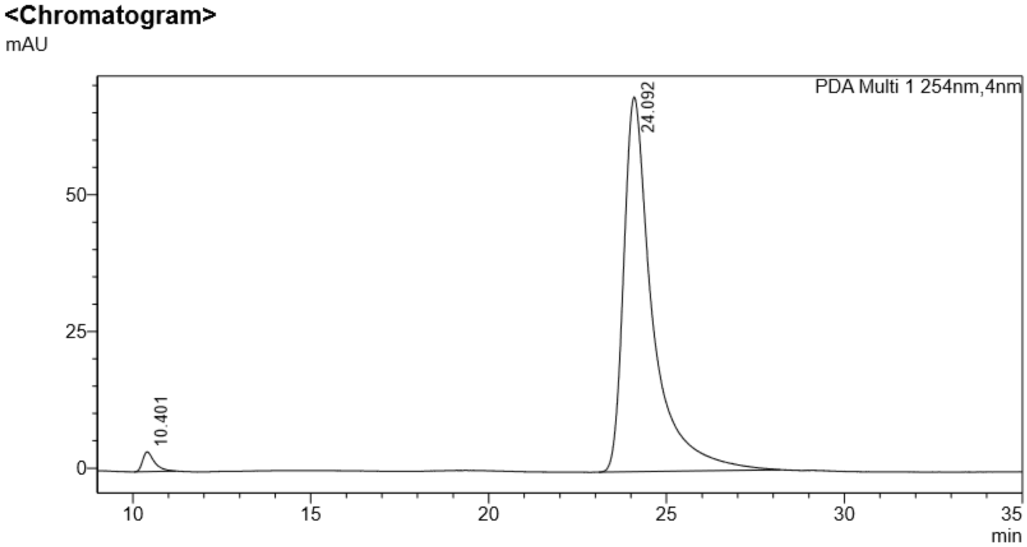

<Peak Table>

PDA Ch1 254nm

Peak#	Ret. Time	Area	Area%	Height
1	10.401	84040	2.125	3606
2	24.092	3870308	97.875	68522
Total		3954349	100.000	72128

Supplementary Figure 266. HPLC Spectra of **12**

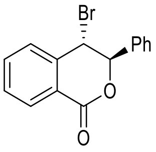

14

<Chromatogram>

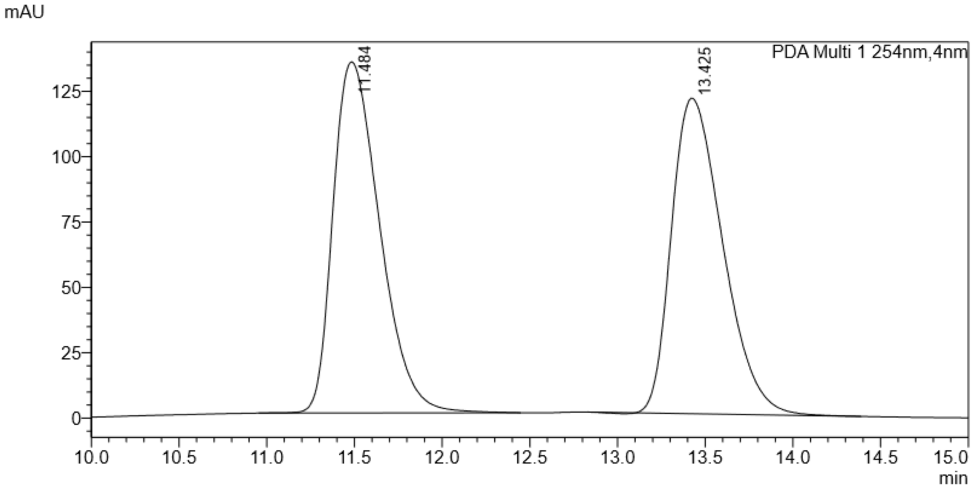

<Peak Table>

PDA Ch1 254nm

Peak#	Ret. Time	Area	Area%	Height
1	11.484	2487004	50.302	134262
2	13.425	2457140	49.698	120622
Total		4944144	100.000	254884

Supplementary Figure 267. HPLC Spectra of racemic **14**

<Chromatogram>

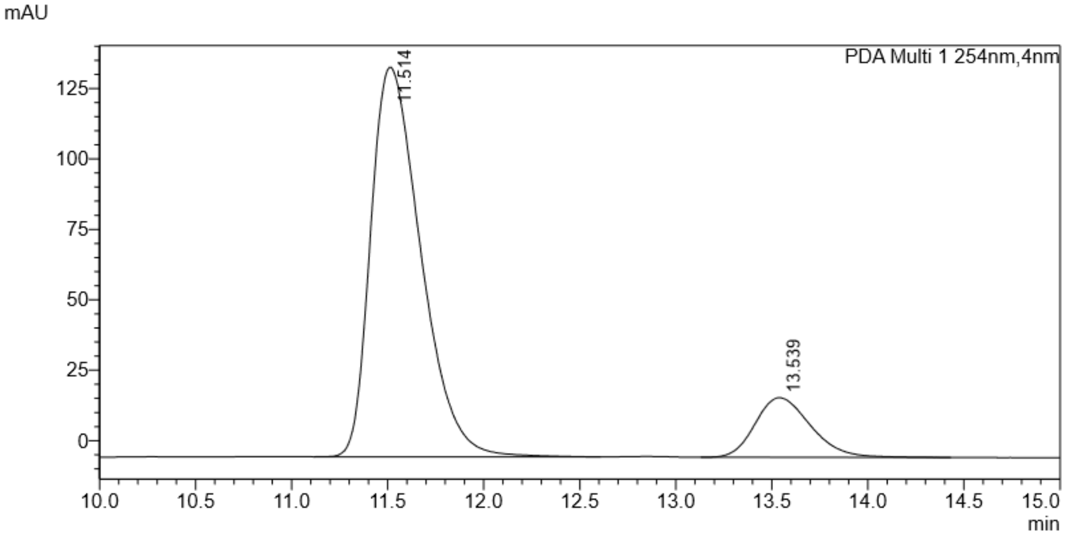

<Peak Table>

PDA Ch1 254nm

Peak#	Ret. Time	Area	Area%	Height
1	11.514	2509816	85.511	138246
2	13.539	425255	14.489	21154
Total		2935071	100.000	159400

Supplementary Figure 268. HPLC Spectra of **14**

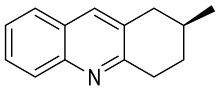

17

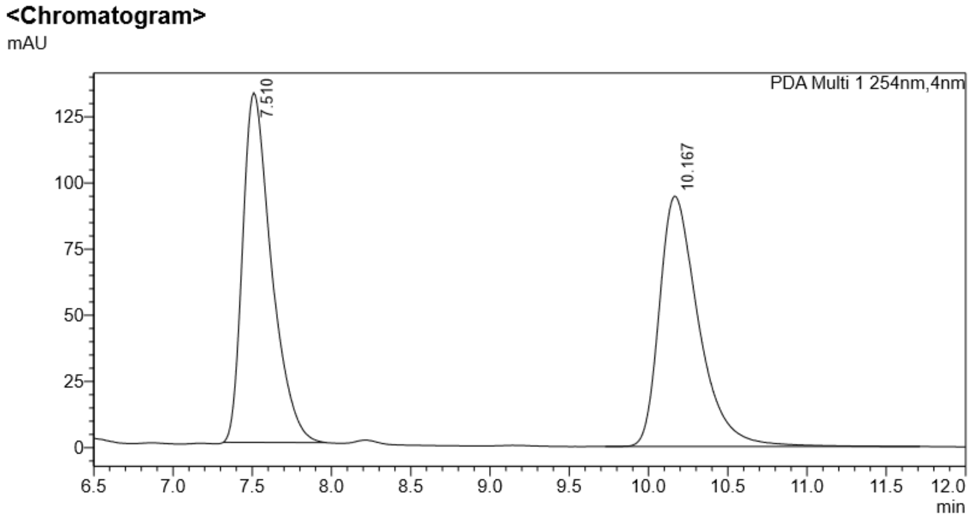

<Peak Table>

PDA Ch1 254nm

Peak#	Ret. Time	Area	Area%	Height
1	7.510	1670377	50.613	132076
2	10.167	1629906	49.387	94576
Total		3300283	100.000	226652

Supplementary Figure 269. HPLC Spectra of racemic **17**

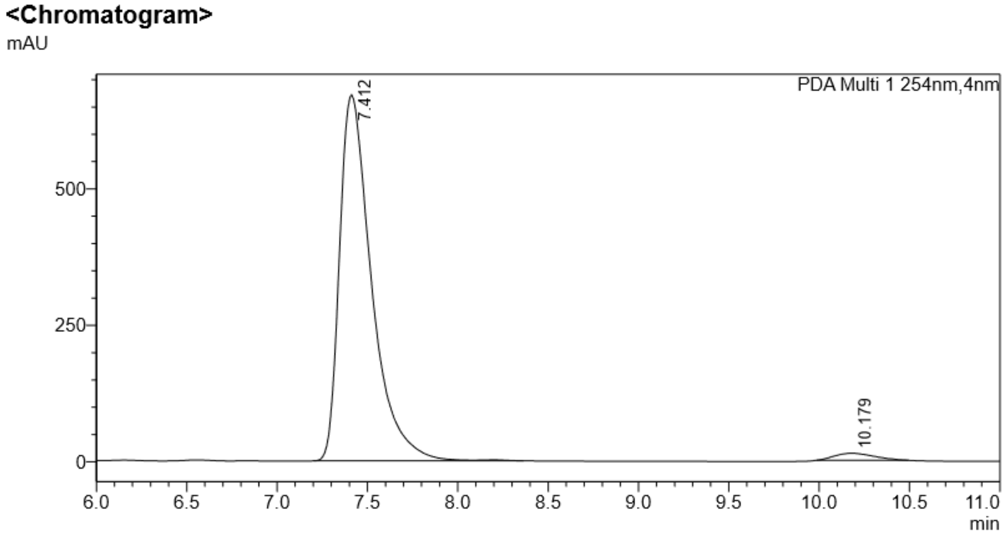

<Peak Table>

PDA Ch1 254nm

Peak#	Ret. Time	Area	Area%	Height
1	7.412	8269053	97.642	670717
2	10.179	199675	2.358	12940
Total		8468727	100.000	683657

Supplementary Figure 270. HPLC Spectra of **17**

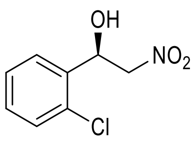

19

<Chromatogram>

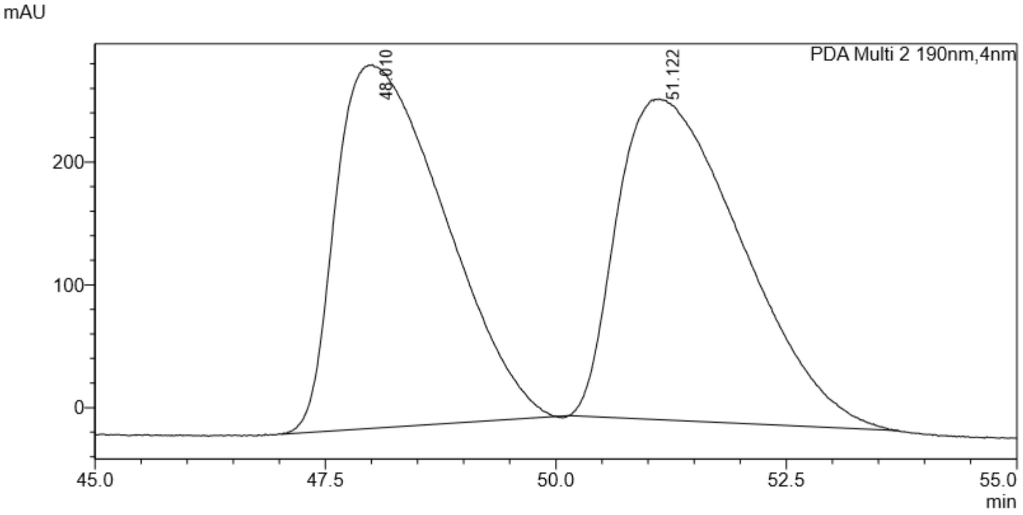

<Peak Table>

PDA Ch2 190nm

Peak#	Ret. Time	Area	Area%	Height
1	48.010	24243838	49.611	296236
2	51.122	24623862	50.389	261313
Total		48867700	100.000	557548

Supplementary Figure 271. HPLC Spectra of racemic **19**

<Chromatogram>

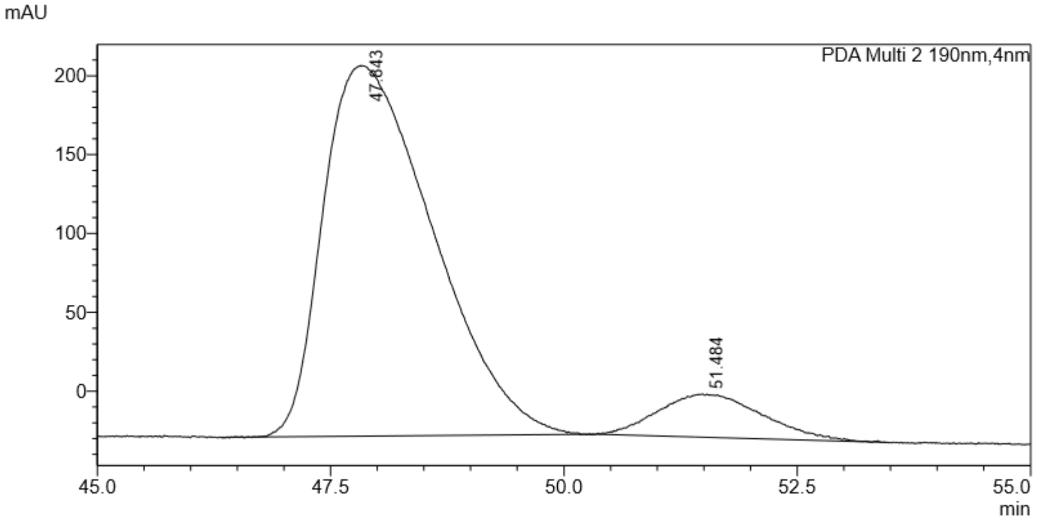

<Peak Table>

PDA Ch2 190nm

Peak#	Ret. Time	Area	Area%	Height
1	47.843	19225961	90.082	234856
2	51.484	2116792	9.918	27286
Total		21342753	100.000	262142

Supplementary Figure 272. HPLC Spectra of **19**

Supplementary References

1 Hande, S. M., Nakajima, M., Kamisaki, H., Tsukano, C. & Takemoto, Y. Flexible Strategy for Syntheses of Spirooxindoles using Palladium-Catalyzed Carbosilylation and Sakurai-Type Cyclization. *Org. Lett.* **13**, 1828-1831(2011).

2 Li, X. & Zou, G. Palladium-catalyzed acylative cross-coupling of amides with diarylborinic acids and sodium tetraarylborates. *J. Organomet. Chem.* **794**, 136-145(2015).

3 Zhao, K. et al. Enhanced Reactivity by Torsional Strain of Cyclic Diaryliodonium in Cu-Catalyzed Enantioselective Ring-Opening Reaction. *Chem*. **4**, 599-612(2018).

4 Rajeshkumar, V., Lee, T. -H. & Chuang, S. -C. Palladium-Catalyzed Oxidative Insertion of Carbon Monoxide to N-Sulfonyl-2-aminobiaryls through C-H Bond Activation: Access to Bioactive Phenanthridinone Derivatives in One Pot. *Org. Lett*, **15**, 1468-1471(2013).

5 Sopeña, S., Martin, E., Escudero-Adán, E. C. & Kleij, A. W. Pushing the Limits with Squaramide-Based Organocatalysts in Cyclic Carbonate Synthesis. *ACS Catal.* **7**, 3532-353(2017).

6 Kohler, M. C., Yost, J. M., Garnsey, M. R. & Coltart, D. M. Direct Carbon-Carbon Bond Formation via Soft Enolization: A Biomimetic Asymmetric Mannich Reaction of Phenylacetate Thioesters. *Org. Lett.* **12**, 3376-3379(2010).

7 Borrero, N. V., DeRatt, L. G., Barbosa, L. F., Abboud, K. A. & Aponick, A. Tandem Gold-Catalyzed Dehydrative Cyclization/Diels-Alder Reactions: Facile Access to Indolocarbazole Alkaloids. *Org. Lett.* **17**, 1754-1757(2015).

8 Nishiyori, R. et al. Design of Chiral Bifunctional Dialkyl Sulfide Catalysts for Regio-, Diastereo-, and Enantioselective Bromolactonization. *Chem. Eur. J.* **24**, 16747-16752(2018).

9 Ma, G. N., Zhang, Y. -P. & Shi, M. *L*-Proline Diamides with an Axially Chiral Binaphthylene Backbone as Efficient - Organocatalysts for Direct Asymmetric Aldol Reactions: The Effect of Acetic Acid. *Synthesis*. **2**, 197–208(2007).

10 D. -J. Cheng. et al. Highly Enantioselective Kinetic Resolution of Axially Chiral BINAM Derivatives Catalyzed by a Brønsted Acid. *Angew. Chem. Int. Ed.* **53**, 3684-3687(2014).

11 Vallavoju, N. et al. Evaluating Thiourea Architecture for Intramolecular [2+2] Photocycloaddition of 4-Alkenylcoumarins. *Adv. Synth. Catal.* **356**，2763-2768(2014).

12 Frisch, M. J. et al. Gaussian16, Gaussian, Inc., Wallingford, CT(2016).

13 Zhao, Y. & Truhlar, D. G. The M06 suite of density functionals for main group thermochemistry, thermochemical kinetics, noncovalent interactions, excited states, and transition elements: two new functionals and systematic testing of four M06-class functionals and 12 other functionals. *Theor. Chem. Acc.* **120**, 215-241(2008).

14 Zhao, Y. & Truhlar, D. G. Exploring the Limit of Accuracy of the Global Hybrid Meta Density Functional for Main-Group Thermochemistry, Kinetics, and Noncovalent Interactions. *J Chem Theory Comput*. **4**, 1849-1868(2008).
15 Zhao, Y. & Truhlar, D. G. Truhlar, Density Functionals with Broad Applicability in Chemistry. *Acc. Chem. Res.* **41**, 157-167(2008).
16 Mennucci, B. & Tomasi, J. Continuum solvation models: A new approach to the problem of solute's charge distribution and cavity boundaries. *J. Chem. Phys.* **106,** 5151-5158(1997).
17 Barone, V. & Cossi, M. Quantum Calculation of Molecular Energies and Energy Gradients in Solution by a Conductor Solvent Model. *J. Phys. Chem. A.* **102**, 1995-2001(1998).
18 Lu, T. & Chen, F. Multiwfn: A multifunctional wavefunction analyzer. *J. Comput. Chem.* **33**, 580-592(2012).
