# Supplementary material for: Organocatalytic asymmetric N-sulfonyl amide C-N bond activation to access axially chiral biaryl amino acids
Source: Nat Commun. 2020 Feb 19;11:946. doi: 10.1038/s41467-020-14799-8 (PMC7031291; doi:10.1038/s41467-020-14799-8)
Supplement: Supplementary file 2 — Source Data [file 41467_2020_14799_MOESM2_ESM.pdf]

Absolute SPE and GFE of the optimized structures.

	SPE (a.u.)	GFEC (a.u.)		SPE (a.u.)	GFEC (a.u.)
**Cat**	-2279.838409	0.492063	**Int2S**	-3998.900343	0.877879
**1c**	-1603.268832	0.302116	**PS**	-1719.023155	0.354059
**2k**	-115.7117124	0.029527	**TS1R**	-3998.872356	0.877766
**Int1**	-3998.894036	0.875675	**Int1R**	-3998.906144	0.882368
**TS1S**	-3998.876628	0.876157	**TS2R**	-3998.900401	0.879124
**Int1S**	-3998.903638	0.882293	**Int2R**	-3998.90604	0.880842
**TS2S**	-3998.896603	0.87505	**PR**	-1719.021702	0.354695
**TS3**	-1718.964968	0.35556			

The single-point energy (SPE) computed at the M06-2X-GD3/6-311++G(2d, 2p)/IEF-PCM$_{o\text{-}xylene}$ level and Gibbs free energy correction (GFEC) calculated at the M06-2X /6-31G(d, p)/IEF-PCM$_{o\text{-}xylene}$ of all reactants, intermediates, transition states, and products involved in the catalytic cycle.

Geometrical Coordinates of the Listed Complexes

Cat

C	-2.48098100	0.36320800	0.41002500
H	-2.64770700	-0.20702200	-0.50440300
C	-0.18622400	-0.19389500	-0.24160100
C	-0.39706700	-1.10520800	-1.38667500
C	1.11477600	-1.24450800	-1.48938200
C	1.20419200	-0.29881600	-0.34792000
C	3.55550600	0.23427900	0.27838000
C	4.19378200	-0.56091100	-0.67569300
H	3.62124600	-1.17123600	-1.36836900
C	5.58378400	-0.56827100	-0.72312200
C	5.70261100	0.96803000	1.09450100
C	4.31901100	1.00019200	1.16708100
H	3.83525600	1.62381700	1.91266700
N	-1.02745500	0.46445100	0.55219800
H	-0.67251000	1.12633900	1.22943000
N	2.16415900	0.30656600	0.39687100
H	1.83580600	0.90996900	1.13834100
O	-1.36308400	-1.51761800	-1.99346900
O	1.87085400	-1.85685400	-2.20730100
F	6.40732400	-0.66687300	-2.92432000
F	5.56539900	-2.47540400	-2.10565200
F	7.59472300	2.29926300	1.51685000
F	5.80752300	2.72810800	2.66036300
C	6.35787700	0.18683400	0.14864600
H	7.44023600	0.16608600	0.09505200
C	6.51830000	1.74469500	2.08983800

C	6.26308000	-1.37684600	-1.79530300
F	7.48985200	-1.76421300	-1.41594500
F	6.96493900	0.95516200	3.07805200
C	-3.12606300	1.74509200	0.22666300
C	-4.63942500	1.59780700	-0.11078500
H	-3.01097900	2.30403800	1.16421300
C	-2.47927300	1.87862800	-2.10917300
C	-3.09035800	3.83445200	-0.89607100
C	-4.87645500	2.20367200	-1.50162600
H	-4.93525400	0.54155700	-0.12215400
H	-5.25759000	2.08562900	0.64861400
H	-2.05693300	2.58463100	-2.83053700
H	-1.83125900	0.99855200	-2.11555100
C	-3.93461400	1.49988400	-2.48915700
C	-4.52378000	3.70981300	-1.49970900
H	-2.47274400	4.49966100	-1.50713900
H	-3.13919800	4.26865600	0.10985600
H	-5.92074100	2.06904000	-1.79838200
H	-4.15894400	1.80213200	-3.51672500
H	-4.08169500	0.41383200	-2.43292500
H	-4.50797800	4.04564500	-2.54465100
N	-2.41879900	2.53386400	-0.79191400
C	-5.51764800	4.55422400	-0.75852600
H	-5.62073400	4.35338600	0.30934700
C	-6.25139600	5.51761000	-1.30841200
H	-6.17023300	5.75276000	-2.36670600
H	-6.95539600	6.10247200	-0.72575500
C	-3.09111800	-0.38103800	1.59094000
C	-3.63459400	-1.69440000	1.45440300
C	-3.12379400	0.20645700	2.83433100
C	-3.63131000	-2.42827500	0.23452300
C	-4.20976300	-2.28179900	2.61890100
C	-3.69997600	-0.47882200	3.92585500
H	-2.71767900	1.20042200	2.99893000
C	-4.19581900	-3.68264800	0.18622800
H	-3.15238500	-2.02348000	-0.64703400
C	-4.79076600	-3.57802200	2.52288100
H	-3.72226000	-0.00547600	4.90546300
C	-4.79132800	-4.25939600	1.34249700
H	-5.22642000	-3.99720800	3.42338400
H	-5.22664500	-5.24856000	1.25018500
N	-4.23630700	-1.67286300	3.83385800
O	-4.23503700	-4.46285000	-0.91637200
C	-3.61901000	-3.95929200	-2.09075000

H	-3.74360800	-4.72893900	-2.85067000
H	-4.10240100	-3.03251900	-2.42178500
H	-2.55507100	-3.76052000	-1.92750900

1c

C	-0.53784200	-2.08189500	-0.16325000
O	-0.43002800	-3.28070000	-0.03827900
C	-1.75809900	-1.34694100	0.24058500
C	-2.00890900	-0.04193300	-0.16128300
C	-2.67961500	-2.09023100	1.02193300
C	-3.34807000	0.47237400	0.04080000
C	-3.85313300	-1.52111300	1.40996900
H	-2.40190300	-3.09608400	1.31332200
C	-3.88753300	1.59719500	-0.64281100
C	-4.23288000	-0.24939300	0.89619700
H	-4.54451300	-2.05190100	2.05757900
C	-5.16057600	2.04889700	-0.39061200
H	-3.31987400	2.07224000	-1.43044400
C	-5.52632800	0.26224700	1.16958300
C	-5.97921900	1.40286100	0.55858000
H	-5.54545400	2.89781300	-0.94615400
H	-6.16437400	-0.29297600	1.85109300
H	-6.97540400	1.77999600	0.76478300
N	0.49653400	-1.29518400	-0.71943400
C	-0.91151500	0.70861400	-0.79205200
C	-0.92893900	2.10192000	-0.98973500
C	0.26696300	0.02910300	-1.17335300
C	0.07204200	2.76073600	-1.68307400
H	-1.72749300	2.68448700	-0.55285900
C	1.22783600	0.67243200	-1.96343700
C	1.12460200	2.02823300	-2.22646400
H	0.02192600	3.83635200	-1.81036800
H	2.05991400	0.10638400	-2.36251800
H	1.88436900	2.51301100	-2.82999300
S	2.13931400	-1.83593500	-0.48819000
O	2.73777400	-2.03752300	-1.79585400
O	2.11889200	-2.90247200	0.48696200
C	2.87091900	-0.40932200	0.27725300
C	4.03608700	0.11358700	-0.26489400
C	2.30415300	0.10594400	1.44079700
C	4.64517400	1.18892700	0.37591600
H	4.44903700	-0.31131400	-1.17388700
C	2.92069700	1.18374200	2.05716800
H	1.39128100	-0.32147700	1.84645200

C	4.09728500	1.73913900	1.53535900
H	5.55851700	1.60741400	-0.03562500
H	2.48705200	1.60352700	2.96017600
C	4.74494600	2.91253700	2.22096100
H	5.69134400	3.17685400	1.74606200
H	4.09009200	3.78835400	2.18867900
H	4.93933300	2.68818800	3.27332500

2k

O	-0.74554700	0.12255300	-0.00001200
H	-1.13302400	-0.75871100	0.00003600
C	0.65958800	-0.01965900	-0.00000300
H	1.08431900	0.98612100	0.00025600
H	1.02771600	-0.54511500	0.89056900
H	1.02783800	-0.54476300	-0.89075000

Int1

C	2.38993500	-1.89582000	1.37552400
H	2.35416700	-2.87024200	1.87783400
C	0.10678200	-2.57022100	0.79517600
C	-0.05330300	-3.85089200	1.50707200
C	-1.49849300	-3.87620800	1.04150400
C	-1.22895700	-2.58065600	0.36539500
C	-3.31702800	-1.68815100	-0.54955700
C	-4.17098500	-2.27173800	0.39227800
H	-3.77833600	-2.83933600	1.22643300
C	-5.54549400	-2.12709500	0.24822400
C	-5.23589700	-0.80603200	-1.70985500
C	-3.86077000	-0.96892600	-1.61827900
H	-3.20516800	-0.51734700	-2.35338400
N	1.15997100	-1.78020200	0.60093800
H	1.03117600	-0.95590600	0.02163900
N	-1.92153300	-1.74429400	-0.43318900
H	-1.41888500	-0.94550900	-0.82005000
O	0.69312200	-4.57696400	2.13944400
O	-2.43471700	-4.63467000	1.16081700
F	-7.57371800	-2.08915600	1.44195300
F	-5.86681900	-2.99389200	2.41669800
F	-5.68315100	1.38452800	-2.42926800
F	-5.09656400	-0.04236400	-3.93389400
C	-6.10024200	-1.38450000	-0.78781100
H	-7.17294700	-1.25450900	-0.86990900
C	-5.77465000	0.08991900	-2.78691900
C	-6.45894300	-2.80671400	1.22907800

F	-6.85145500	-4.01171000	0.78971100
F	-7.06722900	-0.15234000	-3.04588900
C	-1.10219500	1.94242000	-0.96729900
C	2.47883200	-0.78506600	2.44446500
C	3.58380700	-1.06117200	3.49625500
H	2.67847800	0.15850500	1.92102000
C	0.88522000	-1.62742500	4.07925000
C	1.26474600	0.73473100	3.82378100
C	3.04219100	-0.65849200	4.87768700
H	3.84481700	-2.12711900	3.50664500
H	4.50296500	-0.51780900	3.25923100
H	-0.13913700	-1.47973300	4.43376800
H	0.91047800	-2.58574800	3.55460600
C	1.90343400	-1.61556300	5.24894600
C	2.46312900	0.77413700	4.82411400
H	0.31512500	0.87950100	4.34870800
H	1.34936600	1.53017200	3.07198400
H	3.84203500	-0.70164100	5.62252200
H	1.42964900	-1.28365400	6.17869200
H	2.29636200	-2.62146600	5.42375200
H	2.09290000	1.02432300	5.82549800
N	1.18355700	-0.55301500	3.11167200
C	3.49240600	1.79727000	4.44036300
H	3.89578100	1.71942700	3.42903500
C	3.92612100	2.77288700	5.23349400
H	3.54034300	2.89312600	6.24259600
H	4.67891600	3.48285100	4.90542900
C	3.56717100	-1.88413500	0.40392900
C	3.70014200	-2.93323900	-0.56025700
C	4.48275300	-0.85973800	0.35609000
C	2.86128900	-4.07815900	-0.57795900
C	4.72817800	-2.80482600	-1.53617200
C	5.47658800	-0.83727300	-0.64994300
H	4.45922100	-0.04704100	1.07079300
C	3.02925200	-5.04079300	-1.54757700
H	2.11003900	-4.20640800	0.18856500
C	4.86927400	-3.81825500	-2.52505800
H	6.17914000	-0.00608600	-0.68636400
C	4.04385500	-4.90388100	-2.53603900
H	5.65561200	-3.69513600	-3.26184900
H	4.13660900	-5.68534300	-3.28233900
N	5.59648700	-1.75507600	-1.57809100
O	2.28028100	-6.15899800	-1.65031400
C	1.19332800	-6.29574700	-0.74813500

H	0.70054000	-7.23126000	-1.00717000
H	1.53446300	-6.32954000	0.29183200
H	0.48785800	-5.46345200	-0.86472900
O	-0.59188400	0.42468900	1.19522100
H	0.02744500	0.00748700	1.84293000
C	-1.86853300	0.43022400	1.80110100
H	-2.60919500	0.68874900	1.03763000
H	-2.13261700	-0.55881300	2.20459400
H	-1.93411700	1.16788300	2.61277700
O	-1.85440900	1.07174600	-1.36104200
C	-1.55795600	3.16393900	-0.28549300
C	-0.64393300	4.07482600	0.22194600
C	-2.95665200	3.39065900	-0.25090900
C	-1.12641900	5.37068800	0.62317700
C	-3.43267700	4.55381700	0.27707200
H	-3.61348500	2.62769100	-0.65583200
C	-0.27925700	6.48542600	0.87017900
C	-2.53570900	5.57869100	0.69159500
H	-4.50057600	4.74000200	0.33838300
C	-0.79505100	7.69965200	1.25108500
H	0.78651700	6.39488100	0.70228200
C	-3.03659900	6.83391400	1.12091500
C	-2.18739700	7.87046500	1.41148300
H	-0.12700500	8.53884900	1.41434600
H	-4.11274400	6.96360300	1.19004900
H	-2.58183200	8.82943800	1.73053700
N	0.27120200	1.91073700	-1.27344700
C	0.74464800	3.61415700	0.37658100
C	1.60799900	4.13162800	1.35474300
C	1.18744700	2.49657700	-0.36736600
C	2.89624900	3.64801100	1.52091900
H	1.24546100	4.90391100	2.02074600
C	2.46328700	1.96923700	-0.15660800
C	3.32601800	2.57252500	0.74880500
H	3.54308300	4.07790000	2.27761900
H	2.78361200	1.07063200	-0.66794500
H	4.32766600	2.17173100	0.87581900
S	0.66800600	0.77908400	-2.52642200
O	-0.28090900	1.01380500	-3.59403400
O	0.78049600	-0.54068600	-1.90900800
C	2.26286100	1.28785100	-3.08873000
C	2.40182000	2.54370300	-3.67383900
C	3.31391400	0.37821200	-3.02612700
C	3.64260300	2.89837500	-4.18417200

H	1.55830900	3.22530900	-3.72441700
C	4.54443900	0.75108900	-3.55471900
H	3.17161800	-0.59593900	-2.56370000
C	4.72410300	2.00959500	-4.13766900
H	3.77534500	3.87789900	-4.63354500
H	5.37149700	0.04832000	-3.50160400
C	6.05180400	2.39393200	-4.73278800
H	6.07358500	2.14763300	-5.79924400
H	6.23277800	3.46679000	-4.63733600
H	6.87088200	1.85601700	-4.25144900

TS1R

C	1.06007500	2.94475800	-0.36007600
H	0.63166300	3.76726300	-0.94359500
C	-1.26153500	2.22755400	-0.62447900
C	-1.82864100	3.28423300	-1.47426300
C	-3.17437100	2.59940600	-1.35957100
C	-2.49297100	1.55255800	-0.54767700
C	-4.00858500	-0.29695500	0.07191600
C	-5.20649000	0.24465100	-0.40238100
H	-5.24424400	1.24575000	-0.82114000
C	-6.35876100	-0.53251000	-0.35536500
C	-5.15469700	-2.34532600	0.61343400
C	-3.98649500	-1.59629300	0.58414900
H	-3.05537600	-2.02445700	0.94583300
N	-0.04712100	2.02416800	-0.10435600
H	0.09398800	1.17456500	0.44787500
N	-2.79010500	0.39410800	0.06451500
H	-1.98266500	-0.12421900	0.43603900
O	-1.39077700	4.28778900	-2.01171500
O	-4.29505500	2.85043100	-1.74379100
F	-7.46959300	0.86518800	-1.89191900
F	-8.19610400	0.85439400	0.13631200
F	-6.27113900	-4.34756300	1.16928400
F	-4.26359000	-4.50907500	0.38566800
C	-6.35763100	-1.82919000	0.14754300
H	-7.26389500	-2.42126400	0.16760900
C	-5.06983300	-3.75083600	1.14126900
C	-7.64923800	0.08416000	-0.81830800
F	-8.55996400	-0.84585800	-1.14058500
F	-4.57673400	-3.77894200	2.38700800
C	0.53748300	-1.28382300	-0.06177100
C	2.18046100	2.30954800	-1.20659000
C	3.11052700	3.40073700	-1.78236200

H	2.74942700	1.60343500	-0.59226700
C	1.08471300	2.32609600	-3.39744400
C	2.76202100	0.66579900	-2.89333800
C	3.49460300	3.01176100	-3.21525500
H	2.59272200	4.36776400	-1.79798300
H	3.98642900	3.51781500	-1.14336600
H	0.56026100	1.67032500	-4.09738500
H	0.34766900	3.00226100	-2.95884000
C	2.23071500	3.10706100	-4.08746400
C	3.98066900	1.55133500	-3.25030600
H	2.35178400	0.14809700	-3.76513500
H	3.02810600	-0.09258600	-2.15451700
H	4.26597400	3.68420900	-3.60062900
H	2.43390000	2.69569900	-5.08120900
H	1.93906200	4.15212300	-4.21905700
H	4.27135900	1.31673100	-4.28305200
N	1.64665400	1.47038400	-2.31759700
C	5.16499400	1.23068300	-2.36711500
H	5.28132800	0.17347700	-2.11828200
C	6.07019900	2.09113400	-1.90860400
H	6.02644300	3.15425400	-2.12915900
H	6.90275500	1.75093600	-1.30182200
C	1.59008600	3.52582700	0.95140500
C	0.78318100	4.43676200	1.70507300
C	2.81785400	3.18802800	1.46957700
C	-0.47739700	4.91599800	1.25906600
C	1.29153800	4.88705300	2.95563100
C	3.22741700	3.70338800	2.72212300
H	3.49803100	2.52354900	0.95037200
C	-1.20236400	5.78924100	2.03911500
H	-0.84998400	4.63003100	0.28707000
C	0.51234600	5.78633100	3.73596200
H	4.19963000	3.41895800	3.12141700
C	-0.70173100	6.22470600	3.29684300
H	0.92377200	6.10869300	4.68633700
H	-1.30967000	6.90944300	3.87803700
N	2.50097400	4.51092000	3.45418100
O	-2.40663100	6.30009000	1.70338800
C	-3.00611200	5.82755700	0.50820100
H	-3.97092400	6.32625700	0.43376100
H	-2.39642800	6.06327400	-0.37018500
H	-3.15913000	4.74231200	0.55842400
O	0.40361400	-0.57106800	-1.64590400
H	0.88382900	0.47991300	-1.88445200

C	-0.83174300	-0.80062800	-2.30586300
H	-1.21956700	0.14332800	-2.70517300
H	-0.69242200	-1.51776400	-3.12141200
H	-1.55761200	-1.20977300	-1.59656800
O	-0.26286600	-0.71973900	0.72370400
C	2.02487300	-1.11871300	0.16972500
C	2.92522100	-1.89245300	-0.54553800
C	2.45060400	-0.26102200	1.20658500
C	4.31300300	-1.87193800	-0.17396800
C	3.78371100	-0.14384400	1.49716000
H	1.70299800	0.28821200	1.77176500
C	5.28002900	-2.78488200	-0.68355000
C	4.74051200	-0.94612300	0.82496600
H	4.12087800	0.52781600	2.28314600
C	6.59552200	-2.71689600	-0.29750600
H	4.96785000	-3.57608400	-1.35290100
C	6.11322400	-0.88428000	1.18552900
C	7.02781900	-1.73766500	0.62630700
H	7.30776900	-3.43369800	-0.69265800
H	6.41861700	-0.15565000	1.93207700
H	8.07306200	-1.68766200	0.91281900
N	0.22345700	-2.67353700	-0.45736700
C	2.37683000	-2.74739100	-1.61928300
C	3.07084700	-3.06785100	-2.79391400
C	1.05092000	-3.20121600	-1.49639000
C	2.51267400	-3.90817400	-3.74934200
H	4.04943700	-2.63333900	-2.97056200
C	0.49583500	-4.06100000	-2.44152500
C	1.23714800	-4.43552700	-3.55559400
H	3.07070400	-4.14567700	-4.64876000
H	-0.52199500	-4.40544600	-2.29830800
H	0.80275100	-5.10306100	-4.29192000
S	-0.21821500	-3.73935300	0.80644300
O	-0.09916000	-5.06969600	0.23523000
O	-1.47883400	-3.29180200	1.36790900
C	1.05223100	-3.50055600	2.01183600
C	2.30880200	-4.05480500	1.76958200
C	0.82229000	-2.63704800	3.07581300
C	3.36132200	-3.69978100	2.60003300
H	2.45500500	-4.73065000	0.93165500
C	1.88689800	-2.31609900	3.91268500
H	-0.16541500	-2.21384300	3.22013900
C	3.16760300	-2.81639100	3.66990600
H	4.35537700	-4.09519700	2.40692500

H	1.72606300	-1.63955100	4.74688700
C	4.34376000	-2.38504400	4.50330700
H	4.93829500	-3.24452800	4.82382700
H	4.99909700	-1.73769500	3.90874100
H	4.02771300	-1.83095300	5.38901400

TS1S

C	-3.39600200	-0.23541000	0.18565400
H	-3.93876500	-0.75120800	-0.60603600
C	-1.71339600	-1.84397400	-0.52356100
C	-2.46576600	-2.71227100	-1.44711700
C	-1.24534000	-3.61908500	-1.51470800
C	-0.56086600	-2.63360000	-0.64299700
C	1.80900900	-3.19884000	-0.17223800
C	1.86929700	-4.43489000	-0.82660100
H	0.99457000	-4.84126500	-1.32784600
C	3.07519600	-5.12434700	-0.85016200
C	4.14698600	-3.39997400	0.39484600
C	2.95425100	-2.69303600	0.45219400
H	2.91213500	-1.73464000	0.96628500
N	-2.02202500	-0.72033700	0.12158100
H	-1.28405400	-0.23203600	0.63896900
N	0.66244500	-2.41378000	-0.10930400
H	0.78809300	-1.47141500	0.27275900
O	-3.56248000	-2.65941700	-1.97334600
O	-0.97630600	-4.66537400	-2.06207100
F	4.34508700	-6.76147000	-1.97011800
F	2.26532300	-6.56240100	-2.53280000
F	5.53933600	-1.52115600	0.61436900
F	5.20752900	-2.69998200	2.38308200
C	4.23000600	-4.62533500	-0.25292200
H	5.16467600	-5.17007700	-0.30204400
C	5.34953400	-2.78266300	1.04688500
C	3.11900000	-6.47786500	-1.50571600
F	2.79420800	-7.45021800	-0.64021300
F	6.47083800	-3.46692700	0.80638000
C	0.70658200	1.40531100	-0.20569200
C	-3.43357600	1.27074600	-0.10674600
C	-4.88768400	1.81546000	-0.13440300
H	-2.83052600	1.78140300	0.65638000
C	-3.40356200	0.83288900	-2.52853600
C	-2.91237500	3.03160600	-1.66583500
C	-5.24766300	2.21298500	-1.57293300
H	-5.58383200	1.04636900	0.21558800

H	-4.99185000	2.66354800	0.54800800
H	-2.97623900	1.24181800	-3.44928400
H	-3.12422200	-0.22221700	-2.47378900
C	-4.93645600	1.02170400	-2.49023200
C	-4.37023100	3.39993300	-2.02850700
H	-2.20520400	3.31262900	-2.45207600
H	-2.59901000	3.54414200	-0.75411200
H	-6.30500600	2.48349900	-1.63574800
H	-5.31781700	1.19822400	-3.49964100
H	-5.42865700	0.11803800	-2.11103600
H	-4.46562100	3.49401700	-3.11764000
N	-2.76676600	1.57588500	-1.40650800
C	-4.75863200	4.71342900	-1.41299800
H	-4.71597600	4.76856800	-0.32392400
C	-5.12860500	5.78692900	-2.10441800
H	-5.17450300	5.77068900	-3.19031400
H	-5.39991700	6.71497400	-1.61252000
C	-4.06138600	-0.55108500	1.52026700
C	-5.15138700	-1.46976100	1.61264500
C	-3.62673100	0.06500000	2.66967600
C	-5.66916900	-2.20282200	0.50677200
C	-5.74058700	-1.64482400	2.89843200
C	-4.27631200	-0.20258800	3.89527900
H	-2.78262300	0.74734600	2.65957000
C	-6.73284800	-3.05817600	0.68461000
H	-5.19956100	-2.13317100	-0.46537300
C	-6.84725600	-2.52943000	3.03566300
H	-3.92773700	0.28865300	4.80144400
C	-7.33637300	-3.21415400	1.96302800
H	-7.27837400	-2.63878300	4.02495600
H	-8.17462800	-3.89684800	2.05083300
N	-5.30253400	-1.01167300	4.01914900
O	-7.28416300	-3.80972400	-0.29566800
C	-6.69301300	-3.73546800	-1.58222400
H	-7.25510300	-4.42334900	-2.21226600
H	-6.76726700	-2.72013900	-1.99064300
H	-5.63787100	-4.02558300	-1.55569700
O	-0.27382000	1.49244000	-1.62736200
H	-1.38491300	1.43620300	-1.41154800
C	0.08605200	0.46032500	-2.53849400
H	0.90471400	0.80368000	-3.17440600
H	0.39439700	-0.43594400	-1.98783200
H	-0.77889600	0.21646600	-3.16055800
O	0.54289800	0.28445500	0.33751300

C	2.07878800	1.73655900	-0.74784500
C	2.44041100	3.04909500	-0.98540000
C	3.00909300	0.68125400	-0.86494500
C	3.83173100	3.35881300	-1.18031500
C	4.31190200	0.94127100	-1.18358000
H	2.66014300	-0.33133300	-0.70104900
C	4.35652700	4.68101200	-1.13879600
C	4.76076300	2.28268700	-1.30611800
H	5.02938200	0.13255900	-1.28571700
C	5.70023200	4.91927500	-1.29054200
H	3.69395200	5.51309400	-0.93729500
C	6.13984600	2.56302400	-1.49311700
C	6.60352800	3.85255000	-1.49722000
H	6.07356400	5.93678100	-1.23730200
H	6.82444500	1.72735900	-1.61090500
H	7.66093000	4.05564700	-1.63084800
N	0.09833000	2.54479600	0.50924800
C	1.37917500	4.07282500	-0.91052500
C	1.38602000	5.25407400	-1.66599600
C	0.27592400	3.83751500	-0.07205300
C	0.40118400	6.21908800	-1.50030000
H	2.16613300	5.40488800	-2.40383600
C	-0.68621800	4.82636800	0.13283700
C	-0.61619300	6.02283700	-0.56757900
H	0.42783900	7.12393900	-2.09790100
H	-1.49051900	4.63633400	0.83506600
H	-1.37644300	6.78121300	-0.41437700
S	0.20447200	2.41580400	2.21723300
O	-0.67073900	1.32862000	2.63017300
O	-0.02980600	3.75967400	2.72044100
C	1.89624900	1.97387400	2.49916600
C	2.23756600	0.65470100	2.77206900
C	2.86397300	2.95072700	2.26534700
C	3.58591500	0.30959500	2.80691600
H	1.45523500	-0.08034100	2.92804600
C	4.20070600	2.58014400	2.27832700
H	2.56849200	3.97445700	2.05230300
C	4.57836600	1.25661100	2.53757000
H	3.87434100	-0.71533300	3.02332600
H	4.96469300	3.32390500	2.06744300
C	6.03040600	0.86694900	2.48742900
H	6.39203900	0.91720600	1.45461400
H	6.64002600	1.54903700	3.08581500
H	6.18253400	-0.15149800	2.84807600

Int1R

C	3.56486100	-0.44993600	0.36286700
H	4.12340600	0.09894600	-0.39614200
C	2.28635900	1.60766500	0.17565300
C	3.12061700	2.39909700	-0.74024000
C	2.10850700	3.53014900	-0.60377400
C	1.30643600	2.60300600	0.24235100
C	-1.00163800	3.37796800	0.58938100
C	-0.87379700	4.67526200	0.09022000
H	0.10365400	5.08913500	-0.13913400
C	-2.02360900	5.42220100	-0.14319500
C	-3.39572200	3.62449600	0.61691800
C	-2.27072300	2.84939400	0.85331400
H	-2.35916400	1.82568200	1.21122400
N	2.36597200	0.34497400	0.61909700
H	1.71882400	0.01534500	1.33095200
N	0.09346700	2.54200400	0.82369600
H	-0.14802200	1.62149400	1.20797000
O	4.12375500	2.18001400	-1.39658400
O	2.00728800	4.65606300	-1.03167600
F	-0.82239600	6.97602000	-1.44254600
F	-1.69749300	7.68996800	0.39335900
F	-5.66983200	3.96195000	1.13001400
F	-5.20002000	2.38747000	-0.26454200
C	-3.29235200	4.91866000	0.11504300
H	-4.17680600	5.51615800	-0.07207100
C	-4.75763200	3.02953900	0.83097200
C	-1.87554500	6.83784000	-0.62836900
F	-2.96416100	7.24894500	-1.29589600
F	-4.75600000	2.12837500	1.82943100
C	-1.63194100	-3.32130100	-2.26833300
C	3.24174200	-1.84114500	-0.20641400
C	4.50221800	-2.50500000	-0.81162000
H	2.79657000	-2.45619000	0.57419500
C	2.55542600	-0.87009900	-2.39885900
C	1.95344700	-3.16045600	-1.80198500
C	4.33037200	-2.63175600	-2.33250900
H	5.38434900	-1.89562900	-0.58550000
H	4.66046700	-3.48744400	-0.36162900
H	1.77812700	-0.99743100	-3.15660100
H	2.51901700	0.16085600	-2.04457800
C	3.95986100	-1.25424700	-2.90272900
C	3.17725700	-3.60034900	-2.64297000

H	1.02536200	-3.14111000	-2.37265700
H	1.79194100	-3.80002900	-0.92975200
H	5.25862300	-2.98187100	-2.79074000
H	3.96655500	-1.27397200	-3.99522700
H	4.69024300	-0.50217800	-2.58483700
H	2.91530200	-3.48099400	-3.70375300
N	2.18193900	-1.77842700	-1.27107700
C	3.45986900	-5.06354100	-2.41584900
H	2.58338900	-5.70830100	-2.48528200
C	4.64222400	-5.61205500	-2.15361600
H	5.55300300	-5.02609400	-2.06967300
H	4.74004700	-6.68320700	-2.01430700
C	4.41361700	-0.60911600	1.61568600
C	5.70647500	-0.01860100	1.73112600
C	3.92095600	-1.33382800	2.67645300
C	6.29736100	0.79608800	0.72436000
C	6.42220100	-0.27126500	2.93624200
C	4.71432800	-1.49901600	3.83312500
H	2.92755100	-1.77622700	2.63913700
C	7.55548200	1.31956100	0.91677800
H	5.74329600	1.05059600	-0.16967900
C	7.72761000	0.27668800	3.08765000
H	4.32524600	-2.07318200	4.67134100
C	8.28336200	1.04551600	2.10823500
H	8.25486700	0.06389600	4.01132000
H	9.27326900	1.47676600	2.21077500
N	5.92430300	-1.00644400	3.96548100
O	8.19450000	2.11863900	0.03309700
C	7.50154100	2.45162900	-1.15854400
H	8.16558700	3.10509500	-1.72217700
H	7.28103000	1.55374900	-1.74832800
H	6.56036100	2.96807000	-0.94268900
O	-1.17232000	-2.52668300	-3.25029700
H	1.22881900	-1.48579700	-0.86646200
C	-2.03922500	-1.71884300	-4.05004100
H	-1.78827100	-0.66799000	-3.89465700
H	-1.86065900	-1.99828800	-5.08985300
H	-3.08763400	-1.88930900	-3.79879800
O	-0.95084000	-4.26293900	-1.92871200
C	-2.96292600	-3.08769500	-1.61155100
C	-3.36969400	-1.88932800	-1.04477700
C	-3.73891500	-4.26723800	-1.46057300
C	-4.56131400	-1.88030400	-0.24250600
C	-4.92266600	-4.24694500	-0.78018900

H	-3.36833300	-5.18449700	-1.90576000
C	-4.96895000	-0.72988900	0.48579600
C	-5.34898100	-3.06071800	-0.13039500
H	-5.52864700	-5.14473100	-0.69338200
C	-6.10473000	-0.74074800	1.25419600
H	-4.35563100	0.16125900	0.44905300
C	-6.52698700	-3.03994500	0.66128200
C	-6.90023500	-1.90697400	1.33790400
H	-6.38329100	0.15041700	1.80651400
H	-7.12217200	-3.94690500	0.72771700
H	-7.80012100	-1.90475300	1.94477100
N	-0.44325400	-1.37964900	-0.47098700
C	-2.65497900	-0.60756900	-1.31357100
C	-3.39179100	0.42158500	-1.92153400
C	-1.27345400	-0.42155200	-1.10476700
C	-2.78948300	1.58812500	-2.37430900
H	-4.46020600	0.28072500	-2.06088700
C	-0.67406300	0.73800500	-1.61413000
C	-1.40966800	1.73248400	-2.24683000
H	-3.38851000	2.36634100	-2.83526300
H	0.39885800	0.84766300	-1.48636300
H	-0.91042500	2.62663800	-2.61001800
S	-0.42596400	-1.39331900	1.11782000
O	-0.57783800	-0.03982500	1.72514400
O	0.81809700	-2.08821300	1.51337300
C	-1.77247600	-2.34844400	1.77416500
C	-2.68160900	-1.77497400	2.65155300
C	-1.88458400	-3.68060000	1.37390300
C	-3.73376500	-2.55277700	3.13279500
H	-2.57054900	-0.73455700	2.93716200
C	-2.92987700	-4.43943500	1.87286000
H	-1.18676800	-4.09491100	0.65022500
C	-3.87306600	-3.88525300	2.75085600
H	-4.46749200	-2.10781400	3.79899500
H	-3.04095200	-5.47252100	1.55460600
C	-5.01850200	-4.72598200	3.24781400
H	-5.78883900	-4.10906100	3.71547600
H	-5.47693100	-5.27500000	2.41918200
H	-4.67529800	-5.46217000	3.98193300

Int1S

C	3.20943600	-0.82850000	-0.24270400
H	3.44694900	-1.41505700	0.64705800
C	1.01557600	-1.77822100	0.19139800

C	1.30231600	-2.85140000	1.15280800
C	-0.16559500	-3.25418400	1.04909900
C	-0.33995100	-2.09947000	0.13418500
C	-2.62022400	-1.87638500	-0.78595800
C	-3.09230700	-3.13918900	-0.42294000
H	-2.46724100	-3.82545100	0.14140700
C	-4.39712300	-3.49116000	-0.75642200
C	-4.74910000	-1.37556100	-1.79411500
C	-3.46208000	-0.98546000	-1.46870100
H	-3.09908200	0.00772700	-1.71658200
N	1.75888600	-0.81133300	-0.34985700
H	1.36469200	-0.18654700	-1.05290900
N	-1.33223800	-1.43697600	-0.50281200
H	-1.13260700	-0.47377500	-0.79267000
O	2.27661100	-3.19740800	1.79273000
O	-0.86522000	-4.11108000	1.54521400
F	-5.33370100	-4.67067800	1.04421700
F	-4.01354000	-5.76628300	-0.26137100
F	-6.88438200	-0.39932000	-1.95611000
F	-5.19617700	0.83117700	-2.51173000
C	-5.23930600	-2.63343600	-1.44961300
H	-6.25530300	-2.92377100	-1.69180700
C	-5.66013200	-0.42524200	-2.51607600
C	-4.93253100	-4.79360700	-0.23734000
F	-5.99419500	-5.21916400	-0.93667400
F	-5.83618400	-0.78105000	-3.79847800
C	0.31503600	0.52577600	2.95121500
C	3.77062600	0.58961400	-0.08148700
C	5.29572300	0.60894500	0.10242900
H	3.47944700	1.18335000	-0.95181300
C	3.76072900	0.88710300	2.39250500
C	3.38760700	2.79320900	0.89499200
C	5.66332900	1.77456600	1.03403500
H	5.63909200	-0.33421900	0.54624000
H	5.76368200	0.69494100	-0.87887100
H	3.12305300	1.28922900	3.17909200
H	3.67844200	-0.19870800	2.43895800
C	5.21412300	1.39215500	2.45133400
C	4.89350800	3.05029700	0.63659900
H	3.01460000	3.27705600	1.80047400
H	2.75078800	3.09483000	0.06195600
H	6.74033700	1.95890700	1.01560000
H	5.29472200	2.25588000	3.11871900
H	5.85493300	0.60484000	2.85557700

H	5.20420800	3.85052000	1.32091900
N	3.15776300	1.32354900	1.08505100
C	5.14352300	3.54386100	-0.76885200
H	4.38491600	4.22159400	-1.16148000
C	6.20519400	3.26929000	-1.52135500
H	7.00592200	2.61827700	-1.18245800
H	6.31570400	3.70172400	-2.50974900
C	3.85495200	-1.44521000	-1.48050300
C	4.62962100	-2.63986100	-1.42889300
C	3.68666300	-0.82257000	-2.69681700
C	4.80581900	-3.41667600	-0.24965300
C	5.23327800	-3.06799100	-2.64701100
C	4.31875100	-1.34828800	-3.84391900
H	3.06804000	0.06806000	-2.78874500
C	5.56080100	-4.56629500	-0.29360800
H	4.28771100	-3.14721600	0.66119000
C	6.03210400	-4.24664200	-2.64047400
H	4.19020600	-0.85068300	-4.80276300
C	6.19672300	-4.97465200	-1.49967200
H	6.48883100	-4.54615500	-3.57774700
H	6.78995900	-5.88248300	-1.48026400
N	5.08150500	-2.41758000	-3.83043600
O	5.75866300	-5.39329500	0.75720200
C	5.09527000	-5.07082500	1.96918600
H	5.33398400	-5.87304400	2.66591900
H	5.45715400	-4.11671400	2.37048800
H	4.01193800	-5.00511100	1.82576500
O	0.69613000	1.59452400	3.66609200
H	2.12507100	1.18379800	1.09028400
C	-0.18493600	2.27130000	4.56738500
H	-1.15947200	1.78264300	4.61371500
H	0.28840400	2.24340900	5.55029000
H	-0.30344200	3.30283800	4.23186100
O	1.18282900	-0.22245600	2.55079900
C	-1.12143700	0.22688500	2.65341200
C	-2.00097800	1.12605400	2.07299200
C	-1.49292600	-1.12545500	2.88352700
C	-3.29836300	0.65198600	1.67464800
C	-2.75258000	-1.56171200	2.59196300
H	-0.75776800	-1.80236200	3.30781500
C	-4.21478900	1.46806900	0.95542100
C	-3.68671600	-0.68326900	1.98282000
H	-3.03617800	-2.59344000	2.78096200
C	-5.46057400	1.00238100	0.61717100

H	-3.91162500	2.46686500	0.66190500
C	-4.98699900	-1.13194600	1.63563800
C	-5.86171600	-0.30412900	0.98137100
H	-6.13664800	1.63088600	0.04651200
H	-5.25938600	-2.15821700	1.86692400
H	-6.84511600	-0.66261600	0.69496600
N	0.38958300	2.29809900	0.63528300
C	-1.68322500	2.58004400	1.98168700
C	-2.54110200	3.46109900	2.66044300
C	-0.52552600	3.09647000	1.36705200
C	-2.26401400	4.81673200	2.76799900
H	-3.43023900	3.05417500	3.13472600
C	-0.24709300	4.46583200	1.50712300
C	-1.09636100	5.32027500	2.19635000
H	-2.94284200	5.46941400	3.30674000
H	0.65192400	4.84528700	1.02930300
H	-0.85434500	6.37529600	2.27823700
S	0.05688200	2.13576800	-0.89782800
O	-1.15860700	1.33043600	-1.18579300
O	1.28675900	1.63699400	-1.56964600
C	-0.27753600	3.73481100	-1.61310000
C	-1.56505100	4.26063400	-1.56250000
C	0.77954600	4.47730000	-2.12894900
C	-1.78830800	5.54916500	-2.03250600
H	-2.37639100	3.66028900	-1.16271700
C	0.53899700	5.76346800	-2.60019400
H	1.77021400	4.03545600	-2.17866100
C	-0.74377900	6.31720100	-2.55654500
H	-2.79012800	5.96771600	-1.99304800
H	1.35742700	6.34595600	-3.01450700
C	-1.00494700	7.69946900	-3.09486300
H	-1.78636000	8.20532900	-2.52292900
H	-0.10225200	8.31360100	-3.06681600
H	-1.33992500	7.64929800	-4.13598200

TS2R

C	3.51971800	-0.43830100	0.37811200
H	4.06594200	0.10069000	-0.39654500
C	2.26987700	1.64168600	0.21184200
C	3.09590200	2.42563400	-0.72314400
C	2.08162900	3.55596100	-0.58995400
C	1.29198000	2.63819600	0.27329100
C	-1.01372900	3.42224600	0.60856000
C	-0.86813400	4.74076600	0.16672900

H	0.11508600	5.16375900	-0.01087600
C	-2.00763300	5.49954700	-0.06344300
C	-3.40757600	3.67737000	0.57975600
C	-2.28901100	2.88888200	0.81401300
H	-2.39041300	1.85422900	1.13557600
N	2.34518800	0.38464300	0.66628000
H	1.72048100	0.07388400	1.40440700
N	0.07531300	2.58093800	0.85249400
H	-0.17535300	1.66582000	1.23558000
O	4.08841200	2.20212500	-1.39114800
O	1.97072500	4.67550800	-1.03287900
F	-0.62518300	7.36584700	-0.48692300
F	-2.66935000	7.74891100	0.11043500
F	-5.71173600	3.99078700	0.96766100
F	-5.14307300	2.38781900	-0.35865300
C	-3.28675300	4.98982300	0.14021400
H	-4.16410100	5.60344900	-0.03121500
C	-4.77043200	3.06842900	0.73998200
C	-1.87817300	6.90808500	-0.57417400
F	-2.24963000	6.99511300	-1.86162700
F	-4.80391300	2.19255200	1.76088000
C	-1.61053300	-3.39642400	-2.26864800
C	3.16231700	-1.82585000	-0.18369000
C	4.43135300	-2.49448900	-0.77816500
H	2.73843000	-2.43506200	0.61375300
C	2.48075000	-0.86997500	-2.34960700
C	1.89980200	-3.14330100	-1.76339400
C	4.26947200	-2.61925400	-2.29893000
H	5.31622700	-1.89078600	-0.54656600
H	4.58419200	-3.47848400	-0.32857000
H	1.70847000	-0.98410700	-3.11629300
H	2.45681200	0.16569900	-2.00545700
C	3.88697200	-1.24552900	-2.86776100
C	3.12121800	-3.58929700	-2.60913400
H	0.98140300	-3.14820200	-2.34817000
H	1.74307200	-3.79705300	-0.89928500
H	5.20168800	-2.96369700	-2.75500100
H	3.88855500	-1.26829900	-3.96090500
H	4.61747200	-0.48924600	-2.55787000
H	2.85591200	-3.46945500	-3.66955500
N	2.09285200	-1.76619300	-1.22737300
C	3.40395000	-5.05192700	-2.38629600
H	2.52748200	-5.69664500	-2.46254900
C	4.58401200	-5.60238100	-2.11615800

H	5.49340200	-5.01537500	-2.02433500
H	4.68147400	-6.67385400	-1.97782500
C	4.39966000	-0.59783800	1.61046700
C	5.69991700	-0.01609500	1.69401300
C	3.93145200	-1.31617600	2.68655600
C	6.27531500	0.78764100	0.66944000
C	6.44333100	-0.26945900	2.88227100
C	4.75098300	-1.48191200	3.82474400
H	2.93724400	-1.75633800	2.67476100
C	7.54368600	1.29808000	0.82781800
H	5.70340300	1.04049800	-0.21367200
C	7.75704700	0.26632700	2.99950900
H	4.37864400	-2.05153800	4.67375400
C	8.29729800	1.02381400	2.00281200
H	8.30389800	0.05166800	3.91128600
H	9.29424300	1.44431000	2.07864200
N	5.96634900	-0.99638900	3.92739700
O	8.17098600	2.08344100	-0.07692000
C	7.45378500	2.41579800	-1.25416700
H	8.11302200	3.05450400	-1.83995600
H	7.20533300	1.51639700	-1.83021500
H	6.52601300	2.94788300	-1.01963400
O	-1.12759000	-2.60168100	-3.23850400
H	0.89893700	-1.50570100	-0.76950500
C	-1.97150700	-1.78083300	-4.04963600
H	-1.68925800	-0.73624900	-3.90555600
H	-1.79977200	-2.07662600	-5.08592600
H	-3.02471800	-1.91772900	-3.79766500
O	-0.95293800	-4.35347500	-1.92771800
C	-2.93501800	-3.13345600	-1.61352300
C	-3.30921700	-1.92773200	-1.04240600
C	-3.73895800	-4.29462300	-1.46842800
C	-4.50699600	-1.88767400	-0.24814400
C	-4.92105700	-4.24724900	-0.78774300
H	-3.38989000	-5.21833800	-1.91740600
C	-4.89874300	-0.72650500	0.47265000
C	-5.32009400	-3.05137200	-0.13808600
H	-5.54937300	-5.12976700	-0.70338300
C	-6.03922900	-0.71433700	1.23473900
H	-4.27373900	0.15684100	0.43167200
C	-6.49976400	-3.00760400	0.64985100
C	-6.85465700	-1.86614000	1.32170100
H	-6.30794400	0.18483300	1.77837800
H	-7.11175800	-3.90336200	0.71529800

H	-7.75659900	-1.84537400	1.92503200
N	-0.36204200	-1.43114200	-0.40781300
C	-2.56160100	-0.66248900	-1.30846700
C	-3.28225000	0.37231600	-1.92746100
C	-1.18273100	-0.47865600	-1.08677100
C	-2.66914500	1.53594500	-2.37192100
H	-4.34927900	0.23709900	-2.08040500
C	-0.57148400	0.68175300	-1.57345400
C	-1.29250000	1.67822900	-2.21986200
H	-3.25888300	2.31607900	-2.84137800
H	0.49474500	0.79836200	-1.41614600
H	-0.78496400	2.57325900	-2.56856300
S	-0.42644600	-1.39510800	1.21199600
O	-0.67107500	-0.02853800	1.73247900
O	0.81797700	-2.02837500	1.67644900
C	-1.77911200	-2.38196700	1.78396000
C	-2.70261400	-1.83799600	2.66587700
C	-1.86081700	-3.70792700	1.35740600
C	-3.74682700	-2.64090400	3.11989600
H	-2.61180200	-0.80148100	2.97152700
C	-2.90035400	-4.49083800	1.83076200
H	-1.15065900	-4.09902100	0.63286000
C	-3.86119200	-3.96728500	2.70854200
H	-4.49439800	-2.22013000	3.78599500
H	-2.99227300	-5.51863400	1.49081500
C	-5.00268200	-4.83226100	3.17011200
H	-4.65721700	-5.59107500	3.87942900
H	-5.77981800	-4.23756400	3.65461300
H	-5.45128000	-5.35547100	2.31982000

TS2S

C	3.00928400	-0.83149600	-0.48871700
H	3.14332800	-1.18381800	0.53513700
C	0.80757300	-1.82431900	-0.35203100
C	1.09769200	-3.05884700	0.40507000
C	-0.37666800	-3.41686600	0.27004200
C	-0.55692000	-2.11596000	-0.41387500
C	-2.91540700	-1.68494500	-1.03509100
C	-3.40463300	-2.97758700	-0.83110900
H	-2.74924500	-3.77368900	-0.49065700
C	-4.76011500	-3.22491200	-1.02768400
C	-5.13421400	-0.95065600	-1.62286000
C	-3.79463900	-0.66438700	-1.42841300
H	-3.42381100	0.34831700	-1.54839100

N	1.57634300	-0.81022700	-0.74428400
H	1.19904800	-0.06517400	-1.32661300
N	-1.57888600	-1.34026400	-0.85969600
H	-1.38249800	-0.34937900	-0.99331000
O	2.06271000	-3.52131400	0.97026300
O	-1.07198900	-4.34400600	0.63065800
F	-5.56327400	-4.67233500	0.64005700
F	-4.42239300	-5.55907500	-0.96081000
F	-7.14294200	0.19254700	-1.17832500
F	-5.51522300	1.35216300	-1.99985800
C	-5.64262000	-2.23305800	-1.43120000
H	-6.69620800	-2.44574100	-1.57344000
C	-6.08670200	0.14159100	-2.01406100
C	-5.29334500	-4.58379100	-0.67796800
F	-6.43622800	-4.85771100	-1.32466800
F	-6.58765400	-0.05173900	-3.24433100
C	0.74453000	-0.04807700	2.79115500
C	3.59940200	0.58013600	-0.63405200
C	5.13486200	0.57515200	-0.46605100
H	3.34205600	0.94302600	-1.63839800
C	3.64313800	1.48404400	1.65683600
C	3.34340700	2.94299000	-0.24589500
C	5.56978900	1.91951200	0.13502500
H	5.44015100	-0.23352800	0.20943200
H	5.60074200	0.37814000	-1.43282200
H	3.03955200	2.09153300	2.33762600
H	3.53015200	0.45027500	1.98368900
C	5.11829800	1.93523500	1.60360300
C	4.85105500	3.07732500	-0.58245500
H	3.03729700	3.66926000	0.51215000
H	2.71297800	3.08743000	-1.12801400
H	6.65439800	2.04043100	0.06872300
H	5.23912700	2.93932500	2.02392300
H	5.73457500	1.25610900	2.19891600
H	5.20203800	4.01906200	-0.13924500
N	3.02699100	1.59686900	0.30439700
C	5.09835800	3.16743600	-2.06944100
H	4.34899400	3.72869700	-2.62964800
C	6.14525900	2.67282500	-2.72426400
H	6.93245800	2.11708000	-2.22267500
H	6.25596400	2.81701600	-3.79358300
C	3.74182500	-1.74402300	-1.47227800
C	4.71568300	-2.70129600	-1.06217300
C	3.50467700	-1.59671600	-2.81910300

C	5.00374100	-3.00058500	0.29815000
C	5.42000900	-3.39121500	-2.09071400
C	4.24835900	-2.34958500	-3.75248300
H	2.75135700	-0.89985400	-3.17654600
C	5.95662200	-3.94012100	0.60976400
H	4.41252100	-2.54610800	1.07991700
C	6.42210800	-4.33466900	-1.72518600
H	4.06020800	-2.22156700	-4.81662500
C	6.69030300	-4.59980700	-0.41513800
H	6.94935400	-4.83880100	-2.52788200
H	7.43888400	-5.32652200	-0.11780100
N	5.18702700	-3.20459900	-3.41715800
O	6.26918000	-4.32642200	1.87015600
C	5.47452500	-3.80028400	2.91785700
H	5.81836600	-4.28255500	3.83190800
H	5.60681300	-2.71501500	3.00660000
H	4.41406500	-4.02081100	2.75338200
O	1.19397000	0.86582200	3.66566800
H	1.72444500	1.70003500	0.44052500
C	0.39681200	1.36337800	4.74096700
H	-0.59824500	0.91532600	4.74738100
H	0.91834300	1.11352800	5.66656300
H	0.31551600	2.44621100	4.63633000
O	1.56141200	-0.71446000	2.19649600
C	-0.72395400	-0.26578400	2.56206200
C	-1.62172100	0.73171500	2.21631700
C	-1.12892200	-1.62508500	2.62841800
C	-2.97863000	0.36794500	1.91870900
C	-2.43166800	-1.97503500	2.41552600
H	-0.38139900	-2.37831400	2.86089200
C	-3.93989700	1.31436800	1.46647600
C	-3.38862300	-0.98689500	2.06814200
H	-2.73824000	-3.01572500	2.47171000
C	-5.24292400	0.94534100	1.24529500
H	-3.62645500	2.33590400	1.28168000
C	-4.74341200	-1.33970300	1.83320200
C	-5.65641000	-0.39214500	1.45235800
H	-5.95927300	1.67574800	0.88340400
H	-5.03156400	-2.38295300	1.93091100
H	-6.68670600	-0.67094400	1.25741600
N	0.50117900	2.03874900	0.53539600
C	-1.23125700	2.16861500	2.30051600
C	-1.92387200	2.97375500	3.21725900
C	-0.14217600	2.72910600	1.60763300

C	-1.52155500	4.27351800	3.49872300
H	-2.77401300	2.54564200	3.74135900
C	0.27644300	4.02697700	1.92349800
C	-0.39253000	4.79339700	2.87044200
H	-2.07302500	4.86674000	4.22048900
H	1.10930700	4.44687700	1.36697000
H	-0.05313600	5.80021500	3.09059500
S	-0.22883900	2.18712200	-0.89608700
O	-1.58284800	1.60969300	-0.86741100
O	0.71497300	1.63817100	-1.89082600
C	-0.44640500	3.90887000	-1.28290200
C	-1.47965400	4.62109000	-0.67551700
C	0.43428900	4.52810500	-2.16348600
C	-1.61195200	5.97641400	-0.94561200
H	-2.16570700	4.11692300	-0.00119000
C	0.28181800	5.88581600	-2.42697300
H	1.20520000	3.94405500	-2.65531600
C	-0.73584700	6.62735700	-1.82176300
H	-2.41112200	6.53972500	-0.47236900
H	0.95911900	6.37500200	-3.12109800
C	-0.91359900	8.09014600	-2.13180600
H	-1.22886300	8.64583800	-1.24557900
H	0.01204000	8.53131000	-2.50698000
H	-1.68341200	8.22769800	-2.89772000

Int2R

C	3.57101200	-0.48786300	0.34782900
H	4.13161700	0.06213800	-0.40852800
C	2.31909700	1.59700600	0.18782100
C	3.16662900	2.39759000	-0.71945200
C	2.14808200	3.52357100	-0.59293700
C	1.34938200	2.60276800	0.25394100
C	-0.94921400	3.41428100	0.60288700
C	-0.79090000	4.72490600	0.14124900
H	0.19613300	5.13069300	-0.05520000
C	-1.92173600	5.49825900	-0.08293900
C	-3.33979800	3.70763200	0.60486200
C	-2.23043600	2.90472500	0.83376300
H	-2.34637300	1.87674000	1.17025200
N	2.39637900	0.34096700	0.63543300
H	1.75479300	0.01122900	1.34954700
N	0.13179400	2.56155500	0.84120700
H	-0.12626700	1.65864800	1.23967500
O	4.18048200	2.19320500	-1.35734900

O	2.04679500	4.64772200	-1.02895800
F	-0.51719200	7.33809900	-0.54793000
F	-2.54817600	7.75922300	0.06780700
F	-5.62628600	4.06327600	1.05223900
F	-5.12462600	2.47302700	-0.31482200
C	-3.20557100	5.01208100	0.14548600
H	-4.07570000	5.63709400	-0.02178800
C	-4.71130300	3.12503200	0.78723000
C	-1.77734200	6.89785100	-0.61450300
F	-2.16287000	6.97265800	-1.89861000
F	-4.74253800	2.23093700	1.79257000
C	-1.69891400	-3.33980800	-2.26883900
C	3.20544600	-1.85882800	-0.24763000
C	4.48758900	-2.55249800	-0.79299600
H	2.75072500	-2.46840100	0.53599800
C	2.63312200	-0.88581000	-2.41205700
C	1.94817200	-3.11091000	-1.84656200
C	4.35759900	-2.69008000	-2.31664200
H	5.37884400	-1.96061900	-0.55161400
H	4.61531600	-3.53272600	-0.32677900
H	1.88640400	-0.97641400	-3.20798500
H	2.63041500	0.15812200	-2.08446100
C	4.04147400	-1.30392500	-2.89968200
C	3.18062700	-3.61895700	-2.65354400
H	1.04853900	-3.09321900	-2.46602300
H	1.73842700	-3.76748300	-0.99405200
H	5.28782700	-3.07385200	-2.74606600
H	4.07624100	-1.32840700	-3.99297400
H	4.79356800	-0.57748200	-2.56814900
H	2.95441500	-3.49701900	-3.72316800
N	2.17801400	-1.75375800	-1.30734000
C	3.40150500	-5.08830000	-2.41511100
H	2.50713500	-5.70187900	-2.53479700
C	4.54320500	-5.68291000	-2.07948100
H	5.46813100	-5.13054000	-1.94126400
H	4.59161800	-6.75657300	-1.93144300
C	4.42515100	-0.66568600	1.59523100
C	5.72223300	-0.08328300	1.71773600
C	3.93673100	-1.40118600	2.65052600
C	6.31907500	0.73352500	0.71613100
C	6.43874200	-0.34923200	2.91975600
C	4.72965000	-1.58081800	3.80501700
H	2.94582500	-1.84645000	2.60987700
C	7.58193100	1.24495000	0.90934100

H	5.76769300	0.99219600	-0.17804800
C	7.74718600	0.19008500	3.07427500
H	4.33965700	-2.16443100	4.63659200
C	8.30801000	0.96071800	2.09911100
H	8.27397200	-0.03286000	3.99586300
H	9.30146800	1.38352600	2.20289200
N	5.94002600	-1.09169400	3.94395600
O	8.22914000	2.04052500	0.02737400
C	7.54069000	2.37906000	-1.16525900
H	8.21390500	3.02108900	-1.73134700
H	7.30636500	1.48277600	-1.75192400
H	6.60744400	2.90988900	-0.95037900
O	-1.21095200	-2.55842000	-3.24527500
H	0.51493000	-1.52279900	-0.71104200
C	-2.04635500	-1.73054900	-4.05736100
H	-1.75890800	-0.68803000	-3.90801200
H	-1.86946700	-2.02201400	-5.09377100
H	-3.10210900	-1.86325000	-3.81314100
O	-1.04384500	-4.28911600	-1.90545900
C	-3.03189400	-3.06597200	-1.62751600
C	-3.40276100	-1.85871500	-1.05553900
C	-3.84882900	-4.21857500	-1.49102700
C	-4.59941400	-1.80984300	-0.26280500
C	-5.03457900	-4.16154500	-0.81589100
H	-3.50684300	-5.14475600	-1.94048900
C	-4.97359600	-0.65032400	0.46924700
C	-5.42422000	-2.96586900	-0.16038300
H	-5.67165100	-5.03841200	-0.73871000
C	-6.11160600	-0.63099300	1.23467200
H	-4.33604400	0.22423000	0.43670200
C	-6.60273600	-2.91352000	0.62906700
C	-6.94178300	-1.77336700	1.31162500
H	-6.36664600	0.26637100	1.78814500
H	-7.22615500	-3.80180200	0.68807800
H	-7.84265000	-1.74669100	1.91625900
N	-0.50466400	-1.48043700	-0.40386800
C	-2.63396800	-0.60400300	-1.30606600
C	-3.30885800	0.46729000	-1.91232600
C	-1.25457700	-0.47046800	-1.08807800
C	-2.63710200	1.60313200	-2.34659300
H	-4.38043000	0.38320800	-2.06827000
C	-0.57385400	0.65111600	-1.56383500
C	-1.25323800	1.68126100	-2.20369500
H	-3.18881600	2.41427000	-2.80978800

H	0.50074900	0.70195700	-1.41566800
H	-0.70839100	2.55261000	-2.55520200
S	-0.50662400	-1.41806400	1.25516300
O	-0.73154800	-0.04254800	1.73515500
O	0.74793100	-2.05630200	1.65952600
C	-1.87402100	-2.38634900	1.79664000
C	-2.79574300	-1.82897200	2.67295000
C	-1.97096700	-3.70615700	1.35382000
C	-3.85675200	-2.61804900	3.10965700
H	-2.69206900	-0.79587500	2.98627400
C	-3.02994900	-4.47244600	1.80996700
H	-1.26125400	-4.10163800	0.63119700
C	-3.98851300	-3.93917400	2.68484500
H	-4.60361600	-2.19063800	3.77187100
H	-3.13800400	-5.49468000	1.45892100
C	-5.14619600	-4.78943200	3.13155600
H	-4.81545000	-5.55751000	3.83781900
H	-5.91778600	-4.18700700	3.61499900
H	-5.59489000	-5.30079400	2.27446500

Int2S

C	3.03724700	-0.86252300	-0.50370300
H	3.14544200	-1.19415700	0.53023900
C	0.81103100	-1.80760700	-0.40841100
C	1.09150700	-3.06130100	0.32407200
C	-0.39131500	-3.38665500	0.21832800
C	-0.55989700	-2.07976500	-0.45409800
C	-2.92613700	-1.64096800	-1.05524300
C	-3.40883400	-2.93925800	-0.87206300
H	-2.75010800	-3.73809600	-0.54588800
C	-4.76316900	-3.19064300	-1.07089100
C	-5.15064600	-0.90973500	-1.62713700
C	-3.81274200	-0.61889200	-1.42704300
H	-3.44999800	0.39857400	-1.52737300
N	1.60734100	-0.81396900	-0.78817800
H	1.23525500	-0.01715700	-1.29813800
N	-1.58873000	-1.29635000	-0.88060300
H	-1.38300900	-0.31969800	-1.06315900
O	2.06205700	-3.55109300	0.85368300
O	-1.09721300	-4.30088700	0.59357800
F	-5.56360600	-4.66443100	0.57426700
F	-4.41066300	-5.52365900	-1.03306500
F	-7.17178100	0.20909100	-1.17512400
F	-5.54526200	1.39805300	-1.95680200

C	-5.65180900	-2.19800000	-1.45850500
H	-6.70419900	-2.41419800	-1.60421000
C	-6.10804100	0.18394800	-2.00211100
C	-5.28867900	-4.55765900	-0.74157500
F	-6.42725600	-4.83010900	-1.39635900
F	-6.59661800	0.01458400	-3.24100800
C	0.68237100	-0.08042100	2.78140400
C	3.64342000	0.54144600	-0.66330900
C	5.18116300	0.53607300	-0.50455400
H	3.39215500	0.88389900	-1.67847800
C	3.66228900	1.44595500	1.60275300
C	3.36642900	2.88212900	-0.29613200
C	5.60487500	1.88220100	0.10448400
H	5.49444800	-0.27096700	0.16931900
H	5.65022300	0.34521700	-1.47166700
H	3.06443000	2.06157300	2.28316800
H	3.54198300	0.41670000	1.94469000
C	5.14610300	1.88391100	1.57079000
C	4.88399500	3.04439800	-0.60565600
H	3.03639700	3.62325300	0.44006500
H	2.76405100	3.01858000	-1.20085700
H	6.68949000	2.01017100	0.04390300
H	5.27312600	2.88281700	2.00271500
H	5.75712200	1.19423600	2.16066300
H	5.22037400	3.98245300	-0.14257700
N	3.05624300	1.54926100	0.25478000
C	5.15386300	3.16037200	-2.08581800
H	4.41814500	3.74174800	-2.64456700
C	6.20013200	2.66388900	-2.74080000
H	6.97421500	2.08768500	-2.24210900
H	6.32322500	2.82634600	-3.80626500
C	3.76243000	-1.81377300	-1.45148200
C	4.74571500	-2.74684200	-1.00818100
C	3.51898600	-1.72390200	-2.80206000
C	5.04468900	-2.98521600	0.36181800
C	5.45000100	-3.47337400	-2.01043800
C	4.26299600	-2.50981300	-3.70735300
H	2.75952900	-1.04610200	-3.18140000
C	6.01217300	-3.89717400	0.70754000
H	4.45810200	-2.49607900	1.12621000
C	6.46299500	-4.39132300	-1.61125200
H	4.06883900	-2.42693300	-4.77501400
C	6.74406600	-4.59517300	-0.29291400
H	6.99018800	-4.92445000	-2.39507200

H	7.50304100	-5.29952300	0.03049600
N	5.20890800	-3.34452200	-3.34262700
O	6.34342400	-4.22068500	1.98158800
C	5.56502000	-3.64370300	3.01330000
H	5.93342400	-4.06767800	3.94643000
H	5.68479000	-2.55352500	3.03571600
H	4.50378700	-3.88541500	2.88609300
O	1.14141600	0.82661000	3.65883400
H	1.38504200	1.76652400	0.45992500
C	0.36449200	1.29005100	4.76323200
H	-0.63036900	0.84114400	4.77590900
H	0.90252900	1.01450200	5.67194300
H	0.28126100	2.37535700	4.69253900
O	1.49015700	-0.73432300	2.16288900
C	-0.79108900	-0.29525600	2.56825900
C	-1.69594600	0.70674200	2.25333800
C	-1.19337700	-1.65613500	2.60188900
C	-3.05190100	0.34954300	1.94989300
C	-2.49572600	-2.00381700	2.38168500
H	-0.44440300	-2.41378100	2.81273900
C	-4.01516500	1.30555700	1.52240200
C	-3.45635000	-1.01066800	2.06094800
H	-2.79823000	-3.04680800	2.40987200
C	-5.31485500	0.93627400	1.28354300
H	-3.70508200	2.33288300	1.36581200
C	-4.80906300	-1.36199000	1.81313900
C	-5.72356600	-0.40806600	1.45244300
H	-6.03282000	1.67291900	0.93776300
H	-5.09376900	-2.40832400	1.88324200
H	-6.75157500	-0.68554600	1.24393500
N	0.33906100	1.99401500	0.53760500
C	-1.30431500	2.14182100	2.35431700
C	-1.96530400	2.94592100	3.29328600
C	-0.23280300	2.70253400	1.64171600
C	-1.53788200	4.23933100	3.57002200
H	-2.80630500	2.52334700	3.83602200
C	0.21598500	3.99046900	1.94489700
C	-0.41932700	4.75364300	2.91814500
H	-2.06277300	4.83420500	4.31007800
H	1.04113500	4.40262000	1.37111700
H	-0.06307400	5.75388500	3.14034700
S	-0.38733900	2.22749500	-0.91464300
O	-1.77744400	1.78310400	-0.80793500
O	0.48708700	1.57810500	-1.90053300

C	-0.42831800	3.95824100	-1.29244300
C	-1.39609100	4.76402200	-0.69396900
C	0.51798100	4.48713800	-2.16493800
C	-1.39164400	6.12582700	-0.96352500
H	-2.13776500	4.32790000	-0.03158500
C	0.50180600	5.85302800	-2.42548800
H	1.23071700	3.83043600	-2.65220300
C	-0.44539300	6.68889200	-1.82809100
H	-2.13819900	6.76478800	-0.50093300
H	1.22971500	6.27447700	-3.11238900
C	-0.47773800	8.16199500	-2.13655800
H	-0.75973000	8.74317900	-1.25567000
H	0.49263500	8.51516200	-2.49070300
H	-1.21479200	8.37032100	-2.91865500

PR

C	-1.56722500	1.16580300	2.17622700
O	-2.71394300	1.48059800	1.56720000
H	-2.65182900	-1.60382100	0.77395600
C	-3.89669800	1.19151200	2.31394500
H	-3.92839000	1.79398100	3.22381100
H	-4.72869300	1.44059400	1.65831000
H	-3.92273300	0.13531300	2.59171600
O	-1.51456700	0.73832500	3.30809500
C	-0.34714800	1.42816500	1.35346400
C	-0.28055100	1.25331600	-0.01856100
C	0.79469500	1.82315400	2.09655400
C	0.98677600	1.41511700	-0.67305800
C	1.98572100	2.06471300	1.47290100
H	0.68873400	1.93521000	3.17019700
C	1.16857700	1.12334100	-2.05435600
C	2.11808800	1.84421500	0.07859100
H	2.85273600	2.39384700	2.03953300
C	2.39386800	1.27193700	-2.65281900
H	0.32779300	0.75506600	-2.63191800
C	3.36854900	2.00691400	-0.57348200
C	3.50700600	1.72823800	-1.90846800
H	2.51268300	1.03286700	-3.70465800
H	4.21836300	2.34643100	0.01301900
H	4.46948600	1.85024500	-2.39521200
N	-1.85064500	-1.20602800	0.28645000
C	-1.47489400	0.92992600	-0.85503000
C	-1.88967000	1.86297800	-1.81178900
C	-2.21433300	-0.25105200	-0.72124400

C	-3.01014400	1.63795700	-2.60326700
H	-1.32552700	2.78507400	-1.91700400
C	-3.32632800	-0.48728200	-1.52747200
C	-3.73345700	0.45708800	-2.46171200
H	-3.31759700	2.38483300	-3.32776800
H	-3.86418500	-1.42404400	-1.41602600
H	-4.60431700	0.26618500	-3.07969500
S	-0.90140600	-2.52450900	-0.18881600
O	-1.03498300	-2.76228000	-1.61969600
O	-1.25934000	-3.56737100	0.76569600
C	0.74666300	-1.97058800	0.12436400
C	1.66907000	-1.96569900	-0.91217400
C	1.09087600	-1.58987700	1.42115900
C	2.97337000	-1.55792100	-0.64178400
H	1.35970600	-2.25849700	-1.90952100
C	2.39643500	-1.20264900	1.67058400
H	0.33933800	-1.56933700	2.20566700
C	3.35193100	-1.17755800	0.64452100
H	3.70053100	-1.51899600	-1.44754500
H	2.67948300	-0.88764800	2.67096800
C	4.75566900	-0.72260200	0.93951200
H	5.30955100	-0.52539700	0.01920900
H	4.74433700	0.19441400	1.53693800
H	5.29909300	-1.48102600	1.51193500

PS

S	0.15002300	-2.49430900	-1.33725900
O	-0.13073100	-2.42143600	-2.76423500
O	0.35761900	-3.77196300	-0.67224900
N	-1.14077100	-1.75056000	-0.62712000
H	-1.63739100	-1.09858200	-1.22622000
C	-1.50970400	-0.09555500	2.67488900
O	-4.09639000	1.96035000	-0.88017000
O	-3.46155400	-0.17446900	-0.65305900
C	-1.83409200	1.57612700	-0.38412200
C	1.58478600	-1.50167500	-1.00151300
C	-1.26178900	-1.54826800	0.77133800
C	-0.95298100	0.96672700	0.49478400
C	-0.26414800	3.35041900	-0.87377500
H	0.00475000	4.26201600	-1.39938100
C	-1.67092000	-1.19878100	3.50621700
H	-1.84295100	-1.05444100	4.56763600
C	0.32837700	1.57835500	0.72016300
C	-1.41250400	-2.65652200	1.60543300

H	-1.39100400	-3.64399600	1.15874300
C	-3.19073900	1.00726300	-0.63856600
C	-1.28147900	-0.24954500	1.30424900
C	-1.60044800	-2.48243500	2.97139600
C	2.54226500	-1.97220400	-0.10831900
H	2.39029300	-2.92722200	0.38245600
C	0.66667100	2.77811500	0.03132800
C	3.68371900	-1.21137200	0.11354400
H	4.44081900	-1.57009200	0.80565600
C	-1.48525400	2.77272500	-1.06684200
H	-2.20914900	3.21550800	-1.74085300
C	1.74355900	-0.28619100	-1.66241600
H	0.98528600	0.06394800	-2.35739900
C	1.28773700	1.02212600	1.61248400
H	1.06609800	0.08742300	2.11490200
C	3.87360500	0.00873200	-0.54181900
C	1.92336700	3.39302500	0.26969200
H	2.16076800	4.31019000	-0.26211700
C	2.81134100	2.84865400	1.16056900
H	3.76639900	3.33060000	1.34569400
C	2.88807200	0.46288200	-1.42330900
H	3.01941800	1.41755000	-1.92424000
C	2.48820800	1.64745600	1.83307500
H	3.20563300	1.20699900	2.51880800
C	-5.41296000	1.48493000	-1.18070000
H	-6.00984000	2.37376300	-1.37321200
H	-5.81314500	0.92620900	-0.33303700
C	5.12085600	0.81615400	-0.29962400
H	5.98375900	0.35073300	-0.78653400
H	5.34286100	0.88113700	0.76972800
H	5.01580100	1.83046000	-0.69080400
H	-5.38994000	0.83555800	-2.05755000
H	-1.53229800	0.90826100	3.08962200
H	-1.71596100	-3.34986000	3.61285200

TS3

S	2.32264300	-0.82589200	1.42082900
O	2.36037900	-2.27855800	1.31241800
O	2.78639600	-0.21425900	2.66052000
N	0.72188800	-0.46222100	1.07081400
H	0.14380600	-1.19718500	1.47818700
C	-1.74407400	2.27692300	1.12751300
O	-0.41066000	-3.10887000	0.26407300
O	-0.11671900	-1.72231700	-1.48063900
C	-1.95490200	-1.33711900	-0.01974300

C	3.18253700	-0.05769000	0.07789000
C	0.15663500	0.83900400	1.09324400
C	-2.15137600	0.04487200	0.10487100
C	-4.34687000	-1.77608000	-0.11350700
H	-5.18846500	-2.46098300	-0.08580700
C	-0.98677000	3.32965300	1.61149200
H	-1.47328000	4.25939700	1.88483600
C	-3.46630200	0.50342300	-0.32653900
C	0.93401500	1.91381600	1.54188100
H	1.97343000	1.75417300	1.79240200
C	-0.69763300	-2.02457200	-0.46526400
C	-1.20253900	1.03733500	0.71593900
C	0.38363800	3.16709800	1.75581700
C	4.29897400	0.71824400	0.36697200
H	4.58737000	0.88611100	1.39927700
C	-4.56820500	-0.39956700	-0.35973400
C	5.02009600	1.27020200	-0.68729700
H	5.89312900	1.87995200	-0.47522300
C	-3.06201000	-2.23007100	-0.05575700
H	-2.84993800	-3.29344400	-0.03269200
C	2.77354600	-0.30097500	-1.23243700
H	1.89171900	-0.90345900	-1.43305800
C	-3.69187800	1.79951400	-0.87927100
H	-2.84977700	2.46481400	-1.02521800
C	4.63564500	1.05268500	-2.01150800
C	-5.85947200	0.06223000	-0.72943800
H	-6.68464000	-0.64340400	-0.69537000
C	-6.05000700	1.34269900	-1.17435100
H	-7.03506400	1.68089600	-1.47798800
C	3.50633100	0.26352800	-2.26646400
H	3.19689800	0.09238400	-3.29369300
C	-4.93554500	2.20172600	-1.29705900
H	-5.05691700	3.18366300	-1.74243800
C	0.56662800	-3.99369100	-0.30613300
H	0.75854000	-4.74558400	0.45588600
H	1.48058200	-3.45124000	-0.53837800
C	5.40678500	1.65654400	-3.15493200
H	5.74284500	0.88061500	-3.84852300
H	6.28295000	2.20252600	-2.80070000
H	4.77770800	2.34977500	-3.72114500
H	0.15487900	-4.45063300	-1.20892800
H	-2.81329300	2.41737600	1.08523700
H	1.01338300	3.97771500	2.10627500
